# Acyl Group Migration in Pyranosides as Studied by Experimental and Computational Methods

**DOI:** 10.1002/chem.202200499

**Published:** 2022-05-11

**Authors:** Robert Lassfolk, Manuel Pedrón, Tomás Tejero, Pedro Merino, Johan Wärnå, Reko Leino

**Affiliations:** ^1^ Laboratory of Molecular Science and Engineering Åbo Akademi University 20500 Turku Finland; ^2^ Institute of Biocomputation & Physics of Complex Systems (BIFI) University of Zaragoza 50009 Zaragoza Spain; ^3^ Institute of Chemical Synthesis & Homogeneous Catalysis (ISQCH) University of Zaragoza 50009 Zaragoza Spain; ^4^ Laboratory of Industrial Chemistry and Reaction Engineering Åbo Akademi University 20500 Turku Finland

**Keywords:** acyl group migration, carbohydrates, computational chemistry, NMR spectroscopy, reaction mechanism

## Abstract

Acyl group migration affects the synthesis, isolation, manipulation and purification of all acylated organic compounds containing free hydroxyl groups, in particular carbohydrates. While several isolated studies on the migration phenomenon in different buffers have been reported, comprehensive insights into the overall migration process in different monosaccharides under similar conditions have been lacking. Here, we have studied the acyl migration in different monosaccharides using five different acyl groups by a combination of experimental, kinetic and theoretical tools. The results show that the anomeric configuration in the monosaccharide has a major influence on the migration rate, together with the relative configurations of the other hydroxyl groups and the nature of the migrating acyl group. Full mechanistic model, based on computations, demonstrates that the acyl migration proceeds through an anionic stepwise mechanism with linear dependence on the [OH^−^] and the pK_a_ of the hydroxyl group toward which the acyl group is migrating.

## Graphical abstract

**A comprehensive experimental and computational study** on acyl migration in different monosaccharides with five different acyl groups, under similar conditions, provides conclusive evidence for the anionic, stepwise migration mechanism, which depends on the concentration of the hydroxyl anion and pK_a_ of the receiving hydroxyl group, together with the anomeric and relative configurations of the carbohydrate in question.

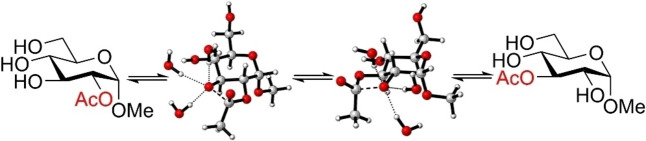

## Introduction

**Scheme 1:**
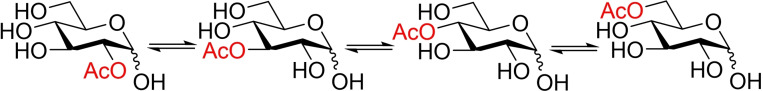
Acyl migration in glucopyranoside.

Acyl group migration is a well‐known phenomenon affecting the synthesis, isolation and purification of organic molecules which contain multiple hydroxyl groups, in particular carbohydrates,[^1^, ^2^, ^3^] where the migration was first reported by Fischer.^[4]^ Several detailed studies on acyl group migration have been described in the literature, mostly in monosaccharides, where the migration commonly is reported to take place between two adjacent hydroxyl groups. The most preferred position is the primary one, resulting in an overall clockwise migration process from O2, the typical starting point, to O6 in pyranosides (Scheme 1).[^5^, ^6^, ^7^, ^8^] Unsuccessful attempts to induce acetyl migration directly from O2 to O6 in a mannopyranoside, by blocking the O3 and O4 positions, have been reported,^[9]^ suggesting that the two hydroxyl groups must be sufficiently close in space to allow for the migration to take place.

Several factors influence the rate of migration in different ways, including the stereochemical relationship of the two hydroxyl groups, the acyl groups involved and the reaction solvent. The stereochemical relationship between the hydroxyl groups depends on the carbohydrate in question, with *cis* relationship being more prone to induce migration compared to *trans*,[^8^, ^10^] due to less ring strain in the five membered transition states. Several different mechanisms have been suggested for the migration process in the literature.[^11^, ^12^, ^13^] Experimental support, however, indicates the mechanism to be base catalyzed, requiring deprotonation as the first step, followed by nucleophilic attack at the carbonyl carbon.[^14^, ^15^, ^16^, ^17^, ^18^] Most of the earlier studies have been carried out in buffers and, with the deprotonation being a key step in the migration process, the pH of the buffer used heavily influences the rate of migration. This was clearly observed in an earlier study where the pH of 7, 7.4 and 8 were used to study the *R*‐ and *S*‐naproxen migration in β‐glucuronides.^[17]^

**Figure 1:**
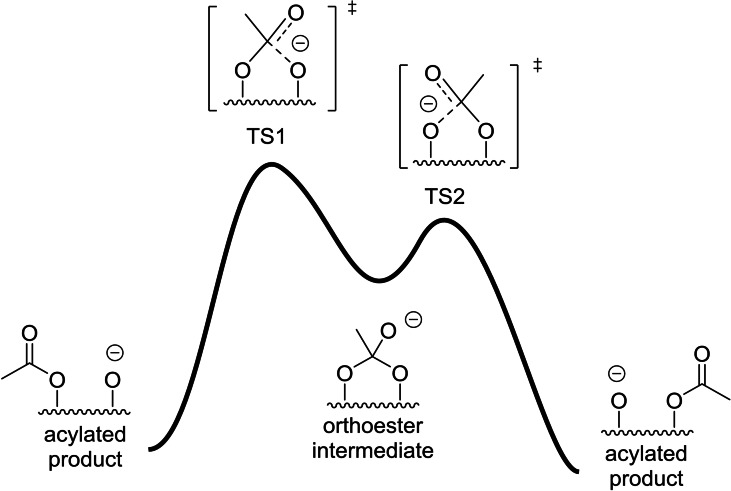
Mechanism of acyl migration assuming preceding deprotonation and the first step as the rate‐limiting transition stage.

The mechanism of migration has also been studied computationally. In general, it is well accepted that the process is stepwise, taking place via an orthoester intermediate, assuming preceding deprotonation and the first step as the rate‐determining stage (Figure 1). Nicholson and co‐workers^[19]^ have reported computational studies on intramolecular acyl group migration at a semiempirical level, supporting the existence of the intermediates, although no transition structures were calculated. Gritsan and co‐workers^[20]^ studied the process in acetoxyanthraquinones, in which there were no hydroxyl groups. Stachulski and co‐workers^[21]^ considered a complete deprotonation and calculated only the first transition structures of the migration processes leading to the orthoester intermediate. It should be noted that since no IRC studies were reported, the connection between the acylated compounds and the orthoester cannot be assessed properly.

More recently, the same authors^[22]^ have reported similar results for the intramolecular transacylation of acyl glucosides. In this study, the only consideration of the first transition state assumes intrinsically that this is the rate‐determining stage. Indeed, NMR experiments showed a first‐order kinetics, in good agreement with a stepwise reaction, in which the first step is rate limiting. By studying the acyl group migration between vicinal OH groups in 2’(3’)‐formylnucleosides, Petkov and co‐workers^[18]^ corroborated the stepwise mechanism via an orthoester intermediate and suggested that the migration is catalyzed by preliminary deprotonation which leads to spontaneous migration. Despite the fact that deprotonation is needed for the acyl group migration, consideration of a full anionic mechanism should be valid only when a complete deprotonation takes place, which is not the case when the reaction is carried out in water. Under these conditions, it should be necessary to consider the available concentration of the anion, which is a function of the pK_a_ of the corresponding hydroxyl group and the pH of the medium.

The nature of the acyl groups also significantly affects the migration. Several migration studies involving different acyl groups have been reported in glucuronic acid, due to the significant role of acyl glucuronides in the metabolism of pharmaceuticals.^[23]^ Two main structural factors affect the rate of migration: steric hindrance and the electronic properties of the acyl group in question. Larger acyl groups tend to migrate slower, and the stereochemistry at the α‐carbon also influences the migration process.[^17^, ^21^, ^24^, ^25^, ^26^] Increasing the electron withdrawing properties of the substituent on the acyl group also generally increases the rate of migration.

Majority of the earlier studies have not been performed under similar conditions, making comparisons between the different reports difficult. Despite of the earlier computational studies, a definitive approach for studying the molecular mechanism, considering all the stationery points (minima and transition structures), the reaction conditions and the different factors influencing the reaction and the reversibility of the process, has been lacking. Here, we describe a comprehensive study on the acyl group migration in different monosaccharides, by means of experimental and computational techniques, setting the stage for better understanding on how the acyl migration process depends on the chemical structures and the electronic properties, and the nature of the acyl groups.

## Results and Discussion

### Migration studies

**Figure 2:**
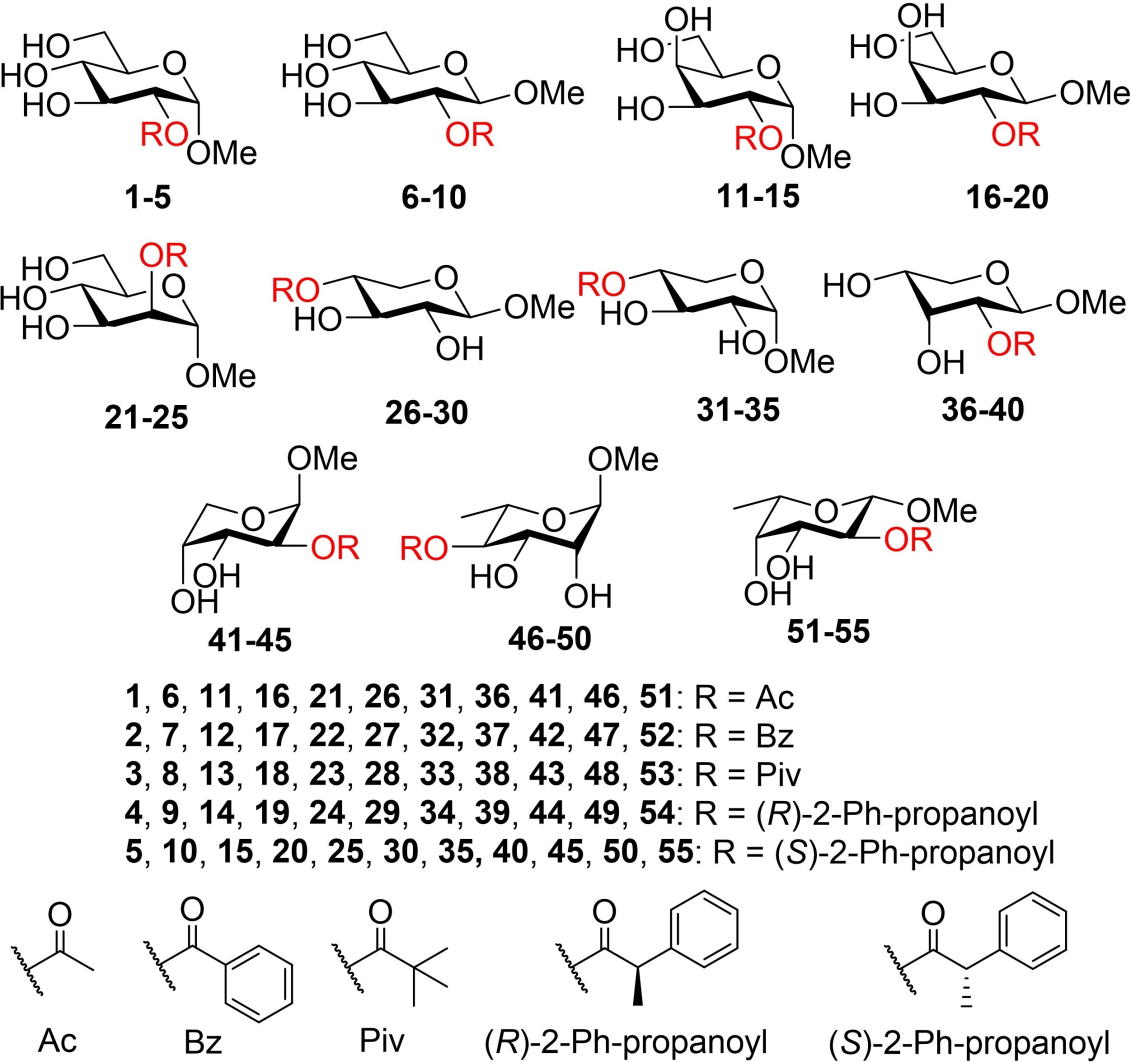
Structures of the investigated compounds.

Synthesis of the investigated compounds (Figure 2) is described in detail in the Supporting Information. Initial position of the acyl group was selected based on the simplest method for protection of the carbohydrate starting material. In most cases, the acyl group in the starting compound used for the migration studies is in the O2 position, but O4 was used when a primary hydroxyl position was not available and a simpler preparative route was available (xylose and rhamnose).

**Scheme 2:**
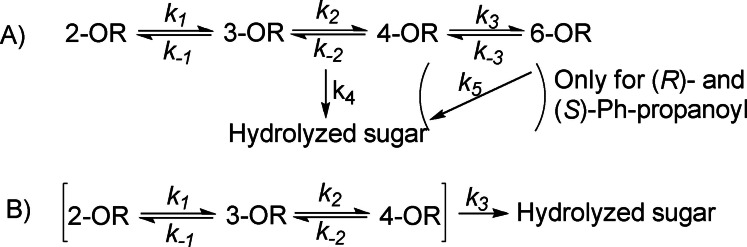
The reaction schemes used for calculations of the rate constants.

An important difference in the reaction schemes employed in this study, compared to most of the previous reports, is that the hydrolysis also takes place from the secondary position in the presence of a primary free hydroxyl. The hydrolysis rates from each position were considered equal for the acetyl, benzoyl and pivaloyl groups as, on average, the hydrolysis is over ten times slower than the migration (Scheme 2). For the (*R*)‐ and (*S*)‐2‐Ph‐propanoyls, however, hydrolysis from the primary positions was differentiated to obtain better fit of the data. As shown in Table 1, hydrolysis from the primary position is slower than from the secondary position. The rate difference significantly depends on the carbohydrate in question and, for example, for the (*R*)‐ and (*S*)‐2‐Ph‐propanoyls almost no hydrolysis is observed in Me α‐d‐mannopyranoside. The reason could be due to flexibility of the primary hydroxyl group in the carbohydrate, allowing the acyl group to protect the carbonyl carbon from hydrolysis, especially in the case of larger acyl groups.

**Table 1:** The rate constants for migration of the acetyl, benzoyl, pivaloyl, (*R*)‐ and (*S*)‐2‐Ph‐propanoyl groups in Me α‐ and β‐d‐glucopyranosides, Me α‐ and β‐d‐galactopyranosides and Me α‐d‐mannopyranoside according to path A displayed in Scheme 2.^[a]^

Acyl group		Ac	Bz	Piv	(*R*)‐2‐Ph‐propanoyl	(*S*)‐2‐Ph‐propanoyl
Compound		**1**	**2**	**3**	**4**	**5**
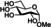 Me α‐d‐Glc	*k_1_* (h^−1^)	2.26E‐01±7.14E‐03	1.43E‐01±2.60E‐03	1.09E‐02±3.54E‐04	5.64E‐02±8.22E‐04	3.79E‐02±7.10E‐04
	*k*_*‐1*_ (h^−1^)	1.84E‐01±1.42E‐02	7.64E‐02±3.98E‐03	1.07E‐02±6.98E‐04	6.44E‐02±2.26E‐03	2.66E‐02±2.26E‐03
	*k_2_* (h^−1^)	1.82E‐01±1.23E‐02	9.24E‐02±6.10E‐03	9.52E‐03±1.02E‐03	3.22E‐02±6.38E‐03	8.62E‐02±9.92E‐03
	*k*_*‐2*_ (h^−1^)	3.64E‐01±1.09E‐01	1.41E‐01±4.52E‐02	1.04E‐02±2.48E‐03	1.38E‐01±9.26E‐02	1.46E‐01±3.62E‐02
	*k_3_* (h^−1^)	4.34E+00±2.20E+00	7.63E‐01±9.42E‐02	9.72E‐03±7.64E‐04	3.75E‐01±7.18E‐02	2.02E‐01±1.89E‐02
	*k*_*‐3*_ (h^−1^)	2.82E‐01±1.79E‐01	4.63E‐02±1.32E‐02	2.71E‐04±3.76E‐04	6.86E‐03±4.00E‐03	1.27E‐02±2.40E‐03
	*k_4_* (h^−1^)	3.17E‐03±2.70E‐04	8.40E‐04±1.56E‐04	2.72E‐04±1.63E‐05	5.01E‐03±1.35E‐04	5.05E‐03±1.73E‐04
	*k_5_* (h^−1^)	–	–	–	2.92E‐04±2.36E‐04	5.97E‐04±2.20E‐04
Compound		**6**	**7**	**8**	**9**	**10**
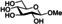 Me β‐d‐Glc	*k_1_* (h^−1^)	7.28E‐01±1.77E‐02	4.59E‐01±1.24E‐02	3.28E‐02±1.13E‐03	1.19E‐01±1.95E‐03	7.95E‐02±2.56E‐03
	*k*_*‐1*_ (h^−1^)	4.00E‐01±2.26E‐02	2.48E‐01±1.54E‐02	1.92E‐02±1.20E‐03	1.27E‐01±4.24E‐03	5.84E‐02±7.98E‐03
	*k_2_* (h^−1^)	4.14E‐01±1.69E‐02	2.06E‐01±1.06E‐02	1.61E‐02±1.25E‐03	5.73E‐02±2.86E‐03	1.20E‐01±1.20E‐02
	*k*_*‐2*_ (h^−1^)	4.62E‐01±1.16E‐01	2.12E‐01±4.70E‐02	1.89E‐02±3.10E‐03	1.13E‐01±2.18E‐02	1.19E‐01±4.30E‐02
	*k_3_* (h^−1^)	3.95E+00±4.46E‐01	1.09E+00±1.13E‐01	1.97E‐02±8.78E‐04	5.17E‐01±5.76E‐02	4.21E‐01±6.24E‐02
	*k*_*‐3*_ (h^−1^)	1.64E‐01±3.50E‐02	9.65E‐02±1.86E‐02	1.42E‐03±2.50E‐04	2.92E‐02±5.76E‐03	2.75E‐02±7.74E‐03
	*k_4_* (h^−1^)	2.66E‐03±3.62E‐04	9.76E‐04±2.42E‐04	3.09E‐04±1.51E‐05	4.09E‐03±1.76E‐04	4.42E‐03±4.12E‐04
	*k_5_* (h^−1^)	–	–	–	5.65E‐04±1.88E‐04	1.36E‐03±2.66E‐04
Compound		**11**	**12**	**13**	**14**	**15**
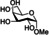 Me α‐d‐Gal	*k_1_* (h^−1^)	1.19E‐01±2.34E‐03	9.20E‐02±1.59E‐03	5.49E‐03±8.62E‐05	2.99E‐02±5.48E‐04	3.41E‐02±9.80E‐04
	*k*_*‐1*_ (h^−1^)	9.55E‐02±8.98E‐03	9.12E‐02±7.36E‐03	4.29E‐03±2.56E‐04	3.26E‐02±4.10E‐03	4.15E‐02±4.62E‐03
	*k_2_* (h^−1^)	8.74E‐01±9.98E‐02	1.17E+00±1.56E‐01	8.24E‐02±1.41E‐02	1.97E+00±6.28E‐01	1.65E+00±9.72E‐01
	*k*_*‐2*_ (h^−1^)	8.73E‐01±1.44E‐01	8.13E‐01±1.38E‐01	5.14E‐02±9.60E‐03	9.81E‐01±3.58E‐01	1.33E+00±8.44E‐01
	*k_3_* (h^−1^)	1.02E+00±1.02E‐01	2.02E‐01±6.98E‐03	1.29E‐03±1.46E‐04	8.43E‐02±4.22E‐03	4.78E‐02±2.64E‐03
	*k*_*‐3*_ (h^−1^)	1.97E‐01±2.62E‐02	4.78E‐02±3.52E‐03	4.47E‐05±6.14E‐04	2.01E‐02±1.58E‐03	4.90E‐03±9.90E‐04
	*k_4_* (h^−1^)	7.40E‐03±4.52E‐04	1.52E‐03±3.18E‐04	2.43E‐04±1.04E‐05	5.05E‐03±1.26E‐04	3.90E‐03±2.30E‐04
	k_5_ (h^−1^)	–	–	–	1.09E‐08±1.16E‐04	6.51E‐04±3.86E‐04
Compound		**16**	**17**	**18**	**19**	**20**
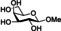 Me β‐d‐Gal	*k_1_* (h^−1^)	5.28E‐01±1.38E‐02	3.00E‐01±3.78E‐03	2.09E‐02±1.07E‐03	7.76E‐02±9.96E‐04	3.01E‐01±3.48E‐03
	*k*_*‐1*_ (h^−1^)	2.19E‐01±1.64E‐02	1.55E‐01±8.04E‐03	1.17E‐02±1.62E‐03	7.33E‐02±5.56E‐03	1.53E‐01±7.32E‐03
	*k_2_* (h^−1^)	8.73E‐01±3.30E‐02	1.15E+00±4.60E‐02	8.35E‐02±1.78E‐02	9.77E‐01±6.92E‐02	1.16E+00±4.22E‐02
	*k*_*‐2*_ (h^−1^)	6.20E‐01±4.06E‐02	5.40E‐01±3.02E‐02	3.87E‐02±9.32E‐03	4.05E‐01±4.04E‐02	5.41E‐01±2.76E‐02
	*k_3_* (h^−1^)	7.55E‐01±2.18E‐02	1.38E‐01±2.24E‐03	1.02E‐03±2.38E‐04	8.57E‐02±1.75E‐03	1.37E‐01±2.16E‐03
	*k*_*‐3*_ (h^−1^)	2.06E‐01±9.36E‐03	4.31E‐02±1.76E‐03	1.33E‐04±1.15E‐03	1.99E‐02±9.80E‐04	4.28E‐02±1.69E‐03
	*k_4_* (h^−1^)	1.36E‐02±1.33E‐03	4.27E‐03±5.56E‐04	9.40E‐04±1.32E‐04	4.36E‐03±1.82E‐04	1.58E‐03±2.28E‐04
	*k_5_* (h^−1^)	–	–	–	8.65E‐04±1.58E‐04	2.28E‐13±1.90E‐04
Compound		**21**	**22**	**23**	**24**	**25**
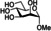 Me α‐d‐Man	*k_1_* (h^−1^)	1.91E+00±6.60E‐02	2.19E+00±1.77E‐01	2.04E‐01±1.07E‐02	2.08E+00±6.88E‐02	1.53E+00±7.52E‐02
	*k*_*‐1*_ (h^−1^)	1.39E+00±5.60E‐02	1.91E+00±1.67E‐01	1.62E‐01±9.66E‐03	1.41E+00±5.06E‐02	1.32E+00±7.46E‐02
	*k_2_* (h^−1^)	1.41E‐01±1.16E‐02	1.35E‐01±1.55E‐02	1.16E‐02±1.27E‐03	2.76E‐02±1.33E‐03	5.44E‐02±3.98E‐03
	*k*_*‐2*_ (h^−1^)	5.63E‐01±1.50E‐01	4.63E‐01±1.15E‐01	2.23E‐02±3.86E‐03	8.32E‐02±1.25E‐02	1.08E‐01±1.97E‐02
	*k_3_* (h^−1^)	1.69E+00±1.66E‐01	5.95E‐01±4.52E‐02	1.19E‐02±1.02E‐03	1.77E‐01±9.78E‐03	1.51E‐01±1.31E‐02
	*k*_*‐3*_ (h^−1^)	7.48E‐02±1.88E‐02	3.82E‐02±6.88E‐03	2.49E‐03±5.48E‐04	1.34E‐02±1.95E‐03	1.58E‐02±2.36E‐03
	*k_4_* (h^−1^)	3.02E‐03±2.20E‐04	6.08E‐04±1.31E‐04	2.90E‐04±2.02E‐05	3.97E‐03±7.84E‐05	4.97E‐03±1.61E‐04
	*k_5_* (h^−1^)	–	–	–	4.61E‐12±7.14E‐08	5.33E‐15±7.98E‐06

[a] Conditions: 100 mM phosphate buffer with 10 % D_2_O, pH 8, 25 °C

**Figure 3:**
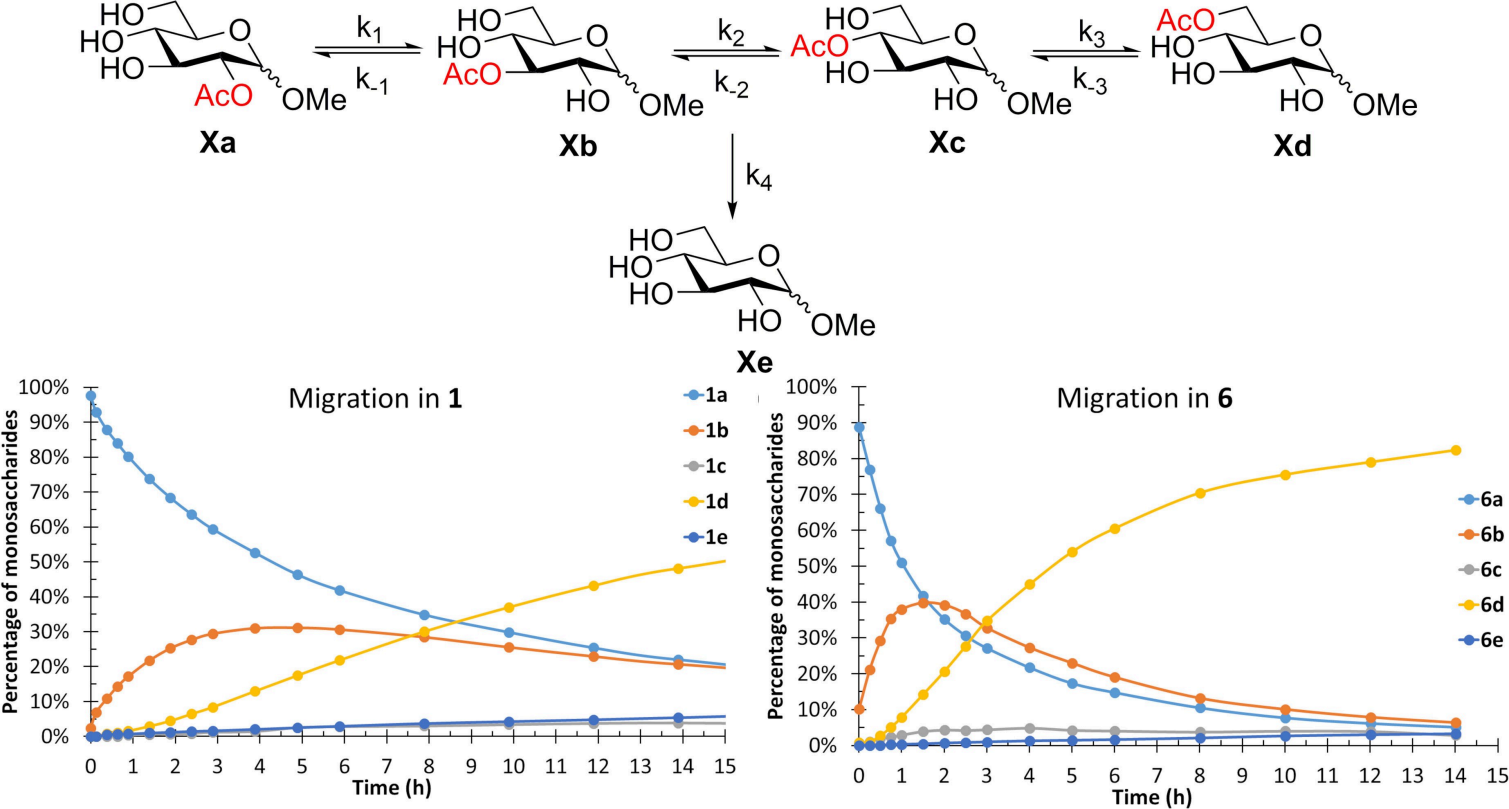
Comparison of the acetyl group migration in Me α‐ and β‐D‐glucopyranoside. Conditions: 100 mM phosphate buffer with 10 % D_2_O, pH 8, 25 °C.

An unexpected observation is that the rate of migration in carbohydrates having the OMe axially positioned at C1 in the preferred chair conformation is much slower than in the corresponding equatorial analogue. This is clearly demonstrated by comparing the Me α‐ and β‐glycopyranosides for glucosides (**1**–**10**), galactosides (**11**–**20**) and xylosides (**26**–**35**) (Tables 1 and 2). The difference is pronounced when the hydroxyl groups are located in a *trans* relationship. The rates of migration over hydroxyl groups in a *trans* relationship are 22–58 % slower for a carbohydrate with an axial anomeric position, compared to a carbohydrate with an equatorial anomeric position (Figure 3). Migration over a *cis* relationship is not affected as much and is actually slightly faster for carbohydrates with an axial anomeric position. The anomeric effect could be involved here, since when the O1 is axial, the bond between the C1 and the ring oxygen becomes shorter,^[27]^ resulting in strain in the formation of the migration transition state, especially for the *trans* hydroxyl groups. The *cis* hydroxyl groups, in turn, offer favorable conditions for the migration intermediate with less strain on the carbohydrate ring. The O4→O6 migration forms a 6‐membered ring with less strain than the corresponding 5‐membered ring. The differences between the rates of migration over *cis* relationships, for example, *k_2_*, *k*_*‐2*_ in **11** and **16**, are not significant and could be due to other factors, such as electronic effects induced by the rings.

**Table 2:** The rate constants for migration of the acetyl, benzoyl, pivaloyl, (R)‐ and (S)‐2‐Ph‐propanoyl groups in Me α‐ and β‐D‐xylopyranosides, Me β‐D‐ribopyranoside, Me β‐D‐arabinopyranoside, Me α‐L‐rhamnopyranoside, and Me β‐L‐fucopyranoside according to path B displayed in Scheme 2.^[a]^

Acyl group		Ac	Bz	Piv	(*R*)‐2‐Ph‐propanoyl	(*S*)‐2‐Ph‐propanoyl
Compound		**26**	**27**	**28**	**29**	**30**
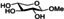 Me β‐d‐Xyl	*k_1_* (h^−1^)	5.94E‐01±3.16E‐01	4.05E‐01±6.88E‐02	2.58E‐02±1.04E‐03	1.22E‐01±3.74E‐02	1.72E‐01±3.08E‐02
	*k*_*‐1*_ (h^−1^)	2.64E‐01±1.22E‐01	2.16E‐01±2.96E‐02	1.83E‐02±6.40E‐04	1.31E‐01±3.46E‐02	1.28E‐01±1.80E‐02
	*k_2_* (h^−1^)	3.28E‐01±3.10E‐02	1.62E‐01±8.62E‐03	8.73E‐03±1.03E‐04	4.95E‐02 ±5.00E‐03	1.02E‐01±6.68E‐03
	*k*_*‐2*_ (h^−1^)	2.81E‐01±1.71E‐02	1.51E‐01±4.00E‐03	8.13E‐03±4.94E‐05	4.20E‐02±2.02E‐03	8.24E‐02±2.68E‐03
	*k_3_* (h^−1^)	5.87E‐03±1.06E‐03	1.35E‐03±3.96E‐04	2.20E‐04±4.48E‐06	2.91E‐03±1.42E‐04	3.57E‐03±1.97E‐04
Compound		**31**	**32**	**33**	**34**	**35**
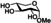 Me α‐d‐Xyl	*k_1_* (h^−1^)	2.11E‐01±2.50E‐02	1.76E‐01±1.27E‐02	8.67E‐03±1.58E‐03	4.68E‐02±1.06E‐02	2.92E‐02±4.36E‐03
	*k*_*‐1*_ (h^−1^)	1.53E‐01±1.44E‐02	1.06E‐01±6.30E‐03	6.68E‐03±7.96E‐04	4.99E‐02±8.98E‐03	4.74E‐02±9.22E‐03
	*k_2_* (h^−1^)	7.30E‐02±2.94E‐03	5.32E‐02±1.26E‐03	5.53E‐03±3.30E‐04	2.20E‐02±2.20E‐03	3.76E‐02±4.36E‐03
	*k*_*‐2*_ (h^−1^)	8.41E‐02±1.35E‐03	6.06E‐02±6.10E‐04	5.15E‐03±1.29E‐04	1.93E‐02±7.60E‐04	4.22E‐02±1.82E‐03
	*k_3_* (h^−1^)	2.17E‐03±1.45E‐04	4.68E‐04±6.48E‐05	1.64E‐04±1.63E‐05	2.26E‐03±1.01E‐04	2.66E‐03±8.74E‐05
Compound		**36**	**37**	**38**	**39**	**40**
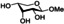 Me β‐d‐Rib	k_1_ (h^−1^)	3.04E+01±8.16E‐01	1.21E+01±5.92E‐02	3.62E‐01±3.30E‐03	7.50E+00±9.92E‐02	1.01E+01±6.18E‐02
	k_‐1_ (h^−1^)	1.13E+01±4.00E‐01	3.35E+00±3.60E‐02	1.05E‐01±2.82E‐03	2.94E+00±5.60E‐02	3.76E+00±3.20E‐02
	k_2_ (h^−1^)	1.85E+01±7.70E‐01	4.92E+00±6.98E‐02	1.92E‐01±6.22E‐03	4.68E+00±1.11E‐01	4.68E+00±3.92E‐02
	k_‐2_ (h^−1^)	2.20E+01±9.96E‐01	8.50E+00±1.41E‐01	3.27E‐01±1.41E‐02	7.45E+00±2.02E‐01	6.55E+00±6.40E‐02
	k_3_ (h^−1^)	^[b]^	^[b]^	^[b]^	^[b]^	^[b]^
Compound		**41**	**42**	**43**	**44**	**45**
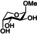 Me β‐d‐Ara	*k_1_* (h^−1^)	1.02E‐01±1.03E‐03	7.22E‐02±9.94E‐04	4.16E‐03±8.32E‐03	2.74E‐02±1.23E‐03	2.52E‐02±1.11E‐03
	*k*_*‐1*_ (h^−1^)	5.40E‐02±3.06E‐03	5.25E‐02±3.24E‐03	3.86E‐03±7.72E‐03	2.44E‐02±3.48E‐03	3.80E‐02±4.46E‐03
	*k_2_* (h^−1^)	1.72E+00±1.96E‐01	1.53E+00±2.50E‐01	1.92E‐01±3.84E‐01	2.00E+00±2.76E+00	2.00E+00±2.22E+00
	*k*_*‐2*_ (h^−1^)	1.31E+00±1.60E‐01	8.94E‐01±1.58E‐01	9.65E‐02±1.93E‐01	1.29E+00±1.81E+00	1.06E+00±1.21E+00
	*k_3_* (h^−1^)	2.27E‐03±2.27E‐03	5.54E‐04±1.67E‐04	1.61E‐04±3.22E‐04	2.23E‐03±1.54E‐04	2.09E‐03±1.30E‐04
Compound		**46**	**47**	**48**	**49**	**50**
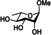 Me α‐l‐Rha	*k_1_* (h^−1^)	1.46E+00±6.42E‐02	1.63E+00±1.93E‐01	1.36E‐01±1.08E‐01	6.98E‐01±1.73E‐01	2.74E+00±1.62E+00
	*k*_*‐1*_ (h^−1^)	1.05E+00±4.30E‐02	1.38E+00±1.56E‐01	1.35E‐01±1.02E‐01	7.54E‐01±1.73E‐01	2.21E+00±1.29E+00
	*k_2_* (h^−1^)	1.27E‐01±1.93E‐03	9.18E‐02±2.86E‐03	9.60E‐03±1.25E‐03	4.82E‐02±3.14E‐03	2.94E‐02±1.14E‐03
	*k*_*‐2*_ (h^−1^)	2.89E‐01±1.54E‐03	1.49E‐01±1.47E‐03	1.32E‐02±6.54E‐04	5.32E‐02±1.41E‐03	4.68E‐02±5.70E‐04
	*k_3_* (h^−1^)	3.73E‐03±1.37E‐04	1.58E‐03±1.86E‐04	2.47E‐04±6.74E‐05	4.75E‐03±1.55E‐04	3.47E‐03±7.56E‐05
Compound		**51**	**52**	**53**	**54**	**55**
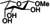 Me β‐l‐Fuc	*k_1_* (h^−1^)	4.25E‐01±1.13E‐03	2.17E‐01±9.16E‐04	1.18E‐02±4.54E‐05	6.22E‐02±9.10E‐04	5.75E‐02±9.20E‐04
	*k*_*‐1*_ (h^−1^)	1.41E‐01±1.33E‐03	9.97E‐02±1.88E‐03	5.98E‐03±9.82E‐05	4.87E‐02±2.72E‐03	6.35E‐02±3.84E‐03
	*k_2_* (h^−1^)	4.39E‐01±2.00E‐03	5.33E‐01±4.72E‐03	2.83E‐02±2.10E‐04	8.16E‐01±8.86E‐02	3.95E‐01±2.26E‐02
	*k*_*‐2*_ (h^−1^)	2.25E‐01±1.59E‐03	1.86E‐01±2.56E‐03	8.19E‐03±9.88E‐05	3.42E‐01±4.16E‐02	1.33E‐01±1.05E‐02
	*k_3_* (h^−1^)	2.50E‐03±6.12E‐05	1.02E‐03±7.42E‐05	7.32E‐05±3.26E‐06	1.34E‐03±8.52E‐05	1.57E‐03±1.02E‐04

[a] Conditions: 100 mM phosphate buffer with 10 % D_2_O, pH 8, 25 °. [b] not determined.

The migration rates between hydroxyl groups with the same relationship and position, for example, O2⇌O3 in α‐glucoside and α‐galactoside, and O3⇌O4 in α‐glucoside and α‐mannoside, have similar values for each acyl group. The strain in the ring, when the transition state is formed, is mostly influenced by the stereochemical and structural relationship of the hydroxyl groups and the strain in the carbohydrate ring by default. The small differences observed are most likely due to the orientations of the other hydroxyl groups, inducing strain and electronic effects on the transition state. Similar reasons, in combination with the electronic properties and steric hindrance of the hydroxyl groups involved, could be responsible for the differences observed between the O2⇌O3 and O3⇌O4 migrations, when these are in an equatorial orientation.

**Figure 4:**
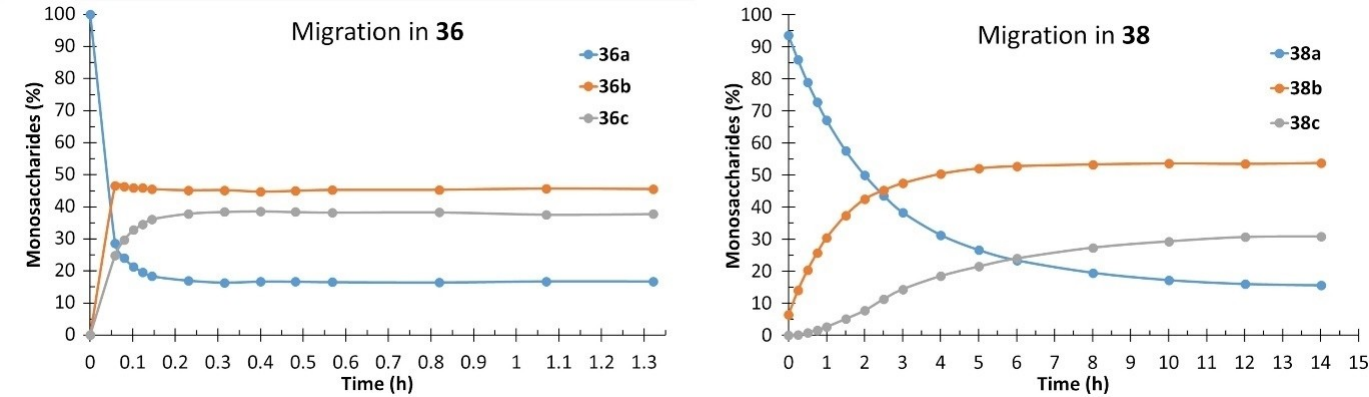
Migration of the acetyl (**36**) and pivaloyl group (**38**) in Me β‐d‐ribopyranoside. Conditions: 100 mM phosphate buffer with 10 % D_2_O, pH 8, 25 °C.

The migration in Me β‐d‐ribopyranoside was surprisingly fast. An equilibrium was reached already after 15–30 min for most of the acyl groups and in 14 h for the pivaloyl group (Figure 4). The difference is significant, since for most of the monosaccharides at least 1 week is needed before reaching the equilibrium in pivaloyl group migration. The rates of migration in Me β‐d‐ribopyranoside are 10–100 times greater than in Me β‐d‐xylopyranoside. Comparing the migration in ribose to other carbohydrates containing hydroxyl groups in *cis* relationships, i. e., arabinopyranoside, fucopyranoside and rhamnopyranoside, it can be observed that the acetyl group migration in ribose is ca. 10 times greater than the migration between other *cis* hydroxyl groups in the other pyranosides. The rate of migration for the other acyl groups in ribose varies significantly, all from 1–50 times the rate between *cis* hydroxyl groups in other pyranosides. In an earlier study by Widmalm and co‐workers,^[28]^ it was shown that the preferred conformations of Nap‐β‐d‐ribopyranoside involve several different skew conformations, in combination with two chair conformations, while for the corresponding xylopyranoside the ^4^C_1_ conformation dominates. These differences in the preferred solution conformations of the monosaccharides clearly have a significant effect on the rate of acyl group migration. The larger acyl groups could shift the preferred conformation towards a less favorable one for migration and, in some cases, to a conformation that results in similar migration rate as in arabinose, fucose and rhamnose.

**Figure 5:**
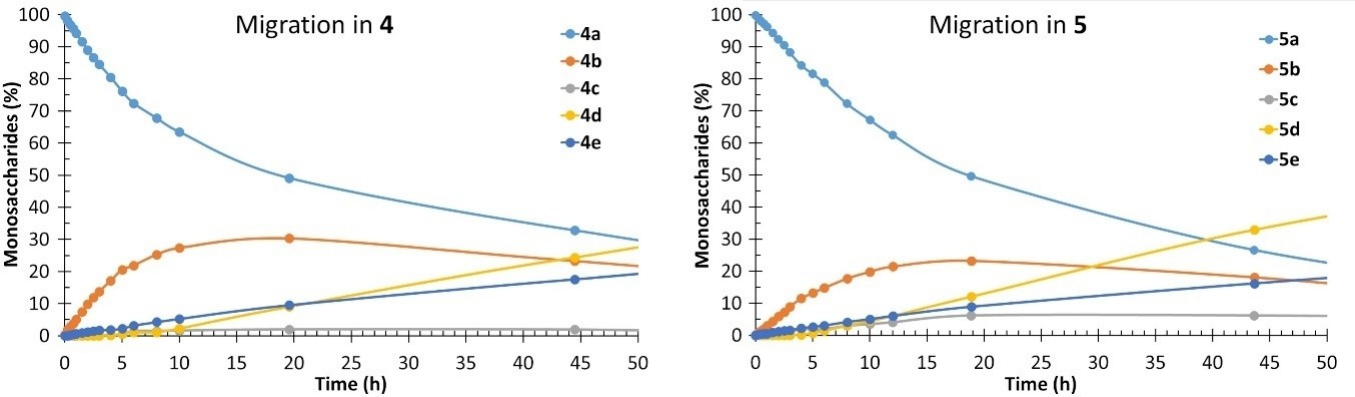
Comparison of the *R* and *S*‐2‐Ph‐Propanoyl migration in Me α‐d‐glucopyranoside. Conditions: 100 mM phosphate buffer with 10 % D_2_O, pH 8, 25 °C.

As expected, a clear difference between the (*R*)‐ and (*S*)‐Ph‐propanoyls was observed in all monosaccharides. It appears that in all d‐sugars investigated with O2, O3 and O4 equatorially positioned, *k*_*‐1*_ (O3→O2) is greater than *k_2_* (O3→O4) with the *R* isomer and *vice versa* with the *S* isomer. This has been investigated in glucuronic acid both experimentally and computationally,[^21^, ^22^, ^29^] with the difference being due to the configuration at the α‐carbon and the preferred transition state. As seen in Figure 5, the concentration of **5 a** decreases slower than **4 a**, because in the *S* isomer (**5 a**) the methyl group blocks the attack from O3 at the ground state. In many cases, the differences are not significant, on the average approximately a factor of two.

An interesting observation is that the rate of the benzoyl group migration is faster than the acetyl group migration when the hydroxyl groups have a *cis* relationship, while being slightly slower in a *trans* relationship. Another surprising observation is that the 2‐Ph‐propanoyls have similar migration rates compared to the acetyl group migration between hydroxyl groups in a *cis* relationship, but much slower compared to acetyl group migration between hydroxyl groups which are *trans*. It seems that the steric properties of the acyl groups affect the rate of migration less in migration over hydroxyl groups in a *cis* relationship, while in migration over hydroxyl groups in a *trans* relationship the influence of the steric properties of the acyl groups on the migration rate is more pronounced.

Several factors affect the rate of migration, such as pH and the temperature, but one factor that has not been investigated earlier is the influence of buffer strength. In the present study, this was elucidated by following the migration of the acetyl group in compound **21** in 50, 100 and 500 mM phosphate buffers under constant pH=8. It was observed that all rate constants are influenced to a similar degree at the same buffer strength and a coefficient could thus be introduced for comparing the rate constants in the 50 and 500 mM buffers to the rate constants in the 100 mM buffer. Migration rates in the 500 mM buffer are 1.49 times the rate at 100 mM and the rate in the 50 mM buffer is 0.87 times the rate at 100 mM. The differences may be due to stabilization of the anion at the higher concentrations, providing the anion more time for nucleophilic attack at the carbonyl carbon. This simple experiment clearly demonstrates the significant effect of the different factors and reaction conditions on the rate of acyl migration.

For verifying that the pH dependency is linear beyond the pH interval 7–8,^[17]^ pH=6, 7, 7.5 and 9 were also employed in studying the migration in compound **1**. The rate constants calculated at pH 8 were used as a starting point and coefficients were then determined to correlate the rate constants at pH 8 to data obtained at the other pH:s (Table 3). In theory, the coefficient could be calculated from the concentration of [OH^–^], providing the theoretical coefficient as [OH^−^]_pH=x_/[OH^−^]_pH=8_. The experimental coefficients determined match well with the theoretical coefficients. The differences between the theoretical and experimental coefficients are most likely due to measuring errors in the migration and the error in preparation of the buffers. The larger error at pH 9 is likely due to the faster migration, resulting in a lower number of scans in the NMR‐spectra, influencing the error margin. These results confirm that prior deprotonation is required in the migration process and that the ability of the solution to deprotonate the hydroxyl groups have a large impact on the rate of migration.

**Table 3:** The pH studied with the theoretical values and the experimental values in relationship to the migration of **1** at pH=8

pH	theoretical coefficient	experimental coefficient
9	10	7.1
8	1	1.0
7.5	0.32	0.35
7	0.1	0.085
6	0.01	0.0082

### Computational studies

**Scheme 3:**
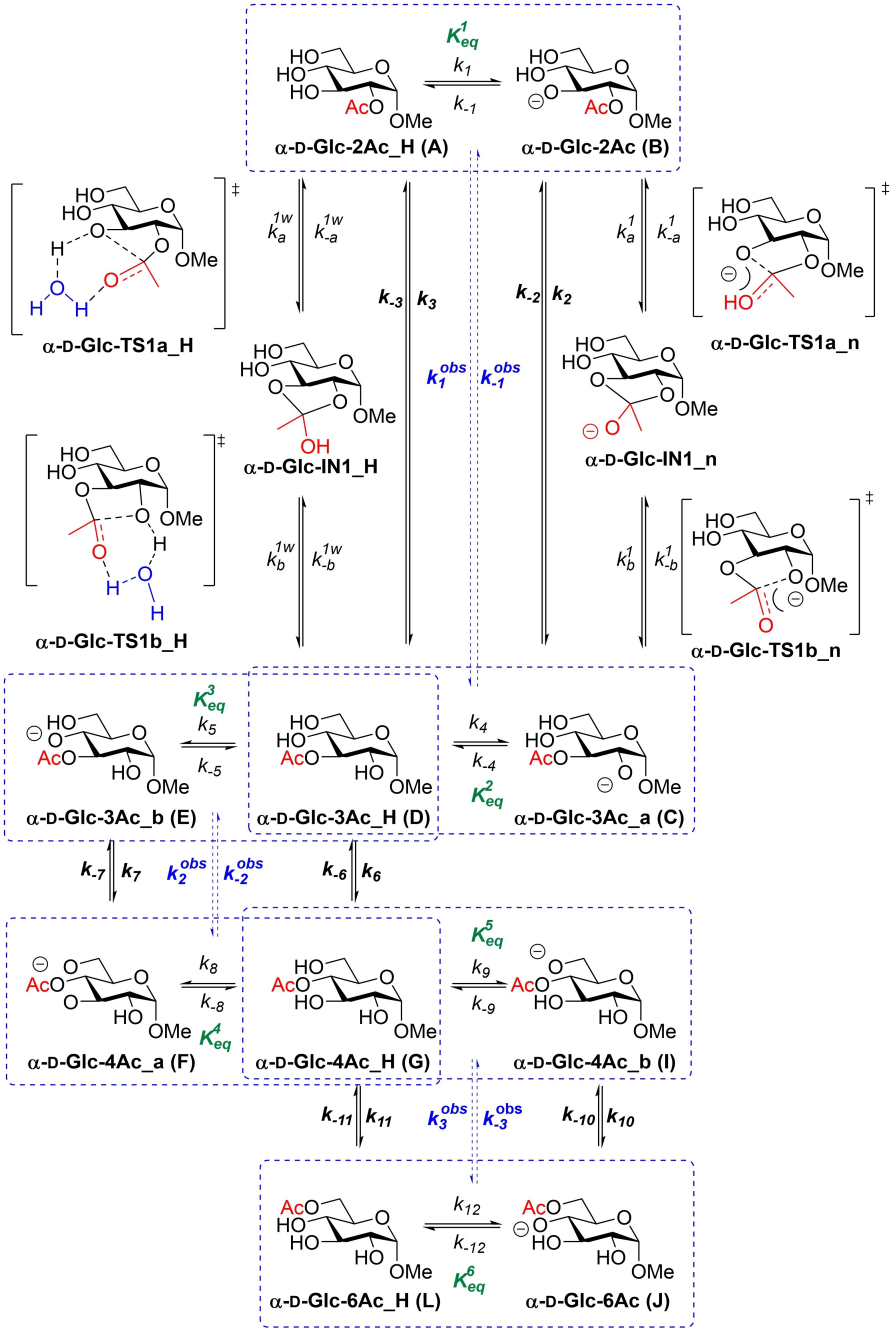
Acetyl group migration in Me α‐d‐glucopyranosides.

The acetyl group migration in Me Ac‐α‐d‐glucopyranosides, in particular the migration from **α‐d‐Glc‐2Ac_H** to **α‐d‐Glc‐3Ac_H**, was used as a starting point. Considering an orthoester intermediate, in agreement with experimental kinetic results, two different mechanisms are, in principle, possible (without considering the limiting factor of hydrolysis), i. e., a neutral mechanism, for which a water molecule would presumably be involved, starting from the protonated form **α‐d‐Glc‐2Ac_H** (Scheme 3, left) and an anionic mechanism starting from the deprotonated form **α‐d‐Glc‐2Ac** (Scheme 3, right).

Both mechanisms are competitive and the corresponding observed rates $k_{1}^{obs}$ and $k_{-1}^{obs}$ depend on the available concentration of the protonated and deprotonated species, which, in turn, are a function of the pH and the pK_a_ of the hydroxyl group involved. It should be noted that a more complex equilibrium with other deprotonated forms exists, but these species are not productive. Consequently, we only considered the deprotonation of the OH attacking the carbonyl group for the model. Previous computational studies have not considered this fact and only calculated full anionic mechanisms, which provided energy barriers considerably lower than those observed experimentally.

The kinetic dependence was obtained by solving the kinetic equations applying the transition state theory (see Supporting Information). The obtained expressions for the observed rates are given in Equations (1) and 2.

(1) $$k_{1}^{obs}={-k}_{3}+\frac{k_{2}\cdot K_{eq}^{1}}{{\left[H\right]}^{+}}$$

(2) $$k_{-1}^{obs}={-k}_{-3}+\frac{k_{-2}\cdot K_{eq}^{2}}{{\left[H\right]}^{+}}$$

These equations clearly showed the contribution of both mechanisms and, in the case of the anionic mechanism the linear dependence of both pH and pK_a_. The corresponding equilibrium constants can be obtained from the pK_a_’s of **α‐d‐Glc‐2Ac_H** and **α‐d‐Glc‐3Ac_H**, according to Equations (3) and 4.

(3) $$K_{eq}^{1}=\;\frac{k_{1}}{k_{-1}}=\;10^{{-pK}_{a}^{1}}$$

(4) $$K_{eq}^{2}=\;\frac{k_{4}}{k_{-4}}=10^{{-pK}_{a}^{2}}$$

The corresponding rate constants for neutral and anionic mechanisms can be obtained from the individual constants [Eqs. (5)‐8]:

(5) $$k_{3}=\;\frac{k_{a}^{1w}\cdot \;k_{b}^{1w}}{k_{-a}^{1w}+k_{b}^{1w}}$$

(6) $$k_{-3}=\;\frac{k_{-a}^{1w}\cdot \;k_{-b}^{1w}}{k_{-a}^{1w}+k_{b}^{1w}}$$

(7) $$k_{2}=\;\frac{k_{a}^{1}\cdot \;k_{b}^{1}}{k_{-a}^{1}+k_{b}^{1}}$$

(8) $$k_{-2}=\;\frac{k_{-a}^{1}\cdot \;k_{-b}^{1}}{k_{-a}^{1}+k_{b}^{1}}$$

In Equations (5)‐(8), $k_{a}^{1w}$ ,$k_{-a}^{1w}$ ,$\;k_{b}^{1w}$ ,$\;k_{-b}^{1w}$ , $k_{a}^{1}$ , $k_{-a}^{1}$ ,$\;k_{b}^{1}$ , and$\;k_{-b}^{1}$ can be obtained from the corresponding energy barriers by using the Eyring equation. The preliminary studies were further extended to the whole migration process in Me α‐d‐glucopyranoside (Scheme 3).

**Figure 6:**
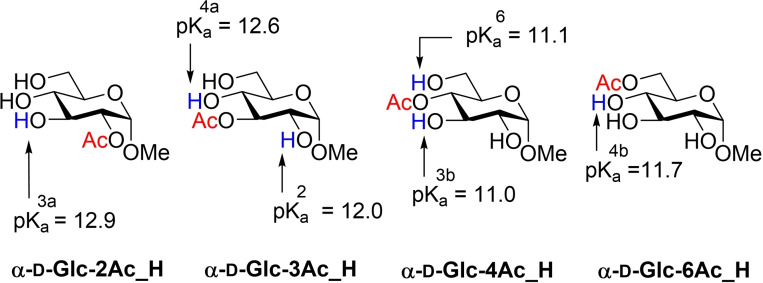
Calculated (m062x/cc‐pvtz/SMD=water) pK_a_ values for **α‐d‐Glc‐2Ac_H** and **α‐d‐Glc‐3Ac_H** and **α‐d‐Glc‐4Ac_H** and **α‐d‐Glc‐6Ac_H**.

There are several methods for calculating pK_a_s and in all cases the task is challenging, mainly because very small variations in energy result in differences between the pK_a_ values of one or two units.^[30]^ The methodology for calculating pK_a_s also depends on the type of the functional group; in particular, determination of pK_a_s of carbohydrates have been recently reported for various d‐glucose and d‐fructose tautomers using high level calculations.^[31]^ Unfortunately, when this methodology was applied to Me *O*‐Ac‐α‐d‐glucopyranosides, pK_a_ values higher than 20 were obtained, clearly out of the range (ca. 10–14) of what would be expected for a carbohydrate. Schlegel and co‐workers^[32]^ reported an efficient method for calculating pK_a_s of thiols in aqueous solution using DFT. The methodology consists of using three explicit molecules of water in addition to a continuum solvent model. After some experiments and benchmarking of levels of theory (see Supporting Information), the model was applied to **α‐d‐Glc‐2Ac_H** and **α‐d‐Glc‐3Ac_H** using m062x/6‐31+G(d,p)/SMD=water as level of theory for the optimization of the structures which has shown excellent results with thiols.^[33]^ After some benchmarking, m062x/cc‐pvtz/SMD=water level of theory was used for energy calculations. The obtained pK_a_ values for **α‐d‐Glc‐2Ac_H** and **α‐d‐Glc‐3Ac_H** were 12.9 and 12.0, respectively (Figure 6), clearly in the range of that expected for a carbohydrate. By applying Equations (3) and (4), the values for the equilibrium constants are: $K_{eq}^{1}$ =1.3 ⋅ 10^−13^ and $K_{eq}^{2}$ = 1 ⋅ 10^−12^. We extended the calculations to the rest of the hydroxyl groups involved in acetyl group migration; the corresponding pK_a_ values are, likewise, given in Figure 6.

The direct acyl migration under neutral conditions involves H‐transfer through a highly strained four membered cyclic transition state that would render the process unattainable in terms of energy barrier. This sort of H‐transfer processes are usually mediated by a molecule of water that facilitates the proton exchange between the functional groups involved in the reaction. The acetyl group migration from **α‐d‐Glc‐2Ac_H** to **α‐d‐Glc‐3Ac_H** mediated by a molecule of water, illustrated in Scheme 5, was calculated and barriers of 28.9 and 30.1 kcal/mol were obtained for the formation of the orthoester and the final release of the migrated acetyl group, respectively. By applying the Eyring equation and according to Equations (5) and (6) the following values were obtained for the rate constants: $k_{3}$ =4.76 ⋅ 10^−10^ s^−1^ and $k_{-3}$ =3.06 ⋅ 10^−11^ s^−1^, which are clearly lower than those observed experimentally. Mediation by two molecules of water gave slightly higher barriers due to the increasing entropy (see Supporting Information).

Calculations were extended to further migrations of Me *O*‐Ac‐α‐d‐glucopyranosides (Scheme 3) and similar energy barriers were obtained. Table 4 lists the formal energy barriers and the corresponding rate constants.

**Table 4:** Calculated formal energy barriers^[a]^ (kcal/mol) and rate constants^[b]^ (s^‐1^) for the acetyl group migration in Me *O*‐Ac‐α‐d‐glucopyranosides under a neutral mechanism with the participation of one molecule of water.

	rate constant	ΔG
k_3_	4.76x10^−10^	30.2
k_‐3_	3.06x10^−11^	31.8
k_6_	1.56x10^−14^	36.3
k_‐6_	1.28x10^−11^	32.3
k_11_	1.54x10^−10^	30.9
k_‐11_	1.07x10^−9^	29.7

[a] Obtained from the individual barriers of the stepwise mechanism calculated at m062x/cc‐pvtz/SMD=water//m062x/6‐31+G(d,p)/SMD=water level of theory. [b] Obtained by applying Equation (5), (6) and similar ones in further migrations (see Supporting Information).

An anionic model based on a naked anion, as suggested by several authors in the past, is far from the real situation when the reaction takes place in water as a solvent, even though a continuum solvent model is considered. In fact, calculation of such a model provides values of rate constants that are not consistent with the values observed experimentally (see Supporting Information). Since the same anions used for pK_a_ calculations should be considered for the anionic mechanism, we decided to keep the model coherent and calculate the mechanism using three explicit molecules of water in addition to the solvent model (Scheme 5).

**Figure 7:**
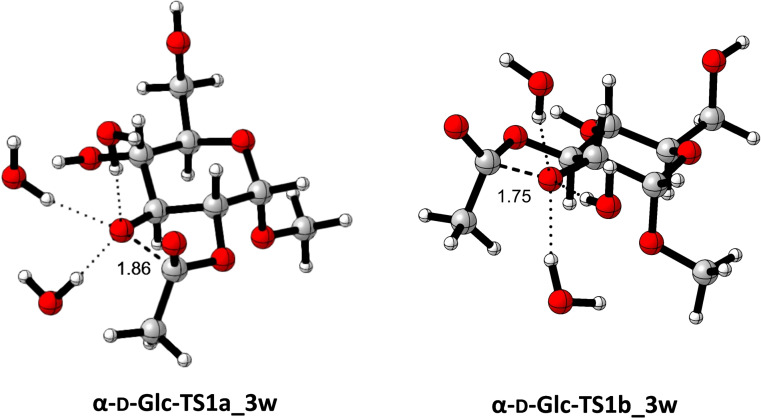
Optimized (m062x/6‐31+G(d,p)/SMD=water) geometries of transition structures **α‐d‐Glc‐TS1a_3 w** and **α‐d‐Glc‐TS1b_3 w**, corresponding to the first migration step from O2 to O3.

For these calculations, it was necessary to evaluate several conformations, in both minima and transition structures. Those models having the same intramolecular interactions were then selected, avoiding spurious interactions such as H‐bonds, unreliable in an aqueous medium, that might contaminate the model. In other words, isodesmic processes also considering non‐covalent interactions have been measured (for details see Supporting Information). Under these conditions, barriers of 14.8 and 12.2 kcal/mol for **α‐d‐Glc‐TS1a_3 w** and **α‐d‐Glc‐TS1b_3 w**, respectively was obtained. The optimized geometries of these transition structures are given in Figure 7.

By applying Equations (7) and (8), *k_2_* =88 s^−1^ and *k*_*‐2*_=6.82 s^−1^ were obtained. Further extension to the rest of the migrations in α‐d‐glucosides provided the values listed in Table 5.

**Table 5:** Calculated (m062x/cc‐pvtz/SMD=water//m062x/6‐31+G(d,p)/SMD=water) formal energy barriers^[a]^ (kcal/mol) and rate constants^[b]^ (s^−1^) for the acetyl group migration in Me *O*‐Ac‐α‐d‐glucopyranosides under an anionic mechanism considering three explicit molecules of water.

	rate constant	ΔG
k_2_	8.80x10^1^	14.8
k_‐2_	6.82x10^0^	16.3
k_7_	1.42x10^0^	17.3
k_‐7_	6.49x10^−2^	19.1
k_10_	2.19x10^2^	14.3
k_‐10_	4.01x10^−1^	18.0

[a] Obtained from the individual barriers of the stepwise mechanism. [b] Obtained by applying Equation (5) and (6) and similar ones in further migrations (see Supporting Information).

**Scheme 4:**
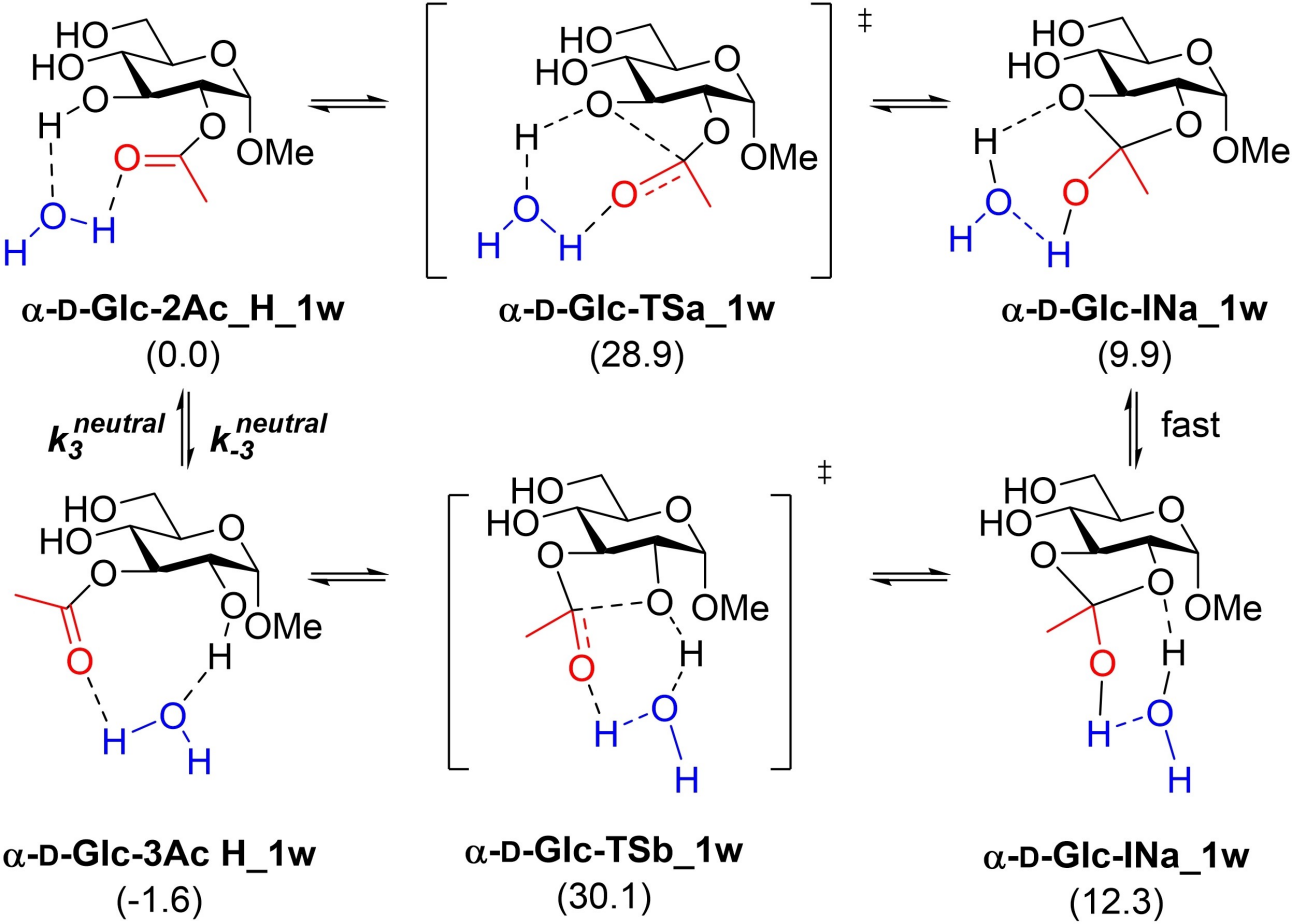
Acetyl group migration under neutral conditions mediated by one molecule of water. Relative energies (kcal/mol) calculated at m062x/cc‐pvtz/SMD=water//m062x/6‐31+G(d,p)/SMD=water level of theory are given in brackets.

Once the two mechanisms and the pK_a_’s have been calculated, it is possible to apply Equations (1) and (2) and those similar corresponding to the rest of the acetyl group migration in Me O‐Ac‐α‐d‐glucopyranosides (see Supporting Information) for calculating the predicted observed constants $k_{n}^{obs}$ . Table 6 collects the predicted values and the corresponding formal energy barriers for the whole migration process at pH=8, as depicted in Scheme 4; the experimental values have been included for the purpose of comparison.

**Table 6:** Calculated formal energy barriers^[a]^ (kcal/mol) and rate constants^[b]^ (s^−1^) for the acetyl group migration in Me *O*‐Ac‐α‐d‐glucopyranosides at pH=8.

	experimental		predicted		
	*rate constant*	*ΔG*	*rate constant*	*ΔG*	*ΔG error*
$k_{1}^{obs}$	6.28×10^−5^	23.2	3.35×10^−4^	22.2	1.0
$k_{-1}^{obs}$	5.11×10^−5^	23.3	4.03×10^−5^	23.5	0.2
$k_{2}^{obs}$	5.06×10^−5^	23.3	3.15×10^−6^	25.0	1.6
$k_{-2}^{obs}$	1.01×10^−4^	22.9	4.50×10^−6^	24.8	1.8
$k_{3}^{obs}$	1.21×10^−3^	21.4	6.32×10^−4^	21.8	0.4
$k_{-3}^{obs}$	7.83×10^−5^	23.1	8.15×10^−5^	23.1	0.0

[a] Obtained from the individual barriers of the stepwise mechanism calculated at m062x/cc‐pvtz/SMD=water//m062x/6‐31+G(d,p)/SMD=water level of theory and then from the rate constants by using the Eyring's equation. [b] Obtained by applying Equation (1) and (2) and similar ones in further migrations (see Supporting Information).

**Scheme 5:**
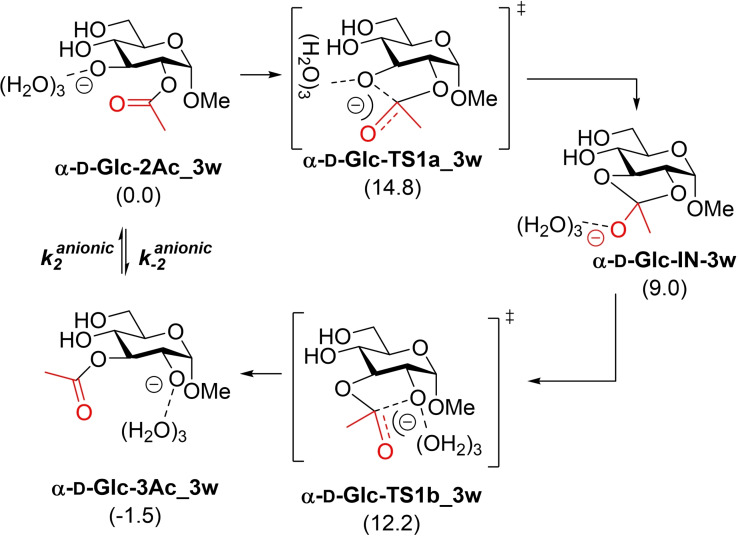
Acetyl group migration under basic conditions considering three explicit molecules of water. Relative energies (kcal/mol) calculated at m062x/cc‐pvtz/SMD=water//m062x/6‐31+G(d,p)/SMD=water level of theory are given in brackets.

Due to the logarithmic dependence of the rate constants on the activation energy, small variations of the latter results in great variations of the former. The experimental error in DFT should be placed in 1–2 kcal/mol,^[34]^ and values within this range can be considered in good agreement with the experimental results. In this regard, all the values predicted for the acetyl group migration in Me *O*‐Ac‐α‐d‐glucopyranosides have errors lower than 2 kcal/mol with respect to those observed experimentally, rendering the model, considering both mechanisms and using three explicit molecules of water in the anionic one, as valid. Moreover, the model also considers the experimentally observed pH dependence and reproduces the observed linearity. Notably, the observed difference of ca. ten orders of magnitude between the rate constants of the neutral mechanism and those of the anionic mechanism, results, at pH=8, in differences of five orders of magnitude between the two terms in Equations (1) and (2). This makes it possible to disregard the term corresponding to the neutral mechanism, providing a simplified general equation [Eq. (9)] for the observed rate constants:

(9) $$K_{i}^{obs}=\frac{k_{j}^{anionic}\cdot K_{eq}^{m}}{{\left[H\right]}^{+}}$$

where i=1, −1, 2, −2, 3, −3; j=2, −2, 7, −7, 10, −10; m=1, 2, 3, 4, 5, 6 (Scheme 3)

**Figure 8:**
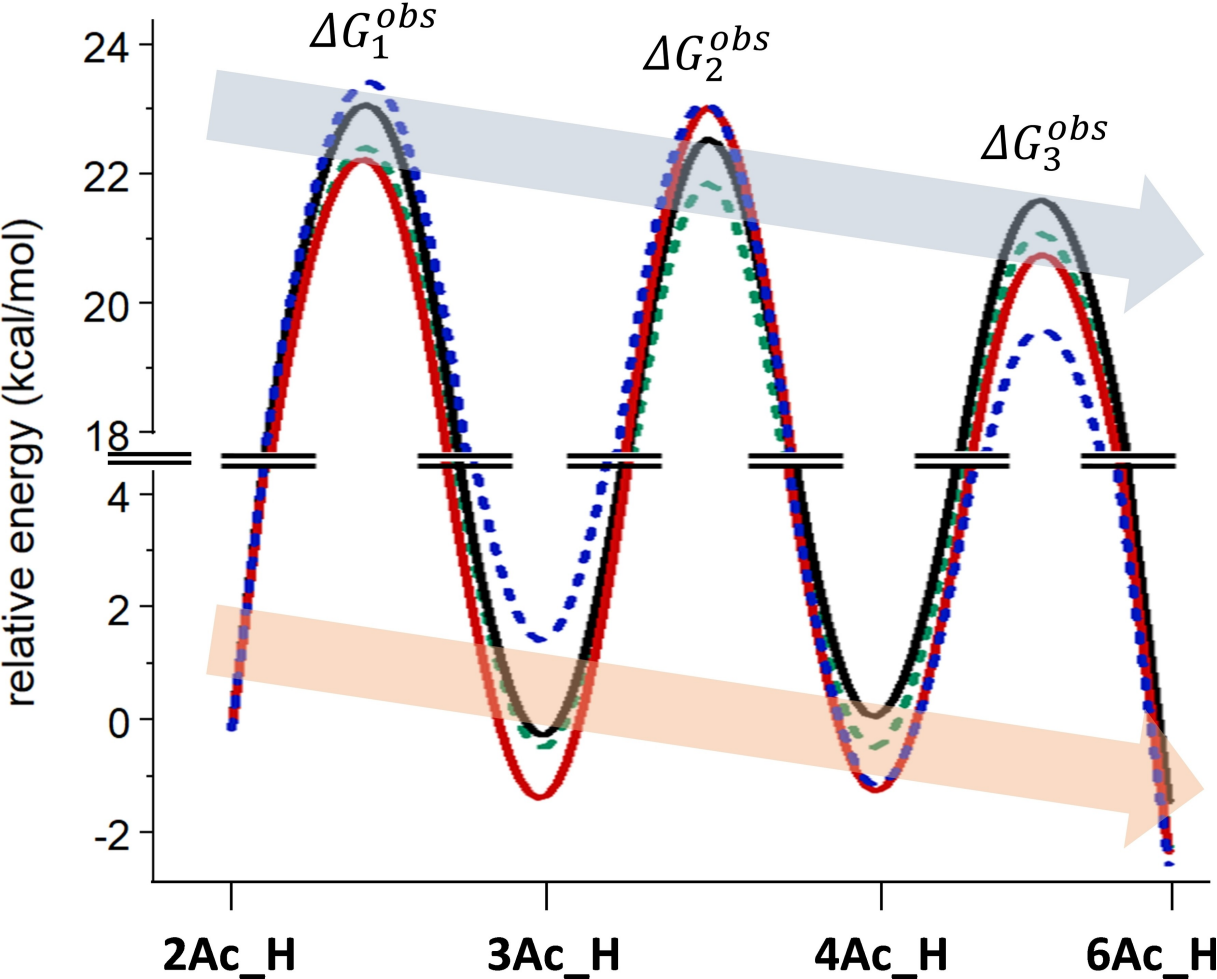
Experimental and calculated (m062x/cc‐pvtz/SMD=water//m062x/6‐31+G(d,p)/SMD=water) energy profiles for the whole migration process in Me *O*‐Ac‐α‐ and β‐d‐glucopyranosides. The maxima correspond to formal barriers (${\Delta G}_{i}^{obs}$ ) calculated from the rate constants $K_{i}^{obs}$ . The real barriers are those corresponding to two‐step processes with an intermediate orthoester as discussed above. Plain and dotted traces correspond to α‐ and β‐D‐glucopyranosides anomers, respectively. Black and red traces correspond to experimental and calculated values of Me *O*‐Ac‐α‐d‐glucopyranosides, respectively. Green and blue dotted traces correspond to experimental and calculated values of Me *O*‐Ac‐β‐d‐glucopyranosides, respectively.

This equation reflects that acyl migration takes place through an anionic mechanism but with a linear dependence on the pH and the pK_a_ of the hydroxyl group where the acyl group is migrating. In fact, analysis of the corresponding barriers and transition structures corresponding to the anionic mechanism only is not a suitable approach because of the different availability of intermediate species that are in equilibrium with the corresponding protonated forms through different values of pK_a_. On the other hand, it is possible to analyze the process considering the calculated formal barriers corresponding to the experimental values of $K_{i}^{obs}$ . Figure 8 illustrates a comparative energy diagram for the complete migration processes of Me *O*‐Ac‐α‐ and β‐d‐glucopyranosides; both experimental and calculated curves are included. This diagram clearly illustrates the close similarity of the energy values obtained for the different steps, within the range of 2 kcal/mol, which makes difficult to predict accurately the experimental values, as discussed above. The graphic also evidences the descending trend of both maxima (kinetic) and minima (thermodynamic) from O2‐Ac to O6‐Ac, supporting the observed increasing values of rate constants and the final formation of the O6‐Ac derivative, respectively.

The full model was further applied to acetyl group migration in acetylated Me d‐glycopyranosides with β‐gluco, α‐ and β‐galacto, and α‐ and β‐xylo configurations, resulting in very good agreement with the experimental observations (Table 7). In all cases, differences in formal free energy barriers were within the range of 2 kcal/mol for the selected level of theory.

**Table 7:** Calculated (formal energy barriers^[a]^ (kcal/mol) and rate constants^[b]^ (s^‐1^) for the acetyl group migration in Me *O*‐Ac‐d‐glycosides at pH=8.

		experimental		predicted		
		*rate constant*	*ΔG*	*rate constant*	*ΔG*	*ΔG error*
**β‐glc**	$k_{1}^{obs}$	2.02×10^−4^	22.5	2.72×10^−5^	23.7	1.2
	$k_{-1}^{obs}$	1.11×10^−4^	22.9	4.28×10^−4^	22.1	0.8
	$k_{2}^{obs}$	1.15×10^−4^	22.8	3.58×10^−4^	22.2	0.7
	$k_{-2}^{obs}$	1.28×10^−4^	22.8	4.71×10^−6^	24.7	2.0
	$k_{3}^{obs}$	1.10×10^−3^	21.5	5.48×10^−3^	20.6	1.0
	$k_{-3}^{obs}$	4.56×10^−5^	23.4	4.01×10^−4^	22.1	1.3
**α‐gal**	$k_{1}^{obs}$	3.31×10^−5^	23.6	2.99×10^−4^	22.3	1.3
	$k_{-1}^{obs}$	2.65×10^−5^	23.7	1.56×10^−4^	22.7	1.1
	$k_{2}^{obs}$	2.43×10^−4^	22.4	6.75×10^−3^	20.4	2.0
	$k_{-2}^{obs}$	2.43×10^−4^	22.4	2.94×10^−5^	23.7	1.3
	$k_{3}^{obs}$	2.83×10^−4^	22.3	4.83×10^−4^	22.0	0.3
	$k_{-3}^{obs}$	5.47×10^−5^	23.3	2.34×10^−4^	22.4	0.9
**β‐gal**	$k_{1}^{obs}$	1.47×10^−4^	22.7	1.19×10^−4^	22.8	0.1
	$k_{-1}^{obs}$	6.08×10^−5^	23.2	3.94×10^−5^	23.5	0.3
	$k_{2}^{obs}$	2.43×10^−4^	22.4	1.88×10^−4^	22.6	0.2
	$k_{-2}^{obs}$	1.72×10^−4^	22.6	8.78×10^−6^	24.4	1.8
	$k_{3}^{obs}$	2.10×10^−4^	22.5	1.49×10^−3^	21.3	1.2
	$k_{-3}^{obs}$	5.72×10^−5^	23.3	5.54×10^−5^	23.3	0.0
**α‐xyl**	$k_{1}^{obs}$	5.86×10^−5^	23.2	2.27×10^−4^	22.4	0.8
	$k_{-1}^{obs}$	4.25×10^−5^	23.4	9.44×10^−4^	21.6	1.8
	$k_{2}^{obs}$	2.03×10^−5^	23.9	1.47×10^−5^	24.1	0.2
	$k_{-2}^{obs}$	2.34×10^−5^	23.8	1.25×10^−4^	22.8	1.0
**β‐xyl**	$k_{1}^{obs}$	1.65×10^−4^	22.6	2.85×10^−5^	23.7	1.0
	$k_{-1}^{obs}$	7.33×10^−5^	23.1	3.52×10^−4^	22.2	0.9
	$k_{2}^{obs}$	9.11×10^−5^	23.0	3.27×10^−4^	22.2	0.8
	$k_{-2}^{obs}$	7.81×10^−5^	23.1	1.90×10^−3^	21.2	1.9

[a] Obtained from the individual barriers of the stepwise mechanism calculated at m062x/cc‐pvtz/SMD=water//m062x/6‐31+G(d,p)/SMD=water level of theory and then from the rate constants by using the Eyring's equation. [b] Obtained by applying equivalent Equations to (1) and (2) (see Supporting Information).

Examination of the geometries of the transition structures corresponding to the same transformation in α and β anomers did not show significant differences suggesting that the observed differences in the rate of migration are not due to steric reasons. Presumably, such differences are due to well‐known diverse electronic features of α and β anomers (anomeric effect) as mentioned.

## Conclusions

Here a comprehensive study of the acyl group migration in monosaccharides has been performed with some new conclusions made about the overall migration process. Configuration of the C1 has a significant impact on the rate of migration over hydroxyl groups in *trans* relationship, which has not been demonstrated earlier. It was also shown that when the hydroxyl groups share a *cis* relationship, the rate of migration is dependent on all of the properties of the acyl groups, while in a *trans* relationship the steric hindrance of the acyl group has a larger influence on the migration rate, due to the steric restriction of the carbohydrate ring. It was also demonstrated that the rate of migration is influenced by the buffer strengths, showing that if similar buffers are not used in the migration studies, the rates are not directly comparable. Computational calculations were utilized to establish a model, which confirms both the stepwise mechanism and the required previous deprotonation of the hydroxyl group towards which the acetyl group is migrating. This requirement makes the process dependent on both the pH and the pK_a_ of the corresponding hydroxyl group. The model, based on considering three explicit molecules of water surrounding the anion, reproduces this dependence and confirms the linearity experimentally observed with respect to the pH. Benchmarking using different levels of theory established m062x/cc‐pvtz/SMD=water as the best one for calculating energy values on geometries optimized at m062x/6‐31+G(d,p)/SMD=water level of theory.

## Experimental Section

**General**: For following the migration process a Bruker Avance‐III spectrometer operating at 500.20 MHz (^1^H) and 125.78 MHz (^13^C) equipped with a Smartprobe: BB/1H was used. The migration was followed with water suppressed ^1^H.

**Preparation of migration samples**: For monitoring the acetyl group migration by NMR spectroscopy, phosphate buffers with 10 % D_2_O were used. The main buffer strength used was 100 mM and other used was 50 and 500 mM. The pH was adjusted with NaOH or phosphoric acid if needed. A concentration of 1–2 mg/ml was used for the monosaccharide migration studies.

**Migration studies**: Due to the large set of compounds, the migrations were followed one time under each set of conditions. Ratios of products were obtained by using the NMR simulation program Chemadder/Spinadder.^[35]^ The peaks selected for determination of the product ratios by integration were: CH_3_ for Ac, (CH_3_)_3_ for Piv, CH for 2‐Ph‐propanoyls, the 2 and 6 protons in Ph for Bz (when separation was observed), and OMe in all of the experiments.

## Conflict of interest

The authors declare no conflict of interest.

1

## Supporting information

As a service to our authors and readers, this journal provides supporting information supplied by the authors. Such materials are peer reviewed and may be re‐organized for online delivery, but are not copy‐edited or typeset. Technical support issues arising from supporting information (other than missing files) should be addressed to the authors.

Supporting Information

## Data Availability

The data that support the findings of this study are available from the corresponding author upon reasonable request.
