# Supplementary material for: Acyl Group Migration in Pyranosides as Studied by Experimental and Computational Methods
Source: Chemistry. 2022 May 11;28(34):e202200499. doi: 10.1002/chem.202200499 (PMC9322027; doi:10.1002/chem.202200499)
Supplement: Supplementary file 1 — Supporting Information [file CHEM-28-0-s001.pdf]

Chemistry–A European Journal

Supporting Information

Acyl Group Migration in Pyranosides as Studied by Experimental and Computational Methods

Robert Lassfolk, Manuel Pedrón, Tomás Tejero, Pedro Merino,* Johan Wärnå, and Reko Leino*

Table of Contents

Synthesis of the investigated monosaccharides S3
Experimental procedures S5
Glucopyranoside derivatives S5
Galactopyranoside derivatives S10
Mannopyranoside derivatives S16
Xylopyranoside derivatives S18
Ribopyranoside derivatives S23
Arabinopyranoside derivatives S26
Rhamnopyranoside derivatives S28
Fucopyranoside derivatives S31
NMR spectra of the synthesized compounds S34
Migration Kinetics S141
Calculation of the rate constants S141
Fitting of the experimental data to the model S143
Glucopyranoside derivatives S143
Galactopyranoside derivatives S145
Mannopyranoside derivatives S147
Xylopyranoside derivatives S148
Ribopyranoside derivatives S150
Arabinopyranoside derivatives S151
Rhamnopyranoside derivatives S152
Fucopyranoside derivatives S153
Computational Studies S154
General Methods S154
Kinetics S156
Neutral mechanism S160

Anionic mechanism S161

pK_a calculations S165

Energy barriers and rate constants S167

Energies S173

Preliminary calculations S173

Model with three explicit molecules of water surrounding the anion S174

pKa values S184

Constant values S185

Transition Structures S187

Neutral mechanism with a one water molecules bridge S192

Neutral mechanism with one water molecules bridge S195

Naked anion S198

Naked anion with one explicit water molecule S201

Naked anion with two explicit water molecules S204

Naked anion with three explicit water molecules S208

D-Glucopyranoside S208

D-Galactopyranoside S236

D-Xylopyranoside S265

References S283

Synthesis of the investigated monosaccharides

Synthesis of the protected glucopyranosides for migration studies was carried out according to procedures described by Hung and coworkers, where the O2 position in Me 4,6-O-benzylidene-α-glucopyranoside was selectively acetylated.[1] This method could also be used for the other acyl groups. The same method was applied to the corresponding β-glucoside (Scheme S1 B), while in Me 4,6-O-benzylidene-α-galactopyranoside the O3 position was selectively acetylated. Consequently, the target galactosides were synthesized from the previously described protected galactosides **68** and **74** (Scheme S1 C for α-anomer and D for β-anomer).[2] Similarly, the protected mannosides were synthesized based on previously described protocols (Scheme S1 E).[3]

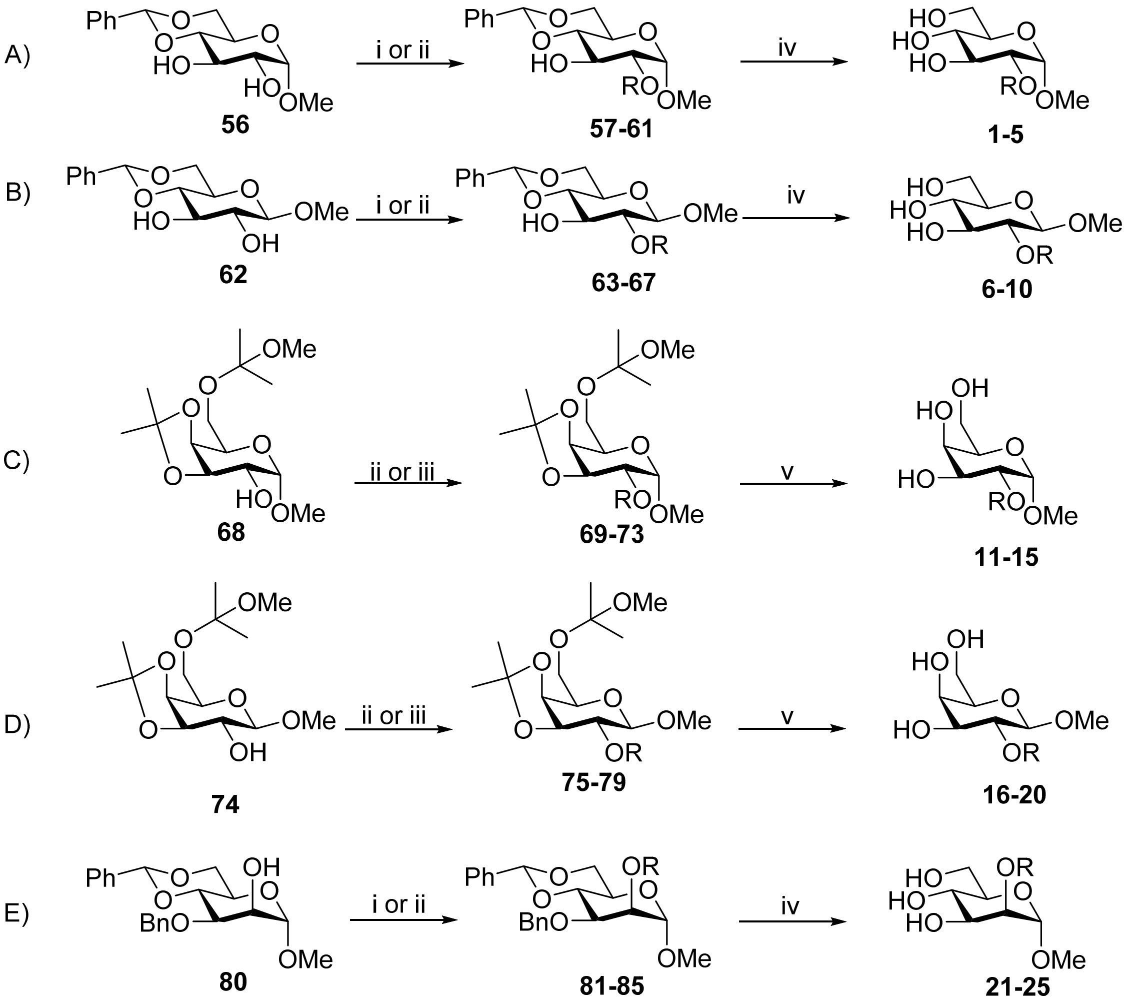

1, **6**, **11**, **16**, **21**, **57**, **63**, **69**, **75**, **81**: R = Ac
2, **7**, **12**, **17**, **22**, **58**, **64**, **70**, **76**, **82**: R = Bz
3, **8**, **13**, **18**, **23**, **59**, **65**, **71**, **77**, **83**: R = Piv
4, **9**, **14**, **19**, **24**, **60**, **66**, **72**, **78**, **84**: R = (*R*)-2-Ph-propanoate
5, **10**, **15**, **20**, **25**, **61**, **67**, **73**, **79**, **85**: R = (*S*)-2-Ph-propanoate

Scheme S1. Synthesis of the target glucopyranosides (A and B), galactopyranosides (C and D) and mannopyranosides (E): i) Et_3N, acylating agent (Ac_2O, BzCl, PivCl), CH_2Cl_2, r.t. 18h ii) DCC, DMAP, (R)- or (S)-2-phenyl-propionic acid, CH_2Cl_2, r.t. iii) acylating agent (Ac_2O, BzCl, PivCl), pyridine r.t. 18 h, iv) 10 m-% Pd/C, 30% AcOH in MeOH, 4 bar H_2, 3-4 h v) 80% AcOH in H_2O, 100 °C, 0.5 h.

The remaining compounds investigated, based on xylopyranoside (Scheme S2 A and B), arabinopyranoside (Scheme S2 C), ribopyranoside (Scheme S2 D), rhamnopyranoside (Scheme S2 E) and fucopyranoside (Scheme S2 F) derivatives, were synthesized by protecting two of the hydroxyl groups as isopropylidene acetals to leave one free hydroxyl group at O2 or O4 (**86**[4], **92**[5], **98**[6], **104**[7], **110**[8], **116**[9]). This free hydroxyl group was then acylated and the isopropylidene acetal cleaved under acidic conditions (Scheme S2).

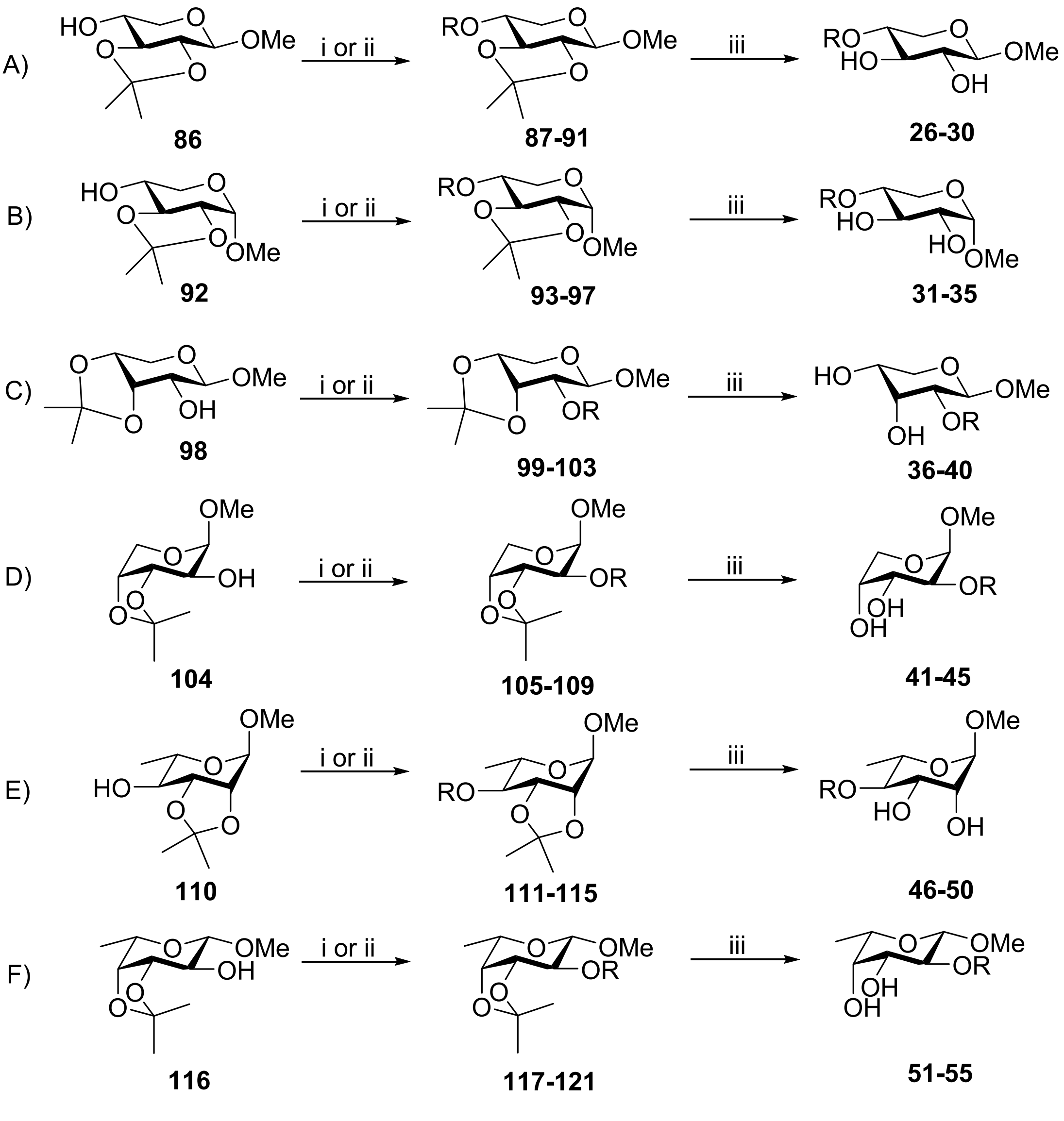

26, **31**, **36**, **41**, **46**, **51**, **87**, **93**, **99**, **105**, **111**, **117**: R = Ac
27, **32**, **37**, **42**, **47**, **52**, **88**, **94**, **100**, **106**, **112**, **118**: R = Bz
28, **33**, **38**, **43**, **48**, **53**, **89**, **95**, **101**, **107**, **113**, **119**: R = Piv
29, **34**, **39**, **44**, **49**, **54**, **90**, **96**, **102**, **108**, **114**, **120**: R = (*R*)-2-Ph-propanoate
30, **35**, **40**, **45**, **50**, **55**, **91**, **97**, **103**, **109**, **115**, **121**: R = (*S*)-2-Ph-propanoate

Scheme S2. Synthesis of the target xylopyranosides (A and B), arabinopyranoside (C), ribopyranoside, (D) rhamnopyranoside (E) and fucopyranoside (F): i) pyridine, acylating agent (Ac_2O, BzCl, PivCl), CH_2Cl_2, r.t. 18 h; ii) DCC, DMAP, (*R*)- or (*S*)-2-phenyl-propionic acid, CH_2Cl_2, r.t. 18 h; iii) 80% AcOH in H_2O, 60–100 °C, 30-45 min.

Experimental procedures

General considerations. For following the migration processes, a Bruker Avance-III spectrometer operating at 500.20 MHz (^{1}H) and 125.78 MHz (^{13}C) equipped with a Smartprobe: BB/1H, and for identification and characterization of the new compounds, a Bruker Avance-III spectrometer operating at 500.20 MHz (^{1}H) and 125.78 MHz (^{13}C) equipped with a Prodigy BBO CryoProbe, were used. The characterizations were performed using a standard set of 1D and 2D NMR spectroscopic techniques: ^{1}H, ^{13}C, DQF-COSY, Multiplicity edited HSQC (CH and CH_3 positive, CH_2 negative, both coupled and decoupled) and HMBC. The reported signals are referenced to an internal standard (TMS δ_H = 0.0 ppm, δ_C = 0.0 ppm) or residual solvent signal (CD_2HOD δ_H = 3.31 ppm, $CHCl_3$ δ_H = 7.26 ppm), or the solvent signal (CD_3OD δ_C = 49.00 ppm, $CDCl_3$ δ_C = 77.16 ppm).Chemical shifts are reported with two decimals for ^{1}H and one decimal for ^{13}C. Where this is not sufficient for distinguishing two signals, an additional decimal is given. Coupling constants are reported in Hz with one decimal and mentioned only the first time they are encountered. Accurate coupling constants and shifts were extracted from the ^{1}H spectra using the NMR simulation software ChemAdder/SpinAdder.[10] TLC analysis was performed on Merck silica gel 60 F254 plates and the spots were visualized with UV light and charring with H_2SO_4/MeOH (1:4) and heating. All reactions were monitored by TLC. Column chromatography was carried out using silica gel 60 (0.040 – 0.060 mm) as a stationary phase and hexane:EtOAc or toluene:EtOAc as eluents.

Standard reaction procedure A1 and A2 for acylation under basic conditions. To a solution of the corresponding monosaccharide substrate in CH_2Cl_2 (1 ml/50 mg) was added the acylation agent (1.1 equivalents) and Et_3N (9 equivalents) (**A1**). Alternatively, to a solution of the monosaccharide substrate in pyridine (1 ml/50 mg) was the acylation agent (3 equivalents) added (**A2**). The reaction was quenched by addition of MeOH after 18 h and the solvents were evaporated. The crude mixture was purified by column chromatography.

Standard reaction procedure B for acylation of acids. To a solution of the substrate in CH_2Cl_2 (1 ml/50 mg) was the acylation agent (1.1 equivalents), DCC (1.4 equivalents) and DMAP (0.1 equivalents) added. After 18 h was the solvents evaporated and the crude mixture was purified by column chromatography.

Standard reaction procedure C for hydrogenolysis of benzyl and benzylidene protecting groups. To a solution of the corresponding substrate in MeOH:AcOH 2:1 (1 ml/10 mg substrate) was added Pd/C 10% w/w (2 weight equivalents). The reaction mixture was stirred under 3 – 4 bar H_2-gas in an autoclave reactor for 4 h, after which the mixture was filtered and the solvent evaporated.

Standard reaction procedure D for removal of isopropylidene acetal and methoxy isopropyl protecting groups. A solution of the corresponding substrate in 80% AcOH (0.5 ml/10 mg substrate) was heated to 100 °C for 30 min. The solvent was evaporated. If, based on NMR-spectroscopy, impurities were present, the crude mixture was washed with EtOAc:hexane (ratio depending on the solubility) to remove.

Glucopyranoside derivatives

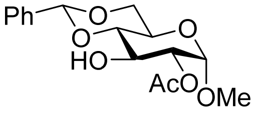

Methyl 2-*O*-acetyl-4,6-*O*-benzylidene-α-D-glucopyranoside (57): Synthesized from **56** (100 mg) according to standard reaction procedure A1. The crude product was purified by column chromatography (hexane:EtOAc 1:1) to

provide **57** as a white solid. Yield: 80 mg (70%), R_f = 0.32. ^{1}H NMR (500.20 MHz, $CDCl_3$, 25 °C): δ = 7.52 – 7.34 (m, 5 H, aromatic H), 5.55 (s, 1 H, 4,6-OC*H*Ph), 4.95 (d, 1 H, $J_{H-1,H-2}$ = 3.7 Hz, H-1), 4.80 (dd, 1 H, $J_{H-2,H-3}$ = 9.9 Hz, H-2), 4.30 (dd, 1 H, $J_{H-5,H-6a}$ = 5.0 Hz, $J_{H-6a,H-6b}$ = – 10.4 Hz, H-6a), 4.18 (ddd, 1 H, $J_{H-3,H-4}$ = 9.2 Hz, $J_{H-3,3-OH}$ = 0.8 Hz, H-3), 3.76 (dd, 1 H, $J_{H-5,H-6b}$ = 10.4 Hz, H-6b), 3.55 (dd, 1 H, $J_{H-4,H-5}$ = 9.7 Hz, H-4), 3.84 (ddd, 1 H, H-5), 3.40 (s, 3 H, 1-OCH_3), 2.55 (d, 1 H, 3-OH), 2.16 (s, 3 H, 2-OCOCH_3) ppm. ^{13}C NMR (125.78 MHz, $CDCl_3$, 25 °C): δ = 170.8 (2-*C*OCH$_3$), 137.1 – 126.4 (aromatic C), 102.2 (4,6-*C*HPh), 97.7 (C-1), 81.5 (C-4), 73.7 (C-2), 69.0 (C-6), 68.8 (C-3), 62.1 (C-5), 55.5 (1-O*C*H$_3$), 21.1 (2-CO*C*H$_3$) ppm.

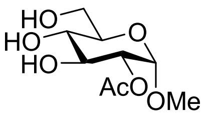

Methyl 2-*O*-acetyl-α-D-glucopyranoside (1): Synthesized from **57** (80 mg) according to the standard reaction procedure C to provide **1** as a white solid. Yield: 55 mg (94%). ^{1}H NMR (500.20 MHz, MeOD, 25 °C): δ = 4.84 (d, 1 H, $J_{H-1,H-2}$ = 3.7 Hz, H-1), 4.58 (dd, 1 H, $J_{H-2,H-3}$ = 9.9 Hz, H-2), 3.83 (dd, 1 H, $J_{H-5,H-6a}$ = 2.4 Hz, $J_{H-6a,H-6b}$ = – 11.9 Hz, H-6a), 3.80 (dd, 1 H, $J_{H-3,H-4}$ = 9.0 Hz, H-3), 3.69 (dd, 1 H, $J_{H-5,H-6b}$ = 5.7 Hz, H-6b), 3.54 (ddd, 1 H, $J_{H-4,H-5}$ = 10.0 Hz, H-5), 3.372 (dd, 1 H, H-4), 3.368 (s, 3 H, 1-OCH_3), 2.09 (s, 3 H, 2-OCOCH_3) ppm. ^{13}C NMR (125.78 MHz, MeOD, 25 °C): δ = 172.4 (2-*C*OCH$_3$), 98.3 (C-1), 75.0 (C-2), 73.4 (C-5), 72.3 (C-3), 71.7 (C-4), 62.5 (C-6), 55.4 (1-O*C*H$_3$), 20.8 (2-CO*C*H$_3$) ppm.

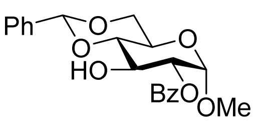

Methyl 2-*O*-benzoyl-4,6-*O*-benzylidene-α-D-glucopyranoside (58): Synthesized from **56** (40 mg) according to standard reaction procedure A1. The crude product was purified by column chromatography (hexane:EtOAc 3:1) to provide **58** as a white solid. Yield: 89 mg (65%), R_f = 0.25. ^{1}H NMR (500.20 MHz, $CDCl_3$, 25 °C): δ = 8.12 – 7.35 (m, 10 H, aromatic H), 5.57 (s, 1 H, 4,6-OC*H*Ph), 5.08 (d, 1 H, $J_{H-1,H-2}$ = 3.8 Hz, H-1), 5.04 (dd, 1 H, $J_{H-2,H-3}$ = 9.7 Hz, H-2), 4.35 (ddd, 1 H, $J_{H-3,H-4}$ = 9.3 Hz, $J_{H-3,3-OH}$ = 2.7 Hz, H-3), 4.33 (dd, 1 H, $J_{H-5,H-6a}$ = 4.9 Hz, $J_{H-6a,H-6b}$ = – 10.4 Hz, H-6a), 3.91 (ddd, 1 H, $J_{H-4,H-5}$ = 9.6 Hz, $J_{H-5,H-6b}$ = 10.4 Hz, H-5), 3.80 (dd, 1 H, H-6b), 3.63 (dd, 1 H, H-4), 3.40 (s, 3 H, 1-OCH_3), 2.57 (d, 1 H, 3-OH) ppm. ^{13}C NMR (125.78 MHz, $CDCl_3$, 25 °C): δ = 166.4 (2-*C*OPh), 137.1 – 126.5 (aromatic C), 102.2 (4,6-*C*HPh), 97.9 (C-1), 81.6 (C-4), 74.2 (C-2), 69.04 (C-6), 68.96 (C-3), 62.2 (C-5), 55.6 (1-O*C*H$_3$) ppm.

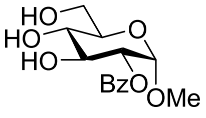

Methyl 2-*O*-benzoyl-α-D-glucopyranoside (2): Synthesized from **58** (80 mg) according to the standard reaction procedure C to provide **2** as a white solid. Yield: 30 mg (97%). ^{1}H NMR (500.20 MHz, MeOD, 25 °C): δ = 8.10 – 8.06 (m, 2 H, aromatic H), 7.64 – 7.59 (m, 1 H, aromatic), 7.51 – 7.46 (m, 2 H, aromatic), 4.99 (d, 1 H, $J_{H-1,H-2}$ = 3.7 Hz, H-1), 4.83 (dd, 1 H, $J_{H-2,H-3}$ = 9.9 Hz, H-2), 3.98 (dd, 1 H, $J_{H-3,H-4}$ = 8.9 Hz, H-3), 3.87 (dd, 1 H, $J_{H-5,H-6a}$ = 2.3 Hz, $J_{H-6a,H-6b}$ = – 11.9 Hz, H-6a), 3.73 (dd, 1 H, $J_{H-5,H-6b}$ = 5.7 Hz, H-6b), 3.62 (ddd, 1 H, $J_{H-4,H-5}$ = 10.0 Hz, H-5), 3.45 (dd, 1 H, H-4), 3.39 (s, 3 H, 1-OCH_3) ppm. ^{13}C NMR (125.78 MHz, MeOD, 25 °C): δ = 167.7 (2-*C*OPh), 134.4, 131.2, 130.8, 129.5 (aromatic C), 98.5 (C-1), 75.5 (C-2), 73.6 (C-5), 72.5 (C-3), 71.9 (C-4), 62.6 (C-6), 55.5 (1-O*C*H$_3$) ppm.

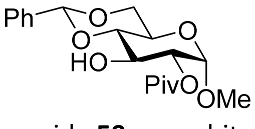

Methyl 4,6-*O*-benzylidene-2-*O*-pivaloyl-α-D-glucopyranoside (59): Synthesized from **56** (40 mg) according to standard reaction procedure A1. The crude product was purified by column chromatography (hexane:EtOAc 3:1) to provide **59** as a white solid. Yield: 38 mg (29%), R_f = 0.26. ^{1}H NMR (500.20 MHz, $CDCl_3$, 25 °C): δ = 7.52 – 7.34 (m, 5 H, aromatic H), 5.56 (s, 1 H, 4,6-OC*H*Ph), 4.94 (d, 1 H, $J_{H-1,H-2}$ = 3.8 Hz, H-1), 4.74 (dd, 1 H, $J_{H-2,H-3}$ = 9.7 Hz, H-2), 4.29 (dd, 1 H, $J_{H-5,H-6a}$ = 5.0 Hz, $J_{H-6a,H-6b}$ = – 10.4 Hz, H-6a), 4.20 (ddd, 1 H, $J_{H-3,H-4}$ = 9.3 Hz, $J_{H-3,3-OH}$ = 2.7 Hz, H-3), 3.85 (ddd, 1 H, $J_{H-4,H-5}$ = 9.6 Hz, $J_{H-5,H-6b}$ = 10.4 Hz, H-5), 3.77 (dd, 1 H, H-6b),

3.56 (dd, 1 H, H-4), 3.39 (s, 3 H, 1-OC*H*$_3$), 2.33 (d, 1 H, 3-OH), 1.25 (s, 9 H, 2-COC*(CH$_3$)$_3$*) ppm. ^{13}C NMR (125.78 MHz, CDCl$_3$, 25 °C): δ = 178.3 (2-*C*OC(CH$_3$)$_3$), 137.2 – 126.4 (aromatic C), 102.1 (4,6-*C*HPh), 97.8 (C-1), 81.5 (C-4), 73.6 (C-2), 69.1 (C-6), 69.0 (C-3), 62.2 (C-5), 55.8 (1-O*C*H$_3$), 39.0 (2-CO*C*(CH$_3$)$_3$), 27.2 (2-COC*(CH$_3$)$_3$*) ppm.

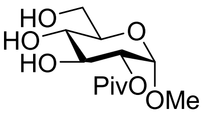

Methyl 2-*O*-pivaloyl-α-D-glucopyranoside (3): Synthesized from **59** (17 mg) according to the standard reaction procedure C to provide **3** as a white solid. Yield: 12 mg (93%). ^{1}H NMR (500.20 MHz, MeOD, 25 °C): δ = 4.82 (d, 1 H, $J_{H\text{-}1,H\text{-}2}$ = 3.7 Hz, H-1), 4.54 (dd, 1 H, $J_{H\text{-}2,H\text{-}3}$ = 9.9 Hz, H-2), 3.83 (dd, 1 H, $J_{H\text{-}5,H\text{-}6a}$ = 2.3 Hz, $J_{H\text{-}6a,H\text{-}6b}$ = – 11.9 Hz, H-6a), 3.81 (dd, 1 H, $J_{H\text{-}3,H\text{-}4}$ = 9.0 Hz, H-3), 3.69 (dd, 1 H, $J_{H\text{-}5,H\text{-}6b}$ = 5.7 Hz, H-6b), 3.56 (ddd, 1 H, $J_{H\text{-}4,H\text{-}5}$ = 10.0 Hz,H-5), 3.37 (s, 3 H, 1-OC*H*$_3$), 3.36 (dd, 1 H, H-4), 1.22 (s, 9 H, 2-COC(*CH$_3$)$_3$* ppm. ^{13}C NMR (125.78 MHz, MeOD, 25 °C): δ = 179.8 (2-*C*OC(CH$_3$)$_3$), 98.4 (C-1), 74.8 (C-2), 73.5 (C-5), 72.4 (C-3), 72.0 (C-4), 62.6 (C-6), 55.3 (1-O*C*H$_3$), 39.9 (2-CO*C*(CH$_3$)$_3$), 27.4 (2-COC*(CH$_3$)$_3$* ppm.

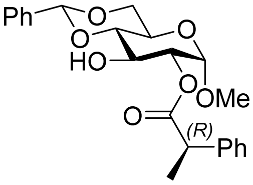

Methyl 2-*O*-(*R*)-2-phenyl-propanoyl-4,6-*O*-benzylidene-α-D-glucopyranoside (60): Synthesized from **56** (100 mg) according to the standard reaction procedure B. The crude product was purified by column chromatography (toluene:EtOAc 7:1) to obtain **60** as a clear oil. Yield: 22 mg (15%), R$_f$ = 0.22. ^{1}H NMR (500.20 MHz, CDCl$_3$, 25 °C): δ = 7.51 – 7.14 (m, 10 H, aromatic H), 5.53 (s, 1 H, 4,6-OC*H*Ph), 4.78 (dd, 1 H, $J_{H\text{-}1,H\text{-}2}$ = 3.8 Hz, $J_{H\text{-}2,H\text{-}3}$ = 9.2 Hz, H-2), 4.77 (d, 1 H, H-1), 4.26 (dd, 1 H, $J_{H\text{-}5,H\text{-}6a}$ = 5.0 Hz, $J_{H\text{-}6a,H\text{-}6b}$ = – 10.4 Hz, H-6a), 4.17 (ddd, 1 H, $J_{H\text{-}3,H\text{-}4}$ = 9.3 Hz, $J_{H\text{-}3,3\text{-}OH}$ = 2.9 Hz, H-3), 3.85 (ddd, 1 H, $J_{2\text{-}CO\underline{CH}CH3Ph,\ 2\text{-}COCH\underline{CH3}Ph}$ = 7.1 Hz, 2-COC*H*CH$_3$Ph), 3.80 (ddd, 1 H, $J_{H\text{-}4,H\text{-}5}$ = 9.6 Hz, $J_{H\text{-}5,H\text{-}6b}$ = 10.4 Hz, H-5), 3.73 (dd, 1 H, H-6b), 3.54 (dd, 1 H, H-4), 3.12 (s, 3 H, 1-OC*H*$_3$), 2.38 (d, 1 H, 3-OH), 1.54 (d, 3 H, 2-COCHC*H$_3$*Ph) ppm. ^{13}C NMR (125.78 MHz, CDCl$_3$, 25 °C): δ = 174.4 (2-*C*OCHCH$_3$Ph), 140.3 – 126.4 (aromatic C), 102.2 (4,6-*C*HPh), 97.6 (C-1), 81.5 (C-4), 73.9 (C-2), 69.0 (C-6), 68.8 (C-3), 62.1 (C-5), 55.6 (1-O*C*H$_3$), 45.3 (2-CO*C*HCH$_3$Ph), 18.3 (2-COCHC*H$_3$*Ph) ppm.

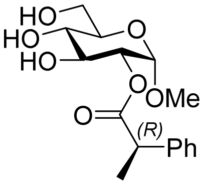

Methyl 2-*O*-(*R*)-2-phenyl-propanoyl-α-D-glucopyranoside (4): Synthesized from **60** (10 mg) according to the standard reaction procedure C to obtain **4** as a white solid. Yield: 6 mg (76%). ^{1}H NMR (500.20 MHz, CDCl$_3$, 25 °C): δ = 7.36 – 7.21 (m, 5 H, aromatic H), 4.65 (d, 1 H, $J_{H\text{-}1,H\text{-}2}$ = 3.7 Hz, H-1), 4.58 (dd, 1 H, $J_{H\text{-}2,H\text{-}3}$ = 10.0 Hz, H-2), 3.83 (ddd, 1 H, $J_{2\text{-}CO\underline{CH}CH3Ph,\ 2\text{-}COCH\underline{CH3}Ph}$ = 7.1 Hz, 2-COC*H*CH$_3$Ph), 3.79 (dd, 1 H, $J_{H\text{-}5,H\text{-}6a}$ = 2.3 Hz, $J_{H\text{-}6a,H\text{-}6b}$ = – 12.0 Hz, H-6a), 3.78 (dd, 1 H, $J_{H\text{-}3,H\text{-}4}$ = 8.9 Hz, H-3), 3.66 (dd, 1 H, $J_{H\text{-}5,H\text{-}6b}$ = 5.7 Hz, H-6b), 3.50 (ddd, 1 H, $J_{H\text{-}4,H\text{-}5}$ = 9.9 Hz, H-5), 3.35 (dd, 1 H, H-4), 3.11 (s, 3 H, 1-OC*H*$_3$), 1.47 (d, 3 H, 2-COCHC*H$_3$*Ph) ppm. ^{13}C NMR (125.78 MHz, CDCl$_3$, 25 °C): δ = 176.0 (2-*C*OCHCH$_3$Ph), 142.0, 129.6, 128.5, 128.1 (aromatic C), 98.2 (C-1), 75.2 (C-2), 73.5 (C-5), 72.2 (C-3), 71.8 (C-4), 62.5 (C-6), 55.6 (1-O*C*H$_3$), 46.4 (2-CO*C*HCH$_3$Ph), 18.9 (2-COCHC*H$_3$*Ph) ppm.

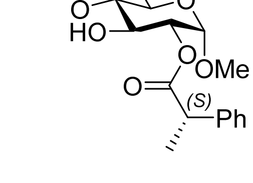

Methyl 2-*O*-(*S*)-2-phenyl-propanoyl-4,6-*O*-benzylidene-α-D-glucopyranoside (61): Synthesized from **56** (100 mg) according to the standard reaction procedure B. The crude product was purified by column chromatography (toluene:EtOAc 7:1) to obtain **61** as a clear oil. Yield: 69 mg (47%), R$_f$ = 0.33. ^{1}H NMR (500.20 MHz, CDCl$_3$, 25 °C): δ = 7.48 – 7.22 (m, 10 H, aromatic H), 5.52 (s, 1 H, 4,6-OC*H*Ph), 4.93 (d, 1 H, $J_{H\text{-}1,H\text{-}2}$ = 3.8 Hz, H-1), 4.71 (dd, 1 H, $J_{H\text{-}2,H\text{-}3}$ = 9.6 Hz, H-2), 4.28 (dd, 1 H, $J_{H\text{-}5,H\text{-}6a}$ = 5.0 Hz, $J_{H\text{-}6a,H\text{-}6b}$ = – 10.4 Hz, H-6a), 4.09 (ddd, 1 H, $J_{H\text{-}3,H\text{-}4}$ = 9.3 Hz, $J_{H\text{-}3,3\text{-}OH}$ = 2.9 Hz, H-3), 3.84 (ddd,

1 H, $J_{2\text{-CO}\underline{\text{CH}}\text{CH3Ph, 2-COCH}\underline{\text{CH3}}\text{Ph}}$ = 7.1 Hz, 2-CO*CH*CH$_3$Ph), 3.82 (ddd, 1 H, $J_{\text{H-4,H-5}}$ = 9.6 Hz, $J_{\text{H-5,H-6b}}$ = 10.4 Hz, H-5), 3.75 (dd, 1 H, H-6b), 3.53 (dd, 1 H, H-4), 3.38 (s, 3 H, 1-OC*H*$_3$), 1.87 (d, 1 H, 3-OH), 1.54 (d, 3 H, 2-COCHC*H*$_3$Ph) ppm. ^{13}C NMR (125.78 MHz, CDCl$_3$, 25 °C): δ = 174.3 (2-*C*OCHCH$_3$Ph), 140.5 – 126.4 (aromatic C), 102.1 (4,6-*C*HPh), 97.8 (C-1), 81.1 (C-4), 74.3 (C-2), 69.0 (C-6), 68.9 (C-3), 62.2 (C-5), 55.6 (1-O*C*H$_3$), 45.6 (2-CO*C*HCH$_3$Ph), 18.3 (2-COCH*C*H$_3$Ph) ppm.

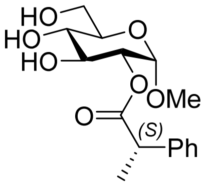

Methyl 2-*O*-(*S*)-2-phenyl-propanoyl-α-D-glucopyranoside (5): Synthesized from **61** (20 mg) according to the standard reaction procedure C to obtain **5** as a white solid. Yield: 11 mg (70%). ^{1}H NMR (500.20 MHz, CDCl$_3$, 25 °C): δ = 7.36 – 7.21 (m, 5 H, aromatic H), 4.83 (d, 1 H, $J_{\text{H-1,H-2}}$ = 3.7 Hz, H-1), 4.57 (dd, 1 H, $J_{\text{H-2,H-3}}$ = 10.1 Hz, H-2), 3.81 (ddd, 1 H, $J_{2\text{-CO}\underline{\text{CH}}\text{CH3Ph, 2-COCH}\underline{\text{CH3}}\text{Ph}}$ = 7.2 Hz, 2-CO*CH*CH$_3$Ph), 3.82 (dd, 1 H, $J_{\text{H-5,H-6a}}$ = 2.3 Hz, $J_{\text{H-6a,H-6b}}$ = – 11.9 Hz, H-6a), 3.80 (dd, 1 H, $J_{\text{H-3,H-4}}$ = 8.9 Hz, H-3), 3.68 (dd, 1 H, $J_{\text{H-5,H-6b}}$ = 5.7 Hz, H-6b), 3.55 (ddd, 1 H, $J_{\text{H-4,H-5}}$ = 9.9 Hz, H-5), 3.342 (s, 3 H, 1-OC*H*$_3$), 3.341 (dd, 1 H, H-4), 1.50 (d, 3 H, 2-COCHC*H*$_3$Ph) ppm. ^{13}C NMR (125.78 MHz, CDCl$_3$, 25 °C): δ = 175.9 (2-*C*OCHCH$_3$Ph), 141.9, 129.5, 128.7, 128.1 (aromatic C), 98.3 (C-1), 75.2 (C-2), 73.5 (C-5), 72.3 (C-3), 71.9 (C-4), 62.5 (C-6), 55.5 (1-O*C*H$_3$), 46.8 (2-CO*C*HCH$_3$Ph), 19.2 (2-COCH*C*H$_3$Ph) ppm.

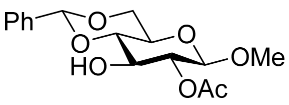

Methyl 2-*O*-acetyl-4,6-*O*-benzylidene-β-D-glucopyranoside (63): Synthesized from **62** (50 mg) according to standard reaction procedure A1. The crude product was purified by column chromatography (hexane:EtOAc 1:1) to provide **63** as a white solid. Yield: 19 mg (33%), R$_f$ = 0.36. ^{1}H NMR (500.20 MHz, CDCl$_3$, 25 °C): δ = 7.51 – 7.34 (m, 5 H, aromatic H), 5.56 (s, 1 H, 4,6-OC*H*Ph), 4.93 (dd, 1 H, $J_{\text{H-1,H-2}}$ = 7.9 Hz, $J_{\text{H-2,H-3}}$ = 9.2 Hz, H-2), 4.45 (d, 1 H, H-1), 4.38 (dd, 1 H, $J_{\text{H-5,H-6a}}$ = 5.0 Hz, $J_{\text{H-6a,H-6b}}$ = – 10.6 Hz, H-6a), 3.90 (ddd, 1 H, $J_{\text{H-3,H-4}}$ = 9.3 Hz, $J_{\text{H-3,3-OH}}$ = 0.8 Hz, H-3), 3.81 (dd, 1 H, $J_{\text{H-5,H-6b}}$ = 10.1 Hz, H-6b), 3.60 (dd, 1 H, $J_{\text{H-4,H-5}}$ = 9.4 Hz, H-4), 3.52 (s, 3 H, 1-OC*H*$_3$), 3.47 (ddd, 1 H, H-5), 2.57 (d, 1 H, 3-OH), 2.09 (s, 3 H, 2-OCOC*H*$_3$) ppm. ^{13}C NMR (125.78 MHz, CDCl$_3$, 25 °C): δ = 170.5 (2-*C*OCH$_3$), 137.0 – 126.4 (aromatic C), 102.4 (C-1), 102.1 (4,6-*C*HPh),81.1 (C-4), 74.2 (C-2), 72.4 (C-3), 68.8 (C-6), 66.3 (C-5), 57.3 (1-O*C*H$_3$), 21.1 (2-CO*C*H$_3$) ppm.

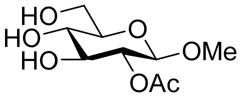

Methyl 2-*O*-acetyl-β-D-glucopyranoside (6): Synthesized from **63** (10 mg) according to the standard reaction procedure C to provide **6** as a white solid. Yield: 6mg (82%). ^{1}H NMR (500.20 MHz, MeOD, 25 °C): δ = 4.68 (dd, 1 H, $J_{\text{H-1,H-2}}$ = 8.0 Hz, $J_{\text{H-2,H-3}}$ = 9.6 Hz, H-2), 4.35 (d, 1 H, H-1), 3.88 (dd, 1 H, $J_{\text{H-5,H-6a}}$ = 2.3 Hz, $J_{\text{H-6a,H-6b}}$ = – 12.0 Hz, H-6a), 3.69 (dd, 1 H, $J_{\text{H-5,H-6b}}$ = 5.9 Hz, H-6b), 3.50 (dd, 1 H, $J_{\text{H-3,H-4}}$ = 9.0 Hz, H-3), 3.47 (s, 3 H, 1-OC*H*$_3$), 3.36 (dd, 1 H, $J_{\text{H-4,H-5}}$ = 99.9 Hz, H-4), 3.30 (ddd, 1 H, H-5), 2.08 (s, 3 H, 2-OCOC*H*$_3$) ppm. ^{13}C NMR (125.78 MHz, MeOD, 25 °C): δ = 171.9 (2-*C*OCH$_3$), 103.1 (C-1), 78.1 (C-5), 76.2 (C-3), 75.2 (C-2), 71.6 (C-4), 62.6 (C-6), 57.0 (1-O*C*H$_3$), 20.9 (2-CO*C*H$_3$) ppm.

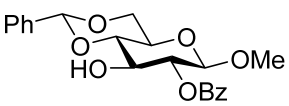

Methyl 2-*O*-benzoyl-4,6-*O*-benzylidene-β-D-glucopyranoside (64): Synthesized from **62** (40 mg) according to standard reaction procedure A1. The crude product was purified by column chromatography (hexane:EtOAc 3:1) to provide **64** as a white solid. Yield: 16 mg (23%), R$_f$ = 0.33. ^{1}H NMR (500.20 MHz, CDCl$_3$, 25 °C): δ = 8.12 – 7.34 (m, 10 H, aromatic H), 5.59 (s, 1 H, 4,6-OC*H*Ph), 5.19 (dd, 1 H, $J_{\text{H-1,H-2}}$ = 7.8 Hz, $J_{\text{H-2,H-3}}$ = 9.1 Hz, H-2), 4.62 (d, 1 H, H-1), 4.41 (dd, 1 H, $J_{\text{H-5,H-6a}}$ = 5.1 Hz, $J_{\text{H-6a,H-6b}}$ = – 10.5 Hz, H-6a), 4.07 (ddd, 1 H, $J_{\text{H-3,H-4}}$ = 9.2 Hz, $J_{\text{H-3,3-OH}}$ = 3.3 Hz, H-3), 3.85 (dd, 1 H, $J_{\text{H-5,H-6b}}$ = 10.2 Hz, H-6b), 3.69 (dd, 1 H, $J_{\text{H-4,H-5}}$ = 9.6 Hz, H-4), 3.55 (ddd, 1 H, H-5), 3.52 (s, 3 H, 1-OC*H*$_3$), 2.64 (d, 1 H, 3-OH) ppm. ^{13}C NMR (125.78 MHz, CDCl$_3$,

25 °C): δ = 166.1 (2-*C*OPh), 137.0 – 126.4 (aromatic C), 102.6 (C-1), 102.1 (4,6-*C*HPh), 81.1 (C-4), 74.9 (C-2), 72.6 (C-3), 68.8 (C-6), 66.3 (C-5), 57.4 (1-OCH_3) ppm.

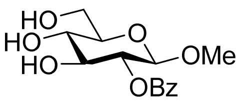

Methyl 2-*O*-benzoyl-β-D-glucopyranoside (7): Synthesized from **64** (10 mg) according to the standard reaction procedure C to provide **7** as a white solid. Yield: 6.4 mg (83%). ^{1}H NMR (500.20 MHz, MeOD, 25 °C): δ = 8.08 – 8.03 (m, 2 H, aromatic H), 7.65 – 7.59 (m, 1 H, aromatic), 7.52 – 7.46 (m, 2 H, aromatic), 4.94 (dd, 1 H, $J_{H-1,H-2}$ = 8.0 Hz,$J_{H-2,H-3}$ = 9.6 Hz, H-2), 4.53 (d, 1 H, H-1), 3.92 (dd, 1 H, $J_{H-5,H-6a}$ = 2.3 Hz, $J_{H-6a,H-6b}$ = – 12.0 Hz, H-6a), 3.73 (dd, 1 H, $J_{H-5,H-6b}$ = 5.8 Hz, H-6b), 3.68 (dd, 1 H, $J_{H-3,H-4}$ = 9.0 Hz, H-3), 3.47 (s, 3 H, 1-OCH_3), 3.44 (dd, 1 H, $J_{H-4,H-5}$ = 9.8 Hz, H-4), 3.38 (ddd, 1 H, H-5) ppm. ^{13}C NMR (125.78 MHz, MeOD, 25 °C): δ = 167.3 (2-*C*OPh), 134.3, 131.5, 130.7, 129.5 (aromatic C), 103.3 (C-1), 78.2 (C-5), 76.2 (C-3), 75.8 (C-2), 71.8 (C-4), 62.7 (C-6), 57.1 (1-OCH_3) ppm.

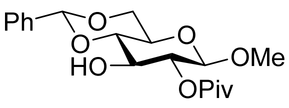

Methyl 4,6-*O*-benzylidene-2-*O*-pivaloyl-β-D-glucopyranoside (65): Synthesized from **62** (40 mg) according to standard reaction procedure A1. The crude product was purified by column chromatography (hexane:EtOAc 3:1) to provide **65** as a white solid. Yield: 9 mg (12%), R_f = 0.36. ^{1}H NMR (500.20 MHz, $CDCl_3$, 25 °C): δ = 7.53 – 7.33 (m, 5 H, aromatic H), 5.56 (s, 1 H, 4,6-OC*H*Ph), 4.88 (dd, 1 H, $J_{H-1,H-2}$ = 7.8 Hz, $J_{H-2,H-3}$ = 9.1 Hz, H-2), 4.45 (d, 1 H, H-1), 4.37 (dd, 1 H, $J_{H-5,H-6a}$ = 5.0 Hz, $J_{H-6a,H-6b}$ = – 10.5 Hz, H-6a), 3.90 (ddd, 1 H, $J_{H-3,H-4}$ = 9.3 Hz, $J_{H-3,3-OH}$ = 1.6 Hz, H-3), 3.81 (dd, 1 H, $J_{H-5,H-6b}$ = 10.1 Hz, H-6b), 3.61 (dd, 1 H, $J_{H-4,H-5}$ = 9.4 Hz, H-4), 3.51 (s, 3 H, 1-OCH_3), 3.47 (ddd, 1 H, H-5), 2.56 (d, 1 H, 3-OH), 1.24 (s, 9 H, 2-COC*(CH$_3$)$_3$*) ppm. ^{13}C NMR (125.78 MHz, $CDCl_3$, 25 °C): δ = 178.1 (2-*C*OC(CH_3)$_3$), 137.1 – 126.4 (aromatic C), 102.7 (C-1), 102.0 (4,6-*C*HPh), 81.1 (C-4), 74.2 (C-2), 72.7 (C-3), 68.8 (C-6), 66.3 (C-5), 57.4 (1-OCH_3), 39.1 (2-CO*C*(CH_3)$_3$), 27.2 (2-COC*(CH$_3$)$_3$*) ppm.

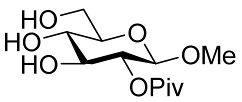

Methyl 2-*O*-pivaloyl-β-D-glucopyranoside (8): Synthesized from **65** (9 mg) according to the standard reaction procedure C to provide **8** as a white solid. Yield: 6 mg (93%). ^{1}H NMR (500.20 MHz, MeOD, 25 °C): δ = 4.67 (dd, 1 H, $J_{H-1,H-2}$ = 8.0 Hz, $J_{H-2,H-3}$ = 9.7 Hz, H-2), 4.35 (d, 1 H, H-1), 3.89 (dd, 1 H, $J_{H-5,H-6a}$ = 2.3 Hz, $J_{H-6a,H-6b}$ = – 12.0 Hz, H-6a), 3.69 (dd, 1 H, $J_{H-5,H-6b}$ = 5.9 Hz, H-6b), 3.51 (dd, 1 H, $J_{H-3,H-4}$ = 8.9 Hz, H-3), 3.47 (s, 3 H, 1-OCH_3), 3.35 (dd, 1 H, $J_{H-4,H-5}$ = 9.9 Hz, H-4), 3.31 (ddd, 1 H, H-5), 1.22 (s, 9 H, 2-COC(*CH$_3$)$_3$* ppm. ^{13}C NMR (125.78 MHz, MeOD, 25 °C): δ = 179.2 (2-*C*OC(CH_3)$_3$), 103.5 (C-1), 78.1 (C-5), 76.1 (C-3), 74.9 (C-2), 71.9 (C-4), 62.6 (C-6), 57.2 (1-OCH_3), 39.9 (2-CO*C*(CH_3)$_3$), 27.5 (2-COC*(CH$_3$)$_3$* ppm.

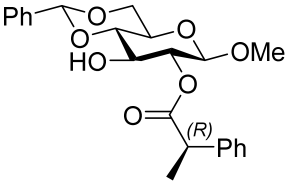

Methyl 2-*O*-(*R*)-2-phenyl-propanoyl-4,6-*O*-benzylidene-β-D-glucopyranoside (66): Synthesized from **62** (100 mg) according to the standard reaction procedure B. The crude product was purified by column chromatography (toluene:EtOAc 7:1) to obtain **66** as a white solid. Yield: 80 mg (23%), R_f = 0.20. ^{1}H NMR (500.20 MHz, $CDCl_3$, 25 °C): δ = 7.50 – 7.22 (m, 10 H, aromatic H), 5.54 (s, 1 H, 4,6-OC*H*Ph), 4.90 (dd, 1 H, $J_{H-1,H-2}$ = 7.9 Hz, $J_{H-2,H-3}$ = 9.2 Hz, H-2), 4.33 (dd, 1 H, $J_{H-5,H-6a}$ = 5.0 Hz, $J_{H-6a,H-6b}$ = – 10.6 Hz, H-6a), 4.19 (d, 1 H, H-1), 3.87 (ddd, 1 H, $J_{H-3,H-4}$ = 9.3 Hz, $J_{H-3,3-OH}$ = 3.3 Hz, H-3), 3.80 (ddd, 1 H, $J_{2-CO\underline{CH}CH3Ph,\ 2-COCH\underline{CH3}Ph}$ = 7.1 Hz, 2-COC*H*CH$_3$Ph), 3.77 (dd, 1 H, $J_{H-5,H-6b}$ = 10.1 Hz, H-6b), 3.58 (dd, 1 H, $J_{H-4,H-5}$ = 9.4 Hz, H-4), 3.38 (ddd, 1 H, H-5), 3.18 (s, 3 H, 1-OCH_3), 2.42 (d, 1 H, 3-OH), 1.52 (d, 3 H, 2-COCHC*H$_3$*Ph) ppm. ^{13}C NMR (125.78 MHz, $CDCl_3$, 25 °C): δ = 173.7 (2-

*C*OCHCH$_3$Ph), 140.7 – 126.4 (aromatic C), 102.7 (C-1), 102.1 (4,6-*C*HPh), 81.0 (C-4), 74.4 (C-2), 72.2 (C-3), 68.7 (C-6), 66.2 (C-5), 57.4 (1-O*C*H$_3$), 45.7 (2-CO*C*HCH$_3$Ph), 18.5 (2-COCHC*H$_3$*Ph) ppm.

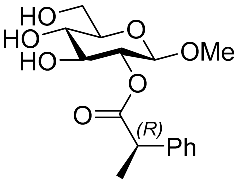

Methyl 2-*O*-(*R*)-2-phenyl-propanoyl-β-D-glucopyranoside (4): Synthesized from **66** (10 mg) according to the standard reaction procedure C to obtain **4** as a white solid. Yield: 7 mg (89%). ^{1}H NMR (500.20 MHz, CDCl$_3$, 25 °C): δ = 7.34 – 7.21 (m, 5 H, aromatic H), 4.68 (dd, 1 H, $J_{H\text{-}1,H\text{-}2}$ = 8.0 Hz, $J_{H\text{-}2,H\text{-}3}$ = 9.7 Hz, H-2), 4.08 (d, 1 H, H-1), 3.84 (dd, 1 H, $J_{H\text{-}5,H\text{-}6a}$ = 2.3 Hz, $J_{H\text{-}6a,H\text{-}6b}$ = – 12.0 Hz, H-6a), 3.81 (ddd, 1 H, $J_{2\text{-}CO\underline{CH}CH3Ph,\ 2\text{-}COCH\underline{CH3}Ph}$ = 7.1 Hz, 2-COC*H*CH$_3$Ph), 3.66 (dd, 1 H, $J_{H\text{-}5,H\text{-}6b}$ = 5.7 Hz, H-6b), 3.49 (dd, 1 H, $J_{H\text{-}3,H\text{-}4}$ = 9.0 Hz, H-3), 3.34 (dd, 1 H, $J_{H\text{-}4,H\text{-}5}$ = 9.8 Hz, H-4), 3.23 (ddd, 1 H, H-5), 3.16 (s, 3 H, 1-OC*H$_3$*), 1.45 (d, 3 H, 2-COCHC*H$_3$*Ph) ppm. ^{13}C NMR (125.78 MHz, CDCl$_3$, 25 °C): δ = 175.3 (2-*C*OCHCH$_3$Ph), 142.4, 129.5, 128.7, 128.0 (aromatic C), 103.5 (C-1), 78.0 (C-5), 75.9 (C-3), 75.4 (C-2), 71.7 (C-4), 62.6 (C-6), 57.3 (1-O*C*H$_3$), 46.7 (2-CO*C*HCH$_3$Ph), 19.1 (2-COCHC*H$_3$*Ph) ppm.

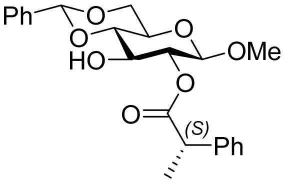

Methyl 2-*O*-(*S*)-2-phenyl-propanoyl-4,6-*O*-benzylidene-β-D-glucopyranoside (67): Synthesized from **62** (100 mg) according to the standard reaction procedure B. The crude product was purified by column chromatography (toluene:EtOAc 7:1) to obtain **67** as a white solid. Yield: 52 mg (35%), R$_f$ = 0.28. ^{1}H NMR (500.20 MHz, CDCl$_3$, 25 °C): δ = 7.47 – 7.22 (m, 10 H, aromatic H), 5.52 (s, 1 H, 4,6-OC*H*Ph), 4.90 (dd, 1 H, $J_{H\text{-}1,H\text{-}2}$ = 7.9 Hz, $J_{H\text{-}2,H\text{-}3}$ = 9.1 Hz, H-2), 4.40 (d, 1 H, H-1), 4.34 (dd, 1 H, $J_{H\text{-}5,H\text{-}6a}$ = 5.0 Hz, $J_{H\text{-}6a,H\text{-}6b}$ = – 10.6 Hz, H-6a), 3.80 (ddd, 1 H, $J_{2\text{-}CO\underline{CH}CH3Ph,\ 2\text{-}COCH\underline{CH3}Ph}$ = 7.1 Hz, 2-COC*H*CH$_3$Ph), 3.78 (dd, 1 H, $J_{H\text{-}5,H\text{-}6b}$ = 10.1 Hz, H-6b), 3.74 (ddd, 1 H, $J_{H\text{-}3,H\text{-}4}$ = 9.4 Hz, $J_{H\text{-}3,3\text{-}OH}$ = 3.5 Hz, H-3), 3.56 (dd, 1 H, $J_{H\text{-}4,H\text{-}5}$ = 9.4 Hz, H-4), 3.47 (s, 3 H, 1-OC*H$_3$*), 3.40 (ddd, 1 H, H-5), 2.13 (d, 1 H, 3-OH), 1.53 (d, 3 H, 2-COCHC*H$_3$*Ph) ppm. ^{13}C NMR (125.78 MHz, CDCl$_3$, 25 °C): δ = 173.8 (2-*C*OCHCH$_3$Ph), 140.7 – 126.4 (aromatic C), 102.3 (C-1), 102.0 (4,6-*C*HPh), 80.8 (C-4), 74.6 (C-2), 72.5 (C-3), 68.7 (C-6), 66.3 (C-5), 57.3 (1-O*C*H$_3$), 45.7 (2-CO*C*HCH$_3$Ph), 18.6 (2-COCHC*H$_3$*Ph) ppm.

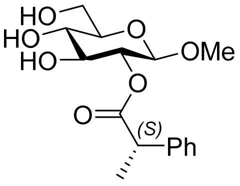

Methyl 2-*O*-(*S*)-2-phenyl-propanoyl-β-D-glucopyranoside (5): Synthesized from **67** (20 mg) according to the standard reaction procedure C to obtain **5** as a white solid. Yield: 13 mg (83%). ^{1}H NMR (500.20 MHz, CDCl$_3$, 25 °C): δ = 7.35 – 7.21 (m, 5 H, aromatic H), 4.70 (dd, 1 H, $J_{H\text{-}1,H\text{-}2}$ = 8.0 Hz, $J_{H\text{-}2,H\text{-}3}$ = 9.7 Hz, H-2), 4.34 (d, 1 H, H-1), 3.87 (dd, 1 H, $J_{H\text{-}5,H\text{-}6a}$ = 2.3 Hz, $J_{H\text{-}6a,H\text{-}6b}$ = – 12.0 Hz, H-6a), 3.80 (ddd, 1 H, $J_{2\text{-}CO\underline{CH}CH3Ph,\ 2\text{-}COCH\underline{CH3}Ph}$ = 7.2 Hz, 2-COC*H*CH$_3$Ph), 3.68 (dd, 1 H, $J_{H\text{-}5,H\text{-}6b}$ = 5.7 Hz, H-6b), 3.46 (dd, 1 H, $J_{H\text{-}3,H\text{-}4}$ = 8.9 Hz, H-3), 3.42 (s, 3 H, 1-OC*H$_3$*), 3.33 (dd, 1 H, $J_{H\text{-}4,H\text{-}5}$ = 9.8 Hz, H-4), 3.28 (ddd, 1 H, H-5), 1.50 (d, 3 H, 2-COCHC*H$_3$*Ph) ppm. ^{13}C NMR (125.78 MHz, CDCl$_3$, 25 °C): δ = 175.3 (2-*C*OCHCH$_3$Ph), 142.0, 129.5, 128.8, 128.0 (aromatic C), 103.3 (C-1), 78.0 (C-5), 76.0 (C-3), 75.4 (C-2), 71.9 (C-4), 62.6 (C-6), 57.1 (1-O*C*H$_3$), 47.0 (2-CO*C*HCH$_3$Ph), 19.4 (2-COCHC*H$_3$*Ph) ppm.

Galactopyranoside derivatives

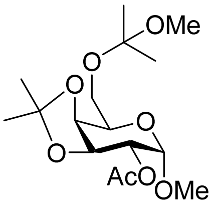

Methyl 2-*O*-acetyl-4,3-*O*-isopropylidene-6-(2-methoxyisopropyl)-α-D-galactopyranoside (69): Synthesized from **68** (50 mg) according to the standard procedure for acylation. The crude product was purified by column chromatography (toluene:EtOAc 4:1 with 0.1% Et$_3$N) to provide **69** as a clear oil. Yield: 22 mg (39%), R$_f$ = 0.23. Completely pure product could not be obtained and the product was used as such in the next step.

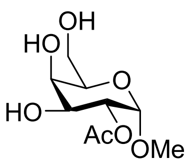

Methyl 2-*O*-acetyl-α-D-galactopyranoside (11): Synthesized from **69** (12 mg) according to the standard reaction procedure D to provide **11** as a white solid. Yield: 5 mg (69%). ^{1}H NMR (500.20 MHz, MeOD, 25 °C): δ = 4.97 (dd, 1 H, $J_{H\text{-}1,H\text{-}2}$ = 3.9 Hz, $J_{H\text{-}2,H\text{-}3}$ = 10.1 Hz, H-2), 4.88 (d, 1 H, H-1), 3.93 (dd, 1 H, $J_{H\text{-}3,H\text{-}4}$ = 3.4 Hz, $J_{H\text{-}4,H\text{-}5}$ = 1.0 Hz, H-4), 3.92 (dd, 1 H, H-3), 3.79 (ddd, 1 H, $J_{H\text{-}5,H\text{-}6a}$ = 7.1 Hz, $J_{H\text{-}5,H\text{-}6b}$ = 5.0 Hz, H-5), 3.74 (dd, 1 H, $J_{H\text{-}6a,H\text{-}6b}$ = – 11.4 Hz, H-6a), 3.70 (dd, 1 H, H-6b), 3.37 (s, 3 H, 1-OCH_3), 2.08 (s, 3 H, 2-OCOCH_3) ppm. ^{13}C NMR (125.78 MHz, MeOD, 25 °C): δ = 172.7 (2-*C*OCH$_3$), 98.6 (C-1), 72.9 (C-2), 72.2 (C-5), 71.1 (C-4), 68.9 (C-3), 62.7 (C-6), 55.5 (1-O*C*H$_3$), 20.8 (2-CO*C*H$_3$) ppm.

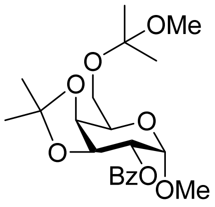

Methyl 2-*O*-benzoyl-4,3-*O*-isopropylidene-6-(2-methoxyisopropyl)-α-D-galactopyranoside (70): Synthesized from **68** (50 mg) according to the standard procedure for acylation. The crude product was purified by column chromatography (hexane:EtOAc 3:1 with 0.1% Et_3N) to provide **70** as a clear oil. Yield: 57 mg (31%), R_f = 0.35. ^{1}H NMR (500.20 MHz, CDCl$_3$, 25 °C): δ = 8.12 – 8.08 (m, 2 H, aromatic H), 7.59– 7.54 (m, 1 H, aromatic H), 7.46– 7.41 (m, 2 H, aromatic H), 5.14 (dd, 1 H, $J_{H\text{-}1,H\text{-}2}$ = 3.6 Hz, $J_{H\text{-}2,H\text{-}3}$ = 8.2 Hz, H-2), 4.98 (d, 1 H, H-1), 4.50 (dd, 1 H, $J_{H\text{-}3,H\text{-}4}$ = 5.4 Hz, H-3), 4.30 (dd, 1 H, $J_{H\text{-}4,H\text{-}5}$ = 2.5 Hz, H-4), 4.13 (ddd, 1 H, $J_{H\text{-}5,H\text{-}6a}$ = 5.6 Hz, $J_{H\text{-}5,H\text{-}6b}$ = 6.9 Hz, H-5), 3.76 (dd, 1 H, $J_{H\text{-}6a,H\text{-}6b}$ = – 9.9 Hz, H-6a), 3.69 (dd, 1 H, H-6b), 3.39 (s, 3 H, 1-OCH_3), 3.25 (s, 3 H, 6-C(CH$_3$)$_2$OCH_3), 1.55 (s, 3 H, 3,4-C(CH_3)$_2$), 1.39 (s, 3 H, 6-C(CH_3)$_2$OCH$_3$), 1.38 (s, 3 H, 6-C(CH_3)$_2$OCH$_3$), 1.35 (s, 3 H, 3,4-C(CH_3)$_2$) ppm. ^{13}C NMR (125.78 MHz, CDCl$_3$, 25 °C): δ = 166.3 (2-*C*OCH$_3$), 133.3, 130.1, 129.9, 128.5 (aromatic C), 109.9 (3,4-*C*(CH$_3$)$_2$), 100.3 (6-*C*(CH$_3$)$_2$OCH$_3$), 97.4 (C-1), 74.0 (C-4), 73.8 (C-3), 72.8 (C-2), 66.9 (C-5), 60.5 (C-6), 55.6 (1-O*C*H$_3$), 48.7 (6-C(CH$_3$)$_2$OCH_3), 28.2 (6-C(*C*H$_3$)$_2$OCH$_3$), 26.5 (6-C(*C*H$_3$)$_2$OCH$_3$), 24.63 (3,4-C(*C*H$_3$)$_2$), 24.55 (3,4-C(*C*H$_3$)$_2$) ppm.

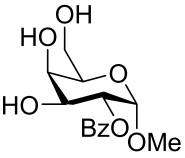

Methyl 2-*O*-benzoyl-α-D-galactopyranoside (12): Synthesized from **70** (10 mg) according to the standard reaction procedure D to provide **12** as a white solid. Yield: 4 mg (55%). ^{1}H NMR (500.20 MHz, MeOD, 25 °C): δ = 8.10– 8.05 (m, 2 H, aromatic H), 7.64– 7.58 (m, 1 H, aromatic H), 7.51– 7.46 (m, 2 H, aromatic H), 5.22 (dd, 1 H, $J_{H\text{-}1,H\text{-}2}$ = 3.8 Hz, $J_{H\text{-}2,H\text{-}3}$ = 10.4 Hz, H-2), 5.03 (d, 1 H, H-1), 4.10 (dd, 1 H, $J_{H\text{-}3,H\text{-}4}$ = 3.4 Hz, H-3), 3.99 (dd, 1 H, $J_{H\text{-}4,H\text{-}5}$ = 1.2 Hz, H-4), 3.86 (ddd, 1 H, $J_{H\text{-}5,H\text{-}6a}$ = 7.0 Hz, $J_{H\text{-}5,H\text{-}6b}$ = 5.1 Hz, H-5), 3.78 (dd, 1 H, $J_{H\text{-}6a,H\text{-}6b}$ = – 11.4 Hz, H-6a), 3.74 (dd, 1 H, H-6b), 3.38 (s, 3 H, 1-OCH_3) ppm. ^{13}C NMR (125.78 MHz, MeOD, 25 °C): δ = 168.0 (2-*C*OPh), 134.3, 131.4, 130.8, 129.5 (aromatic C), 98.8 (C-1), 73.5 (C-5), 72.3 (C-2), 71.3 (C-3), 69.0 (C-4), 62.7 (C-6), 55.6 (1-O*C*H$_3$) ppm.

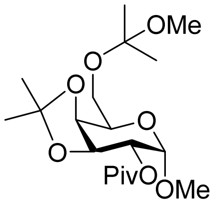

Methyl 4,3-*O*-isopropylidene-6-(2-methoxyisopropyl)-2-*O*-pivaloyl-α-D-galactopyranoside (71): Synthesized from **68** (50 mg) according to the standard procedure for acylation. The crude product was purified by column chromatography (hexane:EtOAc 3:1 with 0.1% Et_3N) to provide **71** as a clear oil. Yield: 57 mg (31%), R_f = 0.35. ^{1}H NMR (500.20 MHz, CDCl$_3$, 25 °C): δ = 4.86 (dd, 1 H, $J_{H\text{-}1,H\text{-}2}$ = 3.7 Hz, $J_{H\text{-}2,H\text{-}3}$ = 8.0 Hz, H-2), 4.82 (d, 1 H, H-1), 4.31 (dd, 1 H, $J_{H\text{-}3,H\text{-}4}$ = 5.4 Hz, H-3), 4.22 (dd, 1 H, $J_{H\text{-}4,H\text{-}5}$ = 2.4 Hz, H-4), 4.07 (ddd, 1 H, $J_{H\text{-}5,H\text{-}6a}$ = 5.6 Hz, $J_{H\text{-}5,H\text{-}6b}$ = 6.9 Hz, H-5), 3.72 (dd, 1 H, $J_{H\text{-}6a,H\text{-}6b}$ = – 9.9 Hz, H-6a), 3.65 (dd, 1 H, H-6b), 3.37 (s, 3 H, 1-OCH_3), 3.22 (s, 3 H, 6-C(CH$_3$)$_2$OCH_3), 1.50 (s, 3 H, 3,4-C(CH_3)$_2$), 1.37 (s, 3 H, 6-C(CH_3)$_2$OCH$_3$), 1.36 (s, 3 H, 6-C(CH_3)$_2$OCH$_3$), 1.33 (s, 3 H, 3,4-C(CH_3)$_2$), 1.23 (s, 9 H, 2-OCOC(CH_3)$_3$) ppm. ^{13}C NMR (125.78 MHz, CDCl$_3$, 25 °C): δ = 178.3 (2-*C*OC(CH$_3$)$_3$), 109.7 (3,4-*C*(CH$_3$)$_2$), 100.3 (6-*C*(CH$_3$)$_2$OCH$_3$), 97.2 (C-1), 73.8 (C-4), 73.7 (C-3), 71.9 (C-2), 66.9 (C-5), 60.5 (C-6), 55.6

(1-OCH_3), 48.7 (6-C(CH_3)$_2$OCH_3), 39.0 (2-CO*C*(CH_3)$_3$), 28.1 (3,4-C(*C*H_3)$_2$), 27.2 (2-COC(*CH$_3$)$_3$*), 26.5 (3,4-C(*C*H_3)$_2$), 24.6 (6-C(*C*H_3)$_2$OCH_3), 24.5 (6-C(*C*H_3)$_2$OCH_3) ppm.

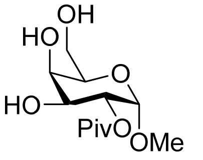

Methyl 2-*O*-pivaloyl-α-D-galactopyranoside (13): Synthesized from **71** (10 mg) according to the standard reaction procedure D to provide **13** as a white solid. Yield: 4.5 mg (63%). ^{1}H NMR (500.20 MHz, MeOD, 25 °C): δ = 4.92 (dd, 1 H, $J_{H-1,H-2}$ = 3.8 Hz, $J_{H-2,H-3}$ = 10.4 Hz, H-2), 4.86 (d, 1 H, H-1), 3.94 (dd, 1 H, $J_{H-3,H-4}$ = 3.5 Hz, H-3), 3.92 (dd, 1 H, $J_{H-4,H-5}$ = 1.2 Hz, H-4), 3.80 (ddd, 1 H, $J_{H-5,H-6a}$ = 7.0Hz, $J_{H-5,H-6b}$ = 5.1 Hz, H-5), 3.74 (dd, 1 H, $J_{H-6a,H-6b}$ = –11.4 Hz, H-6a), 3.70 (dd, 1 H, H-6b), 3.36 (s, 3 H, 1-O*CH$_3$*), 1.22 (s, 9 H, 2-OCOC(C*H$_3$)$_3$*) ppm. ^{13}C NMR (125.78 MHz, MeOD, 25 °C): δ = 180.0 (2-*C*OC(CH_3)$_3$), 98.6 (C-1), 72.7 (C-2), 72.2 (C-5), 71.3 (C-4), 69.0 (C-3), 62.7 (C-6), 55.7 (1-OCH_3), 39.9 (2-CO*C*(CH_3)$_3$), 27.5 (2-COC(*CH$_3$)$_3$*) ppm.

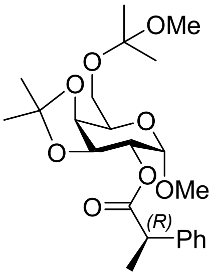

Methyl 4,3-*O*-isopropylidene-6-(2-methoxyisopropyl)-2-*O*-(*R*)-2-phenyl-propanoyl-α-D-galactopyranoside (72): Synthesized from **68** (50 mg) according to the standard reaction procedure B. The crude product was purified by column chromatography (hexane:EtOAc 3:1 + 0.1% Et_3N) to give **72** as a clear oil. Yield: 57 mg (80%), R$_f$ = 0.35. ^{1}H NMR (500.20 MHz, $CDCl_3$, 25 °C): δ = 7.33 – 7.22 (5 H, aromatic H), 4.88 (dd, 1 H, $J_{H-1,H-2}$ = 3.6 Hz, $J_{H-2,H-3}$ = 8.2 Hz, H-2), 4.64 (d, 1 H, H-1), 4.30 (dd, 1 H, $J_{H-3,H-4}$ = 5.4 Hz, H-3), 4.20 (dd, 1 H, $J_{H-4,H-5}$ = 2.4 Hz, H-4), 4.01 (ddd, 1 H, $J_{H-5,H-6a}$ = 5.7 Hz, $J_{H-5,H-6b}$ = 6.9 Hz, H-5), 3.82 (ddd, 1 H, $J_{2\text{-CO}\underline{CH}\text{CH3Ph, 2-COCH}\underline{CH3}\text{Ph}}$ = 7.1 Hz, 2-CO*CH*CH_3Ph), 3.69 (dd, 1 H, $J_{H-6a,H-6b}$ = –9.9 Hz, H-6a), 3.62 (dd, 1 H, H-6b), 3.21 (s, 3 H, 6-C(CH_3)$_2$O*CH$_3$*), 3.11 (s, 3 H, 1-O*CH$_3$*), 1.54 (s, 3 H, 3,4-C(C*H$_3$*)$_2$), 1.50 (d, 3 H, 2-COCH*CH$_3$*Ph), 1.353 (s, 3 H, 6-C(*CH$_3$)$_2$*OCH_3), 1.347 (s, 3 H, 6-C(*CH$_3$)$_2$*OCH_3), 1.33 (s, 3 H, 3,4-C(C*H$_3$*)$_2$) ppm. ^{13}C NMR (125.78 MHz, $CDCl_3$, 25 °C): δ = 174.4 (2-*C*OCHCH_3Ph), 141.6, 128.7, 127.7, 127.2 (aromatic C), 109.8 (3,4-*C*(CH_3)$_2$), 100.3 (6-*C*(CH_3)$_2$OCH_3), 97.0 (C-1), 73.8 (C-4), 73.5 (C-3), 72.5 (C-2), 66.8 (C-5), 60.4 (C-6), 56.5 (1-OCH_3), 48.7 (6-C(CH_3)$_2$O*CH$_3$*), 45.3 (2-CO*C*HCH_3Ph), 28.2 (3,4-C(*C*H_3)$_2$), 26.5 (3,4-C(*C*H_3)$_2$), 24.6 (6-C(*C*H_3)$_2$OCH_3), 24.5 (6-C(*C*H_3)$_2$OCH_3), 18.4 (2-COCH*C*H_3Ph) ppm.

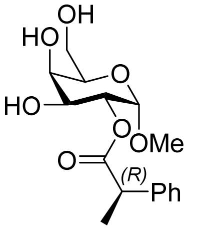

Methyl 2-*O*-(*R*)-2-phenyl-propanoyl-α-D-galactopyranoside (14): Synthesized from **72** (10 mg) according to the standard reaction procedure D to provide **14** as a white solid. Yield: 4 mg (54%). ^{1}H NMR (500.20 MHz, MeOD, 25 °C): δ = 7.36 – 7.21 (5 H, aromatic H), 4.97 (dd, 1 H, $J_{H-1,H-2}$ = 3.8 Hz, $J_{H-2,H-3}$ = 10.1 Hz, H-2), 4.70 (d, 1 H, H-1), 3.91 (dd, 1 H, $J_{H-3,H-4}$ = 3.4 Hz, $J_{H-4,H-5}$ = 1.1 Hz, H-4), 3.90 (dd, 1 H, H-3), 3.83 (ddd, 1 H, $J_{2\text{-CO}\underline{CH}\text{CH3Ph, 2-COCH}\underline{CH3}\text{Ph}}$ = 7.1 Hz, 2-CO*CH*CH_3Ph), 3.74 (ddd, 1 H, $J_{H-5,H-6a}$ = 7.1 Hz, $J_{H-5,H-6b}$ = 5.2 Hz, H-5), 3.70 (dd, 1 H, $J_{H-6a,H-6b}$ = – 11.5 Hz, H-6a), 3.67 (dd, 1 H, H-6b), 3.11 (s, 3 H, 1-O*CH$_3$*), 1.46 (s, 3 H, 2-COCH*CH$_3$*Ph) ppm. ^{13}C NMR (125.78 MHz, MeOD, 25 °C): δ = 176.2 (2-*C*OCHCH_3Ph), 142.1, 129.6, 128.6, 128.1 (aromatic C), 98.5 (C-1), 73.0 (C-2), 72.2 (C-5), 71.1 (C-4), 68.8 (C-3), 62.6 (C-6), 55.6 (1-O*C*H_3), 46.4 (2-CO*CH*CH_3Ph), 18.8 (2-COCH*C*H_3Ph) ppm.

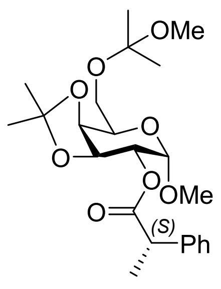

Methyl 4,3-*O*-isopropylidene-6-(2-methoxyisopropyl)-2-*O*-(*S*)-2-phenyl-propanoyl-α-D-galactopyranoside (73): Synthesized from **68** (44 mg) according to the standard reaction procedure B. The crude product was purified by column chromatography (hexane:EtOAc 3:1 + 0.1% Et_3N) to give **73** as a clear oil. Yield: 54 mg (84%), R_f = 0.33. ^1H NMR (500.20 MHz, $CDCl_3$, 25 °C): δ = 7.34 – 7.22 (5 H, aromatic H), 4.91 (dd, 1 H, $J_{H-1,H-2}$ = 3.6 Hz, $J_{H-2,H-3}$ = 7.9 Hz, H-2), 4.83 (d, 1 H, H-1), 4.27 (dd, 1 H, $J_{H-3,H-4}$ = 5.5 Hz, H-3), 4.18 (dd, 1 H, $J_{H-4,H-5}$ = 2.4 Hz, H-4), 4.04 (ddd, 1 H, $J_{H-5,H-6a}$ = 5.6 Hz, $J_{H-5,H-6b}$ = 6.9 Hz, H-5), 3.81 (ddd, 1 H, $J_{2-CO\underline{CH}CH3Ph, 2-COCH\underline{CH3}Ph}$ = 7.2 Hz, 2-CO*CH*CH_3Ph), 3.70 (dd, 1 H, $J_{H-6a,H-6b}$ = – 9.9 Hz, H-6a), 3.64 (dd, 1 H, H-6b), 3.36 (s, 3 H, 1-O*CH*$_3$), 3.22 (s, 3 H, 6-$C(CH_3)_2$O*CH*$_3$), 1.53 (d, 3 H, 2-COCH*CH*$_3$Ph), 1.46 (s, 3 H, 3,4-C(*CH*$_3$)$_2$), 1.37 (s, 3 H, 6-C(*CH*$_3$)$_2$$OCH_3$), 1.36 (s, 3 H, 6-C(*CH*$_3$)$_2$$OCH_3$), 1.29 (s, 3 H, 3,4-C(*CH*$_3$)$_2$) ppm. ^{13}C NMR (125.78 MHz, $CDCl_3$, 25 °C): δ = 174.3 (2-*C*OCHCH_3Ph), 140.4, 128.6, 127.7, 127.2 (aromatic C), 109.8 (3,4-*C*(CH_3)$_2$), 100.3 (6-*C*(CH_3)$_2$$OCH_3$), 97.1 (C-1), 73.8 (C-4), 73.5 (C-3), 72.3 (C-2), 67.0 (C-5), 60.4 (C-6), 55.5 (1-O*C*H_3), 48.7 (6-$C(CH_3)_2$O*CH*$_3$), 45.7 (2-CO*C*HCH_3Ph), 28.1 (3,4-C(*C*H_3)$_2$), 26.5 (3,4-C(*C*H_3)$_2$), 24.6 (6-C(*C*H_3)$_2$$OCH_3$), 24.5 (6-C(*C*$H_3$)$_2$$OCH_3$), 18.9 (2-COCH*C*$H_3$Ph) ppm.

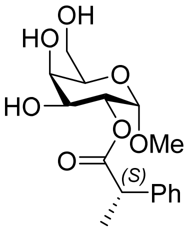

Methyl 2-*O*-(*S*)-2-phenyl-propanoyl-α-D-galactopyranoside (15): Synthesized from **73** (10 mg) according to the standard reaction procedure D to provide **15** as a white solid. Yield: 4.1 mg (55%).

^1H NMR (500.20 MHz, MeOD, 25 °C): δ = 7.37 – 7.21 (5 H, aromatic H), 4.96 (dd, 1 H, $J_{H-1,H-2}$ = 3.8 Hz, $J_{H-2,H-3}$ = 10.4 Hz, H-2), 4.87 (d, 1 H, H-1), 3.92 (dd, 1 H, $J_{H-3,H-4}$ = 3.5 Hz,H-3), 3.90 (dd, 1 H, $J_{H-4,H-5}$ = 1.2 Hz, H-4), 3.81 (ddd, 1 H, $J_{2-CO\underline{CH}CH3Ph, 2-COCH\underline{CH3}Ph}$ = 7.2 Hz, 2-CO*CH*CH_3Ph), 3.79 (ddd, 1 H, $J_{H-5,H-6a}$ = 7.1 Hz, $J_{H-5,H-6b}$ = 5.1 Hz, H-5), 3.73 (dd, 1 H, $J_{H-6a,H-6b}$ = – 11.4 Hz, H-6a), 3.69 (dd, 1 H, H-6b), 3.34 (s, 3 H, 1-O*CH*$_3$), 1.50 (s, 3 H, 2-COCH*CH*$_3$Ph) ppm. ^{13}C NMR (125.78 MHz, MeOD, 25 °C): δ = 176.1 (2-*C*OCHCH_3Ph), 141.9, 129.5, 128.7, 128.1 (aromatic C), 98.5 (C-1), 73.1 (C-2), 72.2 (C-5), 71.3 (C-4), 68.8 (C-3), 62.7 (C-6), 55.6 (1-O*C*H_3), 46.9 (2-CO*CH*CH_3Ph), 19.3 (s, 3 H, 2-COCH*C*H_3Ph) ppm.

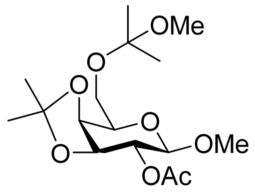

Methyl 2-*O*-acetyl-4,3-*O*-isopropylidene-6-(2-methoxyisopropyl)-β-D-galactopyranoside (75): Synthesized from **74** (89 mg) according to the standard procedure for acylation. The crude product was purified by column chromatography (hexane:EtOAc 1:1 with 0.1% Et_3N) to provide **75** as a white solid. Yield: 74 mg (73%), R_f = 0.35. ^1H NMR (500.20 MHz, $CDCl_3$, 25 °C): δ = 4.97 (dd, 1 H, $J_{H-1,H-2}$ = 8.2 Hz, $J_{H-2,H-3}$ = 7.5 Hz, H-2), 4.26 (d, 1 H, H-1), 4.19 (dd, 1 H, $J_{H-3,H-4}$ = 5.4 Hz, $J_{H-4,H-5}$ = 2.1 Hz, H-4), 4.15 (dd, 1 H, H-3), 3.87 (ddd, 1 H, $J_{H-5,H-6a}$ = 6.6 Hz, $J_{H-5,H-6b}$ = 5.7 Hz, H-5), 3.73 (dd, 1 H, $J_{H-6a,H-6b}$ = – 9.9 Hz, H-6a), 3.71 (dd, 1 H, H-6b), 3.47 (s, 3 H, 1-O*CH*$_3$), 3.24 (s, 3 H, 6-$C(CH_3)_2$O*CH*$_3$), 2.10 (s, 3 H, 2-OCO*CH*$_3$), 1.56 (s, 3 H, 3,4-C(*CH*$_3$)$_2$), 1.37 (s, 3 H, 6-C(*CH*$_3$)$_2$$OCH_3$), 1.36 (s, 3 H, 6-C(*CH*$_3$)$_2$$OCH_3$), 1.33 (s, 3 H, 3,4-C(*CH*$_3$)$_2$) ppm. ^{13}C NMR (125.78 MHz, $CDCl_3$, 25 °C): δ = 169.9 (2-*C*OCH_3), 110.6 (3,4-*C*(CH_3)$_2$), 101.4 (C-1), 100.3 (6-*C*(CH_3)$_2$$OCH_3$), 77.4 (C-3), 74.0 (C-4), 73.1 (C-2), 72.4 (C-5), 60.5 (C-6), 56.6 (1-O*C*H_3), 48.7 (6-$C(CH_3)_2$O*CH*$_3$), 27.8 (3,4-C(*C*H_3)$_2$), 26.4 (3,4-C(*C*H_3)$_2$), 24.6 (6-C(*C*H_3)$_2$$OCH_3$), 24.5 (6-C(*C*$H_3$)$_2$$OCH_3$), 21.2 (2-CO*C*$H_3$) ppm.

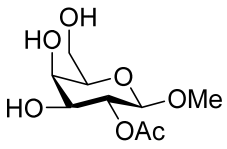

Methyl 2-*O*-acetyl-β-D-galactopyranoside (16): Synthesized from **75** (23 mg) according to the standard reaction procedure D to provide **16** as a white solid. Yield: 8 mg (52%). ^{1}H NMR (500.20 MHz, MeOD, 25 °C): δ = 5.00 (dd, 1 H, $J_{H\text{-}1,H\text{-}2}$ = 8.0 Hz, $J_{H\text{-}2,H\text{-}3}$ = 10.0 Hz, H-2), 4.31 (d, 1 H, H-1), 3.88 (dd, 1 H, $J_{H\text{-}3,H\text{-}4}$ = 3.4 Hz, $J_{H\text{-}4,H\text{-}5}$ = 1.1 Hz, H-4), 3.78 (dd, 1 H, $J_{H\text{-}5,H\text{-}6a}$ = 6.9 Hz, $J_{H\text{-}6a,H\text{-}6b}$ = – 11.4 Hz, H-6a), 3.74 (dd, 1 H, $J_{H\text{-}5,H\text{-}6b}$ = 5.5 Hz, H-6b), 3.64 (dd, 1 H, H-3), 3.54 (ddd, 1 H, H-5), 3.47 (s, 3 H, 1-OCH_3), 2.07 (s, 3 H, 2-OCOCH_3) ppm. ^{13}C NMR (125.78 MHz, MeOD, 25 °C): δ = 172.2 (2-*C*OCH$_3$), 103.5 (C-1), 76.8 (C-5), 73.8 (C-2), 73.2 (C-3), 70.4 (C-4), 62.3 (C-6), 56.9 (1-O*C*H$_3$), 21.0 (2-CO*C*H$_3$) ppm.

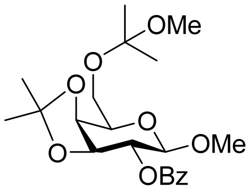

Methyl 2-*O*-benzoyl-4,3-*O*-isopropylidene-6-(2-methoxyisopropyl)-β-D-galactopyranoside (76): Synthesized from **74** (50 mg) according to the standard procedure for acylation. The crude product was purified by column chromatography (hexane:EtOAc 2:1 with 0.1% Et$_3$N) to provide **76** as a white solid. Yield: 21 mg (31%), R$_f$ = 0.30. ^{1}H NMR (500.20 MHz, CDCl$_3$, 25 °C): δ = 8.10– 8.02 (m, 2 H, aromatic H), 7.57– 7.53 (m, 1 H, aromatic H), 7.46– 7.41 (m, 2 H, aromatic H), 5.23 (dd, 1 H, $J_{H\text{-}1,H\text{-}2}$ = 8.0 Hz, $J_{H\text{-}2,H\text{-}3}$ = 7.3 Hz, H-2), 4.42 (d, 1 H, H-1), 4.33 (dd, 1 H, $J_{H\text{-}3,H\text{-}4}$ = 5.4 Hz, H-3), 4.26 (dd, 1 H, $J_{H\text{-}4,H\text{-}5}$ = 2.1 Hz, H-4), 3.94 (ddd, 1 H, $J_{H\text{-}5,H\text{-}6a}$ = 6.6 Hz, $J_{H\text{-}5,H\text{-}6b}$ = 5.8 Hz, H-5), 3.77 (dd, 1 H, $J_{H\text{-}6a,H\text{-}6b}$ = – 9.9 Hz, H-6a), 3.76 (dd, 1 H, H-6b), 3.47 (s, 3 H, 1-OCH_3), 3.26 (s, 3 H, 6-C(CH$_3$)$_2$OCH_3), 1.61 (s, 3 H, 3,4-C(CH_3)$_2$), 1.39 (s, 3 H, 6-C(CH_3)$_2$OCH$_3$), 1.38 (s, 3 H, 6-C(CH_3)$_2$OCH$_3$), 1.34 (s, 3 H, 3,4-C(CH_3)$_2$) ppm. ^{13}C NMR (125.78 MHz, CDCl$_3$, 25 °C): δ = 165.6 (2-*C*OCH$_3$), 133.2, 130.2, 130.0, 128.4 (aromatic C), 110.7 (3,4-*C*(CH$_3$)$_2$), 101.6 (C-1), 100.3 (6-*C*(CH$_3$)$_2$OCH$_3$), 77.2 (C-3), 74.0 (C-4), 73.7 (C-2), 72.5 (C-5), 60.5 (C-6), 56.7 (1-O*C*H$_3$), 48.7 (6-C(CH$_3$)$_2$OCH_3), 27.9 (3,4-C(*C*H$_3$)$_2$), 26.4 (3,4-C(*C*H$_3$)$_2$), 24.59 (6-C(*C*H$_3$)$_2$OCH$_3$), 24.56 (6-C(*C*H$_3$)$_2$OCH$_3$) ppm.

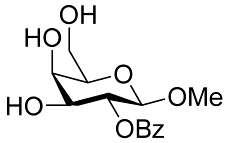

Methyl 2-*O*-benzoyl-β-D-galactopyranoside (17): Synthesized from **76** (10 mg) according to the standard reaction procedure D to provide **17** as a white solid. Yield: 3 mg (43%). ^{1}H NMR (500.20 MHz, MeOD, 25 °C): δ = 8.10– 8.02 (m, 2 H, aromatic H), 7.64– 7.58 (m, 1 H, aromatic H), 7.51– 7.46 (m, 2 H, aromatic H), 5.26 (dd, 1 H, $J_{H\text{-}1,H\text{-}2}$ = 8.0 Hz, $J_{H\text{-}2,H\text{-}3}$ = 10.0 Hz, H-2), 4.49 (d, 1 H, H-1), 3.93 (dd, 1 H, $J_{H\text{-}3,H\text{-}4}$ = 3.4 Hz, $J_{H\text{-}4,H\text{-}5}$ = 1.1 Hz, H-4), 3.83 (dd, 1 H, H-3), 3.82 (dd, 1 H, $J_{H\text{-}5,H\text{-}6a}$ = 6.8 Hz, $J_{H\text{-}6a,H\text{-}6b}$ = – 11.3 Hz, H-6a), 3.78 (dd, 1 H, $J_{H\text{-}5,H\text{-}6b}$ = 5.4 Hz, H-6b), 3.63 (ddd, 1 H, H-5), 3.47 (s, 3 H, 1-OCH_3) ppm. ^{13}C NMR (125.78 MHz, MeOD, 25 °C): δ = 167.6 (2-*C*OPh), 134.2, 131.7, 130.7, 129.5 (aromatic C), 103.7 (C-1), 76.9 (C-5), 74.4 (C-2), 73.3 (C-3), 70.6 (C-4), 62.4 (C-6), 57.0 (1-O*C*H$_3$) ppm.

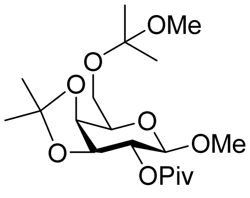

Methyl 4,3-*O*-isopropylidene-6-(2-methoxyisopropyl)-2-*O*-pivaloyl-β-D-galactopyranoside (77): Synthesized from **74** (50 mg) according to the standard procedure for acylation. The crude product was purified by column chromatography (hexane:EtOAc 3:1 with 0.1% Et$_3$N) to provide **77** as a clear oil. Yield: 13 mg (20%), R$_f$ = 0.39. ^{1}H NMR (500.20 MHz, CDCl$_3$, 25 °C): δ = 4.95 (dd, 1 H, $J_{H\text{-}1,H\text{-}2}$ = 8.0 Hz, $J_{H\text{-}2,H\text{-}3}$ = 7.2 Hz, H-2), 4.25 (d, 1 H, H-1), 4.19 (dd, 1 H, $J_{H\text{-}3,H\text{-}4}$ = 5.4 Hz, $J_{H\text{-}4,H\text{-}5}$ = 2.1 Hz, H-4), 4.15 (dd, 1 H, H-3), 3.86 (ddd, 1 H, $J_{H\text{-}5,H\text{-}6a}$ = 6.7 Hz, $J_{H\text{-}5,H\text{-}6b}$ = 5.7 Hz, H-5), 3.73 (dd, 1 H, $J_{H\text{-}6a,H\text{-}6b}$ = – 9.9 Hz, H-6a), 3.72 (dd, 1 H, H-6b), 3.46 (s, 3 H, 1-OCH_3), 3.24 (s, 3 H, 6-C(CH$_3$)$_2$OCH_3), 1.56 (s, 3 H, 3,4-C(CH_3)$_2$), 1.373 (s, 3 H, 6-C(CH_3)$_2$OCH$_3$), 1.366 (s, 3 H, 6-C(CH_3)$_2$OCH$_3$), 1.33 (s, 3 H, 3,4-C(CH_3)$_2$), 1.22 (s, 9 H, 2-OCOC(CH_3)$_3$) ppm. ^{13}C NMR (125.78 MHz, CDCl$_3$, 25 °C): δ = 177.3 (2-*C*OC(CH$_3$)$_3$), 110.5 (3,4-*C*(CH$_3$)$_2$), 101.8 (C-1), 100.3 (6-*C*(CH$_3$)$_2$OCH$_3$), 77.4 (C-3), 73.9 (C-4), 72.8 (C-2), 72.4 (C-5), 60.5 (C-6),

56.7 (1-OCH_3), 48.7 (6-C(CH_3)$_2$OCH_3), 38.9 (2-CO*C*(CH_3)$_3$), 27.8 (3,4-*C*(CH_3)$_2$), 27.2 (2-COC(CH_3)$_3$), 26.5 (3,4-C(CH_3)$_2$), 24.59 (6-C(CH_3)$_2$OCH_3), 24.56 (6-C(CH_3)$_2$OCH_3) ppm.

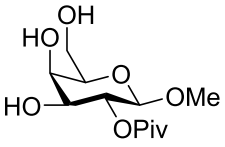

Methyl 2-*O*-pivaloyl-β-D-galactopyranoside (18): Synthesized from **77** (10 mg) according to the standard reaction procedure D to provide **18** as a white solid. Yield: 4.5 mg (63%). ^{1}H NMR (500.20 MHz, MeOD, 25 °C): δ = 4.98 (dd, 1 H, $J_{H\text{-}1,H\text{-}2}$ = 8.0 Hz, $J_{H\text{-}2,H\text{-}3}$ = 10.0 Hz, H-2), 4.32 (d, 1 H, H-1), 3.87 (dd, 1 H, $J_{H\text{-}3,H\text{-}4}$ = 3.4 Hz, $J_{H\text{-}4,H\text{-}5}$ = 1.1 Hz, H-4), 3.78 (dd, 1 H, $J_{H\text{-}5,H\text{-}6a}$ = 6.8 Hz, $J_{H\text{-}6a,H\text{-}6b}$ = – 11.3 Hz, H-6a), 3.74 (dd, 1 H, $J_{H\text{-}5,H\text{-}6b}$ = 5.4 Hz, H-6b), 3.66 (dd, 1 H, H-3), 3.55 (ddd, 1 H, H-5), 3.47 (s, 3 H, 1-OCH_3), 1.22 (s, 9 H, 2-OCOC(CH_3)$_3$) ppm. ^{13}C NMR (125.78 MHz, MeOD, 25 °C): δ = 179.5 (2-*C*OC(CH_3)$_3$), 103.9 (C-1), 76.8 (C-5), 73.5 (C-2), 73.2 (C-3), 70.6 (C-4), 62.4 (C-6), 57.1 (1-OCH_3), 39.9 (2-CO*C*(CH_3)$_3$), 27.5 (2-COC(CH_3)$_3$) ppm.

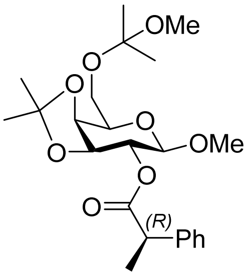

Methyl 4,3-*O*-isopropylidene-6-(2-methoxyisopropyl)-2-*O*-(*R*)-2-phenyl-propanoyl-β-D-galactopyranoside (78): Synthesized from **74** (50 mg) according to the standard reaction procedure B. The crude product was purified by column chromatography (hexane:EtOAc 3:1 + 0.1% Et_3N) to give **78** as a white solid. Yield: 54 mg (76%), R_f = 0.23. ^{1}H NMR (500.20 MHz, $CDCl_3$, 25 °C): δ = 7.33 – 7.21 (5 H, aromatic H), 4.97 (dd, 1 H, $J_{H\text{-}1,H\text{-}2}$ = 8.2 Hz, $J_{H\text{-}2,H\text{-}3}$ = 7.4 Hz, H-2), 4.15 (dd, 1 H, $J_{H\text{-}3,H\text{-}4}$ = 5.4 Hz, $J_{H\text{-}4,H\text{-}5}$ = 2.1 Hz, H-4), 4.12 (dd, 1 H, H-3), 3.98 (d, 1 H, H-1), 3.77 (ddd, 1 H, $J_{H\text{-}5,H\text{-}6}$ = 6.2 Hz, H-5), 3.75 (ddd, 1 H, $J_{2\text{-}CO\underline{CH}CH3Ph,\ 2\text{-}COCH\underline{CH3}Ph}$ = 7.1 Hz, 2-CO*C*HCH$_3$Ph), 3.69 (dd, 2 H, H-6), 3.21 (s, 3 H, 6-C(CH_3)$_2$OCH_3), 3.17 (s, 3 H, 1-OCH_3), 1.57 (s, 3 H, 3,4-C(CH_3)$_2$), 1.49 (d, 3 H, 2-COCHCH_3Ph), 1.34 (s, 6 H, 6-C(CH_3)$_2$OCH_3), 1.33 (s, 3 H, 3,4-C(CH_3)$_2$) ppm. ^{13}C NMR (125.78 MHz, $CDCl_3$, 25 °C): δ = 173.3 (2-*C*OCHCH$_3$Ph), 141.0, 128.6, 127.8, 127.0 (aromatic C), 110.6 (3,4-*C*(CH_3)$_2$), 101.8 (C-1), 100.3 (6-*C*(CH_3)$_2$OCH_3), 77.0 (C-3), 73.9 (C-4), 73.4 (C-2), 72.3 (C-5), 60.4 (C-6), 56.7 (1-OCH_3), 48.7 (6-C(CH_3)$_2$OCH_3), 45.7 (2-CO*C*HCH$_3$Ph), 27.9 (3,4-C(CH_3)$_2$), 26.5 (3,4-C(CH_3)$_2$), 24.6 (6-C(CH_3)$_2$OCH_3), 24.5 (6-C(CH_3)$_2$OCH_3), 18.8 (2-COCHCH_3Ph) ppm.

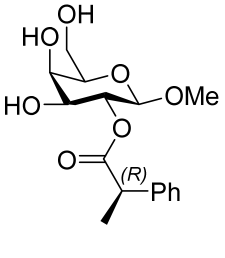

Methyl 2-*O*-(*R*)-2-phenyl-propanoyl-β-D-galactopyranoside (19): Synthesized from **78** (10 mg) according to the standard reaction procedure D to provide **19** as a white solid. Yield: 5.7 mg (77%). ^{1}H NMR (500.20 MHz, MeOD, 25 °C): δ = 7.34 – 7.20 (5 H, aromatic H), 5.00 (dd, 1 H, $J_{H\text{-}1,H\text{-}2}$ = 8.0 Hz, $J_{H\text{-}2,H\text{-}3}$ = 10.0 Hz, H-2), 4.04 (d, 1 H, H-1), 3.85 (dd, 1 H, $J_{H\text{-}3,H\text{-}4}$ = 3.4 Hz, $J_{H\text{-}4,H\text{-}5}$ = 1.0 Hz, H-4), 3.81 (ddd, 1 H, $J_{2\text{-}CO\underline{CH}CH3Ph,\ 2\text{-}COCH\underline{CH3}Ph}$ = 7.1 Hz, 2-CO*C*HCH$_3$Ph), 3.74 (dd, 1 H, $J_{H\text{-}5,H\text{-}6a}$ = 6.8 Hz, $J_{H\text{-}6a,H\text{-}6b}$ = – 11.4 Hz, H-6a), 3.71 (dd, 1 H, $J_{H\text{-}5,H\text{-}6b}$ = 5.4 Hz, H-6b), 3.62 (dd, 1 H, H-3), 3.47 (ddd, 1 H, H-5), 3.16 (s, 3 H, 1-OCH_3), 1.33 (s, 3 H, 2-COCHCH_3Ph) ppm. ^{13}C NMR (125.78 MHz, MeOD, 25 °C): δ = 175.5 (2-*C*OCHCH$_3$Ph), 142.4, 129.5, 128.7, 127.9 (aromatic C), 104.0 (C-1), 76.7 (C-5), 74.0 (C-2), 73.0 (C-3), 70.4 (C-4), 62.3 (C-6), 57.2 (1-OCH_3), 46.7 2-CO*C*HCH$_3$Ph), 19.1 (s, 3 H, 2-COCH*C*H$_3$Ph) ppm.

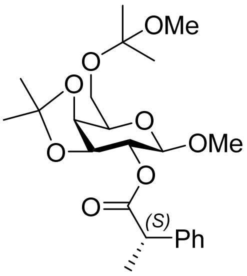

Methyl 4,3-*O*-isopropylidene-6-(2-methoxyisopropyl)-2-*O*-(*S*)-2-phenyl-propanoyl-β-D-galactopyranoside (79): Synthesized from **74** (50 mg) according to the standard reaction procedure B. The crude product was purified by column chromatography (hexane:EtOAc 3:1 + 0.1% Et_3N) to give **79** as a clear oil. Yield: 52 mg (73%), R_f = 0.27. ^{1}H NMR (500.20 MHz, $CDCl_3$, 25 °C): δ = 7.34 – 7.22 (5 H, aromatic H), 4.96 (dd, 1 H, $J_{H\text{-}1,H\text{-}2}$ = 7.9 Hz, $J_{H\text{-}2,H\text{-}3}$ = 7.1 Hz, H-2), 4.24 (d, 1 H, H-1), 4.11 (dd, 1 H, $J_{H\text{-}3,H\text{-}4}$ = 5.5 Hz, $J_{H\text{-}4,H\text{-}5}$ = 2.1 Hz, H-4), 4.04 (dd, 1 H, H-

3), 3.81 (ddd, 1 H, $J_{H\text{-}5,H\text{-}6a}$ = 6.7 Hz, $J_{H\text{-}5,H\text{-}6b}$ = 5.7 Hz, H-5), 3.77 (ddd, 1 H, $J_{2\text{-}CO\underline{CH}CH3Ph,\ 2\text{-}COCH\underline{CH3}Ph}$ = 7.2 Hz, 2-COC*H*CH$_3$Ph), 3.71 (dd, 1 H, $J_{H\text{-}5,H\text{-}6a}$ = 6.8 Hz, $J_{H\text{-}6a,H\text{-}6b}$ = – 9.9 Hz, H-6a), 3.69 (dd, 1 H, $J_{H\text{-}5,H\text{-}6b}$ = 5.4 Hz, H-6b), 3.40 (s, 3 H, 1-OC*H$_3$*), 3.22 (s, 3 H, 6-C(CH$_3$)$_2$OC*H$_3$*), 1.54 (s, 3 H, 3,4-C(C*H$_3$*)$_2$), 1.52 (d, 3 H, 2-COCHC*H$_3$*Ph), 1.36 (s, 3 H, 6-C(*CH$_3$)$_2$*OCH$_3$), 1.35 (s, 3 H, 6-C(*CH$_3$)$_2$*OCH$_3$), 1.29 (s, 3 H, 3,4-C(C*H$_3$*)$_2$) ppm. ^{13}C NMR (125.78 MHz, CDCl$_3$, 25 °C): δ = 173.4 (2-*C*OCHCH$_3$Ph), 140.7, 128.6, 127.8, 127.1 (aromatic C), 110.5 (3,4-*C*(CH$_3$)$_2$), 101.4 (C-1), 100.3 (6-*C*(CH$_3$)$_2$OCH$_3$), 76.8 (C-3), 73.9 (C-4), 73.4 (C-2), 72.4 (C-5), 60.5 (C-6), 56.6 (1-O*C*H$_3$), 48.7 (6-C(CH$_3$)$_2$OC*H$_3$*), 45.8 (2-CO*C*HCH$_3$Ph), 27.8 (3,4-C(*C*H$_3$)$_2$), 26.4 (3,4-C(*C*H$_3$)$_2$), 24.6 (6-C(*C*H$_3$)$_2$OCH$_3$), 24.5 (6-C(*C*H$_3$)$_2$OCH$_3$), 19.0 (2-COCH*C*H$_3$Ph) ppm.

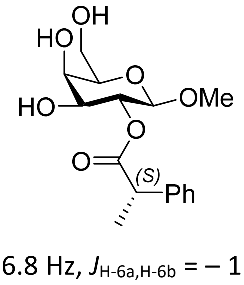

Methyl 2-*O*-(*R*)-2-phenyl-propanoyl-β-D-galactopyranoside (20): Synthesized from **79** (10 mg) according to the standard reaction procedure D to provide **20** as a white solid. Yield: 5.7 mg (77%). ^{1}H NMR (500.20 MHz, MeOD, 25 °C): δ = 7.35 – 7.20 (5 H, aromatic H), 5.01 (dd, 1 H, $J_{H\text{-}1,H\text{-}2}$ = 8.0 Hz, $J_{H\text{-}2,H\text{-}3}$ = 10.0 Hz, H-2), 4.30 (d, 1 H, H-1), 3.83 (dd, 1 H, $J_{H\text{-}3,H\text{-}4}$ = 3.4 Hz, $J_{H\text{-}4,H\text{-}5}$ = 1.1 Hz, H-4), 3.80 (ddd, 1 H, $J_{2\text{-}CO\underline{CH}CH3Ph,\ 2\text{-}COCH\underline{CH3}Ph}$ = 7.2 Hz, 2-COC*H*CH$_3$Ph), 3.76 (dd, 1 H, $J_{H\text{-}5,H\text{-}6a}$ = 6.8 Hz, $J_{H\text{-}6a,H\text{-}6b}$ = – 11.4 Hz, H-6a), 3.73 (dd, 1 H, $J_{H\text{-}5,H\text{-}6b}$ = 5.4 Hz, H-6b), 3.60 (dd, 1 H, H-3), 3.52 (ddd, 1 H, H-5), 3.42 (s, 3 H, 1-OC*H$_3$*), 1.50 (s, 3 H, 2-COCHC*H$_3$*Ph) ppm. ^{13}C NMR (125.78 MHz, MeOD, 25 °C): δ = 175.6 (2-*C*OCHCH$_3$Ph), 142.1, 129.5, 128.8, 128.0 (aromatic C), 103.7 (C-1), 76.7 (C-5), 74.0 (C-2), 73.1 (C-3), 70.6 (C-4), 62.4 (C-6), 57.0 (1-O*C*H$_3$), 47.1 (2-COC*H*CH$_3$Ph), 19.4 (s, 3 H, 2-COCH*C*H$_3$Ph) ppm.

Mannopyranoside derivatives

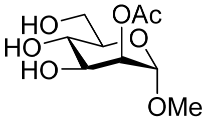

Methyl 2-*O*-benzoyl-α-D-mannopyranoside (21): See ref [3] for synthesis and characterization

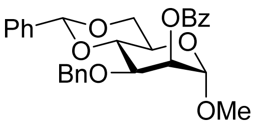

Methyl 3-*O*-benzyl-2-*O*-benzoyl-4,6-*O*-benzylidene-α-D-mannopyranoside (82): Synthesized from **80** (100 mg) according to standard reaction procedure A1. The crude product was purified by column chromatography (hexane:EtOAc 3:1) to provide **82** as a white solid. Yield: 40 mg (35%), R_f = 0.41. ^{1}H NMR (500.20 MHz, CDCl$_3$, 25 °C): δ = 8.14 – 7.19 (m, 15 H, aromatic H), 5.69 (s, 1 H, 4,6-OC*H*Ph), 5.60 (dd, 1 H, $J_{H\text{-}1,H\text{-}2}$ = 1.5 Hz, $J_{H\text{-}2,H\text{-}3}$ = 3.5 Hz, H-2), 4.83 (d, 1 H, H-1), 4.74 (d, 1 H, *J* = -12.4, 3-OC*H$_2$*Ph), 4.70 (d, 1 H, 3-OC*H$_2$*Ph), 4.32 (dd, 1 H, $J_{H\text{-}5,H\text{-}6a}$ = 5.1 Hz, $J_{H\text{-}6a,H\text{-}6b}$ = – 10.5 Hz, H-6a), 4.18 (dd, 1 H, $J_{H\text{-}3,H\text{-}4}$ = 9.9 Hz, $J_{H\text{-}4,H\text{-}5}$ = 9.3 Hz, H-4), 4.11 (dd, 1 H, H-3), 3.911 (dd, 1 H, $J_{H\text{-}5,H\text{-}6b}$ = 10.3 Hz, H-6b), 3.907 (ddd, 1 H, H-5), 3.41 (s, 3 H, 1-OC*H$_3$*) ppm. ^{13}C NMR (125.78 MHz, CDCl$_3$, 25 °C): δ = 165.9 (2-*C*OPh), 138.2 – 126.3 (aromatic C), 101.8 (4,6-*C*HPh), 100.0 (C-1), 78.8 (C-4), 74.1 (C-3), 72.1 (3-*C*H$_2$Ph), 70.3 (C-2), 69.1 (C-6), 63.9 (C-5), 55.3 (1-O*C*H$_3$) ppm.

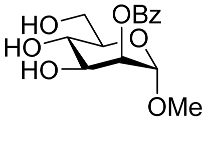

Methyl 2-*O*-benzoyl-α-D-mannopyranoside (22): Synthesized from **82** (20 mg) according to the standard reaction procedure C to provide **22** as a white solid. Yield: 10 mg (80%). ^{1}H NMR (500.20 MHz, MeOD, 25 °C): δ = 8.10– 7.45 (m, 5 H, aromatic H), 5.24 (dd, 1 H, $J_{H\text{-}1,H\text{-}2}$ = 1.7 Hz, $J_{H\text{-}2,H\text{-}3}$ = 3.5 Hz, H-2), 4.78 (d, 1 H, H-1), 3.98 (dd, 1 H, $J_{H\text{-}3,H\text{-}4}$ = 9.6 Hz, H-3), 3.89 (dd, 1 H, $J_{H\text{-}5,H\text{-}6a}$ = 2.3 Hz, $J_{H\text{-}6a,H\text{-}6b}$ = – 11.9 Hz, H-6a), 3.83 (dd, 1 H, $J_{H\text{-}4,H\text{-}5}$ = 9.9 Hz, H-4), 3.78 (dd, 1 H, $J_{H\text{-}5,H\text{-}6b}$ = 5.7 Hz, H-6b), 3.59 (ddd, 1 H, H-5), 3.43 (s, 3 H, 1-OC*H$_3$*) ppm. ^{13}C NMR (125.78 MHz, MeOD, 25 °C): δ = 167.5 (2-*C*OPh), 134.4 – 129.5 (aromatic C), 100.0 (C-1), 74.7 (C-5), 74.4 (C-2), 71.0 (C-3), 68.9 (C-4), 62.7 (C-6), 55.4 (1-O*C*H$_3$) ppm.

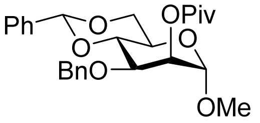

Methyl 3-*O*-benzyl-4,6-*O*-benzylidene-2-*O*-pivaloyl-α-D-mannopyranoside (83): Synthesized from **80** (40 mg) according to standard reaction procedure A1. The crude product was purified by column chromatography (hexane:EtOAc 4:1) to provide **83** as a white solid. Yield: 50 mg (61%), R_f = 0.42. ^{1}H NMR (500.20 MHz, $CDCl_3$, 25 °C): δ = 7.53 – 7.22 (m, 10 H, aromatic H), 5.64 (s, 1 H, 4,6-OC*H*Ph), 5.37 (dd, 1 H, $J_{H\text{-}1,H\text{-}2}$ = 1.7 Hz, $J_{H\text{-}2,H\text{-}3}$ = 3.2 Hz, H-2), 4.67 (d, 1 H, *J* = -12.2, 3-OCH_2Ph), 4.65 (d, 1 H, H-1), 4.64 (d, 1 H, 3-OCH_2Ph), 4.28 (dd, 1 H, $J_{H\text{-}5,H\text{-}6a}$ = 5.0 Hz, $J_{H\text{-}6a,H\text{-}6b}$ = – 10.5 Hz, H-6a), 4.01 (dd, 1 H, $J_{H\text{-}3,H\text{-}4}$ = 9.8 Hz, H-3), 3.99 (dd, 1 H, $J_{H\text{-}4,H\text{-}5}$ = 9.2 Hz, H-4), 3.842 (ddd, 1 H, $J_{H\text{-}5,H\text{-}6b}$ = 10.4 Hz, H-5), 3.837 (dd, 1 H, H-6b), 3.37 (s, 3 H, 1-OCH_3), 1.26 (s, 9 H, 2-COC(CH_3)$_3$) ppm. ^{13}C NMR (125.78 MHz, $CDCl_3$, 25 °C): δ = 177.8 (2-*C*OC(CH_3)$_3$), 138.3 – 126.3 (aromatic C), 101.8 (4,6-*C*HPh), 100.0 (C-1), 78.8 (C-4), 74.0 (C-3), 71.8 (3-*C*H_2Ph), 69.2 (C-2), 69.1 (C-6), 63.8 (C-5), 55.2 (1-O*C*H_3), 39.1 (2-CO*C*(CH_3)$_3$, 27.3 (2-COC(CH_3)$_3$ ppm.

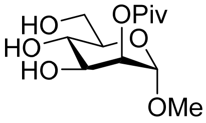

Methyl 2-*O*-pivaloyl-α-D-mannopyranoside (23): Synthesized from **83** (9 mg) according to the standard reaction procedure C to provide **23** as a white solid. Yield: 4.4 mg (80%). ^{1}H NMR (500.20 MHz, MeOD, 25 °C): δ = 4.95 (dd, 1 H, $J_{H\text{-}1,H\text{-}2}$ = 1.7 Hz, $J_{H\text{-}2,H\text{-}3}$ = 3.4 Hz, H-2), 4.59 (d, 1 H, H-1), 3.88 (dd, 1 H, $J_{H\text{-}3,H\text{-}4}$ = 9.5 Hz, H-3), 3.86 (dd, 1 H, $J_{H\text{-}5,H\text{-}6a}$ = 2.4 Hz, $J_{H\text{-}6a,H\text{-}6b}$ = – 11.9 Hz, H-6a), 3.68 (dd, 1 H, $J_{H\text{-}5,H\text{-}6b}$ = 6.1 Hz, H-6b), 3.63 (dd, 1 H, $J_{H\text{-}4,H\text{-}5}$ = 9.9 Hz, H-4), 3.52 (ddd, 1 H, H-5), 3.39 (s, 3 H, 1-OCH_3), 1.22 (s, 9 H, 2-COC(CH_3)$_3$) ppm. ^{13}C NMR (125.78 MHz, MeOD, 25 °C): δ = 179.6 (2-*C*OC(CH_3)$_3$, 99.7 (C-1), 74.7 (C-5), 73.6 (C-2), 70.8 (C-3), 69.0 (C-4), 62.9 (C-6), 55.3 (1-O*C*H_3), 40.1 (2-CO*C*(CH_3)$_3$, 27.5 (2-COC(CH_3)$_3$ ppm.

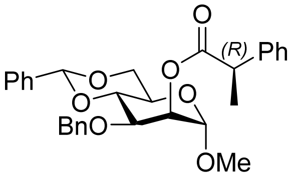

Methyl 3-*O*-benzyl-4,6-*O*-benzylidene-2-*O*-(*R*)-2-phenyl-propanoyl-α-D-mannopyranoside (84): Synthesized from **80** (50 mg) according to the standard reaction procedure B. The crude product was purified by column chromatography (hexane:EtOAc 3:1) to provide **84** as a white solid. Yield: 50 mg (79%), R_f = 0.36. ^{1}H NMR (500.20 MHz, $CDCl_3$, 25 °C): δ = 7.50 – 7.20 (m, 15 H, aromatic H), 5.52 (s, 1 H, 4,6-OC*H*Ph), 5.34 (dd, 1 H, $J_{H\text{-}1,H\text{-}2}$ = 1.7 Hz, $J_{H\text{-}2,H\text{-}3}$ = 3.4 Hz, H-2), 4.66 (d, 1 H, H-1), 4.58 (d, 1 H, *J* = -12.1, 3-OCH_2Ph), 4.56 (d, 1 H, 3-OCH_2Ph), 4.24 (dd, 1 H, $J_{H\text{-}5,H\text{-}6a}$ = 4.6 Hz, $J_{H\text{-}6a,H\text{-}6b}$ = – 10.1 Hz, H-6a), 3.94 (dd, 1 H, $J_{H\text{-}3,H\text{-}4}$ = 9.6 Hz, H-3), 3.84 (ddd, 1 H, $J_{2\text{-}CO\underline{CH}CH3Ph,\ 2\text{-}COCH\underline{CH3}Ph}$ = 7.2 Hz, 2-COC*H*CH_3Ph), 3.79 (ddd, 1 H, $J_{H\text{-}4,H\text{-}5}$ = 9.4 Hz, $J_{H\text{-}5,H\text{-}6b}$ = 10.3 Hz, H-5), 3.77 (dd, 1 H, H-4), 3.76 (dd, 1 H, H-6b), 3.36 (s, 3 H, 1-OCH_3), 1.55 (d, 3 H, 2-COCHCH_3Ph) ppm. ^{13}C NMR (125.78 MHz, $CDCl_3$, 25 °C): δ = 173.9 (2-*C*OCHCH_3Ph), 140.2 – 126.2 (aromatic C), 101.7 (4,6-*C*HPh), 99.9 (C-1), 78.6 (C-4), 74.0 (C-3), 72.0 (3-*C*H_2Ph), 70.0 (C-2), 69.0 (C-6), 63.8 (C-5), 55.2 (1-O*C*H_3), 45.6 (2-CO*C*HCH_3Ph), 18.5 (2-COCH*C*H_3Ph) ppm.

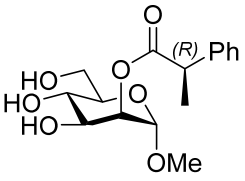

Methyl 2-*O*-(*R*)-2-phenyl-propanoyl-α-D-mannopyranoside (24): Synthesized from **84** (9 mg) according to the standard reaction procedure C to provide **24** as a white solid. Yield: 3 mg (46%). ^{1}H NMR (500.20 MHz, MeOD, 25 °C): δ = 7.37 – 7.20 (m, 5 H, aromatic H), 4.97 (dd, 1 H, $J_{H\text{-}1,H\text{-}2}$ = 1.7 Hz, $J_{H\text{-}2,H\text{-}3}$ = 3.5 Hz, H-2), 4.62 (d, 1 H, H-1), 3.843 (dd, 1 H, $J_{H\text{-}5,H\text{-}6a}$ = 2.1 Hz, $J_{H\text{-}6a,H\text{-}6b}$ = – 11.8 Hz, H-6a), 3.842 (dd, 1 H, $J_{H\text{-}3,H\text{-}4}$ = 9.3 Hz, H-3), 3.820 (ddd, 1 H, $J_{2\text{-}CO\underline{CH}CH3Ph,\ 2\text{-}COCH\underline{CH3}Ph}$ = 7.2 Hz, 2-COC*H*CH_3Ph), 3.66 (dd, 1 H, $J_{H\text{-}5,H\text{-}6b}$ = 6.1 Hz, H-6b), 3.53 (dd, 1 H, $J_{H\text{-}4,H\text{-}5}$ = 9.9 Hz,H-4), 3.51 (ddd, 1 H, H-5), 3.38 (s, 3 H, 1-OCH_3), 1.51 (d, 3 H, 2-COCHCH_3Ph) ppm. ^{13}C NMR (125.78 MHz, MeOD, 25 °C): δ = 175.7 (2-*C*OCHCH_3Ph), 141.8 – 128.1 (aromatic C), 99.7 (C-1), 74.6 (C-5), 74.0 (C-2), 70.8 (C-3), 68.9 (C-4), 62.9 (C-6), 55.3 (1-O*C*H_3), 46.9 (2-CO*C*HCH_3Ph), 19.1 (2-COCH*C*H_3Ph) ppm.

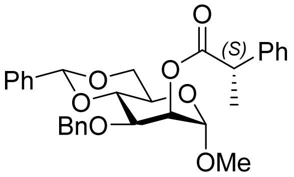

Methyl 3-*O*-benzyl-4,6-*O*-benzylidene-2-*O*-(*S*)-2-phenyl-propanoyl-α-D-mannopyranoside (85): Synthesized from **80** (50 mg) according to the standard reaction procedure B. The crude product was purified by column chromatography (hexane:EtOAc 3:1) to provide **85** as a white solid. Yield: 45 mg (71%), R_f = 0.45. ^{1}H NMR (500.20 MHz, $CDCl_3$, 25 °C): δ = 7.52 – 7.24 (m, 15 H, aromatic H), 5.60 (s, 1 H, 4,6-OC*H*Ph), 5.36 (dd, 1 H, $J_{H\text{-}1,H\text{-}2}$ = 1.7 Hz, $J_{H\text{-}2,H\text{-}3}$ = 3.4 Hz, H-2), 4.69 (d, 1 H, *J* = -12.1, 3-OCH_2Ph), 4.66 (d, 1 H, 3-OCH_2Ph), 4.55 (d, 1 H, H-1), 4.23 (dd, 1 H, $J_{H\text{-}5,H\text{-}6a}$ = 4.8 Hz, $J_{H\text{-}6a,H\text{-}6b}$ = – 10.2 Hz, H-6a), 3.99 (dd, 1 H, $J_{H\text{-}3,H\text{-}4}$ = 9.9 Hz, H-3), 3.95 (dd, 1 H, $J_{H\text{-}4,H\text{-}5}$ = 9.3 Hz, H-4), 3.84 (ddd, 1 H, $J_{2\text{-}CO\underline{CH}CH3Ph,\, 2\text{-}COCH\underline{CH3}Ph}$ = 7.2 Hz, 2-COC*H*CH_3Ph), 3.79 (ddd, 1 H, $J_{H\text{-}5,H\text{-}6b}$ = 10.4 Hz, H-5), 3.76 (dd, 1 H, H-6b), 3.32 (s, 3 H, 1-OCH_3), 1.53 (d, 3 H, 2-COCHCH_3Ph) ppm. ^{13}C NMR (125.78 MHz, $CDCl_3$, 25 °C): δ = 174.0 (2-*C*OCHCH_3Ph), 140.3 – 126.3 (aromatic C), 101.8 (4,6-*C*HPh), 99.7 (C-1), 78.6 (C-3), 74.0 (C-4), 72.2 (3-*C*H_2Ph), 70.0 (C-2), 69.0 (C-6), 63.8 (C-5), 55.2 (1-OCH_3), 45.6 (2-CO*C*HCH_3Ph), 18.7 (2-COCHCH_3Ph) ppm.

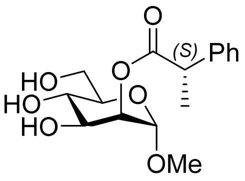

Methyl 2-*O*-(*S*)-2-phenyl-propanoyl-α-D-mannopyranoside (25): Synthesized from **85** (10 mg) according to the standard reaction procedure C to provide **25** as a white solid. Yield: 3 mg (46%). ^{1}H NMR (500.20 MHz, MeOD, 25 °C): δ = 7.32 – 7.21 (m, 5 H, aromatic H), 4.96 (dd, 1 H, $J_{H\text{-}1,H\text{-}2}$ = 1.7 Hz, $J_{H\text{-}2,H\text{-}3}$ = 3.5 Hz, H-2), 4.47 (d, 1 H, H-1), 3.841 (dd, 1 H, $J_{H\text{-}3,H\text{-}4}$ = 9.5 Hz, H-3), 3.839 (ddd, 1 H, $J_{2\text{-}CO\underline{CH}CH3Ph,\, 2\text{-}COCH\underline{CH3}Ph}$ = 7.2 Hz, 2-COC*H*CH_3Ph), 3.79 (dd, 1 H, $J_{H\text{-}5,H\text{-}6a}$ = 2.3 Hz, $J_{H\text{-}6a,H\text{-}6b}$ = – 11.8 Hz, H-6a), 3.55 (dd, 1 H, $J_{H\text{-}5,H\text{-}6b}$ = 6.5 Hz, H-6b), 3.49 (dd, 1 H, $J_{H\text{-}4,H\text{-}5}$ = 9.9 Hz,H-4), 3.46 (ddd, 1 H, H-5), 3.33 (s, 3 H, 1-OCH_3), 1.48 (d, 3 H, 2-COCHCH_3Ph) ppm. ^{13}C NMR (125.78 MHz, MeOD, 25 °C): δ = 175.7 (2-*C*OCHCH_3Ph), 142.0 – 128.1 (aromatic C), 99.5 (C-1), 74.6 (C-5), 74.0 (C-2), 70.7 (C-3), 69.0 (C-4), 63.0 (C-6), 55.3 (1-OCH_3), 46.5 (2-CO*C*HCH_3Ph), 18.9 (2-COCH*C*H_3Ph) ppm.

Xylopyranoside derivatives

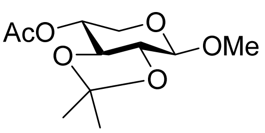

Methyl 4-*O*-acetyl-2,3-*O*-isopropylidene-β-D-xylopyranoside (87): Synthesized from **86** (50 mg) according to the standard procedure for acylation. The crude product was purified by column chromatography (hexane:EtOAc 4:1) to provide **87** as a white solid. Yield: 50 mg (83%), R_f = 0.38 (hexane:EtOAc 4:1). ^{1}H NMR (500.20 MHz, $CDCl_3$, 25 °C): δ = 4.96 (ddd, 1 H, $J_{H\text{-}3,H\text{-}4}$ = 9.0 Hz, $J_{H\text{-}4,H\text{-}5a}$ = 5.2 Hz, $J_{H\text{-}4,H\text{-}5b}$ = 5.8 Hz, H-4), 4.57 (d, 1 H, $J_{H\text{-}1,H\text{-}2}$ = 7.1 Hz, H-1), 4.13 (dd, 1 H, $J_{H\text{-}5a,H\text{-}5b}$ = – 12.5 Hz, H-5a), 3.71 (dd, 1 H, $J_{H\text{-}2,H\text{-}3}$ = 9.9 Hz, H-3), 3.47 (s, 3 H, 1-OCH_3), 3.41 (dd, 1 H, H-2), 3.29 (dd, 1 H, H-5b), 2.04 (s, 3 H, 4-OCOCH_3), 1.41 (s, 3 H, 2,3-OC$(CH_3)_2$), 1.39 (s, 3 H, 2,3-OC$(CH_3)_2$) ppm. ^{13}C NMR (125.78 MHz, $CDCl_3$, 25 °C): δ = 170.4 (4-*C*OCH_3), 112.5 (2,3-O*C*$(CH_3)_2$), 102.5 (C-1), 77.4 (C-3), 77.0 (C-2), 71.6 (C-4), 64.7 (C-5), 56.4 (1-OCH_3), 26.9 (2,3-OC$(CH_3)_2$), 26.8 (2,3-OC$(CH_3)_2$), 21.1 (4-COCH_3) ppm.

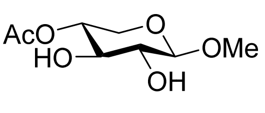

Methyl 4-*O*-acetyl-β-D-xylopyranoside (26): Synthesized from **87** (9 mg) according to the standard reaction procedure D to provide **26** as a white solid. Yield: 6.0 mg (80%). ^{1}H NMR (500.20 MHz, MeOD, 25 °C): δ = 4.67 (ddd, 1 H, $J_{H\text{-}3,H\text{-}4}$ = 9.1 Hz, $J_{H\text{-}4,H\text{-}5a}$ = 5.4 Hz, $J_{H\text{-}4,H\text{-}5b}$ = 10.0 Hz, H-4), 4.14 (d, 1 H, $J_{H\text{-}1,H\text{-}2}$ = 7.5 Hz, H-1), 3.96 (dd, 1 H, $J_{H\text{-}5a,H\text{-}5b}$ = – 11.5 Hz, H-5a), 3.54 (dd, 1 H, $J_{H\text{-}2,H\text{-}3}$ = 9.2 Hz, H-3), 3.49 (s, 3 H, 1-OCH_3), 3.24 (dd, 1 H, H-5b), 3.22 (dd, 1 H, H-2), 2.06 (s, 3 H, 4-OCOCH_3) ppm. ^{13}C NMR (125.78 MHz, MeOD, 25 °C): δ = 172.2 (4-*C*OCH_3), 106.0 (C-1), 74.93 (C-3), 74.90 (C-2), 73.1 (C-4), 63.6 (C-5), 57.2 (1-OCH_3), 20.8 (4-COCH_3) ppm.

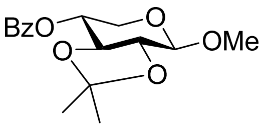

Methyl 4-*O*-benzoyl-2,3-*O*-isopropylidene-β-D-xylopyranoside (88): Synthesized from **86** (50 mg) according to the standard procedure for acylation. The crude product was purified by column chromatography (hexane:EtOAc 4:1) to provide **88** as a clear oil. Yield: 57 mg (76%), R_f = 0.40 (hexane:EtOAc 4:1). ^{1}H NMR (500.20 MHz, $CDCl_3$, 25 °C): δ = 8.08 – 8.03 (m, 2 H, aromatic H), 7.60 – 7.55 (m, 1 H, aromatic H), 7.47 – 7.42 (m, 2 H, aromatic H), 5.30 (ddd, 1 H, $J_{H-3,H-4}$ = 8.9 Hz, $J_{H-4,H-5a}$ = 5.2 Hz, $J_{H-4,H-5b}$ = 5.6 Hz, H-4), 4.71 (d, 1 H, $J_{H-1,H-2}$ = 7.1 Hz, H-1), 4.31 (dd, 1 H, $J_{H-5a,H-5b}$ = – 12.6 Hz, H-5a), 3.95 (dd, 1 H, $J_{H-2,H-3}$ = 9.9 Hz, H-3), 3.57 (dd, 1 H, H-2), 3.54 (s, 3 H, 1-OC*H*$_3$), 3.52 (dd, 1 H, H-5b), 1.50 (s, 3 H, 2,3-OC*(CH$_3$)$_2$*), 1.48 (s, 3 H, 2,3-OC*(CH$_3$)$_2$*) ppm. ^{13}C NMR (125.78 MHz, $CDCl_3$, 25 °C): δ = 165.9 (4-*C*OPh), 133.5, 130.0, 129.6, 128.5 (aromatic C), 112.6 (2,3-O*C*(CH$_3$)$_2$), 102.5 (C-1), 77.6 (C-3), 77.0 (C-2), 72.2 (C-4), 64.9 (C-5), 56.4 (1-O*C*H$_3$), 26.9 (2,3-OC*(CH$_3$)$_2$*), 26.8 (2,3-OC*(CH$_3$)$_2$*) ppm.

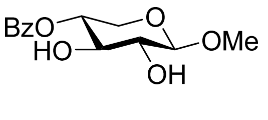

Methyl 4-*O*-benzoyl-β-D-xylopyranoside (27): Synthesized from **88** (10 mg) according to the standard reaction procedure D to provide **27** as a white solid. Yield: 7.0 mg (80%). ^{1}H NMR (500.20 MHz, MeOD, 25 °C): δ = 8.07 – 8.03 (m, 2 H, aromatic H), 7.64 – 7.59 (m, 1 H, aromatic H), 7.51 – 7.46 (m, 2 H, aromatic H), 4.93 (ddd, 1 H, $J_{H-3,H-4}$ = 9.1 Hz, $J_{H-4,H-5a}$ = 5.4 Hz, $J_{H-4,H-5b}$ = 9.9Hz, H-4), 4.22 (d, 1 H, $J_{H-1,H-2}$ = 7.5 Hz, H-1), 4.10 (dd, 1 H, $J_{H-5a,H-5b}$ = – 11.5 Hz, H-5a), 3.73 (dd, 1 H, $J_{H-2,H-3}$ = 9.1 Hz, H-3), 3.51 (s, 3 H, 1-OC*H*$_3$), 3.39 (dd, 1 H, H-5b), 3.30 (dd, 1 H, H-2) ppm. ^{13}C NMR (125.78 MHz, MeOD, 25 °C): δ = 167.5 (4-*C*OPh), 134.4, 130.7, 129.5 (aromatic C), 106.0 (C-1), 75.04 (C-2), 74.96 (C-3), 73.7 (C-4), 63.7 (C-5), 57.3 (1-O*C*H$_3$) ppm.

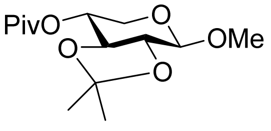

Methyl 2,3-*O*-isopropylidene-4-*O*-pivaoyl-β-D-xylopyranoside (89): Synthesized from **86** (50 mg) according to the standard procedure for acylation. The crude product was purified by column chromatography (hexane:EtOAc 5:1) to provide **89** as a clear oil. Yield: 25 mg (35%), R_f = 0.32 (hexane:EtOAc 5:1). ^{1}H NMR (500.20 MHz, $CDCl_3$, 25 °C): δ = 5.02 (ddd, 1 H, $J_{H-3,H-4}$ = 8.8 Hz, $J_{H-4,H-5a}$ = 5.2 Hz, $J_{H-4,H-5b}$ = 5.3 Hz, H-4), 4.66 (d, 1 H, $J_{H-1,H-2}$ = 7.0 Hz, H-1), 4.17 (dd, 1 H, $J_{H-5a,H-5b}$ = – 12.6 Hz, H-5a), 3.79 (dd, 1 H, $J_{H-2,H-3}$ = 9.9 Hz, H-3), 3.51 (s, 3 H, 1-OC*H*$_3$), 3.49 (dd, 1 H, H-2), 3.34 (dd, 1 H, H-5b), 1.47 (s, 3 H, 2,3-OC*(CH$_3$)$_2$*), 1.45 (s, 3 H, 2,3-OC*(CH$_3$)$_2$*), 1.22 (s, 9 H, 4-OCOC(C*H*$_3$)$_3$) ppm. ^{13}C NMR (125.78 MHz, $CDCl_3$, 25 °C): δ = 177.9 (4-*C*OC(C*H*$_3$)$_3$), 112.3 (2,3-O*C*(CH$_3$)$_2$), 102.3 (C-1), 77.4 (C-3), 76.8 (C-2), 71.4 (C-4), 64.8 (C-5), 56.2 (1-O*C*H$_3$), 38.8 (4-CO*C*(C*H*$_3$)$_3$), 27.1 (4-COC*(CH$_3$)$_3$*), 26.8 (2,3-OC*(CH$_3$)$_2$*), 26.7 (2,3-OC*(CH$_3$)$_2$*) ppm.

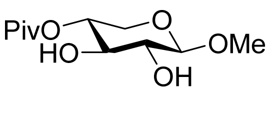

Methyl 4-*O*-pivaoyl-β-D-xylopyranoside (28): Synthesized from **89** (4 mg) according to the standard reaction procedure D to provide **28** as a white solid. Yield: 2.5 mg (73%). ^{1}H NMR (500.20 MHz, MeOD, 25 °C): δ = 4.66 (ddd, 1 H, $J_{H-3,H-4}$ = 9.3 Hz, $J_{H-4,H-5a}$ = 5.4 Hz, $J_{H-4,H-5b}$ = 9.9 Hz, H-4), 4.15 (d, 1 H, $J_{H-1,H-2}$ = 7.5 Hz, H-1), 3.93 (dd, 1 H, $J_{H-5a,H-5b}$ = – 11.4 Hz, H-5a), 3.55 (dd, 1 H, $J_{H-2,H-3}$ = 9.1 Hz, H-3), 3.49 (s, 3 H, 1-OC*H*$_3$), 3.23 (dd, 1 H, H-5b), 3.22 (dd, 1 H, H-2), 1.20 (s, 9 H, 4-OCOC(C*H*$_3$)$_3$) ppm. ^{13}C NMR (125.78 MHz, MeOD, 25 °C): δ = 179.5 (4-*C*OC(C*H*$_3$)$_3$, 106.0 (C-1), 75.1 (C-2), 75.0 (C-3), 72.9 (C-4), 63.6 (C-5), 57.2 (1-O*C*H$_3$), 39.9 (4-CO*C*(C*H*$_3$)$_3$), 27.5 (4-COC*(CH$_3$)$_3$*) ppm.

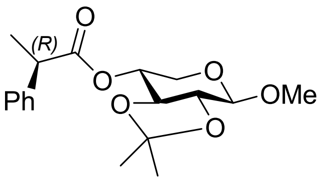

Methyl 2,3-*O*-isopropylidene-4-*O*-(*R*)-2-phenyl-propanoyl-β-D-xylopyranoside (90): Synthesized from **86** (50 mg) according to the standard reaction procedure B. The crude product was purified by column chromatography (hexane:EtOAc 5:1) to give **90** as a clear oil. Yield: 42 mg (46%), R_f = 0.28 (hexane:EtOAc 5:1). ^{1}H NMR (500.20 MHz, $CDCl_3$, 25 °C): δ = 7.34 – 7.23 (m, 5 H, aromatic H), 5.05 (ddd, 1 H, $J_{H-3,H-4}$ = 8.8 Hz, $J_{H-4,H-5a}$ = 5.2 Hz, $J_{H-4,H-5b}$ = 5.5 Hz, H-4), 4.62 (d, 1 H, $J_{H-1,H-2}$ = 7.1 Hz, H-1), 4.15 (dd, 1 H, $J_{H-5a,H-5b}$ = – 12.6 Hz, H-5a), 3.77 (ddd, 1 H, $J_{4-CO\underline{CH}CH3Ph,\ 4-COCH\underline{CH3}Ph}$ = 7.2 Hz, 4-COC*H*CH_3Ph), 3.71 (dd, 1 H, $J_{H-2,H-3}$ = 10.0 Hz, H-3), 3.49 (s, 3 H, 1-OCH_3), 3.46 (dd, 1 H, H-2), 3.35 (dd, 1 H, H-5b), 1.53 (d, 3 H, 4-COCHCH_3Ph), 1.43 (s, 3 H, 2,3-OC$(CH_3)_2$), 1.42 (s, 3 H, 2,3-OC$(CH_3)_2$) ppm. ^{13}C NMR (125.78 MHz, $CDCl_3$, 25 °C): δ = 174.0 (4-*C*OCHCH_3Ph), 140.1, 128.7, 127.7, 127.3 (aromatic C), 112.4 (2,3-O*C*$(CH_3)_2$), 102.4 (C-1), 77.5 (C-3), 76.9 (C-2), 71.8 (C-4), 64.7 (C-5), 56.3 (1-OCH_3), 45.5 (4-CO*CH*CH_3Ph), 26.9 (2,3-OC$(CH_3)_2$), 26.7 (2,3-OC$(CH_3)_2$), 18.9 (4-COCHCH_3Ph) ppm.

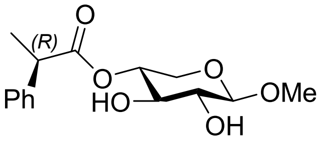

Methyl 4-*O*-(*R*)-2-phenyl-propanoyl-β-D-xylopyranoside (29): Synthesized from **90** (10 mg) according to the standard reaction procedure D to provide **29** as a white solid. Yield: 5 mg (57%). ^{1}H NMR (500.20 MHz, MeOD, 25 °C): δ = 7.33 – 7.20 (m, 5 H, aromatic H), 4.68 (ddd, 1 H, $J_{H-3,H-4}$ = 9.0 Hz, $J_{H-4,H-5a}$ = 5.4 Hz, $J_{H-4,H-5b}$ = 9.9 Hz, H-4), 4.13 (d, 1 H, $J_{H-1,H-2}$ = 7.5 Hz, H-1), 3.95 (dd, 1 H, $J_{H-5a,H-5b}$ = – 11.5 Hz, H-5a), 3.79 (ddd, 1 H, $J_{4-CO\underline{CH}CH3Ph,\ 4-COCH\underline{CH3}Ph}$ = 7.2 Hz, 4-COC*H*CH_3Ph), 3.49 (dd, 1 H, $J_{H-2,H-3}$ = 9.1 Hz, H-3), 3.47 (s, 3 H, 1-OCH_3), 3.24 (dd, 1 H, H-5b), 3.19 (dd, 1 H, H-2), 1.48 (d, 3 H, 4-COCHCH_3Ph) ppm. ^{13}C NMR (125.78 MHz, MeOD, 25 °C): δ = 175.7 (4-*C*OCHCH_3Ph), 141.8, 129.6, 128.6, 128.1 (aromatic C), 106.0 (C-1), 75.1 (C-2), 74.8 (C-3), 73.3 (C-4), 63.5 (C-5), 57.2 (1-OCH_3), 46.7 (4-CO*CH*CH_3Ph), 19.2 (4-COCHCH_3Ph) ppm.

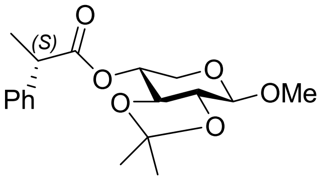

Methyl 2,3-*O*-isopropylidene-4-*O*-(*S*)-2-phenyl-propanoyl-β-D-xylopyranoside (91): Synthesized from **86** (50 mg) according to the standard reaction procedure B. The crude product was purified by column chromatography (hexane:EtOAc 4:1) to give **91** as a clear oil. Yield: 50 mg (61%), R_f = 0.37 (hexane:EtOAc 4:1). ^{1}H NMR (500.20 MHz, $CDCl_3$, 25 °C): δ = 7.36 – 7.23 (m, 5 H, aromatic H), 5.04 (ddd, 1 H, $J_{H-3,H-4}$ = 9.1 Hz, $J_{H-4,H-5a}$ = 5.3 Hz, $J_{H-4,H-5b}$ = 5.9 Hz, H-4), 4.56 (d, 1 H, $J_{H-1,H-2}$ = 7.0 Hz, H-1), 4.05 (dd, 1 H, $J_{H-5a,H-5b}$ = – 12.5 Hz, H-5a), 3.77 (ddd, 1 H, $J_{4-CO\underline{CH}CH3Ph,\ 4-COCH\underline{CH3}Ph}$ = 7.1 Hz, 4-COC*H*CH_3Ph), 3.73 (dd, 1 H, $J_{H-2,H-3}$ = 9.6 Hz, H-3), 3.46 (s, 3 H, 1-OCH_3), 3.45 (dd, 1 H, H-2), 3.08 (dd, 1 H, H-5b), 1.48 (d, 3 H, 4-COCHCH_3Ph), 1.47 (s, 3 H, 2,3-OC$(CH_3)_2$), 1.46 (s, 3 H, 2,3-OC$(CH_3)_2$) ppm. ^{13}C NMR (125.78 MHz, $CDCl_3$, 25 °C): δ = 174.0 (4-*C*OCHCH_3Ph), 140.5, 128.9, 127.42, 127.39 (aromatic C), 112.5 (2,3-O*C*$(CH_3)_2$), 102.4 (C-1), 77.2 (C-3), 76.0 (C-2), 71.7 (C-4), 64.4 (C-5), 56.4 (1-OCH_3), 45.3 (4-CO*CH*CH_3Ph), 26.9 (2,3-OC$(CH_3)_2$), 26.8 (2,3-OC$(CH_3)_2$), 18.7 (4-COCHCH_3Ph) ppm.

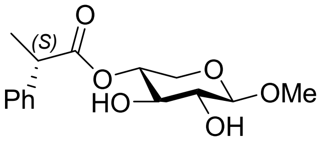

Methyl 4-*O*-(*S*)-2-phenyl-propanoyl-β-D-xylopyranoside (30): Synthesized from **91** (10 mg) according to the standard reaction procedure D to provide **30** as a white solid. Yield: 6 mg (66%). ^{1}H NMR (500.20 MHz, MeOD, 25 °C): δ = 7.33 – 7.20 (m, 5 H, aromatic H), 4.69 (ddd, 1 H, $J_{H-3,H-4}$ = 9.1 Hz, $J_{H-4,H-5a}$ = 5.5 Hz, $J_{H-4,H-5b}$ = 10.0 Hz, H-4), 4.08 (d, 1 H, $J_{H-1,H-2}$ = 7.5 Hz, H-1), 3.80 (ddd, 1 H, $J_{4-CO\underline{CH}CH3Ph,\ 4-COCH\underline{CH3}Ph}$ = 7.1 Hz, 4-COC*H*CH_3Ph), 3.79 (dd, 1 H, $J_{H-5a,H-5b}$ = – 11.5 Hz, H-5a), 3.50

(dd, 1 H, $J_{\text{H-2,H-3}}$ = 9.2 Hz, H-3), 3.45 (s, 3 H, 1-OCH_3), 3.20 (dd, 1 H, H-2), 3.02 (dd, 1 H, H-5b), 1.45 (d, 3 H, 4-COCHCH_3Ph) ppm. ^{13}C NMR (125.78 MHz, MeOD, 25 °C): δ = 175.7 (4-*C*OCHCH$_3$Ph), 142.2, 129.7, 128.4, 128.2 (aromatic C), 105.9 (C-1), 75.0 (C-2), 74.8 (C-3), 73.2 (C-4), 63.3 (C-5), 57.2 (1-O*C*H$_3$), 46.6 (4-CO*C*HCH$_3$Ph), 19.1 (4-COCH*C*H$_3$Ph) ppm.

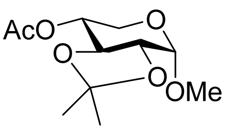

Methyl 4-*O*-acetyl-2,3-*O*-isopropylidene-α-D-xylopyranoside (93): Synthesized from **92** (50 mg) according to the standard procedure for acylation. The crude product was purified by column chromatography (hexane:EtOAc 4:1) to provide **93** as a white solid. Yield: 25 mg (38%), R_f = 0.25 hexane:EtOAc 4:1). ^{1}H NMR (500.20 MHz, CDCl$_3$, 25 °C): δ = 5.03 (ddd, 1 H, $J_{\text{H-3,H-4}}$ = 10.0 Hz, $J_{\text{H-4,H-5a}}$ = 5.4 Hz, $J_{\text{H-4,H-5b}}$ = 10.0 Hz, H-4), 5.02 (d, 1 H, $J_{\text{H-1,H-2}}$ = 3.0 Hz, H-1), 4.09 (dd, 1 H, $J_{\text{H-2,H-3}}$ = 9.5 Hz, H-3), 3.91 (dd, 1 H, $J_{\text{H-5a,H-5b}}$ = – 11.0 Hz, H-5a), 3.53 (dd, 1 H, H-2), 3.46 (s, 3 H, 1-OCH_3), 3.29 (dd, 1 H, H-5b), 2.09 (s, 3 H, 4-OCOCH_3), 1.49 (s, 3 H, 2,3-OC$(CH_3)_2$), 1.45 (s, 3 H, 2,3-OC$(CH_3)_2$) ppm. ^{13}C NMR (125.78 MHz, CDCl$_3$, 25 °C): δ = 170.2 (4-*C*OCH$_3$), 111.0 (2,3-O*C*(CH$_3$)$_2$), 97.9 (C-1), 76.1 (C-2), 73.7 (C-3), 71.5 (C-4), 59.6 (C-5), 55.8 (1-O*C*H$_3$), 26.9 (2,3-OC$(CH_3)_2$), 26.6 (2,3-OC$(CH_3)_2$), 21.1 (4-CO*C*H$_3$) ppm.

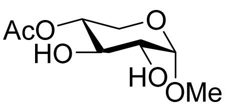

Methyl 4-*O*-acetyl-α-D-xylopyranoside (31): Synthesized from **93** (10 mg) according to the standard reaction procedure D to provide **31** as a white solid. Yield: 6.0 mg (72%). ^{1}H NMR (500.20 MHz, MeOD, 25 °C): δ = 4.67 (ddd, 1 H, $J_{\text{H-3,H-4}}$ = 9.1 Hz, $J_{\text{H-4,H-5a}}$ = 5.8 Hz, $J_{\text{H-4,H-5b}}$ = 10.5 Hz, H-4), 4.64 (d, 1 H, $J_{\text{H-1,H-2}}$ = 3.7 Hz, H-1), 3.75 (dd, 1 H, $J_{\text{H-2,H-3}}$ = 9.7 Hz, H-3), 3.64 (dd, 1 H, $J_{\text{H-5a,H-5b}}$ = – 10.9 Hz, H-5a), 3.443 (dd, 1 H, H-5b), 3.440 (dd, 1 H, H-2), 3.39 (s, 3 H, 1-OCH_3), 2.05 (s, 3 H, 4-OCOCH_3) ppm. ^{13}C NMR (125.78 MHz, MeOD, 25 °C): δ = 172.2 (4-*C*OCH$_3$), 101.4 (C-1), 73.5 (C-2), 73.3 (C-4), 72.2 (C-3), 59.4 (C-5), 55.7 (1-O*C*H$_3$), 20.8 (4-CO*C*H$_3$) ppm.

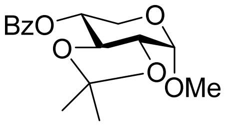

Methyl 4-*O*-benzoyl-2,3-*O*-isopropylidene-α-D-xylopyranoside (94): Synthesized from **92** (50 mg) according to the standard procedure for acylation. The crude product was purified by column chromatography (hexane:EtOAc 4:1) to provide **94** as a clear oil. Yield: 40 mg (49%), R_f = 0.36 (hexane:EtOAc 4:1). ^{1}H NMR (500.20 MHz, CDCl$_3$, 25 °C): δ = 8.08 – 8.03 (m, 2 H, aromatic H), 7.60 – 7.54 (m, 1 H, aromatic H), 7.47 – 7.41 (m, 2 H, aromatic H), 5.31 (ddd, 1 H, $J_{\text{H-3,H-4}}$ = 9.9 Hz, $J_{\text{H-4,H-5a}}$ = 5.5 Hz, $J_{\text{H-4,H-5b}}$ = 10.0 Hz, H-4), 5.07 (d, 1 H, $J_{\text{H-1,H-2}}$ = 2.9 Hz, H-1), 4.25 (dd, 1 H, $J_{\text{H-2,H-3}}$ = 9.5 Hz, H-3), 4.03 (dd, 1 H, $J_{\text{H-5a,H-5b}}$ = – 11.0 Hz, H-5a), 3.62 (dd, 1 H, H-2), 3.49 (s, 3 H, 1-OCH_3), 3.44 (dd, 1 H, H-5b), 1.51 (s, 3 H, 2,3-OC$(CH_3)_2$), 1.46 (s, 3 H, 2,3-OC$(CH_3)_2$) ppm. ^{13}C NMR (125.78 MHz, CDCl$_3$, 25 °C): δ = 165.7 (4-*C*OPh), 133.5, 130.0, 129.6, 128.5 (aromatic C), 111.1 (2,3-O*C*(CH$_3$)$_2$), 98.0 (C-1), 76.1 (C-2), 73.9 (C-3), 71.8 (C-4), 59.7 (C-5), 55.9 (1-O*C*H$_3$), 26.9 (2,3-OC$(CH_3)_2$), 26.7 (2,3-OC$(CH_3)_2$) ppm.

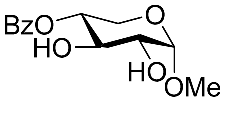

Methyl 4-*O*-benzoyl-α-D-xylopyranoside (32): Synthesized from **94** (10 mg) according to the standard reaction procedure D to provide **32** as a white solid. Yield: 8.0 mg (92%). ^{1}H NMR (500.20 MHz, MeOD, 25 °C): δ = 8.07 – 8.02 (m, 2 H, aromatic H), 7.64 – 7.58 (m, 1 H, aromatic H), 7.51 – 7.45 (m, 2 H, aromatic H), 4.93 (ddd, 1 H, $J_{\text{H-3,H-4}}$ = 9.1 Hz, $J_{\text{H-4,H-5a}}$ = 5.8 Hz, $J_{\text{H-4,H-5b}}$ = 10.6 Hz, H-4), 4.70 (d, 1 H, $J_{\text{H-1,H-2}}$ = 3.7 Hz, H-1), 3.94 (dd, 1 H, $J_{\text{H-2,H-3}}$ = 9.7 Hz, H-3), 3.79 (dd, 1 H, $J_{\text{H-5a,H-5b}}$ = – 10.9 Hz, H-5a), 3.59 (dd, 1 H, H-5b), 3.52 (dd, 1 H, H-2), 3.42 (s, 3 H, 1-OCH_3) ppm. ^{13}C NMR (125.78 MHz, MeOD, 25 °C): δ = 167.5 (4-*C*OPh), 134.4, 131.2, 130.7, 129.5 (aromatic C), 101.5 (C-1), 73.8 (C-4), 73.6 (C-2), 72.3 (C-3), 59.6 (C-5), 55.8 (1-O*C*H$_3$) ppm.

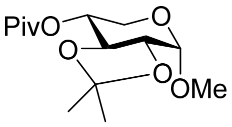

Methyl 2,3-*O*-isopropylidene-4-*O*-pivaoyl-α-D-xylopyranoside (95): Synthesized from **92** (50 mg) according to the standard procedure for acylation. The crude product was purified by column chromatography (hexane:EtOAc 4:1) to provide **95** as a clear oil. Yield: 39 mg (51%), R_f = 0.38 (hexane:EtOAc 4:1). ^{1}H NMR (500.20 MHz, $CDCl_3$, 25 °C): δ = 5.03 (ddd, 1 H, $J_{H-3,H-4}$ = 10.0 Hz, $J_{H-4,H-5a}$ = 5.4 Hz, $J_{H-4,H-5b}$ = 10.0 Hz, H-4), 5.02 (d, 1 H, $J_{H-1,H-2}$ = 3.0 Hz, H-1), 4.07 (dd, 1 H, $J_{H-2,H-3}$ = 9.5 Hz, H-3), 3.85 (dd, 1 H, $J_{H-5a,H-5b}$ = – 11.0 Hz, H-5a), 3.53 (dd, 1 H, H-2), 3.47 (s, 3 H, 1-OCH_3), 3.26 (dd, 1 H, H-5b), 1.47 (s, 3 H, 2,3-OC$(CH_3)_2$), 1.44 (s, 3 H, 2,3-OC$(CH_3)_2$), 1.21 (s, 9 H, 4-OCOC(CH_3)$_3$) ppm. ^{13}C NMR (125.78 MHz, $CDCl_3$, 25 °C): δ = 177.8 (4-*C*OC(CH_3)$_3$), 110.9 (2,3-O*C*(CH$_3$)$_2$), 98.0 (C-1), 76.0 (C-2), 73.9 (C-3), 71.1 (C-4), 59.6 (C-5), 55.8 (1-O*C*H$_3$), 39.0 (4-CO*C*(CH_3)$_3$), 27.3 (4-COC$(CH_3)_3$), 27.0 (2,3-OC$(CH_3)_2$), 26.6 (2,3-OC$(CH_3)_2$) ppm.

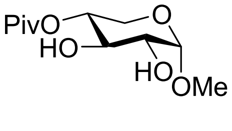

Methyl 4-*O*-pivaoyl-α-D-xylopyranoside (33): Synthesized from **95** (10 mg) according to the standard reaction procedure D to provide **33** as a white solid. Yield: 8 mg (33%). ^{1}H NMR (500.20 MHz, MeOD, 25 °C): δ = 4.65 (ddd, 1 H, $J_{H-3,H-4}$ = 9.2 Hz, $J_{H-4,H-5a}$ = 5.8 Hz, $J_{H-4,H-5b}$ = 10.7 Hz, H-4), 4.65 (d, 1 H, $J_{H-1,H-2}$ = 3.7 Hz, H-1), 3.76 (dd, 1 H, $J_{H-2,H-3}$ = 9.8 Hz, H-3), 3.62 (dd, 1 H, $J_{H-5a,H-5b}$ = – 10.7 Hz, H-5a), 3.44 (dd, 1 H, H-2), 3.42 (dd, 1 H, H-5b), 3.39 (s, 3 H, 1-OCH_3), 1.20 (s, 9 H, 4-OCOC(CH_3)$_3$) ppm. ^{13}C NMR (125.78 MHz, MeOD, 25 °C): δ = 179.5 (4-*C*OC(CH_3)$_3$, 101.5 (C-1), 73.7 (C-2), 73.0 (C-4), 72.3 (C-3), 59.5 (C-5), 55.7 (1-O*C*H$_3$), 39.9 (4-CO*C*(CH_3)$_3$), 27.5 (4-COC$(CH_3)_3$) ppm.

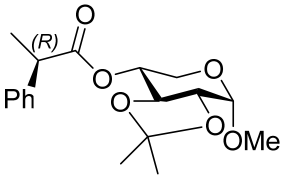

Methyl 2,3-*O*-isopropylidene-4-*O*-(*R*)-2-phenyl-propanoyl-α-D-xylopyranoside (96): Synthesized from **92** (50 mg) according to the standard reaction procedure B. The crude product was purified by column chromatography (hexane:EtOAc 4:1) to give **96** as a clear oil. Yield: 50 mg (56%), R_f = 0.33 (hexane:EtOAc 4:1). ^{1}H NMR (500.20 MHz, $CDCl_3$, 25 °C): δ = 7.34 – 7.23 (m, 5 H, aromatic H), 5.06 (ddd, 1 H, $J_{H-3,H-4}$ = 9.9 Hz, $J_{H-4,H-5a}$ = 5.5 Hz, $J_{H-4,H-5b}$ = 10.1 Hz, H-4), 5.00 (d, 1 H, $J_{H-1,H-2}$ = 3.0 Hz, H-1), 4.01 (dd, 1 H, $J_{H-2,H-3}$ = 9.5 Hz, H-3), 3.85 (dd, 1 H, $J_{H-5a,H-5b}$ = – 11.0 Hz, H-5a), 3.76 (ddd, 1 H, $J_{4\text{-CO}\underline{CH}\text{CH3Ph}, 4\text{-COCH}\underline{CH3}\text{Ph}}$ = 7.2 Hz, 4-CO*CH*CH$_3$Ph), 3.50 (dd, 1 H, H-2), 3.45 (s, 3 H, 1-OCH_3),3.28 (dd, 1 H, H-5b), 1.52 (d, 3 H, 4-COCHCH_3Ph), 1.44 (s, 3 H, 2,3-OC$(CH_3)_2$), 1.42 (s, 3 H, 2,3-OC$(CH_3)_2$) ppm. ^{13}C NMR (125.78 MHz, $CDCl_3$, 25 °C): δ = 173.8 (4-*C*OCHCH$_3$Ph), 140.1, 128.7, 127.7, 127.3 (aromatic C), 110.9 (2,3-O*C*(CH$_3$)$_2$), 97.9 (C-1), 75.9 (C-2), 73.9 (C-3), 71.4 (C-4), 59.5 (C-5), 55.8 (1-O*C*H$_3$), 45.5 (4-CO*CH*CH$_3$Ph), 27.0 (2,3-OC$(CH_3)_2$), 26.5 (2,3-OC$(CH_3)_2$), 19.0 (4-COCH*CH$_3$*Ph) ppm.

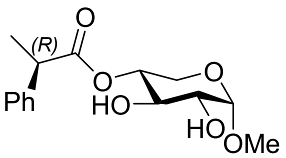

Methyl 4-*O*-(*R*)-2-phenyl-propanoyl-α-D-xylopyranoside (34): Synthesized from **96** (10 mg) according to the standard reaction procedure D to provide **34** as a white solid. Yield: 8.5 mg (96%). ^{1}H NMR (500.20 MHz, MeOD, 25 °C): δ = 7.33 – 7.20 (m, 5 H, aromatic H), 4.68 (ddd, 1 H, $J_{H-3,H-4}$ = 9.1 Hz, $J_{H-4,H-5a}$ = 5.8 Hz, $J_{H-4,H-5b}$ = 10.5 Hz, H-4), 4.63 (d, 1 H, $J_{H-1,H-2}$ = 3.4 Hz, H-1), 3.78 (ddd, 1 H, $J_{4\text{-CO}\underline{CH}\text{CH3Ph}, 4\text{-COCH}\underline{CH3}\text{Ph}}$ = 7.2 Hz, 4-CO*CH*CH$_3$Ph), 3.70 (dd, 1 H, $J_{H-2,H-3}$ = 9.7 Hz, H-3), 3.63 (dd, 1 H, $J_{H-5a,H-5b}$ = – 10.9 Hz, H-5a), 3.43 (dd, 1 H, H-5b), 3.41 (dd, 1 H, H-2), 3.38 (s, 3 H, 1-OCH_3), 1.48 (d, 3 H, 4-COCHCH_3Ph) ppm. ^{13}C NMR (125.78 MHz, MeOD, 25 °C): δ = 175.7 (4-*C*OCHCH$_3$Ph), 141.8, 129.6, 128.6, 128.1 (aromatic C), 101.4 (C-1), 73.6 (C-2), 73.4 (C-4), 72.2 (C-3), 59.5 (C-5), 55.7 (1-O*C*H$_3$), 46.7 (4-CO*CH*CH$_3$Ph), 19.2 (4-COCH*CH$_3$*Ph) ppm.

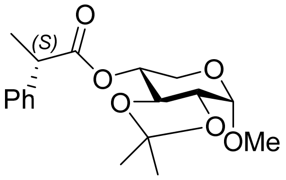

Methyl 2,3-*O*-isopropylidene-4-*O*-(*S*)-2-phenyl-propanoyl-α-D-xylopyranoside (97): Synthesized from **92** (50 mg) according to the standard reaction procedure B. The crude product was purified by column chromatography (hexane:EtOAc 4:1) to give **97** as a clear oil. Yield: 50 mg (56%), R_f = 0.32 (hexane:EtOAc 4:1). ^{1}H NMR (500.20 MHz, $CDCl_3$, 25 °C): δ = 7.36 – 7.23 (m, 5 H, aromatic H), 5.05 (ddd, 1 H, $J_{H-3,H-4}$ = 10.0 Hz, $J_{H-4,H-5a}$ = 5.4 Hz, $J_{H-4,H-5b}$ = 10.1 Hz, H-4), 4.98 (d, 1 H, $J_{H-1,H-2}$ = 2.9Hz, H-1), 4.03 (dd, 1 H, $J_{H-2,H-3}$ = 9.5 Hz, H-3), 3.77 (ddd, 1 H, $J_{4-CO\underline{CH}CH3Ph,\ 4-COCH\underline{CH3}Ph}$ = 7.0 Hz, 4-COC*H*CH_3Ph), 3.71 (dd, 1 H, $J_{H-5a,H-5b}$ = – 11.0 Hz, H-5a), 3.51 (dd, 1 H, H-2), 3.39 (s, 3 H, 1-OCH_3), 3.03 (dd, 1 H, H-5b), 1.48 (s, 3 H, 2,3-OC$(CH_3)_2$), 1.47 (d, 3 H, 4-COCHCH_3Ph), 1.45 (s, 3 H, 2,3-OC$(CH_3)_2$) ppm. ^{13}C NMR (125.78 MHz, $CDCl_3$, 25 °C): δ = 173.9 (4-*C*OCHCH$_3$Ph), 140.6, 128.9, 127.4, 127.3 (aromatic C), 111.0 (2,3-O*C*$(CH_3)_2$), 97.9 (C-1), 76.0 (C-2), 73.6 (C-3), 71.5 (C-4), 59.2 (C-5), 55.8 (1-OCH_3), 45.3 (4-CO*CH*CH_3Ph), 26.9 (2,3-OC$(CH_3)_2$), 26.6 (2,3-OC$(CH_3)_2$), 18.7 (4-COCHCH_3Ph) ppm.

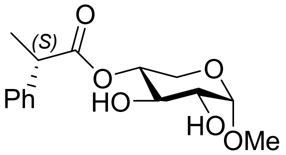

Methyl 4-*O*-(*S*)-2-phenyl-propanoyl-α-D-xylopyranoside (35): Synthesized from **97** (10 mg) according to the standard reaction procedure D to provide **35** as a white solid. Yield: 8.4 mg (95%). ^{1}H NMR (500.20 MHz, MeOD, 25 °C): δ = 7.33 – 7.20 (m, 5 H, aromatic H), 4.68 (ddd, 1 H, $J_{H-3,H-4}$ = 9.2 Hz, $J_{H-4,H-5a}$ = 5.8 Hz, $J_{H-4,H-5b}$ = 10.6 Hz, H-4), 4.61 (d, 1 H, $J_{H-1,H-2}$ = 3.7 Hz, H-1), 3.79 (ddd, 1 H, $J_{4-CO\underline{CH}CH3Ph,\ 4-COCH\underline{CH3}Ph}$ = 7.1 Hz, 4-COC*H*CH_3Ph), 3.71 (dd, 1 H, $J_{H-2,H-3}$ = 9.7 Hz, H-3), 3.47 (dd, 1 H, $J_{H-5a,H-5b}$ = – 10.9 Hz, H-5a), 3.43 (dd, 1 H, H-2), 3.32 (s, 3 H, 1-OCH_3), 3.20 (dd, 1 H, H-5b), 1.45 (d, 3 H, 4-COCHCH_3Ph) ppm. ^{13}C NMR (125.78 MHz, MeOD, 25 °C): δ = 175.7 (4-*C*OCHCH$_3$Ph), 142.2, 129.7, 128.4, 128.2 (aromatic C), 101.4 (C-1), 73.5 (C-2), 73.2 (C-4), 72.1 (C-3), 59.1 (C-5), 55.7 (1-OCH_3), 46.6 (4-CO*CH*CH_3Ph), 19.1 (4-COCHCH_3Ph) ppm.

Ribopyranoside derivatives

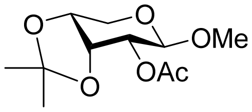

Methyl 2-*O*-acetyl-3,4-*O*-isopropylidene-β-D-ribopyranoside (99): Synthesized from **98** (50 mg) according to the standard procedure for acylation. The crude product was purified by column chromatography (hexane:EtOAc 3:1) to provide **99** as a clear oil. Yield: 40 mg (61%), R_f = 0.23 (hexane:EtOAc 3:1). ^{1}H NMR (500.20 MHz, $CDCl_3$, 25 °C): δ = 4.90 (dd, 1 H, $J_{H-1,H-2}$ = 6.2 Hz, $J_{H-2,H-3}$ = 3.3 Hz, H-2), 4.75 (d, 1 H, H-1), 4.56 (dd, 1 H, $J_{H-3,H-4}$ = 7.2Hz, H-3), 4.31 (ddd, 1 H, $J_{H-4,H-5a}$ = 2.6 Hz, $J_{H-4,H-5b}$ = 1.7 Hz, H-4), 3.79 (dd, 1 H, $J_{H-5a,H-5b}$ = – 13.1 Hz, H-5a), 3.69 (dd, 1 H, H-5b), 3.41 (s, 3 H, 1-OCH_3), 2.17 (s, 3 H, 2-OCOCH_3), 1.55 (s, 3 H, 3,4-OC$(CH_3)_2$), 1.34 (s, 3 H, 3,4-OC$(CH_3)_2$) ppm. ^{13}C NMR (125.78 MHz, $CDCl_3$, 25 °C): δ = 170.5 (2-*C*OCH$_3$), 110.4 (3,4-O*C*$(CH_3)_2$), 98.8 (C-1), 73.6 (C-4), 71.8 (C-3), 70.2 (C-2), 62.4 (C-5), 55.7 (1-OCH_3), 26.5 (3,4-OC$(CH_3)_2$), 25.3 (3,4-OC$(CH_3)_2$), 21.3 (2-COCH_3) ppm.

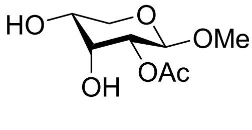

Methyl 2-*O*-acetyl-β-D-ribopyranoside (36): Synthesized from **99** (10 mg) according to the standard reaction procedure D to provide **36** as a white solid. Yield: 8 mg (96%). ^{1}H NMR (500.20 MHz, MeOD, 25 °C): δ = 4.67 (dd, 1 H, $J_{H-1,H-2}$ = 5.2 Hz, $J_{H-2,H-3}$ = 3.4 Hz, H-2), 4.64 (d, 1 H, H-1), 4.01 (dd, 1 H, $J_{H-3,H-4}$ = 3.0 Hz, H-3), 3.76 (dd, 1 H, $J_{H-4,H-5a}$ = 3.9 Hz, $J_{H-5a,H-5b}$ = – 11.8 Hz, H-5a), 3.74 (ddd, 1 H, $J_{H-4,H-5b}$ = 7.0 Hz, H-4), 3.68 (dd, 1 H, H-5b), 3.40 (s, 3 H, 1-OCH_3), 2.10 (s, 3 H, 2-OCOCH_3) ppm. ^{13}C NMR (125.78 MHz, MeOD, 25 °C): δ = 172.0 (2-*C*OCH$_3$), 100.5 (C-1), 73.3 (C-2), 68.8 (C-4), 68.2 (C-3), 64.7 (C-5), 56.3 (1-OCH_3), 20.9 (2-COCH_3) ppm.

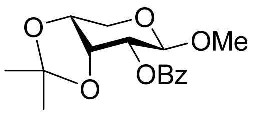

Methyl 2-*O*-benzoyl-3,4-*O*-isopropylidene-β-D-ribopyranoside (100): Synthesized from **98** (50 mg) according to the standard procedure for acylation. The crude product was purified by column chromatography (hexane:EtOAc 3:1) to provide **100** as a clear oil. Yield: 60 mg (74%), R_f = 0.35 (hexane:EtOAc 3:1). ^{1}H NMR (500.20 MHz, $CDCl_3$, 25 °C): δ = 8.14 – 8.09 (m, 2 H, aromatic H), 7.60 – 7.55 (m, 1 H, aromatic H), 7.49 – 7.43 (m, 2 H, aromatic H), 5.13 (dd, 1 H, $J_{H\text{-}1,H\text{-}2}$ = 6.2 Hz, $J_{H\text{-}2,H\text{-}3}$ = 3.3 Hz, H-2), 4.91 (d, 1 H, H-1), 4.69 (dd, 1 H, $J_{H\text{-}3,H\text{-}4}$ = 7.2 Hz, H-3), 4.36 (ddd, 1 H, $J_{H\text{-}4,H\text{-}5a}$ = 2.6 Hz, $J_{H\text{-}4,H\text{-}5b}$ = 1.8 Hz, H-4), 3.87 (dd, 1 H, $J_{H\text{-}5a,H\text{-}5b}$ = – 13.1 Hz, H-5a), 3.74 (dd, 1 H, H-5b), 3.44 (s, 3 H, 1-OC*H*$_3$), 1.51 (s, 3 H, 3,4-OC*(CH$_3$)$_2$*), 1.32 (s, 3 H, 3,4-OC*(CH$_3$)$_2$*) ppm. ^{13}C NMR (125.78 MHz, $CDCl_3$, 25 °C): δ = 166.0 (2-*C*OPh), 133.4, 130.2, 129.8, 128.5 (aromatic C), 110.4 (3,4-O*C*(CH$_3$)$_2$), 99.0 (C-1), 73.7 (C-4), 71.9 (C-3), 70.9 (C-2), 62.6 (C-5), 55.8 (1-O*C*H$_3$), 26.5 (3,4-OC*(CH$_3$)$_2$*), 25.3 (3,4-OC*(CH$_3$)$_2$*) ppm.

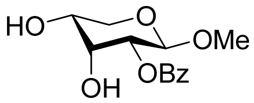

Methyl 2-*O*-benzoyl-β-D ribopyranoside (37): Synthesized from **100** (10 mg) according to the standard reaction procedure D to provide **37** as a clear oil. Yield: 8.4 mg (97%). ^{1}H NMR (500.20 MHz, MeOD, 25 °C): δ = 8.12 – 8.08 (m, 2 H, aromatic H), 7.63 – 7.59 (m, 1 H, aromatic H), 7.50 – 7.46 (m, 2 H, aromatic H), 4.95 (dd, 1 H, $J_{H\text{-}1,H\text{-}2}$ = 5.3 Hz, $J_{H\text{-}2,H\text{-}3}$ = 3.5 Hz, H-2), 4.79 (d, 1 H, H-1), 4.14 (dd, 1 H, $J_{H\text{-}3,H\text{-}4}$ = 2.6 Hz, H-3), 3.83 (ddd, 1 H, $J_{H\text{-}4,H\text{-}5a}$ = 3.7 Hz, $J_{H\text{-}4,H\text{-}5b}$ = 6.9 Hz, H-4), 3.82 (dd, 1 H, $J_{H\text{-}5a,H\text{-}5b}$ = – 11.7 Hz, H-5a), 3.76 (dd, 1 H, H-5b), 3.43 (s, 3 H, 1-OC*H*$_3$) ppm. ^{13}C NMR (125.78 MHz, MeOD, 25 °C): δ = 167.4 (2-*C*OPh), 134.4, 131.3, 130.9, 129.5 (aromatic C), 100.6 (C-1), 73.6 (C-2), 68.7 (C-4), 68.4 (C-3), 64.6 (C-5), 56.4 (1-O*C*H$_3$) ppm.

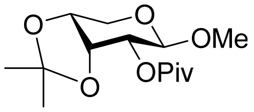

Methyl 3,4-*O*-isopropylidene-2-*O*-pivaoyl-β-D-ribopyranoside (101): Synthesized from **98** (50 mg) according to the standard procedure for acylation. The crude product was purified by column chromatography (hexane:EtOAc 3:1) to provide **101** as a clear oil. Yield: 53 mg (70%) R_f = 0.45 (hexane:EtOAc 3:1). ^{1}H NMR (500.20 MHz, $CDCl_3$, 25 °C): δ = 4.84 (dd, 1 H, $J_{H\text{-}1,H\text{-}2}$ = 6.7 Hz, $J_{H\text{-}2,H\text{-}3}$ = 3.1 Hz, H-2), 4.74 (d, 1 H, H-1), 4.53 (dd, 1 H, $J_{H\text{-}3,H\text{-}4}$ = 7.3 Hz, H-3), 4.30 (ddd, 1 H, $J_{H\text{-}4,H\text{-}5a}$ = 2.7 Hz, $J_{H\text{-}4,H\text{-}5b}$ = 2.2 Hz, H-4), 3.78 (dd, 1 H, $J_{H\text{-}5a,H\text{-}5b}$ = – 12.9 Hz, H-5a), 3.63 (dd, 1 H, H-5b), 3.42 (s, 3 H, 1-OC*H*$_3$), 1.52 (s, 3 H, 3,4-OC*(CH$_3$)$_2$*), 1.31 (s, 3 H, 3,4-OC*(CH$_3$)$_2$*), 1.25 (s, 9 H, 2-OCOC(C*H*$_3$)$_3$) ppm. ^{13}C NMR (125.78 MHz, $CDCl_3$, 25 °C): δ = 178.0 (2-*C*OC(CH$_3$)$_3$), 110.3 (3,4-O*C*(CH$_3$)$_2$), 99.1 (C-1), 73.9 (C-4), 72.1 (C-3), 70.5 (C-2), 63.1 (C-5), 55.9 (1-O*C*H$_3$), 39.0 (2-CO*C*(C*H*$_3$)$_3$), 27.2 (2-COC*(CH$_3$)$_3$*), 26.6 (3,4-OC*(CH$_3$)$_2$*), 25.2 (3,4-OC*(CH$_3$)$_2$*) ppm.

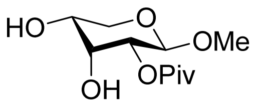

Methyl 2-*O*-pivaoyl-β-D-ribopyranoside (38): Synthesized from **101** (10 mg) according to the standard reaction procedure D to provide **38** as a clear oil. Yield: 6.0 mg (70%). ^{1}H NMR (500.20 MHz, MeOD, 25 °C): δ = 4.67 (dd, 1 H, $J_{H\text{-}1,H\text{-}2}$ = 5.5 Hz, $J_{H\text{-}2,H\text{-}3}$ = 3.4 Hz, H-2), 4.62 (d, 1 H, H-1), 4.02 (dd, 1 H, $J_{H\text{-}3,H\text{-}4}$ = 2.7Hz, H-3), 3.76 (dd, 1 H, $J_{H\text{-}4,H\text{-}5a}$ = 3.7 Hz, $J_{H\text{-}5a,H\text{-}5b}$ = – 11.4 Hz, H-5a), 3.74 (ddd, 1 H, $J_{H\text{-}4,H\text{-}5b}$ = 7.1 Hz, H-4), 3.69 (dd, 1 H, H-5b), 3.41 (s, 3 H, 1-OC*H*$_3$), 1.22 (s, 9 H, 2-OCOC(C*H*$_3$)$_3$) ppm. ^{13}C NMR (125.78 MHz, MeOD, 25 °C): δ = 179.3 (2-*C*OC(CH$_3$)$_3$), 100.7 (C-1), 73.0 (C-2), 68.8 (C-4), 68.4 (C-3), 64.5 (C-5), 56.5 (1-O*C*H$_3$), 39.9 (2-CO*C*(C*H*$_3$)$_3$), 27.5 (2-COC*(CH$_3$)$_3$*) ppm.

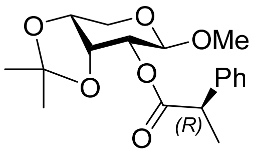

Methyl 3,4-*O*-isopropylidene-2-*O*-(*R*)-2-phenyl-propanoyl-β-D-ribopyranoside (102): Synthesized from **98** (40 mg) according to the standard reaction procedure B. The crude product was purified by column chromatography (hexane:EtOAc 3:1) to give **102** as a clear oil. Yield: 46 mg

(65%), R_f = 0.33 (hexane:EtOAc 3:1). ^{1}H NMR (500.20 MHz, $CDCl_3$, 25 °C): δ = 7.34 – 7.22 (m, 5 H, aromatic H), 4.91 (dd, 1 H, $J_{H\text{-}1,H\text{-}2}$ = 6.5 Hz, $J_{H\text{-}2,H\text{-}3}$ = 3.2 Hz, H-2), 4.55 (dd, 1 H, $J_{H\text{-}3,H\text{-}4}$ = 7.4 Hz, H-3), 4.54 (d, 1 H, H-1), 4.30 (ddd, 1 H, $J_{H\text{-}4,H\text{-}5a}$ = 2.5 Hz, $J_{H\text{-}4,H\text{-}5b}$ = 1.8 Hz, H-4), 3.86 (ddd, 1 H, $J_{2\text{-CO}\underline{\text{CH}}\text{CH3Ph, 2-COCH}\underline{\text{CH3}}\text{Ph}}$ = 7.2 Hz, 2-CO*C**H*CH_3Ph), 3.75 (dd, 1 H, $J_{H\text{-}5a,H\text{-}5b}$ = – 13.0 Hz, H-5a), 3.63 (dd, 1 H, H-5b), 3.16 (s, 3 H, 1-OCH_3), 1.53 (s, 3 H, 3,4-OC$(CH_3)_2$), 1.52 (d, 3 H, 2-COCHCH_3Ph), 1.34 (s, 3 H, 3,4-OC$(CH_3)_2$) ppm. ^{13}C NMR (125.78 MHz, $CDCl_3$, 25 °C): δ = 174.0 (2-*C*OCHCH_3Ph), 140.4, 128.7, 127.7, 127.2 (aromatic C), 110.4 (3,4-O*C*$(CH_3)_2$), 99.0 (C-1), 73.9 (C-4), 72.1 (C-3), 70.5 (C-2), 62.7 (C-5), 55.8 (1-OCH_3), 45.4 (2-CO*CH*CH_3Ph), 26.5 (3,4-OC$(CH_3)_2$), 26.3 (3,4-OC$(CH_3)_2$), 18.6 (2-COCHCH_3Ph) ppm.

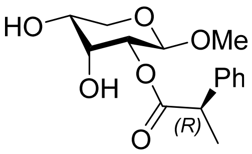

Methyl 2-*O*-(*R*)-2-phenyl-propanoyl-β-D-ribopyranoside (39): Synthesized from **102** (10 mg) according to the standard reaction procedure D to provide **39** as a clear oil. Yield: 8.5 mg (96%). ^{1}H NMR (500.20 MHz, MeOD, 25 °C): δ = 7.36 – 7.21 (m, 5 H, aromatic H), 4.69 (dd, 1 H, $J_{H\text{-}1,H\text{-}2}$ = 5.6 Hz, $J_{H\text{-}2,H\text{-}3}$ = 3.4 Hz, H-2), 4.45 (d, 1 H, H-1), 4.02 (dd, 1 H, $J_{H\text{-}3,H\text{-}4}$ = 2.5 Hz, H-3), 3.85 (ddd, 1 H, $J_{2\text{-CO}\underline{\text{CH}}\text{CH3Ph, 2-COCH}\underline{\text{CH3}}\text{Ph}}$ = 7.1 Hz, 2-CO*CH*CH_3Ph), 3.71 (ddd, 1 H, $J_{H\text{-}4,H\text{-}5a}$ = 3.6 Hz, $J_{H\text{-}4,H\text{-}5b}$ = 7.7 Hz, H-4), 3.70 (dd, 1 H, $J_{H\text{-}5a,H\text{-}5b}$ = – 12.0 Hz, H-5a), 3.62 (dd, 1 H, H-5b), 3.21 (s, 3 H, 1-OCH_3), 1.47 (d, 3 H, 2-COCHCH_3Ph) ppm. ^{13}C NMR (125.78 MHz, MeOD, 25 °C): δ = 175.3 (2-*C*OCHCH_3Ph), 142.0, 129.6, 128.7, 128.1 (aromatic C), 100.6 (C-1), 73.3 (C-2), 68.8 (C-4), 68.6 (C-3), 64.5 (C-5), 56.5 (1-OCH_3), 46.5 (2-CO*CH*CH_3Ph), 18.8 (2-COCHCH_3Ph) ppm.

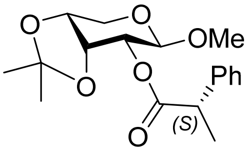

Methyl 3,4-*O*-isopropylidene-2-*O*-(*S*)-2-phenyl-propanoyl-β-D-ribopyranoside (103): Synthesized from **98** (50 mg) according to the standard reaction procedure B. The crude product was purified by column chromatography (hexane:EtOAc 3:1) to give **103** as a clear oil. Yield: 68 mg (76%), R_f = 0.33 (hexane:EtOAc 3:1). ^{1}H NMR (500.20 MHz, $CDCl_3$, 25 °C): δ = 7.35 – 7.23 (m, 5 H, aromatic H), 4.90 (dd, 1 H, $J_{H\text{-}1,H\text{-}2}$ = 3.4 Hz, $J_{H\text{-}2,H\text{-}3}$ = 8.0 Hz, H-2), 4.74 (d, 1 H, H-1), 4.44 (dd, 1 H, $J_{H\text{-}3,H\text{-}4}$ = 5.5 Hz, H-3), 4.26 (ddd, 1 H, $J_{H\text{-}4,H\text{-}5a}$ = 0.9 Hz, $J_{H\text{-}4,H\text{-}5b}$ = 2.8 Hz, H-4), 3.86 (ddd, 1 H, $J_{2\text{-CO}\underline{\text{CH}}\text{CH3Ph, 2-COCH}\underline{\text{CH3}}\text{Ph}}$ = 7.2 Hz, 2-CO*H*CH_3Ph), 3.78 (dd, 1 H, $J_{H\text{-}5a,H\text{-}5b}$ = – 13.4 Hz, H-5a), 3.63 (dd, 1 H, H-5b), 3.41 (s, 3 H, 1-OCH_3), 1.54 (d, 3 H, 2-COCHCH_3Ph), 1.45 (s, 3 H, 3,4-OC$(CH_3)_2$), 1.23 (s, 3 H, 3,4-OC$(CH_3)_2$) ppm. ^{13}C NMR (125.78 MHz, $CDCl_3$, 25 °C): δ = 174.2 (2-*C*OCHCH_3Ph), 140.1, 128.6, 127.7, 127.3 (aromatic C), 110.2 (3,4-O*C*$(CH_3)_2$), 98.9 (C-1), 73.5 (C-4), 71.8 (C-3), 70.5 (C-2), 62.8 (C-5), 55.8 (1-OCH_3), 45.4 (2-CO*CH*CH_3Ph), 26.5 (3,4-OC$(CH_3)_2$), 25.2 (3,4-OC$(CH_3)_2$), 18.9 (2-COCHCH_3Ph) ppm.

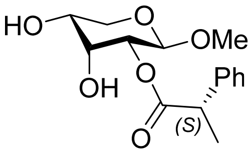

Methyl 2-*O*-(*S*)-2-phenyl-propanoyl-β-D-ribopyranoside (40): Synthesized from **103** (10 mg) according to the standard reaction procedure D to provide **40** as a white solid. Yield: 8.5 mg (96%). ^{1}H NMR (500.20 MHz, MeOD, 25 °C): δ = 7.36 – 7.21 (m, 5 H, aromatic H), 4.72 (dd, 1 H, $J_{H\text{-}1,H\text{-}2}$ = 5.2 Hz, $J_{H\text{-}2,H\text{-}3}$ = 3.1 Hz, H-2), 4.63 (d, 1 H, H-1), 3.94 (dd, 1 H, $J_{H\text{-}3,H\text{-}4}$ = 3.6 Hz, H-3), 3.84 (ddd, 1 H, $J_{2\text{-CO}\underline{\text{CH}}\text{CH3Ph, 2-COCH}\underline{\text{CH3}}\text{Ph}}$ = 7.2 Hz, 2-CO*CH*CH_3Ph), 3.74 (dd, 1 H, $J_{H\text{-}4,H\text{-}5a}$ = 3.5 Hz, $J_{H\text{-}5a,H\text{-}5b}$ = – 11.6 Hz, H-5a), 3.69 (ddd, 1 H, $J_{H\text{-}4,H\text{-}5b}$ = 6.9 Hz, H-4), 3.66 (dd, 1 H, H-5b), 3.38 (s, 3 H, 1-OCH_3), 1.50 (d, 3 H, 2-COCHCH_3Ph) ppm. ^{13}C NMR (125.78 MHz, MeOD, 25 °C): δ = 175.4 (2-*C*OCHCH_3Ph), 141.9, 129.6, 128.7, 128.2 (aromatic C), 100.5 (C-1), 73.2 (C-2), 68.7 (C-4), 68.1 (C-3), 64.4 (C-5), 56.4 (1-OCH_3), 46.8 (2-CO*CH*CH_3Ph), 19.1 (2-COCHCH_3Ph) ppm.

Arabinopyranoside derivatives

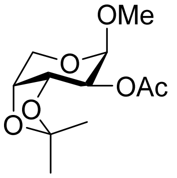

Methyl 2-*O*-acetyl-3,4-*O*-isopropylidene-β-D-arabinopyranoside (105): Synthesized from **104** (50 mg) according to the standard procedure for acylation. The crude product was purified by column chromatography (hexane:EtOAc 3:1) to provide **105** as a clear oil. Yield: 48 mg (74%), R_f = 0.22 (hexane:EtOAc 3:1). ^{1}H NMR (500.20 MHz, $CDCl_3$, 25 °C): δ = 4.92 (dd, 1 H, $J_{H\text{-}1,H\text{-}2}$ = 3.4 Hz, $J_{H\text{-}2,H\text{-}3}$ = 8.3 Hz, H-2), 4.82 (d, 1 H, H-1), 4.31 (dd, 1 H, $J_{H\text{-}3,H\text{-}4}$ = 5.5 Hz, H-3), 4.26 (ddd, 1 H, $J_{H\text{-}4,H\text{-}5a}$ = 0.9 Hz, $J_{H\text{-}4,H\text{-}5b}$ = 2.9 Hz, H-4), 4.01 (dd, 1 H, $J_{H\text{-}5a,H\text{-}5b}$ = – 13.4 Hz, H-5a), 3.93 (dd, 1 H, H-5b), 3.39 (s, 3 H, 1-OCH_3), 2.14 (s, 3 H, 2-OCOCH_3), 1.55 (s, 3 H, 3,4-OC$(CH_3)_2$), 1.37 (s, 3 H, 3,4-OC$(CH_3)_2$) ppm. ^{13}C NMR (125.78 MHz, $CDCl_3$, 25 °C): δ = 170.7 (2-*C*OCH_3), 109.6 (3,4-O*C*$(CH_3)_2$), 97.4 (C-1), 73.7 (C-4), 73.1 (C-3), 72.4 (C-2), 58.5 (C-5), 55.8 (1-O*C*H_3), 28.1 (3,4-OC$(CH_3)_2$), 26.5 (3,4-OC$(CH_3)_2$), 21.2 (2-CO*C*H_3), 16.6 (C-6) ppm.

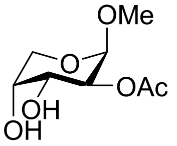

Methyl 2-*O*-acetyl-β-D-arabinopyranoside (41): Synthesized from **105** (10 mg) according to the standard reaction procedure D to provide **41** as a white solid. Yield: 5 mg (60%). ^{1}H NMR (500.20 MHz, MeOD, 25 °C): δ = 4.98 (dd, 1 H, $J_{H\text{-}1,H\text{-}2}$ = 3.6 Hz, $J_{H\text{-}2,H\text{-}3}$ = 10.1 Hz, H-2), 4.84 (d, 1 H, H-1), 3.93 (dd, 1 H, $J_{H\text{-}3,H\text{-}4}$ = 3.5 Hz, H-3), 3.89 (ddd, 1 H, $J_{H\text{-}4,H\text{-}5a}$ = 1.6 Hz, $J_{H\text{-}4,H\text{-}5b}$ = 2.3 Hz, H-4), 3.81 (dd, 1 H, $J_{H\text{-}5a,H\text{-}5b}$ = – 12.4 Hz, H-5a), 3.60 (dd, 1 H, H-5b), 3.34 (s, 3 H, 1-OCH_3), 2.07 (s, 3 H, 2-OCOCH_3) ppm. ^{13}C NMR (125.78 MHz, MeOD, 25 °C): δ = 172.6 (2-*C*OCH_3), 99.2 (C-1), 72.8 (C-2), 70.8 (C-4), 68.2 (C-3), 63.8 (C-5), 55.6 (1-O*C*H_3), 20.8 (2-CO*C*H_3), 16.6 (C-6) ppm.

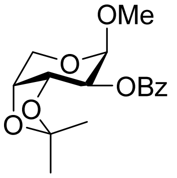

Methyl 2-*O*-benzoyl-3,4-*O*-isopropylidene-β-D-arabinopyranoside (106): Synthesized from **104** (50 mg) according to the standard procedure for acylation. The crude product was purified by column chromatography (hexane:EtOAc 3:1) to provide **106** as a clear oil. Yield: 75 mg (92%), R_f = 0.37 (hexane:EtOAc 3:1). ^{1}H NMR (500.20 MHz, $CDCl_3$, 25 °C): δ = 8.12 – 8.08 (m, 2 H, aromatic H), 7.59 – 7.55 (m, 1 H, aromatic H), 7.47 – 7.42 (m, 2 H, aromatic H), 5.16 (dd, 1 H, $J_{H\text{-}1,H\text{-}2}$ = 3.4 Hz, $J_{H\text{-}2,H\text{-}3}$ = 8.2 Hz, H-2), 4.95 (d, 1 H, H-1), 4.50 (dd, 1 H, $J_{H\text{-}3,H\text{-}4}$ = 5.5 Hz, H-3), 4.31 (ddd, 1 H, $J_{H\text{-}4,H\text{-}5a}$ = 0.8 Hz, $J_{H\text{-}4,H\text{-}5b}$ = 2.9 Hz, H-4), 4.06 (dd, 1 H, $J_{H\text{-}5a,H\text{-}5b}$ = – 13.4 Hz, H-5a), 3.99 (dd, 1 H, H-5b), 3.39 (s, 3 H, 1-OCH_3), 1.58 (s, 3 H, 3,4-OC$(CH_3)_2$), 1.38 (s, 3 H, 3,4-OC$(CH_3)_2$) ppm. ^{13}C NMR (125.78 MHz, $CDCl_3$, 25 °C): δ = 166.9 (2-*C*OPh), 133.3, 130.1, 129.9, 128.5 (aromatic C), 109.6 (3,4-O*C*$(CH_3)_2$), 97.5 (C-1), 73.8 (C-4), 73.2 (C-3), 72.9 (C-2), 58.6 (C-5), 55.9 (1-O*C*H_3), 28.2 (3,4-OC$(CH_3)_2$), 26.5 (3,4-OC$(CH_3)_2$) ppm.

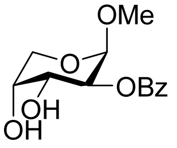

Methyl 2-*O*-benzoyl-β-D-arabinopyranoside (42): Synthesized from **106** (10 mg) according to the standard reaction procedure D to provide **42** as a white solid. Yield: 8.0 mg (92%). ^{1}H NMR (500.20 MHz, MeOD, 25 °C): δ = 8.08 – 8.05 (m, 2 H, aromatic H), 7.63 – 7.59 (m, 1 H, aromatic H), 7.50 – 7.46 (m, 2 H, aromatic H), 5.22 (dd, 1 H, $J_{H\text{-}1,H\text{-}2}$ = 3.6 Hz, $J_{H\text{-}2,H\text{-}3}$ = 10.1 Hz, H-2), 4.99 (d, 1 H, H-1), 4.11 (dd, 1 H, $J_{H\text{-}3,H\text{-}4}$ = 3.4 Hz, H-3), 3.96 (ddd, 1 H, $J_{H\text{-}4,H\text{-}5a}$ = 1.6 Hz, $J_{H\text{-}4,H\text{-}5b}$ = 2.3 Hz, H-4), 3.88 (dd, 1 H, $J_{H\text{-}5a,H\text{-}5b}$ = – 12.4 Hz, H-5a), 3.66 (dd, 1 H, H-5b), 3.36 (s, 3 H, 1-OCH_3) ppm. ^{13}C NMR (125.78 MHz, MeOD, 25 °C): δ = 167.9 (2-*C*OPh), 134.3, 131.3, 130.8, 129.5 (aromatic C), 99.3 (C-1), 73.4 (C-2), 71.0 (C-4), 68.3 (C-3), 63.9 (C-5), 55.8 (1-O*C*H_3) ppm.

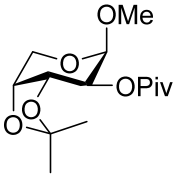

Methyl 3,4-*O*-isopropylidene-2-*O*-pivaoyl-β-D-arabinopyranoside (107): Synthesized from **104** (50 mg) according to the standard procedure for acylation. The crude product was purified by column chromatography (hexane:EtOAc 4:1) to provide **107** as a clear oil. Yield: 73 mg (96%), R_f = 0.32 (hexane:EtOAc 4:1). ^{1}H NMR (500.20 MHz, $CDCl_3$, 25 °C): δ = 4.87 (dd, 1 H, $J_{H-1,H-2}$ = 3.5 Hz, $J_{H-2,H-3}$ = 8.0 Hz, H-2), 4.79 (d, 1 H, H-1), 4.31 (dd, 1 H, $J_{H-3,H-4}$ = 5.5 Hz, H-3), 4.24 (ddd, 1 H, $J_{H-4,H-5a}$ = 0.8 Hz, $J_{H-4,H-5b}$ = 2.8 Hz, H-4), 3.99 (dd, 1 H, $J_{H-5a,H-5b}$ = – 13.4 Hz, H-5a), 3.94 (dd, 1 H, H-5b), 3.35 (s, 3 H, 1-OC*H*$_3$), 1.54 (s, 3 H, 3,4-OC*(CH$_3$)$_2$*), 1.36 (s, 3 H, 3,4-OC*(CH$_3$)$_2$*), 1.24 (s, 9 H, 2-OCOC(C*H*$_3$)$_3$) ppm. ^{13}C NMR (125.78 MHz, $CDCl_3$, 25 °C): δ = 178.3 (4-*C*OC(C*H*$_3$)$_3$), 109.5 (3,4-O*C*(CH_3)$_2$), 97.4 (C-1), 73.6 (C-4), 73.1 (C-3), 72.0 (C-2), 58.6 (C-5), 55.9 (1-O*C*H_3), 38.9 (4-CO*C*(C*H*$_3$)$_3$), 28.1 (3,4-OC*(CH$_3$)$_2$*), 27.2 (4-COC*(CH$_3$)$_3$*), 26.5 (3,4-OC*(CH$_3$)$_2$*) ppm.

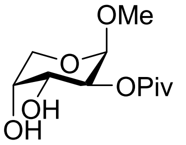

Methyl 2-*O*-pivaoyl-β-D-arabinopyranoside (43): Synthesized from **107** (10 mg) according to the standard reaction procedure D to provide **43** as a white solid. Yield: 8.5 mg (98%). ^{1}H NMR (500.20 MHz, MeOD, 25 °C): δ = 4.93 (dd, 1 H, $J_{H-1,H-2}$ = 3.6 Hz, $J_{H-2,H-3}$ = 10.1 Hz, H-2), 4.82 (d, 1 H, H-1), 3.94 (dd, 1 H, $J_{H-3,H-4}$ = 3.5 Hz, H-3), 3.88 (ddd, 1 H, $J_{H-4,H-5a}$ = 1.3 Hz, $J_{H-4,H-5b}$ = 2.3 Hz, H-4), 3.82 (dd, 1 H, $J_{H-5a,H-5b}$ = – 12.3 Hz, H-5a), 3.61 (dd, 1 H, H-5b), 3.34 (s, 3 H, 1-OC*H*$_3$), 1.21 (s, 9 H, 2-OCOC(C*H*$_3$)$_3$) ppm. ^{13}C NMR (125.78 MHz, MeOD, 25 °C): δ = 179.9 (4-*C*OC(C*H*$_3$)$_3$), 99.2 (C-1), 72.6 (C-2), 71.0 (C-4), 68.2 (C-3), 63.9 (C-5), 55.8 (1-O*C*H_3), 39.8 (4-CO*C*(C*H*$_3$)$_3$), 27.5 (4-COC*(CH$_3$)$_3$*) ppm.

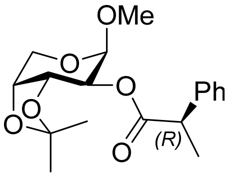

Methyl 3,4-*O*-isopropylidene-2-*O*-(*R*)-2-phenyl-propanoyl-β-D-arabinopyranoside (108): Synthesized from **104** (50 mg) according to the standard reaction procedure B. The crude product was purified by column chromatography (hexane:EtOAc 4:1) to give **108** as a clear oil. Yield: 62 mg (76%), R_f = 0.26 (hexane:EtOAc 4:1). ^{1}H NMR (500.20 MHz, $CDCl_3$, 25 °C): δ = 7.34 – 7.22 (m, 5 H, aromatic H), 4.91 (dd, 1 H, $J_{H-1,H-2}$ = 3.4 Hz, $J_{H-2,H-3}$ = 8.0 Hz, H-2), 4.79 (d, 1 H, H-1), 4.26 (dd, 1 H, $J_{H-3,H-4}$ = 5.5 Hz, H-3), 4.19 (ddd, 1 H, $J_{H-4,H-5a}$ = 0.9 Hz, $J_{H-4,H-5b}$ = 2.8 Hz, H-4), 3.97 (dd, 1 H, $J_{H-5a,H-5b}$ = – 13.4 Hz, H-5a), 3.91 (dd, 1 H, H-5b), 3.82 (ddd, 1 H, $J_{2-CO\underline{CH}CH3Ph, 2-COCH\underline{CH3}Ph}$ = 7.2 Hz, 2-COC*H*CH_3Ph), 3.35 (s, 3 H, 1-OC*H*$_3$), 1.54 (d, 3 H, 2-COCHC*H*$_3$Ph), 1.49 (s, 3 H, 3,4-OC*(CH$_3$)$_2$*), 1.33 (s, 3 H, 3,4-OC*(CH$_3$)$_2$*) ppm. ^{13}C NMR (125.78 MHz, $CDCl_3$, 25 °C): δ = 174.3 (2-*C*OCHCH_3Ph), 140.4, 128.7, 128.6, 127.7, 127.2 (aromatic C), 109.5 (3,4-O*C*(CH_3)$_2$), 97.3 (C-1), 73.6 (C-4), 72.9 (C-3), 72.4 (C-2), 58.7 (C-5), 55.8 (1-O*C*H_3), 45.7 (2-CO*CH*CH_3Ph), 28.1 (3,4-OC*(CH$_3$)$_2$*), 26.5 (3,4-OC*(CH$_3$)$_2$*), 18.9 (2-COCH*CH$_3$*Ph) ppm.

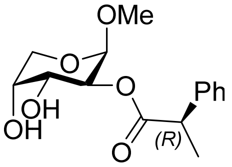

Methyl 2-*O*-(*R*)-2-phenyl-propanoyl-β-D-arabinopyranoside (44): Synthesized from **108** (14 mg) according to the standard reaction procedure D to provide **44** as a white solid. Yield: 12 mg (12%). ^{1}H NMR (500.20 MHz, MeOD, 25 °C): δ = 7.36 – 7.20 (m, 5 H, aromatic H), 4.96 (dd, 1 H, $J_{H-1,H-2}$ = 3.5 Hz, $J_{H-2,H-3}$ = 9.9 Hz, H-2), 4.82 (d, 1 H, H-1), 3.91 (dd, 1 H, $J_{H-3,H-4}$ = 3.5 Hz, H-3), 3.84 (ddd, 1 H, $J_{H-4,H-5a}$ = 1.6 Hz, $J_{H-4,H-5b}$ = 2.5 Hz, H-4), 3.799 (ddd, 1 H, $J_{2-CO\underline{CH}CH3Ph, 2-COCH\underline{CH3}Ph}$ = 7.2 Hz, 2-COC*H*CH_3Ph), 3.799 (dd, 1 H, $J_{H-5a,H-5b}$ = – 12.3 Hz, H-5a), 3.59 (dd, 1 H, H-5b), 3.32 (s, 3 H, 1-OC*H*$_3$), 1.49 (d, 3 H, 2-COCHC*H*$_3$Ph) ppm. ^{13}C NMR (125.78 MHz, MeOD, 25 °C): δ = 176.0 (2-*C*OCHCH_3Ph), 141.9, 129.6, 129.5, 128.7, 128.1 (aromatic C), 99.1 (C-1), 73.1 (C-2), 70.8 (C-4), 68.1 (C-3), 63.9 (C-5), 55.8 (1-O*C*H_3), 46.9 (2-CO*CH*CH_3Ph), 19.2 (2-COCH*CH$_3$*Ph) ppm.

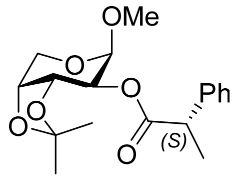

Methyl 3,4-*O*-isopropylidene-2-*O*-(*S*)-2-phenyl-propanoyl-β-D-arabinopyranoside (109): Synthesized from **104** (50 mg) according to the standard reaction procedure B. The crude product was purified by column chromatography (hexane:EtOAc 4:1) to give **109** as a white solid. Yield: 78 mg (88%), R_f = 0.27 (hexane:EtOAc 4:1). ^{1}H NMR (500.20 MHz, $CDCl_3$, 25 °C): δ = 7.34 – 7.22 (m, 5 H, aromatic H), 4.88 (dd, 1 H, $J_{H\text{-}1,H\text{-}2}$ = 3.4 Hz, $J_{H\text{-}2,H\text{-}3}$ = 8.0 Hz, H-2), 4.60 (d, 1 H, H-1), 4.29 (dd, 1 H, $J_{H\text{-}3,H\text{-}4}$ = 5.5 Hz, H-3), 4.21 (ddd, 1 H, $J_{H\text{-}4,H\text{-}5a}$ = 0.9 Hz, $J_{H\text{-}4,H\text{-}5b}$ = 2.8 Hz, H-4), 3.96 (dd, 1 H, $J_{H\text{-}5a,H\text{-}5b}$ = – 13.4 Hz, H-5a), 3.87 (dd, 1 H, H-5b), 3.82 (ddd, 1 H, $J_{2\text{-}CO\underline{CH}CH3Ph,\ 2\text{-}COCH\underline{CH3}Ph}$ = 7.2 Hz, 2-CO*CH*CH_3Ph), 3.09 (s, 3 H, 1-OCH_3), 1.56 (s, 3 H, 3,4-OC$(CH_3)_2$), 1.51 (d, 3 H, 2-COCHCH_3Ph), 1.36 (s, 3 H, 3,4-OC$(CH_3)_2$) ppm. ^{13}C NMR (125.78 MHz, $CDCl_3$, 25 °C): δ = 174.4 (2-*C*OCHCH_3Ph), 140.6, 128.7, 127.6, 127.2 (aromatic C), 109.6 (3,4-O*C*$(CH_3)_2$), 97.1 (C-1), 73.7 (C-4), 73.0 (C-3), 72.6 (C-2), 58.5 (C-5), 55.7 (1-OCH_3), 45.3 (2-CO*CH*CH_3Ph), 28.2 (3,4-OC$(CH_3)_2$), 26.5 (3,4-OC$(CH_3)_2$), 18.3 (2-COCHCH_3Ph) ppm.

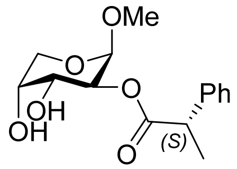

Methyl 2-*O*-(*S*)-2-phenyl-propanoyl-β-D-arabinopyranoside (45): Synthesized from **109** (10 mg) according to the standard reaction procedure D to provide **45** as a white solid. Yield: 8.8 mg (99%). ^{1}H NMR (500.20 MHz, MeOD, 25 °C): δ = 7.33 – 7.20 (m, 5 H, aromatic H), 4.98 (dd, 1 H, $J_{H\text{-}1,H\text{-}2}$ = 3.6 Hz, $J_{H\text{-}2,H\text{-}3}$ = 10.2 Hz, H-2), 4.65 (d, 1 H, H-1), 3.91 (dd, 1 H, $J_{H\text{-}3,H\text{-}4}$ = 3.5 Hz, H-3), 3.87 (ddd, 1 H, $J_{H\text{-}4,H\text{-}5a}$ = 1.4 Hz, $J_{H\text{-}4,H\text{-}5b}$ = 2.2 Hz, H-4), 3.82 (ddd, 1 H, $J_{2\text{-}CO\underline{CH}CH3Ph,\ 2\text{-}COCH\underline{CH3}Ph}$ = 7.1 Hz, 2-CO*CH*CH_3Ph), 3.76 (dd, 1 H, $J_{H\text{-}5a,H\text{-}5b}$ = – 12.4 Hz, H-5a), 3.56 (dd, 1 H, H-5b), 3.08 (s, 3 H, 1-OCH_3), 1.46 (d, 3 H, 2-COCHCH_3Ph) ppm. ^{13}C NMR (125.78 MHz, MeOD, 25 °C): δ = 176.1 (2-*C*OCHCH_3Ph), 142.1, 129.6, 128.5, 128.1 (aromatic C), 99.0 (C-1), 73.0 (C-2), 70.9 (C-4), 67.0 (C-3), 63.8 (C-5), 55.8 (1-OCH_3), 46.4 (2-CO*CH*CH_3Ph), 18.8 (2-COCHCH_3Ph) ppm.

Rhamnopyranoside derivatives

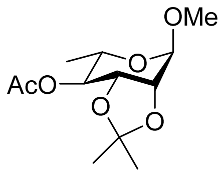

Methyl 4-*O*-acetyl-2,3-*O*-isopropylidene-α-L-rhamnopyranoside (111): Synthesized from **110** (50 mg) according to the standard procedure for acylation. The crude product was purified by column chromatography (hexane:EtOAc 3:1) to provide **111** as a white solid. Yield: 52 mg (87%), R_f = 0.38 (hexane:EtOAc 3:1). ^{1}H NMR (500.20 MHz, $CDCl_3$, 25 °C): δ = 4.89 (d, 1 H, $J_{H\text{-}1,H\text{-}2}$ = 0.6 Hz, H-1), 4.86 (dd, 1 H, $J_{H\text{-}3,H\text{-}4}$ = 7.9 Hz, $J_{H\text{-}4,H\text{-}5}$ = 10.1 Hz, H-4), 4.15 (dd, 1 H, $J_{H\text{-}2,H\text{-}3}$ = 5.5 Hz, H-3), 4.13 (dd, 1 H, H-2), 3.70 (dddd, 1 H, $J_{H\text{-}5,H\text{-}6}$ = 6.3 Hz, H-5), 3.38 (s, 3 H, 1-OCH_3), 2.09 (s, 3 H, 4-OCOCH_3), 1.57 (s, 3 H, 2,3-OC$(CH_3)_2$), 1.34 (s, 3 H, 2,3-OC$(CH_3)_2$), 1.17 (d, 3 H, H-6) ppm. ^{13}C NMR (125.78 MHz, $CDCl_3$, 25 °C): δ = 170.3 (4-*C*OCH_3), 109.9 (2,3-O*C*$(CH_3)_2$), 98.2 (C-1), 76.1 (C-2), 76.0 (C-3), 74.6 (C-4), 64.0 (C-5), 55.4 (1-OCH_3), 27.8 (2,3-OC$(CH_3)_2$), 26.5 (2,3-OC$(CH_3)_2$), 21.2 (4-COCH_3), 17.1 (C-6) ppm.

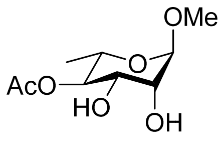

Methyl 4-*O*-acetyl-α-L-rhamnopyranoside (46): Synthesized from **111** (10 mg) according to the standard reaction procedure D to provide **46** as a white solid. Yield: 3.2 mg (44%). ^{1}H NMR (500.20 MHz, MeOD, 25 °C): δ = 4.92 (dd, 1 H, $J_{H\text{-}3,H\text{-}4}$ = 9.8 Hz, $J_{H\text{-}4,H\text{-}5}$ = 9.8 Hz, H-4), 4.59 (d, 1 H, $J_{H\text{-}1,H\text{-}2}$ = 0.6 Hz, H-1), 3.81 (dd, 1 H, $J_{H\text{-}2,H\text{-}3}$ = 3.3 Hz, H-2), 3.76 (dd, 1 H, H-3), 3.68 (dddd, 1 H, $J_{H\text{-}5,H\text{-}6}$ = 6.3 Hz, H-5), 3.36 (s, 3 H, 1-OCH_3), 2.08 (s, 3 H, 4-OCOCH_3), 1.15 (d, 3 H, H-6) ppm. ^{13}C NMR (125.78 MHz, MeOD, 25 °C): δ = 172.5 (4-*C*OCH_3), 102.7 (C-1), 75.5 (C-4), 72.2 (C-2), 70.4 (C-3), 67.5 (C-5), 55.2 (1-OCH_3), 21.0 (4-COCH_3), 17.8 (C-6) ppm.

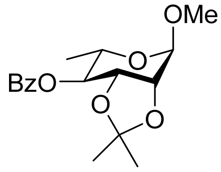

Methyl 4-*O*-benzoyl-2,3-*O*-isopropylidene-α-L-rhamnopyranoside (112): Synthesized from **110** (50 mg) according to the standard procedure for acylation. The crude product was purified by column chromatography (hexane:EtOAc 7:1) to provide **112** as a clear oil. Yield: 65 mg (88%), R_f = 0.26 (hexane:EtOAc 7:1). ^{1}H NMR (500.20 MHz, $CDCl_3$, 25 °C): δ = 8.08 – 8.03 (m, 2 H, aromatic H), 7.59 – 7.53 (m, 1 H, aromatic H), 7.46 – 7.41 (m, 2 H, aromatic H), 5.12 (dd, 1 H, $J_{H\text{-}3,H\text{-}4}$ = 7.8 Hz, $J_{H\text{-}4,H\text{-}5}$ = 10.1 Hz, H-4), 4.95 (d, 1 H, $J_{H\text{-}1,H\text{-}2}$ = 0.6 Hz, H-1), 4.33 (dd, 1 H, $J_{H\text{-}2,H\text{-}3}$ = 5.4 Hz, H-3), 4.19 (dd, 1 H, H-2), 3.86 (dddd, 1 H, $J_{H\text{-}5,H\text{-}6}$ = 6.3 Hz, H-5), 3.42 (s, 3 H, 1-OC*H*$_3$), 1.62 (s, 3 H, 2,3-OC*(CH$_3$)$_2$*), 1.35 (s, 3 H, 2,3-OC*(CH$_3$)$_2$*), 1.23 (d, 3 H, H-6) ppm. ^{13}C NMR (125.78 MHz, $CDCl_3$, 25 °C): δ = 165.9 (4-*C*OPh), 133.3, 130.0, 129.9, 128.5 (aromatic C), 110.0 (2,3-O*C*(CH_3)$_2$), 98.2 (C-1), 76.1 (C-2), 76.0 (C-3), 75.2 (C-4), 64.2 (C-5), 55.2 (1-O*C*H$_3$), 27.9 (2,3-OC*(CH$_3$)$_2$*), 26.5 (2,3-OC*(CH$_3$)$_2$*), 17.3 (C-6) ppm.

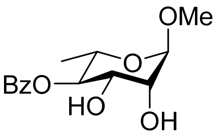

Methyl 4-*O*-benzoyl-α-L-rhamnopyranoside (47): Synthesized from **112** (10 mg) according to the standard reaction procedure D to provide **47** as a white solid. Yield: 4.5 mg (51%). ^{1}H NMR (500.20 MHz, MeOD, 25 °C): δ = 8.07 – 8.03 (m, 2 H, aromatic H), 7.64 – 7.59 (m, 1 H, aromatic H), 7.51 – 7.46 (m, 2 H, aromatic H), 5.18 (dd, 1 H, $J_{H\text{-}3,H\text{-}4}$ = 9.8 Hz, $J_{H\text{-}4,H\text{-}5}$ = 9.7 Hz, H-4), 4.65 (d, 1 H, $J_{H\text{-}1,H\text{-}2}$ = 1.7 Hz, H-1), 3.94 (dd, 1 H, $J_{H\text{-}2,H\text{-}3}$ = 3.4 Hz, H-3), 3.869 (dd, 1 H, H-2), 3.865 (dddd, 1 H, $J_{H\text{-}5,H\text{-}6}$ = 6.3 Hz, H-5), 3.40 (s, 3 H, 1-OC*H*$_3$), 1.19 (d, 3 H, H-6) ppm. ^{13}C NMR (125.78 MHz, MeOD, 25 °C): δ = 166.4 (4-*C*OPh), 132.9, 130.1, 129.3, 128.2 (aromatic C), 101.4 (C-1), 74.7 (C-4), 71.0 (C-2), 69.0 (C-3), 66.2 (C-5), 53.9 (1-O*C*H$_3$), 16.5 (C-6) ppm.

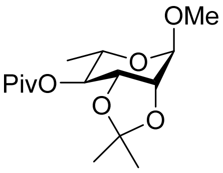

Methyl 2,3-*O*-isopropylidene-4-*O*-pivaoyl-α-L-rhamnopyranoside (113): Synthesized from **110** (50 mg) according to the standard procedure for acylation. The crude product was purified by column chromatography (hexane:EtOAc 7:1) to provide **113** as a clear oil. Yield: 35 mg (51%), R_f = 0.32 (hexane:EtOAc 7:1). ^{1}H NMR (500.20 MHz, $CDCl_3$, 25 °C): δ = 4.89 (d, 1 H, $J_{H\text{-}1,H\text{-}2}$ = 0.6 Hz, H-1), 4.84 (dd, 1 H, $J_{H\text{-}3,H\text{-}4}$ = 7.8 Hz, $J_{H\text{-}4,H\text{-}5}$ = 10.1 Hz, H-4), 4.14 (dd, 1 H, $J_{H\text{-}2,H\text{-}3}$ = 5.5 Hz, H-3), 4.12 (dd, 1 H, H-2), 3.72 (dddd, 1 H, $J_{H\text{-}5,H\text{-}6}$ = 6.3 Hz, H-5), 3.39 (s, 3 H, 1-OC*H*$_3$), 1.56 (s, 3 H, 2,3-OC*(CH$_3$)$_2$*), 1.34 (s, 3 H, 2,3-OC*(CH$_3$)$_2$*), 1.22 (s, 9 H, 4-OCOC(C*H*$_3$)$_3$), 1.16 (d, 3 H, H-6) ppm. ^{13}C NMR (125.78 MHz, $CDCl_3$, 25 °C): δ = 177.6 (4-*C*OC(C*H*$_3$)$_3$), 109.9 (2,3-O*C*(CH_3)$_2$), 98.2 (C-1), 76.1 (C-2), 76.0 (C-3), 74.3 (C-4), 64.1 (C-5), 55.1 (1-O*C*H$_3$), 38.9 (4-CO*C*(C*H*$_3$)$_3$), 27.9 (2,3-OC*(CH$_3$)$_2$*), 27.3 (4-COC*(CH$_3$)$_3$*), 26.6 (2,3-OC*(CH$_3$)$_2$*), 17.2 (C-6) ppm.

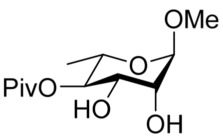

Methyl 4-*O*-pivaoyl-α-L-rhamnopyranoside (48): Synthesized from **113** (10 mg) according to the standard reaction procedure D to provide **48** as a white solid. Yield: 5 mg (57%). ^{1}H NMR (500.20 MHz, MeOD, 25 °C): δ = 4.90 (dd, 1 H, $J_{H\text{-}3,H\text{-}4}$ = 9.8 Hz, $J_{H\text{-}4,H\text{-}5}$ = 9.8 Hz, H-4), 4.60 (d, 1 H, $J_{H\text{-}1,H\text{-}2}$ = 1.7 Hz, H-1), 3.80 (dd, 1 H, $J_{H\text{-}2,H\text{-}3}$ = 3.5 Hz, H-2), 3.76 (dd, 1 H, H-3), 3.70 (dddd, 1 H, $J_{H\text{-}5,H\text{-}6}$ = 6.3 Hz, H-5), 3.37 (s, 3 H, 1-OC*H*$_3$), 1.22 (s, 9 H, 4-OCOC(C*H*$_3$)$_3$), 1.13 (d, 3 H, H-6) ppm. ^{13}C NMR (125.78 MHz, MeOD, 25 °C): δ = 179.6 (4-*C*OC(C*H*$_3$)$_3$), 102.7 (C-1), 75.1 (C-4), 72.4 (C-2), 70.5 (C-3), 67.5 (C-5), 55.2 (1-O*C*H$_3$), 39.9 (4-CO*C*(C*H*$_3$)$_3$), 27.5 (4-COC*(CH$_3$)$_3$*), 17.9 (C-6) ppm.

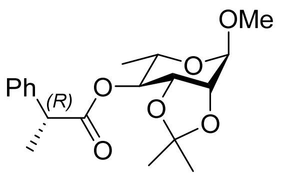

Methyl 2,3-*O*-isopropylidene-4-*O*-(*R*)-2-phenyl-propanoyl-α-L-rhamnopyranoside (114): Synthesized from **110** (50 mg) according to the standard reaction procedure B. The crude product was purified by column chromatography (hexane:EtOAc 7:1) to give **114** as a clear oil. Yield: 43 mg (54%), R_f = 0.22 (hexane:EtOAc 7:1). ^{1}H NMR (500.20 MHz, $CDCl_3$, 25 °C): δ = 7.26 – 7.15 (m, 5 H, aromatic H), 4.78 (d, 1 H, $J_{H-1,H-2}$ = 0.7 Hz, H-1), 4.75 (dd, 1 H, $J_{H-3,H-4}$ = 7.9 Hz, $J_{H-4,H-5}$ = 10.1 Hz, H-4), 4.06 (dd, 1 H, $J_{H-2,H-3}$ = 5.5 Hz, H-3), 4.03 (dd, 1 H, H-2), 3.66 (ddd, 1 H, $J_{4\text{-CO}\underline{\text{CH}}\text{CH3Ph, 4-COCH}\underline{\text{CH3}}\text{Ph}}$ = 7.1 Hz, 4-CO*C*HCH_3Ph),3.44 (dddd, 1 H, $J_{H-5,H-6}$ = 6.3 Hz, H-5), 3.24 (s, 3 H, 1-OCH_3), 1.52 (s, 3 H, 2,3-OC$(CH_3)_2$), 1.44 (d, 3 H, 4-COCHCH_3Ph)1.28 (s, 3 H, 2,3-OC$(CH_3)_2$), 0.69 (d, 3 H, H-6) ppm. ^{13}C NMR (125.78 MHz, $CDCl_3$, 25 °C): δ = 173.7 (4-*C*OCHCH$_3$Ph), 140.6, 128.8, 127.7, 127.3 (aromatic C), 109.9 (2,3-O*C*$(CH_3)_2$), 98.1 (C-1), 76.1 (C-2), 75.9 (C-3), 74.8 (C-4), 63.9 (C-5), 55.0 (1-O*C*H$_3$), 45.7 (4-CO*CH*CH$_3$Ph), 27.9 (2,3-OC$(CH_3)_2$), 26.6 (2,3-OC$(CH_3)_2$), 18.3 (4-COCH*C*H_3Ph), 16.6 (C-6) ppm.

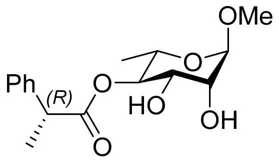

Methyl 4-*O*-(*R*)-2-phenyl-propanoyl-α-L-rhamnopyranoside (49): Synthesized from **114** (10 mg) according to the standard reaction procedure D to provide **49** as a white solid. Yield: 5 mg (56%). ^{1}H NMR (500.20 MHz, MeOD, 25 °C): δ = 7.34 – 7.22 (m, 5 H, aromatic H), 4.88 (dd, 1 H, $J_{H-3,H-4}$ = 9.8 Hz, $J_{H-4,H-5}$ = 9.7 Hz, H-4), 4.54 (d, 1 H, $J_{H-1,H-2}$ = 1.7 Hz, H-1), 3.794 (dd, 1 H, $J_{H-2,H-3}$ = 3.5 Hz, H-2), 3.788 (ddd, 1 H, $J_{4\text{-CO}\underline{\text{CH}}\text{CH3Ph, 4-COCH}\underline{\text{CH3}}\text{Ph}}$ = 7.1 Hz, 4-CO*CH*CH$_3$Ph), 3.74 (dd, 1 H, H-3), 3.46 (dddd, 1 H, $J_{H-5,H-6}$ = 6.3 Hz, H-5), 3.30 (s, 3 H, 1-OCH_3), 1.47 (d, 3 H, 4-COCHCH_3Ph), 0.75 (d, 3 H, H-6) ppm. ^{13}C NMR (125.78 MHz, MeOD, 25 °C): δ = 175.7 (4-*C*OCHCH$_3$Ph), 142.1, 129.7, 128.6, 128.3 (aromatic C), 102.7 (C-1), 75.5 (C-4), 72.2 (C-2), 70.4 (C-3), 67.4 (C-5), 55.2 (1-O*C*H$_3$), 46.8 (4-CO*CH*CH$_3$Ph), 18.6 (C-6), 17.4 (4-COCH*C*H_3Ph) ppm.

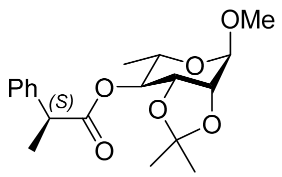

Methyl 2,3-*O*-isopropylidene-4-*O*-(*S*)-2-phenyl-propanoyl-α-L-rhamnopyranoside (115): Synthesized from **110** (50 mg) according to the standard reaction procedure B. The crude product was purified by column chromatography (hexane:EtOAc 6:1) to give **115** as a clear oil. Yield: 53 mg (66%), R_f = 0.31 (hexane:EtOAc 6:1). ^{1}H NMR (500.20 MHz, $CDCl_3$, 25 °C): δ = 7.34 – 7.23 (m, 5 H, aromatic H), 4.86 (d, 1 H, $J_{H-1,H-2}$ = 0.6 Hz, H-1), 4.84 (dd, 1 H, $J_{H-3,H-4}$ = 7.8 Hz, $J_{H-4,H-5}$ = 10.1 Hz, H-4), 4.08 (dd, 1 H, $J_{H-2,H-3}$ = 5.5 Hz, H-2), 4.06 (dd, 1 H, H-3), 3.77 (ddd, 1 H, $J_{4\text{-CO}\underline{\text{CH}}\text{CH3Ph, 4-COCH}\underline{\text{CH3}}\text{Ph}}$ = 7.2 Hz, 4-CO*CH*CH$_3$Ph), 3.67 (dddd, 1 H, $J_{H-5,H-6}$ = 6.3 Hz, H-5), 3.36 (s, 3 H, 1-OCH_3), 1.55 (s, 3 H, 2,3-OC$(CH_3)_2$), 1.53 (d, 3 H, 4-COCHCH_3Ph), 1.32 (s, 3 H, 2,3-OC$(CH_3)_2$), 1.06 (d, 3 H, H-6) ppm. ^{13}C NMR (125.78 MHz, $CDCl_3$, 25 °C): δ = 173.7 (4-*C*OCHCH$_3$Ph), 140.3, 128.7, 127.7, 127.2 (aromatic C), 109.9 (2,3-O*C*$(CH_3)_2$), 98.2 (C-1), 76.0 (C-2), 75.5 (C-3), 75.0 (C-4), 63.9 (C-5), 55.1 (1-O*C*H$_3$), 45.9 (4-CO*CH*CH$_3$Ph), 27.8 (2,3-OC$(CH_3)_2$), 26.6 (2,3-OC$(CH_3)_2$), 18.6 (4-COCH*C*H_3Ph), 17.0 (C-6) ppm.

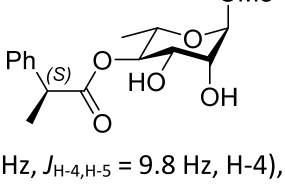

Methyl 4-*O*-(*S*)-2-phenyl-propanoyl-α-L-rhamnopyranoside (50): Synthesized from **115** (10 mg) according to the standard reaction procedure D to provide **50** as a white solid. Yield: 7 mg (79%). ^{1}H NMR (500.20 MHz, MeOD, 25 °C): δ = 7.35 – 7.21 (m, 5 H, aromatic H), 4.90 (dd, 1 H, $J_{H-3,H-4}$ = 9.8 Hz, $J_{H-4,H-5}$ = 9.8 Hz, H-4), 4.57 (d, 1 H, $J_{H-1,H-2}$ = 1.7 Hz, H-1), 3.80 (ddd, 1 H, $J_{4\text{-CO}\underline{\text{CH}}\text{CH3Ph, 4-COCH}\underline{\text{CH3}}\text{Ph}}$ = 7.2 Hz, 4-CO*CH*CH$_3$Ph), 3.76 (dd, 1 H, $J_{H-2,H-3}$ = 3.5 Hz, H-2), 3.71 (dd, 1 H, H-3), 3.66 (dddd, 1 H, $J_{H-5,H-6}$ = 6.3 Hz, H-5), 3.34 (s, 3 H, 1-OCH_3), 1.51 (d, 3 H, 4-COCHCH_3Ph), 1.05 (d, 3 H, H-6) ppm. ^{13}C NMR (125.78 MHz, MeOD, 25 °C): δ = 175.8 (4-*C*OCHCH$_3$Ph), 141.8, 129.5, 128.7, 128.1 (aromatic C), 102.7 (C-1), 75.7 (C-

4), 72.3 (C-2), 70.3 (C-3), 67.4 (C-5), 55.2 (1-O*C*H$_3$), 47.1 (4-CO*CH*CH$_3$Ph), 18.9 (C-6), 17.8 (4-COCH*CH$_3$*Ph) ppm.

Fucopyranoside derivatives

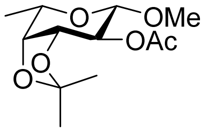

Methyl 2-*O*-acetyl-3,4-*O*-isopropylidene-β-L-fucopyranoside (117): Synthesized from **116** (50 mg) according to the standard procedure for acylation. The crude product was purified by column chromatography (hexane:EtOAc 4:1) to provide **117** as a white solid. Yield: 48 mg (80%), R_f = 0.12 (hexane:EtOAc 4:1). ^{1}H NMR (500.20 MHz, CDCl$_3$, 25 °C): δ = 4.88 (dd, 1 H, $J_{H\text{-}1,H\text{-}2}$ = 8.0 Hz, $J_{H\text{-}2,H\text{-}3}$ = 7.6 Hz, H-2), 4.15 (d, 1 H, H-1), 4.07 (dd, 1 H, $J_{H\text{-}3,H\text{-}4}$ = 5.3 Hz, H-3), 3.96 (dd, 1 H, $J_{H\text{-}4,H\text{-}5}$ = 2.2 Hz, H-4), 3.81 (dddd, 1 H, $J_{H\text{-}5,H\text{-}6}$ = 6.7 Hz, H-5), 3.40 (s, 3 H, 1-OC*H$_3$*), 2.03 (s, 3 H, 2-OCOC*H$_3$*), 1.50 (s, 3 H, 3,4-OC*(CH$_3$)$_2$*), 1.37 (d, 3 H, H-6), 1.28 (s, 3 H, 3,4-OC*(CH$_3$)$_2$*) ppm. ^{13}C NMR (125.78 MHz, CDCl$_3$, 25 °C): δ = 169.9 (2-*C*OCH$_3$), 110.3 (3,4-O*C*(CH$_3$)$_2$), 101.3 (C-1), 77.3 (C-3), 76.6 (C-4), 73.1 (C-2), 69.1 (C-5), 56.5 (1-O*C*H$_3$), 27.9 (3,4-OC*(CH$_3$)$_2$*), 26.5 (3,4-OC*(CH$_3$)$_2$*), 21.2 (2-CO*C*H$_3$), 16.6 (C-6) ppm.

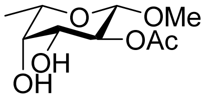

Methyl 2-*O*-acetyl-β-L-fucopyranoside (51): Synthesized from **117** (10 mg) according to the standard reaction procedure D to provide **51** as a white solid. Yield: 4.6 mg (54%). ^{1}H NMR (500.20 MHz, MeOD, 25 °C): δ = 4.96 (dd, 1 H, $J_{H\text{-}1,H\text{-}2}$ = 8.0 Hz, $J_{H\text{-}2,H\text{-}3}$ = 9.6 Hz, H-2), 4.28 (d, 1 H, H-1), 3.66 (dddd, 1 H, $J_{H\text{-}4,H\text{-}5}$ = 1.4 Hz, $J_{H\text{-}5,H\text{-}6}$ = 6.7 Hz, H-5), 3.64 (dd, 1 H, $J_{H\text{-}3,H\text{-}4}$ = 3.6 Hz, H-4), 3.63 (dd, 1 H, H-3), 3.43 (s, 3 H, 1-OC*H$_3$*), 2.06 (s, 3 H, 2-OCOC*H$_3$*), 1.28 (d, 3 H, H-6) ppm. ^{13}C NMR (125.78 MHz, MeOD, 25 °C): δ = 172.3 (2-*C*OCH$_3$), 103.4 (C-1), 73.6 (C-2), 73.4 (C-3), 73.1 (C-4), 72.1 (C-5), 56.8 (1-O*C*H$_3$), 21.0 (2-CO*C*H$_3$), 16.6 (C-6) ppm.

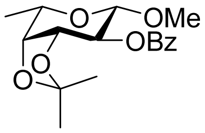

Methyl 2-*O*-benzoyl-3,4-*O*-isopropylidene-β-L-fucopyranoside (118): Synthesized from **116** (50 mg) according to the standard procedure for acylation. The crude product was purified by column chromatography (hexane:EtOAc 4:1) to provide **118** as a clear oil. Yield: 68 mg (92%), R_f = 0.26 (hexane:EtOAc 4:1). ^{1}H NMR (500.20 MHz, CDCl$_3$, 25 °C): δ = 8.07 – 8.03 (m, 2 H, aromatic H), 7.57 – 7.53 (m, 1 H, aromatic H), 7.44 – 7.41 (m, 2 H, aromatic H), 5.21 (dd, 1 H, $J_{H\text{-}1,H\text{-}2}$ = 8.0 Hz, $J_{H\text{-}2,H\text{-}3}$ = 7.5 Hz, H-2), 4.38 (d, 1 H, H-1), 4.31 (dd, 1 H, $J_{H\text{-}3,H\text{-}4}$ = 5.4 Hz, H-3), 4.09 (dd, 1 H, $J_{H\text{-}4,H\text{-}5}$ = 2.2 Hz, H-4), 3.95 (dddd, 1 H, $J_{H\text{-}5,H\text{-}6}$ = 6.7 Hz, H-5), 3.46 (s, 3 H, 1-OC*H$_3$*), 1.62 (s, 3 H, 3,4-OC*(CH$_3$)$_2$*), 1.47 (d, 3 H, H-6), 1.34 (s, 3 H, 3,4-OC*(CH$_3$)$_2$*) ppm. ^{13}C NMR (125.78 MHz, CDCl$_3$, 25 °C): δ = 164.6 (2-*C*OPh), 132.1, 129.0, 127.4 (aromatic C), 109.4 (3,4-O*C*(CH$_3$)$_2$), 100.5 (C-1), 76.4 (C-3), 75.6 (C-4), 72.7 (C-2), 68.1 (C-5), 55.7 (1-O*C*H$_3$), 26.9 (3,4-OC*(CH$_3$)$_2$*), 25.5 (3,4-OC*(CH$_3$)$_2$*), 15.7 (C-6) ppm.

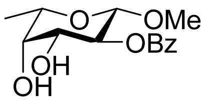

Methyl 2-*O*-benzoyl-β-L-fucopyranoside (52): Synthesized from **118** (10 mg) according to the standard reaction procedure D to provide **52** as a white solid. Yield: 8.0 mg (91%). ^{1}H NMR (500.20 MHz, MeOD, 25 °C): δ = 8.07 – 8.03 (m, 2 H, aromatic H), 7.64 – 7.59 (m, 1 H, aromatic H), 7.51 – 7.46 (m, 2 H, aromatic H), 5.22 (dd, 1 H, $J_{H\text{-}1,H\text{-}2}$ = 8.0 Hz, $J_{H\text{-}2,H\text{-}3}$ = 10.0 Hz, H-2), 4.46 (d, 1 H, H-1), 3.81 (dd, 1 H, $J_{H\text{-}3,H\text{-}4}$ = 3.4 Hz, H-3), 3.75 (dddd, 1 H, $J_{H\text{-}4,H\text{-}5}$ = 0.8 Hz, $J_{H\text{-}5,H\text{-}6}$ = 6.7 Hz, H-5), 3.70 (dd, 1 H, H-4), 3.43 (s, 3 H, 1-OC*H$_3$*), 1.32 (d, 3 H, H-6) ppm. ^{13}C NMR (125.78 MHz, MeOD, 25 °C): δ = 167.6 (2-*C*OPh), 134.2, 131.7, 130.7, 129.5 (aromatic C), 103.5 (C-1), 74.3 (C-2), 73.4 (C-3), 73.3 (C-4), 72.2 (C-5), 56.9 (1-O*C*H$_3$), 16.6 (C-6) ppm.

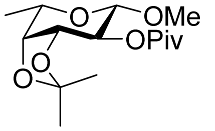

Methyl 3,4-*O*-isopropylidene-2-*O*-pivaoyl-β-L-fucopyranoside (119): Synthesized from **116** (50 mg) according to the standard procedure for acylation. The crude product was purified by column chromatography (hexane:EtOAc 4:1) to provide **119** as a clear oil. Yield: 49 mg (71%), R_f = 0.35 (hexane:EtOAc 4:1). ^{1}H NMR (500.20 MHz, $CDCl_3$, 25 °C): δ = 4.93 (dd, 1 H, $J_{H-1,H-2}$ = 8.0 Hz, $J_{H-2,H-3}$ = 7.3 Hz, H-2), 4.22 (d, 1 H, H-1), 4.13 (dd, 1 H, $J_{H-3,H-4}$ = 5.4 Hz, H-3), 4.02 (dd, 1 H, $J_{H-4,H-5}$ = 2.1 Hz, H-4), 3.87 (dddd, 1 H, $J_{H-5,H-6}$ = 6.6 Hz, H-5), 3.46 (s, 3 H, 1-OCH_3), 1.58 (s, 3 H, 3,4-OC*(CH$_3$)$_2$*), 1.44 (d, 3 H, H-6), 1.35 (s, 3 H, 3,4-OC*(CH$_3$)$_2$*), 1.22 (s, 9 H, 2-OCOC(CH_3)$_3$) ppm. ^{13}C NMR (125.78 MHz, $CDCl_3$, 25 °C): δ = 177.3 (4-*C*OC(CH_3)$_3$), 110.3 (3,4-O*C*(CH$_3$)$_2$), 101.8 (C-1), 77.4 (C-3), 76.6 (C-4), 72.8 (C-2), 69.1 (C-5), 56.7 (1-O*C*H$_3$), 38.9 (4-CO*C*(CH_3)$_3$), 27.9 (3,4-OC*(CH$_3$)$_2$*), 27.2 (4-COC*(CH$_3$)$_3$*), 26.6 (3,4-OC*(CH$_3$)$_2$*), 16.7 (C-6) ppm.

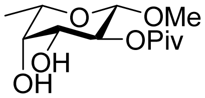

Methyl 2-*O*-pivaoyl-β-L-fucopyranoside (53): Synthesized from **119** (10 mg) according to the standard reaction procedure D to provide **53** as a white solid. Yield: 7.0 mg (81%). ^{1}H NMR (500.20 MHz, MeOD, 25 °C): δ = 4.94 (dd, 1 H, $J_{H-1,H-2}$ = 8.0 Hz, $J_{H-2,H-3}$ = 9.8 Hz, H-2), 4.28 (d, 1 H, H-1), 3.68 (dddd, 1 H, $J_{H-4,H-5}$ = 1.2 Hz, $J_{H-5,H-6}$ = 6.7 Hz, H-5), 3.64 (dd, 1 H, $J_{H-3,H-4}$ = 3.5 Hz, H-3), 3.63 (dd, 1 H, H-4), 3.43 (s, 3 H, 1-OCH_3), 1.21 (s, 9 H, 4-OCOC(CH_3)$_3$), 1.29 (d, 3 H, H-6) ppm. ^{13}C NMR (125.78 MHz, MeOD, 25 °C): δ = 179.5 (4-*C*OC(CH_3)$_3$), 103.7 (C-1), 73.39 (C-2), 73.34 (C-3), 73.3 (C-4), 72.1 (C-5), 57.0 (1-O*C*H$_3$), 39.9 (4-CO*C*(CH_3)$_3$), 27.5 (4-COC*(CH$_3$)$_3$*), 16.6 (C-6) ppm.

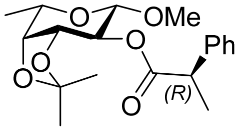

Methyl 3,4-*O*-isopropylidene-2-*O*-(*R*)-2-phenyl-propanoyl-β-L-fucopyranoside (120): Synthesized from **116** (50 mg) according to the standard reaction procedure B. The crude product was purified by column chromatography (hexane:EtOAc 4:1) to give **120** as a clear oil. Yield: 66 mg (81%), R_f = 0.26 (hexane:EtOAc 4:1). ^{1}H NMR (500.20 MHz, $CDCl_3$, 25 °C): δ = 7.34 – 7.22 (m, 5 H, aromatic H), 4.94 (dd, 1 H, $J_{H-1,H-2}$ = 8.1 Hz, $J_{H-2,H-3}$ = 7.3 Hz, H-2), 4.20 (d, 1 H, H-1), 4.03 (dd, 1 H, $J_{H-3,H-4}$ = 5.4 Hz, H-3), 3.95 (dd, 1 H, $J_{H-4,H-5}$ = 2.1 Hz, H-4), 3.81 (dddd, 1 H, $J_{H-5,H-6}$ = 6.5 Hz, H-5), 3.77 (ddd, 1 H, $J_{2\text{-CO}\underline{CH}\text{CH3Ph}, 2\text{-COCH}\underline{CH3}\text{Ph}}$ = 7.2 Hz, 2-COC*H*CH$_3$Ph), 3.40 (s, 3 H, 1-OC*H$_3$*), 1.56 (s, 3 H, 3,4-OC*(CH$_3$)$_2$*), 1.52 (d, 3 H, 2-COCHC*H$_3$*Ph), 1.41 (d, 3 H, H-6), 1.32 (s, 3 H, 3,4-OC*(CH$_3$)$_2$*) ppm. ^{13}C NMR (125.78 MHz, $CDCl_3$, 25 °C): δ = 173.1 (2-*C*OCHCH$_3$Ph), 140.8, 128.6, 127.8, 127.1 (aromatic C), 110.3 (3,4-O*C*(CH$_3$)$_2$), 101.4 (C-1), 77.1 (C-3), 76.5 (C-4), 73.5 (C-2), 69.0 (C-5), 56.6 (1-O*C*H$_3$), 45.9 (2-CO*CH*CH$_3$Ph), 27.9 (3,4-OC*(CH$_3$)$_2$*), 26.6 (3,4-OC*(CH$_3$)$_2$*), 19.0 (2-COCH*CH$_3$*Ph), 16.7 (C-6) ppm.

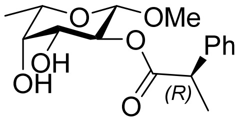

Methyl 2-*O*-(*R*)-2-phenyl-propanoyl-β-L-fucopyranoside (54): Synthesized from **120** (10 mg) according to the standard reaction procedure D to provide **54** as a white solid. Yield: 5.1 mg (58%). ^{1}H NMR (500.20 MHz, MeOD, 25 °C): δ = 7.34 – 7.20 (m, 5 H, aromatic H), 4.97 (dd, 1 H, $J_{H-1,H-2}$ = 8.0 Hz, $J_{H-2,H-3}$ = 9.7 Hz, H-2), 4.27 (d, 1 H, H-1), 3.79 (ddd, 1 H, $J_{2\text{-CO}\underline{CH}\text{CH3Ph}, 2\text{-COCH}\underline{CH3}\text{Ph}}$ = 7.2 Hz, 2-COC*H*CH$_3$Ph), 3.64 (dddd, 1 H, $J_{H-4,H-5}$ = 1.3 Hz, $J_{H-5,H-6}$ = 6.5 Hz, H-5), 3.594 (dd, 1 H, $J_{H-3,H-4}$ = 3.6 Hz, H-4), 3.588 (dd, 1 H, H-3), 3.38 (s, 3 H, 1-OC*H$_3$*), 1.49 (d, 3 H, 2-COCHC*H$_3$*Ph), 1.27 (d, 3 H, H-6) ppm.^{13}C NMR (125.78 MHz, MeOD, 25 °C): δ = 175.6 (2-*C*OCHCH$_3$Ph), 142.1, 129.4, 128.8, 128.0 (aromatic C), 103.5 (C-1), 73.9 (C-2), 73.28 (C-3), 73.26 (C-4), 72.0 (C-5), 55.9 (1-O*C*H$_3$), 47.1 (2-CO*CH*CH$_3$Ph), 19.4 (2-COCH*CH$_3$*Ph), 16.6 (C-6) ppm.

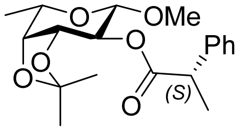

Methyl 3,4-*O*-isopropylidene-2-*O*-(*S*)-2-phenyl-propanoyl-β-L-fucopyranoside (121): Synthesized from **116** (50 mg) according to the standard reaction procedure B. The crude product was purified by column chromatography (hexane:EtOAc 4:1) to give **121** as a clear oil. Yield: 74 mg (92%), R_f = 0.25 (hexane:EtOAc 4:1). ^{1}H NMR (500.20 MHz, $CDCl_3$, 25 °C): δ = 7.33 – 7.20 (m, 5 H, aromatic H), 4.95 (dd, 1 H, $J_{H\text{-}1,H\text{-}2}$ = 8.3 Hz, $J_{H\text{-}2,H\text{-}3}$ = 7.5 Hz, H-2), 4.07 (dd, 1 H, $J_{H\text{-}3,H\text{-}4}$ = 5.4 Hz, H-3), 3.98 (dd, 1 H, $J_{H\text{-}4,H\text{-}5}$ = 2.1 Hz, H-4), 3.94 (d, 1 H, H-1), 3.77 (dddd, 1 H, $J_{H\text{-}5,H\text{-}6}$ = 6.6 Hz, H-5), 3.75 (ddd, 1 H, $J_{2\text{-}CO\underline{CH}CH3Ph,\ 2\text{-}COCH\underline{CH3}Ph}$ = 7.1 Hz, 2-CO*CH*CH_3Ph), 3.16 (s, 3 H, 1-O*CH*$_3$), 1.58 (s, 3 H, 3,4-OC*(CH$_3$)$_2$*), 1.49 (d, 3 H, 2-COCH*CH*$_3$Ph), 1.39 (d, 3 H, H-6), 1.35 (s, 3 H, 3,4-OC*(CH$_3$)$_2$*) ppm. ^{13}C NMR (125.78 MHz, $CDCl_3$, 25 °C): δ = 173.3 (2-*C*OCHCH_3Ph), 141.1, 128.7, 127.8, 127.0 (aromatic C), 110.4 (3,4-O*C*$(CH_3)_2$), 101.7 (C-1), 77.2 (C-3), 76.6 (C-4), 73.4 (C-2), 69.0 (C-5), 56.7 (1-O*C*H_3), 45.7 (2-CO*CH*CH_3Ph), 28.0 (3,4-OC*(CH$_3$)$_2$*), 26.6 (3,4-OC*(CH$_3$)$_2$*), 18.8 (2-COCH*CH*$_3$Ph), 16.7 (C-6) ppm.

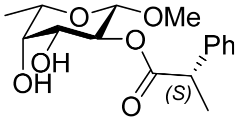

Methyl 2-*O*-(*S*)-2-phenyl-propanoyl-β-L-fucopyranoside (55): Synthesized from **121** (10 mg) according to the standard reaction procedure D to provide **55** as a white solid. Yield: 8.5 mg (96%). ^{1}H NMR (500.20 MHz, MeOD, 25 °C): δ = 7.33 – 7.20 (m, 5 H, aromatic H), 4.96 (dd, 1 H, $J_{H\text{-}1,H\text{-}2}$ = 8.0 Hz, $J_{H\text{-}2,H\text{-}3}$ = 9.7 Hz, H-2), 4.01 (d, 1 H, H-1), 3.80 (ddd, 1 H, $J_{2\text{-}CO\underline{CH}CH3Ph,\ 2\text{-}COCH\underline{CH3}Ph}$ = 7.1 Hz, 2-CO*CH*CH_3Ph), 3.612 (dd, 1 H, $J_{H\text{-}3,H\text{-}4}$ = 3.5 Hz, $J_{H\text{-}4,H\text{-}5}$ = 1.3 Hz, H-4), 3.606 (dd, 1 H, H-3), 3.59 (dddd, 1 H, $J_{H\text{-}5,H\text{-}6}$ = 6.5 Hz, H-5), 3.12 (s, 3 H, 1-O*CH*$_3$), 1.44 (d, 3 H, 2-COCH*CH*$_3$Ph), 1.24 (d, 3 H, H-6) ppm. ^{13}C NMR (125.78 MHz, MeOD, 25 °C): δ = 175.5 (2-*C*OCHCH_3Ph), 142.4, 129.5, 128.7, 127.9 (aromatic C), 103.8 (C-1), 73.8 (C-2), 73.15 (C-3), 73.08 (C-4), 72.0 (C-5), 57.1 (1-O*C*H_3), 46.7 (2-CO*CH*CH_3Ph), 19.1 (2-COCH*CH*$_3$Ph), 16.6 (C-6) ppm.

NMR spectra of the synthesized compounds

Methyl 2-*O*-acetyl-4,6-*O*-benzylidene-α-D-glucopyranoside (57):

^{1}H NMR (500.20 MHz, $CDCl_3$, 25°C):

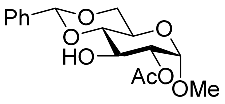

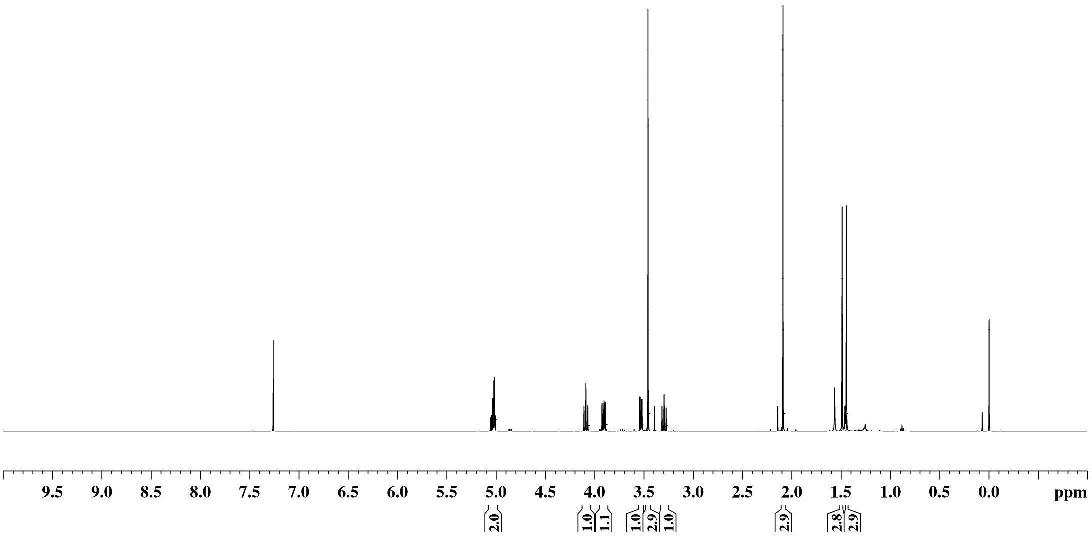

^{13}C NMR (125.8 MHz, $CDCl_3$, 25°C):

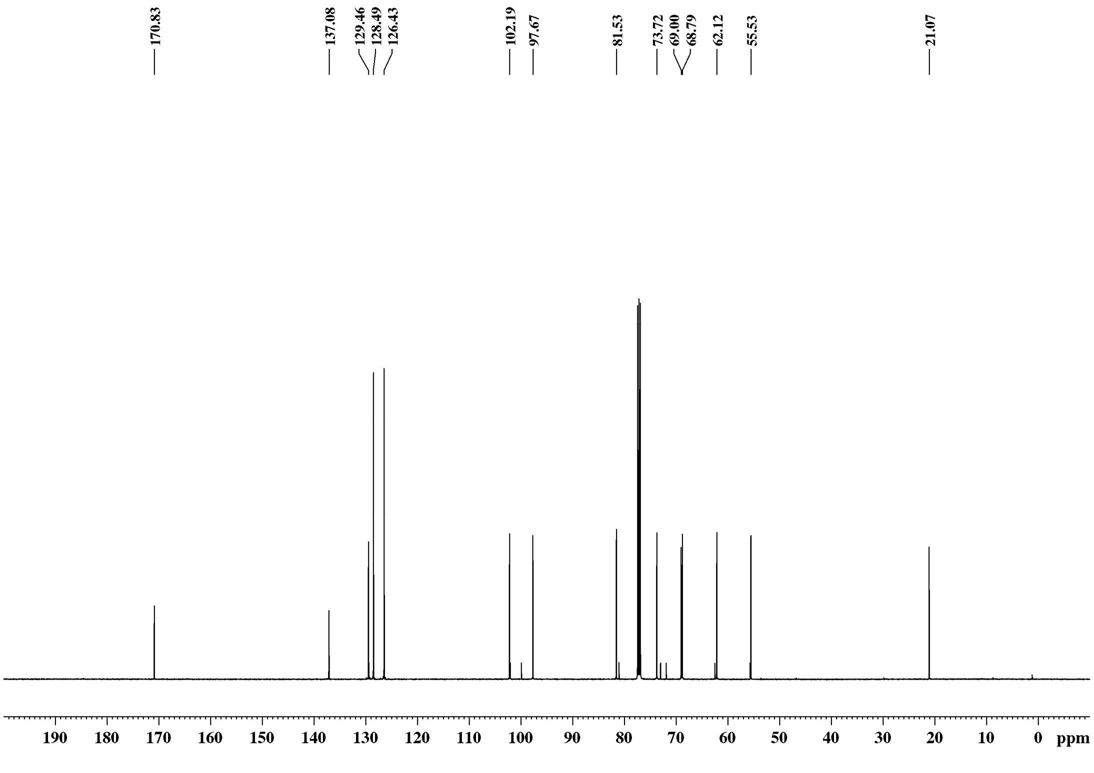

Methyl 2-*O*-acetyl-α-D-glucopyranoside (1):

^{1}H NMR (500.20 MHz, MeOD, 25°C):

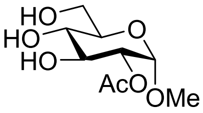

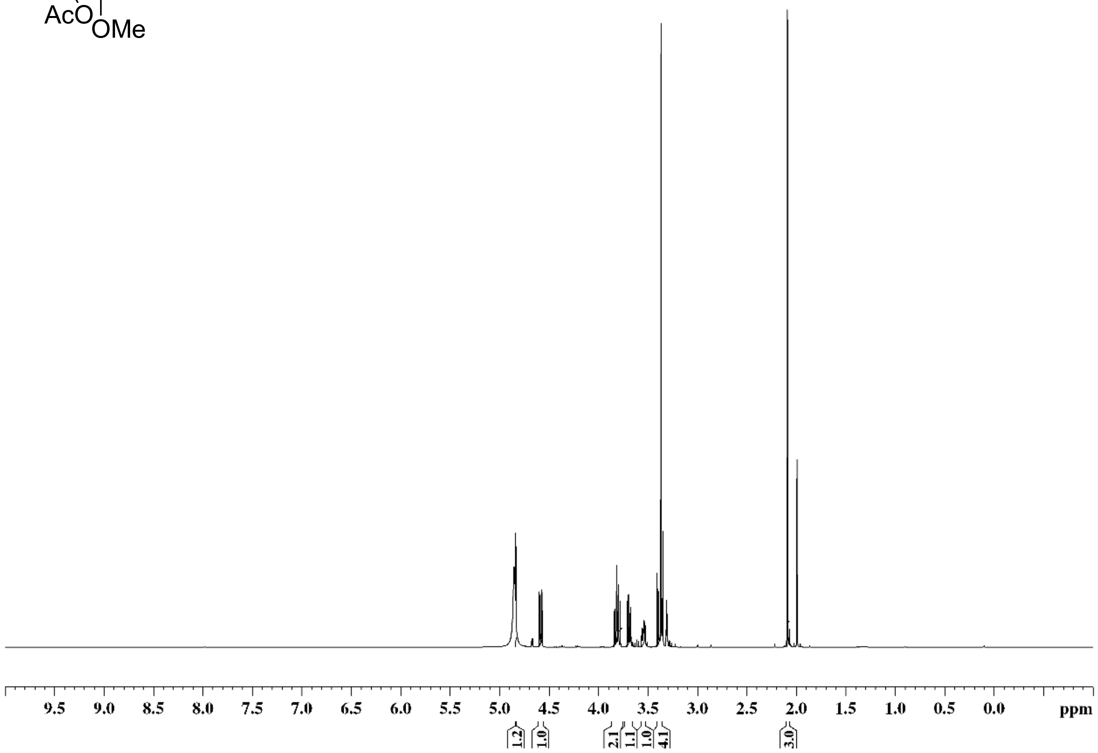

^{13}C NMR (125.8 MHz, MeOD, 25°C):

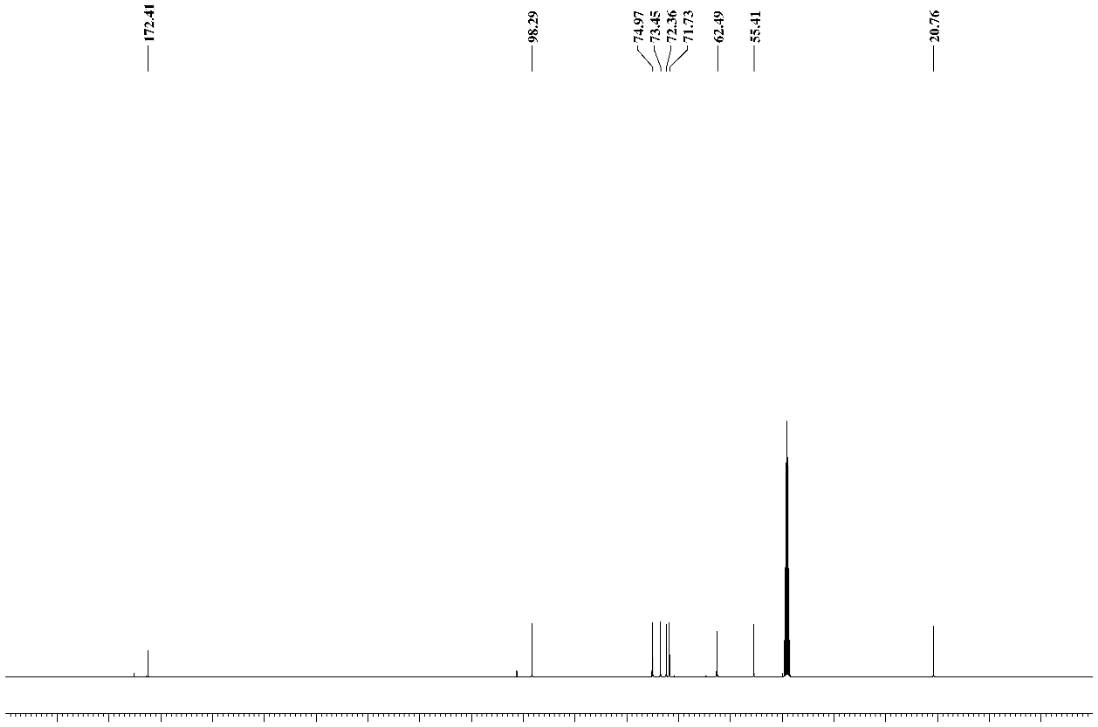

Methyl 2-*O*-benzoyl-4,6-*O*-benzylidene-α-D-glucopyranoside (58):

^{1}H NMR (500.20 MHz, $CDCl_3$, 25°C):

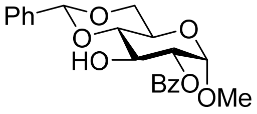

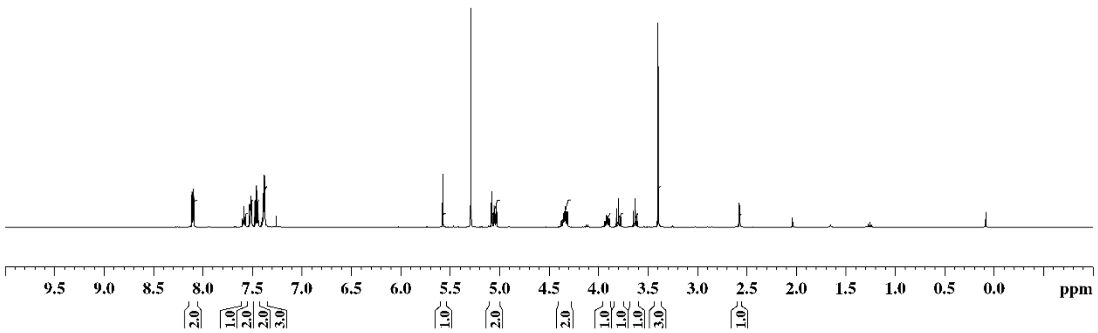

^{13}C NMR (125.8 MHz, $CDCl_3$, 25°C):

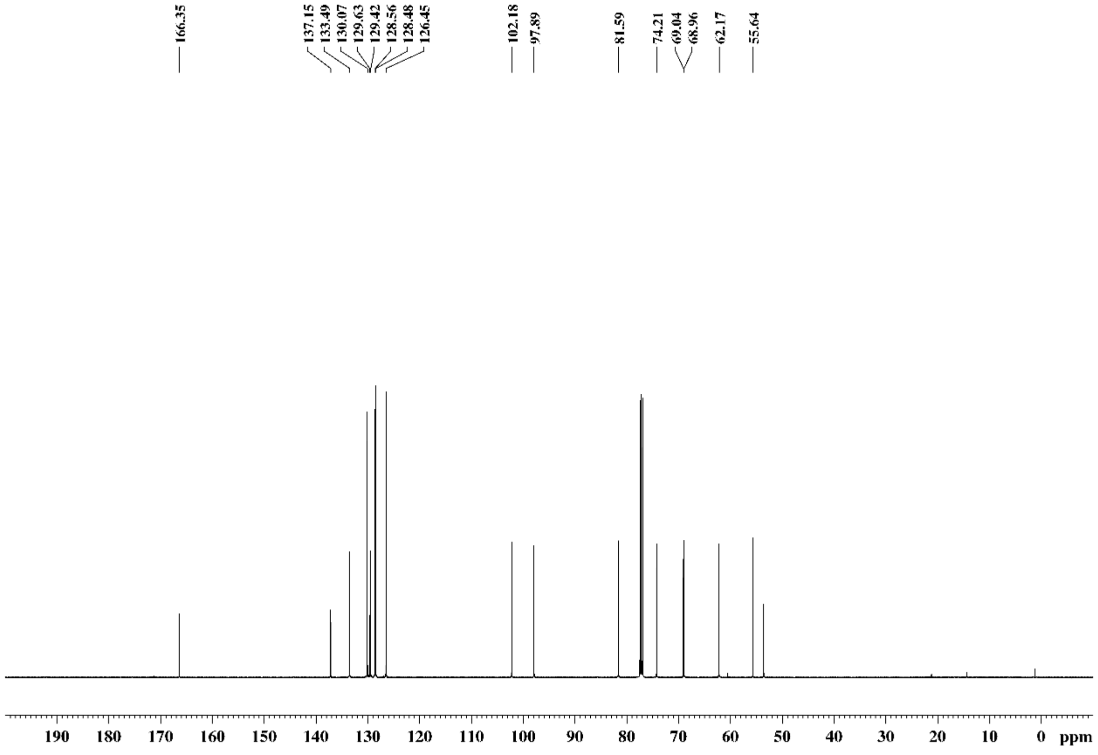

Methyl 2-*O*-benzoyl-α-D-glucopyranoside (2):

^{1}H NMR (500.20 MHz, MeOD, 25°C):

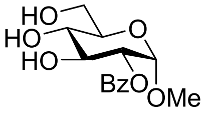

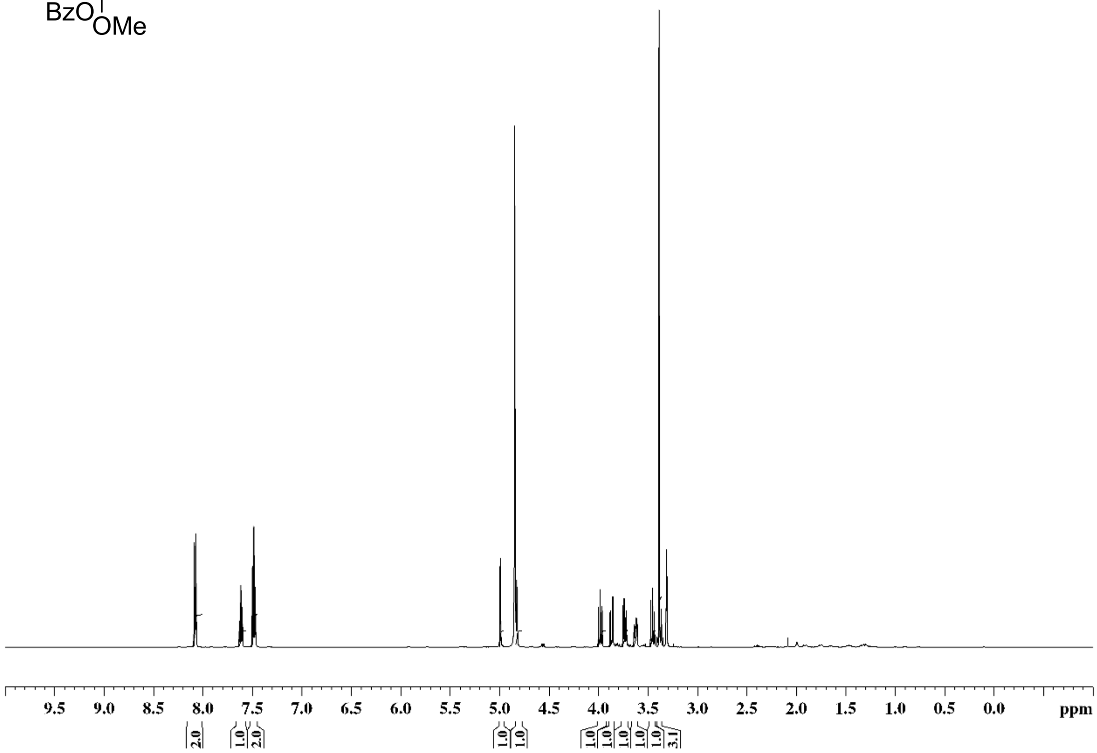

^{13}C NMR (125.8 MHz, MeOD, 25°C):

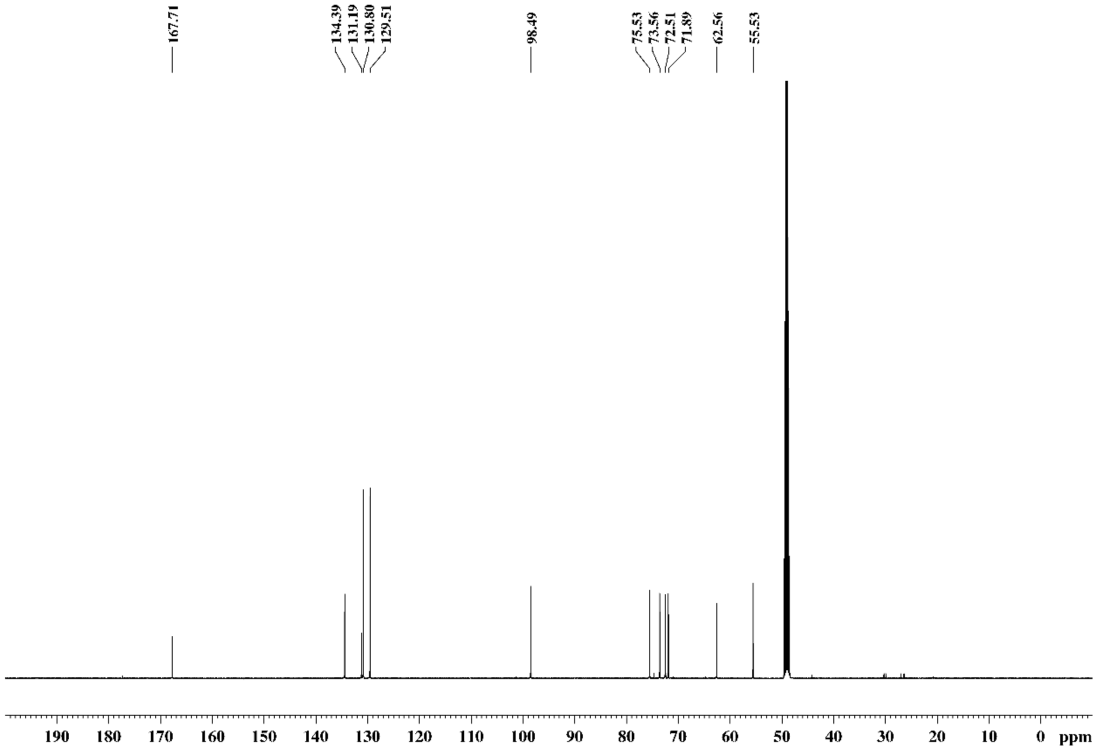

Methyl 4,6-*O*-benzylidene-2-*O*-pivaloyl-α-D-glucopyranoside (59):

^{1}H NMR (500.20 MHz, CDCl$_3$, 25°C):

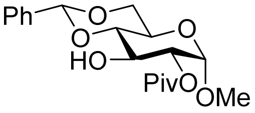

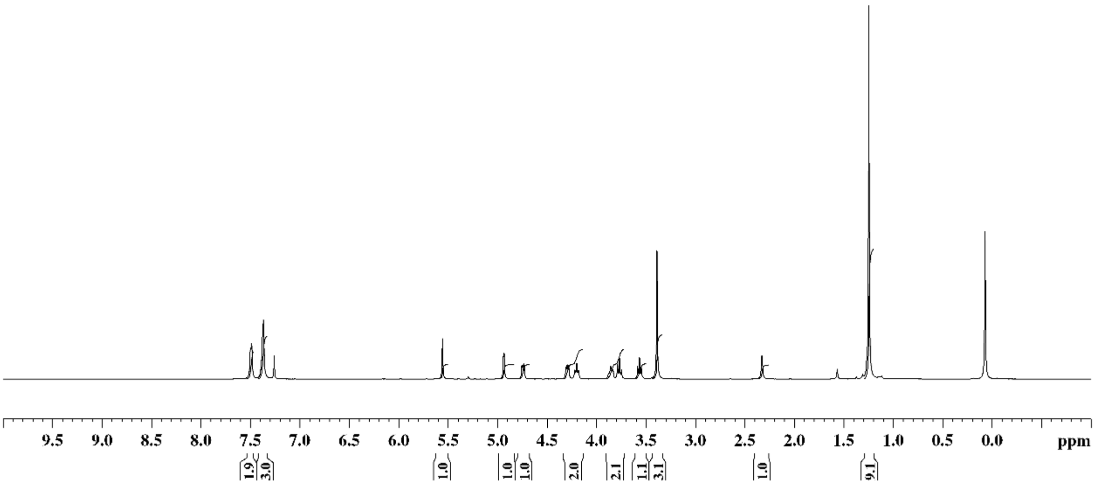

^{13}C NMR (125.8 MHz, CDCl$_3$, 25°C):

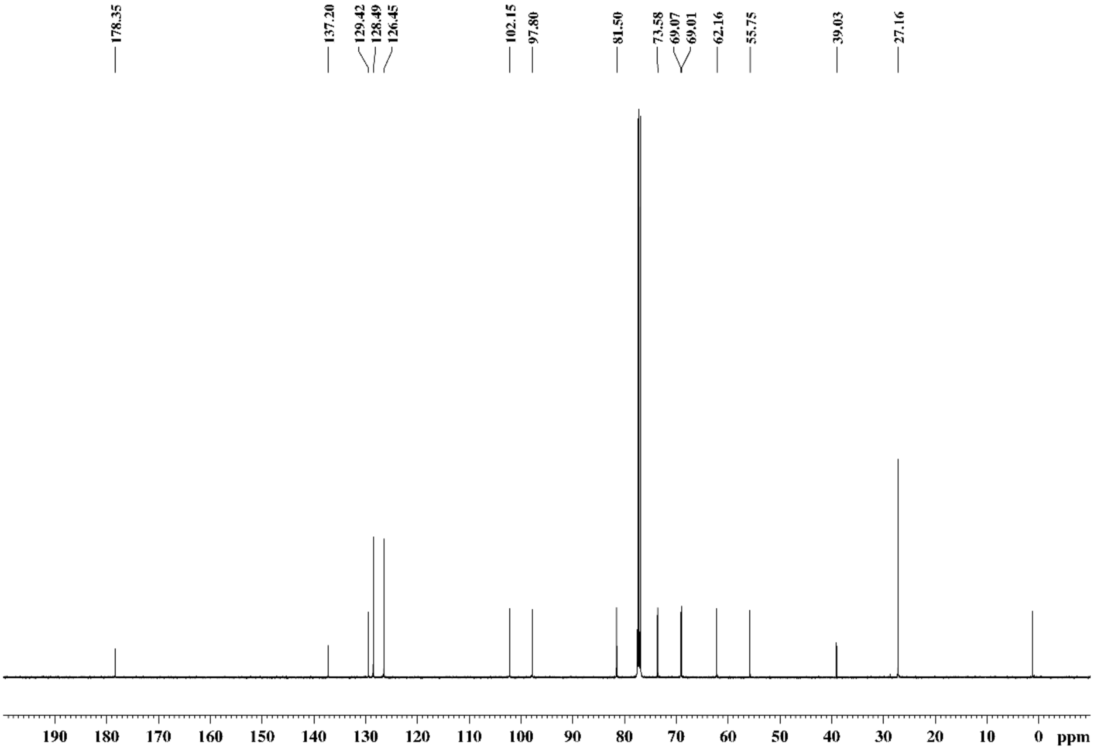

Methyl 2-*O*-pivaloyl-α-D-glucopyranoside (3):

^{1}H NMR (500.20 MHz, MeOD, 25°C):

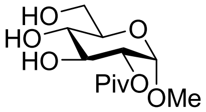

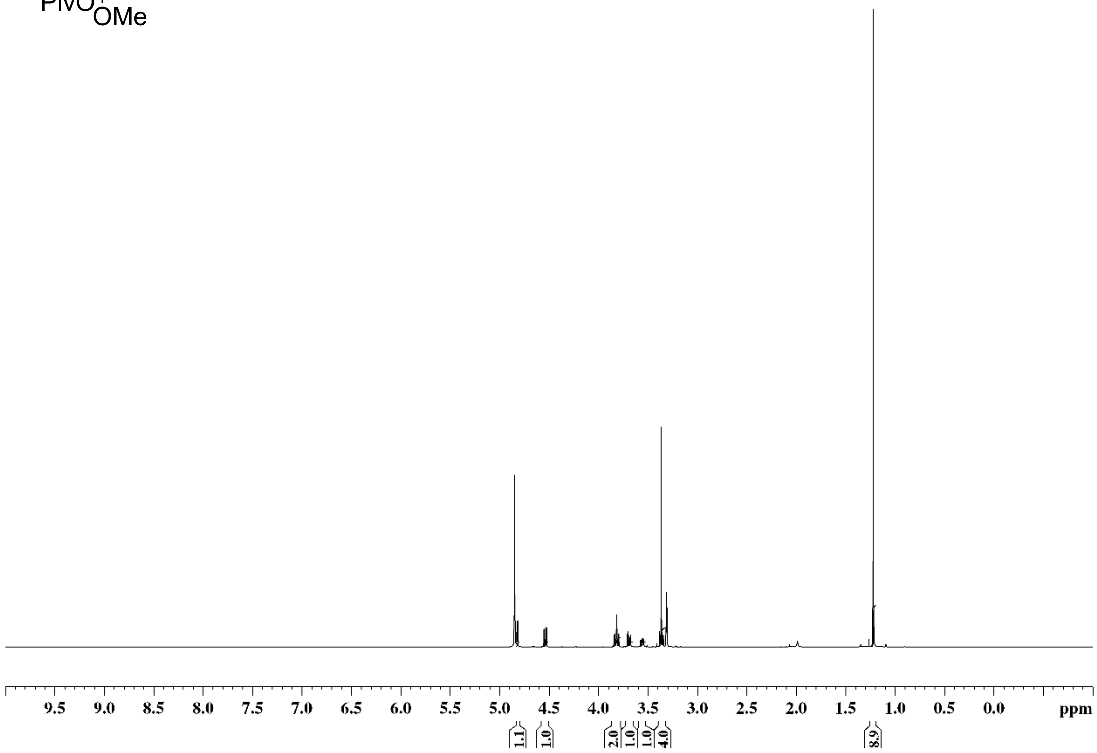

^{13}C NMR (125.8 MHz, MeOD, 25°C):

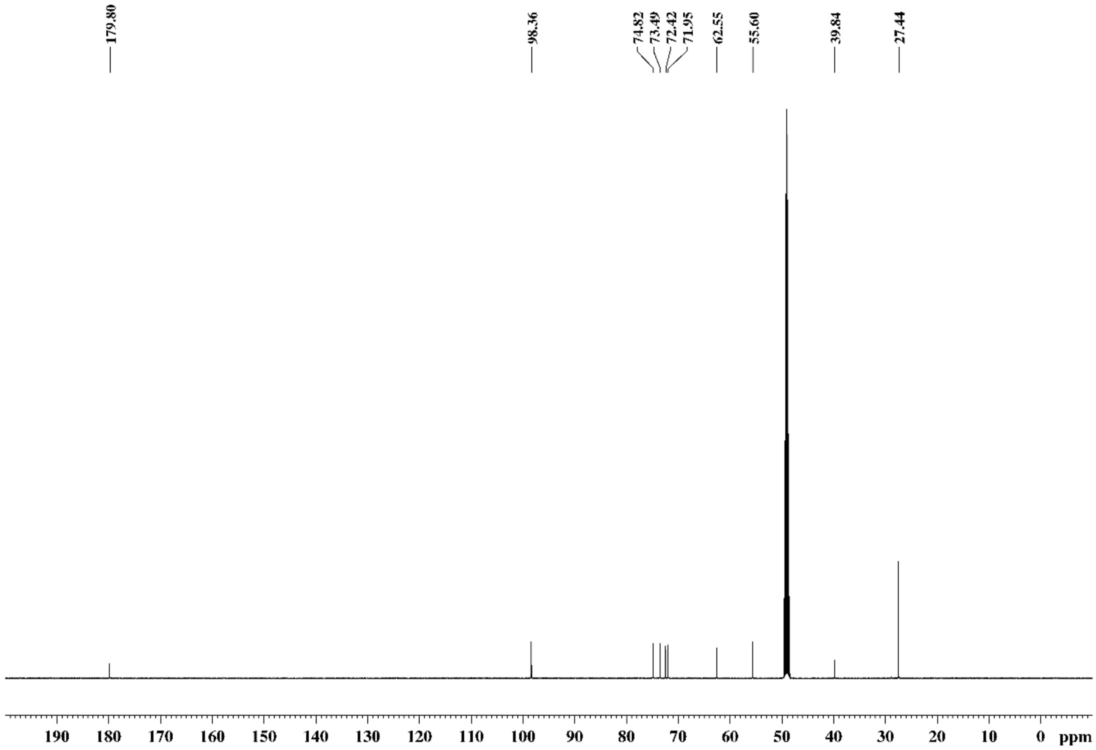

Methyl 2-*O*-(*R*)-2-phenyl-propanoyl-4,6-*O*-benzylidene-α-D-glucopyranoside (60):

^{1}H NMR (500.20 MHz, CDCl$_3$, 25°C):

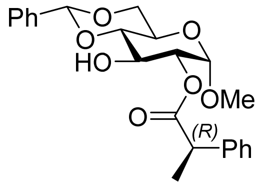

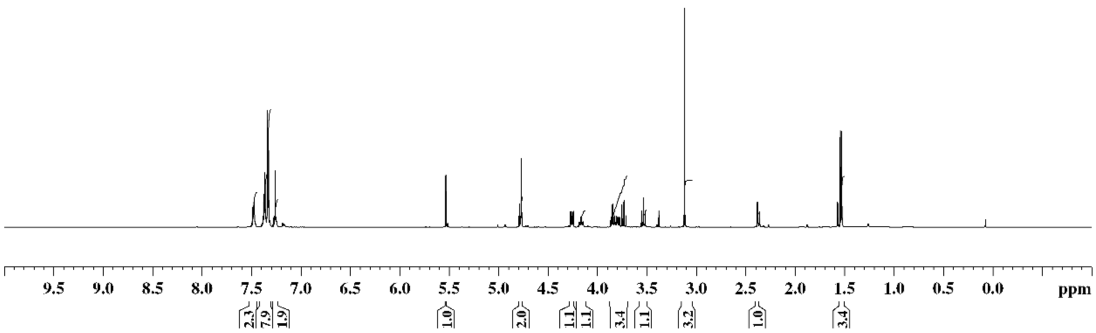

^{13}C NMR (125.8 MHz, CDCl$_3$, 25°C):

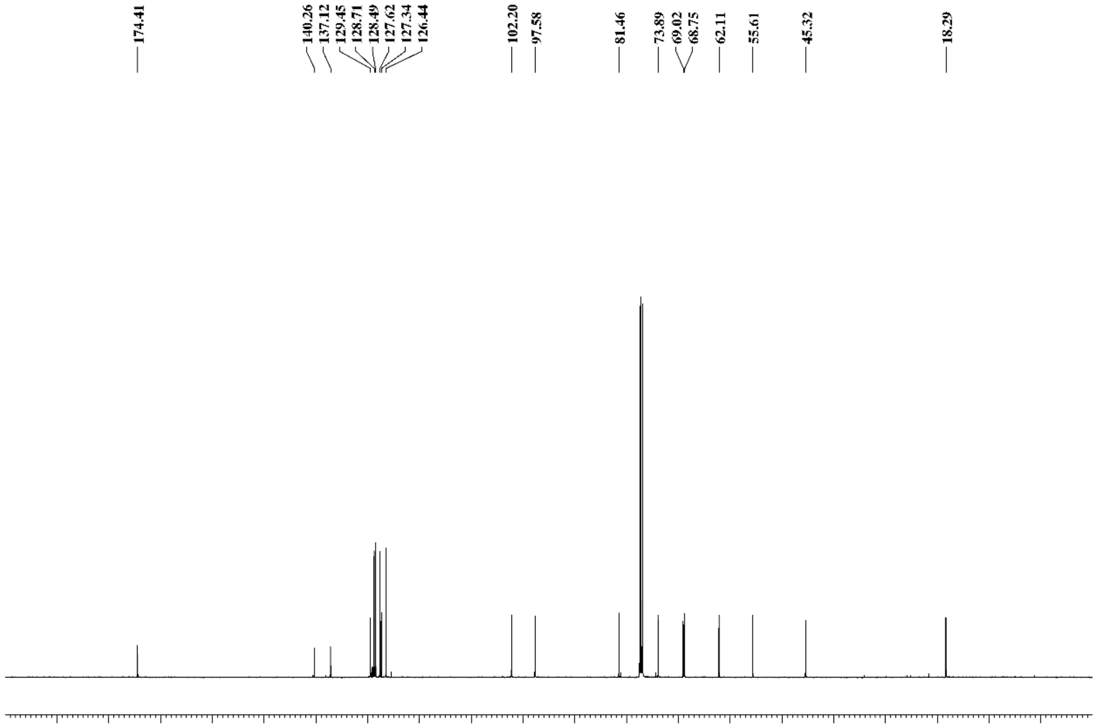

Methyl 2-*O*-(*R*)-2-phenyl-propanoyl-α-D-glucopyranoside (4):

^{1}H NMR (500.20 MHz, MeOD, 25°C):

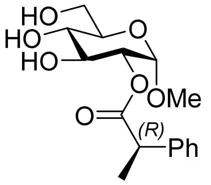

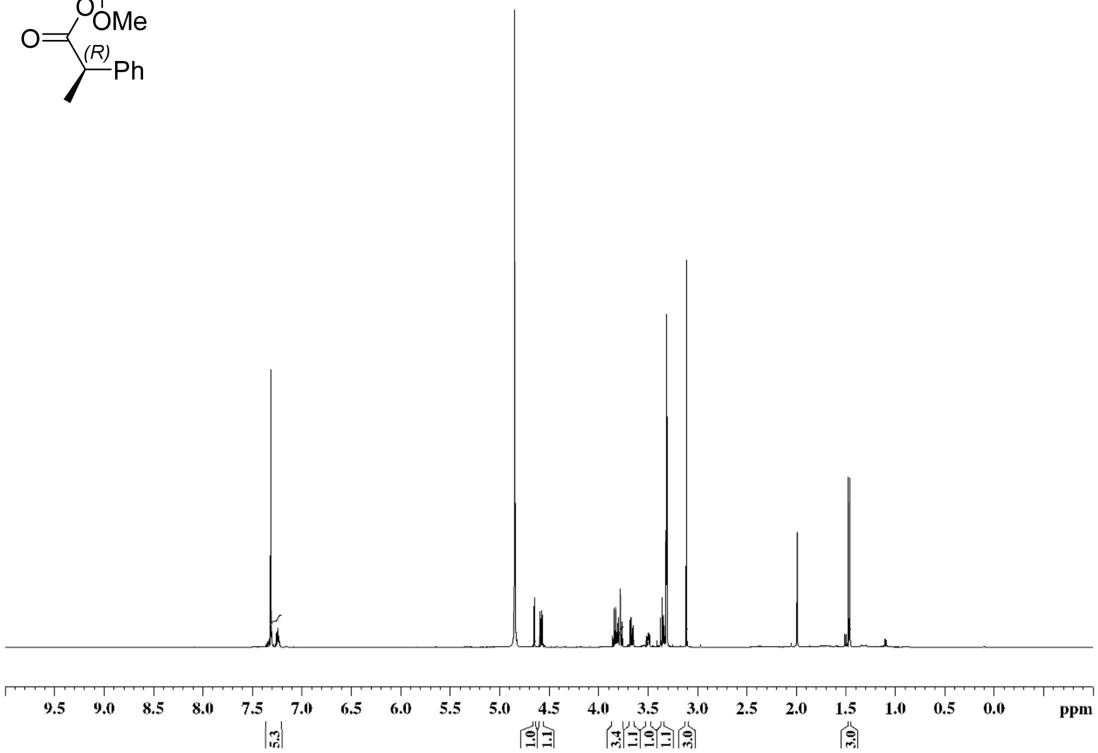

^{13}C NMR (125.8 MHz, MeOD, 25°C):

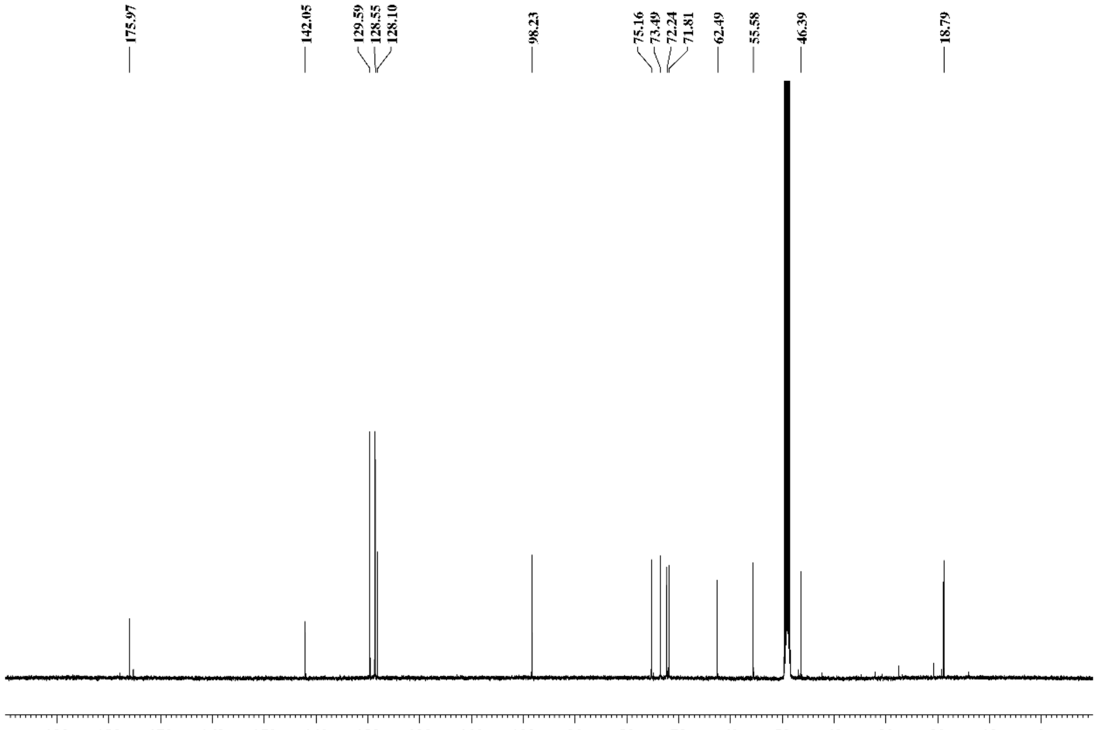

Methyl 2-*O*-(*S*)-2-phenyl-propanoyl-4,6-*O*-benzylidene-α-D-glucopyranoside (61):

^{1}H NMR (500.20 MHz, CDCl$_3$, 25°C):

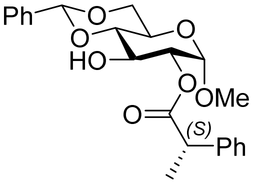

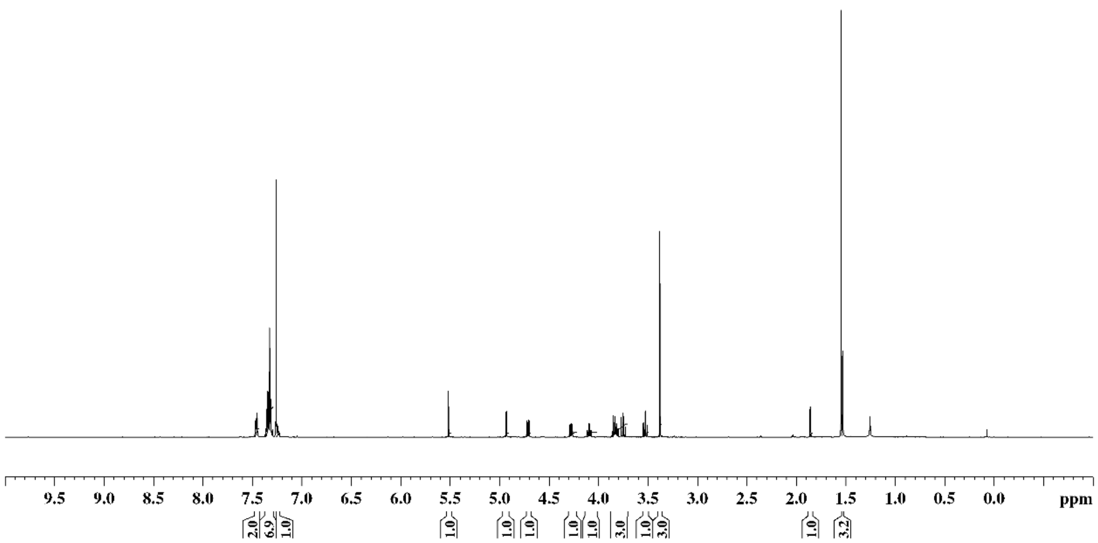

^{13}C NMR (125.8 MHz, CDCl$_3$, 25°C):

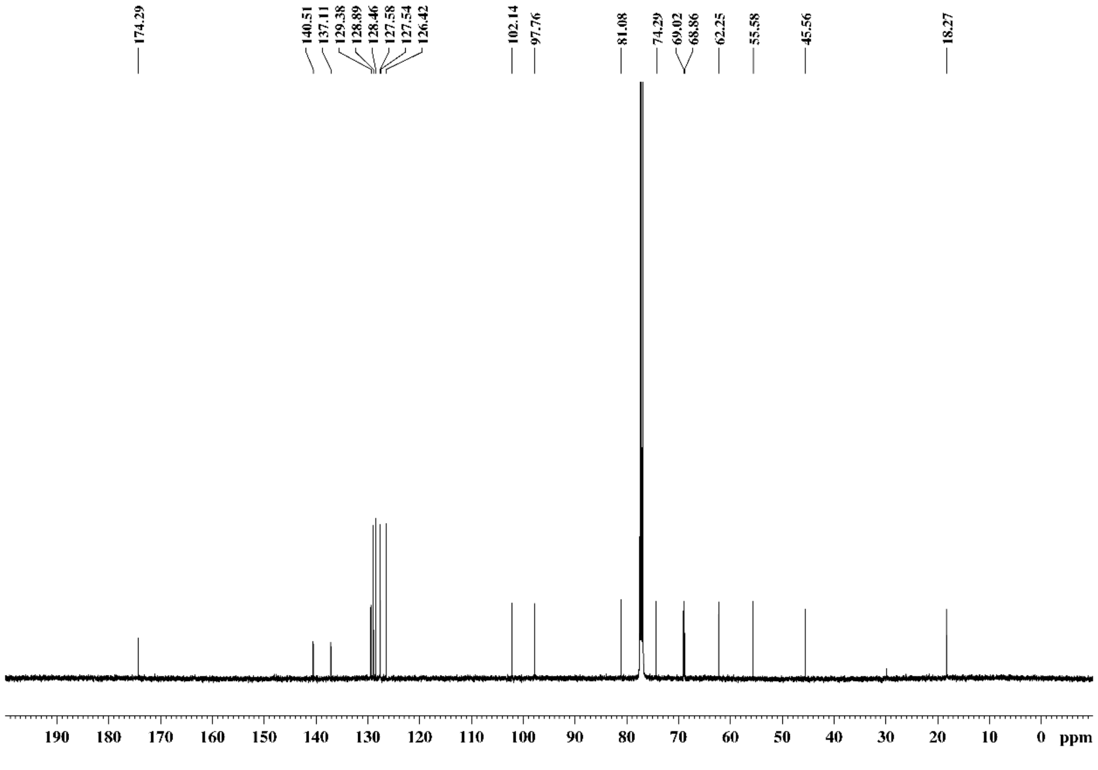

Methyl 2-*O*-(*S*)-2-phenyl-propanoyl-α-D-glucopyranoside (5):

^{1}H NMR (500.20 MHz, MeOD, 25°C):

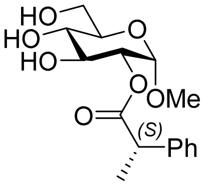

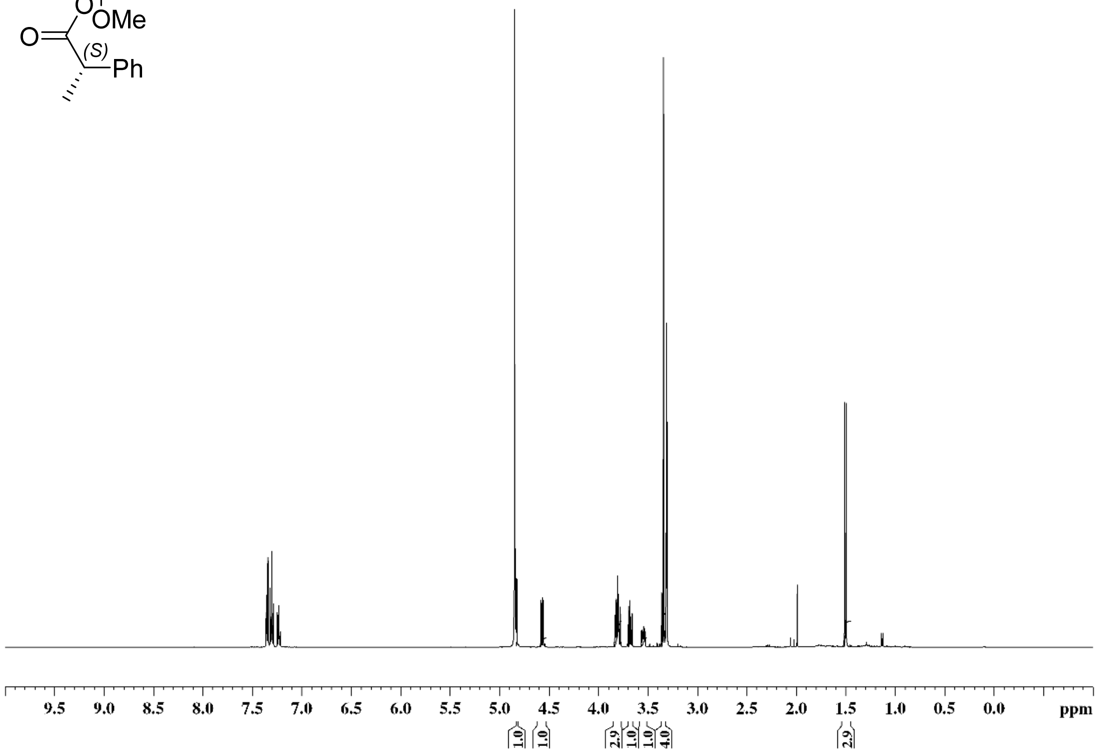

^{13}C NMR (125.8 MHz, MeOD, 25°C):

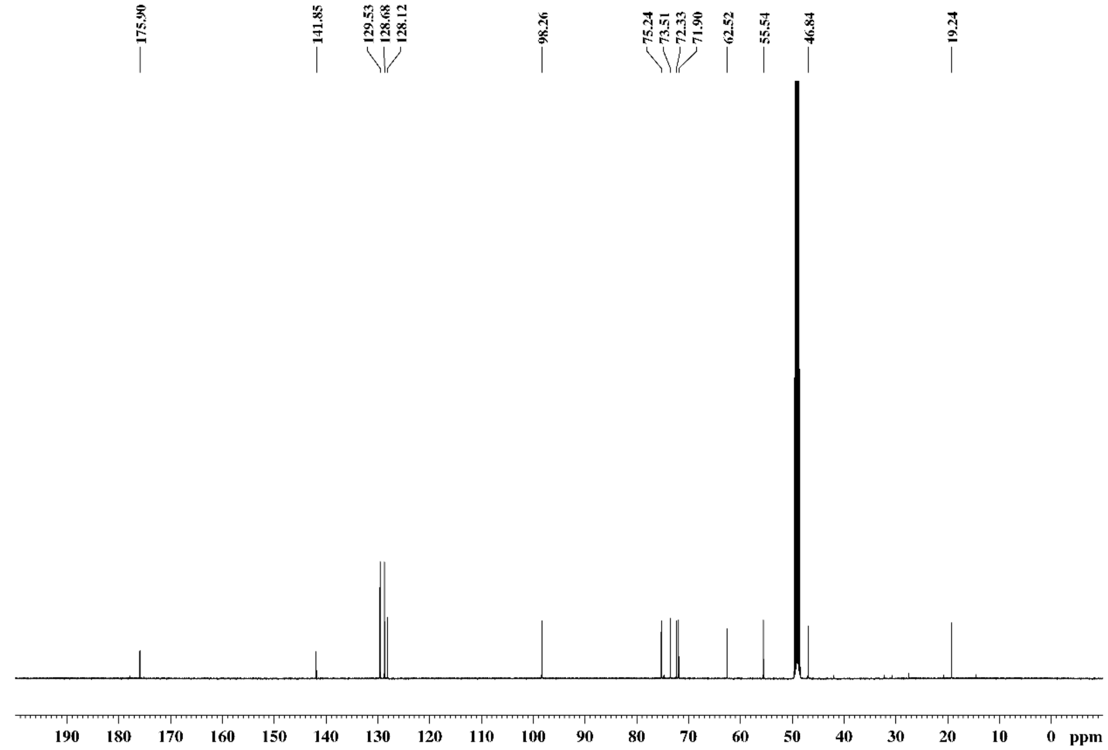

Methyl 2-*O*-acetyl-4,6-*O*-benzylidene-β-D-glucopyranoside (63):

^{1}H NMR (500.20 MHz, $CDCl_3$, 25°C):

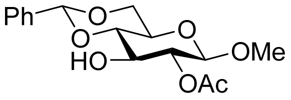

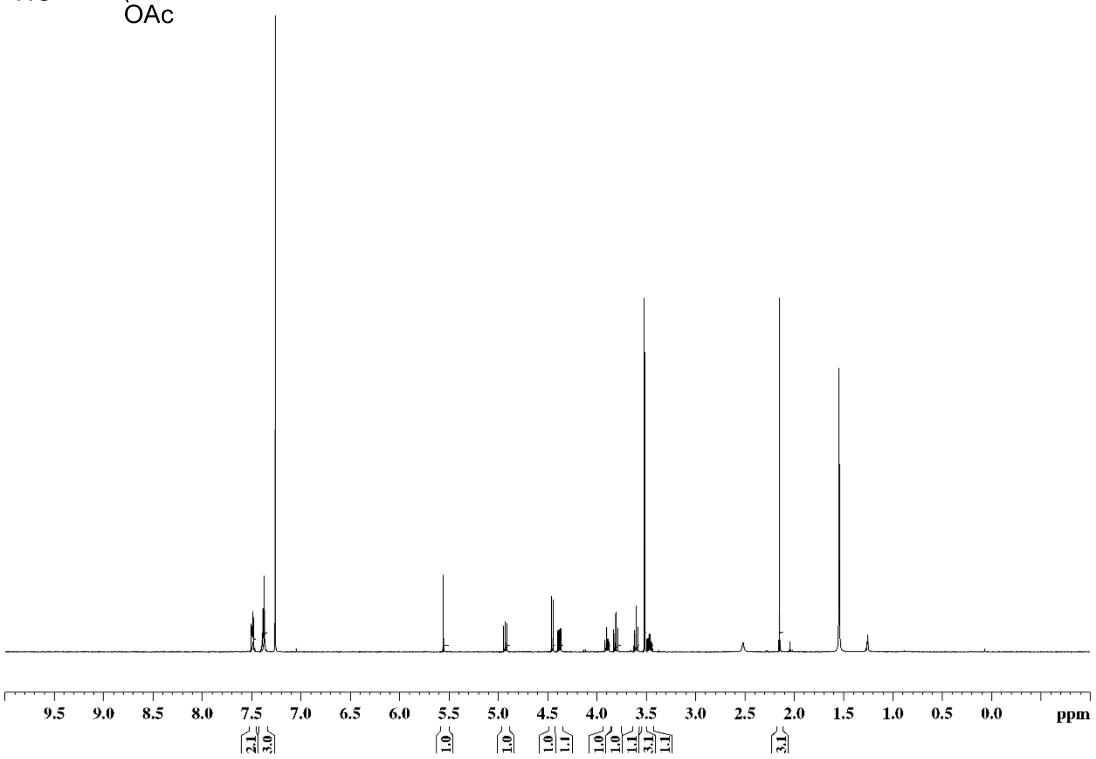

^{13}C NMR (125.8 MHz, $CDCl_3$, 25°C):

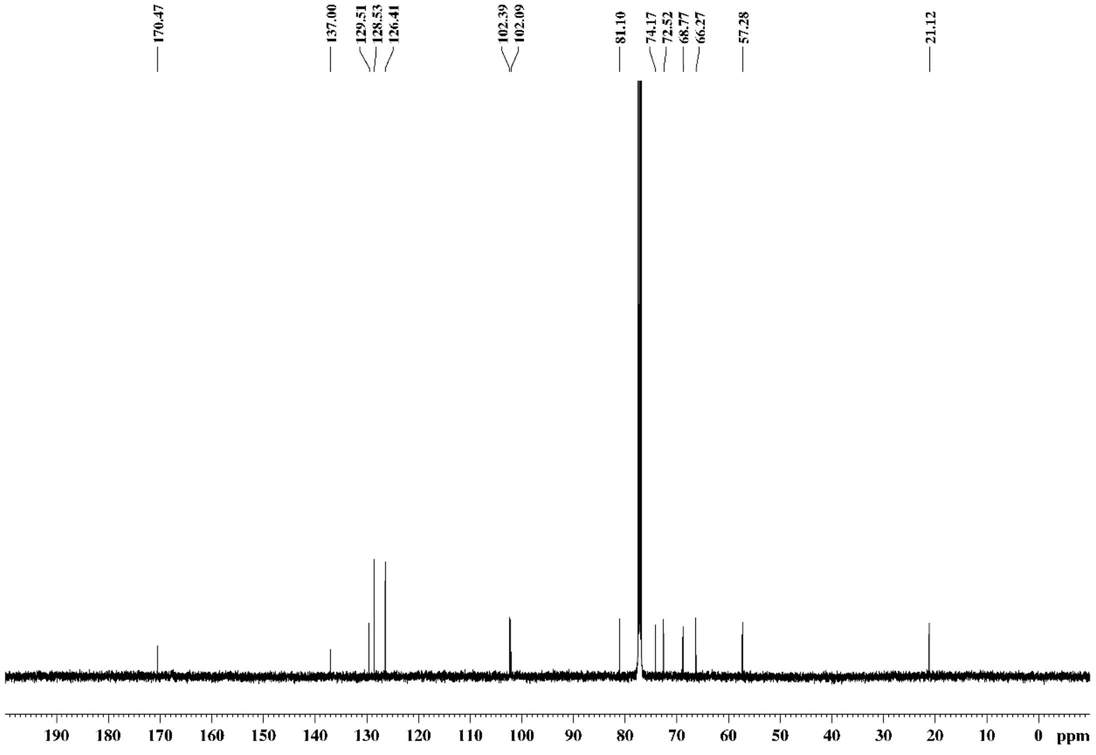

Methyl 2-*O*-acetyl-β-D-glucopyranoside (6):

^{1}H NMR (500.20 MHz, MeOD, 25°C):

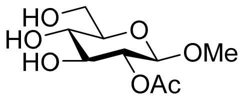

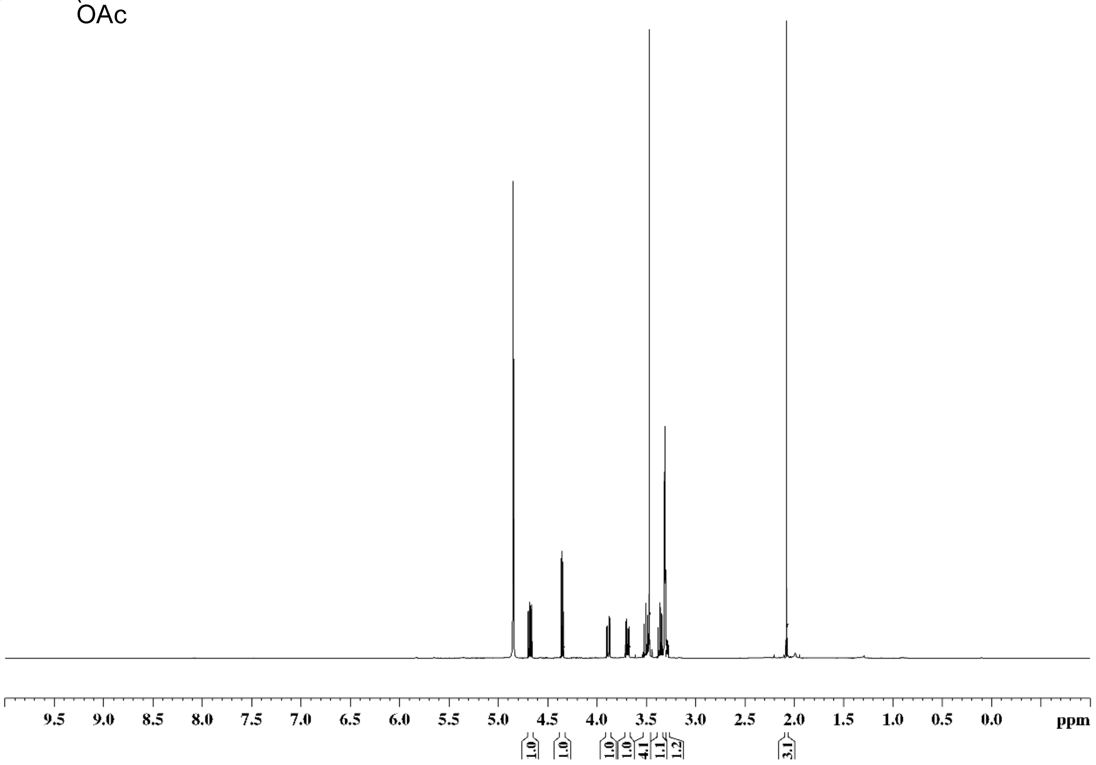

^{13}C NMR (125.8 MHz, MeOD, 25°C):

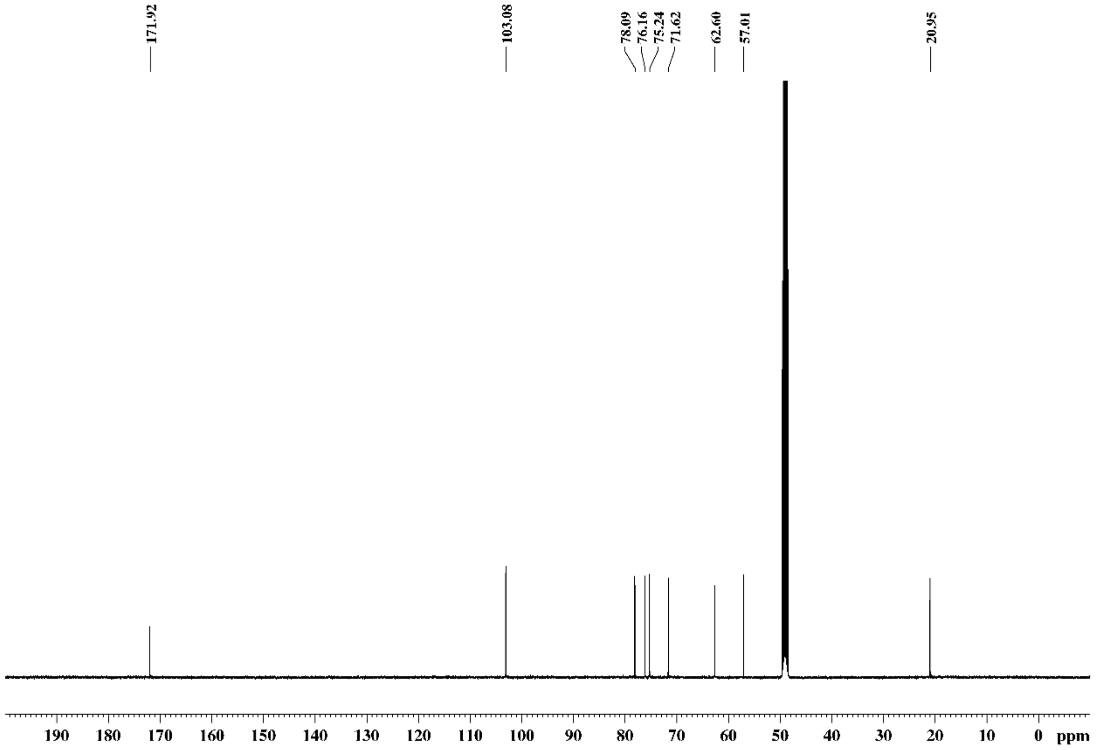

Methyl 2-*O*-benzoyl-4,6-*O*-benzylidene-β-D-glucopyranoside (64):

^{1}H NMR (500.20 MHz, CDCl$_3$, 25°C):

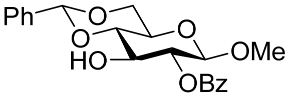

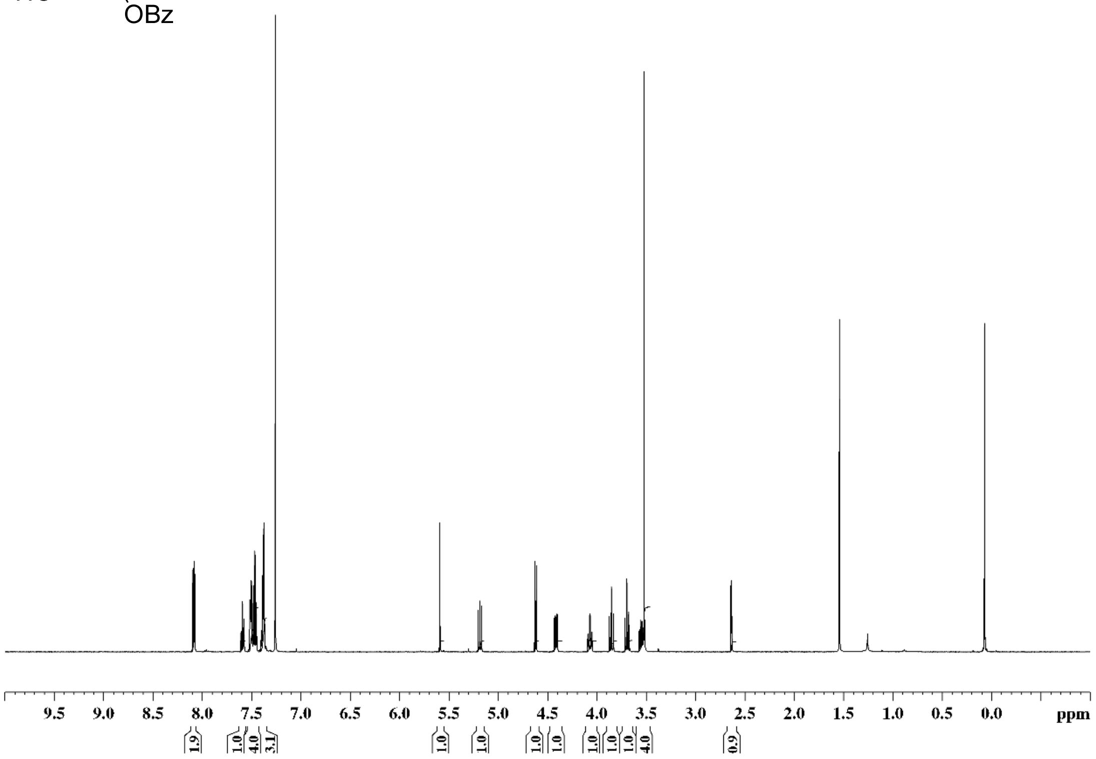

^{13}C NMR (125.8 MHz, CDCl$_3$, 25°C):

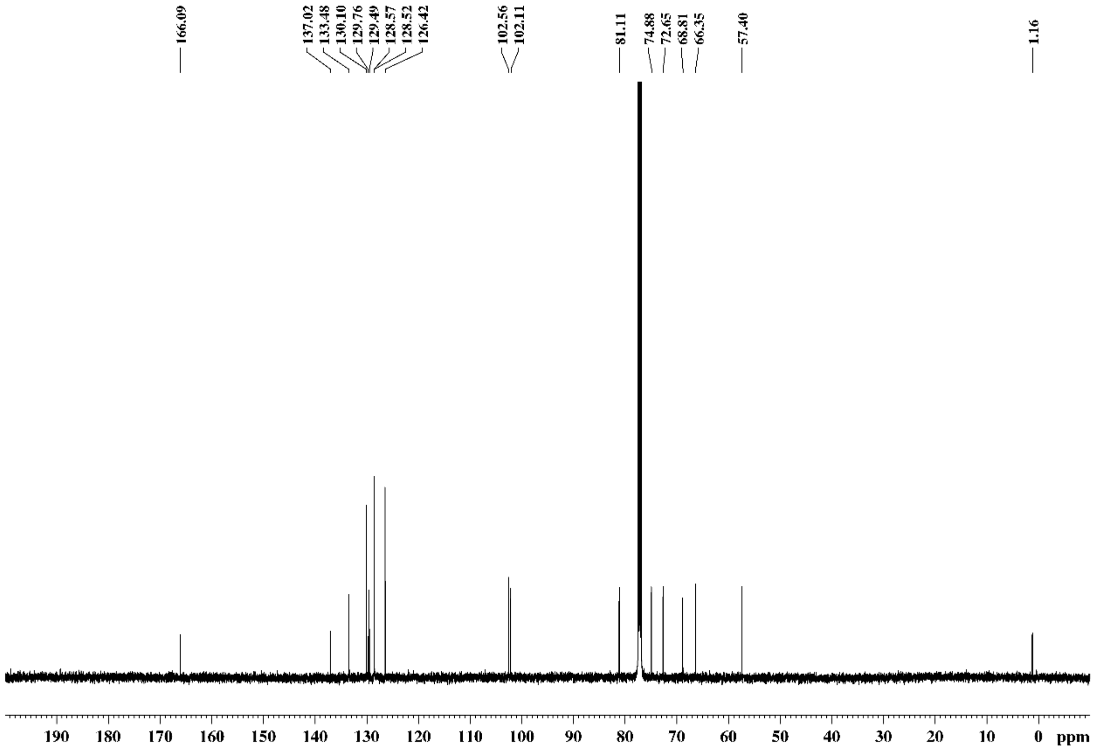

Methyl 2-*O*-benzoyl-β-D-glucopyranoside (7):

^{1}H NMR (500.20 MHz, MeOD, 25°C):

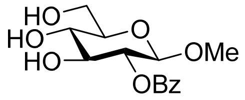

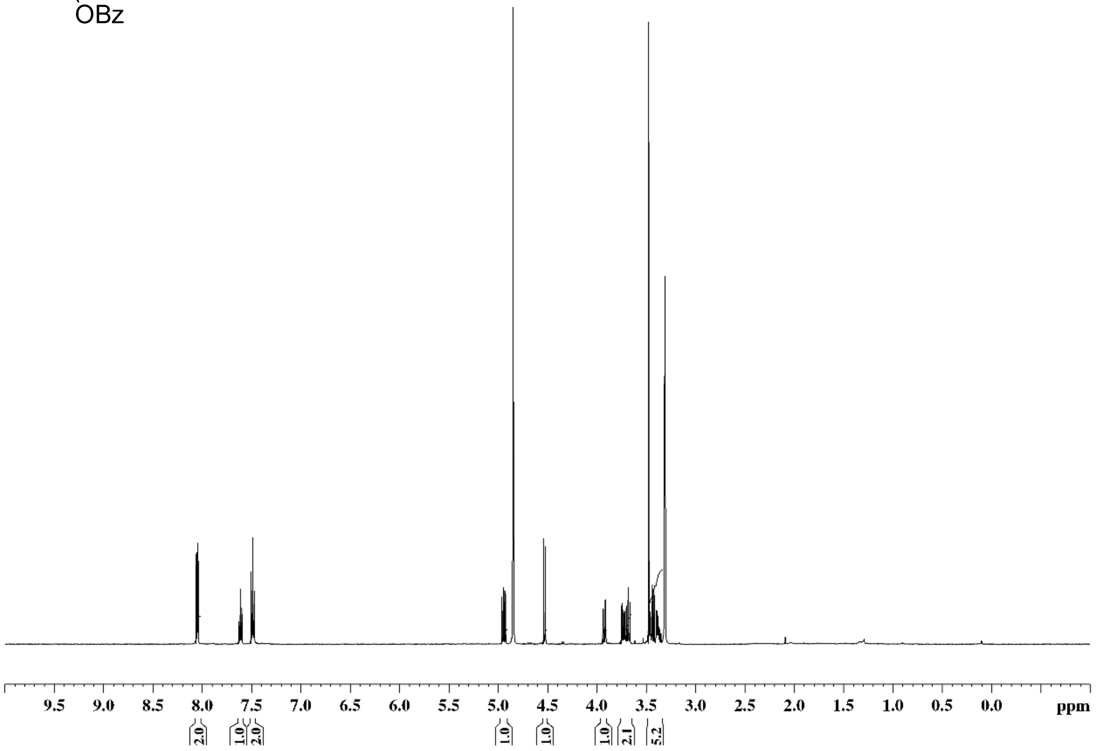

^{13}C NMR (125.8 MHz, MeOD, 25°C):

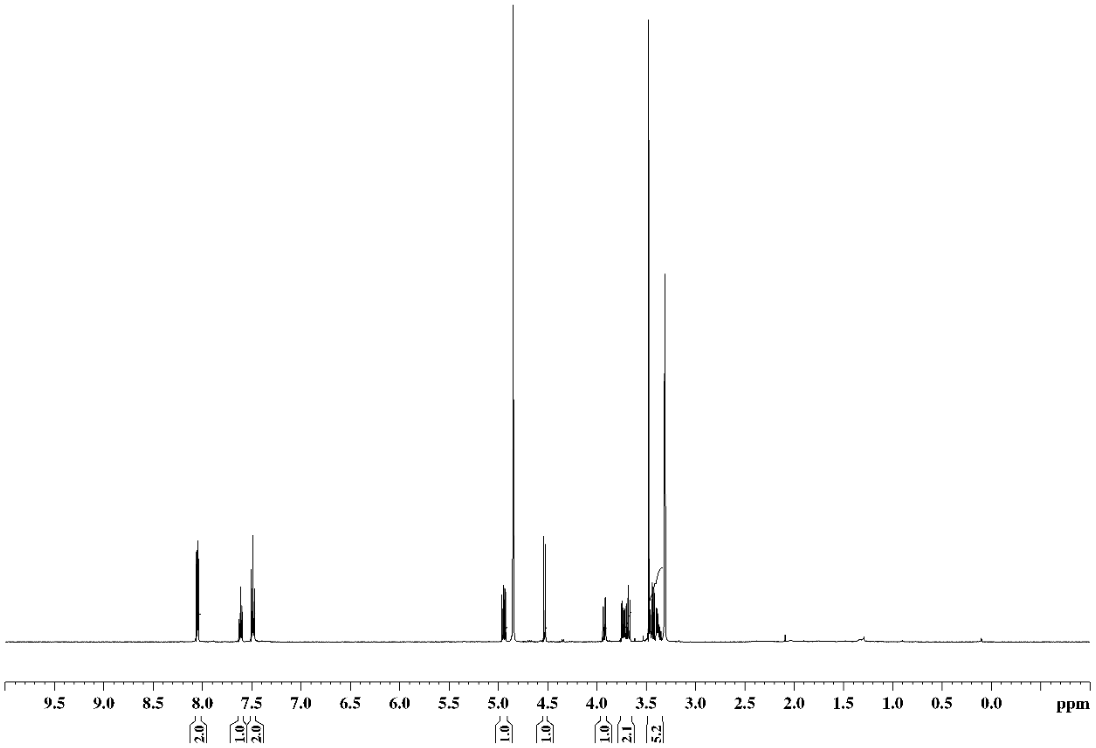

Methyl 4,6-*O*-benzylidene-2-*O*-pivaloyl-β-D-glucopyranoside (65):

^{1}H NMR (500.20 MHz, $CDCl_3$, 25°C):

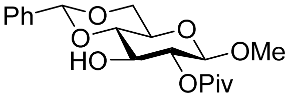

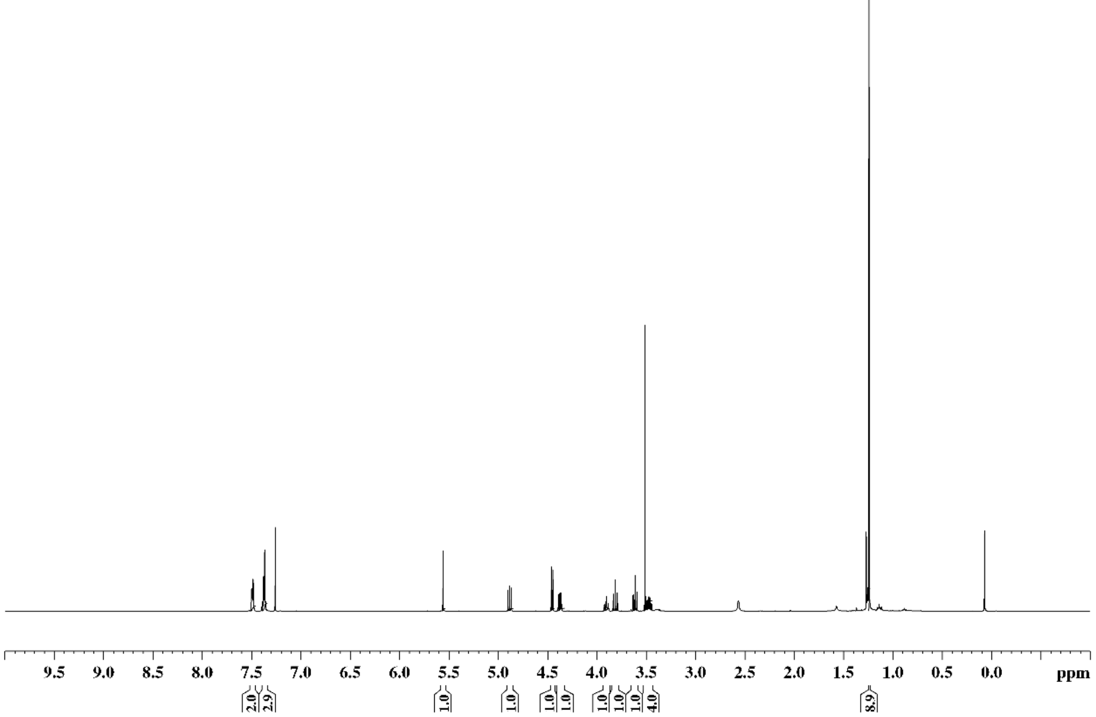

^{13}C NMR (125.8 MHz, $CDCl_3$, 25°C):

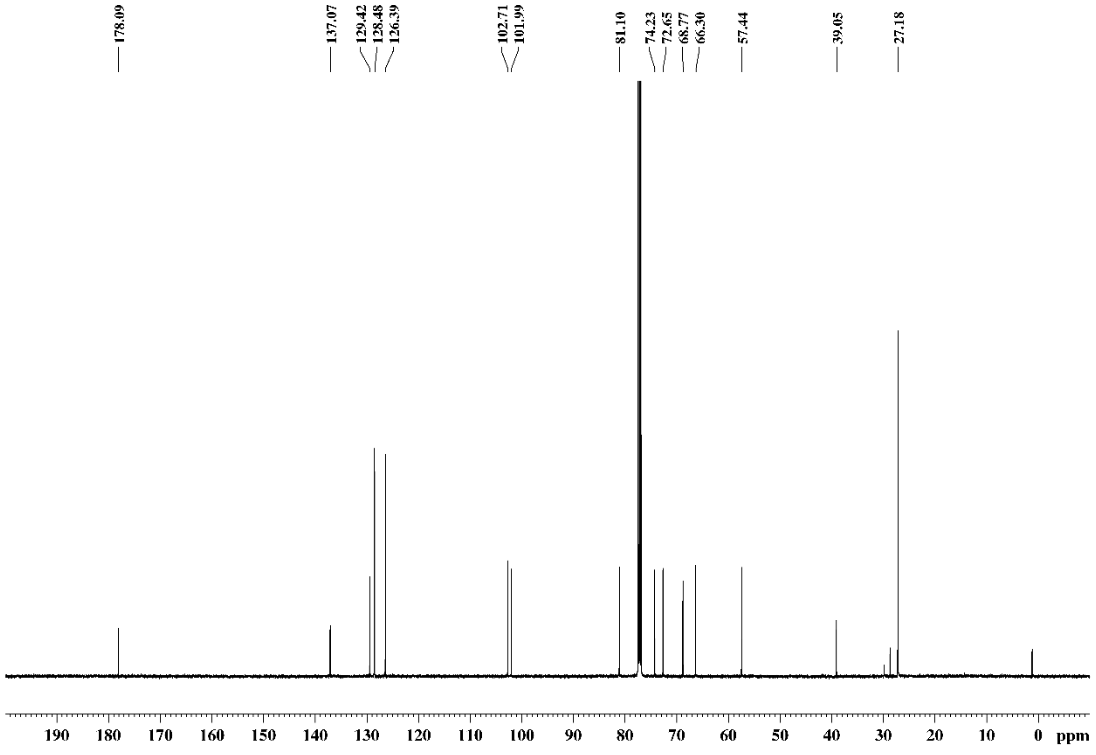

Methyl 2-*O*-pivaloyl-β-D-glucopyranoside (8):

^{1}H NMR (500.20 MHz, MeOD, 25°C):

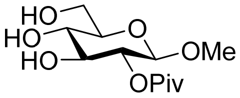

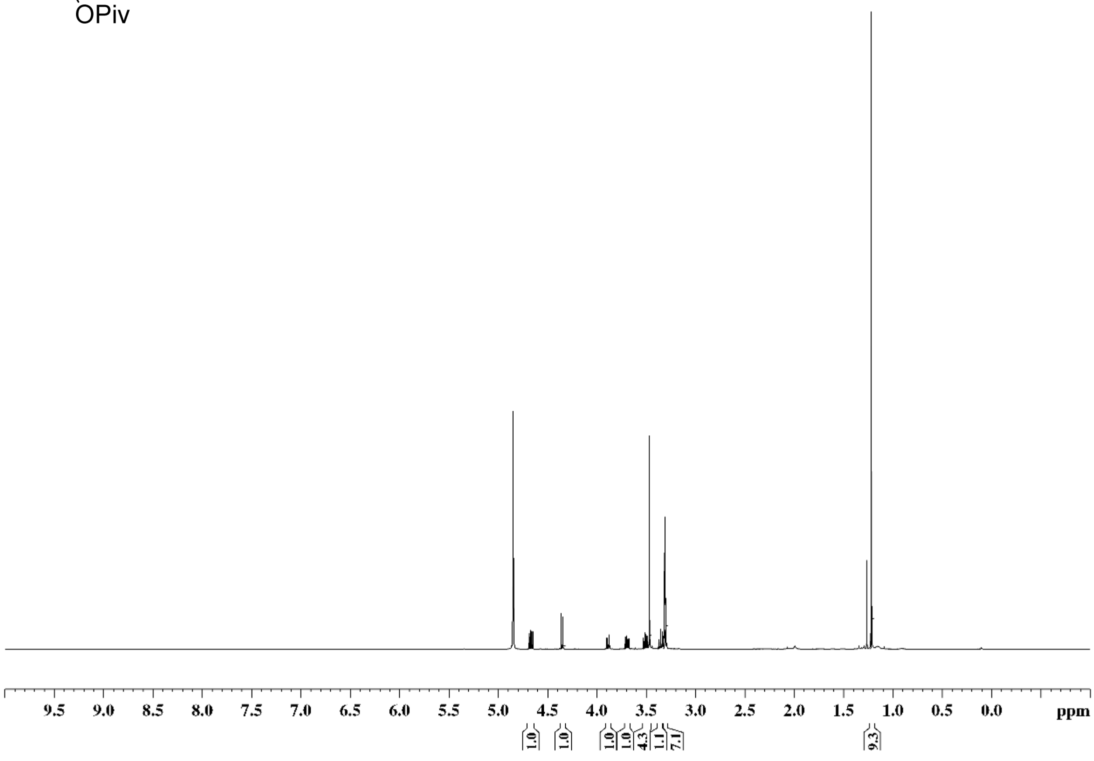

^{13}C NMR (125.8 MHz, MeOD, 25°C):

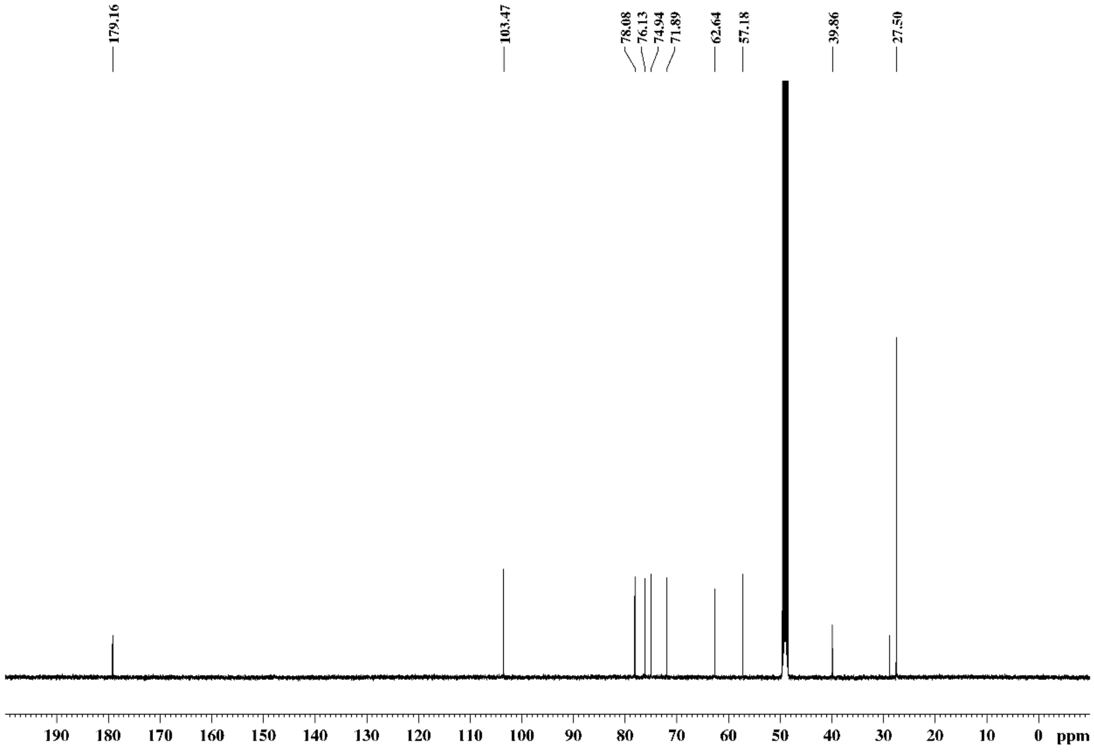

Methyl 2-*O*-(*R*)-2-phenyl-propanoyl-4,6-*O*-benzylidene-β-D-glucopyranoside (66):

^{1}H NMR (500.20 MHz, $CDCl_3$, 25°C):

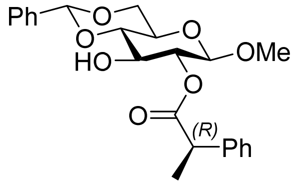

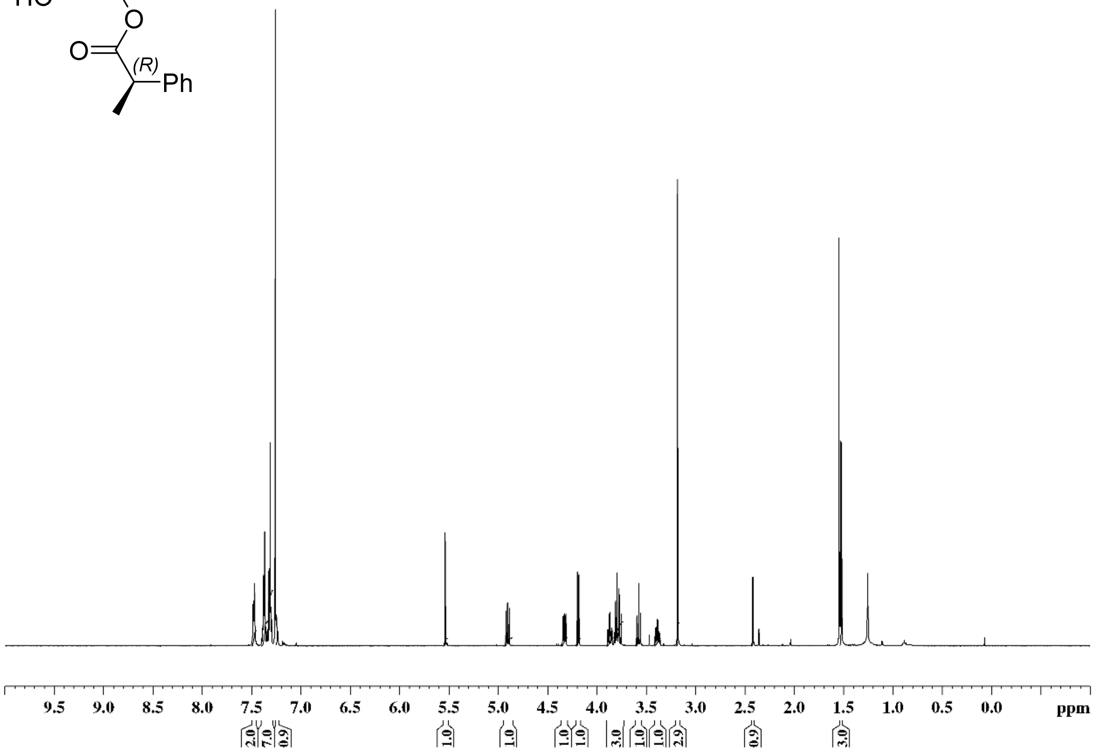

^{13}C NMR (125.8 MHz, $CDCl_3$, 25°C):

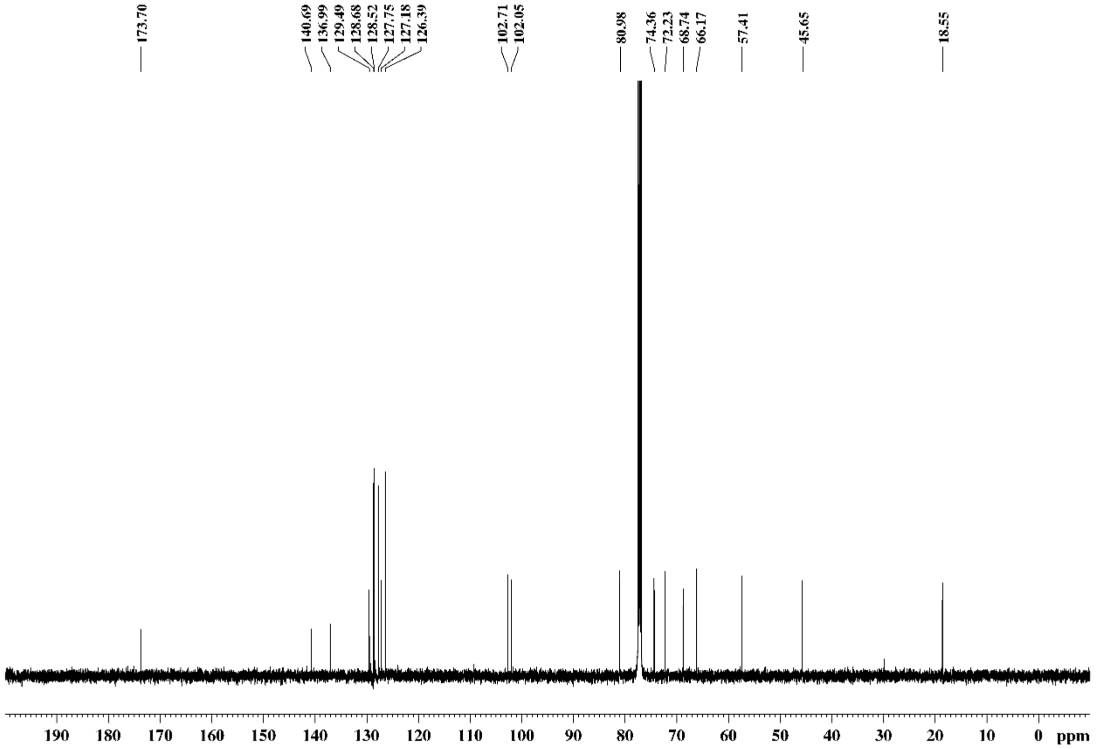

Methyl 2-*O*-(*R*)-2-phenyl-propanoyl-β-D-glucopyranoside (4):

^{1}H NMR (500.20 MHz, MeOD, 25°C):

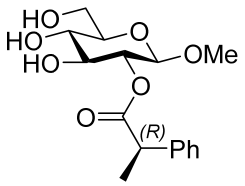

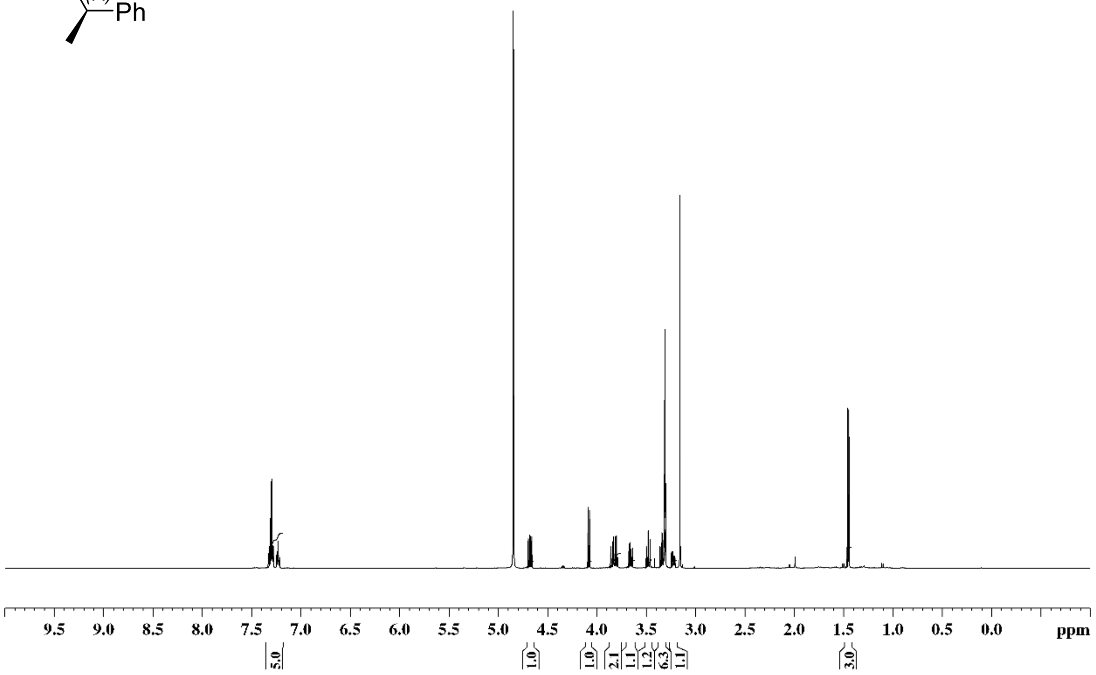

^{13}C NMR (125.8 MHz, MeOD, 25°C):

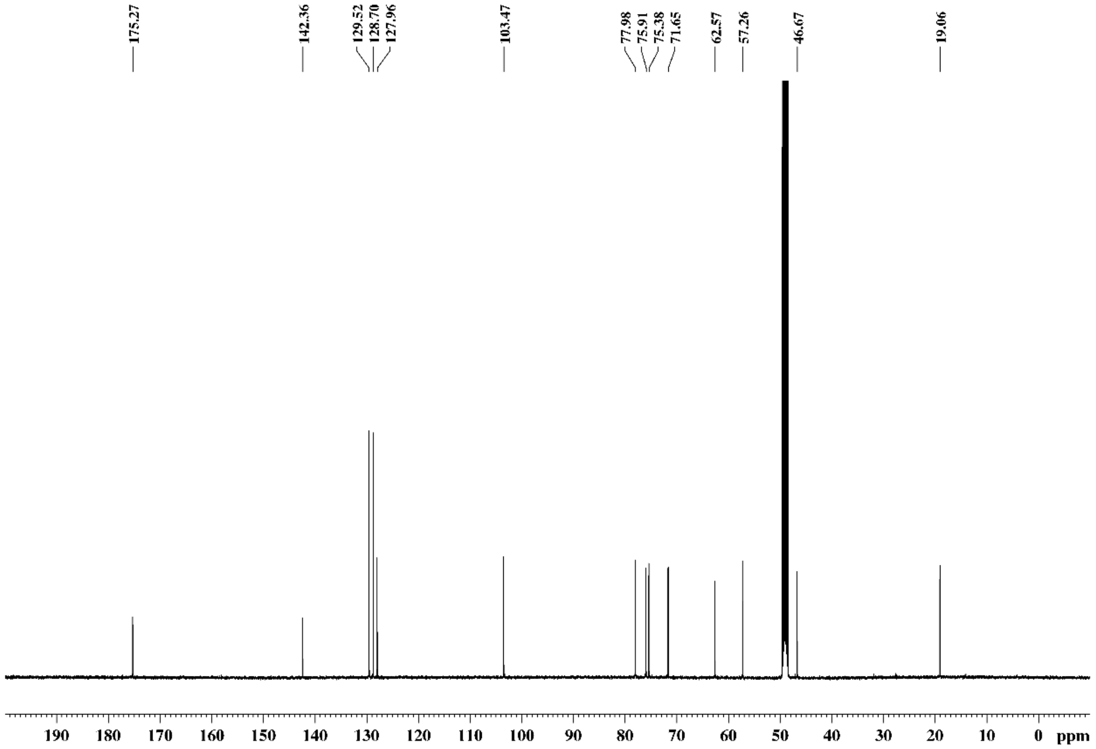

Methyl 2-*O*-(*S*)-2-phenyl-propanoyl-4,6-*O*-benzylidene-β-D-glucopyranoside (67):

^{1}H NMR (500.20 MHz, $CDCl_3$, 25°C):

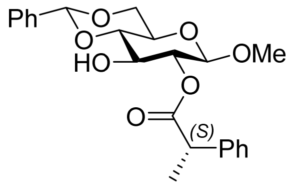

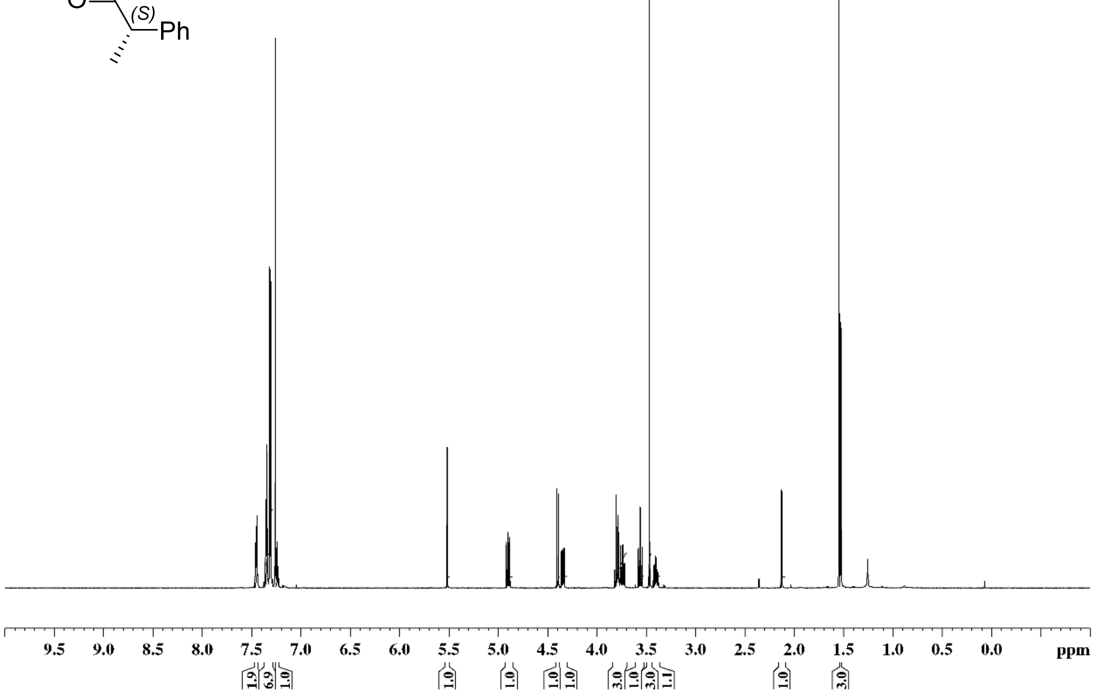

^{13}C NMR (125.8 MHz, $CDCl_3$, 25°C):

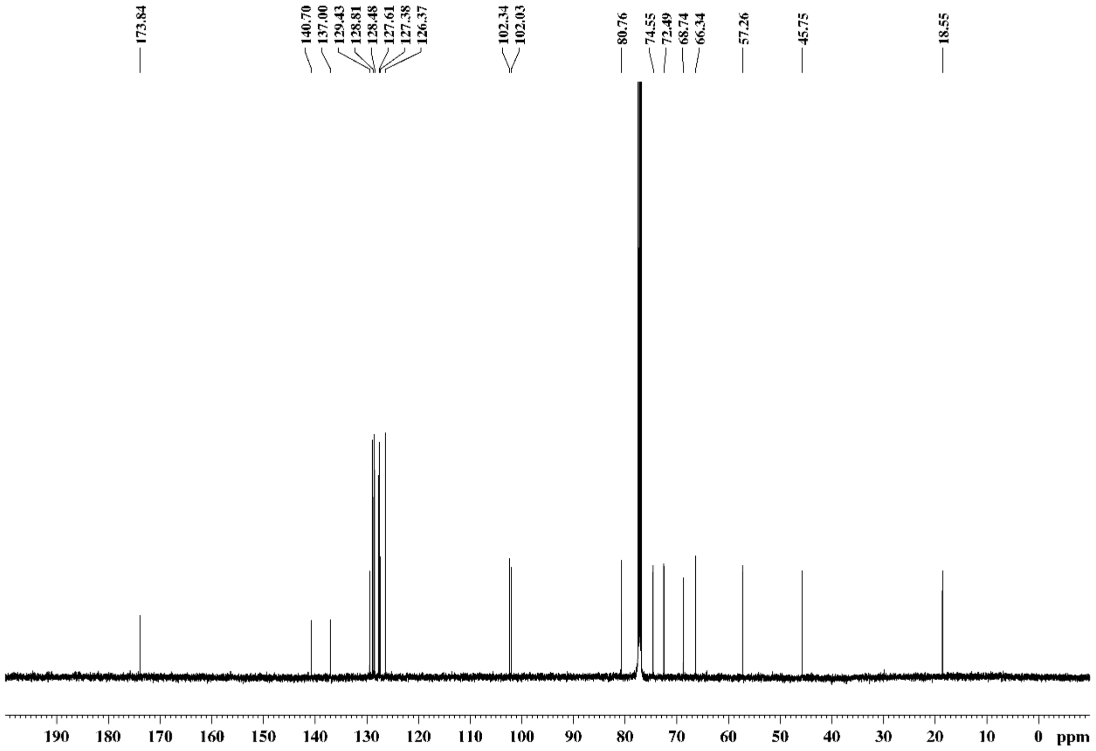

Methyl 2-*O*-(*S*)-2-phenyl-propanoyl-β-D-glucopyranoside (5):

^{1}H NMR (500.20 MHz, MeOD, 25°C):

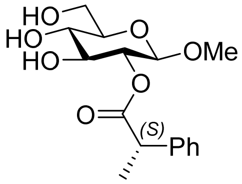

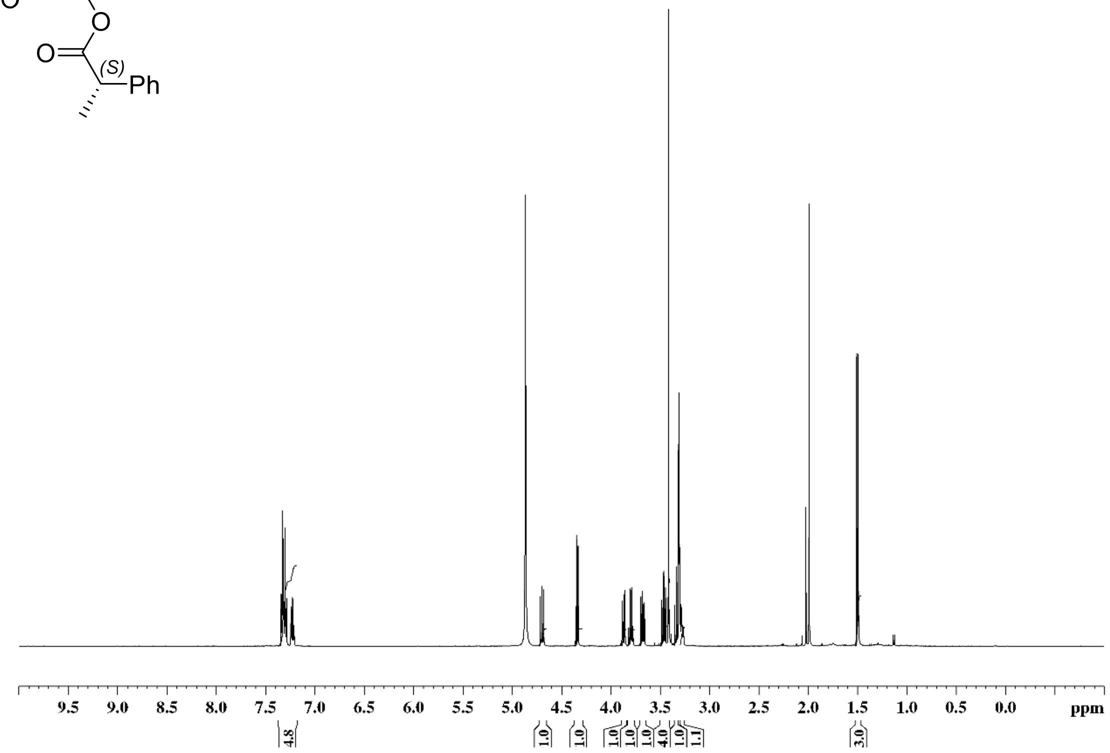

^{13}C NMR (125.8 MHz, MeOD, 25°C):

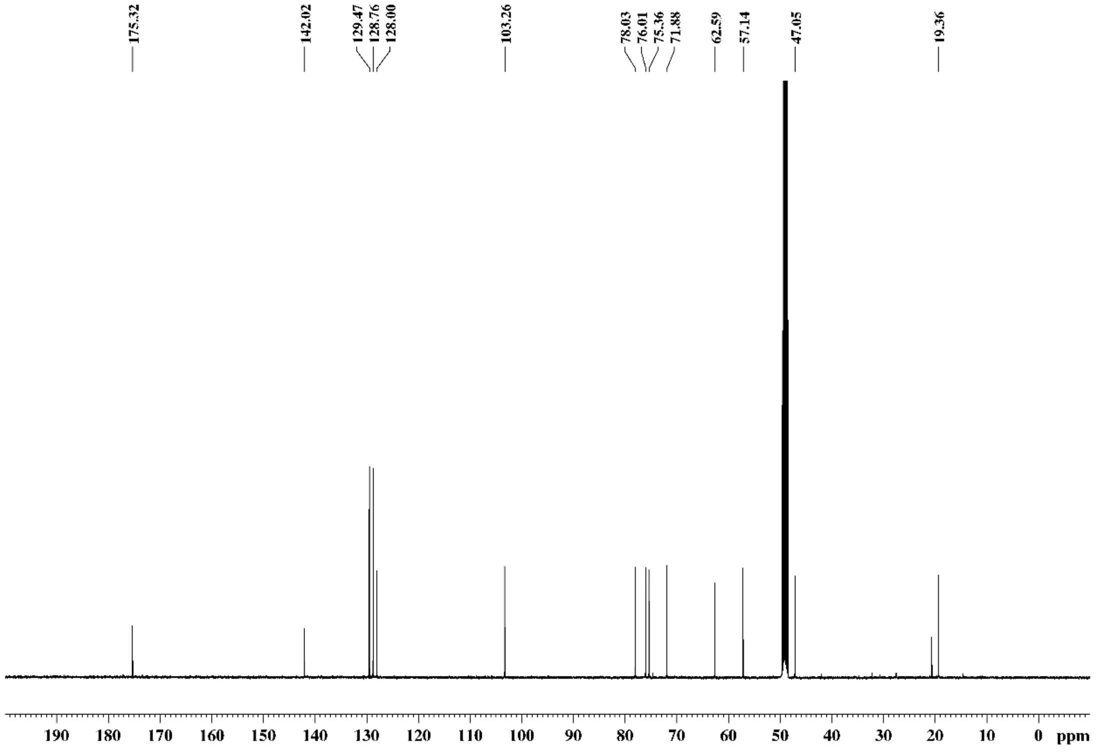

Methyl 2-*O*-acetyl-α-D-galactopyranoside (11):

^{1}H NMR (500.20 MHz, MeOD, 25°C):

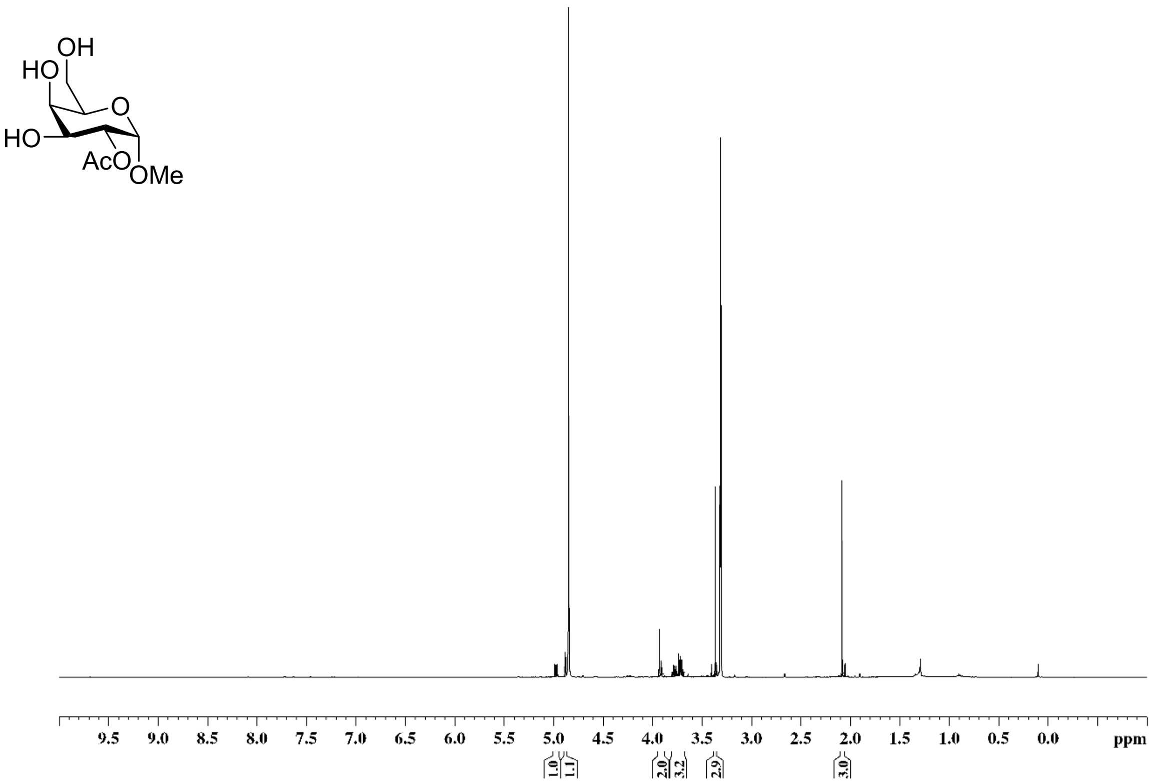

^{13}C NMR (125.8 MHz, MeOD, 25°C):

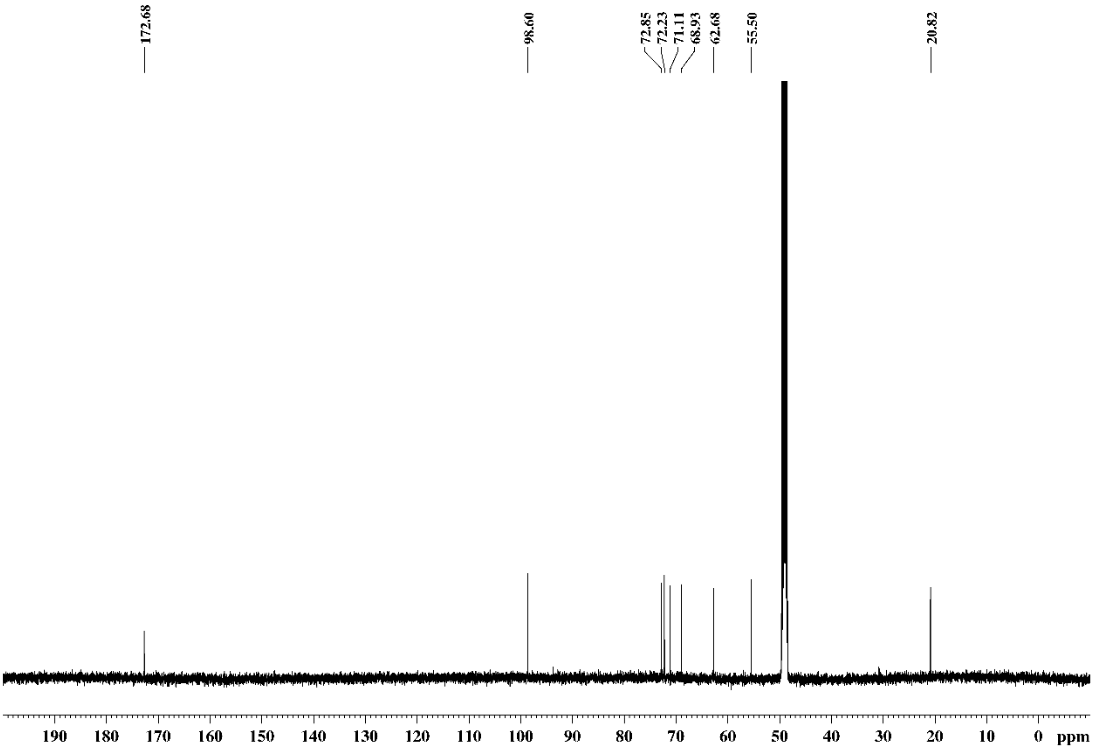

Methyl 2-*O*-benzoyl-4,3-*O*-isopropylidene-6-(2-methoxyisopropyl)-α-D-galactopyranoside (70):

^{1}H NMR (500.20 MHz, $CDCl_3$, 25°C):

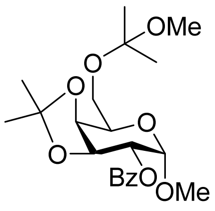

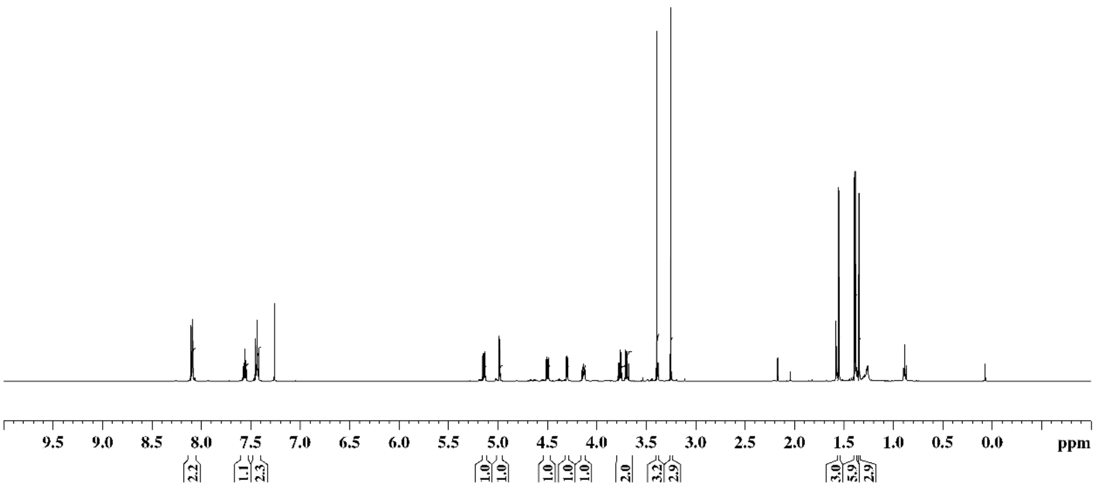

^{13}C NMR (125.8 MHz, $CDCl_3$, 25°C):

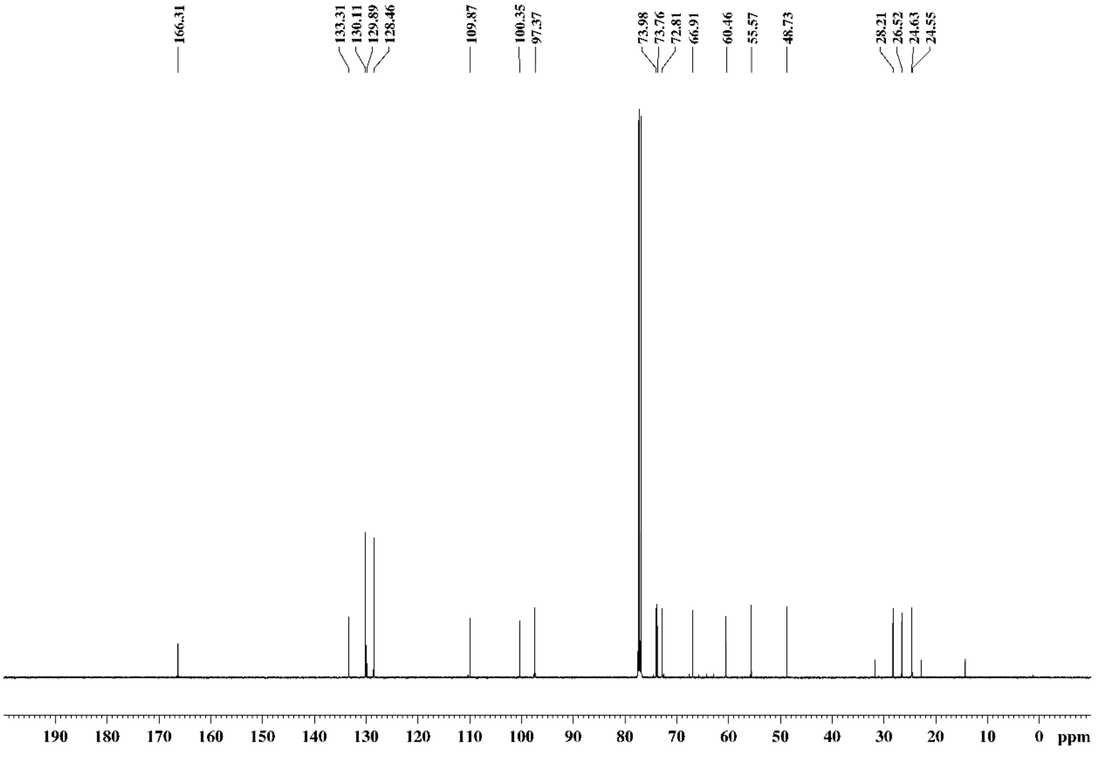

Methyl 2-*O*-benzoyl-α-D-galactopyranoside (12):

^{1}H NMR (500.20 MHz, MeOD, 25°C):

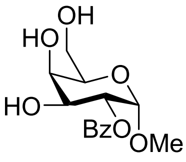

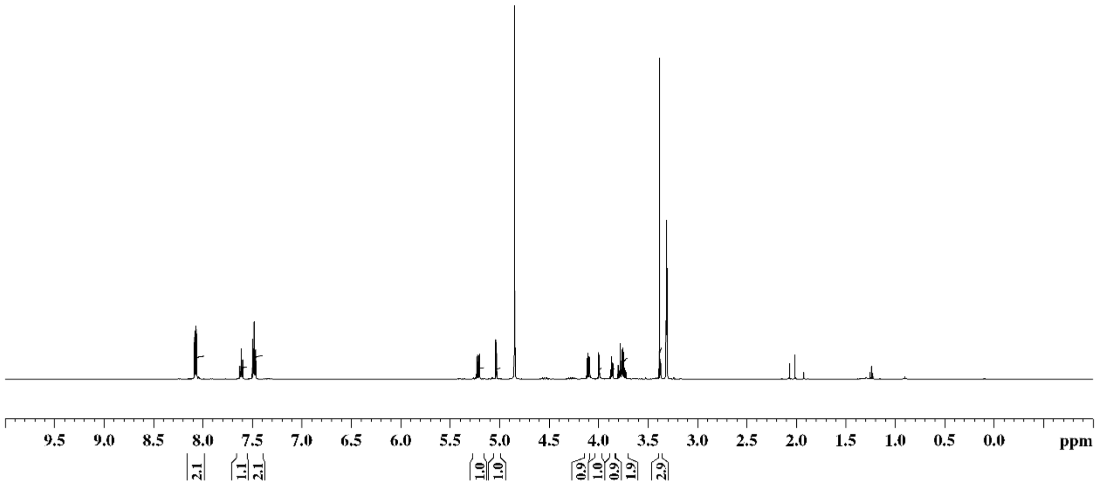

^{13}C NMR (125.8 MHz, MeOD, 25°C):

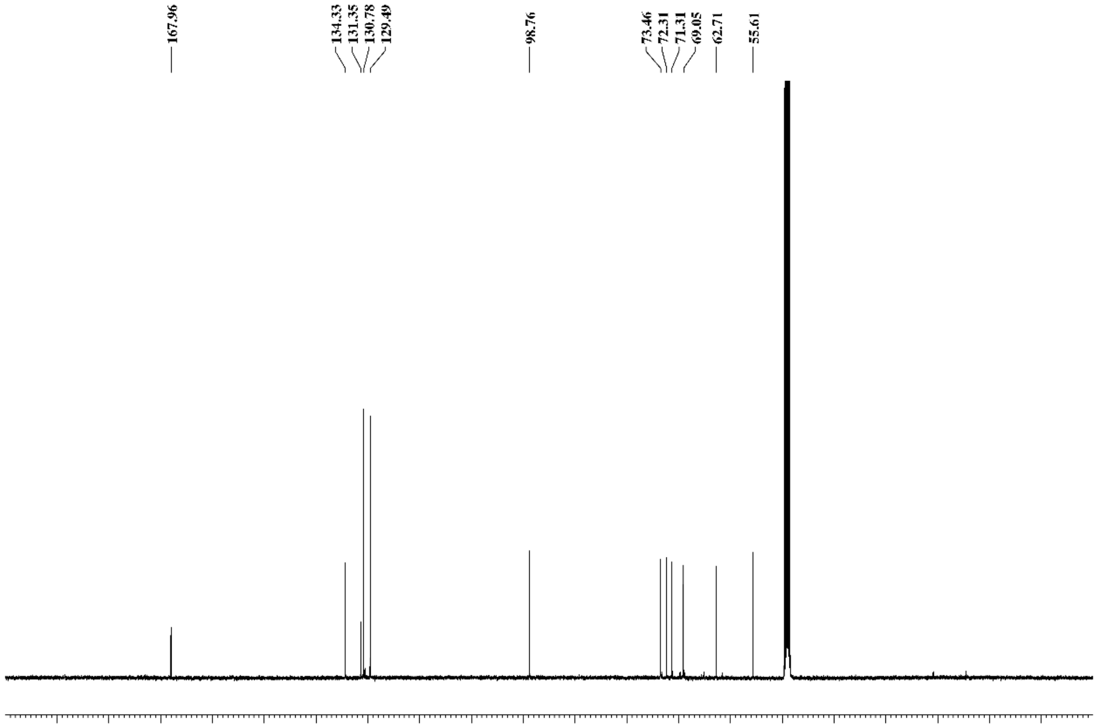

Methyl 4,3-*O*-isopropylidene-6-(2-methoxyisopropyl)-2-*O*-pivaloyl-α-D-galactopyranoside (71):

^{1}H NMR (500.20 MHz, $CDCl_3$, 25°C):

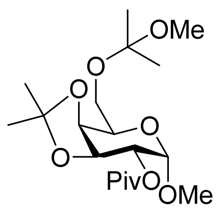

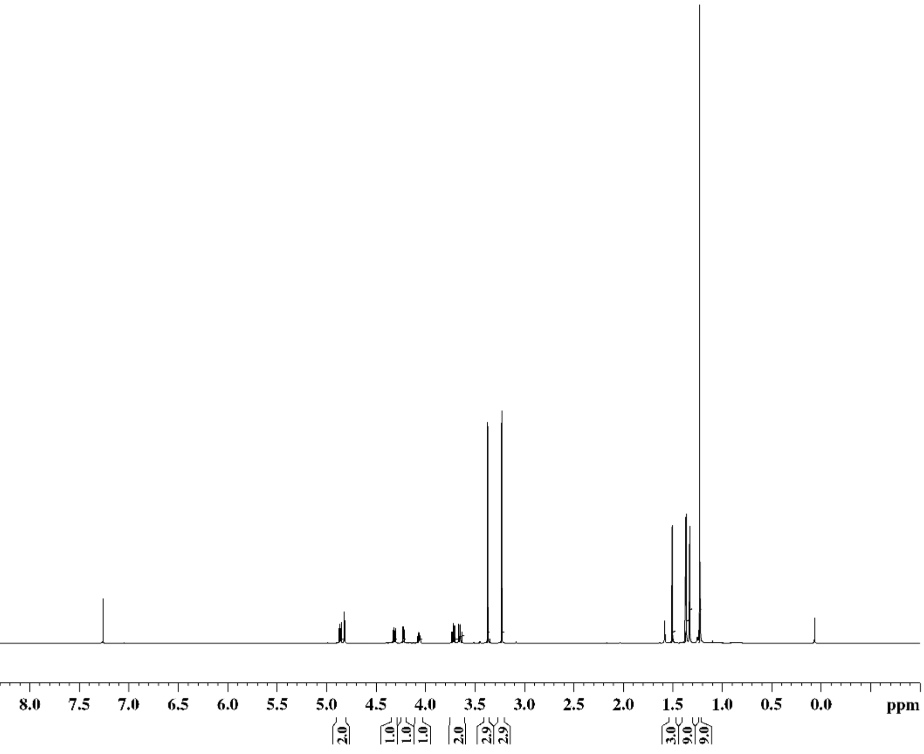

^{13}C NMR (125.8 MHz, $CDCl_3$, 25°C):

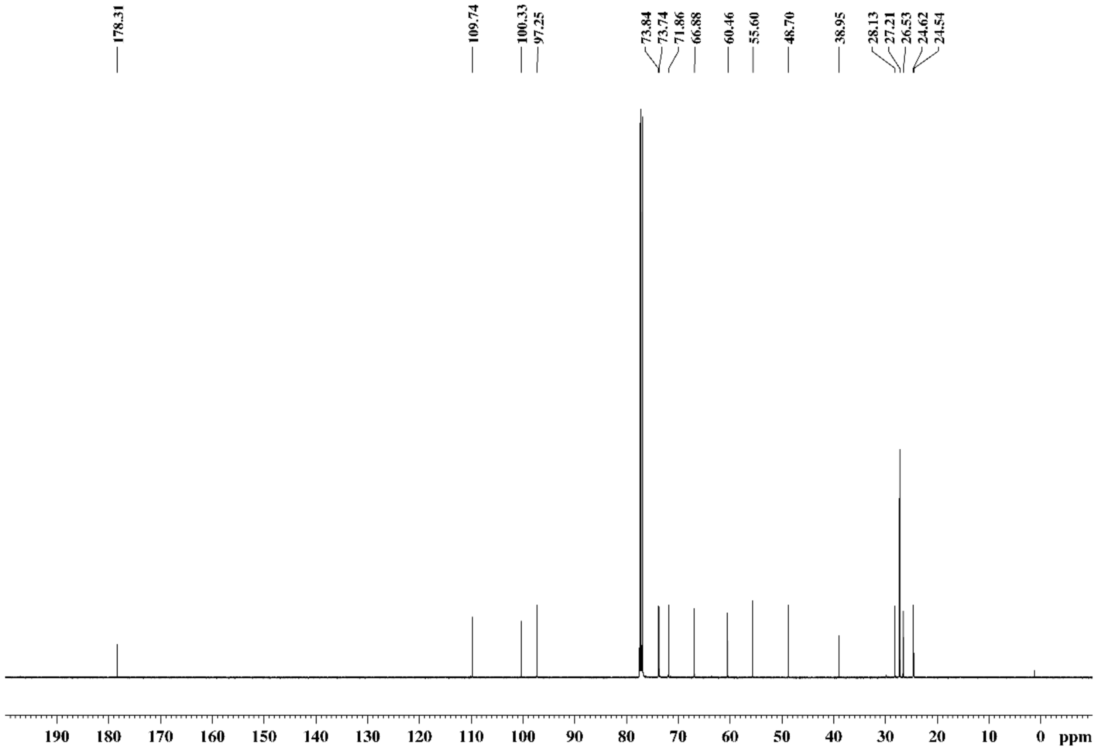

Methyl 2-*O*-pivaloyl-α-D-galactopyranoside (13):

^{1}H NMR (500.20 MHz, MeOD, 25°C):

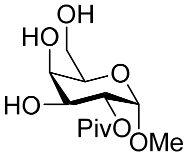

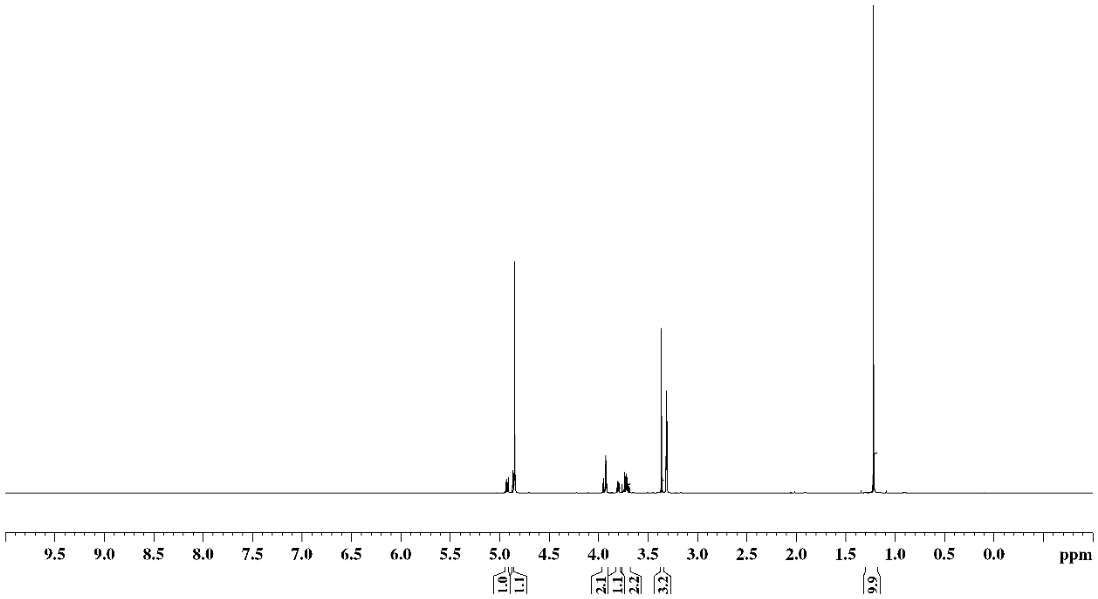

^{13}C NMR (125.8 MHz, MeOD, 25°C):

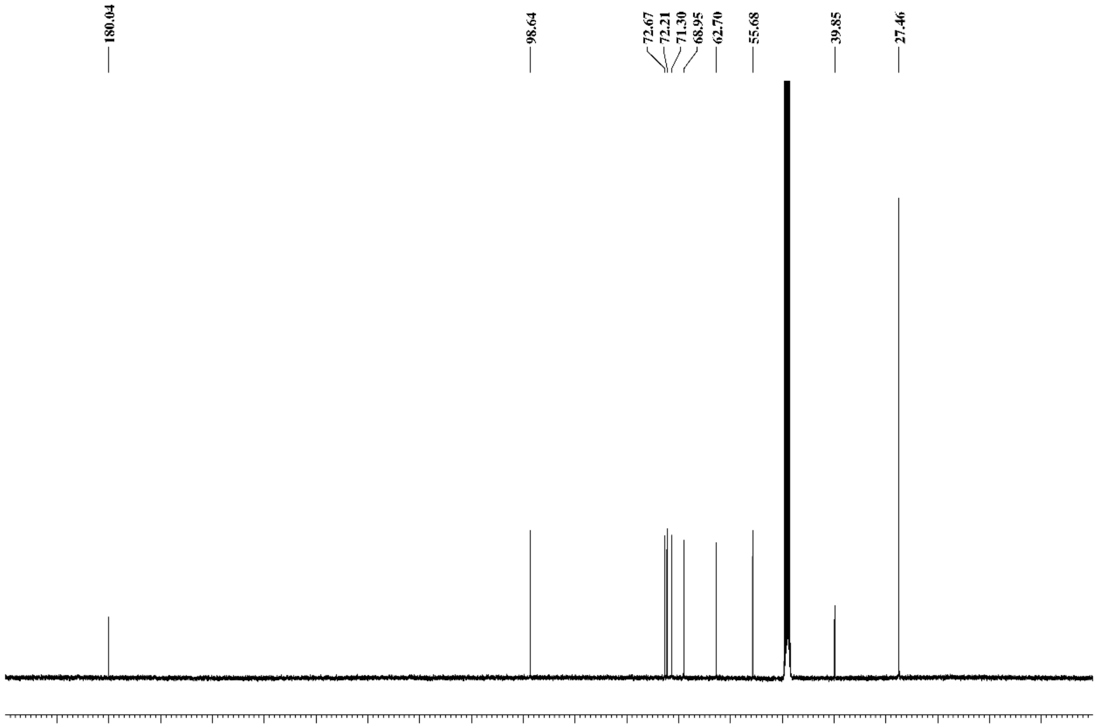

Methyl 4,3-*O*-isopropylidene-6-(2-methoxyisopropyl)-2-*O*-(*R*)-2-phenyl-propanoyl-α-D-galactopyranoside (72):

^{1}H NMR (500.20 MHz, CDCl$_3$, 25°C):

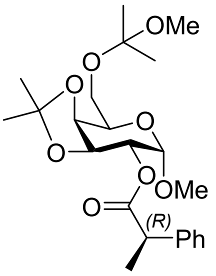

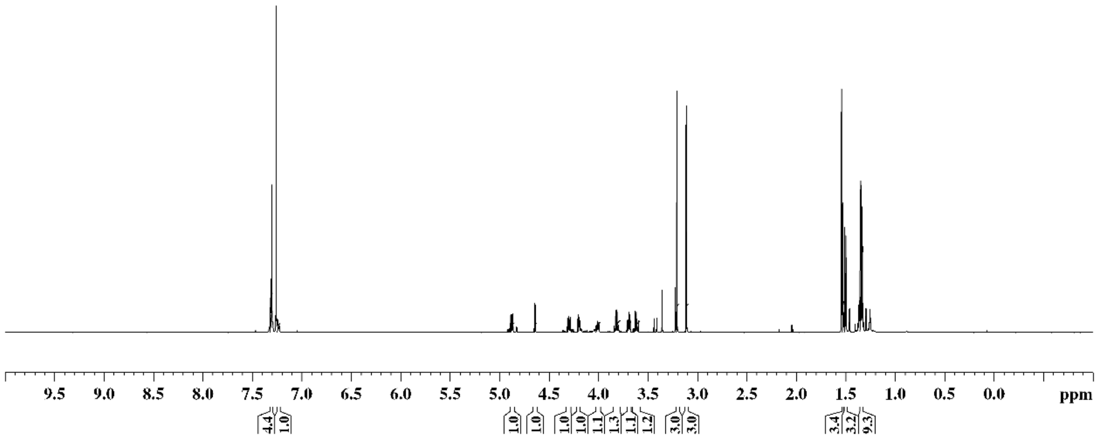

^{13}C NMR (125.8 MHz, CDCl$_3$, 25°C):

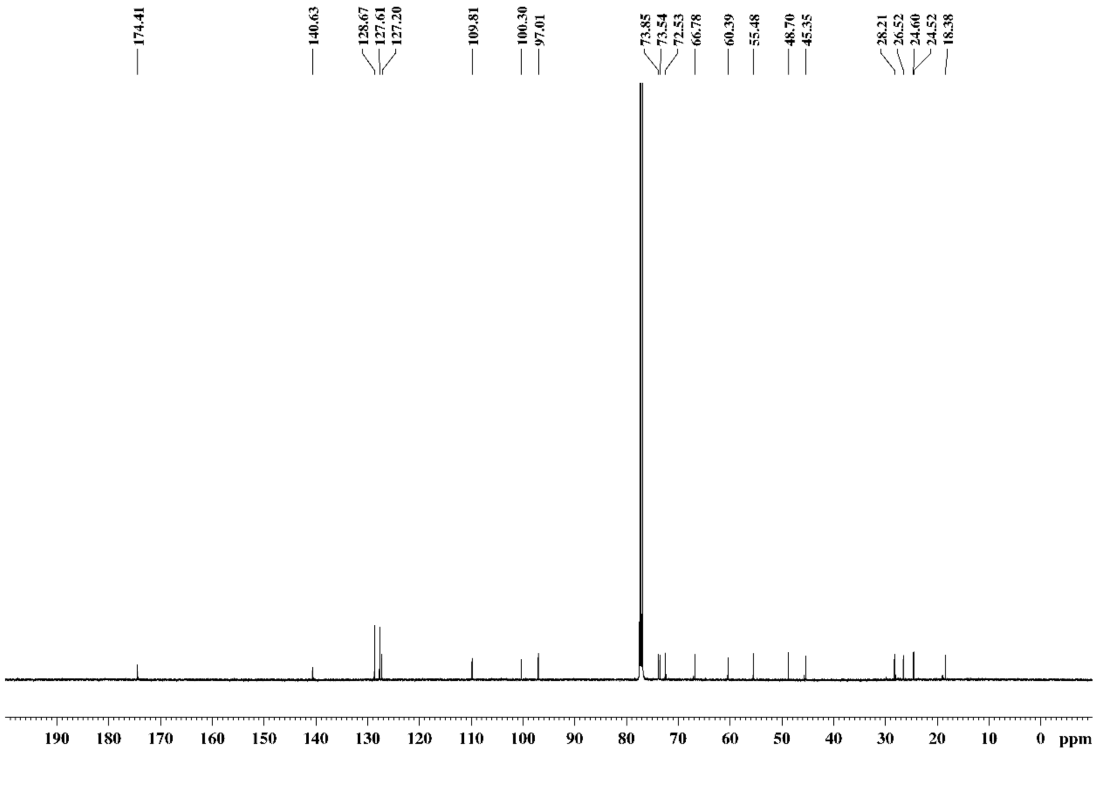

Methyl 2-*O*-(*R*)-2-phenyl-propanoyl-α-D-galactopyranoside (14):

^{1}H NMR (500.20 MHz, MeOD, 25°C):

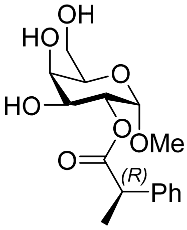

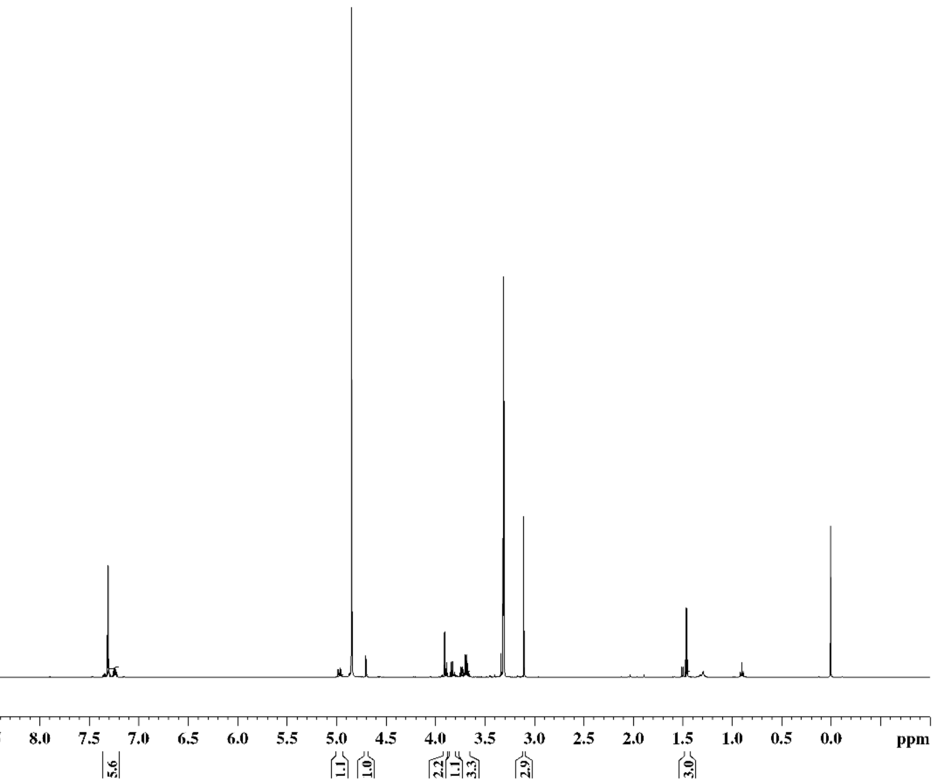

^{13}C NMR (125.8 MHz, MeOD, 25°C):

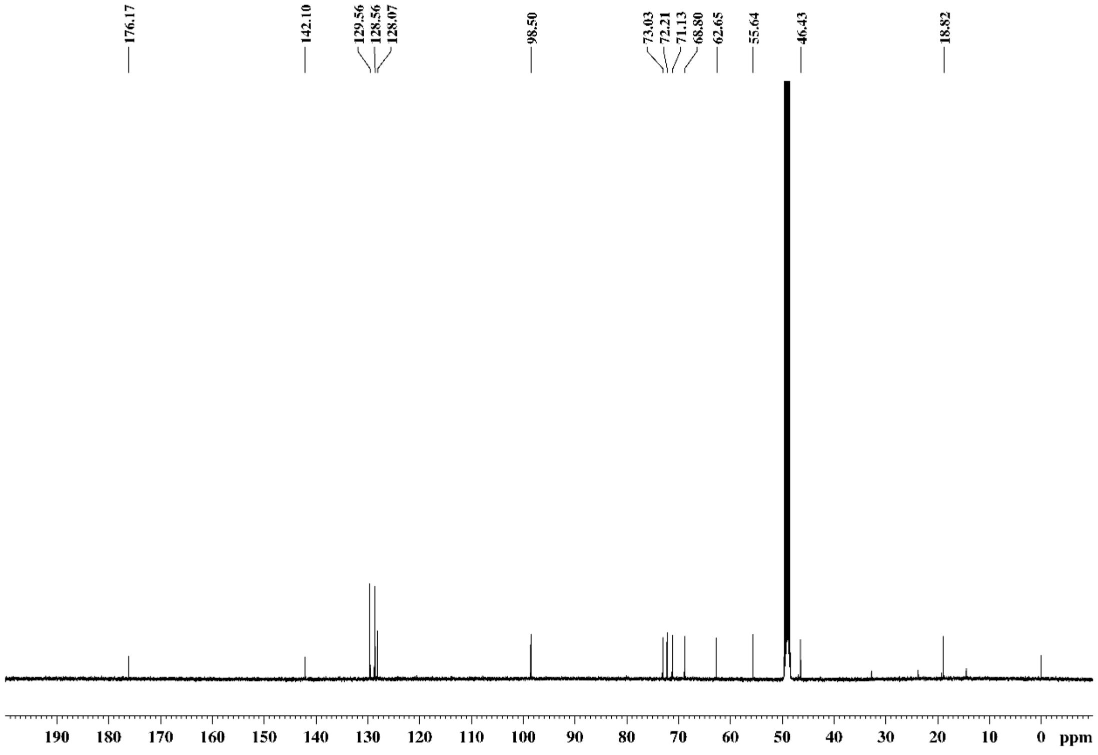

Methyl 4,3-*O*-isopropylidene-6-(2-methoxyisopropyl)-2-*O*-(*S*)-2-phenyl-propanoyl-α-D-galactopyranoside (73):

^{1}H NMR (500.20 MHz, $CDCl_3$, 25°C):

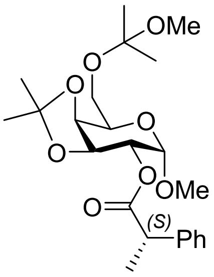

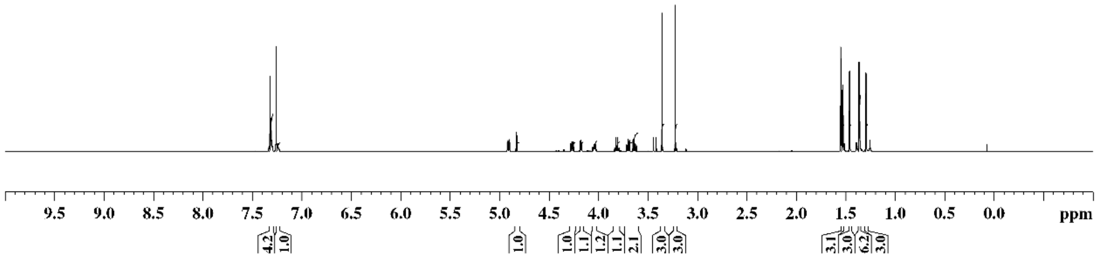

^{13}C NMR (125.8 MHz, $CDCl_3$, 25°C):

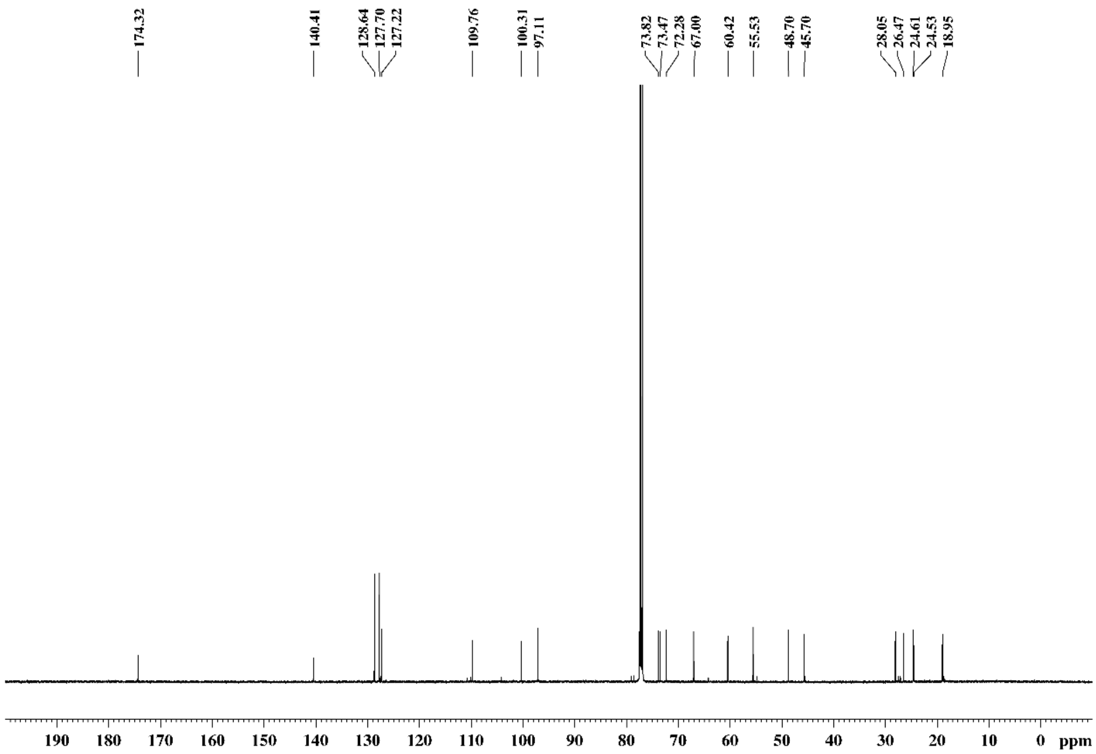

Methyl 2-*O*-(*S*)-2-phenyl-propanoyl-α-D-galactopyranoside (15):

^{1}H NMR (500.20 MHz, MeOD, 25°C):

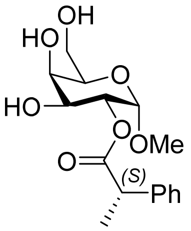

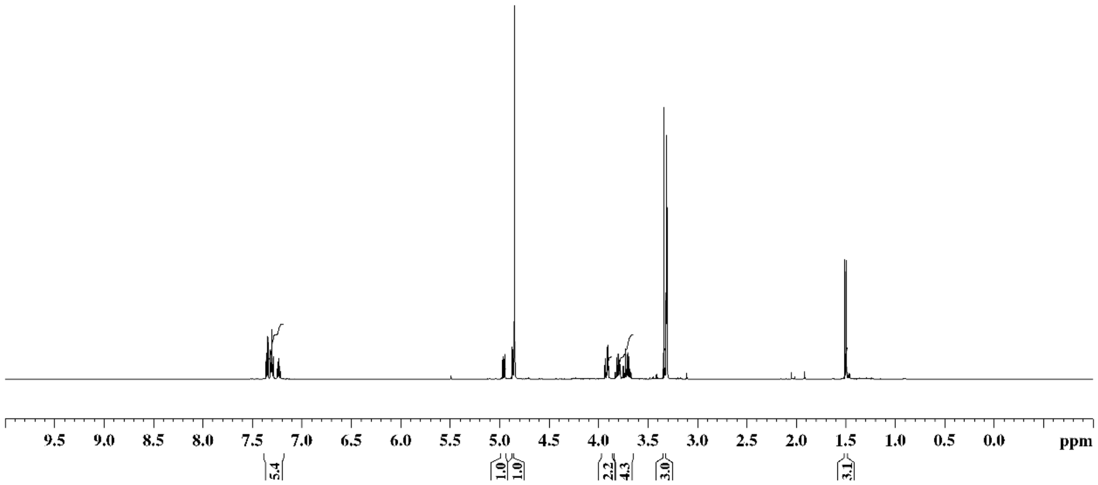

^{13}C NMR (125.8 MHz, MeOD, 25°C):

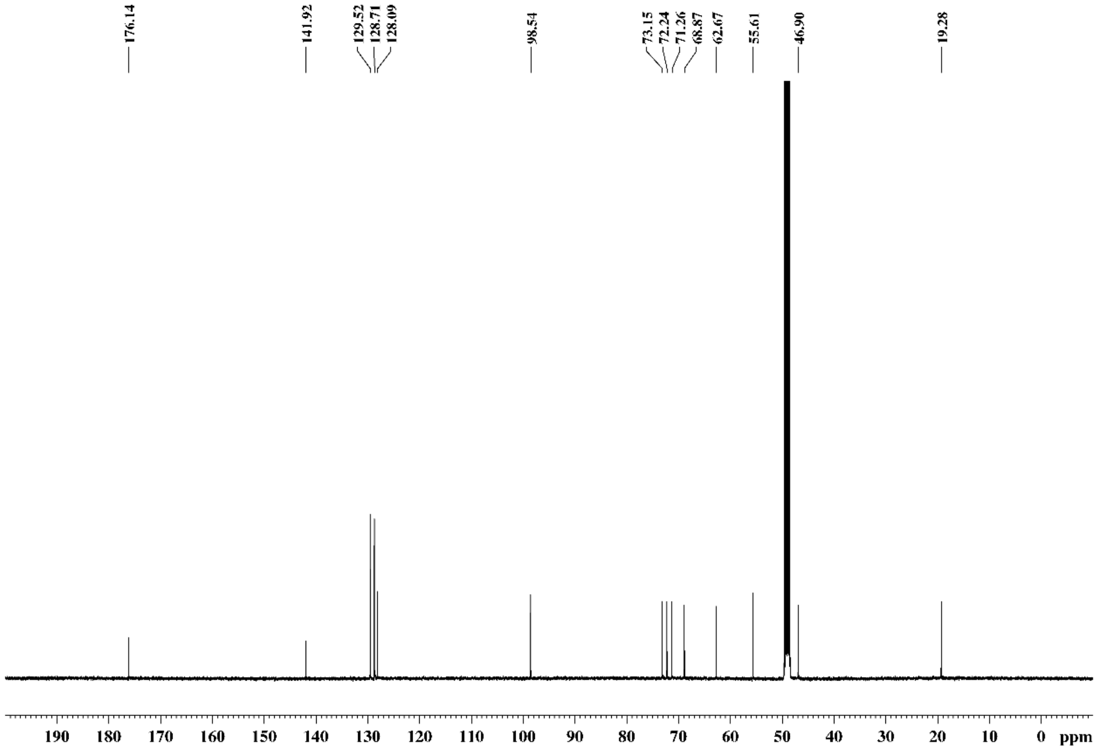

Methyl 2-*O*-acetyl-4,3-*O*-isopropylidene-6-(2-methoxyisopropyl)-β-D-galactopyranoside (75):

^{1}H NMR (500.20 MHz, $CDCl_3$, 25°C):

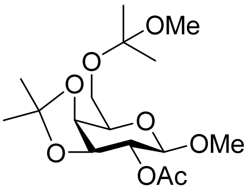

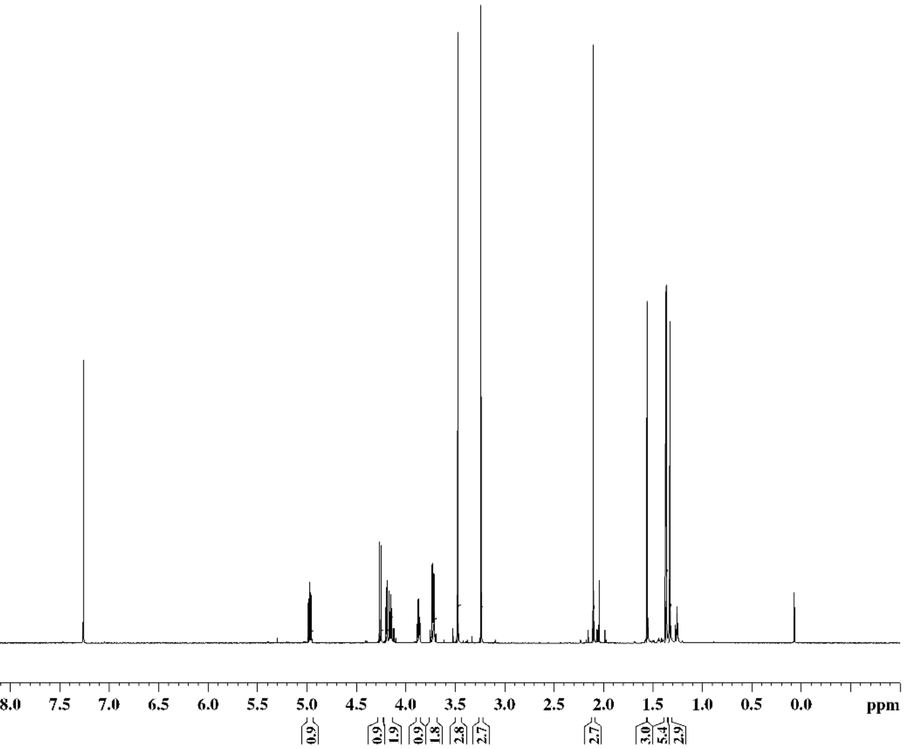

^{13}C NMR (125.8 MHz, $CDCl_3$, 25°C):

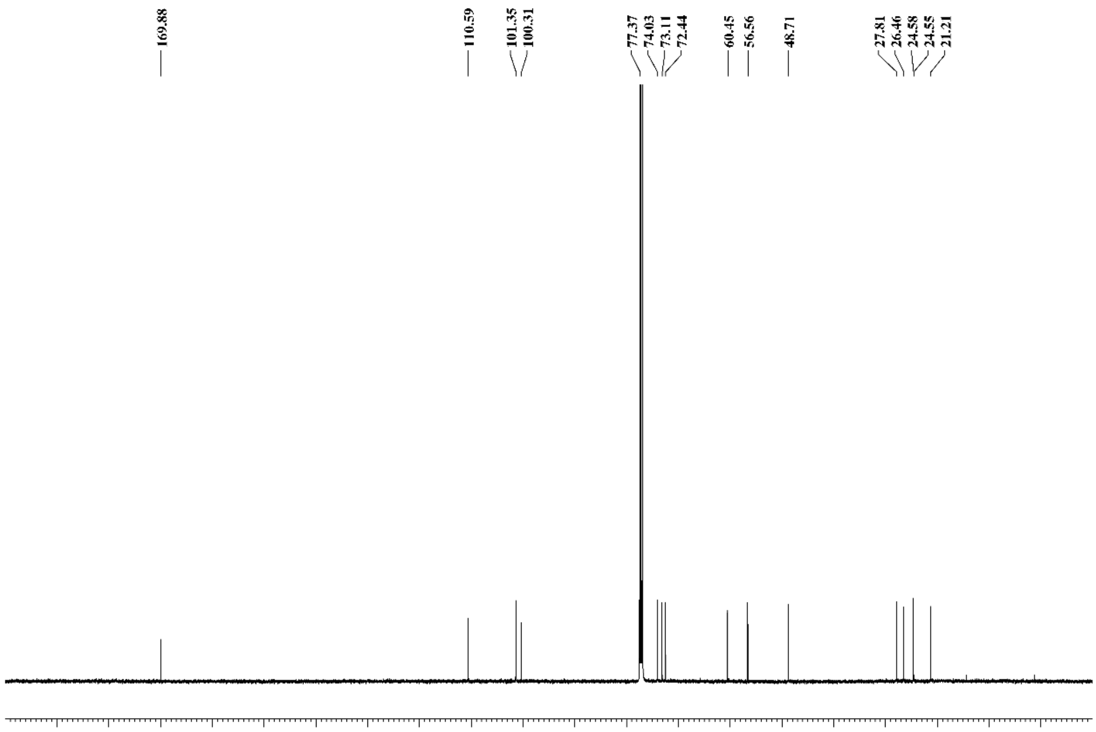

Methyl 2-*O*-acetyl-β-D-galactopyranoside (16):

^{1}H NMR (500.20 MHz, MeOD, 25°C):

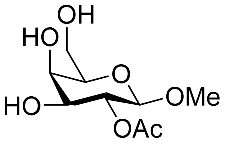

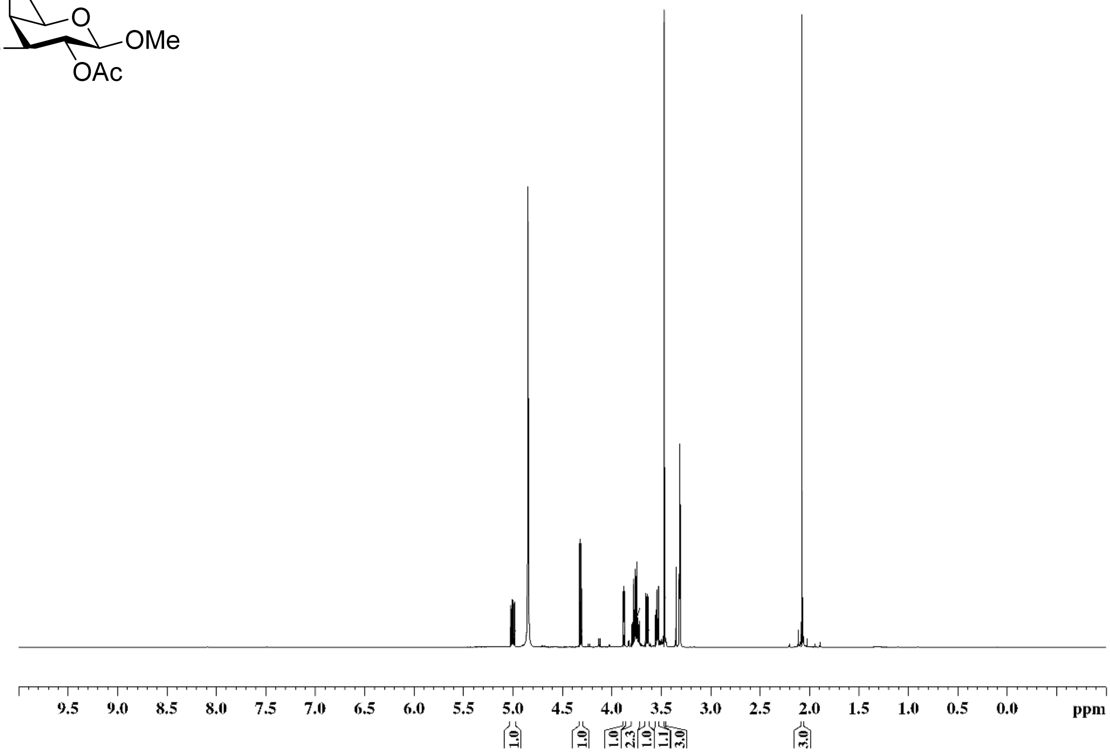

^{13}C NMR (125.8 MHz, MeOD, 25°C):

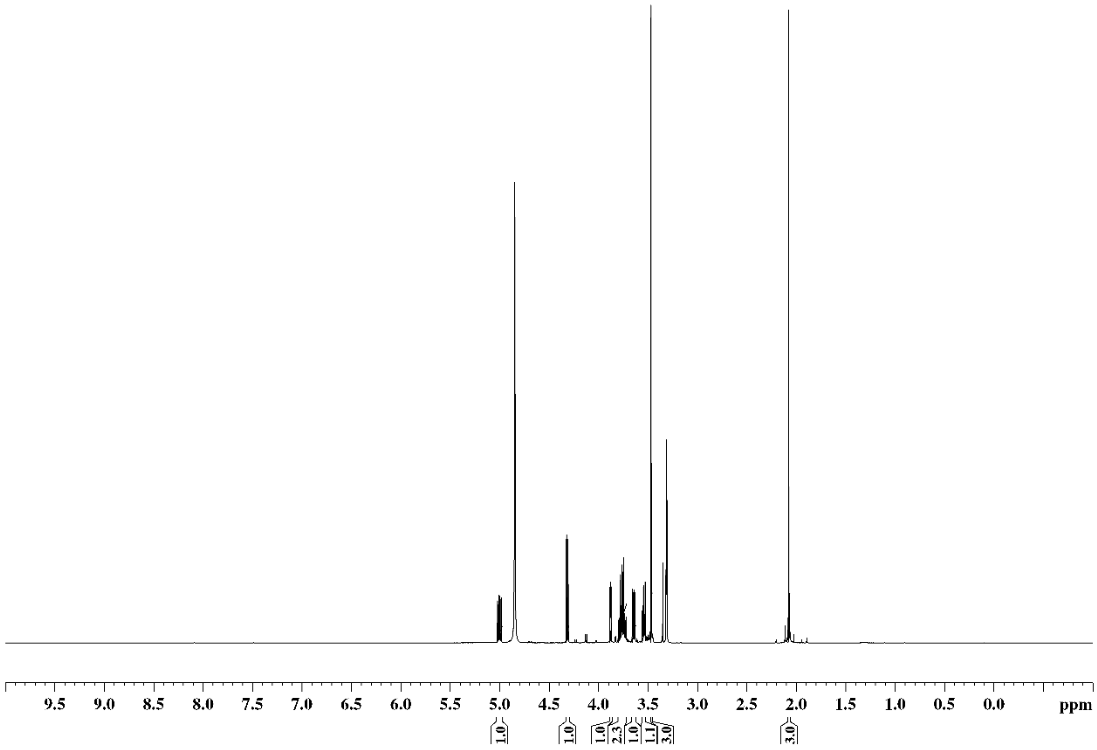

Methyl 2-*O*-benzoyl-4,3-*O*-isopropylidene-6-(2-methoxyisopropyl)-β-D-galactopyranoside (76):

^{1}H NMR (500.20 MHz, $CDCl_3$, 25°C):

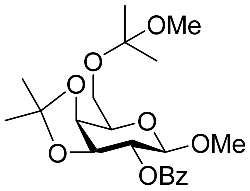

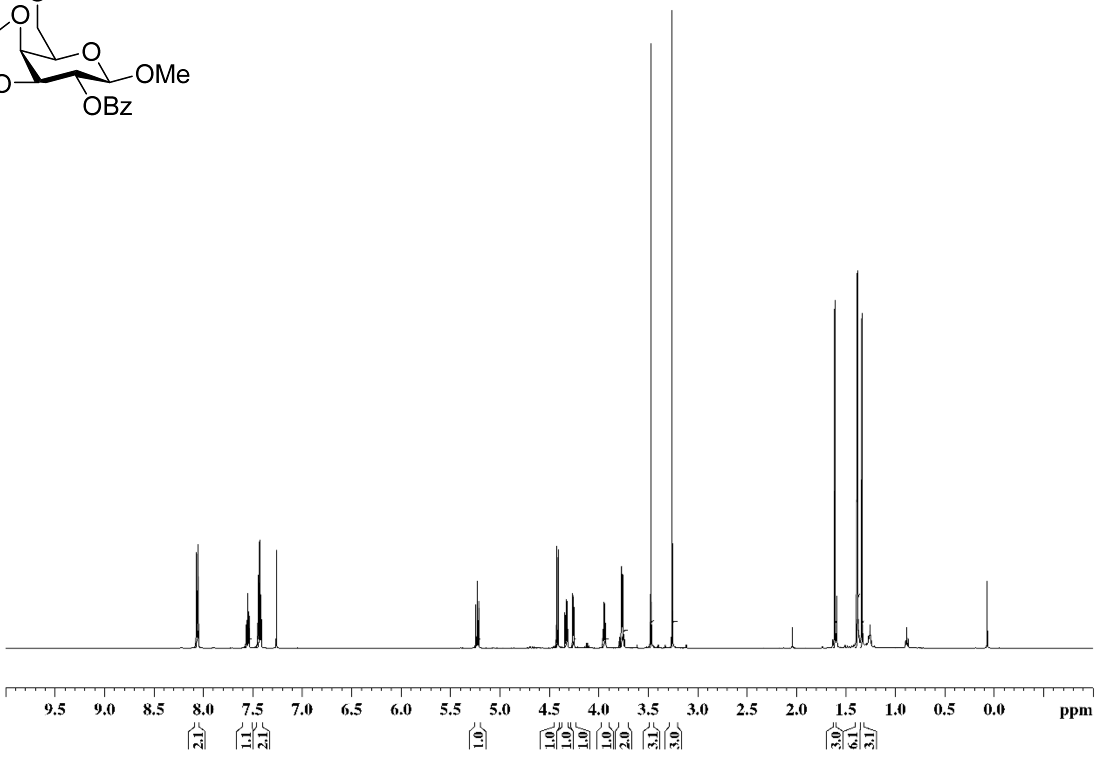

^{13}C NMR (125.8 MHz, $CDCl_3$, 25°C):

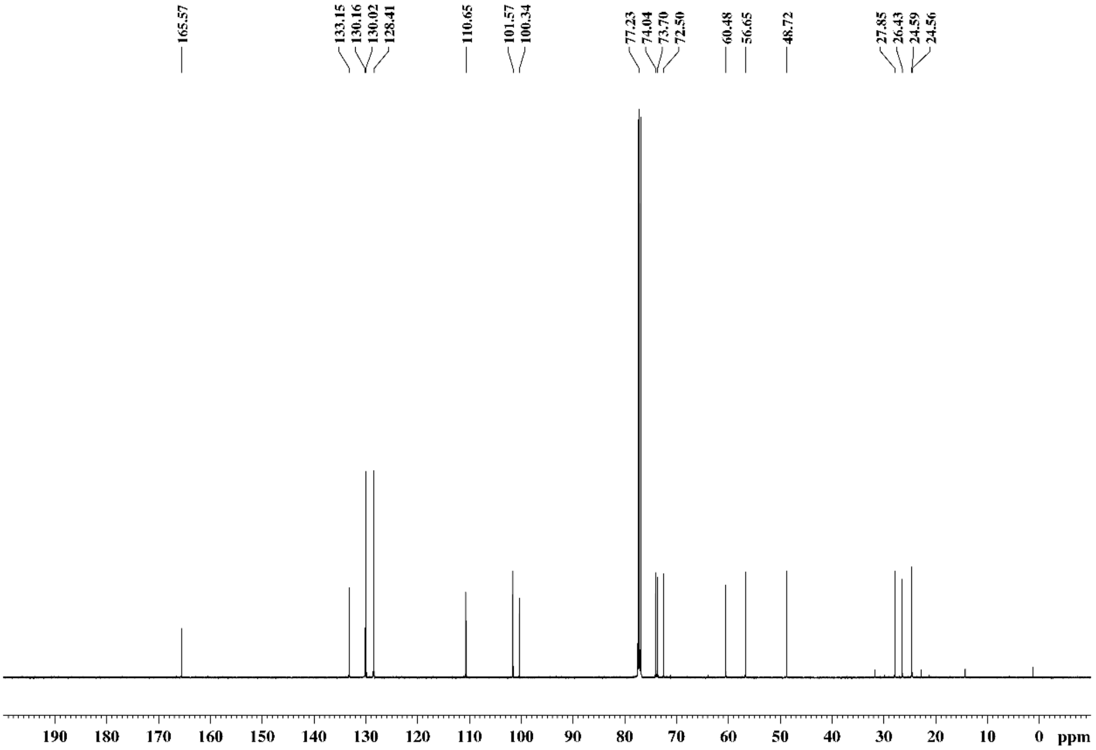

Methyl 2-*O*-benzoyl-β-D-galactopyranoside (17):

^{1}H NMR (500.20 MHz, MeOD, 25°C):

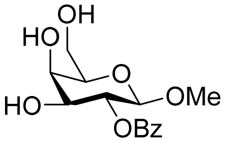

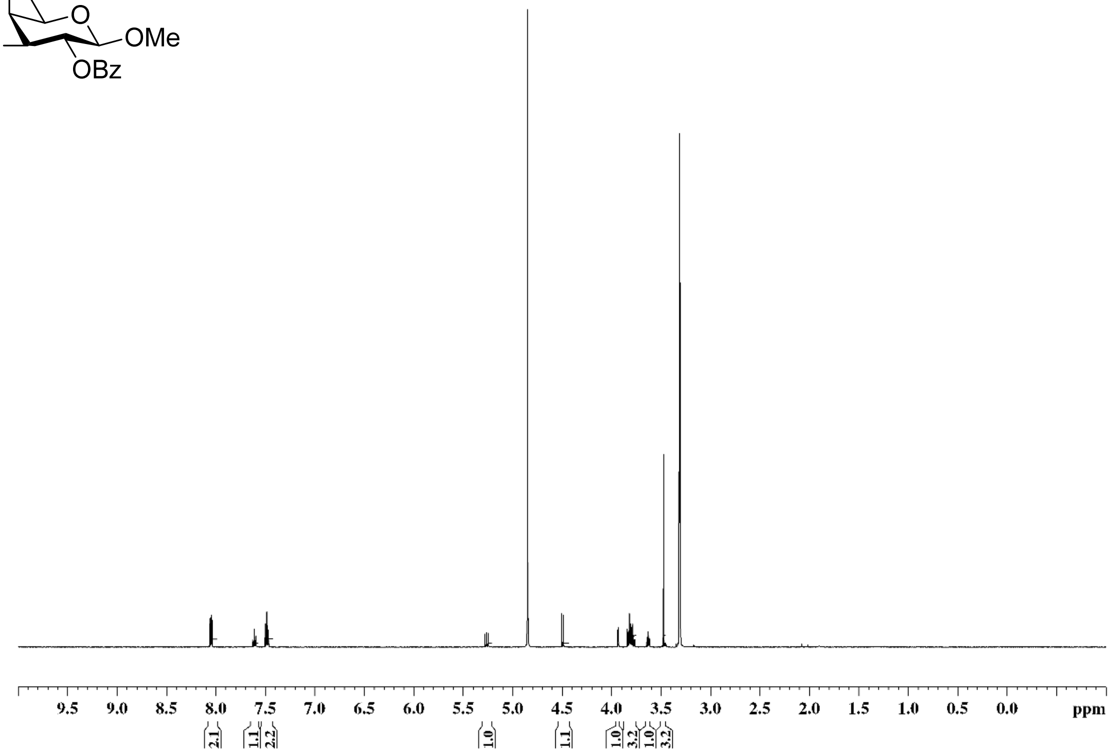

^{13}C NMR (125.8 MHz, MeOD, 25°C):

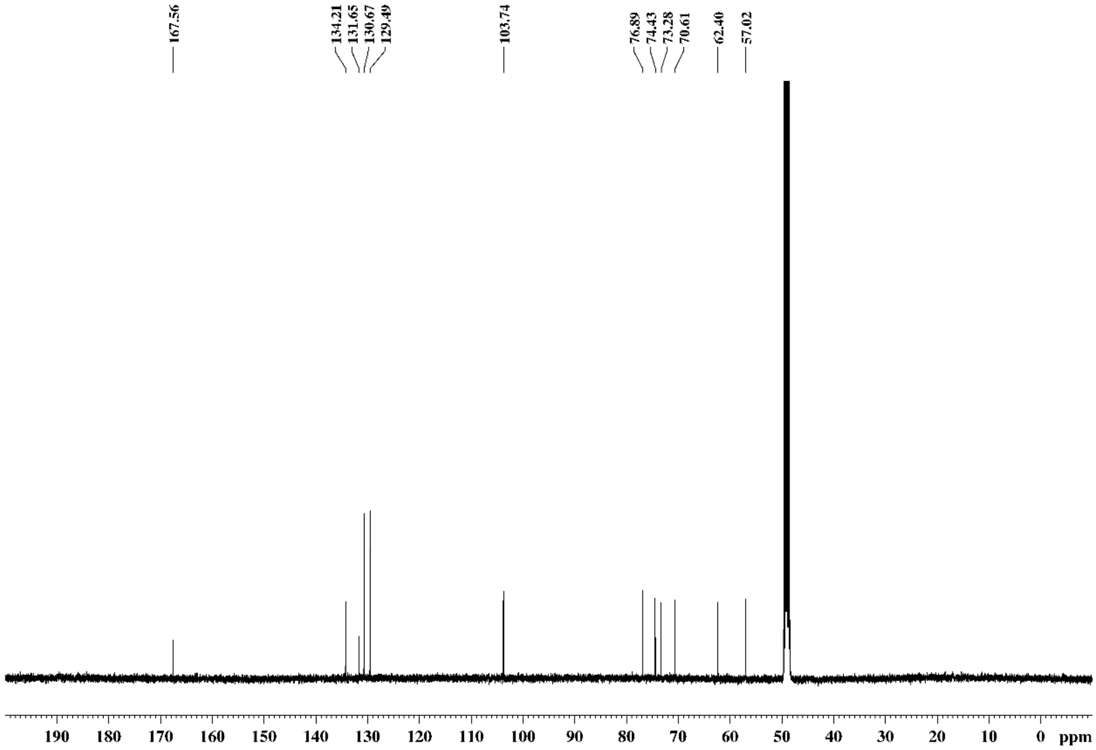

Methyl 4,3-*O*-isopropylidene-6-(2-methoxyisopropyl)-2-*O*-pivaloyl-β-D-galactopyranoside (77):

^{1}H NMR (500.20 MHz, $CDCl_3$, 25°C):

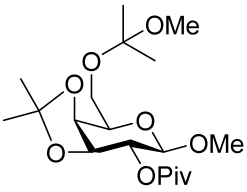

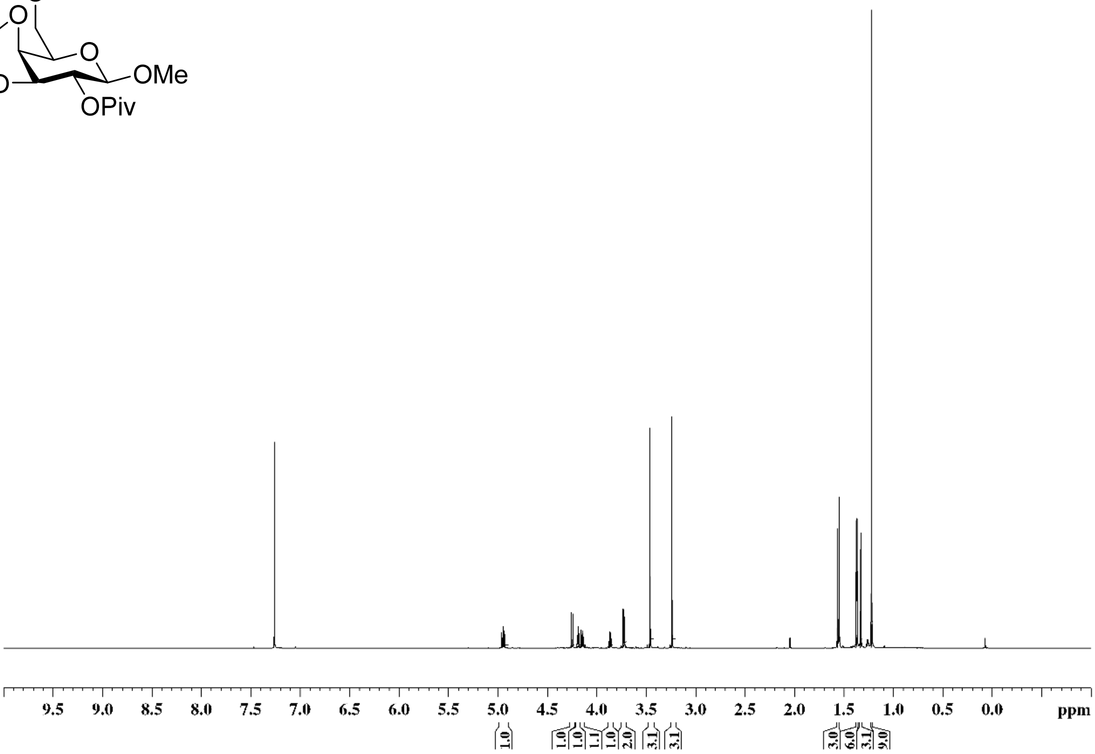

^{13}C NMR (125.8 MHz, $CDCl_3$, 25°C):

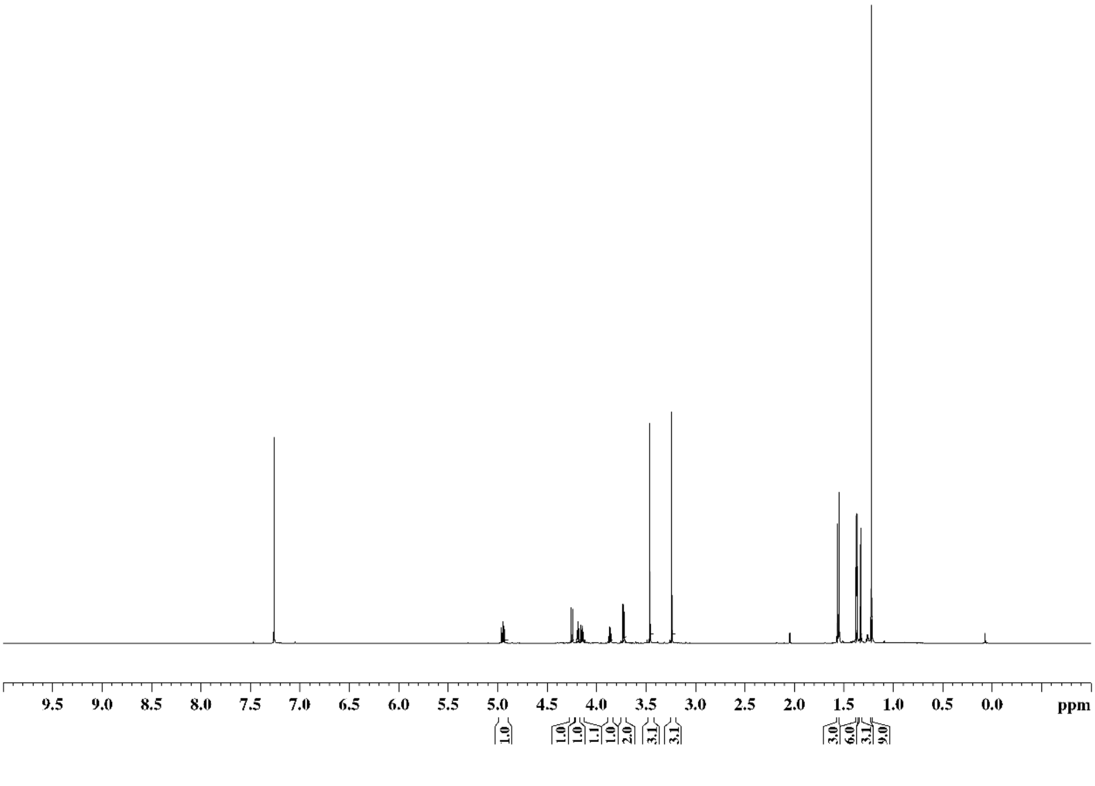

Methyl 2-*O*-pivaloyl-β-D-galactopyranoside (18):

^{1}H NMR (500.20 MHz, MeOD, 25°C):

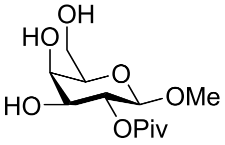

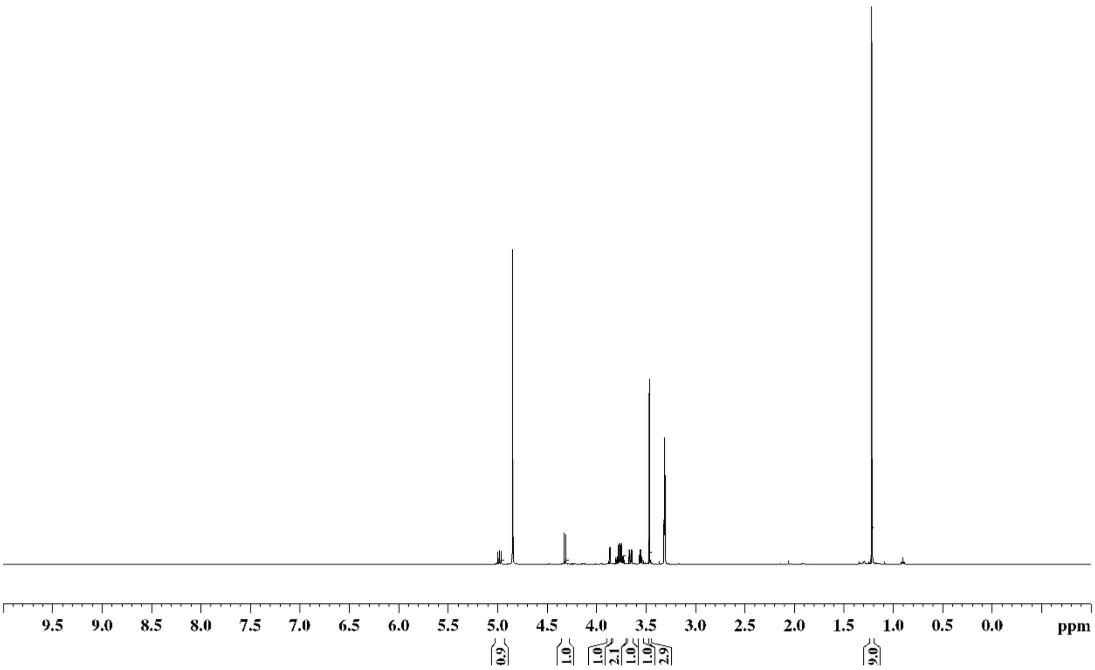

^{13}C NMR (125.8 MHz, MeOD, 25°C):

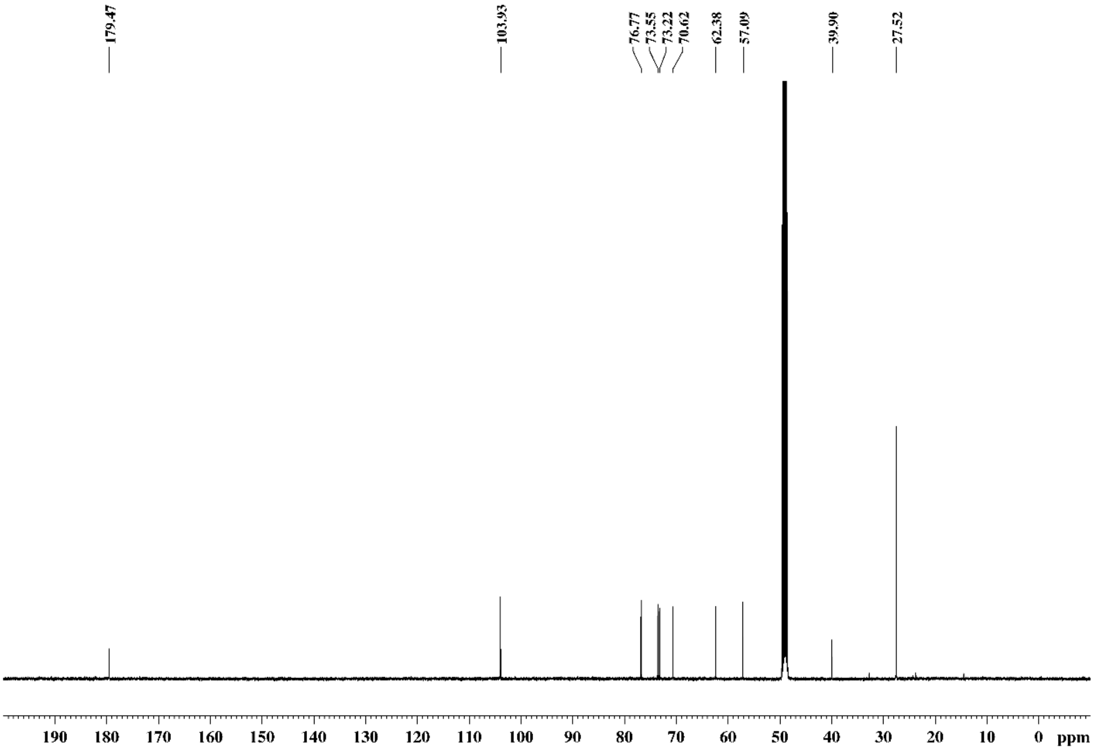

Methyl 4,3-*O*-isopropylidene-6-(2-methoxyisopropyl)-2-*O*-(*R*)-2-phenyl-propanoyl-β-D-galactopyranoside (78):

^{1}H NMR (500.20 MHz, $CDCl_3$, 25°C):

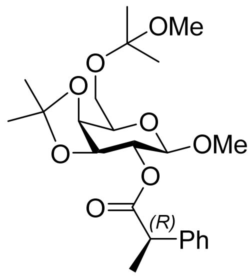

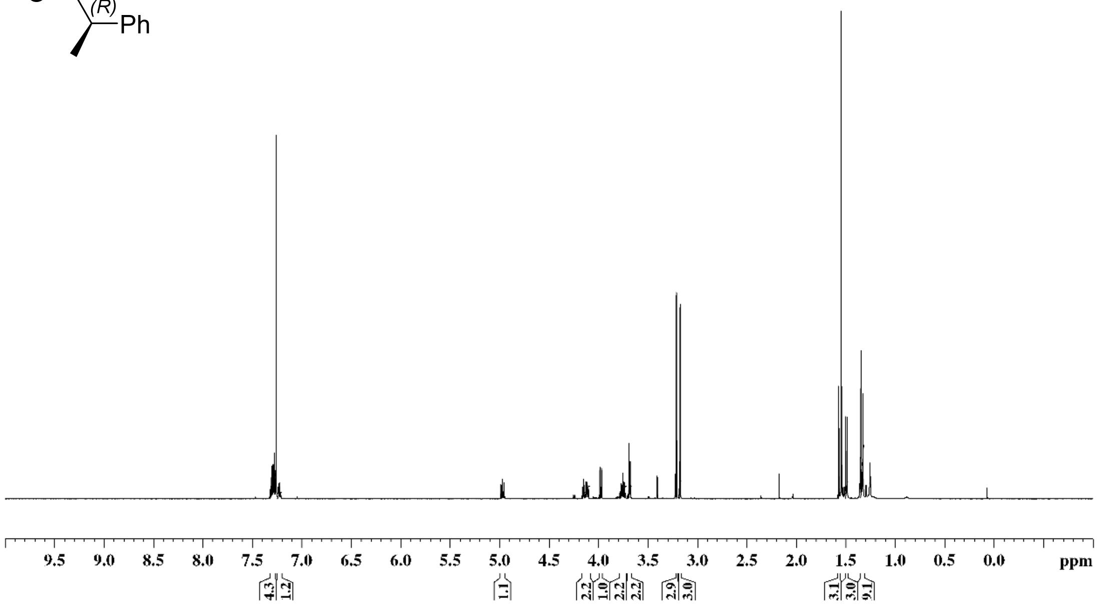

^{13}C NMR (125.8 MHz, $CDCl_3$, 25°C):

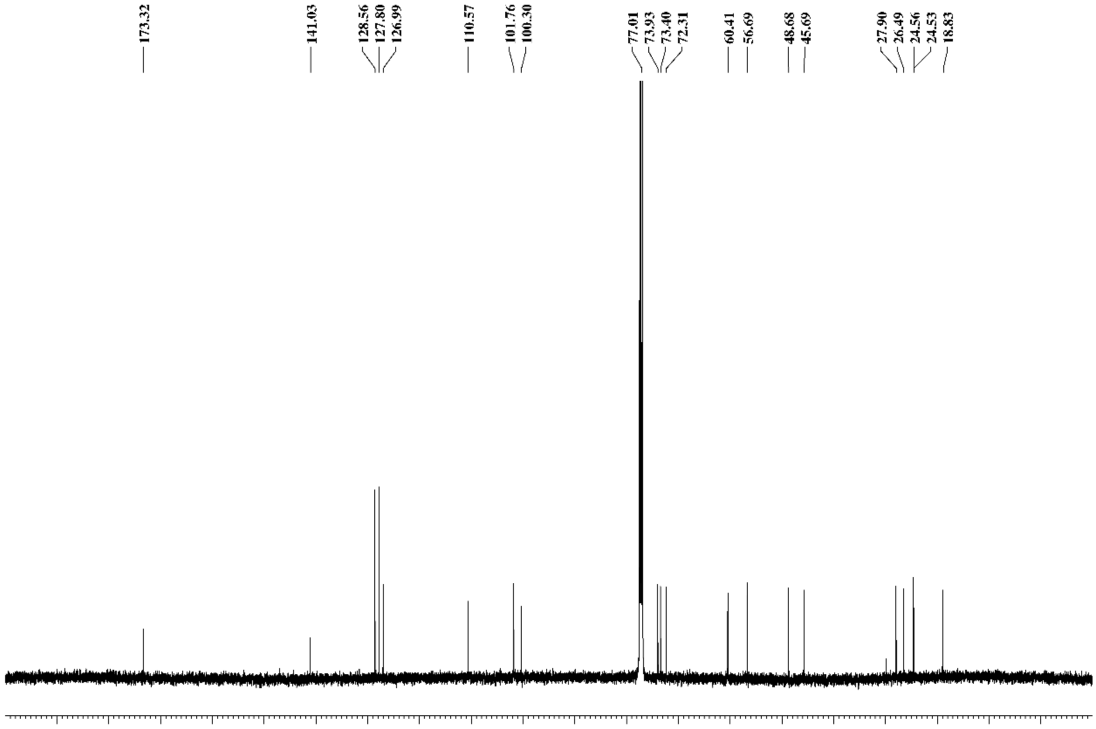

Methyl 2-*O*-(*R*)-2-phenyl-propanoyl-β-D-galactopyranoside (19):

^{1}H NMR (500.20 MHz, MeOD, 25°C):

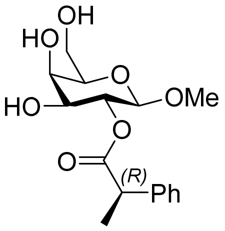

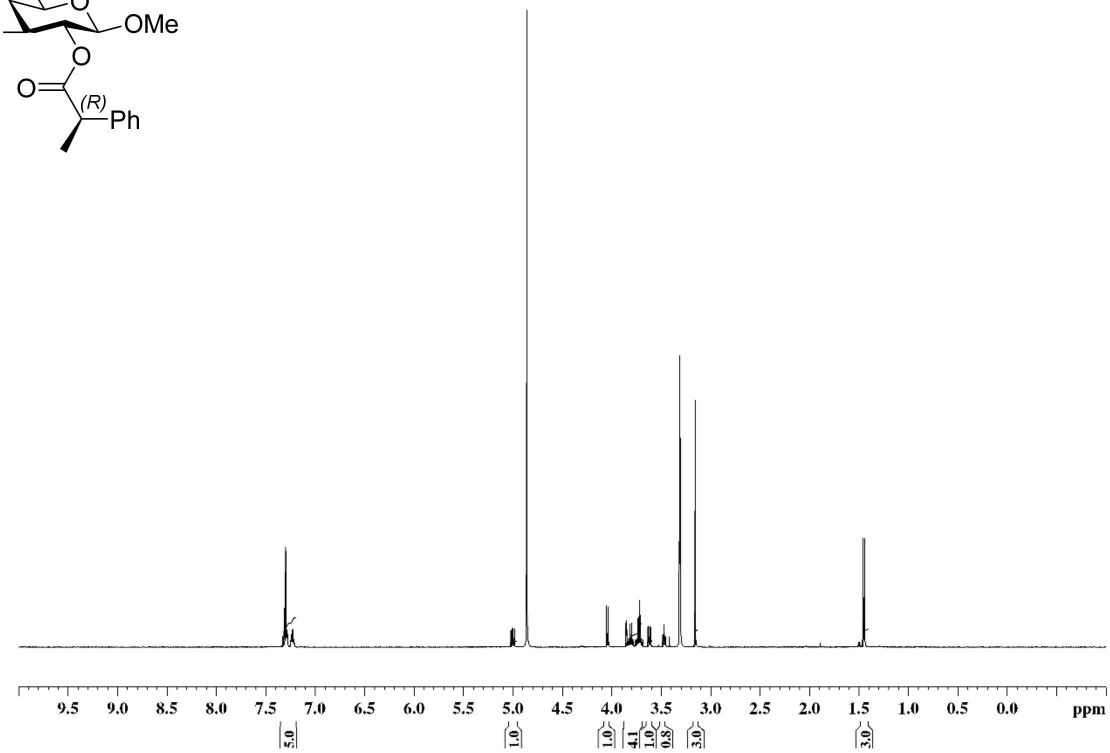

^{13}C NMR (125.8 MHz, MeOD, 25°C):

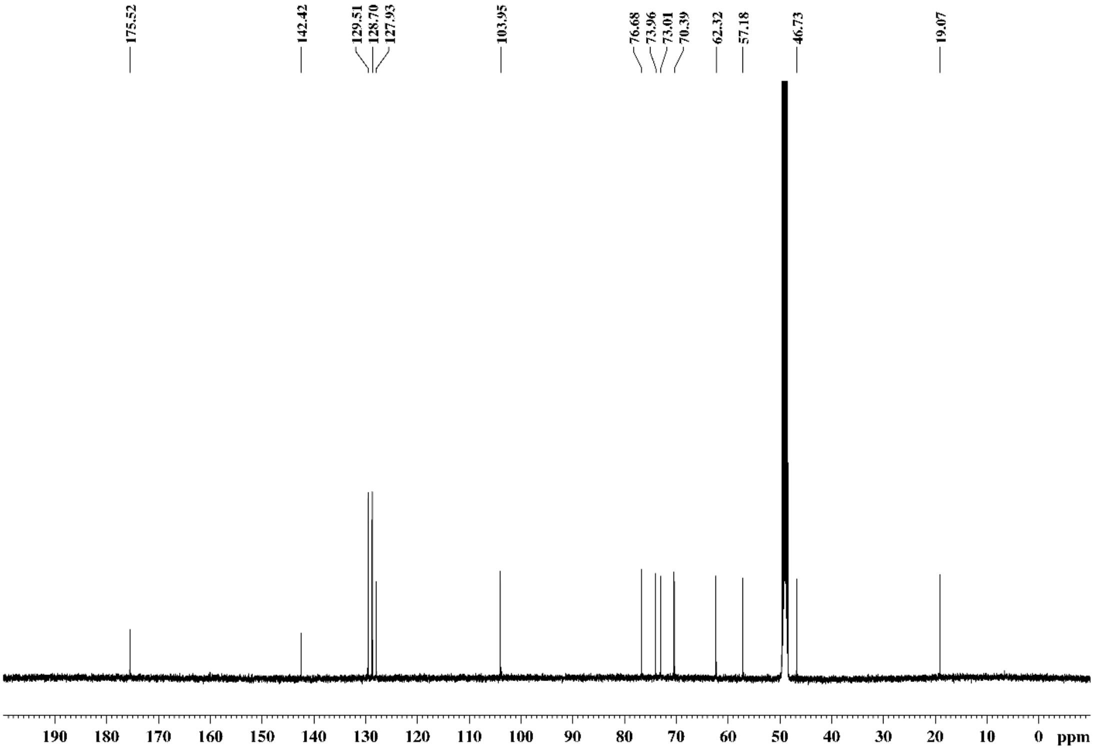

Methyl 4,3-*O*-isopropylidene-6-(2-methoxyisopropyl)-2-*O*-(*S*)-2-phenyl-propanoyl-β-D-galactopyranoside (79):

^{1}H NMR (500.20 MHz, CDCl$_3$, 25°C):

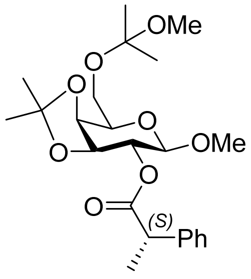

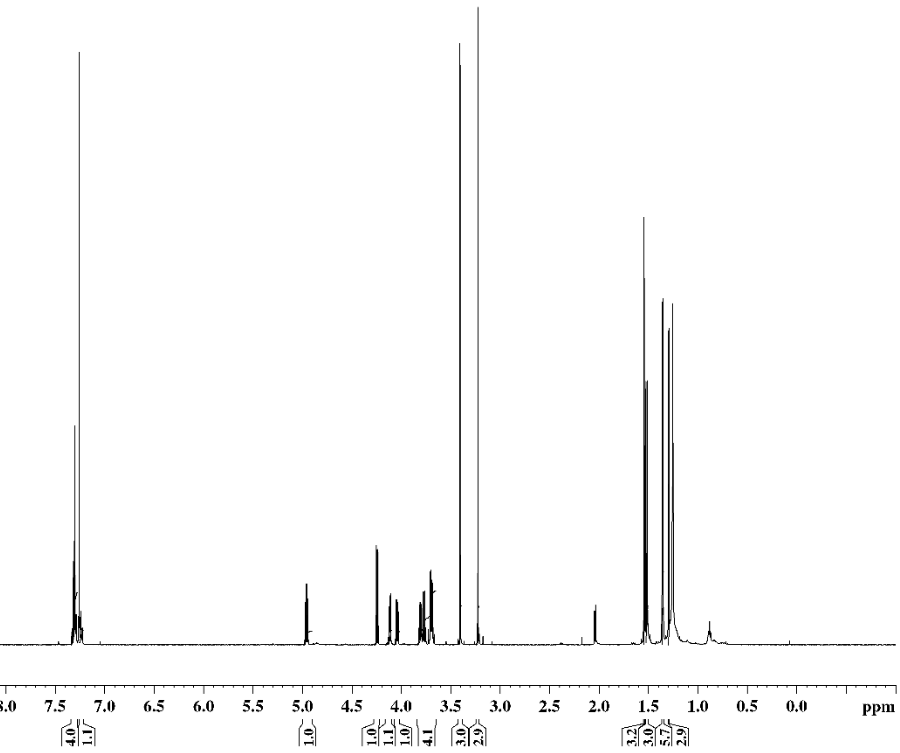

^{13}C NMR (125.8 MHz, CDCl$_3$, 25°C):

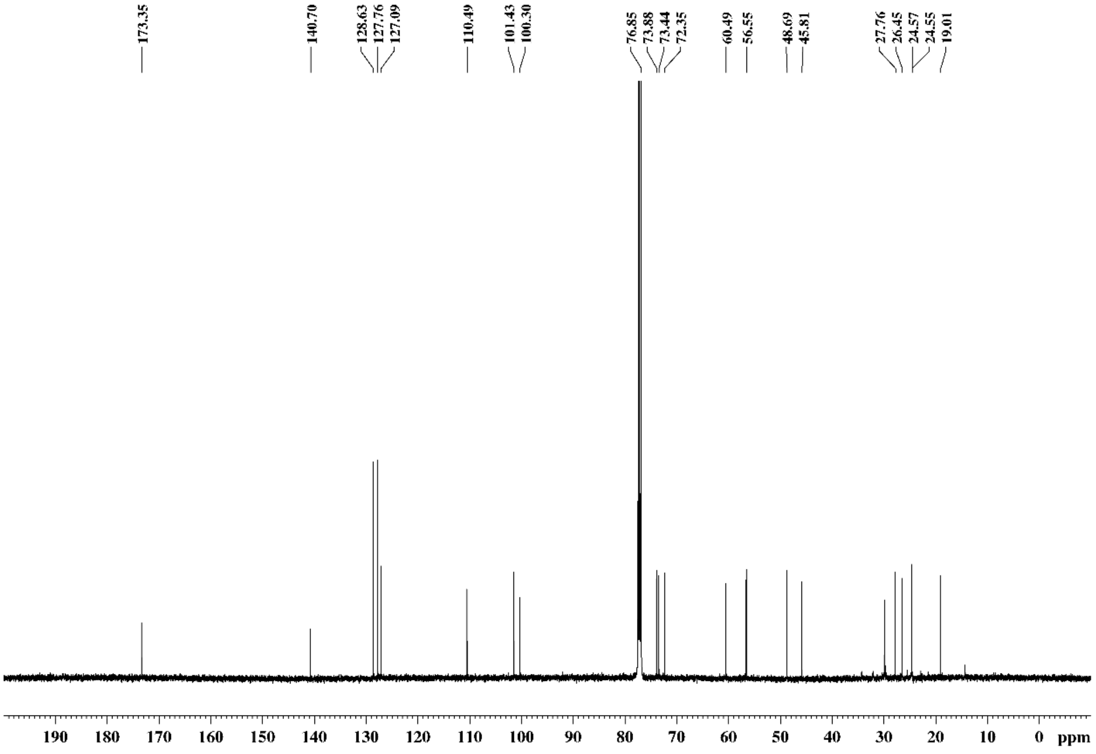

Methyl 2-*O*-(*R*)-2-phenyl-propanoyl-β-D-galactopyranoside (20):

^{1}H NMR (500.20 MHz, MeOD, 25°C):

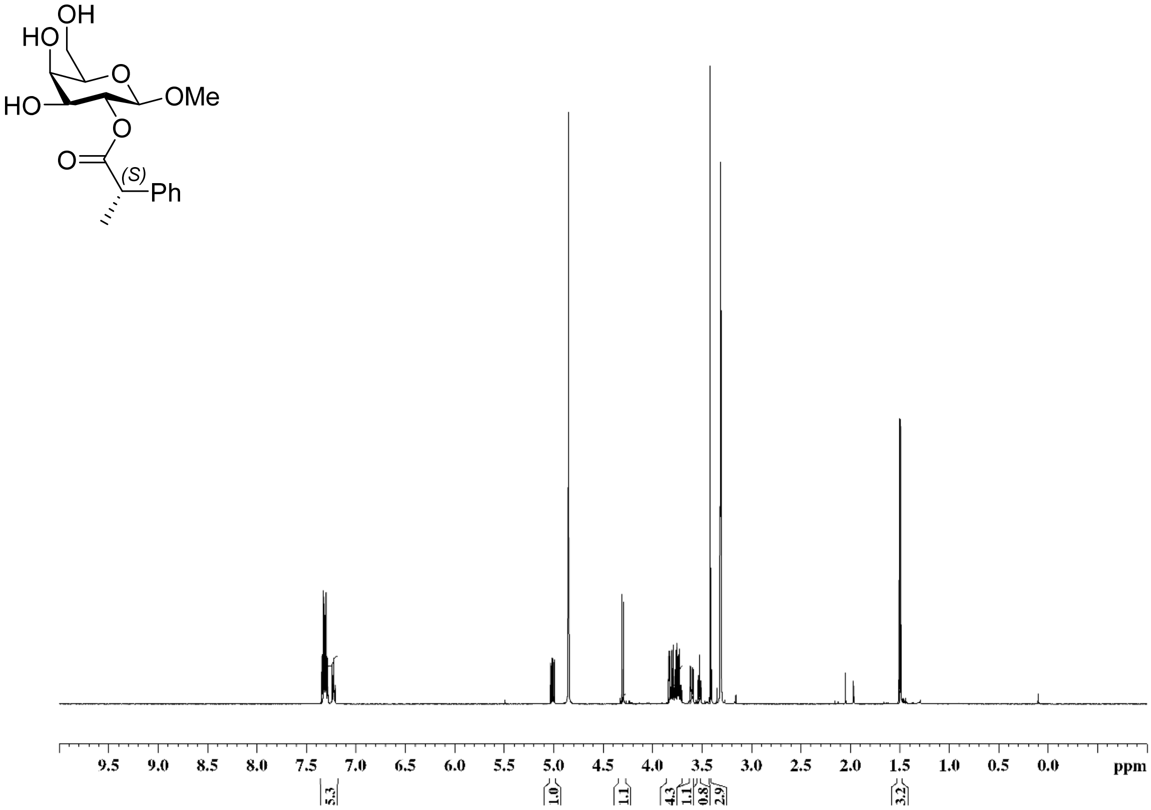

^{13}C NMR (125.8 MHz, MeOD, 25°C):

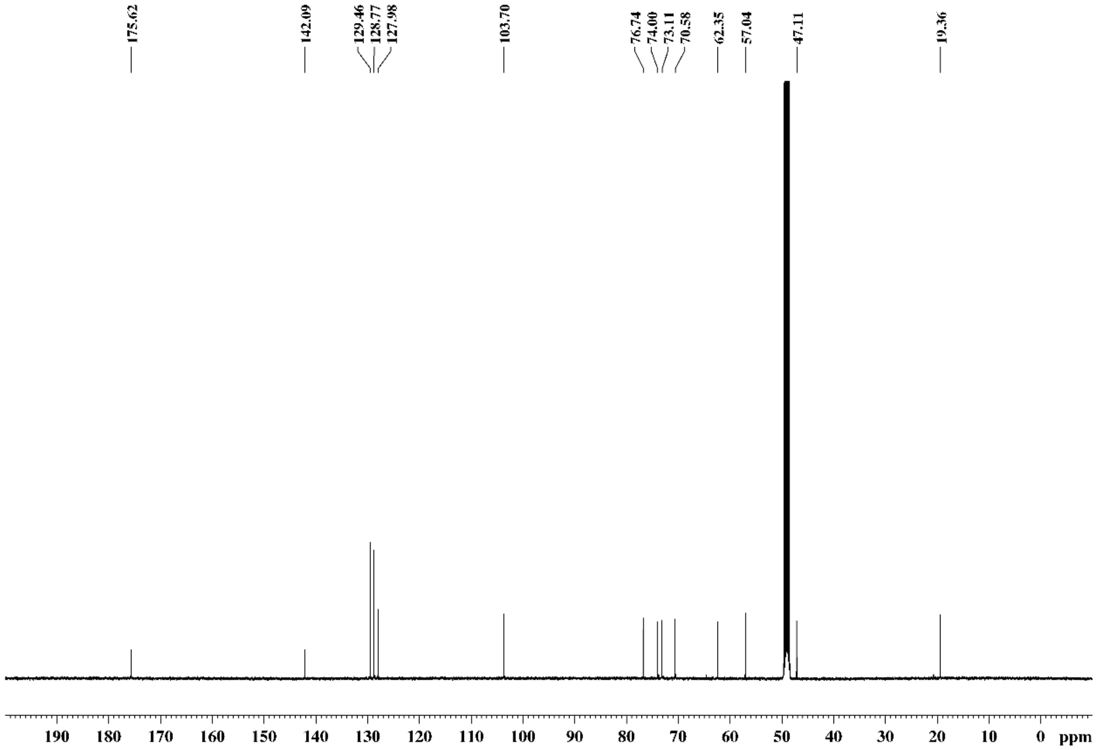

Methyl 3-*O*-benzyl-2-*O*-benzoyl-4,6-*O*-benzylidene-α-D-mannopyranoside (82):

^{1}H NMR (500.20 MHz, $CDCl_3$, 25°C):

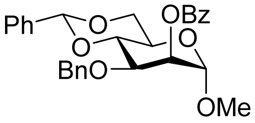

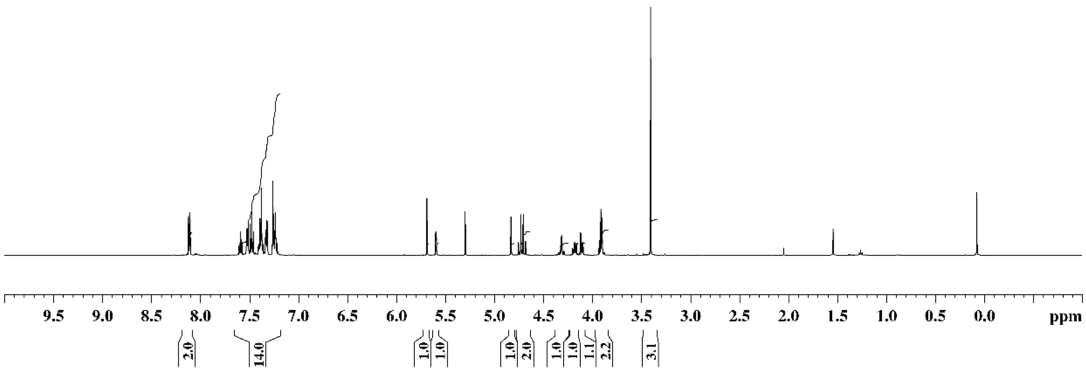

^{13}C NMR (125.8 MHz, $CDCl_3$, 25°C):

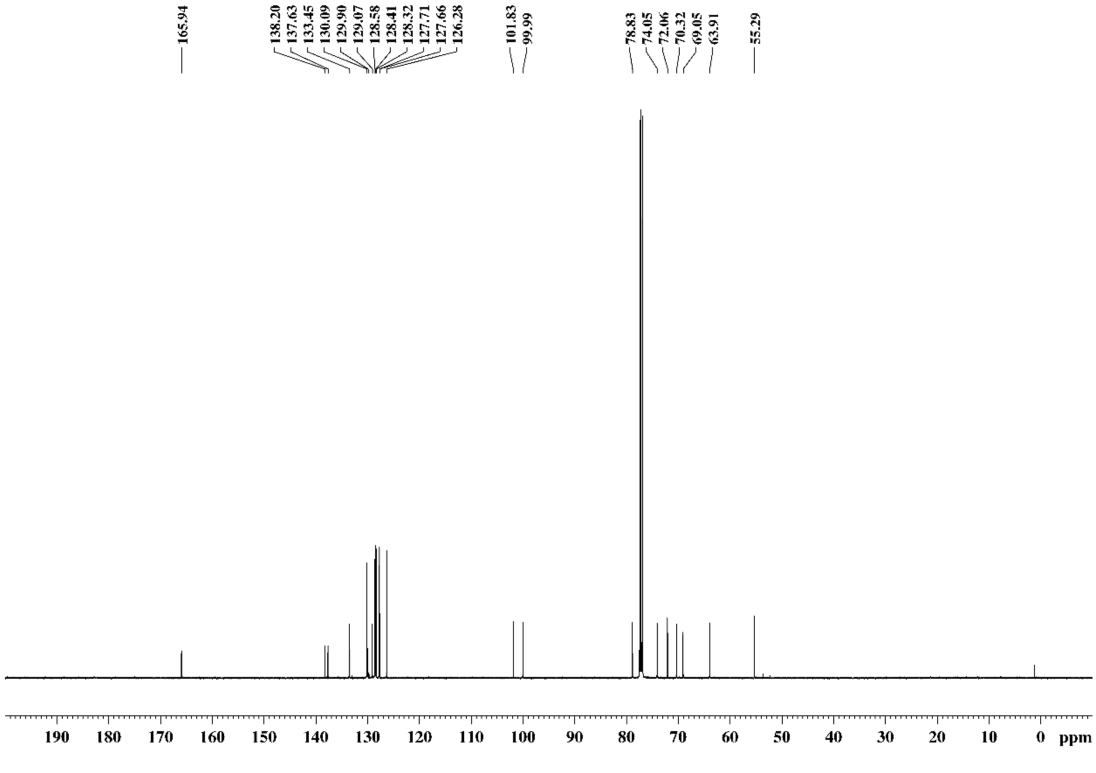

Methyl 2-*O*-benzoyl-α-D-mannopyranoside (22):

^{1}H NMR (500.20 MHz, MeOD, 25°C):

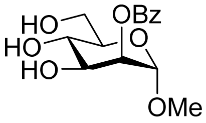

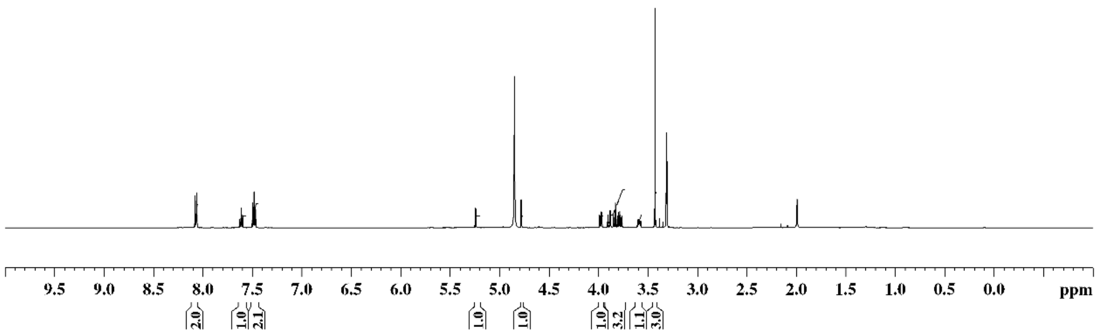

^{13}C NMR (125.8 MHz, MeOD, 25°C):

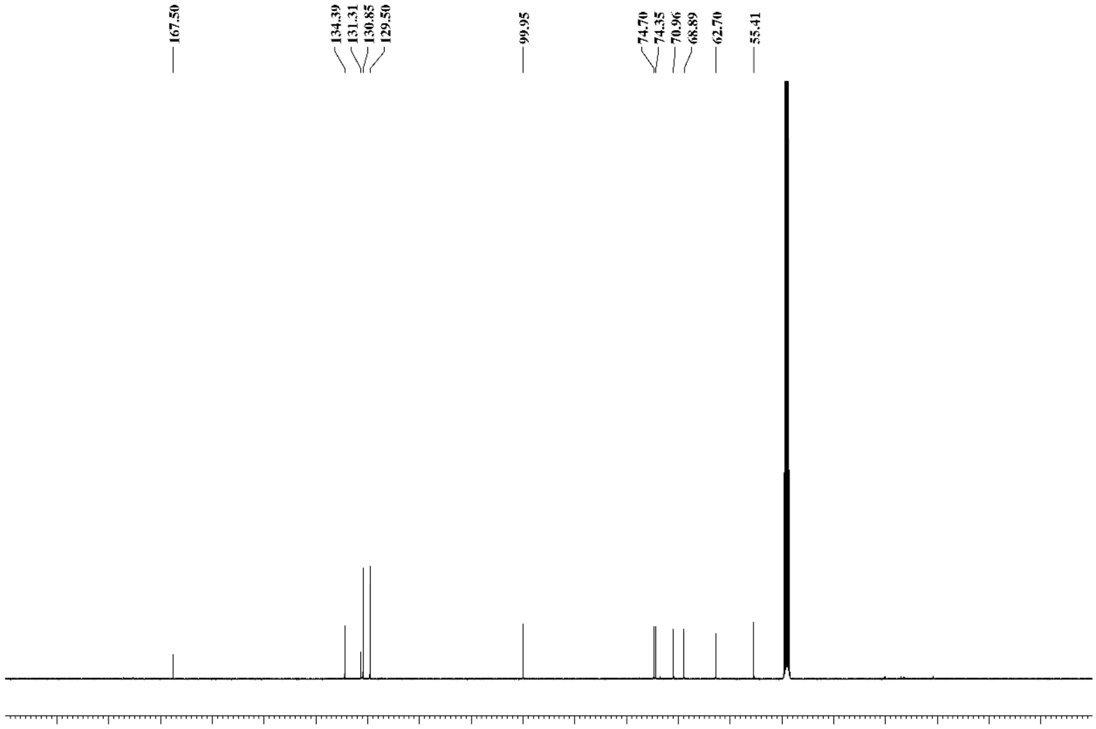

Methyl 3-*O*-benzyl-4,6-*O*-benzylidene-2-*O*-pivaloyl-α-D-mannopyranoside (83):

^{1}H NMR (500.20 MHz, CDCl$_3$, 25°C):

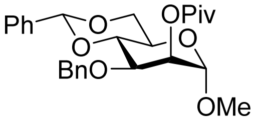

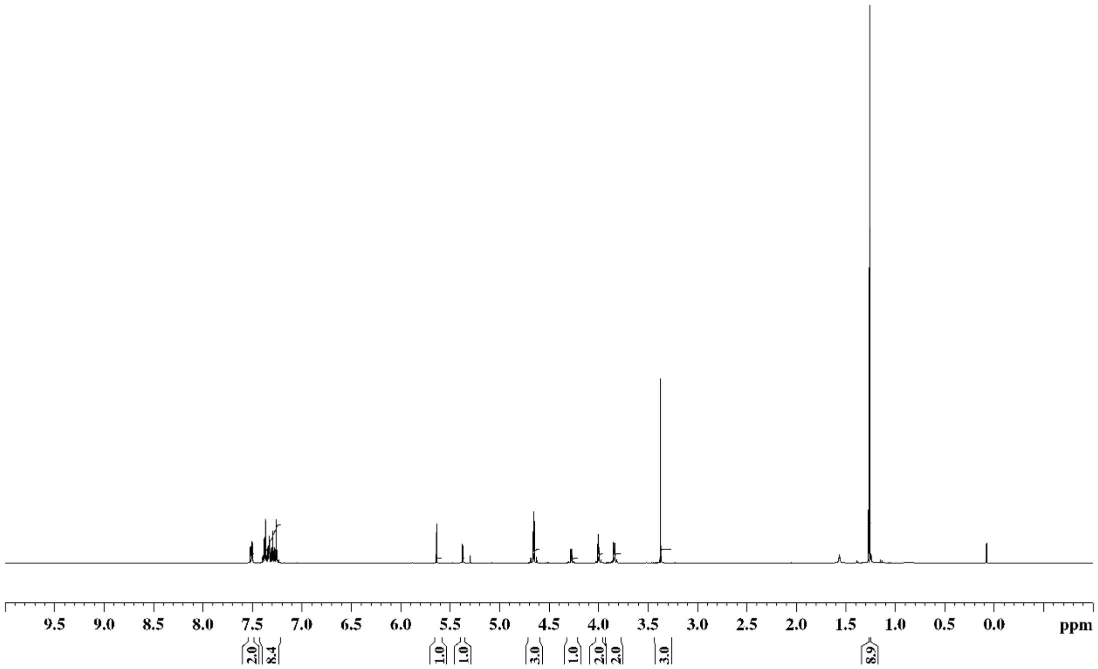

^{13}C NMR (125.8 MHz, CDCl$_3$, 25°C):

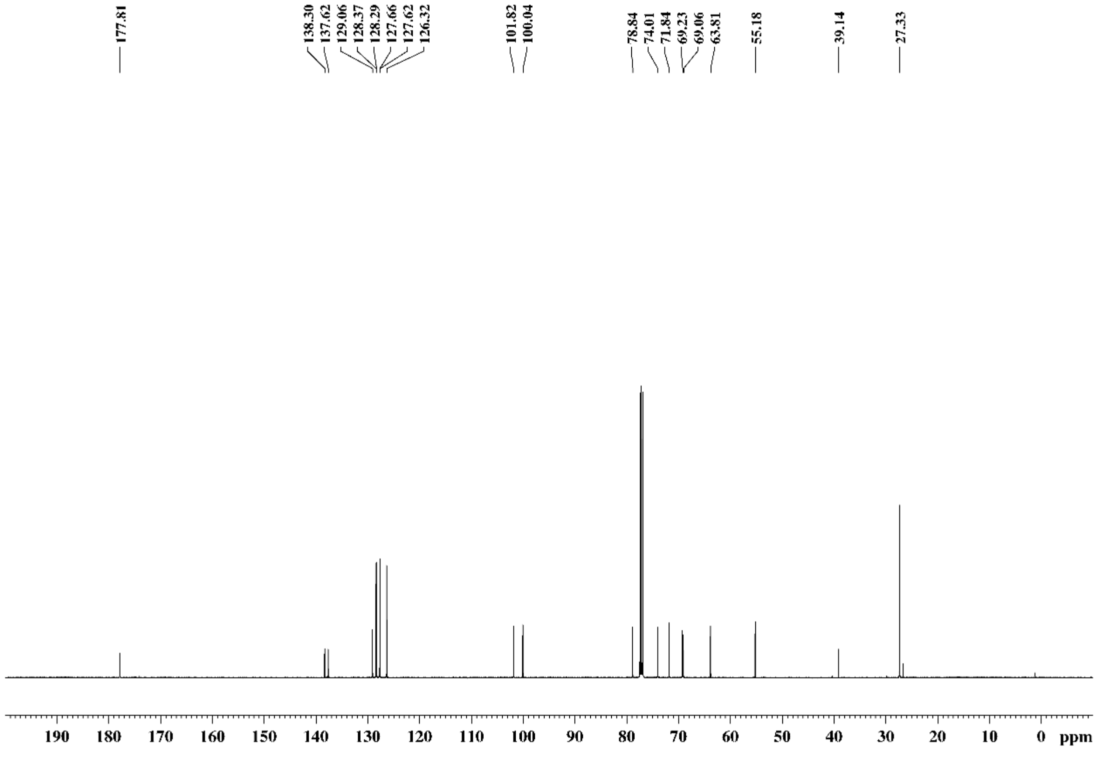

Methyl 2-*O*-pivaloyl-α-D-mannopyranoside (23):

^{1}H NMR (500.20 MHz, MeOD, 25°C):

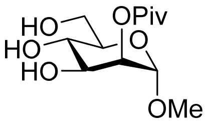

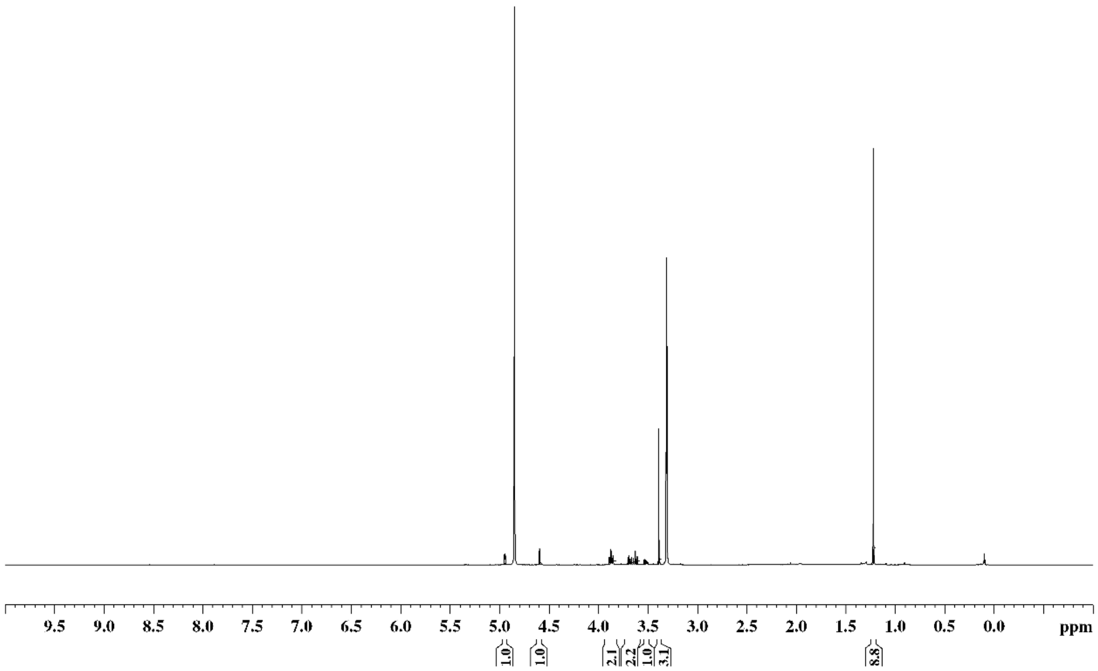

^{13}C NMR (125.8 MHz, MeOD, 25°C):

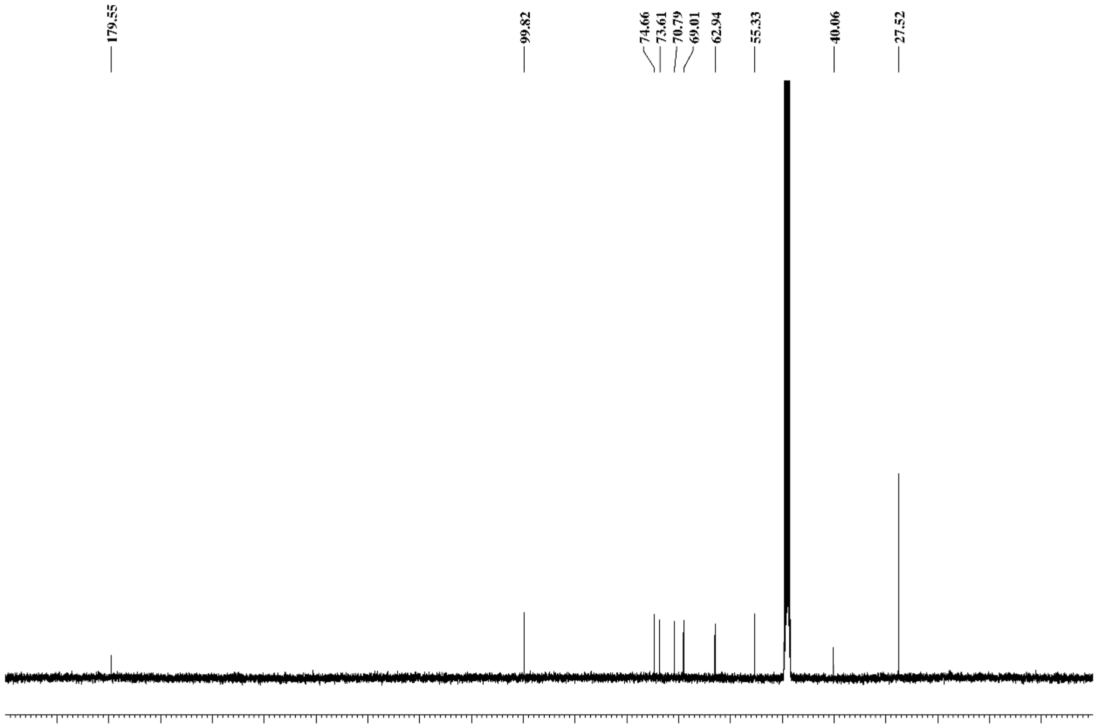

Methyl 3-*O*-benzyl-4,6-*O*-benzylidene-2-*O*-(*R*)-2-phenyl-propanoyl-α-D-mannopyranoside (84):

^{1}H NMR (500.20 MHz, $CDCl_3$, 25°C):

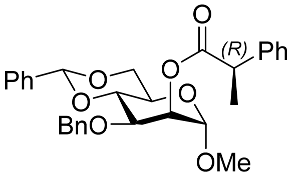

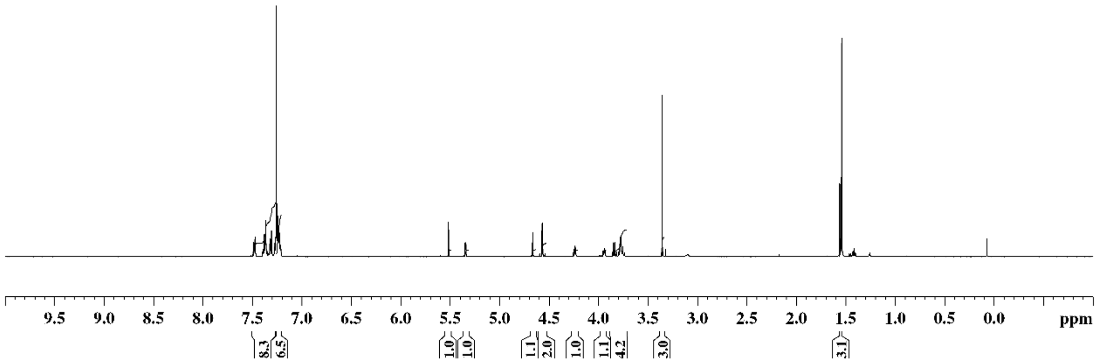

^{13}C NMR (125.8 MHz, $CDCl_3$, 25°C):

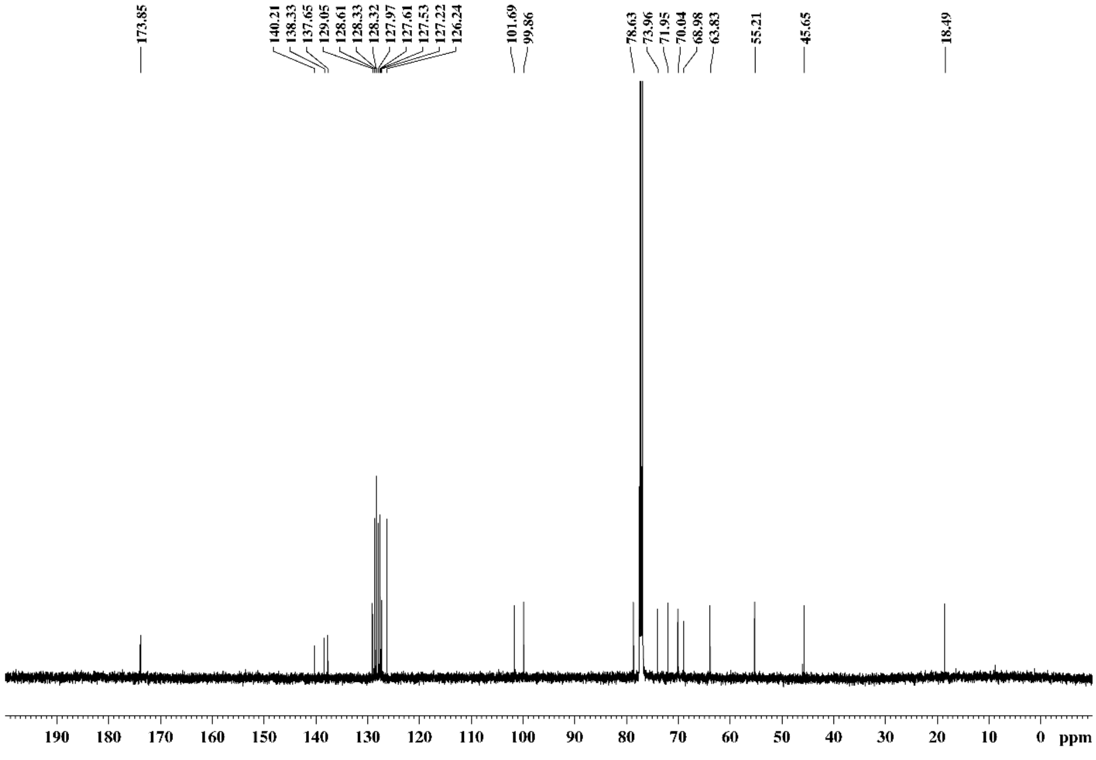

Methyl 2-*O*-(*R*)-2-phenyl-propanoyl-α-D-mannopyranoside (24):

^{1}H NMR (500.20 MHz, MeOD, 25°C):

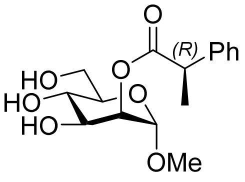

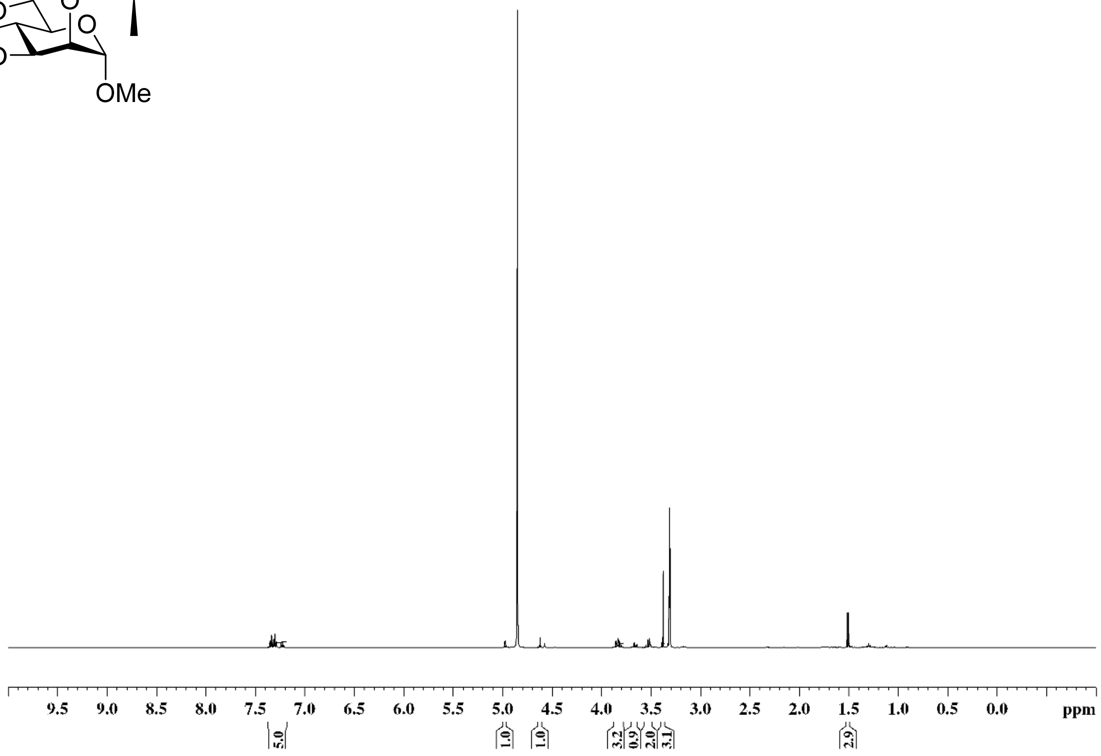

^{13}C NMR (125.8 MHz, MeOD, 25°C):

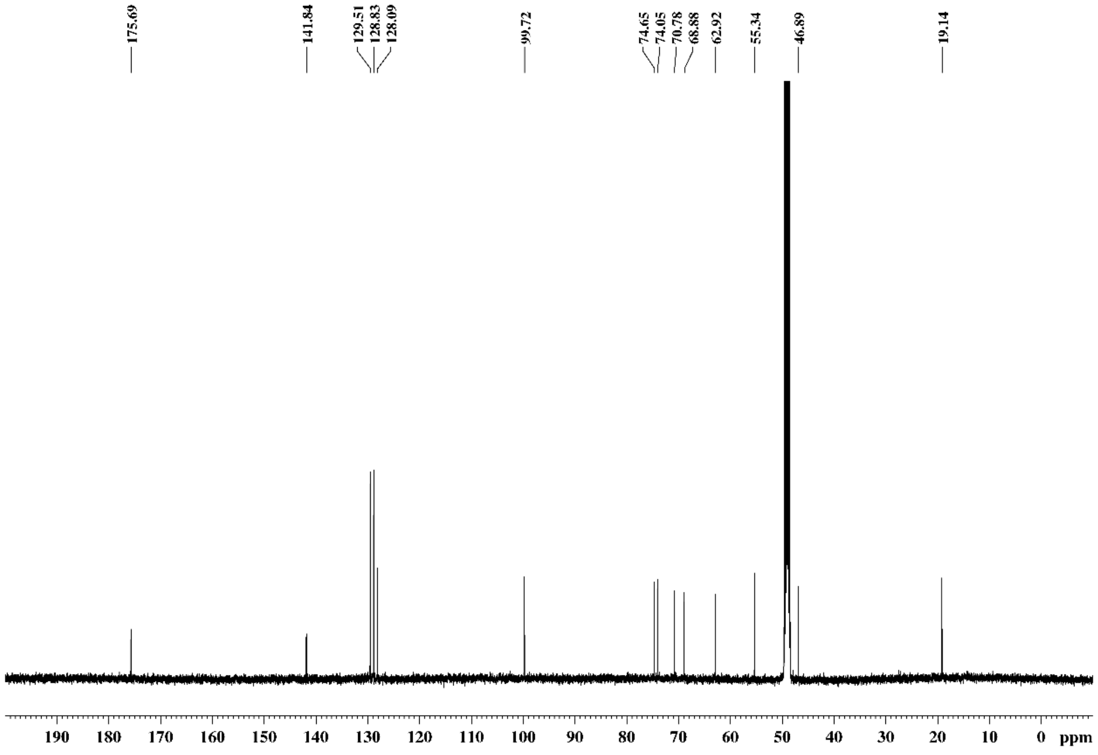

Methyl 3-*O*-benzyl-4,6-*O*-benzylidene-2-*O*-(*S*)-2-phenyl-propanoyl-α-D-mannopyranoside (85):

^{1}H NMR (500.20 MHz, $CDCl_3$, 25°C):

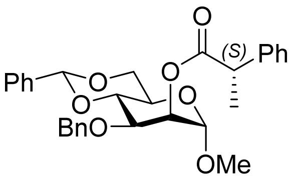

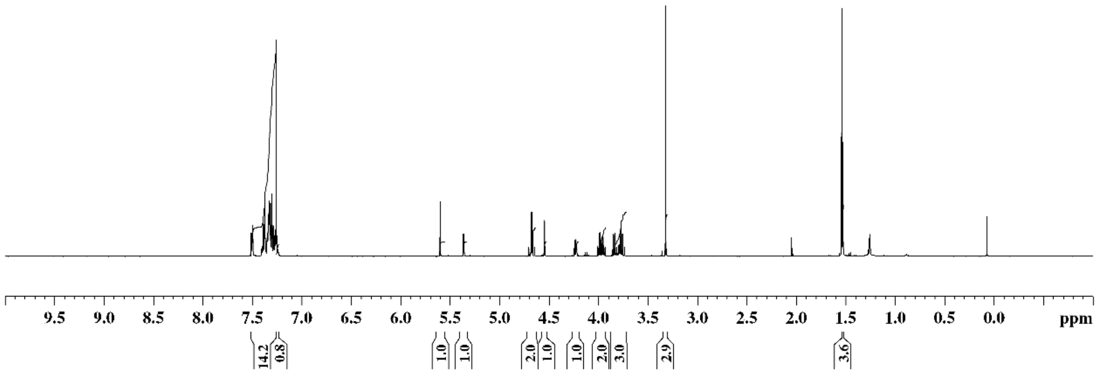

^{13}C NMR (125.8 MHz, $CDCl_3$, 25°C):

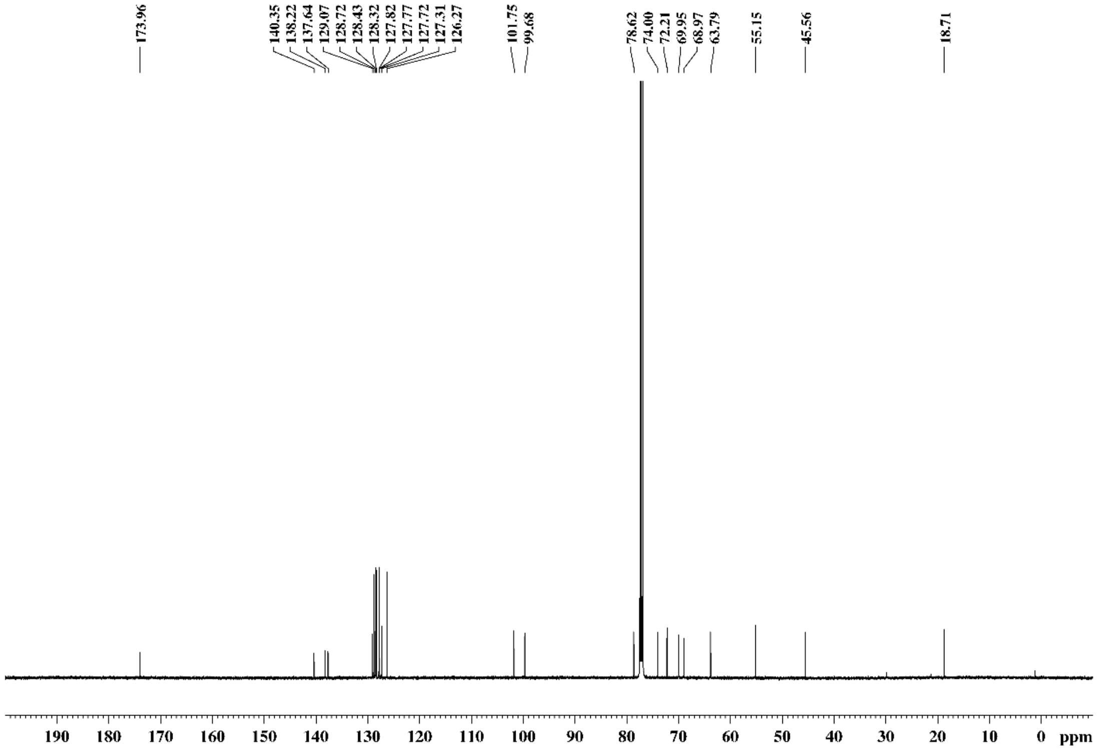

Methyl 2-*O*-(*S*)-2-phenyl-propanoyl-α-D-mannopyranoside (25):

^{1}H NMR (500.20 MHz, MeOD, 25°C):

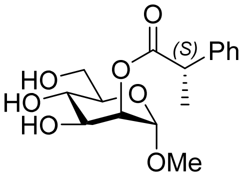

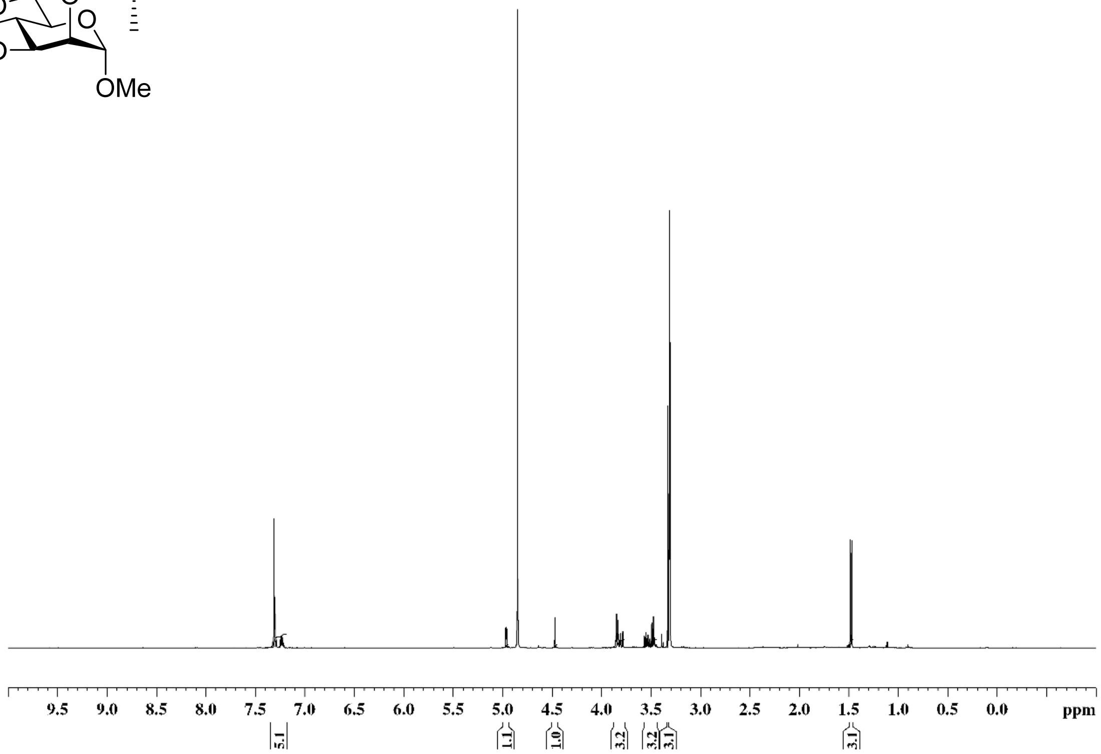

^{13}C NMR (125.8 MHz, MeOD, 25°C):

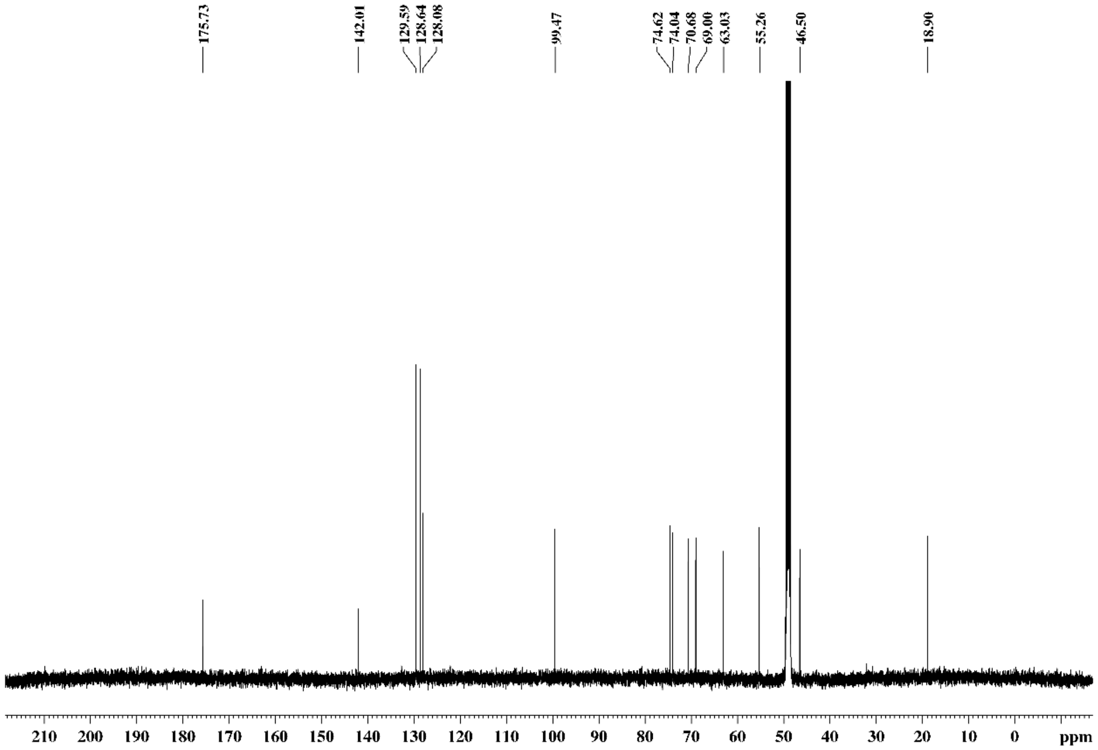

Methyl 4-*O*-acetyl-2,3-*O*-isopropylidene-β-D-xylopyranoside (87):

^{1}H NMR (500.20 MHz, $CDCl_3$, 25°C):

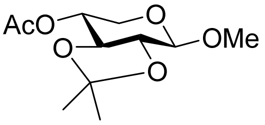

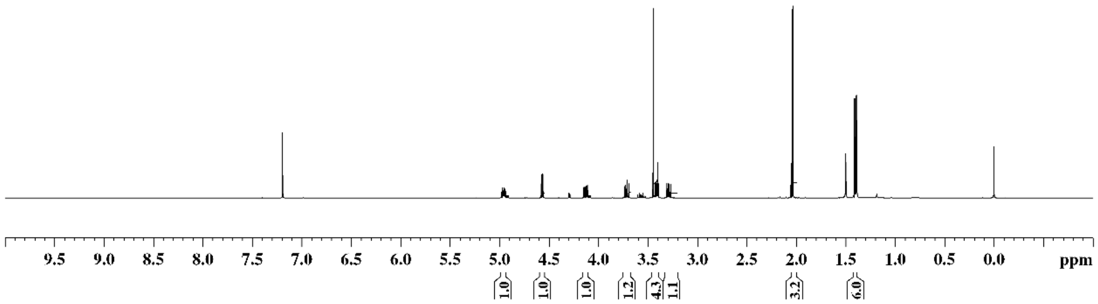

^{13}C NMR (125.8 MHz, $CDCl_3$, 25°C):

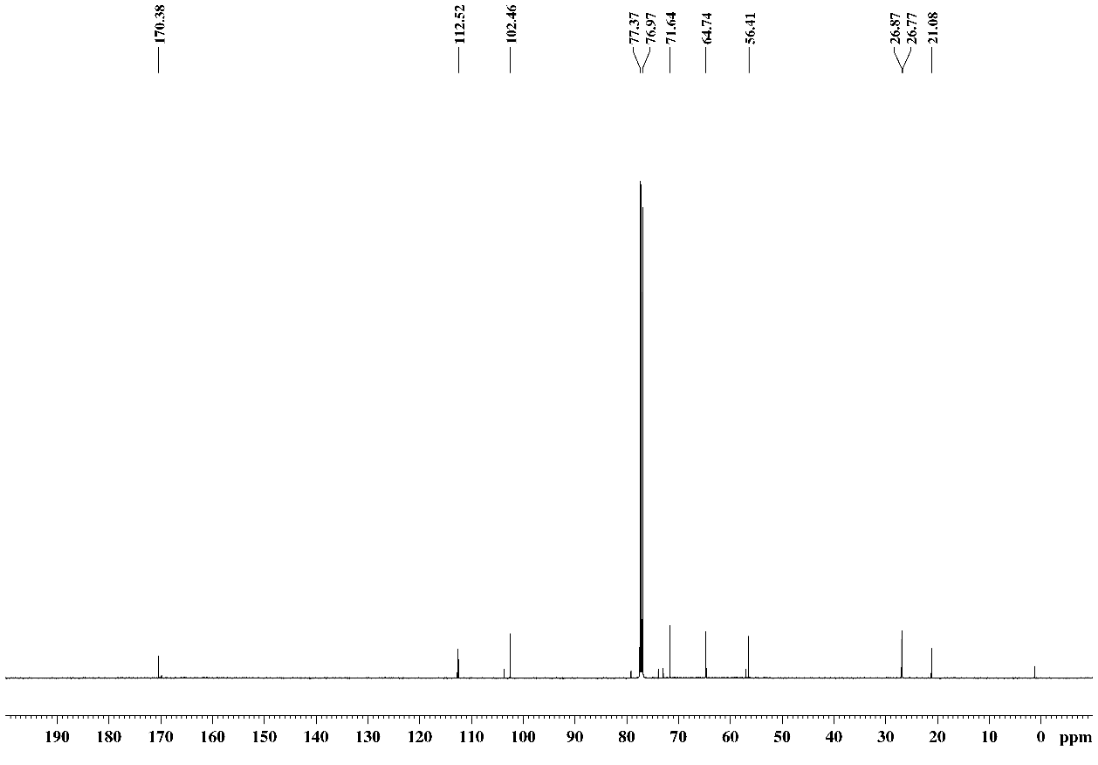

Methyl 4-*O*-acetyl-β-D-xylopyranoside (26):

^{1}H NMR (500.20 MHz, MeOD, 25°C):

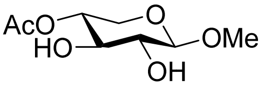

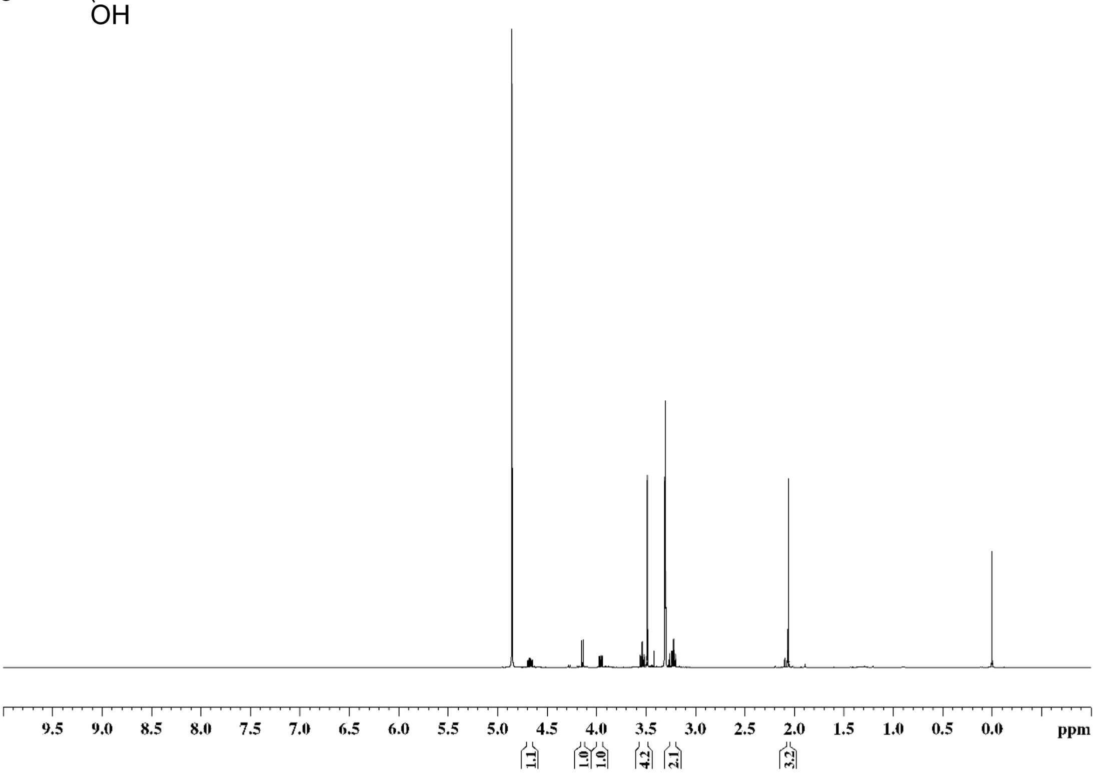

^{13}C NMR (125.8 MHz, MeOD, 25°C):

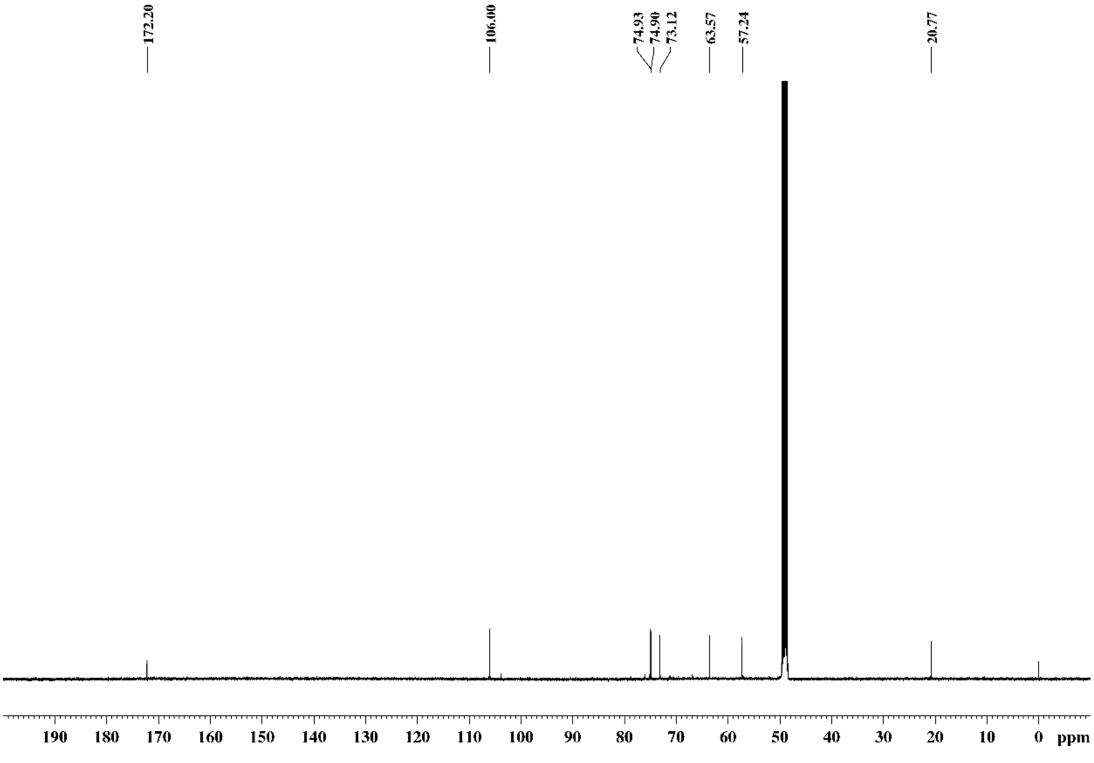

Methyl 4-*O*-benzoyl-2,3-*O*-isopropylidene-β-D-xylopyranoside (88):

^{1}H NMR (500.20 MHz, $CDCl_3$, 25°C):

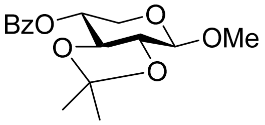

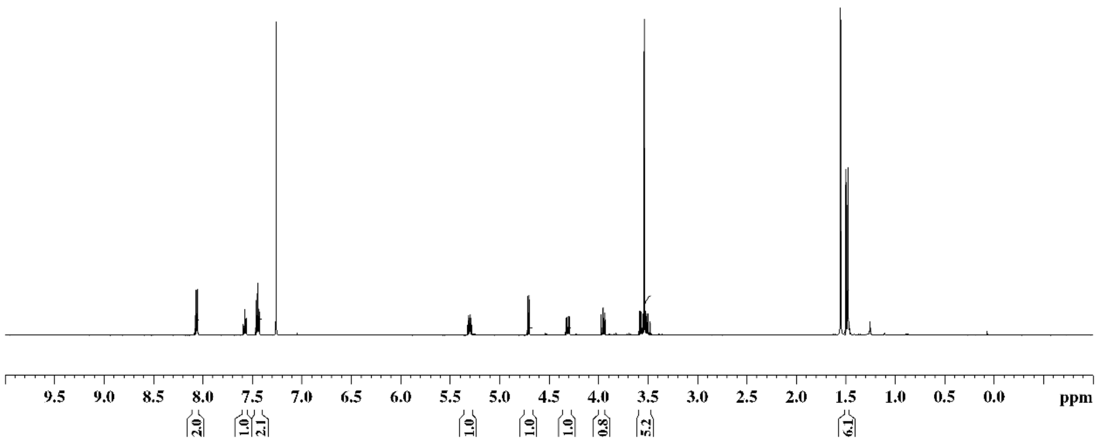

^{13}C NMR (125.8 MHz, $CDCl_3$, 25°C):

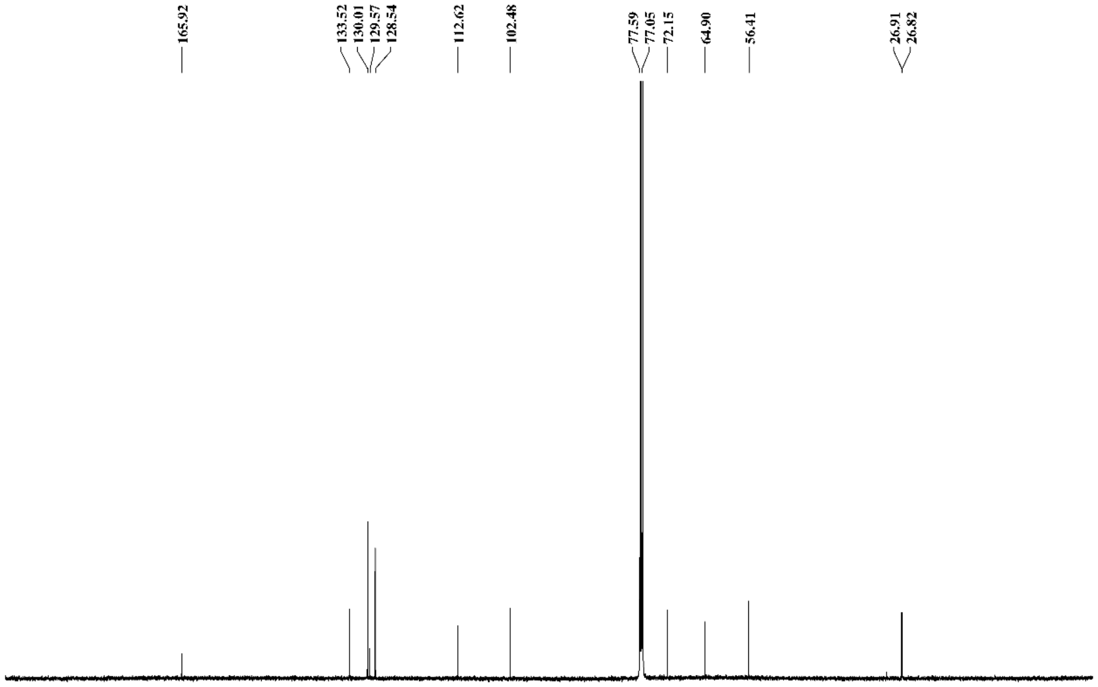

Methyl 4-*O*-benzoyl-β-D-xylopyranoside (27):

^{1}H NMR (500.20 MHz, MeOD, 25°C):

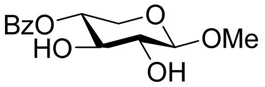

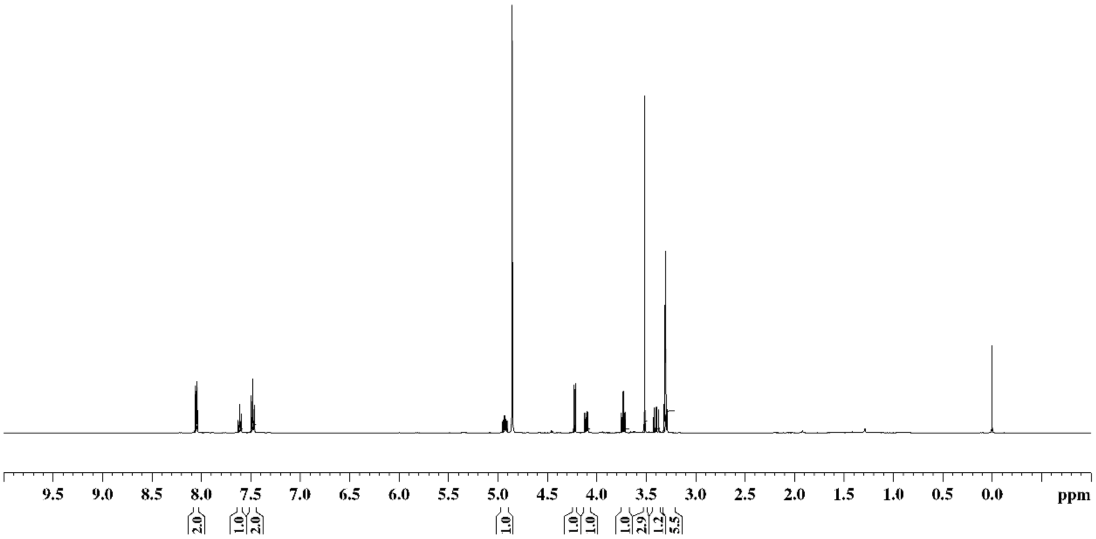

^{13}C NMR (125.8 MHz, MeOD, 25°C):

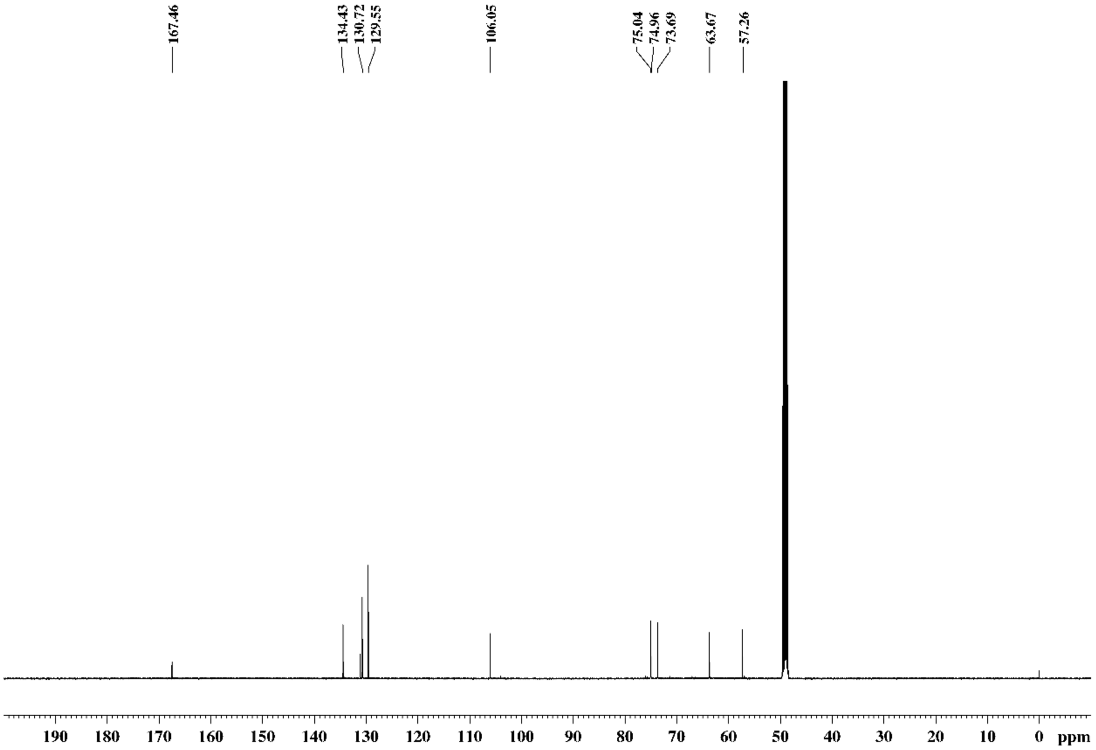

Methyl 2,3-*O*-isopropylidene-4-*O*-pivaoyl-β-D-xylopyranoside (89):

^{1}H NMR (500.20 MHz, $CDCl_3$, 25°C):

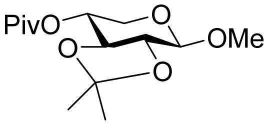

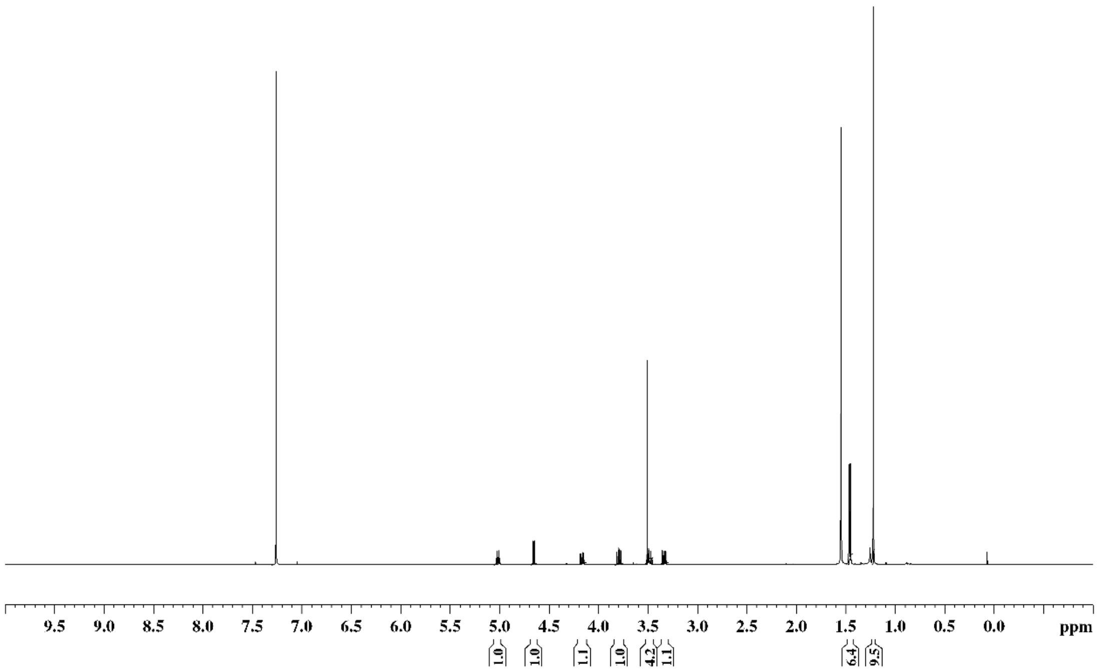

^{13}C NMR (125.8 MHz, $CDCl_3$, 25°C):

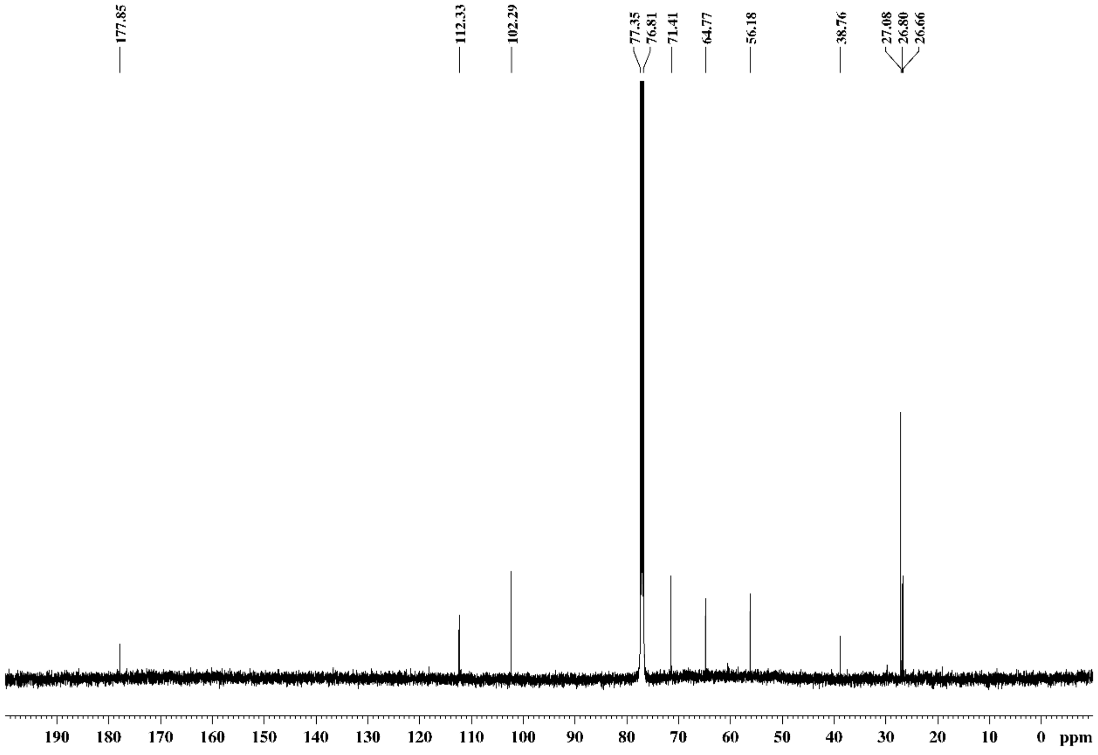

Methyl 4-*O*-pivaoyl-β-D-xylopyranoside (28):

^{1}H NMR (500.20 MHz, MeOD, 25°C):

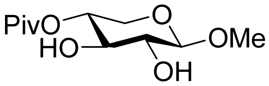

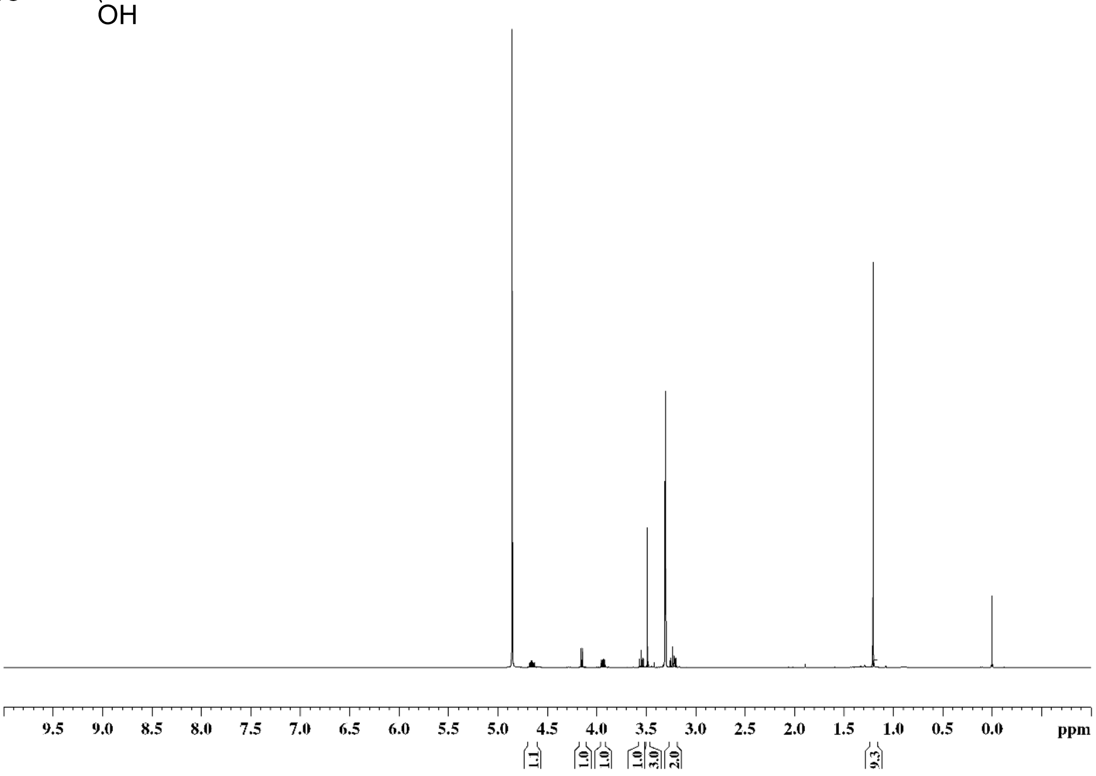

^{13}C NMR (125.8 MHz, MeOD, 25°C):

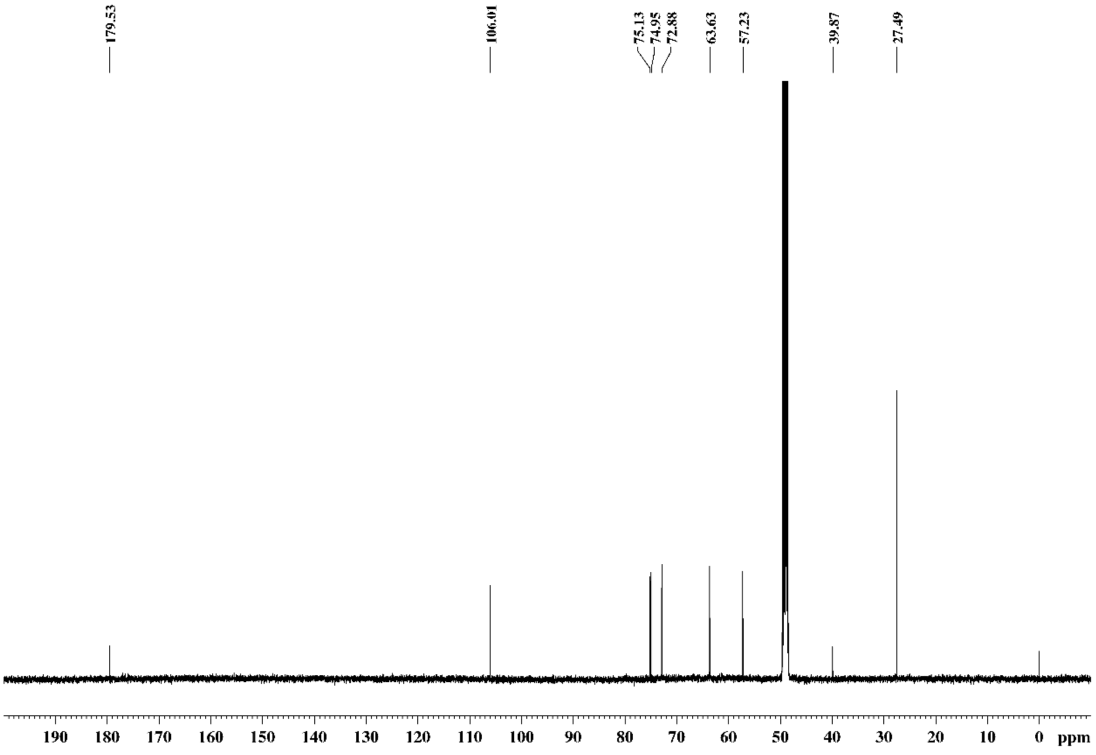

Methyl 2,3-*O*-isopropylidene-4-*O*-(*R*)-2-phenyl-propanoyl-β-D-xylopyranoside (90):

^{1}H NMR (500.20 MHz, $CDCl_3$, 25°C):

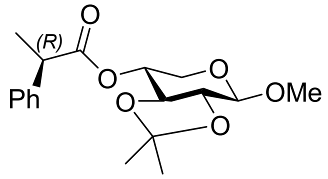

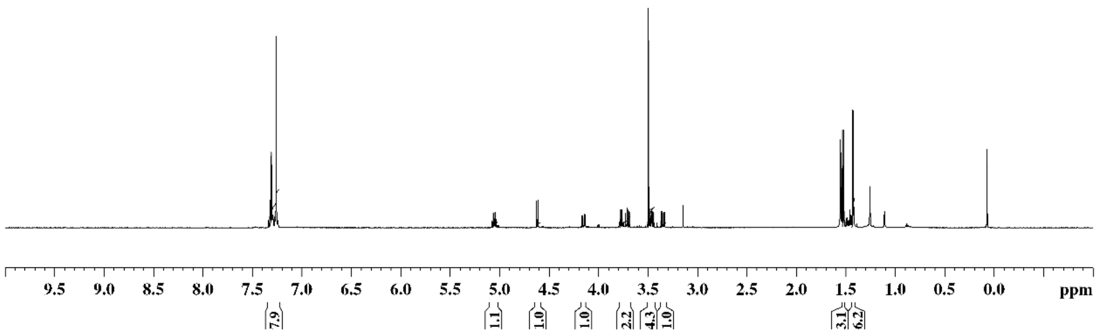

^{13}C NMR (125.8 MHz, $CDCl_3$, 25°C):

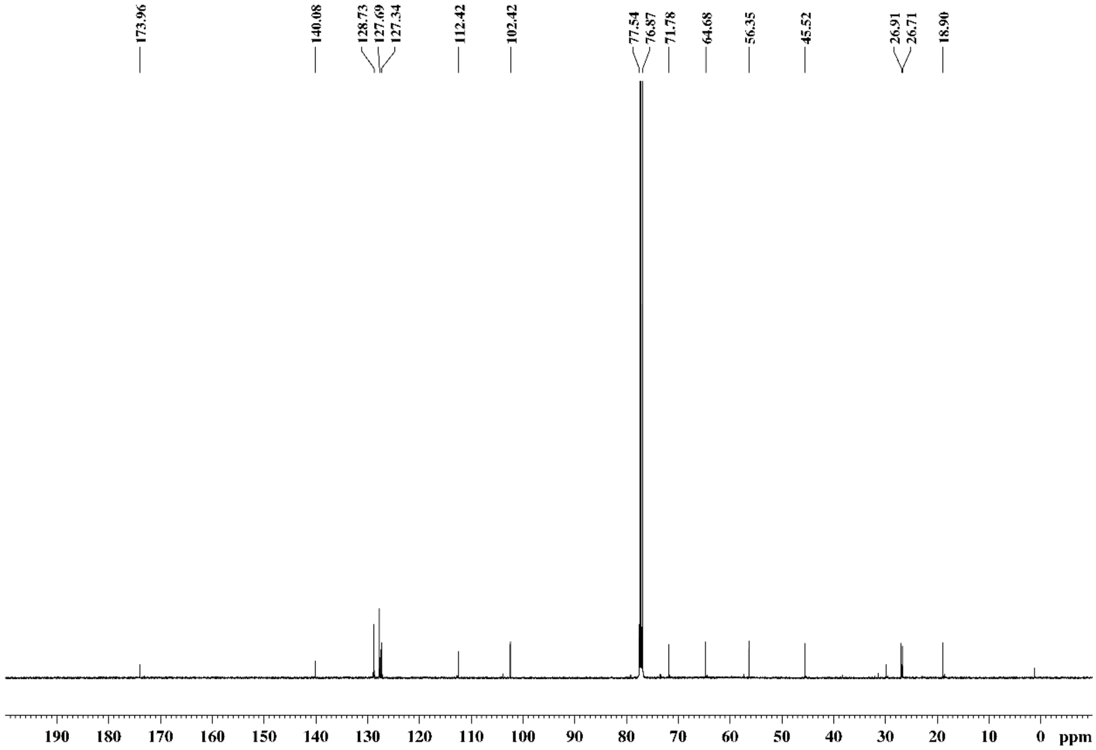

Methyl 4-*O*-(*R*)-2-phenyl-propanoyl-β-D-xylopyranoside (29):

^{1}H NMR (500.20 MHz, MeOD, 25°C):

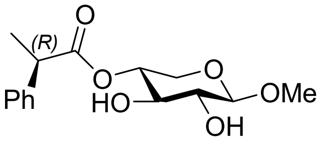

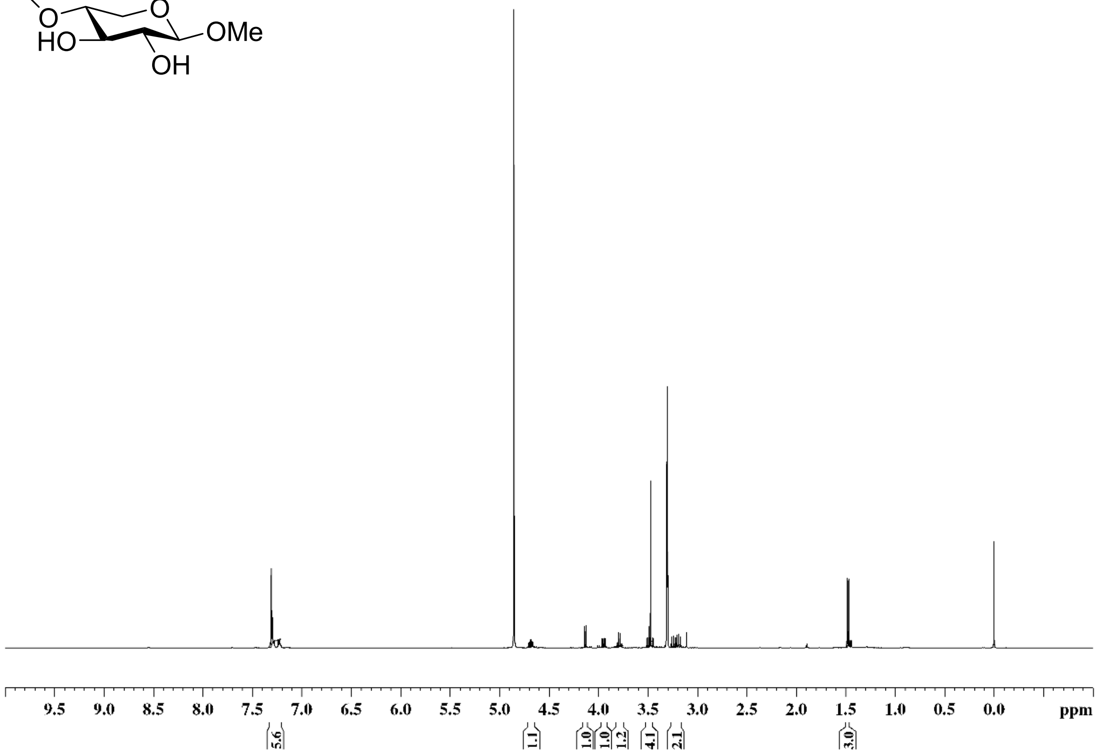

^{13}C NMR (125.8 MHz, MeOD, 25°C):

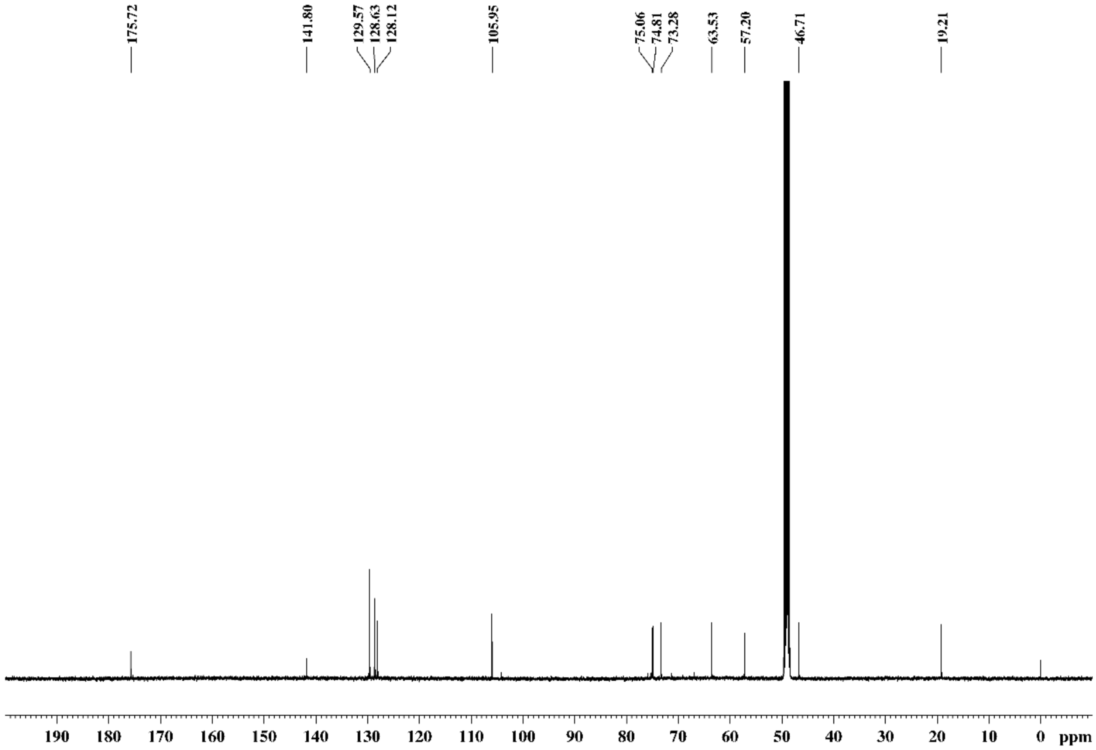

Methyl 2,3-*O*-isopropylidene-4-*O*-(*S*)-2-phenyl-propanoyl-β-D-xylopyranoside (91):

^{1}H NMR (500.20 MHz, $CDCl_3$, 25°C):

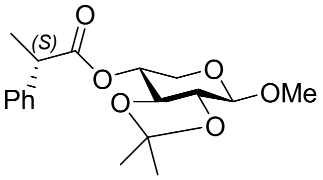

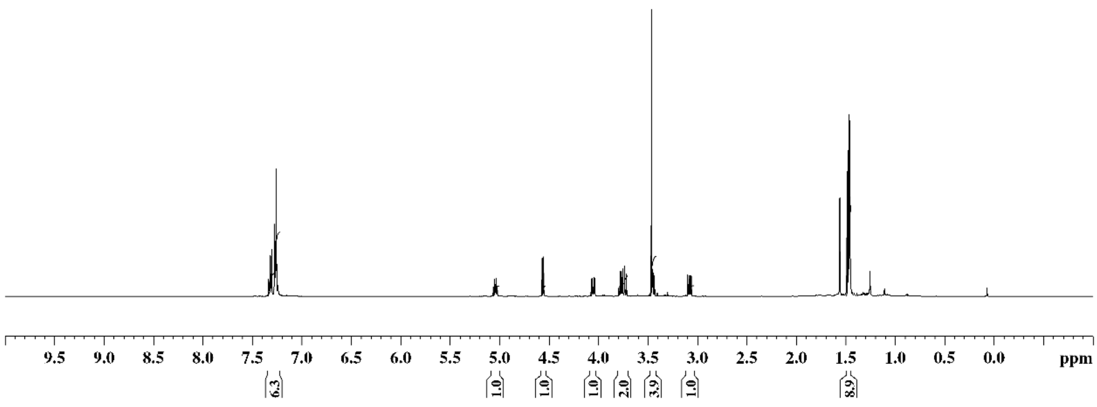

^{13}C NMR (125.8 MHz, $CDCl_3$, 25°C):

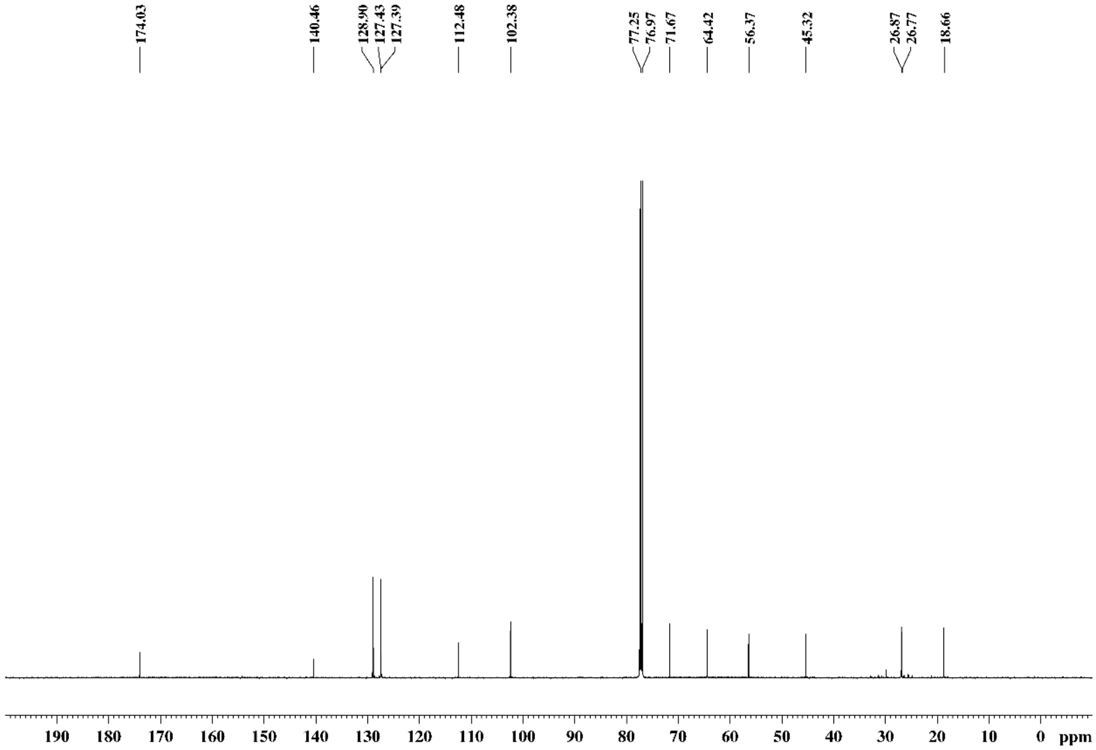

Methyl 4-*O*-(*S*)-2-phenyl-propanoyl-β-D-xylopyranoside (30):

^{1}H NMR (500.20 MHz, MeOD, 25°C):

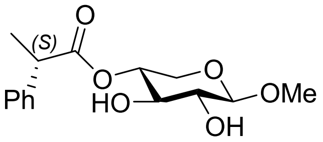

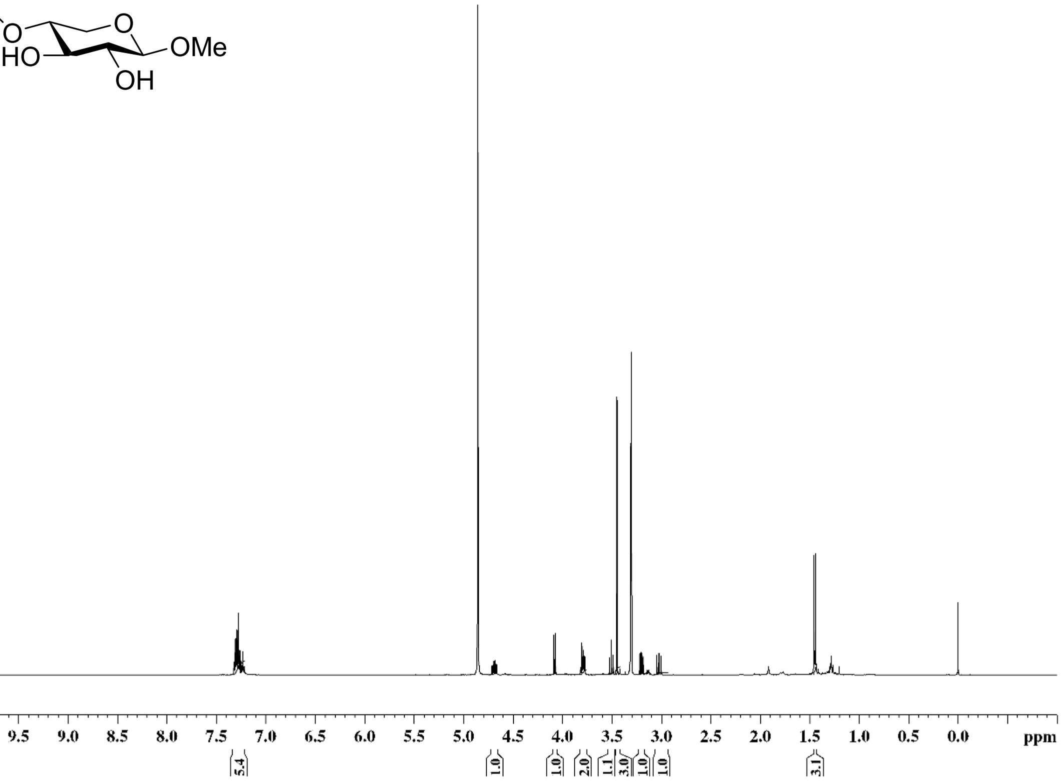

^{13}C NMR (125.8 MHz, MeOD, 25°C):

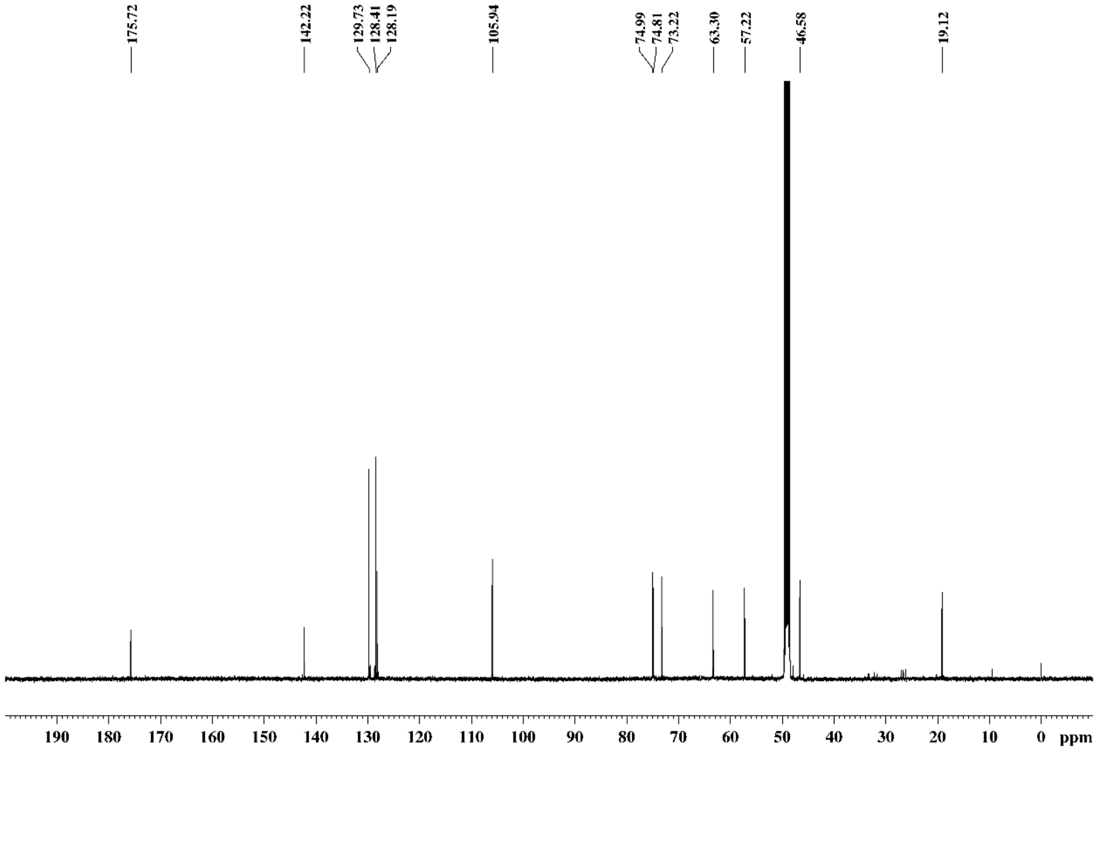

Methyl 4-*O*-acetyl-2,3-*O*-isopropylidene-α-D-xylopyranoside (93):

^{1}H NMR (500.20 MHz, $CDCl_3$, 25°C):

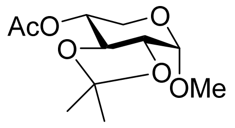

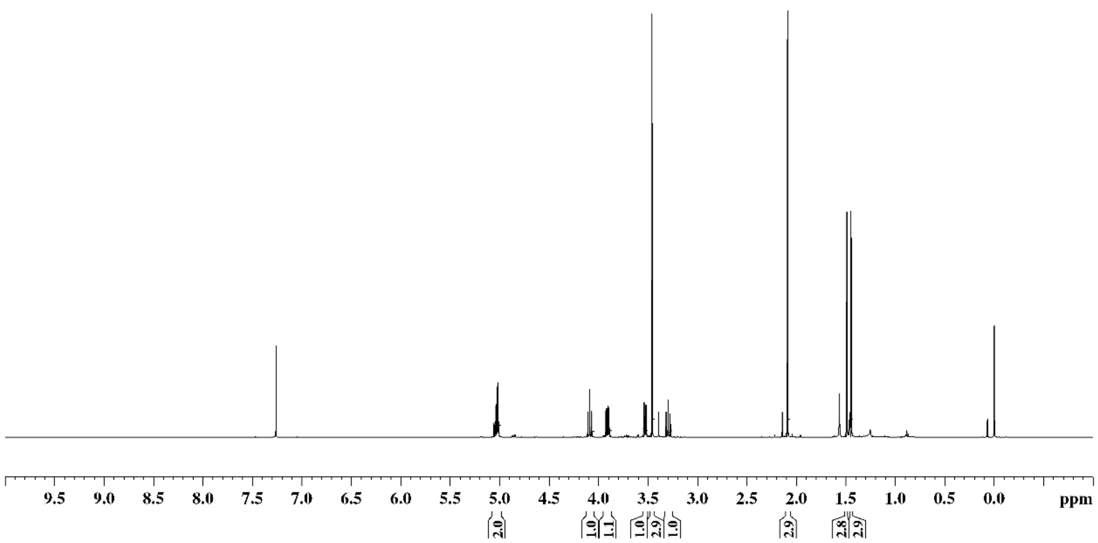

^{13}C NMR (125.8 MHz, $CDCl_3$, 25°C):

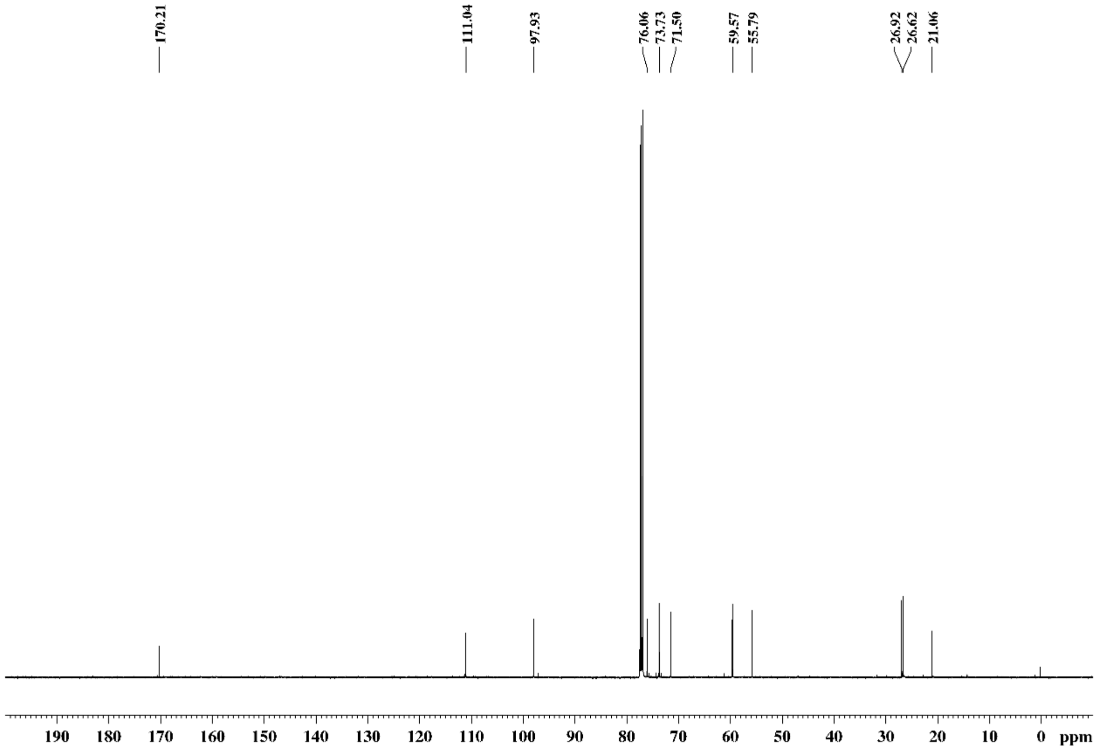

Methyl 4-*O*-acetyl-α-D-xylopyranoside (31):

^{1}H NMR (500.20 MHz, MeOD, 25°C):

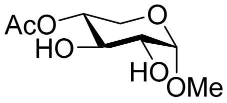

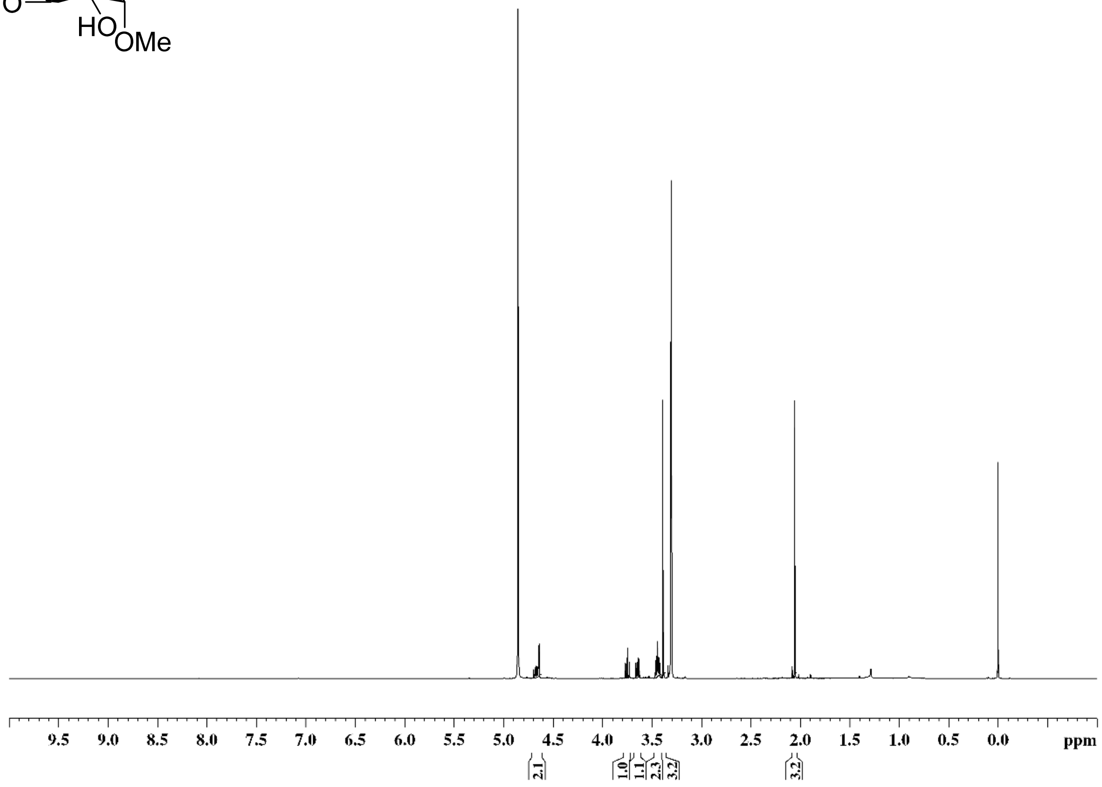

^{13}C NMR (125.8 MHz, MeOD, 25°C):

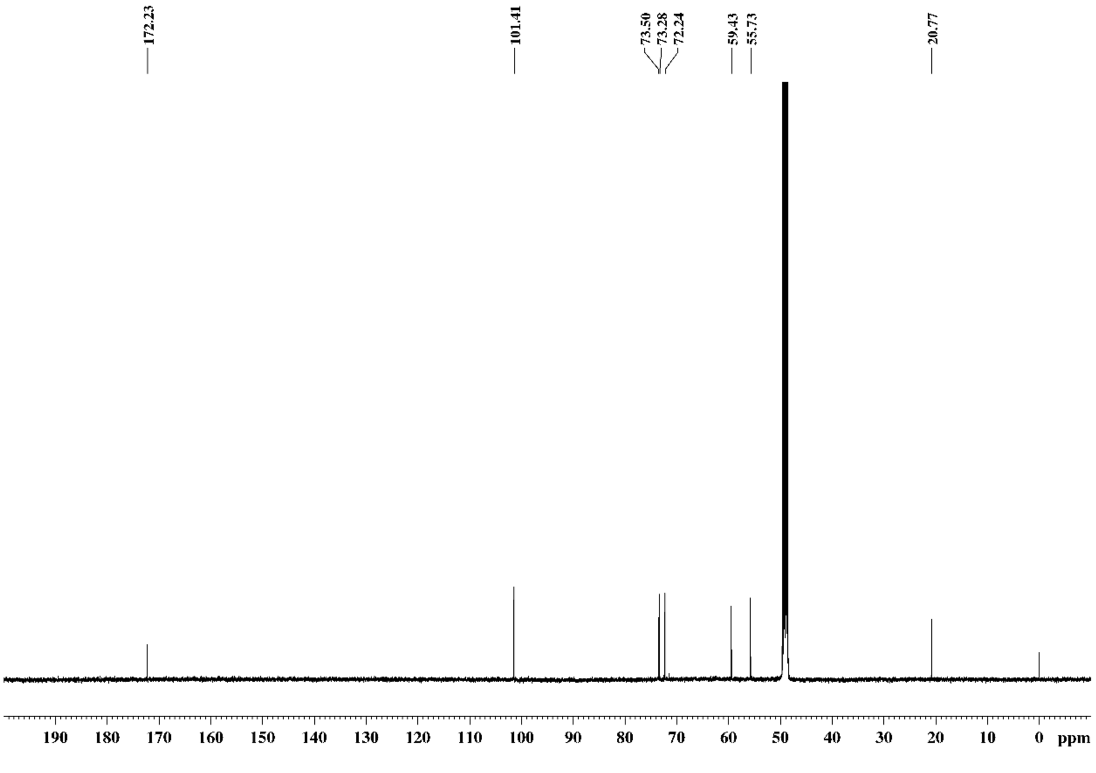

Methyl 4-*O*-benzoyl-2,3-*O*-isopropylidene-α-D-xylopyranoside (94):

^{1}H NMR (500.20 MHz, $CDCl_3$, 25°C):

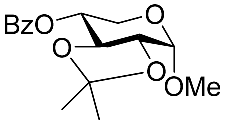

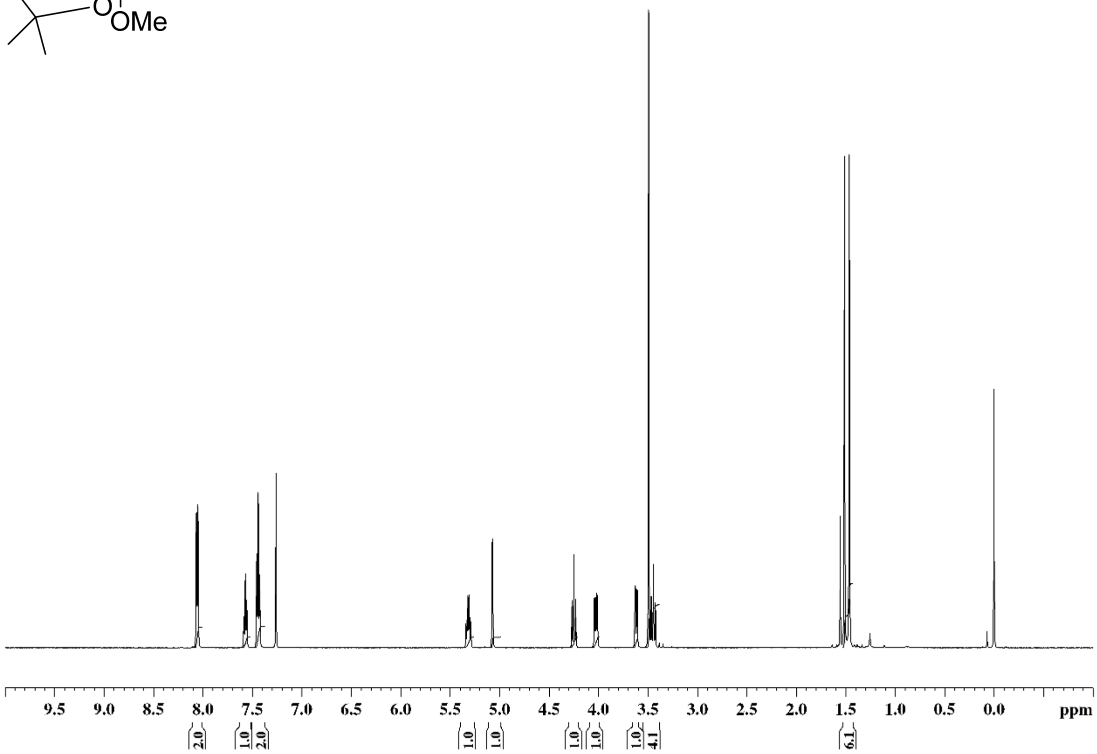

^{13}C NMR (125.8 MHz, $CDCl_3$, 25°C):

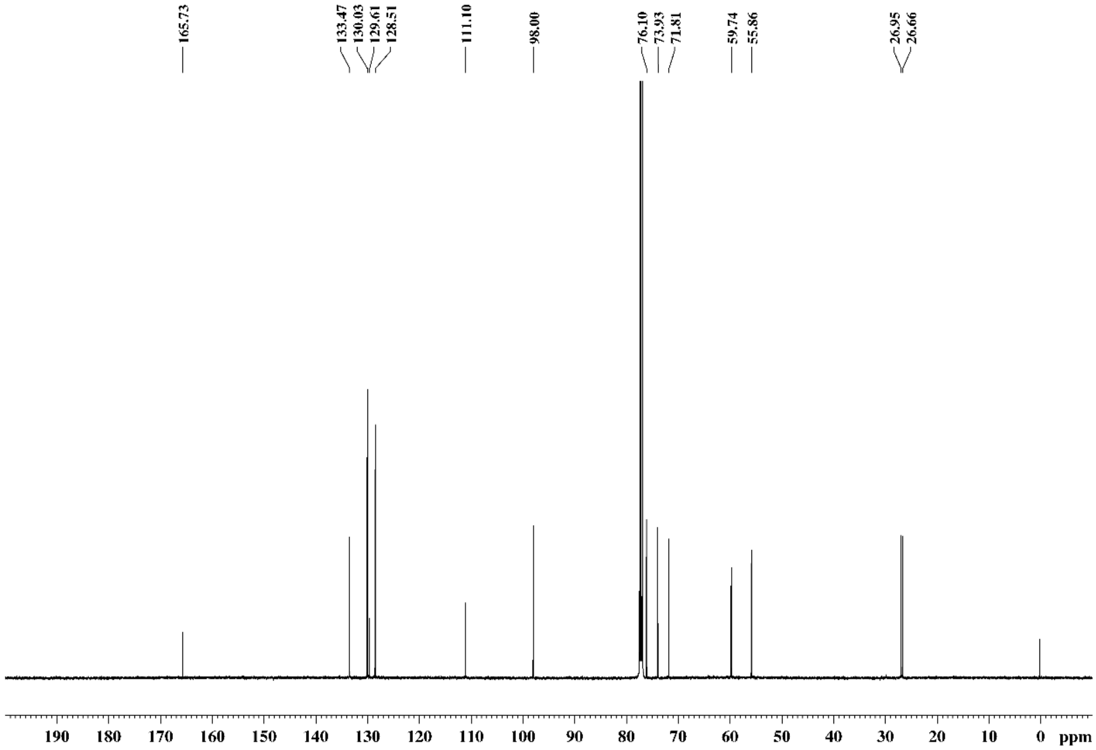

Methyl 4-*O*-benzoyl-α-D-xylopyranoside (32):

^{1}H NMR (500.20 MHz, MeOD, 25°C):

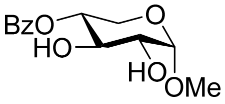

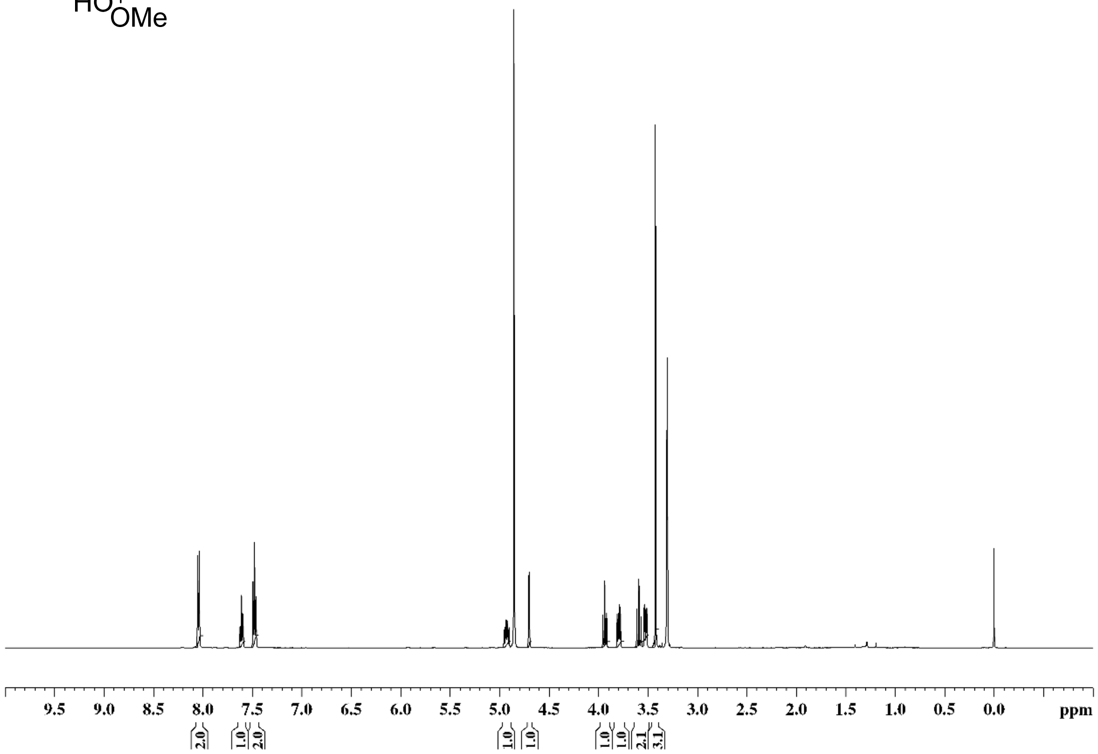

^{13}C NMR (125.8 MHz, MeOD, 25°C):

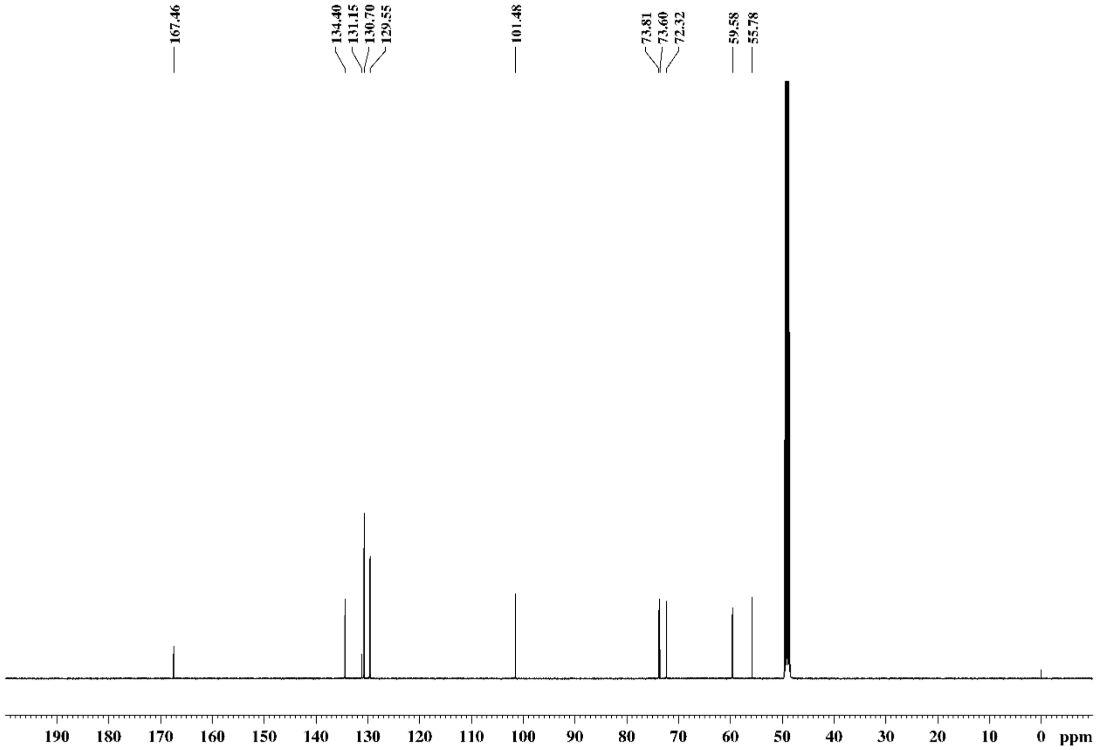

Methyl 2,3-*O*-isopropylidene-4-*O*-pivaoyl-α-D-xylopyranoside (95):

^{1}H NMR (500.20 MHz, $CDCl_3$, 25°C):

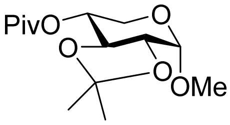

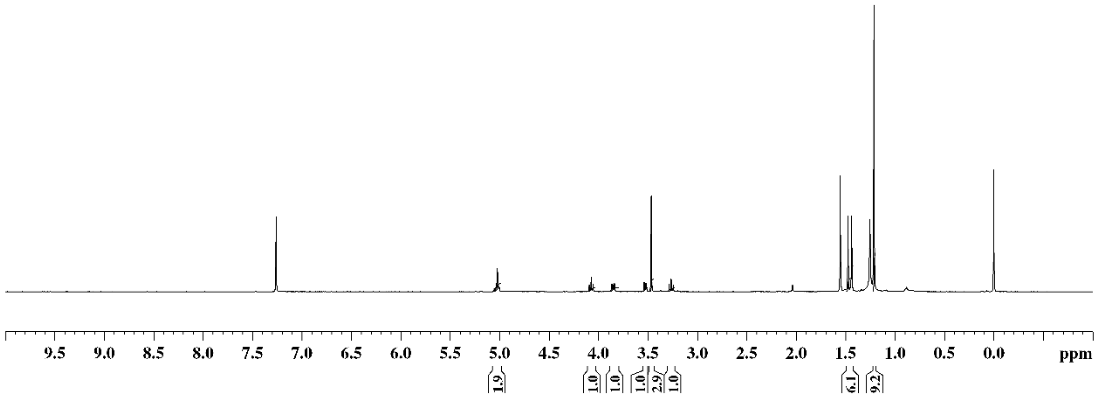

^{13}C NMR (125.8 MHz, $CDCl_3$, 25°C):

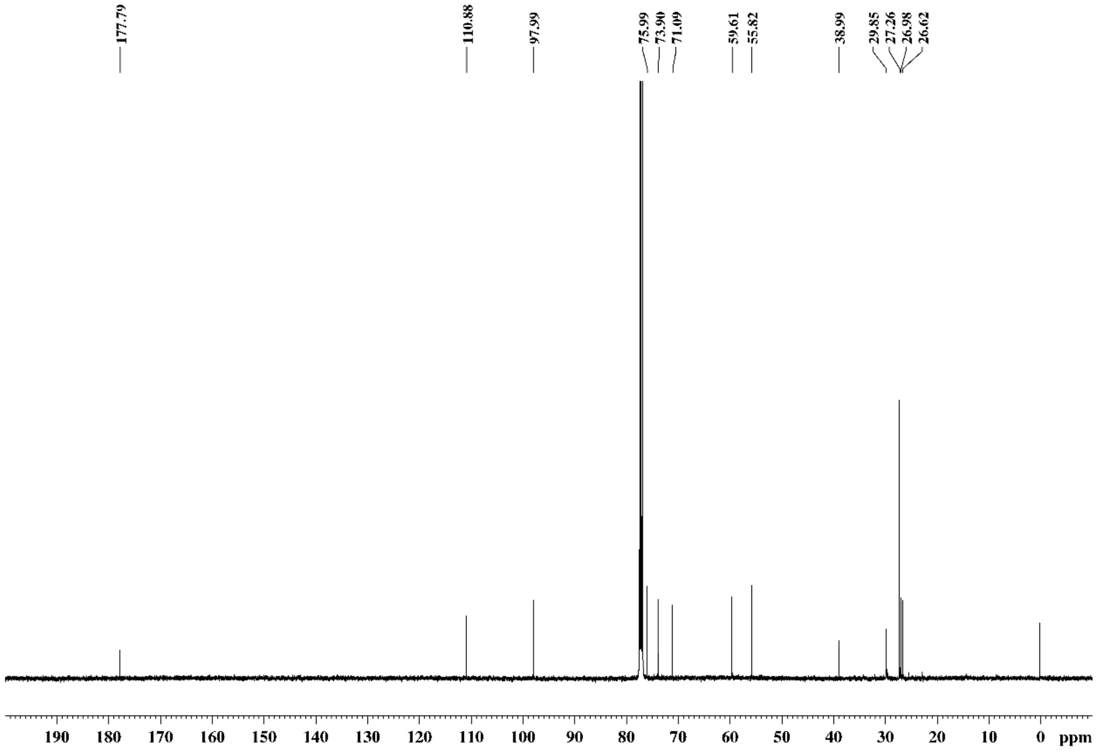

Methyl 4-*O*-pivaoyl-α-D-xylopyranoside (33):

^{1}H NMR (500.20 MHz, MeOD, 25°C):

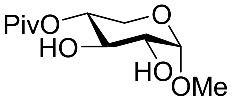

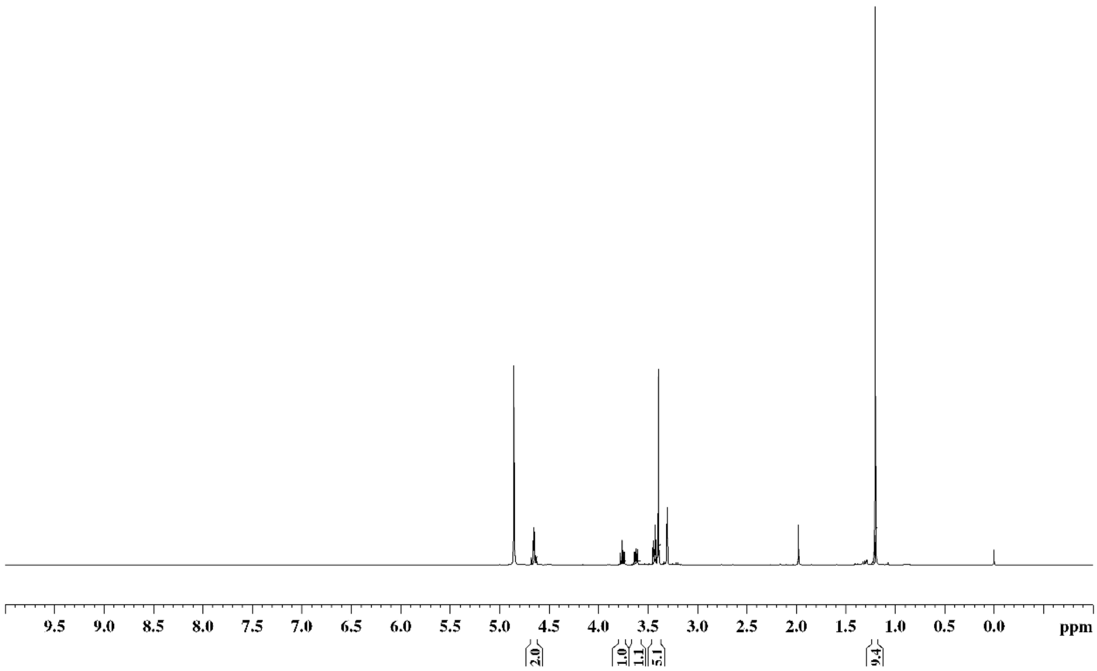

^{13}C NMR (125.8 MHz, MeOD, 25°C):

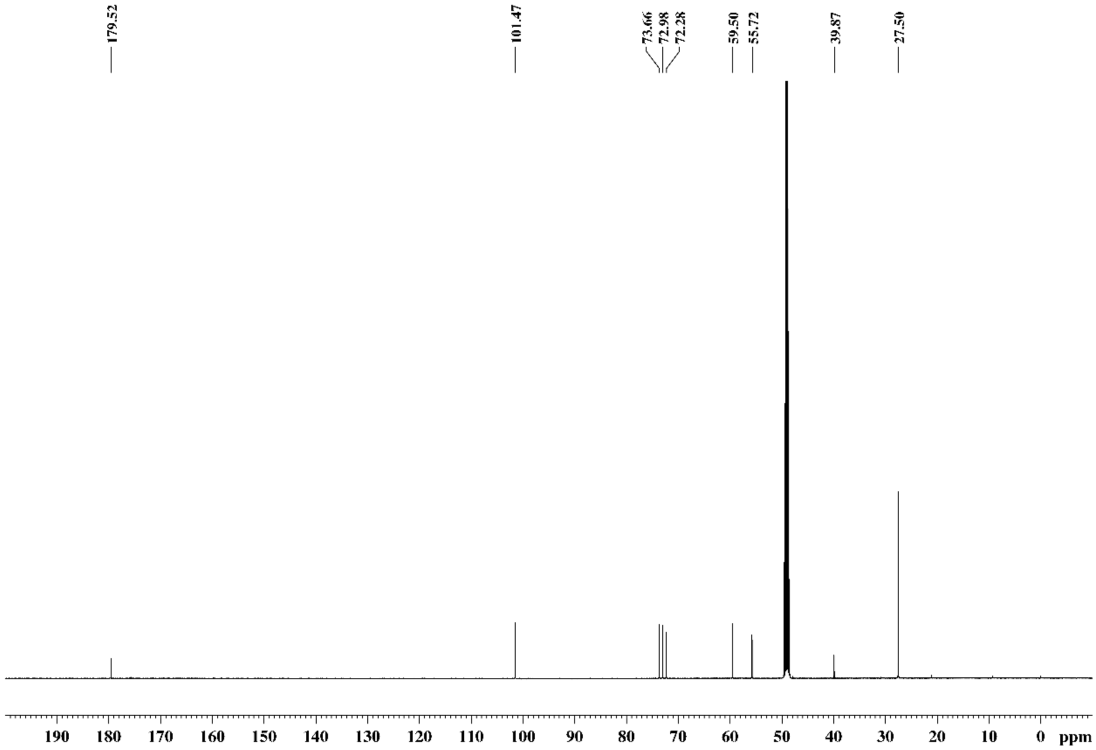

Methyl 2,3-*O*-isopropylidene-4-*O*-(*R*)-2-phenyl-propanoyl-α-D-xylopyranoside (96):

^{1}H NMR (500.20 MHz, $CDCl_3$, 25°C):

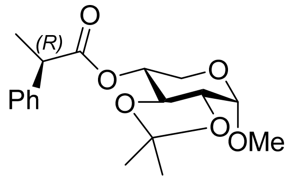

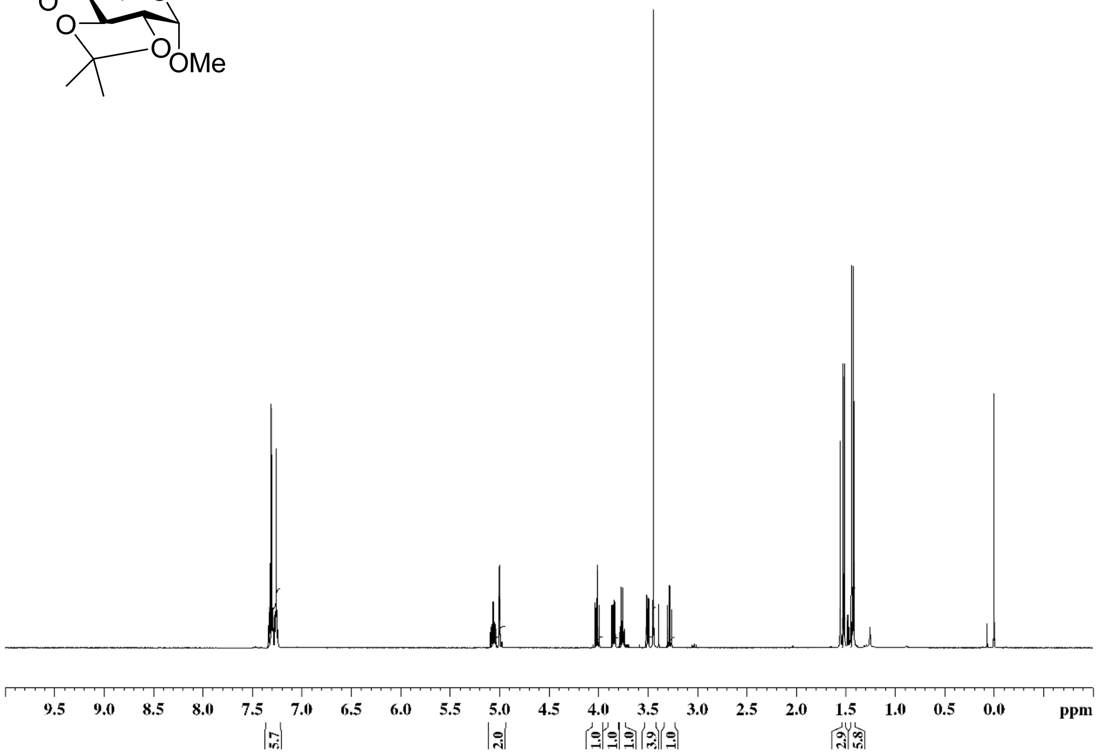

^{13}C NMR (125.8 MHz, $CDCl_3$, 25°C):

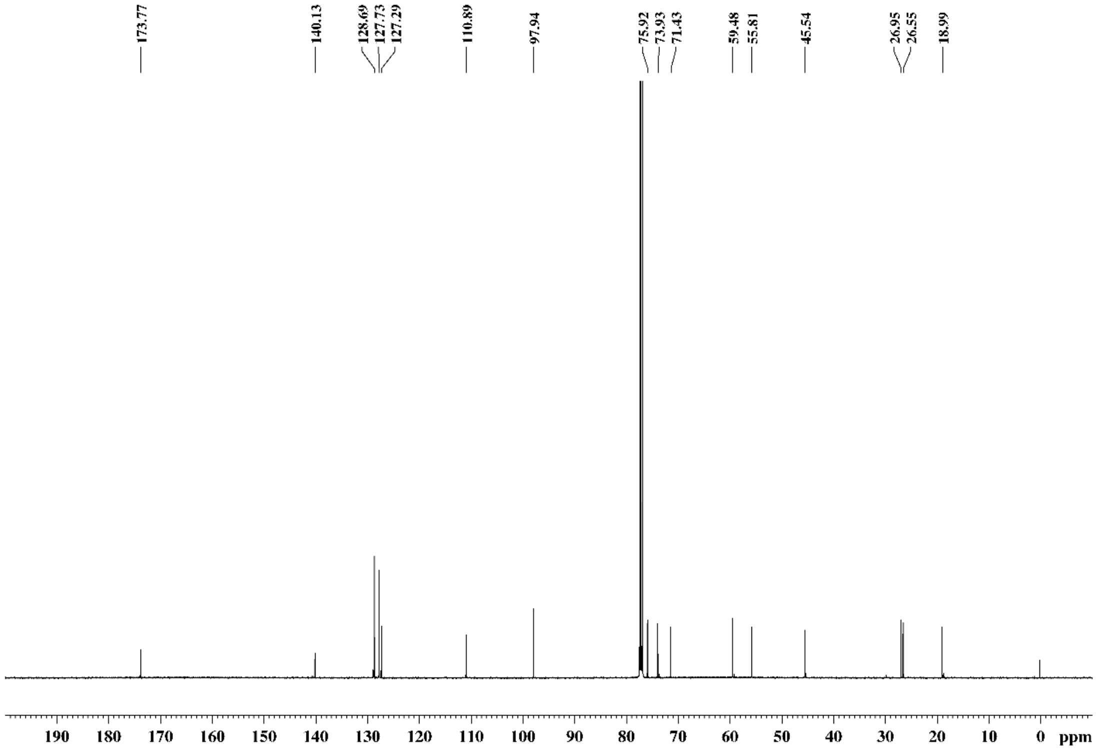

Methyl 4-*O*-(*R*)-2-phenyl-propanoyl-α-D-xylopyranoside (34):

^{1}H NMR (500.20 MHz, MeOD, 25°C):

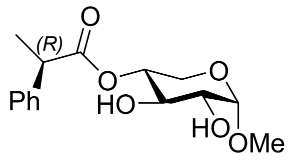

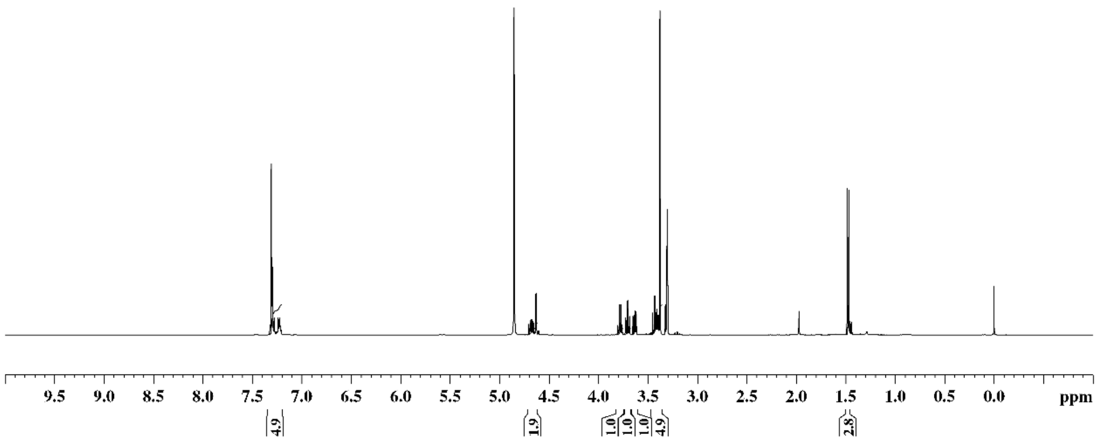

^{13}C NMR (125.8 MHz, MeOD, 25°C):

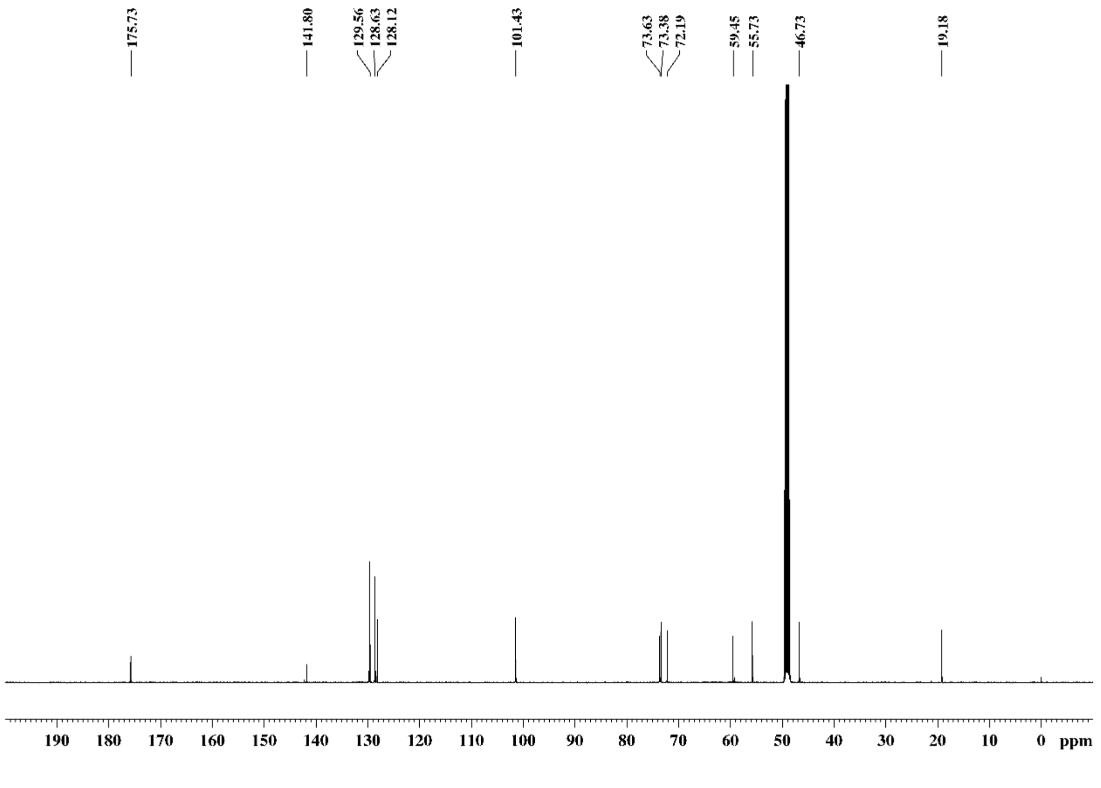

Methyl 2,3-*O*-isopropylidene-4-*O*-(*S*)-2-phenyl-propanoyl-α-D-xylopyranoside (97):

^{1}H NMR (500.20 MHz, $CDCl_3$, 25°C):

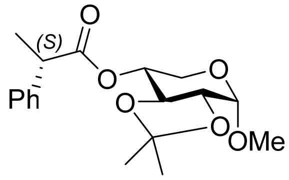

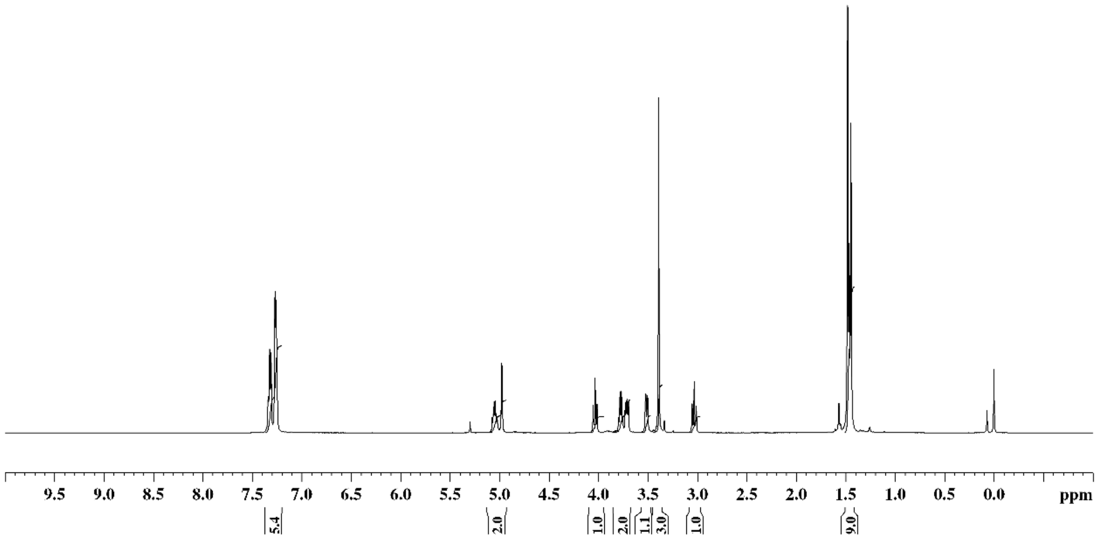

^{13}C NMR (125.8 MHz, $CDCl_3$, 25°C):

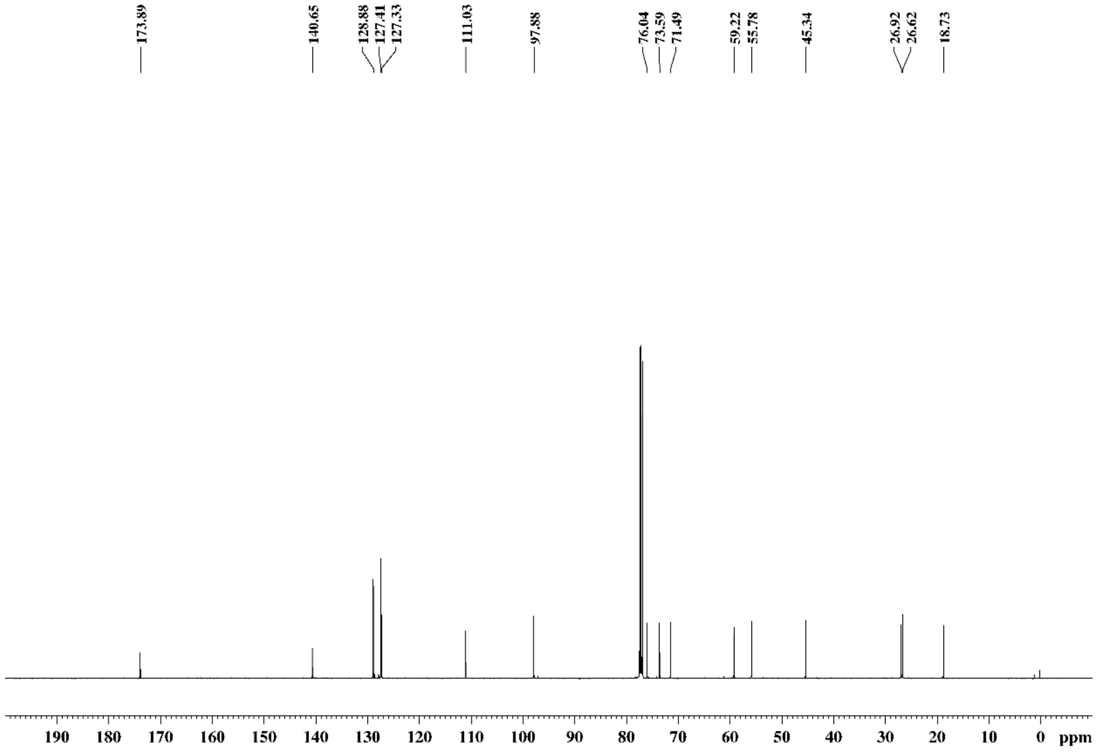

Methyl 4-*O*-(*S*)-2-phenyl-propanoyl-α-D-xylopyranoside (35):

^{1}H NMR (500.20 MHz, MeOD, 25°C):

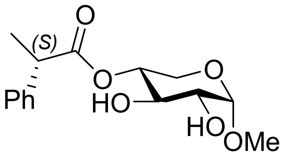

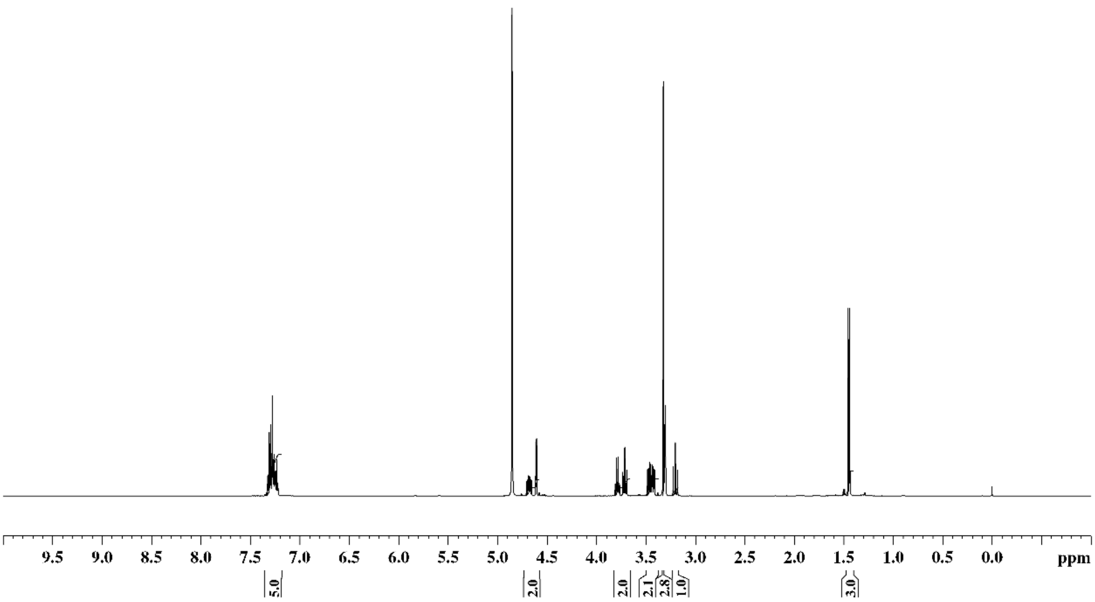

^{13}C NMR (125.8 MHz, MeOD, 25°C):

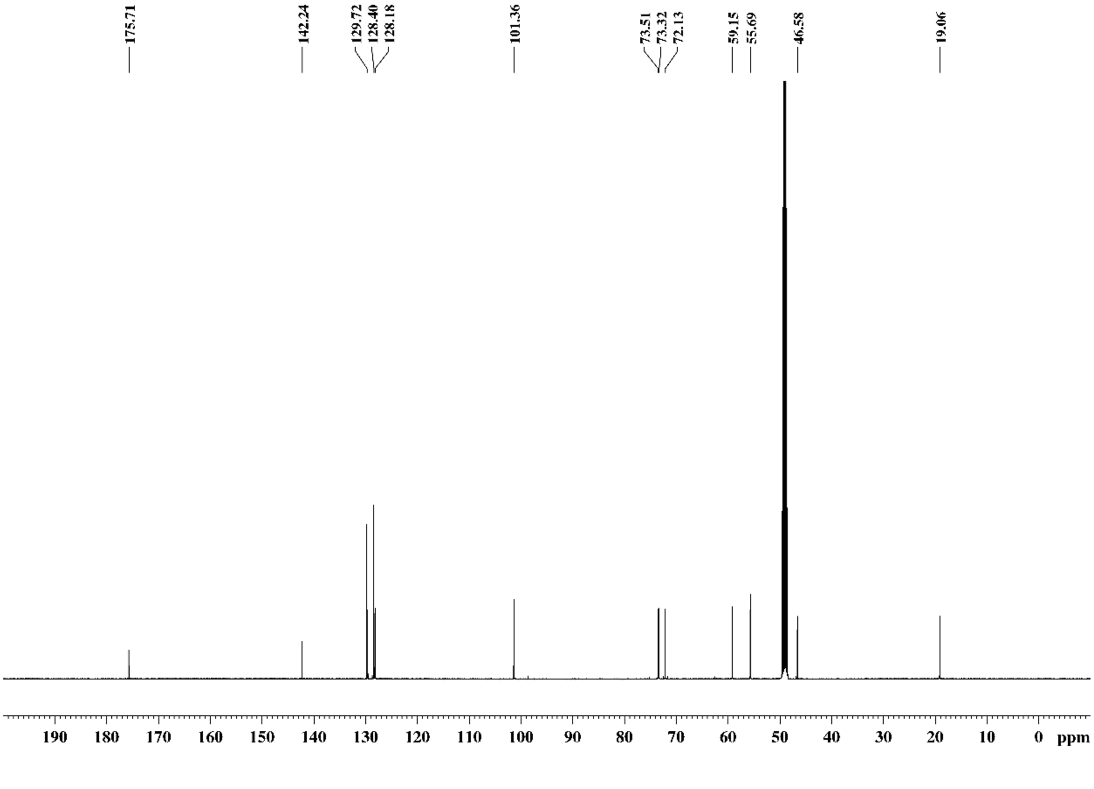

Methyl 2-*O*-acetyl-3,4-*O*-isopropylidene-β-D-ribopyranoside (99):

^{1}H NMR (500.20 MHz, $CDCl_3$, 25°C):

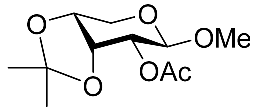

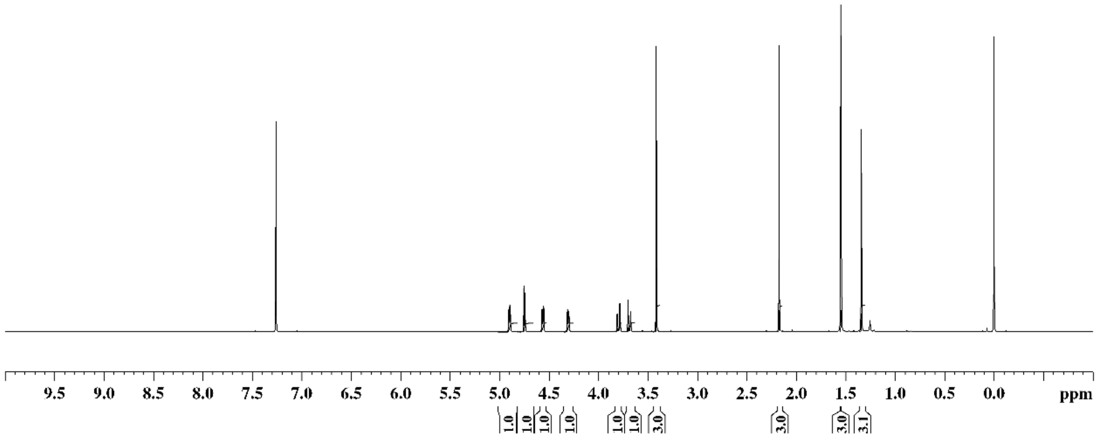

^{13}C NMR (125.8 MHz, $CDCl_3$, 25°C):

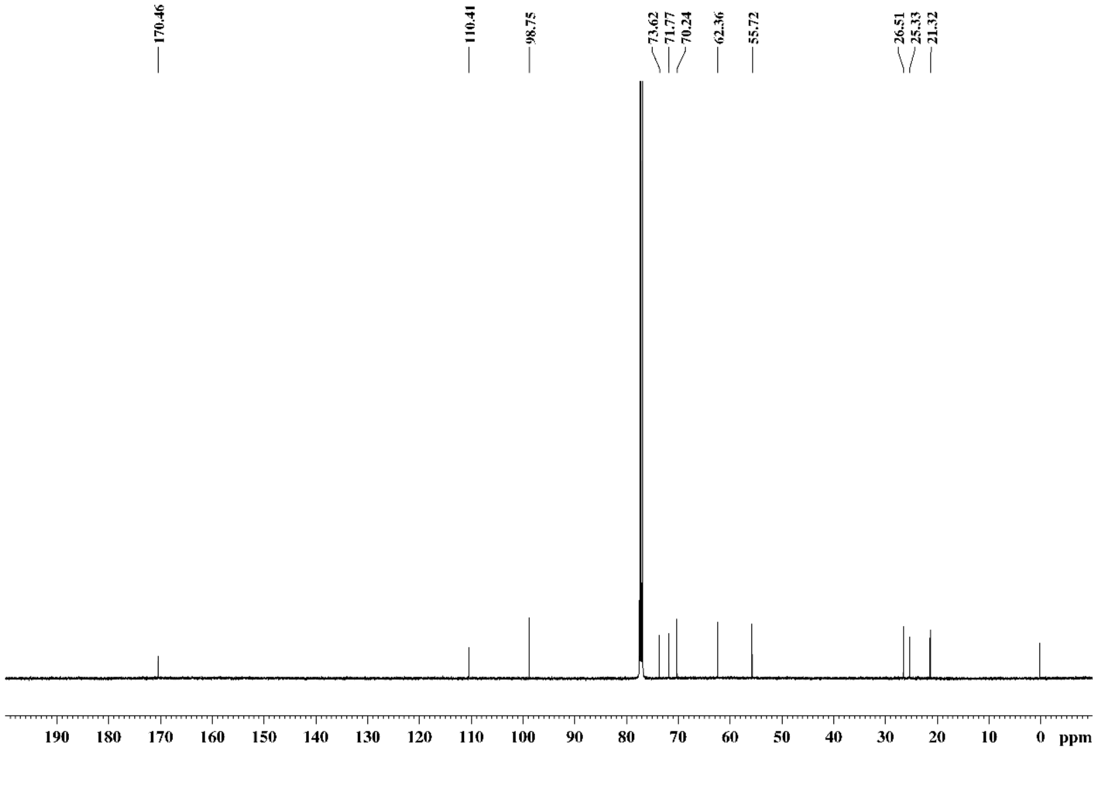

Methyl 2-*O*-acetyl-β-D-ribopyranoside (36):

^{1}H NMR (500.20 MHz, MeOD, 25°C):

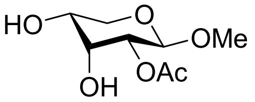

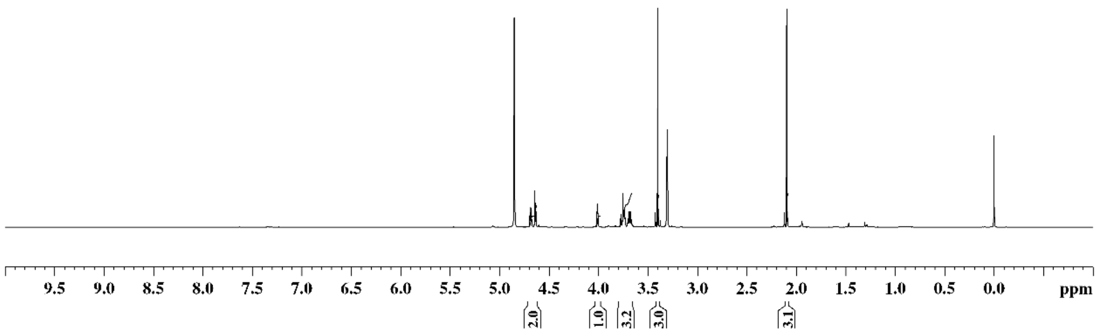

^{13}C NMR (125.8 MHz, MeOD, 25°C):

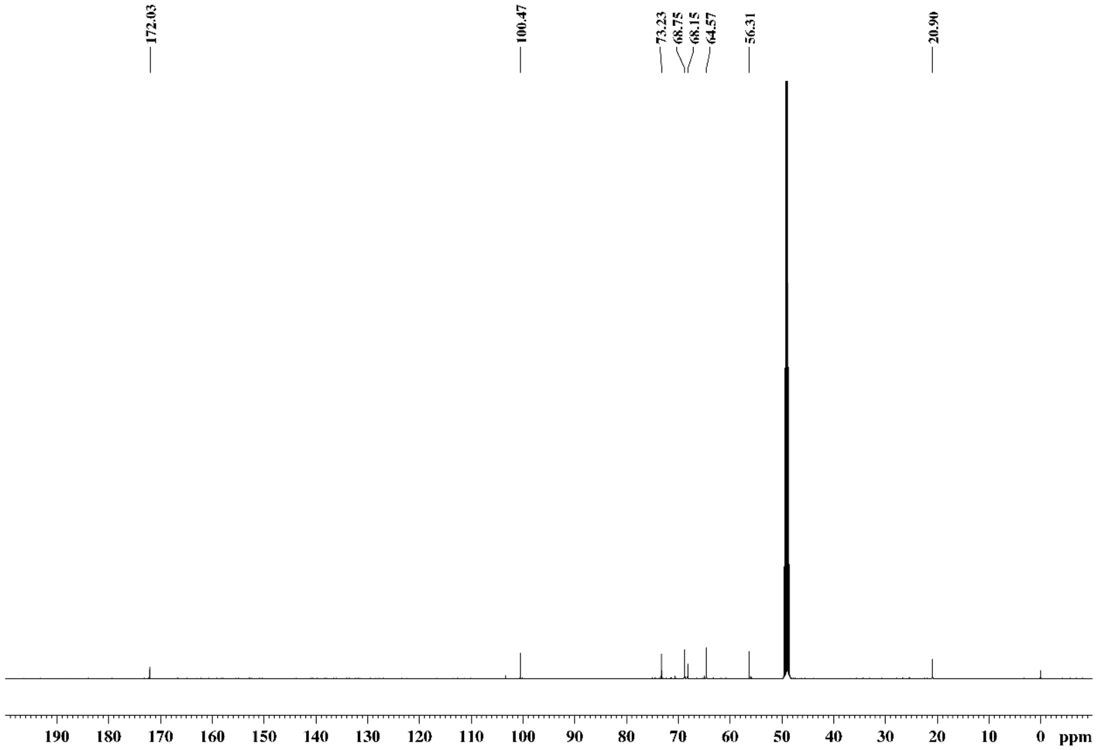

Methyl 2-*O*-benzoyl-3,4-*O*-isopropylidene-β-D-ribopyranoside (100):

^{1}H NMR (500.20 MHz, $CDCl_3$, 25°C):

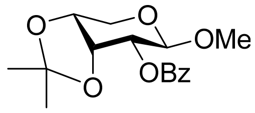

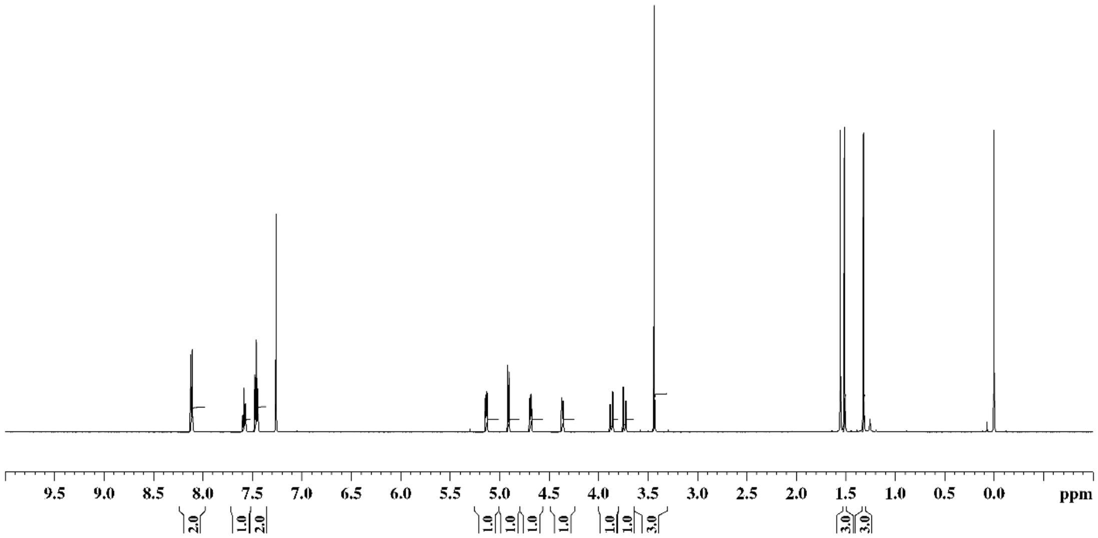

^{13}C NMR (125.8 MHz, $CDCl_3$, 25°C):

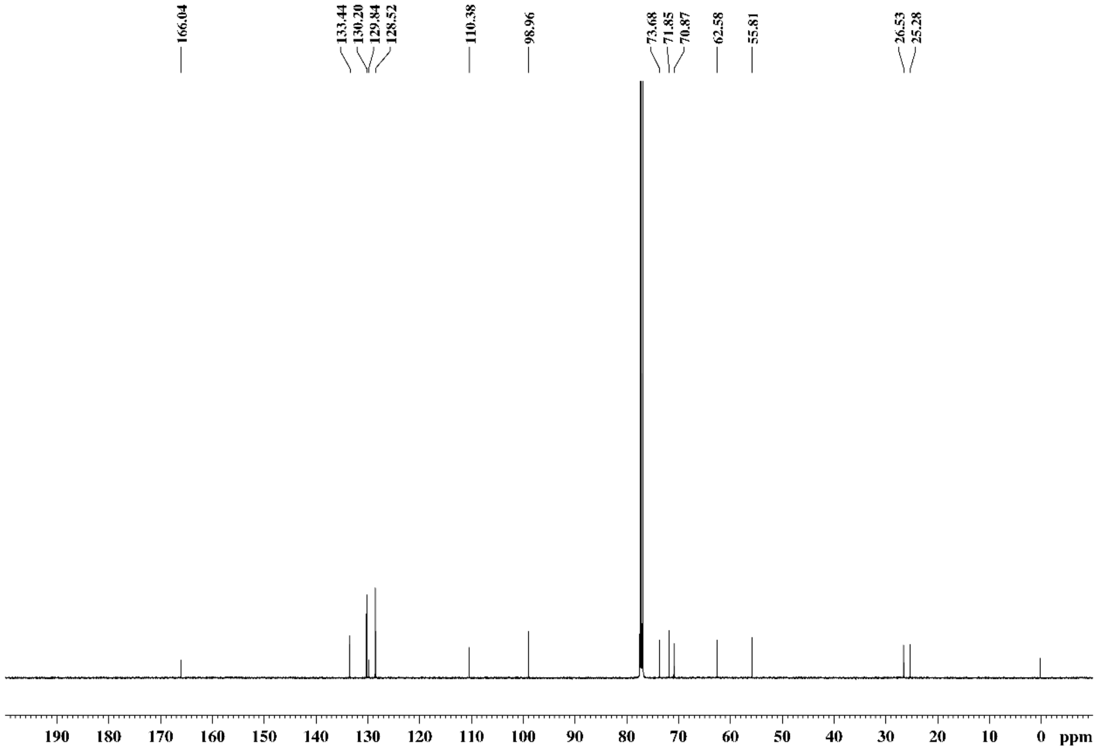

Methyl 2-*O*-benzoyl-β-D-ribopyranoside (37):

^{1}H NMR (500.20 MHz, MeOD, 25°C):

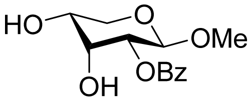

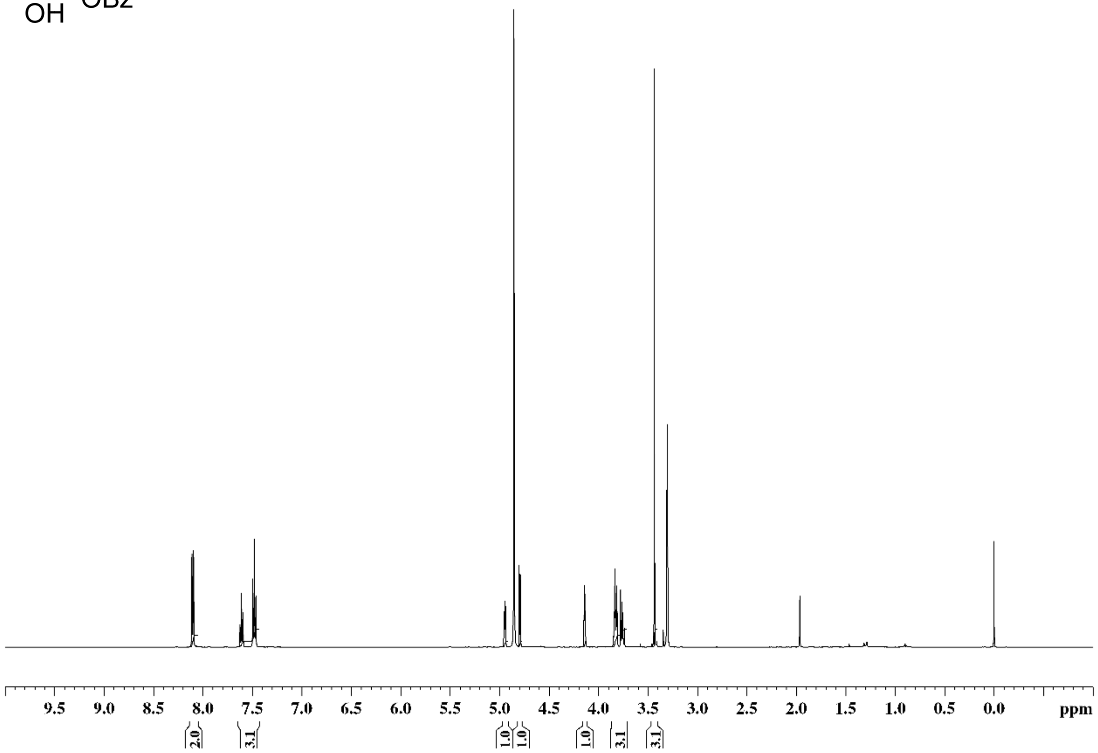

^{13}C NMR (125.8 MHz, MeOD, 25°C):

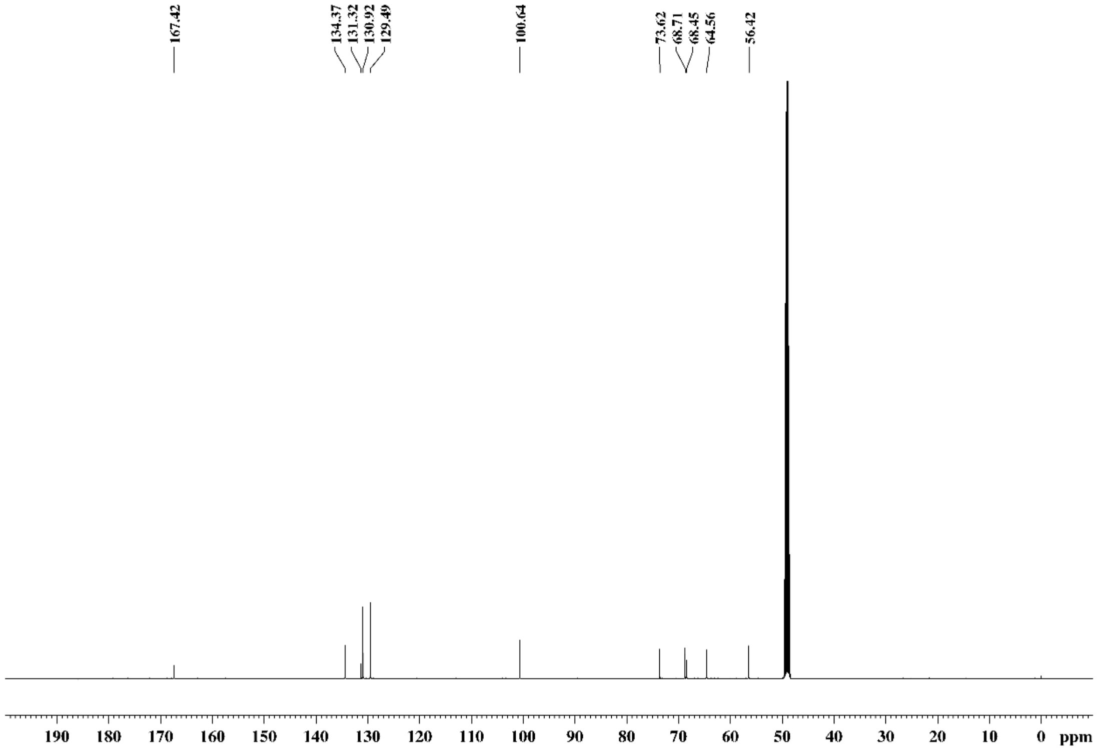

Methyl 3,4-*O*-isopropylidene-2-*O*-pivaoyl-β-D-ribopyranoside (101):

^{1}H NMR (500.20 MHz, $CDCl_3$, 25°C):

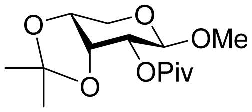

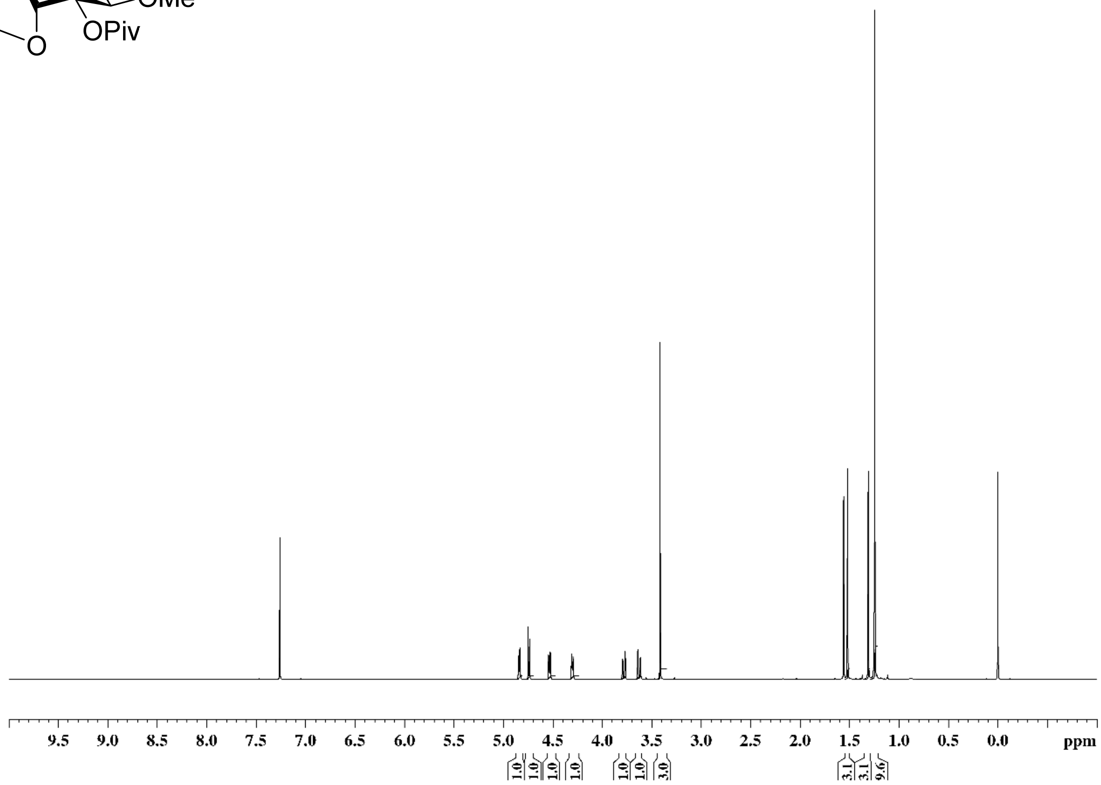

^{13}C NMR (125.8 MHz, $CDCl_3$, 25°C):

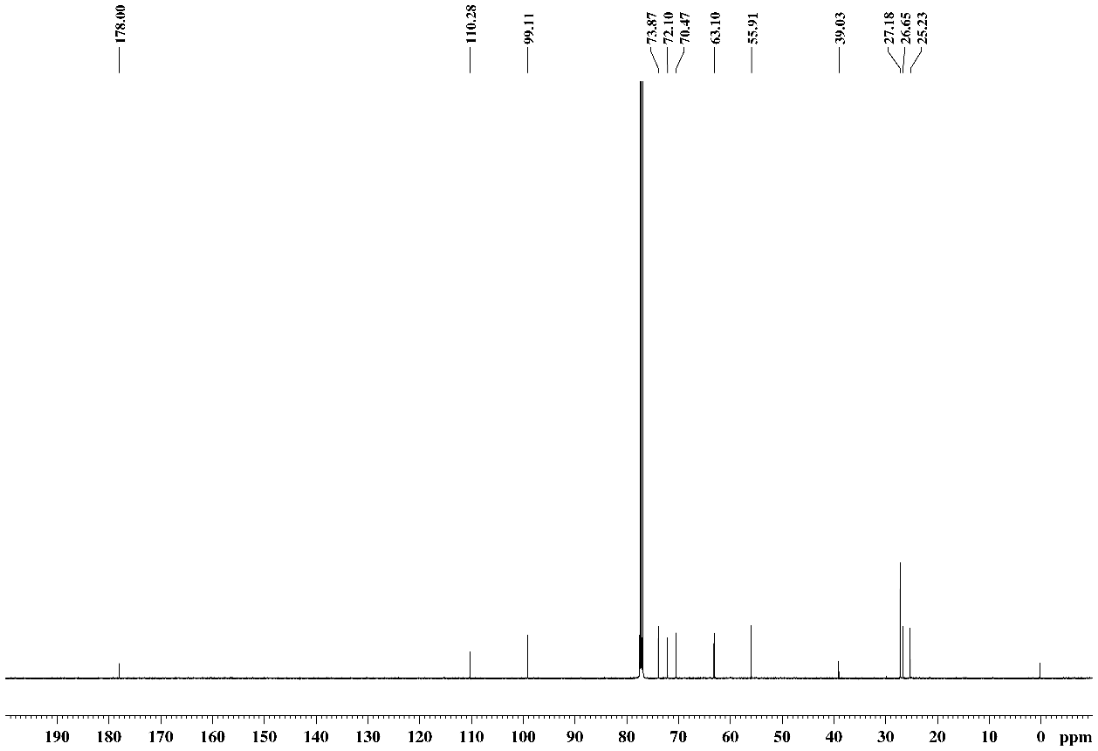

Methyl 2-*O*-pivaoyl-β-D-ribopyranoside (38):

^{1}H NMR (500.20 MHz, MeOD, 25°C):

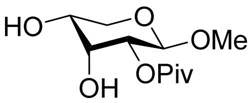

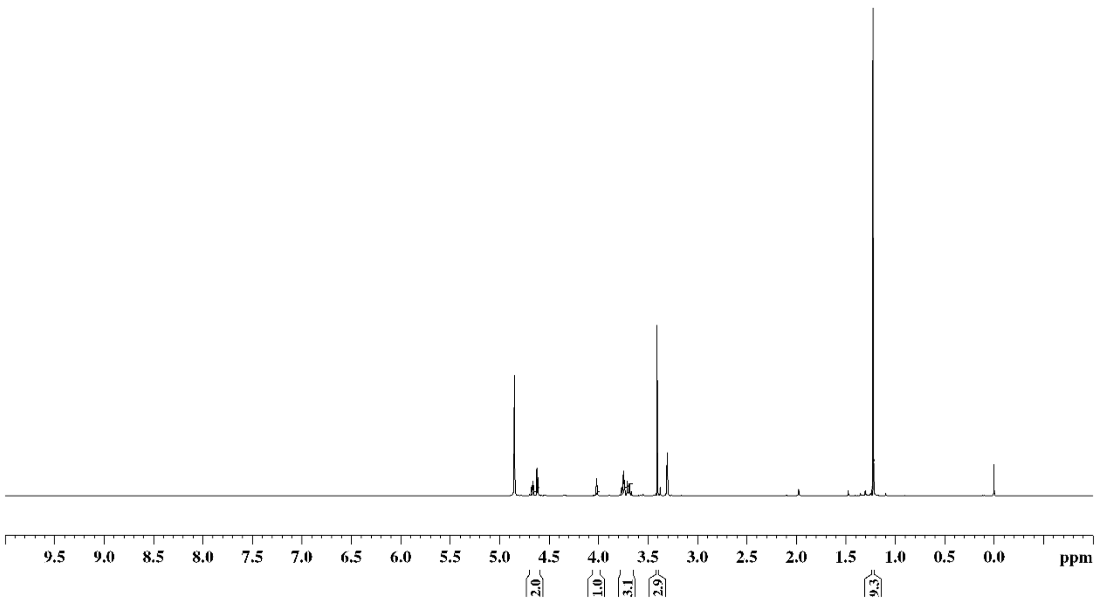

^{13}C NMR (125.8 MHz, MeOD, 25°C):

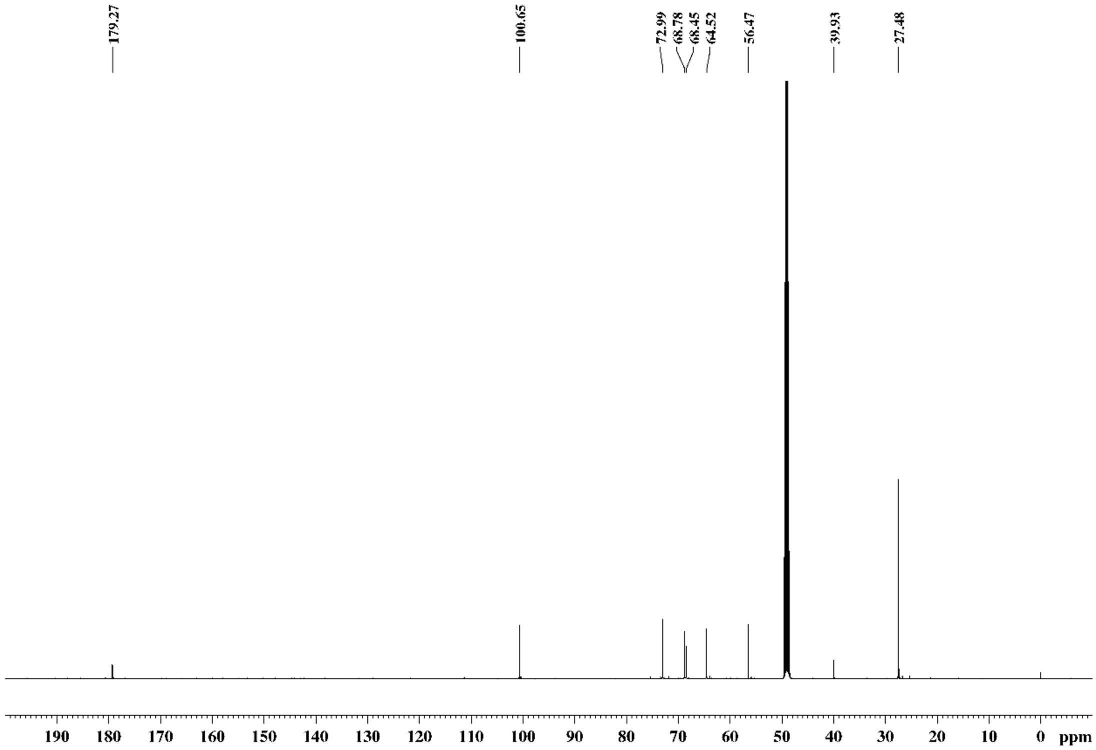

Methyl 3,4-*O*-isopropylidene-2-*O*-(*R*)-2-phenyl-propanoyl-β-D-ribopyranoside (102):
^{1}H NMR (500.20 MHz, $CDCl_3$, 25°C):

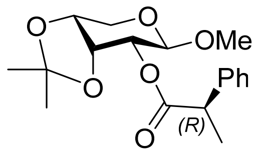

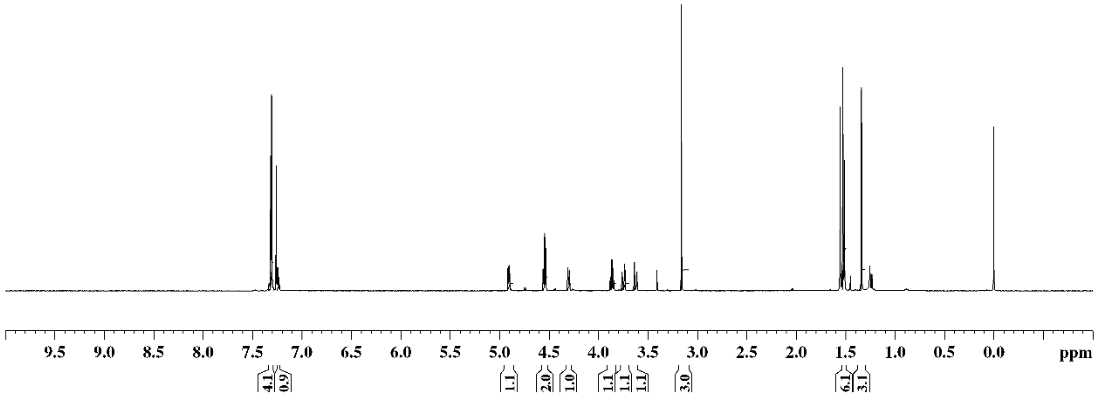

^{13}C NMR (125.8 MHz, $CDCl_3$, 25°C):

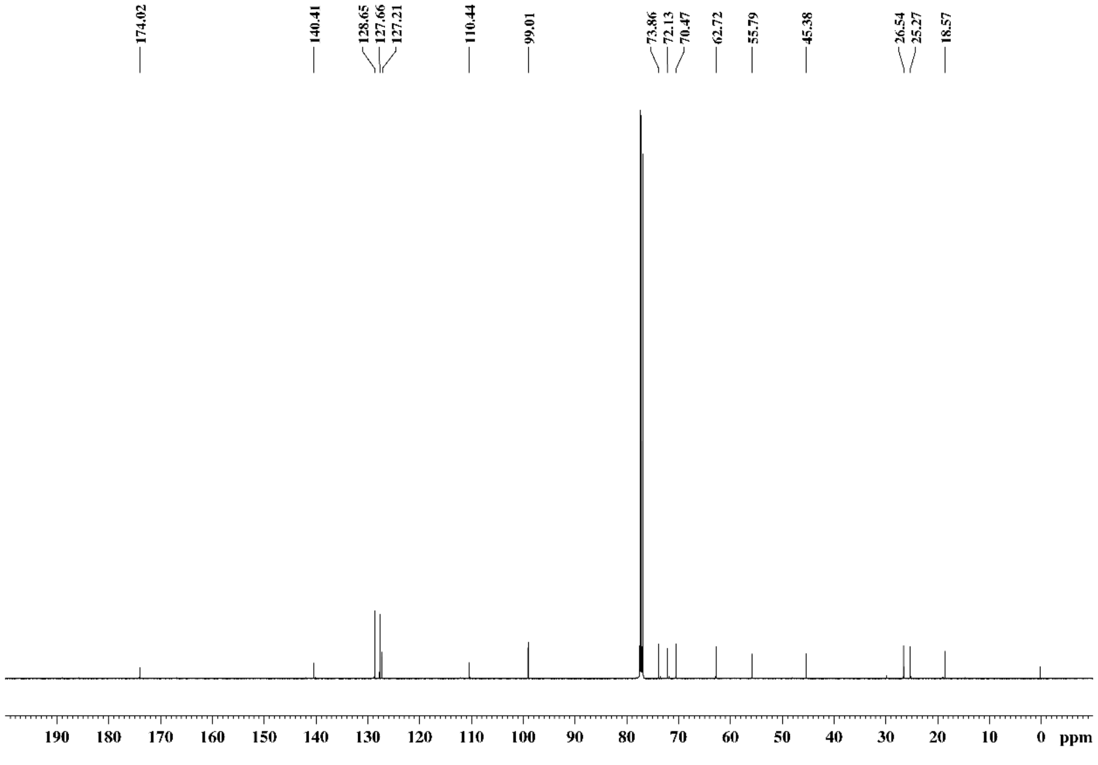

Methyl 2-*O*-(*R*)-2-phenyl-propanoyl-β-D-ribopyranoside (39):

^{1}H NMR (500.20 MHz, MeOD, 25°C):

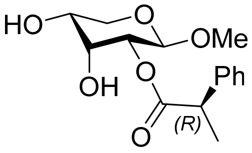

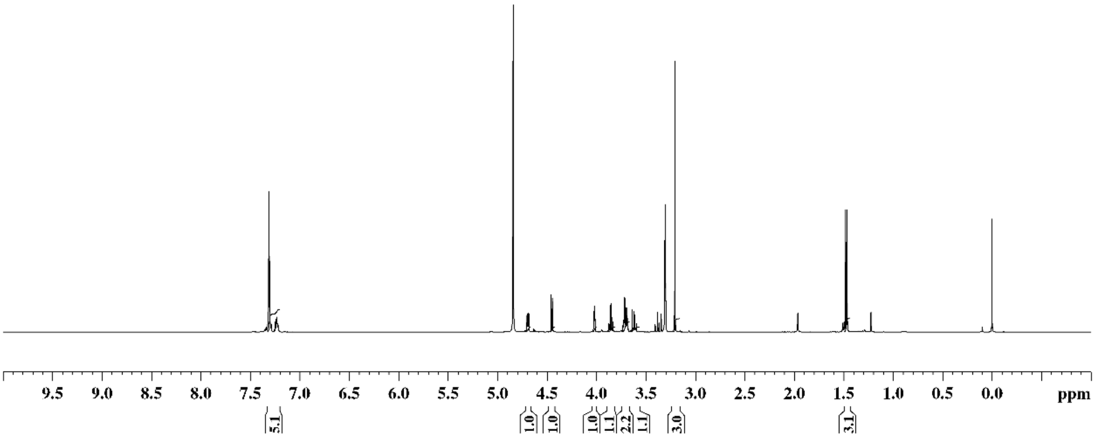

^{13}C NMR (125.8 MHz, MeOD, 25°C):

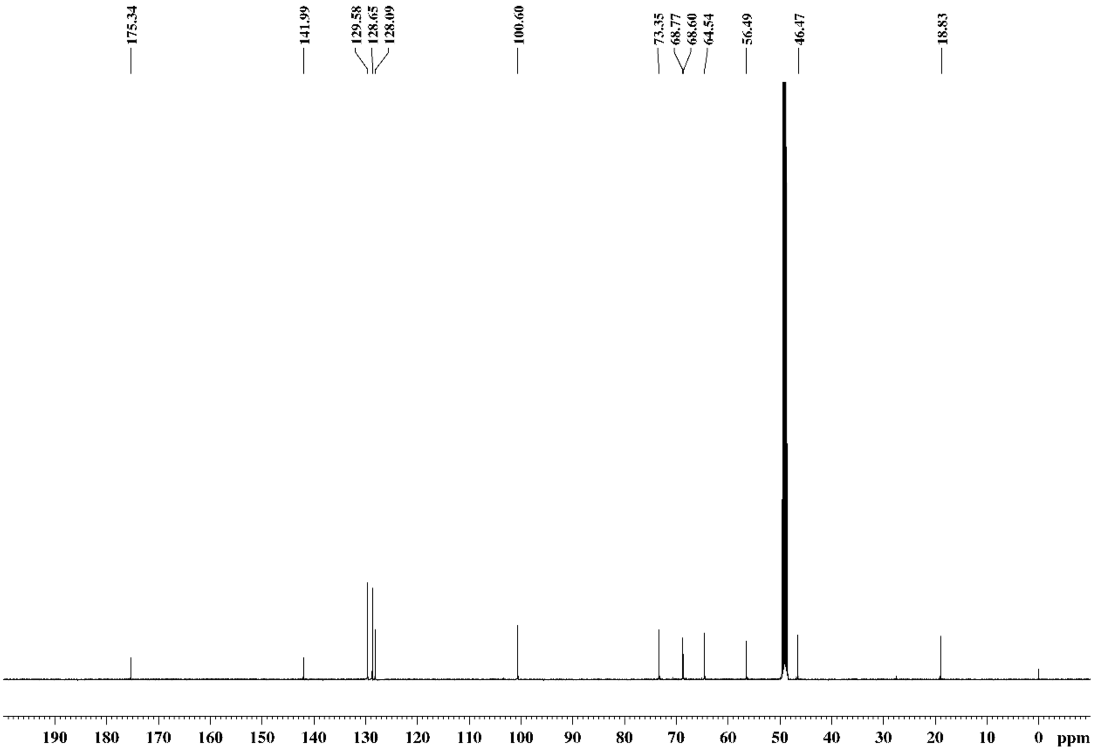

Methyl 3,4-*O*-isopropylidene-2-*O*-(*S*)-2-phenyl-propanoyl-β-D-ribopyranoside (103):

^{1}H NMR (500.20 MHz, CDCl$_3$, 25°C):

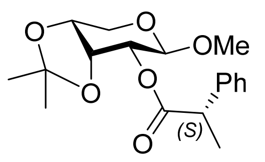

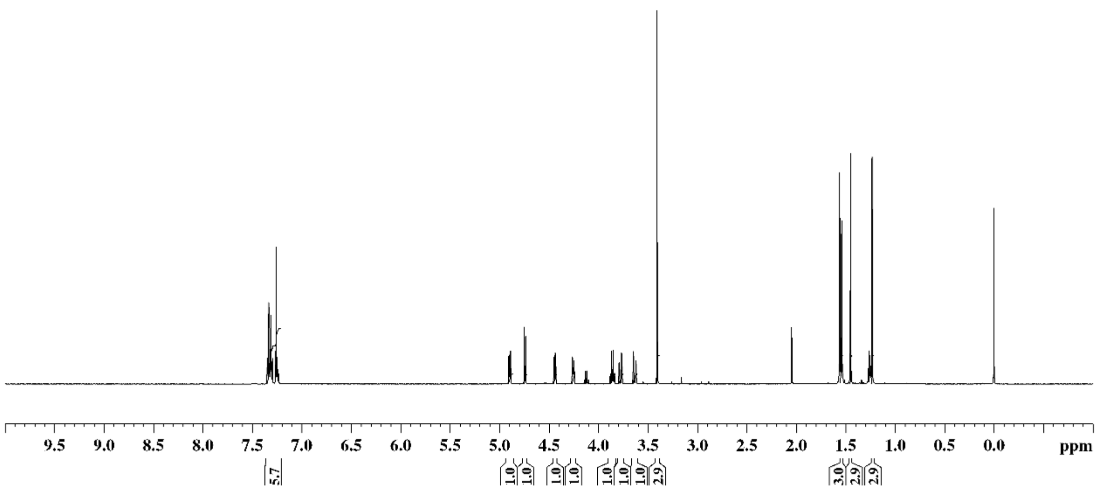

^{13}C NMR (125.8 MHz, CDCl$_3$, 25°C):

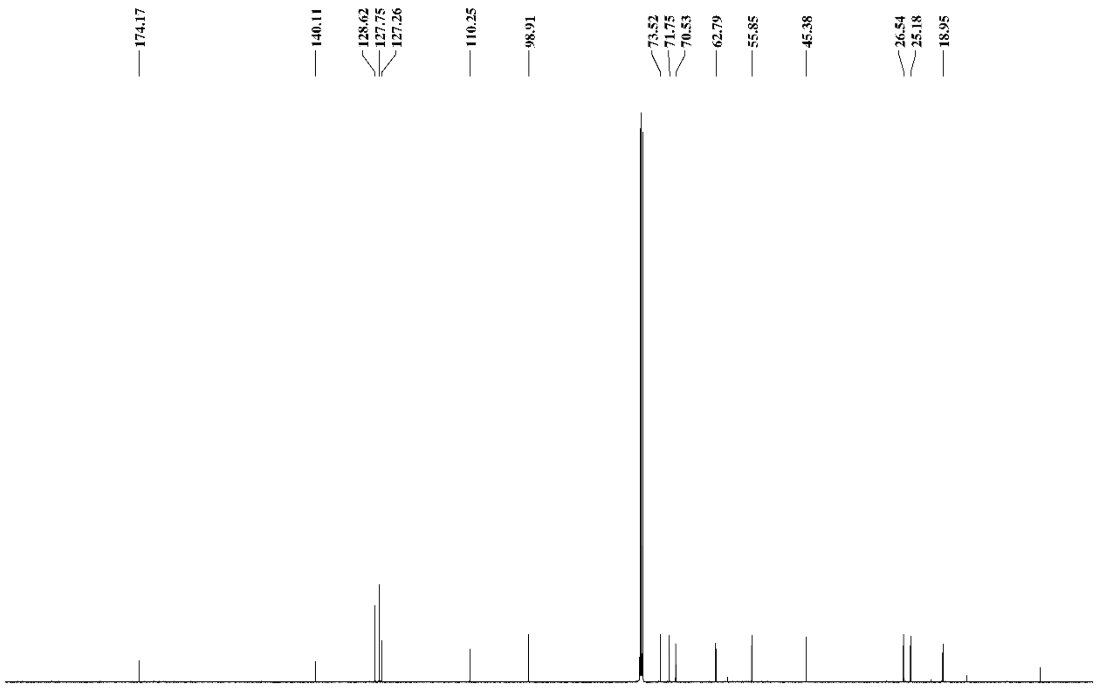

Methyl 2-*O*-(*S*)-2-phenyl-propanoyl-β-D-ribopyranoside (40):

^{1}H NMR (500.20 MHz, MeOD, 25°C):

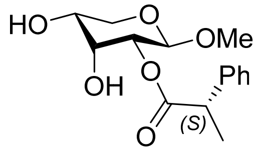

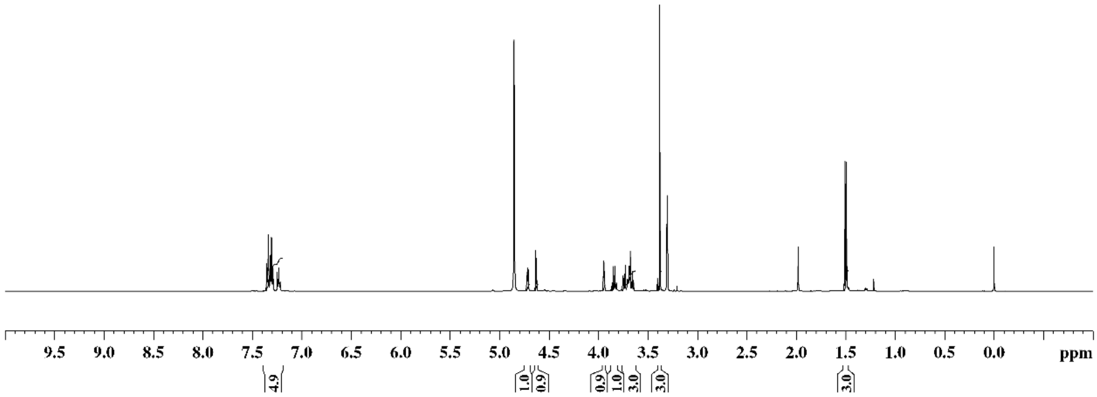

^{13}C NMR (125.8 MHz, MeOD, 25°C):

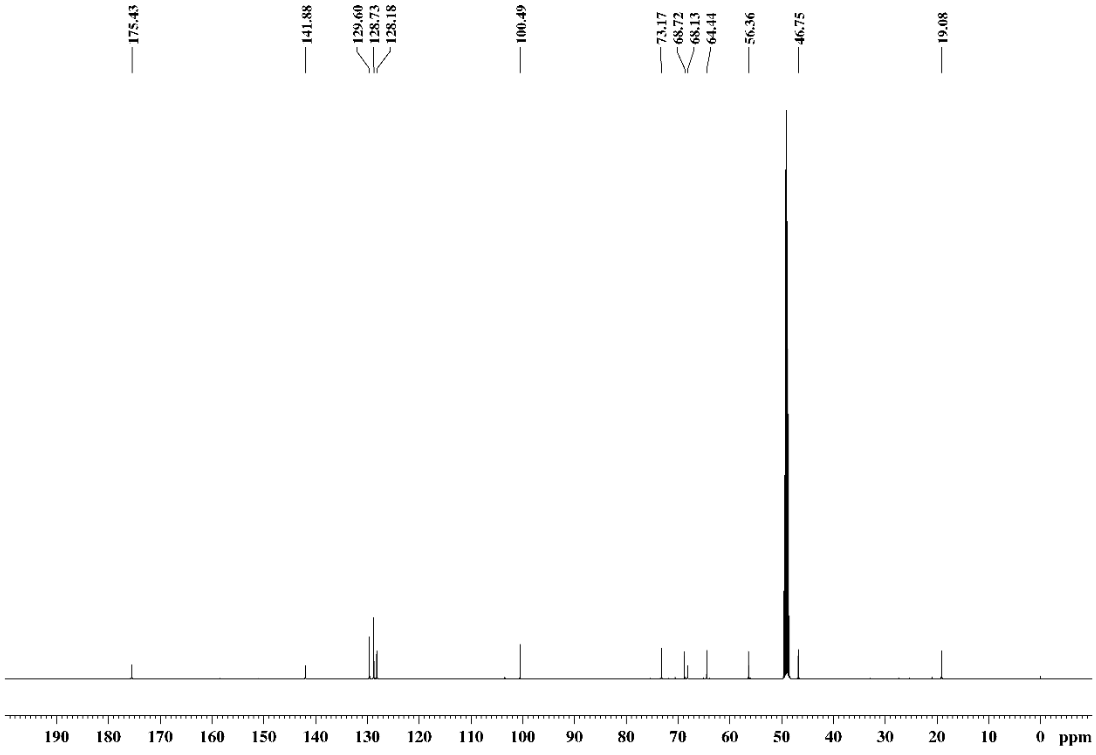

Methyl 2-*O*-acetyl-3,4-*O*-isopropylidene-β-D-arabinopyranoside (105):

^{1}H NMR (500.20 MHz, $CDCl_3$, 25°C):

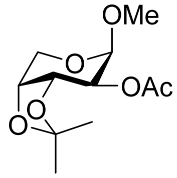

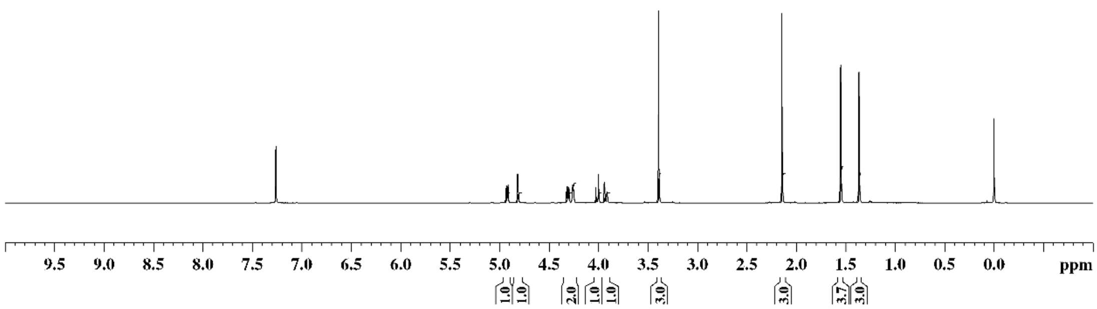

^{13}C NMR (125.8 MHz, $CDCl_3$, 25°C):

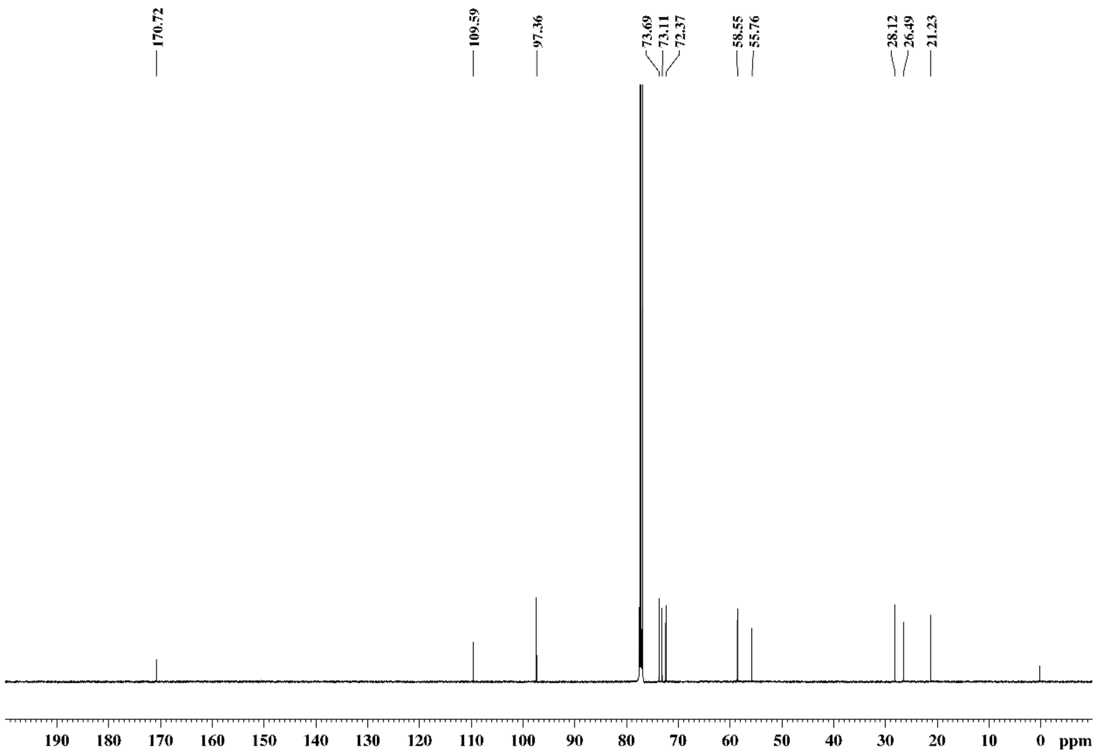

Methyl 2-*O*-acetyl-β-D-arabinopyranoside (41):

^{1}H NMR (500.20 MHz, MeOD, 25°C):

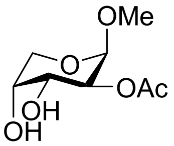

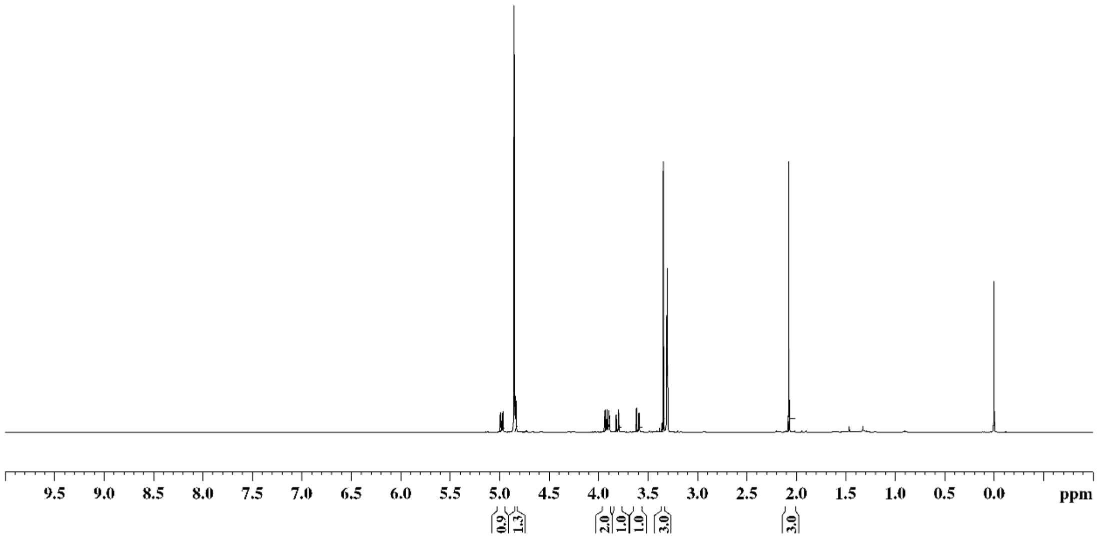

^{13}C NMR (125.8 MHz, MeOD, 25°C):

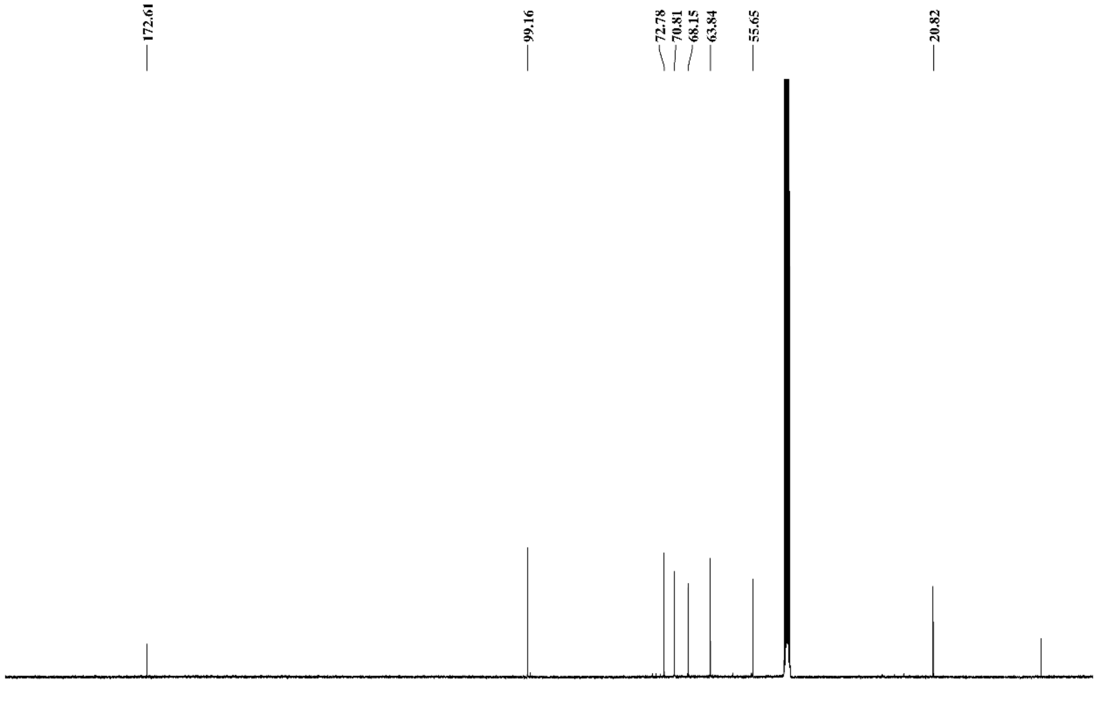

Methyl 2-*O*-benzoyl-3,4-*O*-isopropylidene-β-D-arabinopyranoside (106):

^{1}H NMR (500.20 MHz, CDCl$_3$, 25°C):

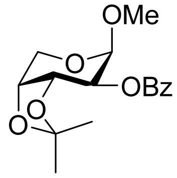

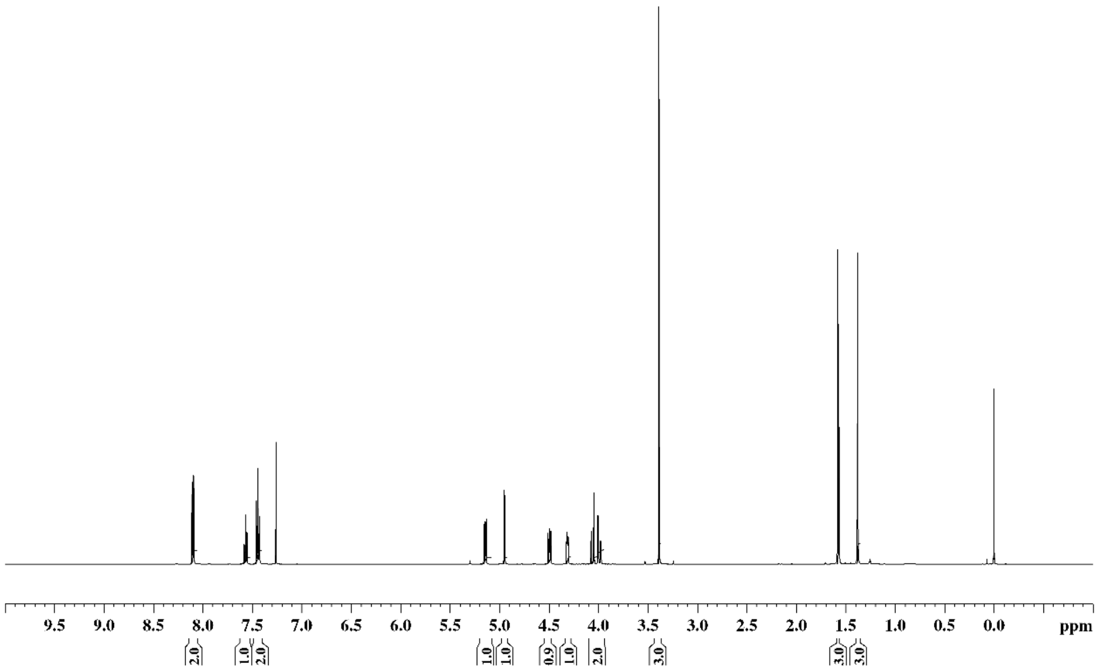

^{13}C NMR (125.8 MHz, CDCl$_3$, 25°C):

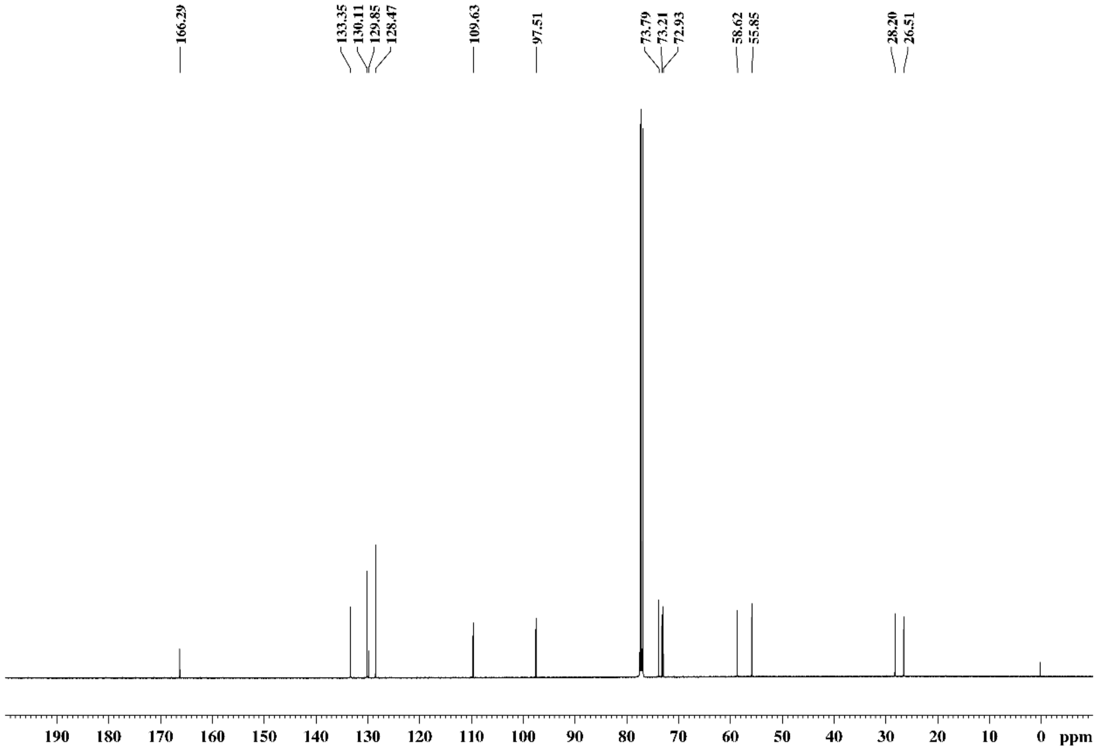

Methyl 2-*O*-benzoyl-β-D-arabinopyranoside (42):

^{1}H NMR (500.20 MHz, MeOD, 25°C):

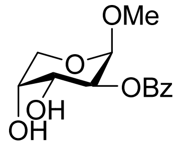

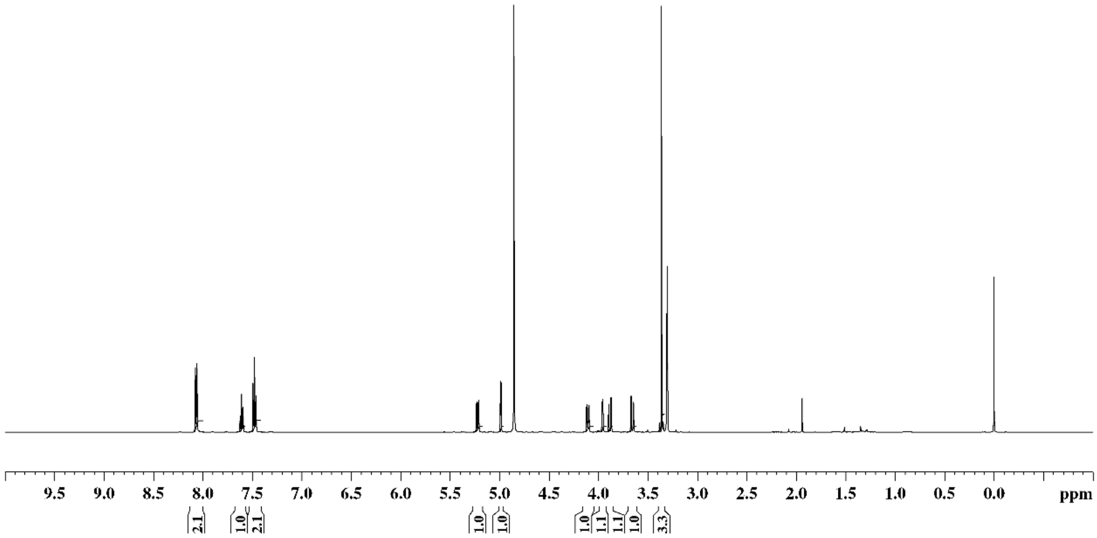

^{13}C NMR (125.8 MHz, MeOD, 25°C):

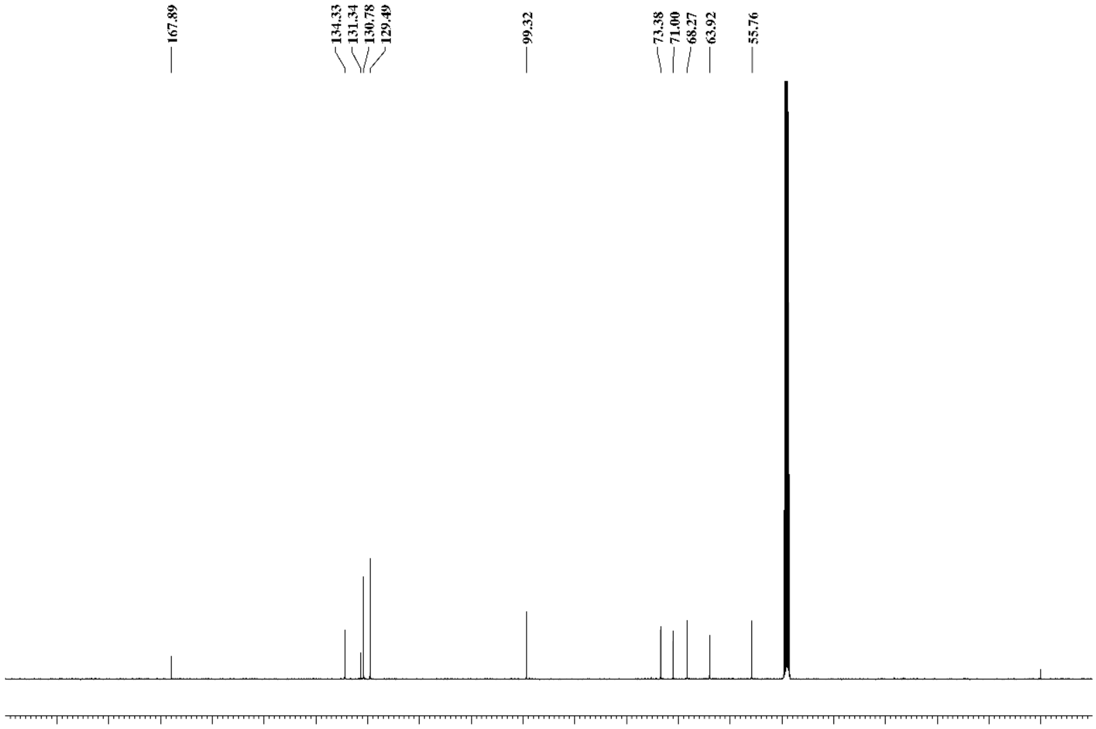

Methyl 3,4-*O*-isopropylidene-2-*O*-pivaoyl-β-D-arabinopyranoside (107):

^{1}H NMR (500.20 MHz, $CDCl_3$, 25°C):

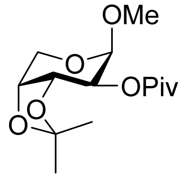

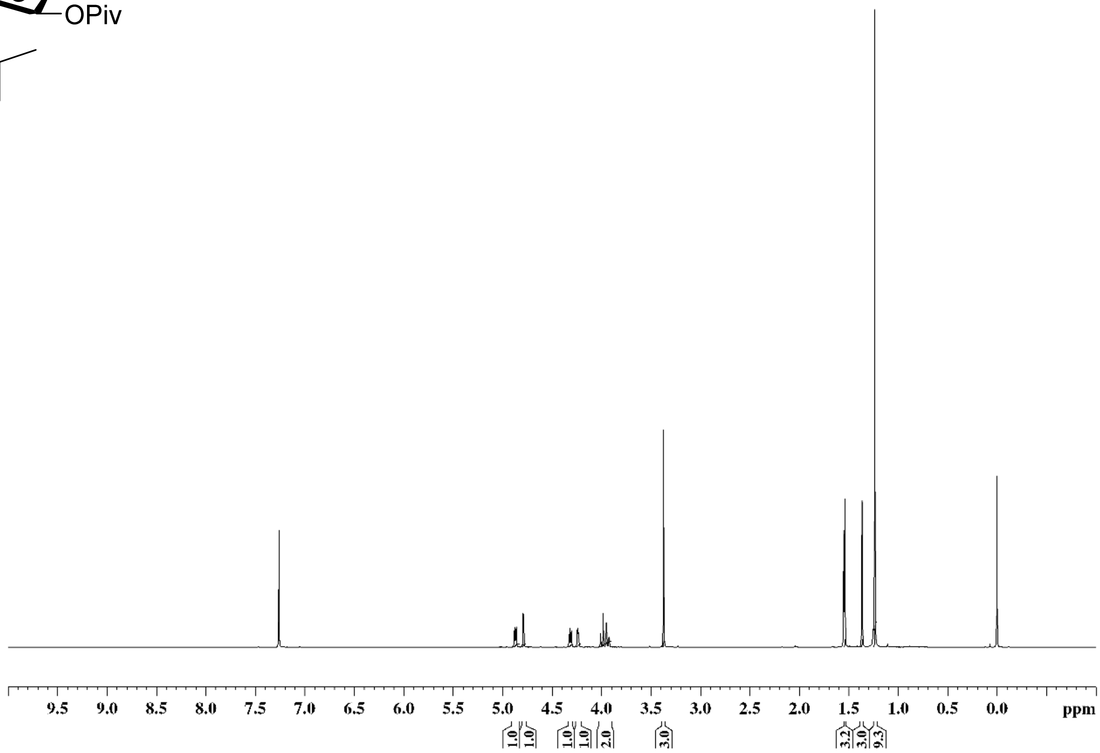

^{13}C NMR (125.8 MHz, $CDCl_3$, 25°C):

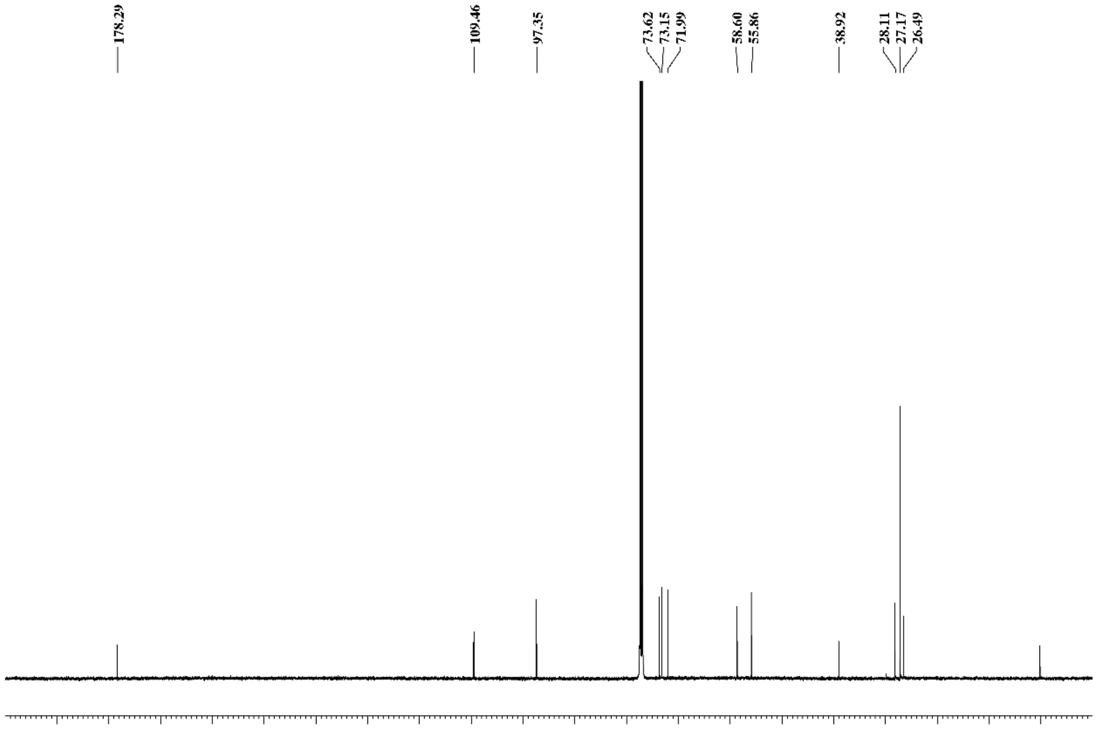

Methyl 2-*O*-pivaoyl-β-D-arabinopyranoside (43):

^{1}H NMR (500.20 MHz, MeOD, 25°C):

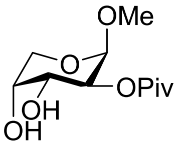

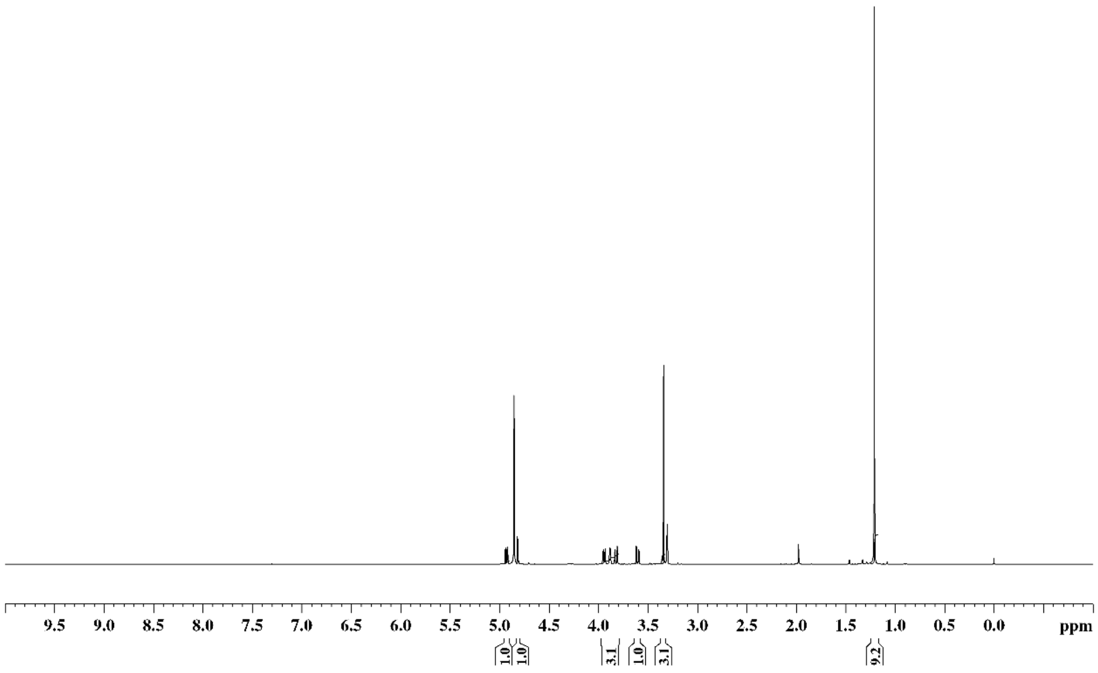

^{13}C NMR (125.8 MHz, MeOD, 25°C):

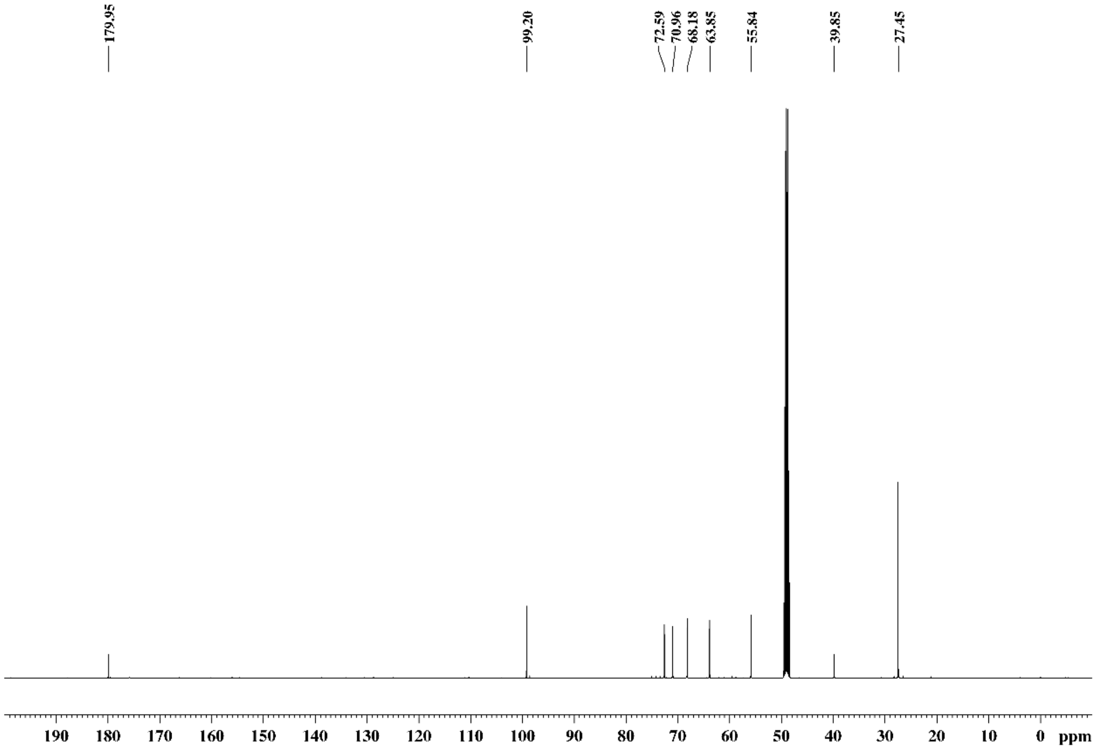

Methyl 3,4-*O*-isopropylidene-2-*O*-(*R*)-2-phenyl-propanoyl-β-D-arabinopyranoside (108):

^{1}H NMR (500.20 MHz, $CDCl_3$, 25°C):

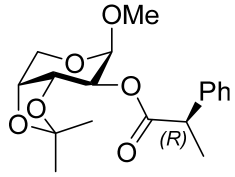

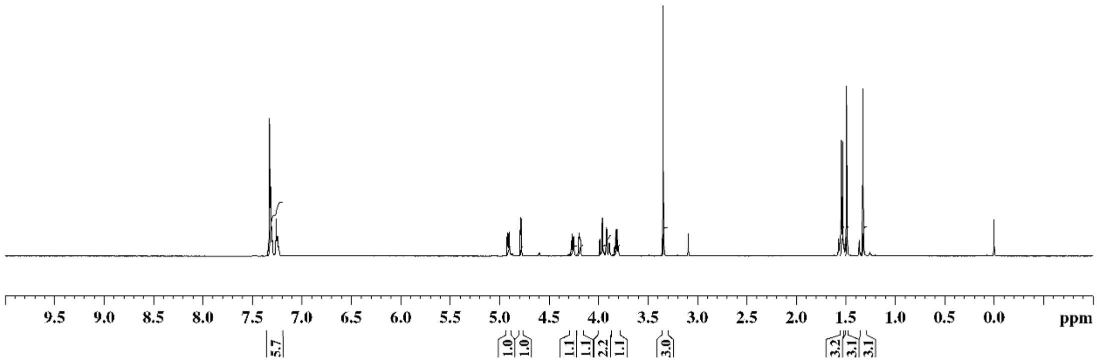

^{13}C NMR (125.8 MHz, $CDCl_3$, 25°C):

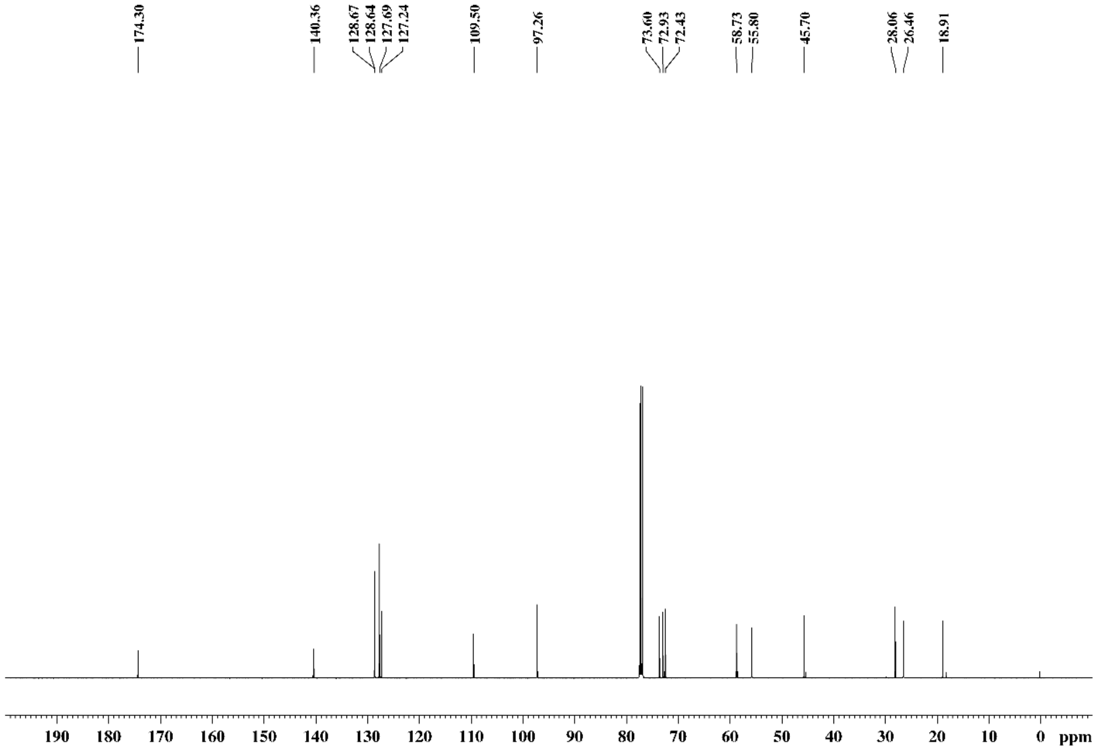

Methyl 2-*O*-(*R*)-2-phenyl-propanoyl-β-D-arabinopyranoside (44):

^{1}H NMR (500.20 MHz, MeOD, 25°C):

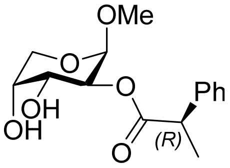

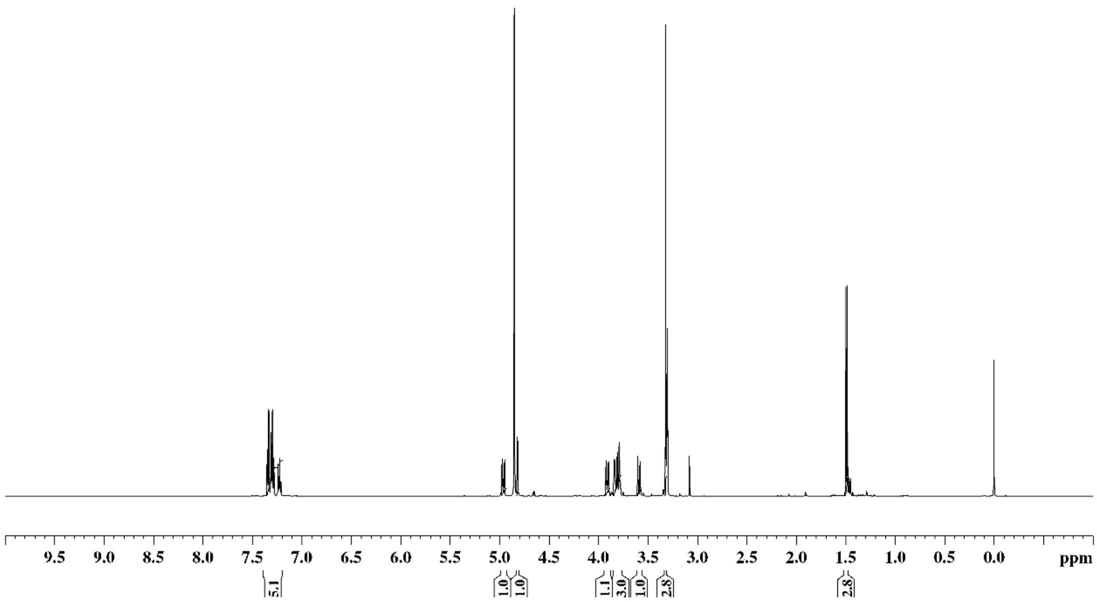

^{13}C NMR (125.8 MHz, MeOD, 25°C):

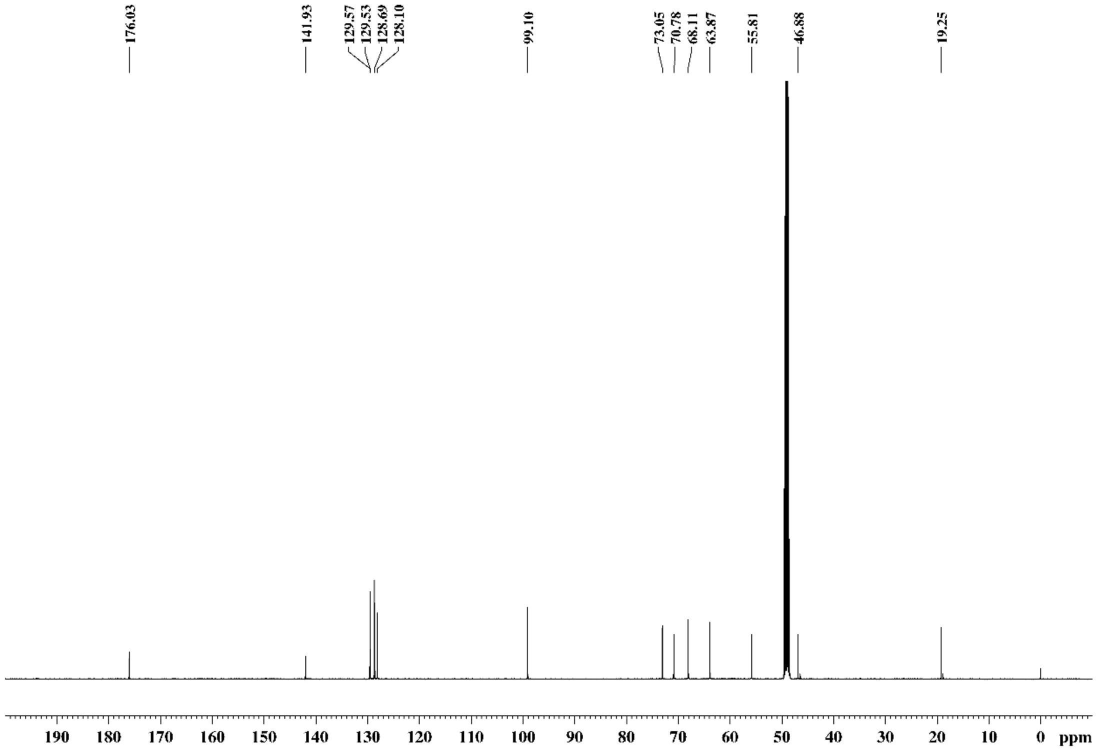

Methyl 3,4-*O*-isopropylidene-2-*O*-(*S*)-2-phenyl-propanoyl-β-D-arabinopyranoside (109):

^{1}H NMR (500.20 MHz, $CDCl_3$, 25°C):

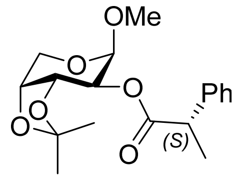

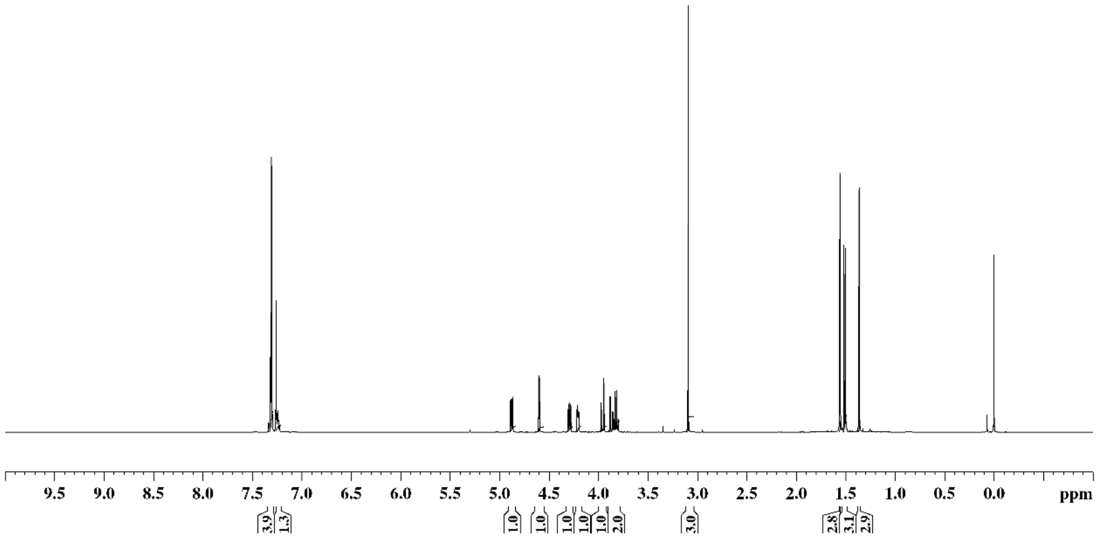

^{13}C NMR (125.8 MHz, $CDCl_3$, 25°C):

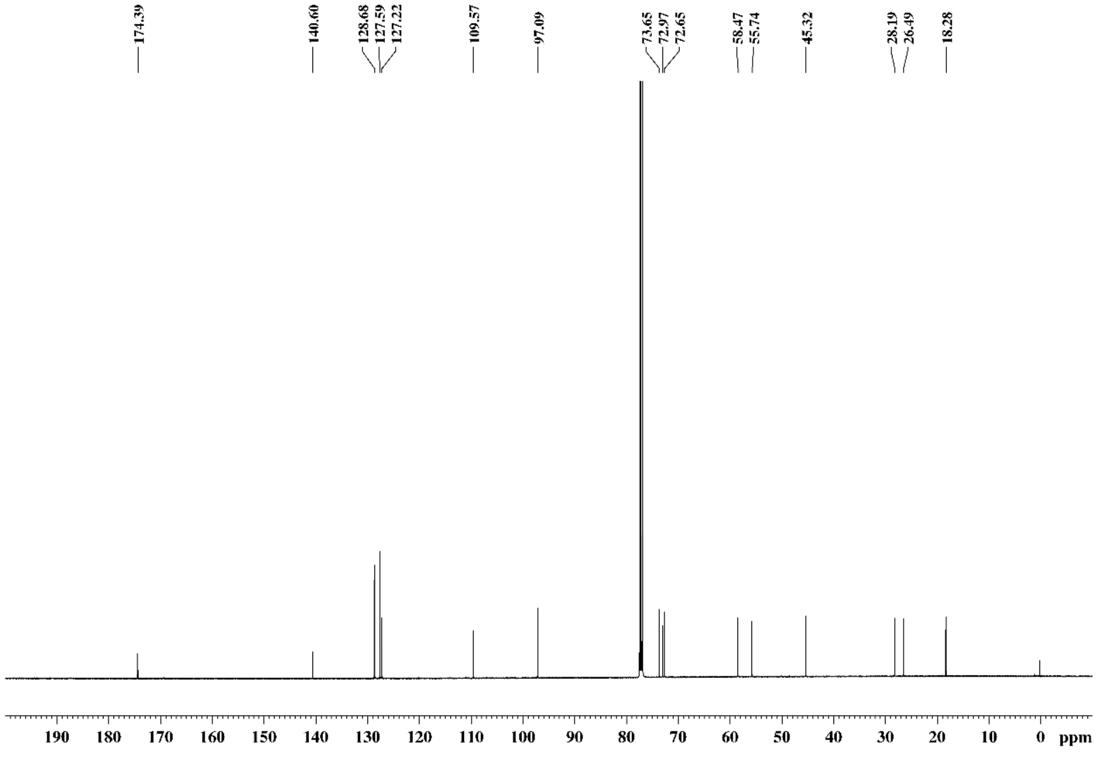

Methyl 2-*O*-(*S*)-2-phenyl-propanoyl-β-D-arabinopyranoside (45):

^{1}H NMR (500.20 MHz, MeOD, 25°C):

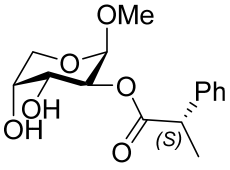

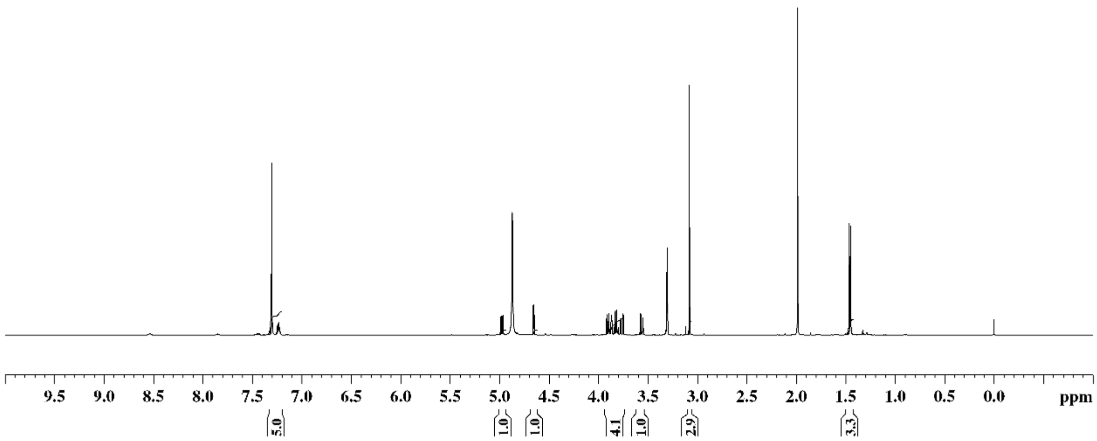

^{13}C NMR (125.8 MHz, MeOD, 25°C):

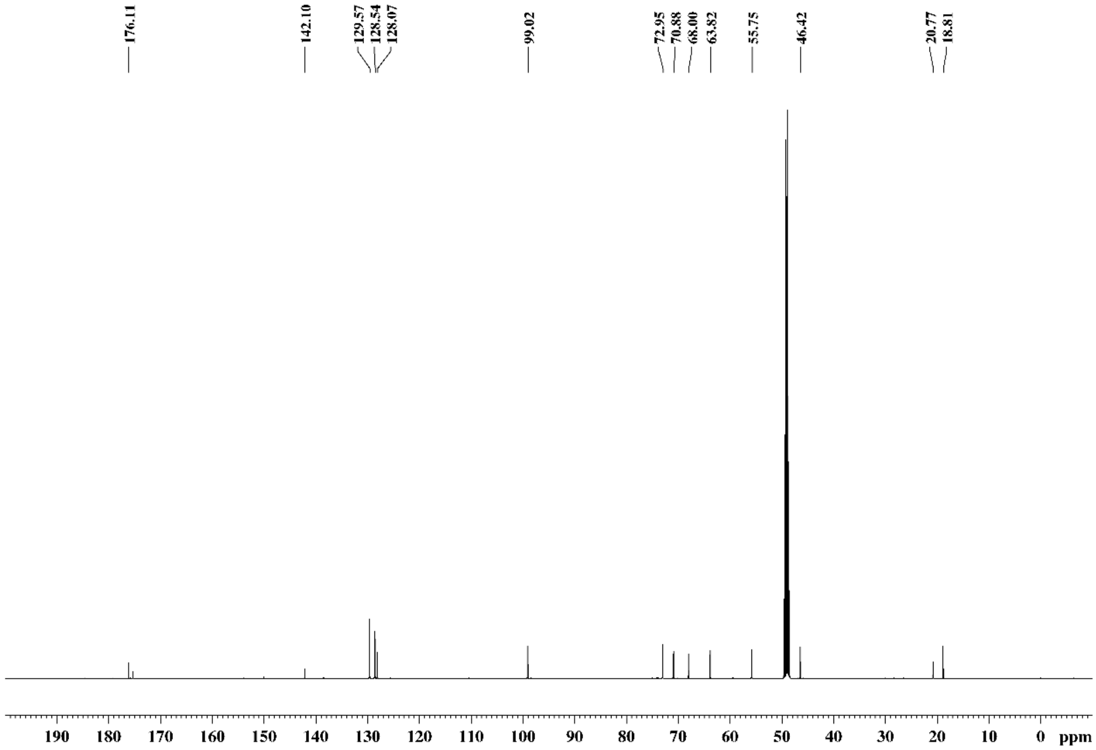

Methyl 4-*O*-acetyl-2,3-*O*-isopropylidene-α-L-rhamnopyranoside (111):

^{1}H NMR (500.20 MHz, $CDCl_3$, 25°C):

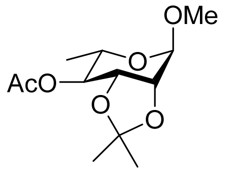

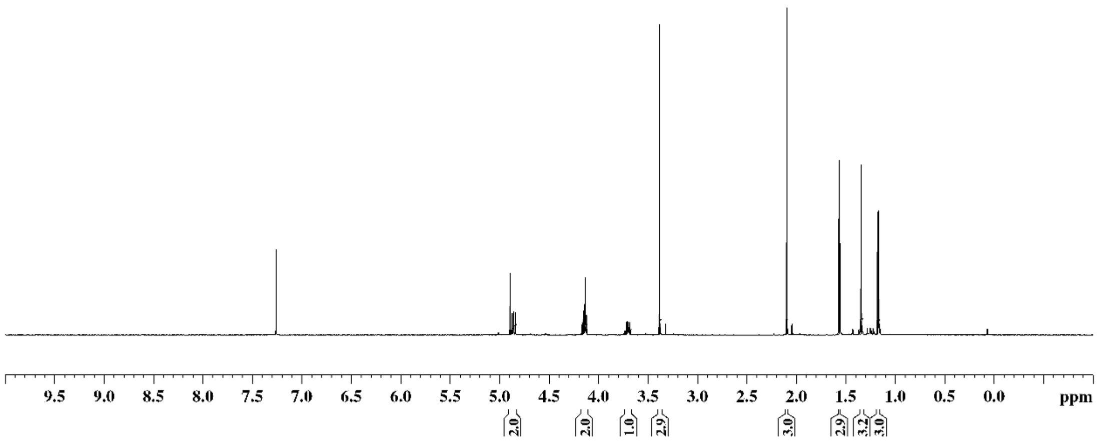

^{13}C NMR (125.8 MHz, $CDCl_3$, 25°C):

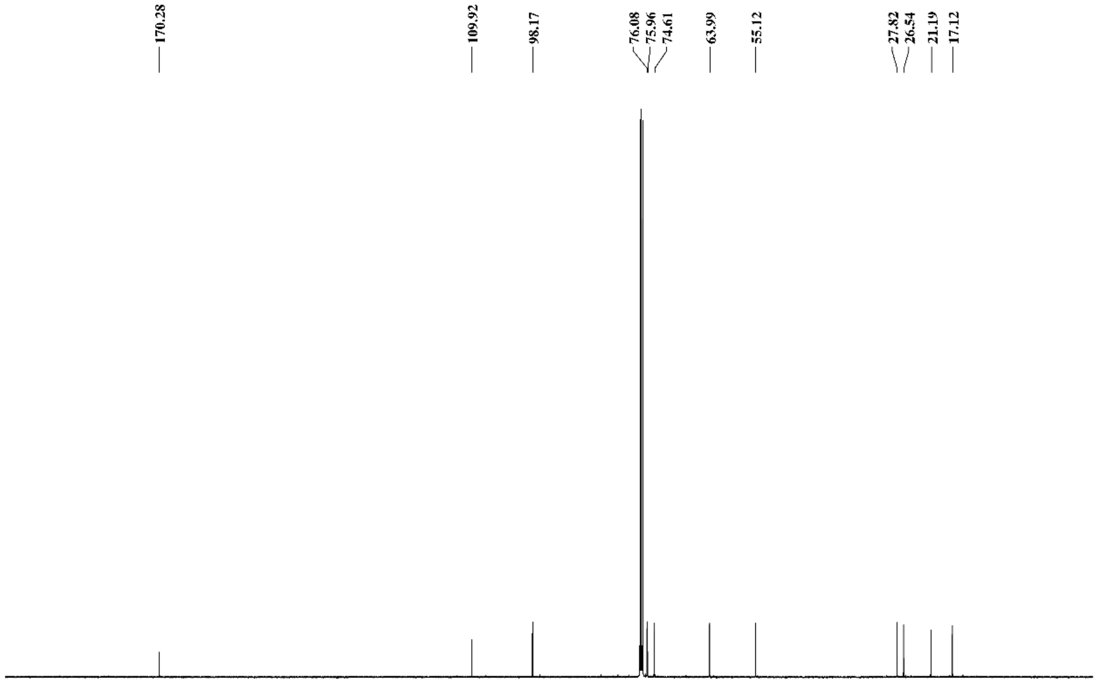

Methyl 4-*O*-acetyl-α-L-rhamnopyranoside (46):

^{1}H NMR (500.20 MHz, MeOD, 25°C):

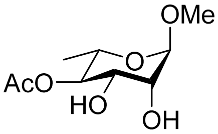

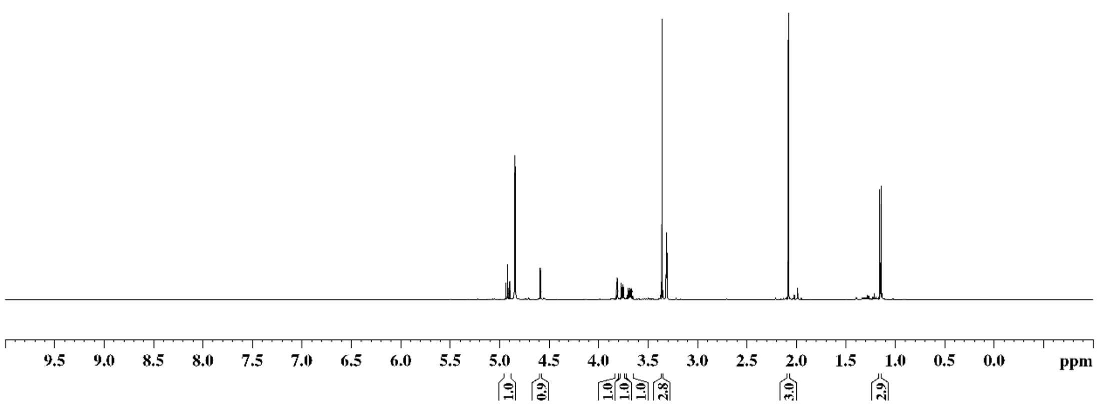

^{13}C NMR (125.8 MHz, MeOD, 25°C):

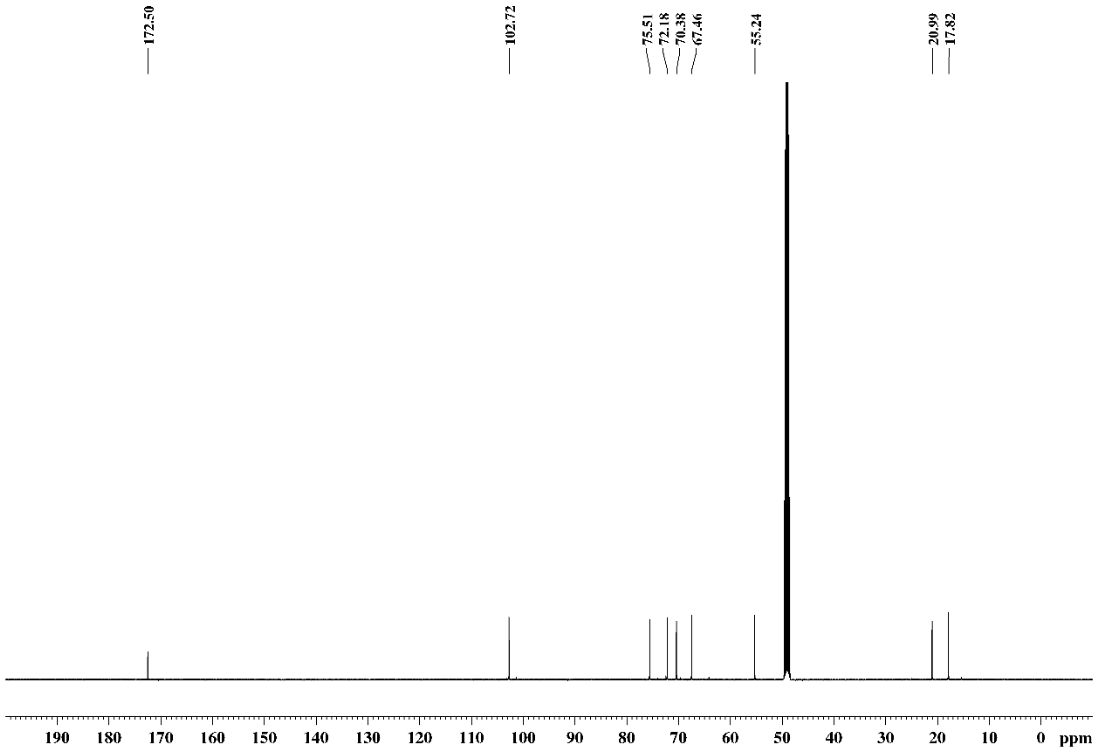

Methyl 4-*O*-benzoyl-2,3-*O*-isopropylidene-α-L-rhamnopyranoside (112):

^{1}H NMR (500.20 MHz, $CDCl_3$, 25°C):

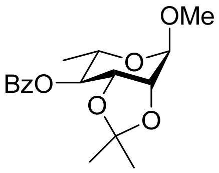

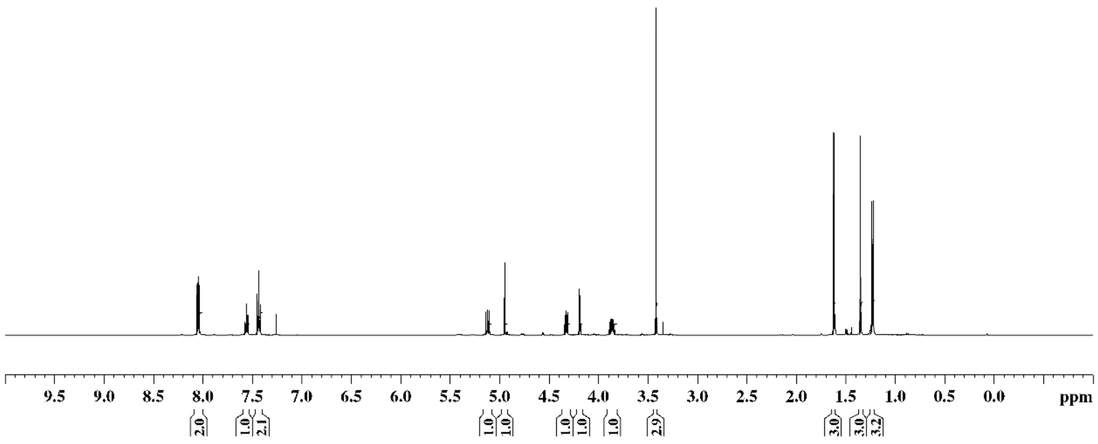

^{13}C NMR (125.8 MHz, $CDCl_3$, 25°C):

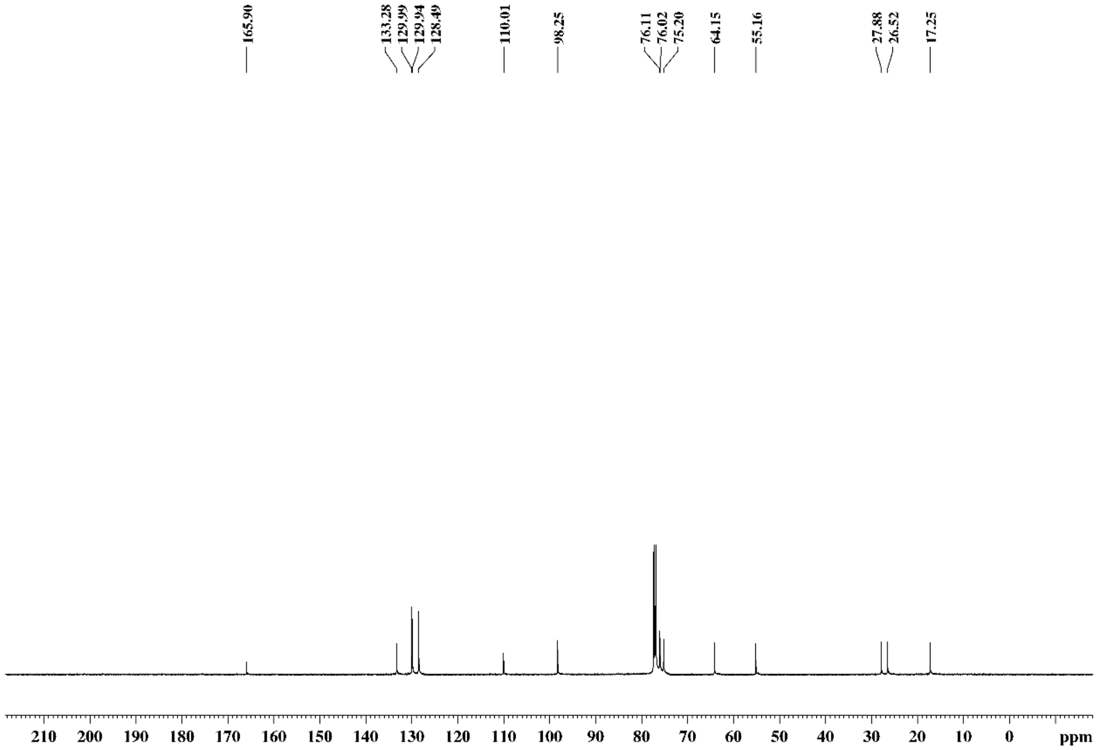

Methyl 4-*O*-benzoyl-α-L-rhamnopyranoside (47):

^{1}H NMR (500.20 MHz, MeOD, 25°C):

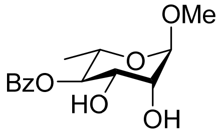

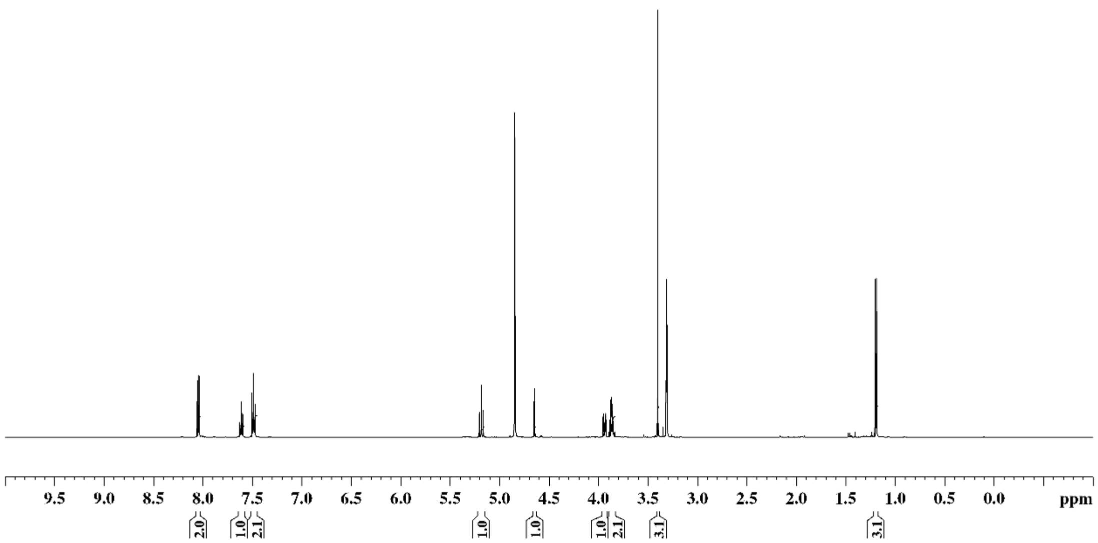

^{13}C NMR (125.8 MHz, MeOD, 25°C):

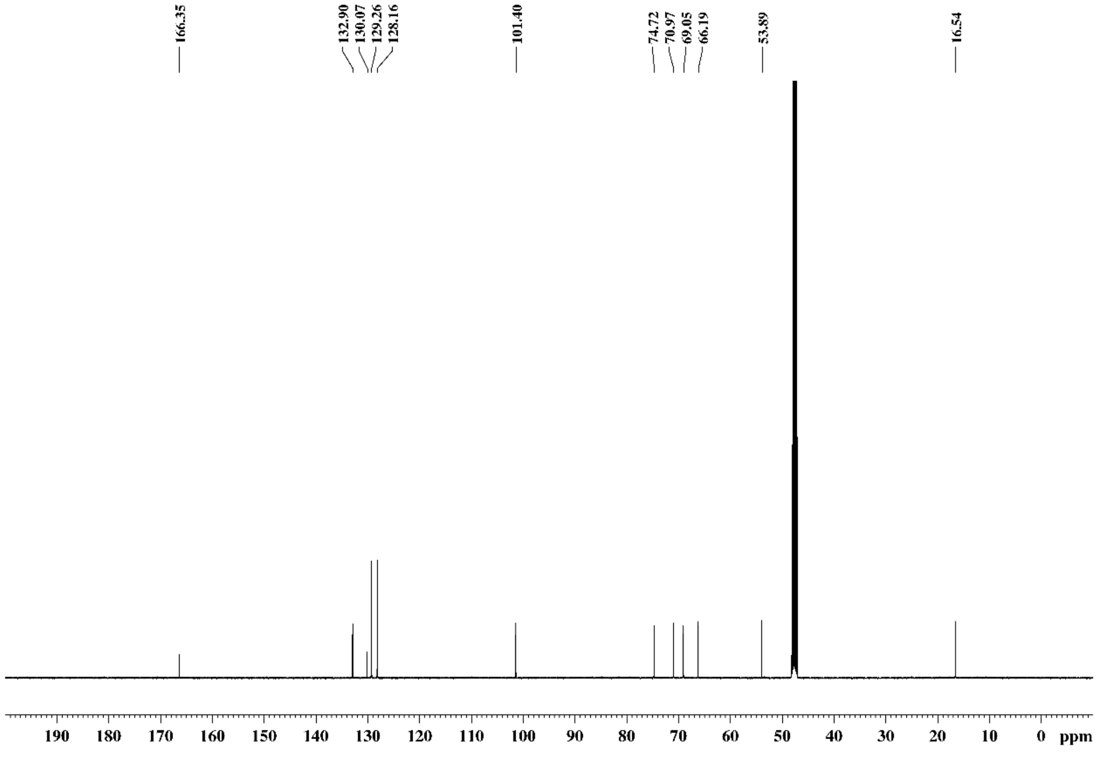

Methyl 2,3-*O*-isopropylidene-4-*O*-pivaoyl-α-L-rhamnopyranoside (113):

^{1}H NMR (500.20 MHz, $CDCl_3$, 25°C):

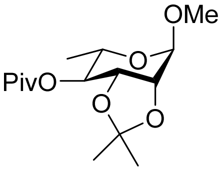

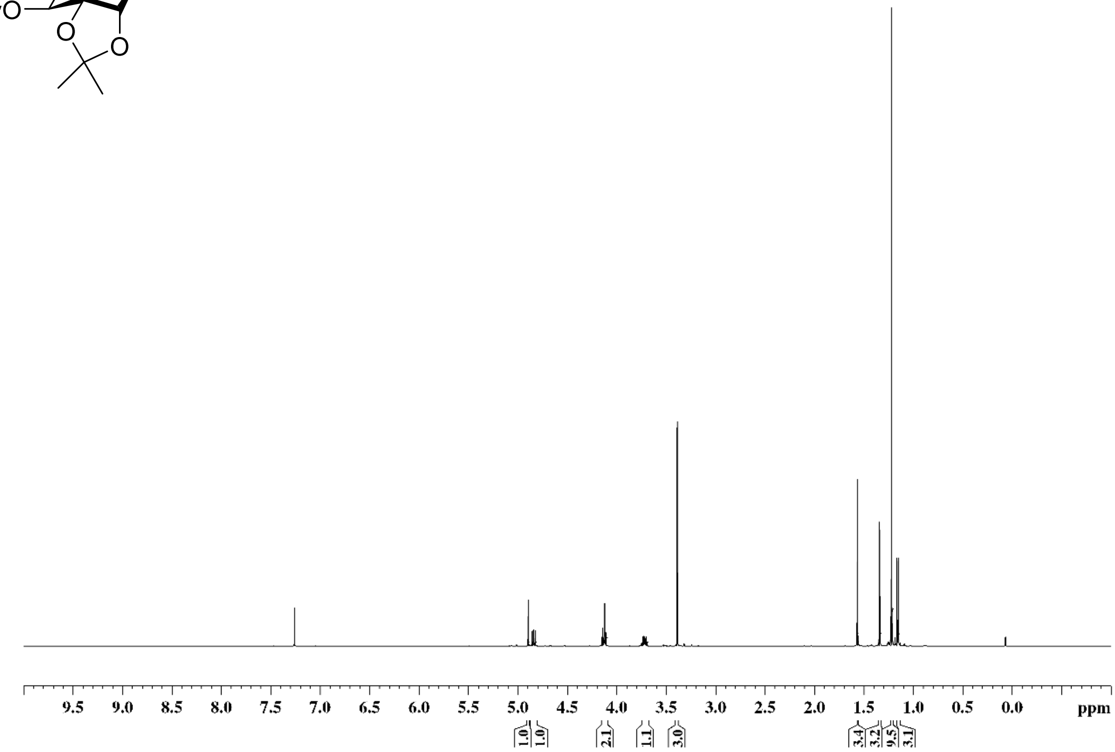

^{13}C NMR (125.8 MHz, $CDCl_3$, 25°C):

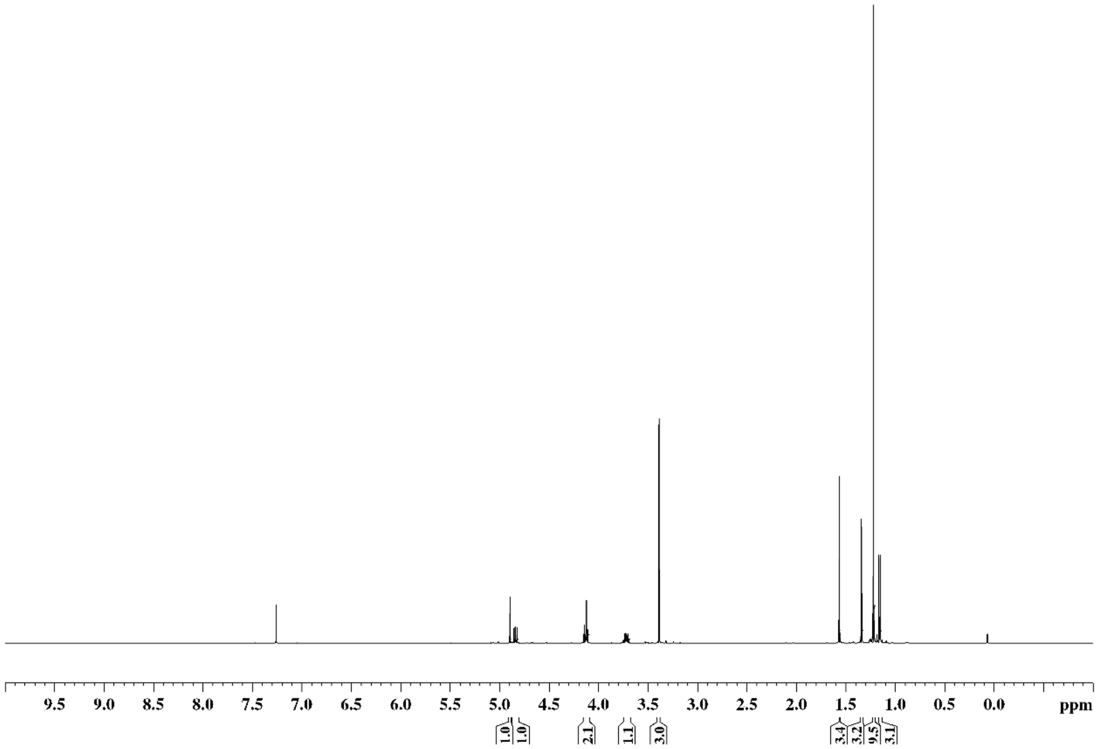

Methyl 4-*O*-pivaoyl-α-L-rhamnopyranoside (48):

^{1}H NMR (500.20 MHz, MeOD, 25°C):

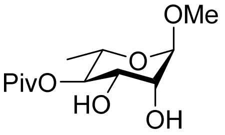

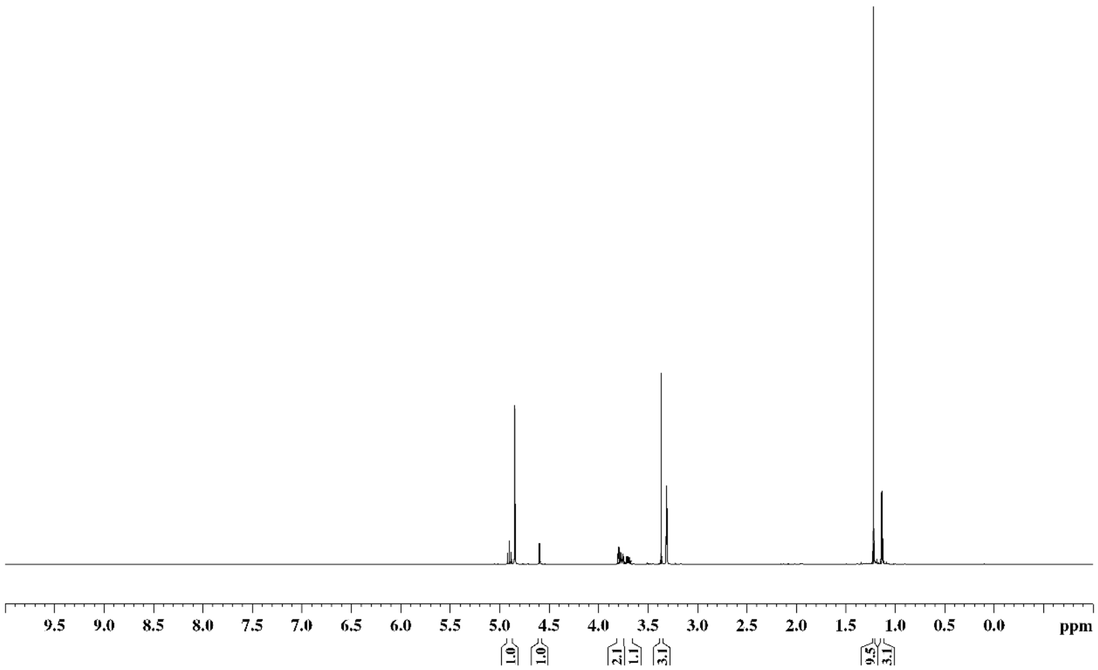

^{13}C NMR (125.8 MHz, MeOD, 25°C):

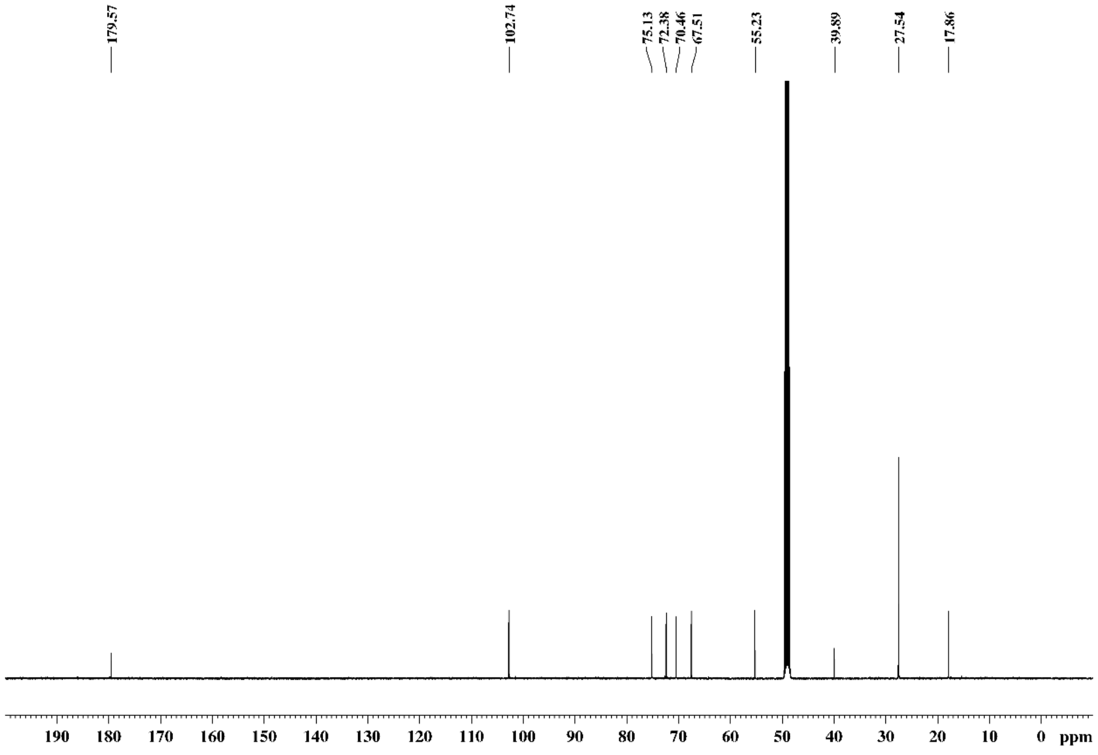

Methyl 2,3-*O*-isopropylidene-4-*O*-(*R*)-2-phenyl-propanoyl-α-L-rhamnopyranoside (114):

^{1}H NMR (500.20 MHz, $CDCl_3$, 25°C):

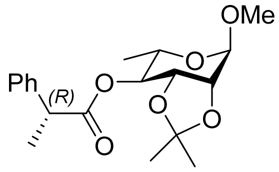

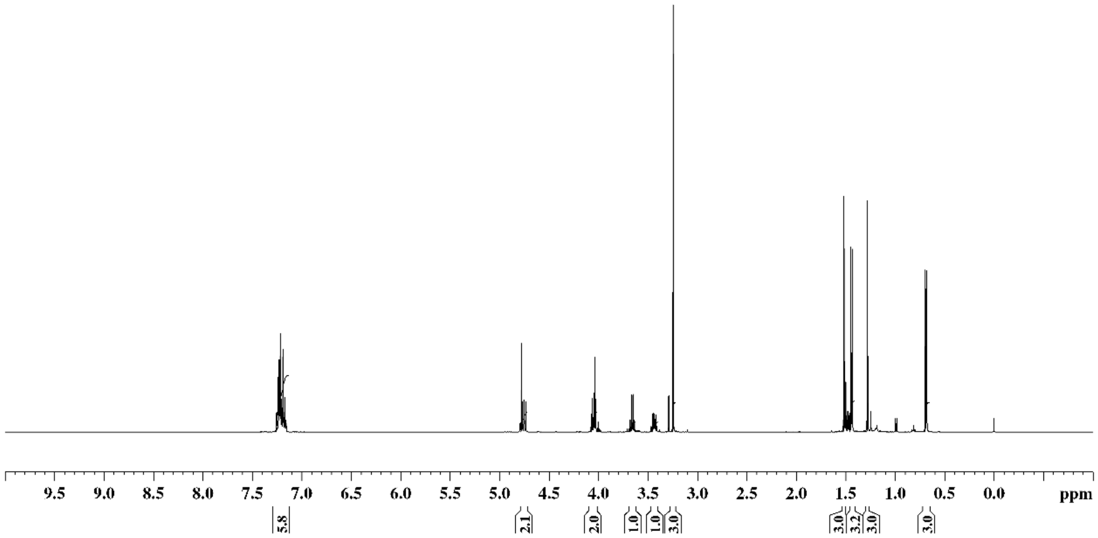

^{13}C NMR (125.8 MHz, $CDCl_3$, 25°C):

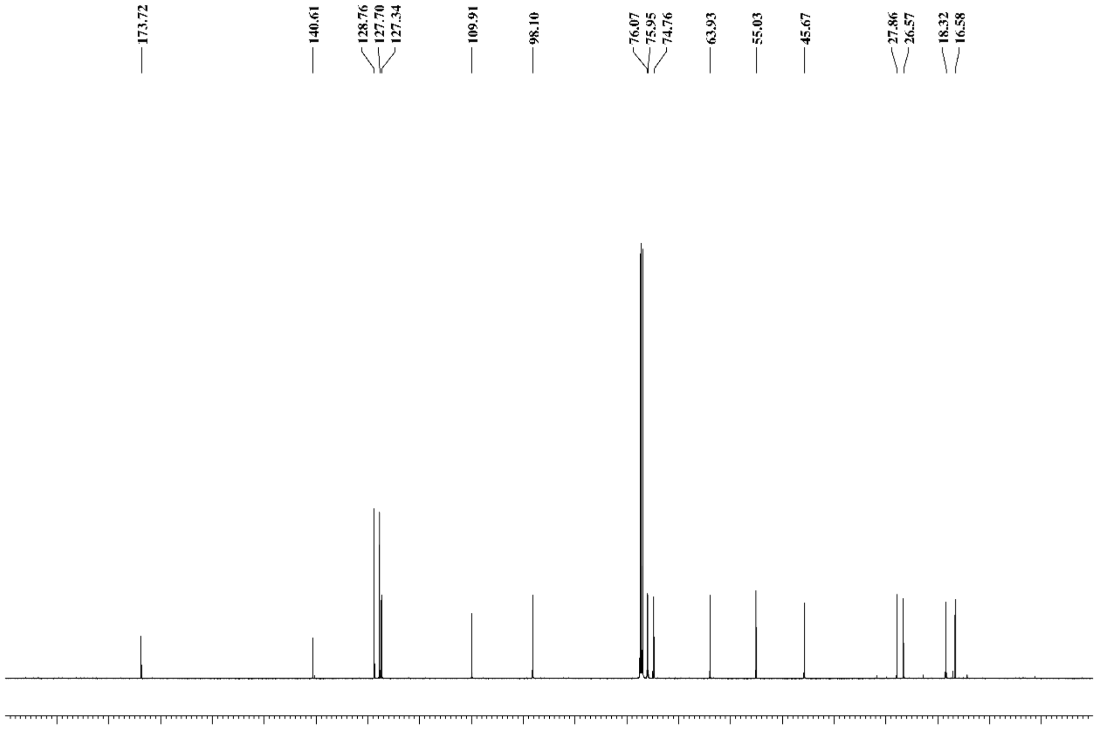

Methyl 4-*O*-(*R*)-2-phenyl-propanoyl-α-L-rhamnopyranoside (49):

^{1}H NMR (500.20 MHz, MeOD, 25°C):

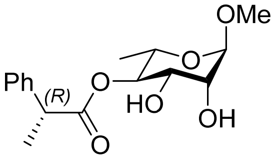

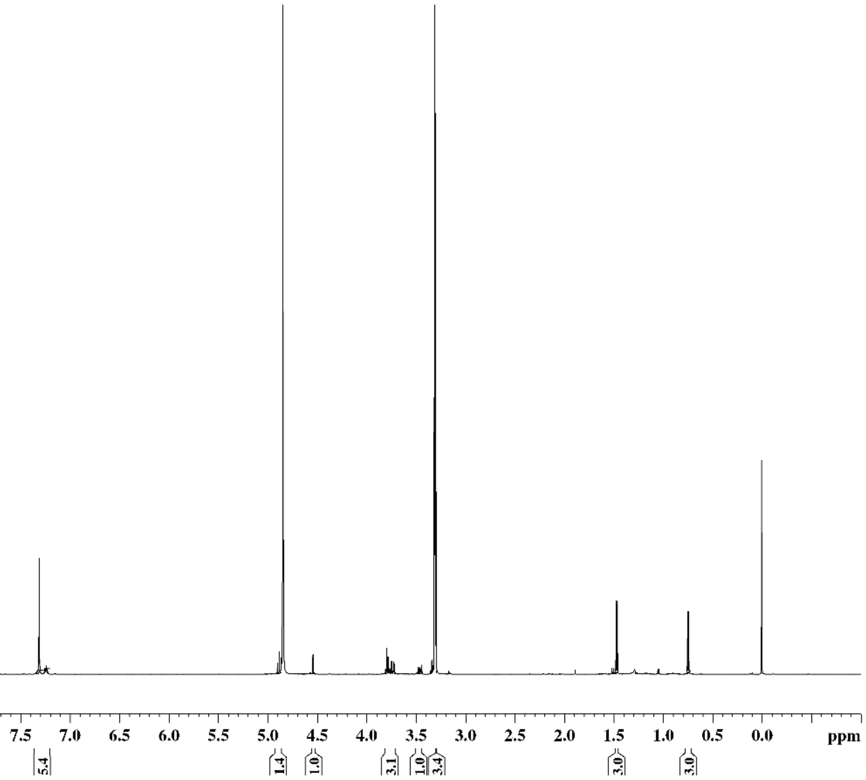

^{13}C NMR (125.8 MHz, MeOD, 25°C):

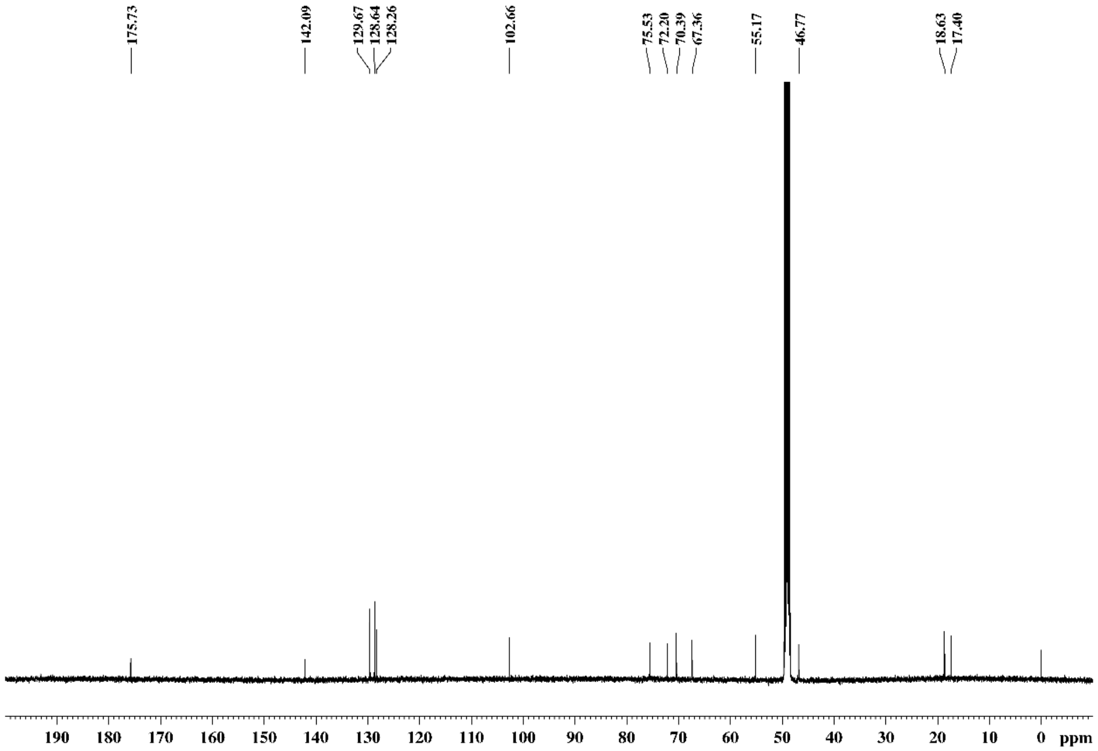

Methyl 2,3-*O*-isopropylidene-4-*O*-(*S*)-2-phenyl-propanoyl-α-L-rhamnopyranoside (115):

^{1}H NMR (500.20 MHz, $CDCl_3$, 25°C):

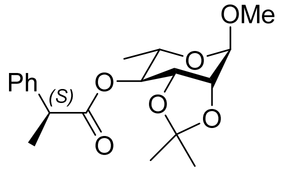

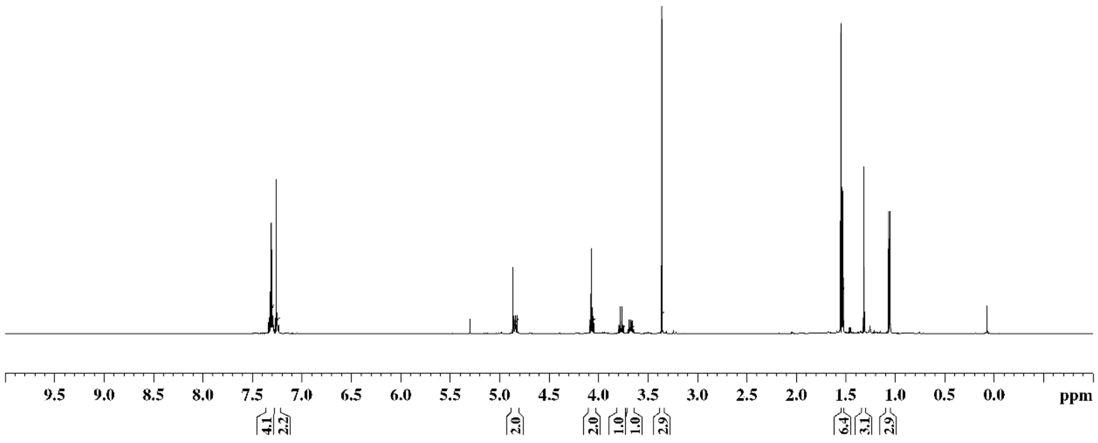

^{13}C NMR (125.8 MHz, $CDCl_3$, 25°C):

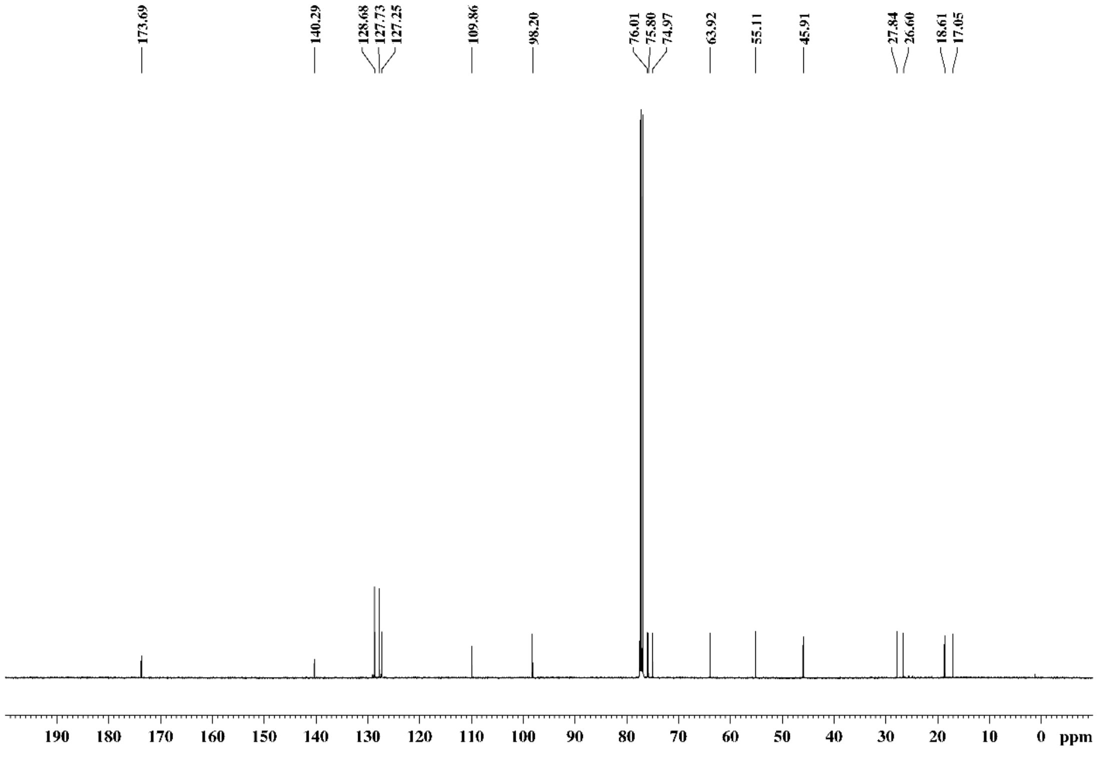

Methyl 4-*O*-(*S*)-2-phenyl-propanoyl-α-L-rhamnopyranoside (50):

^{1}H NMR (500.20 MHz, MeOD, 25°C):

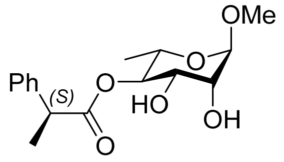

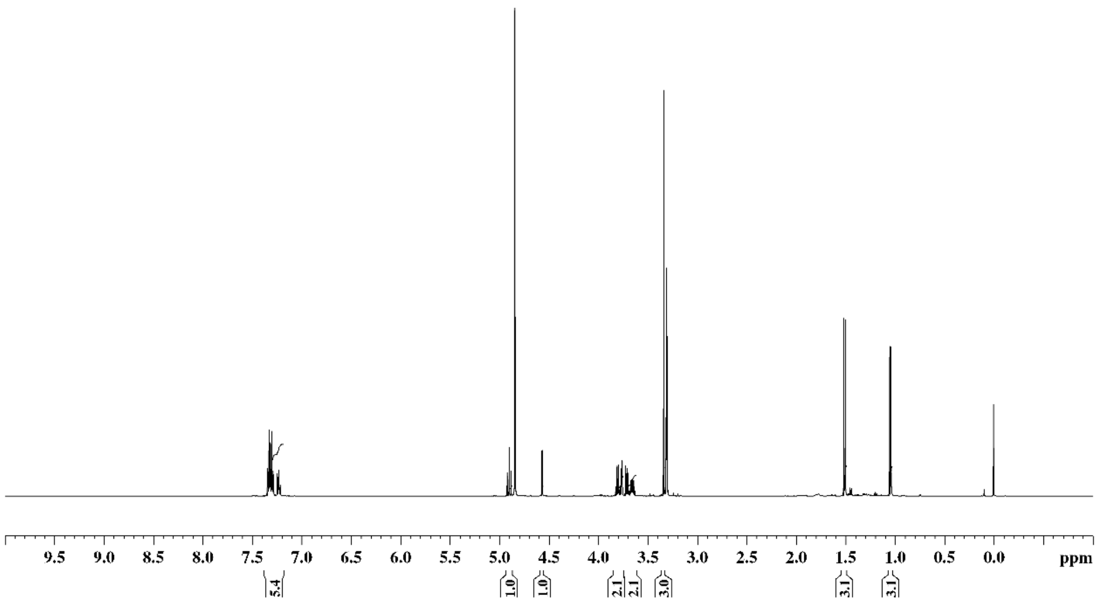

^{13}C NMR (125.8 MHz, MeOD, 25°C):

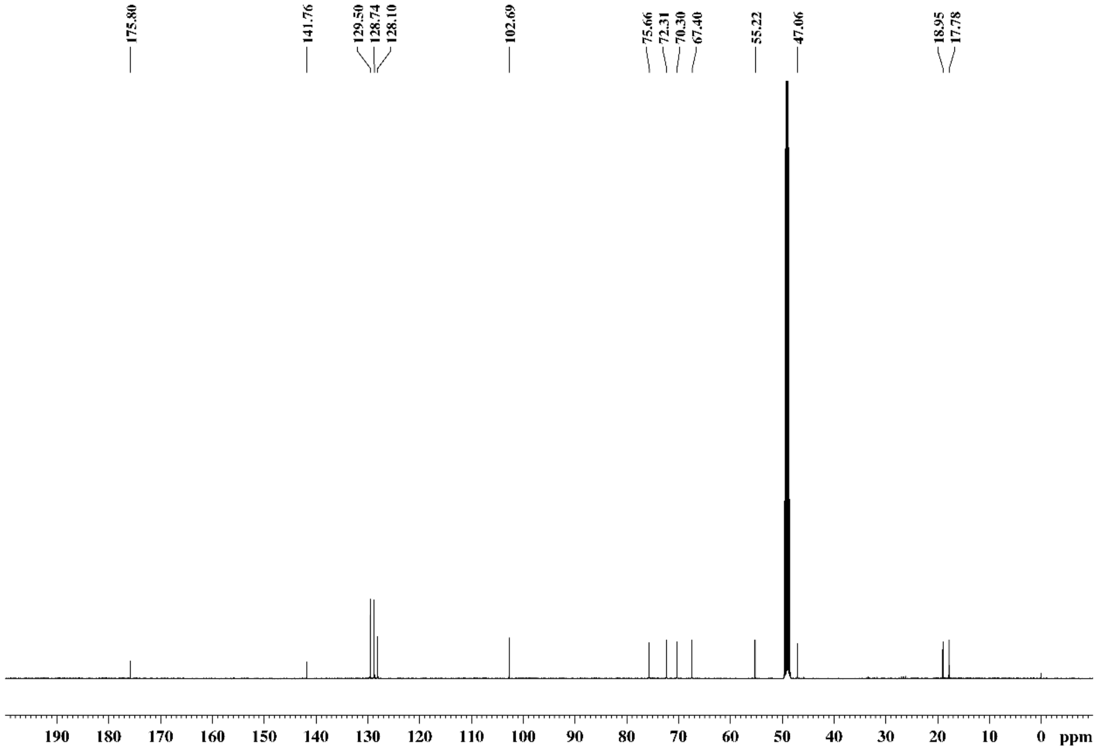

Methyl 2-*O*-acetyl-3,4-*O*-isopropylidene-β-L-fucopyranoside (117):

^{1}H NMR (500.20 MHz, $CDCl_3$, 25°C):

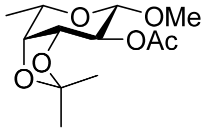

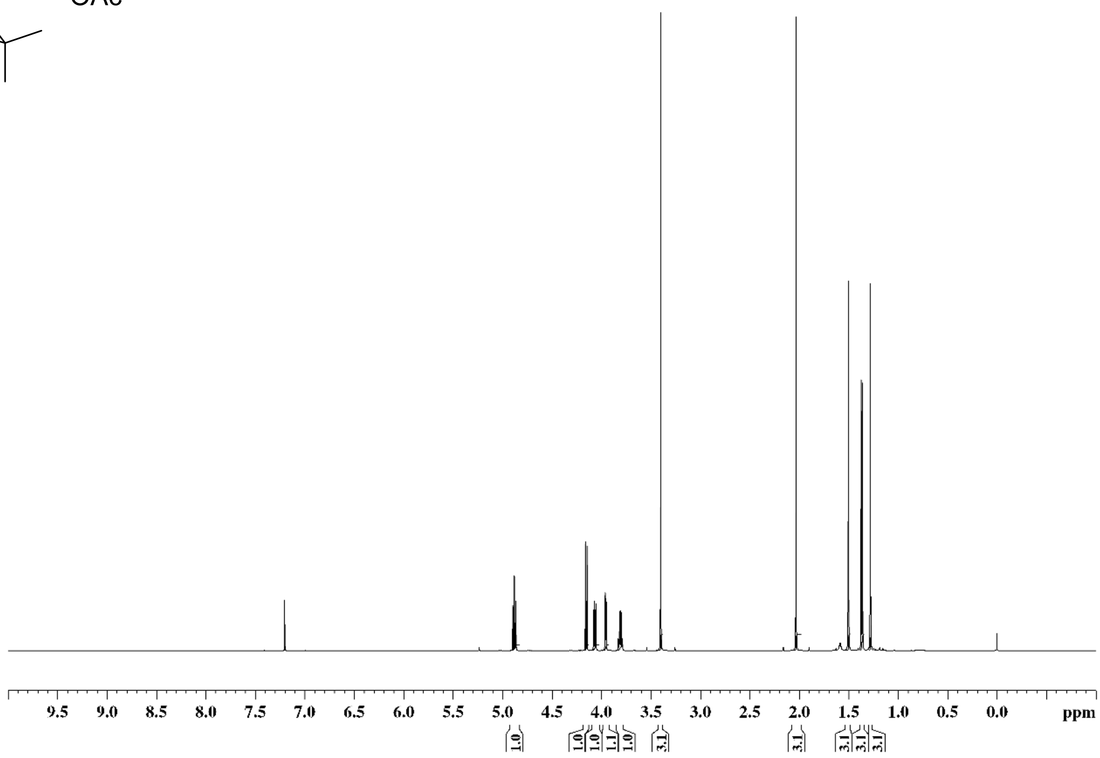

^{13}C NMR (125.8 MHz, $CDCl_3$, 25°C):

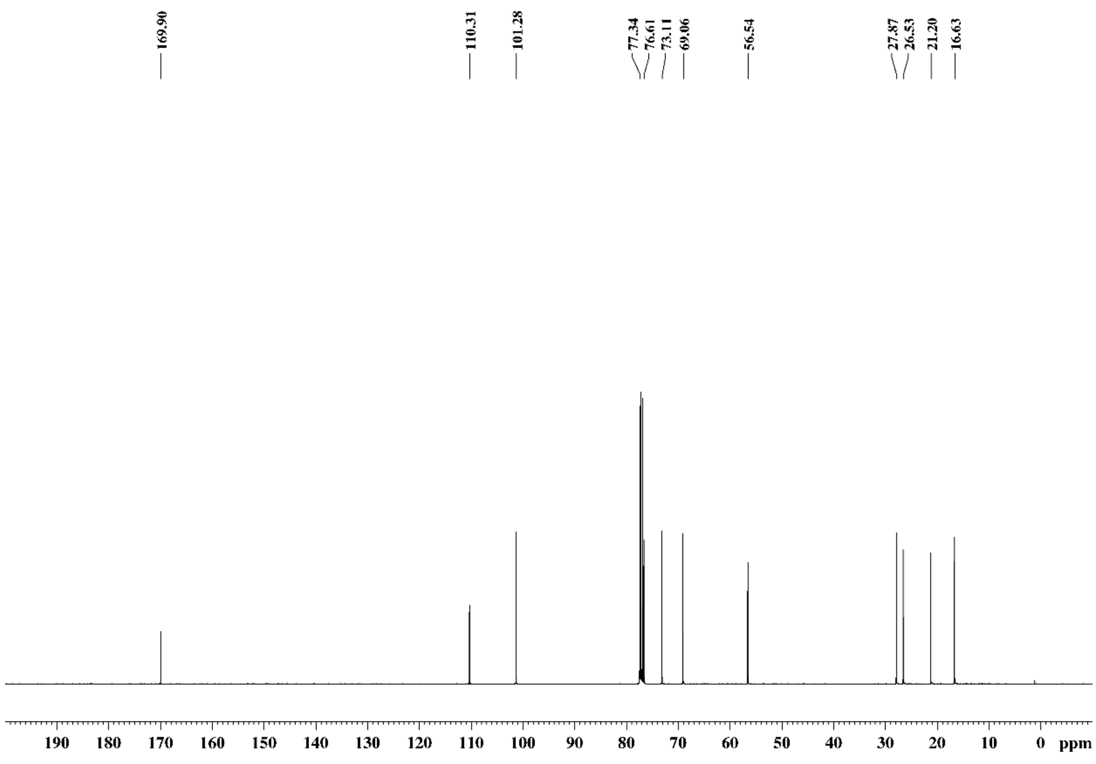

Methyl 2-*O*-acetyl-β-L-fucopyranoside (51):

^{1}H NMR (500.20 MHz, MeOD, 25°C):

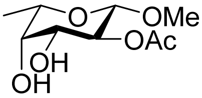

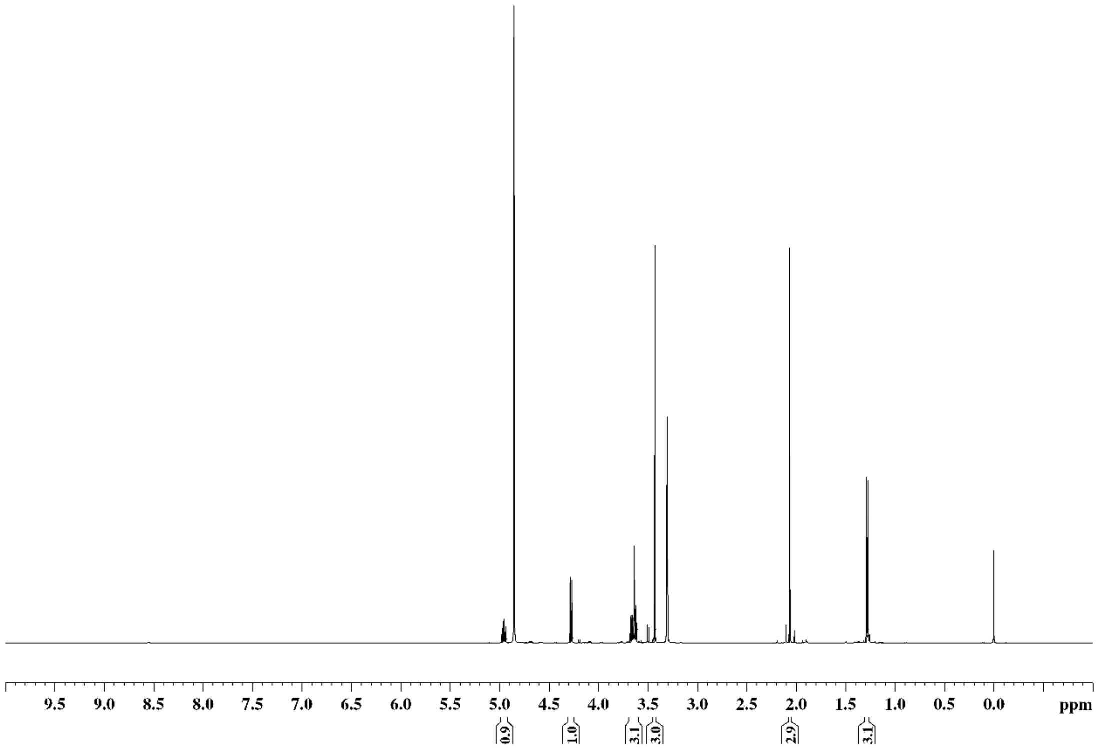

^{13}C NMR (125.8 MHz, MeOD, 25°C):

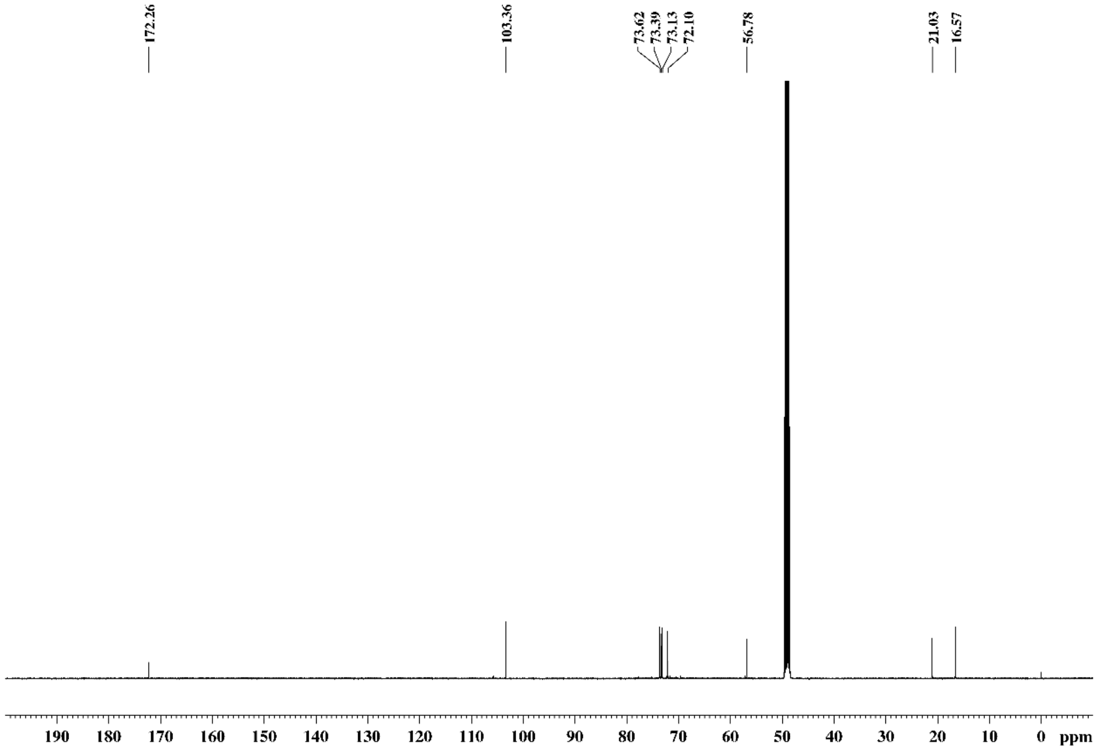

Methyl 2-*O*-benzoyl-3,4-*O*-isopropylidene-β-L-fucopyranoside (118):

^{1}H NMR (500.20 MHz, $CDCl_3$, 25°C):

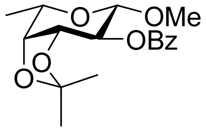

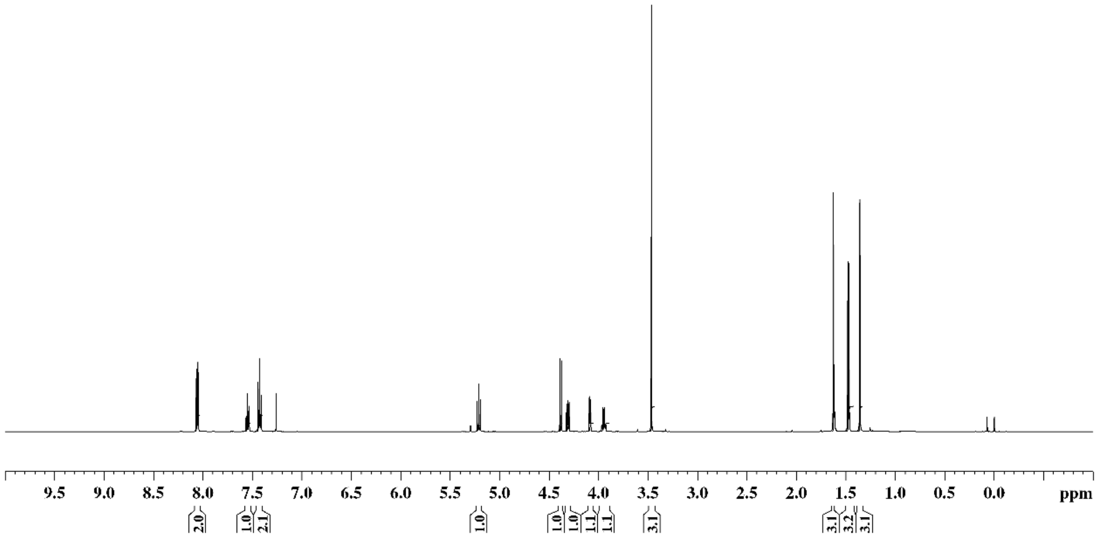

^{13}C NMR (125.8 MHz, $CDCl_3$, 25°C):

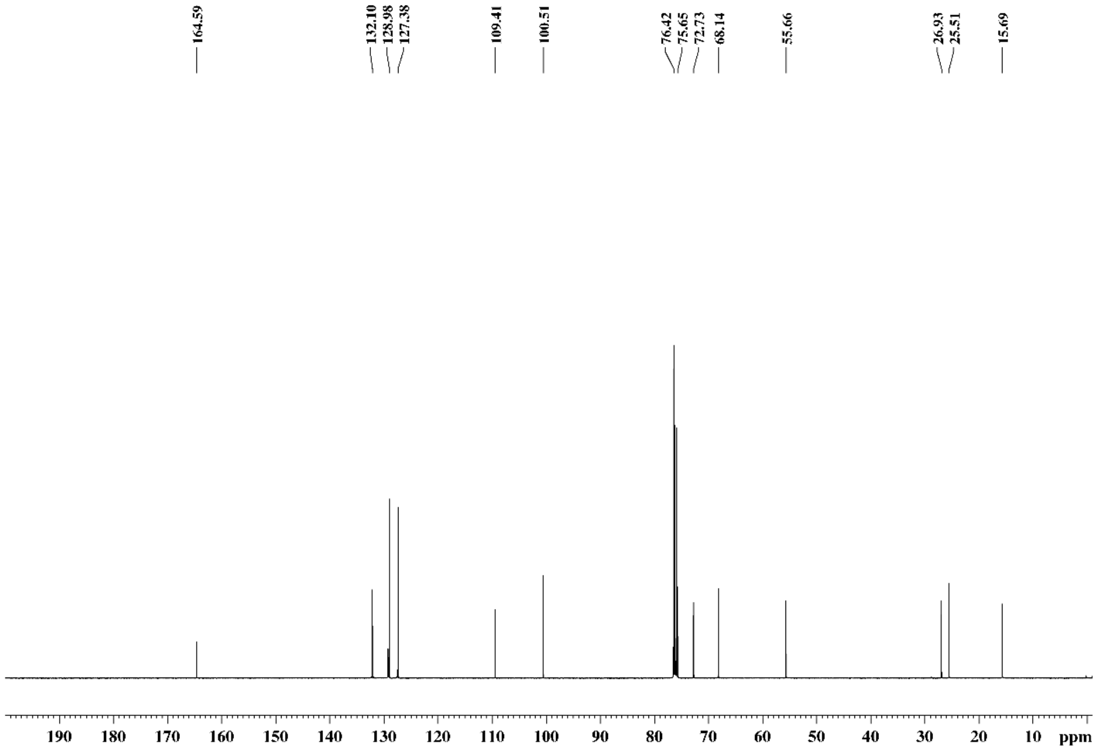

Methyl 2-*O*-benzoyl-β-L-fucopyranoside (52):

^{1}H NMR (500.20 MHz, MeOD, 25°C):

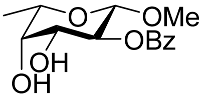

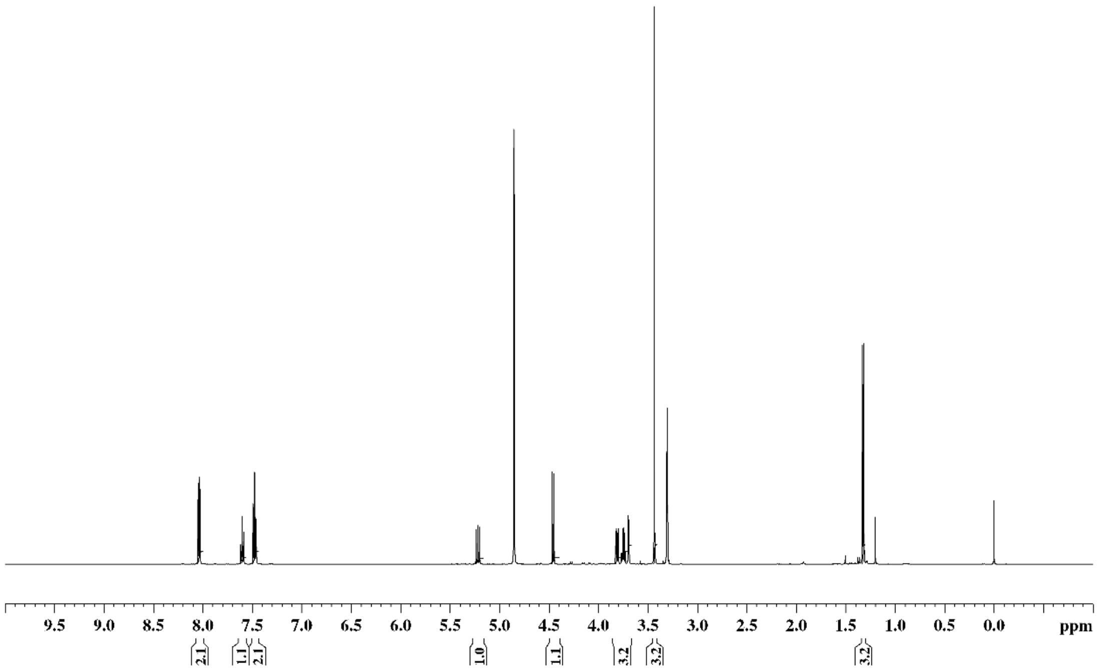

^{13}C NMR (125.8 MHz, MeOD, 25°C):

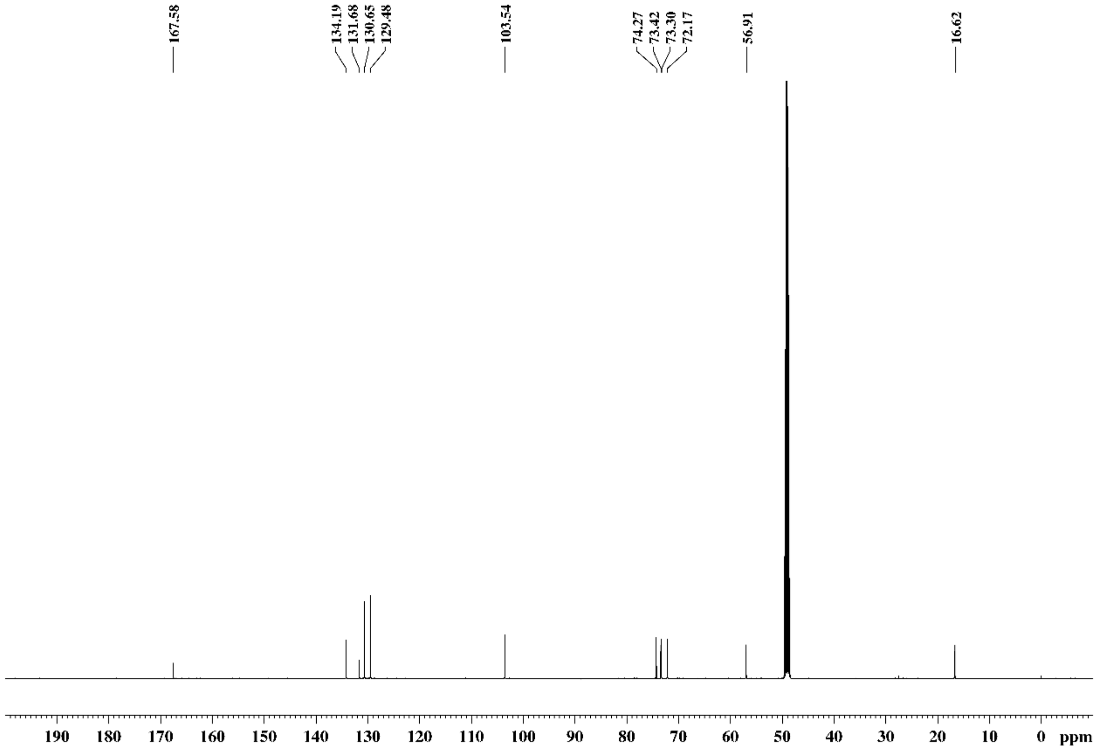

Methyl 3,4-*O*-isopropylidene-2-*O*-pivaoyl-β-L-fucopyranoside (119):

^{1}H NMR (500.20 MHz, $CDCl_3$, 25°C):

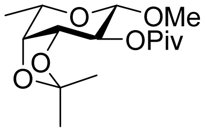

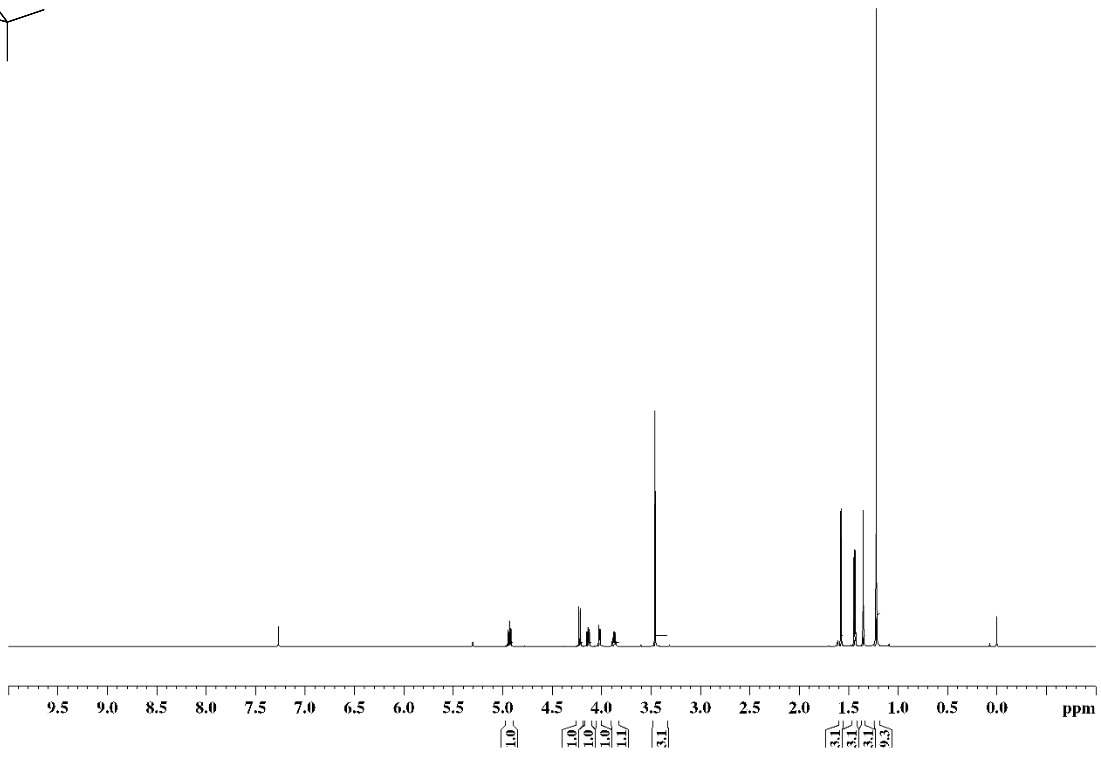

^{13}C NMR (125.8 MHz, $CDCl_3$, 25°C):

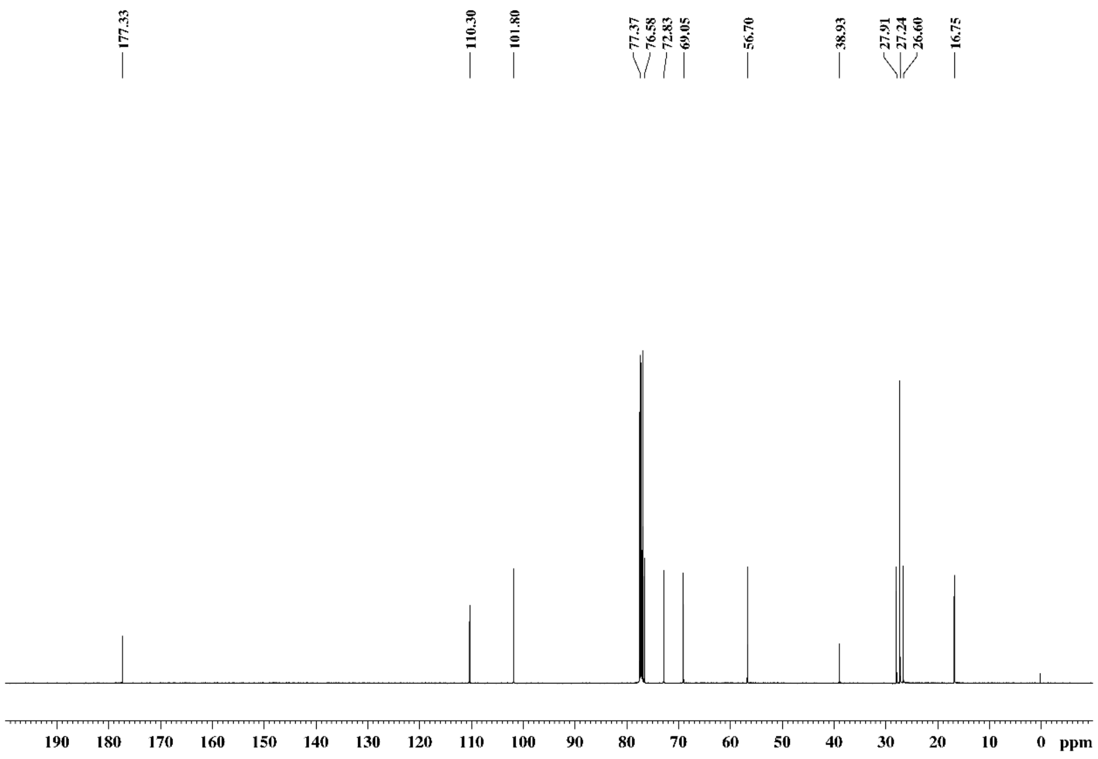

Methyl 2-*O*-pivaoyl-β-L-fucopyranoside (53):

^{1}H NMR (500.20 MHz, MeOD, 25°C):

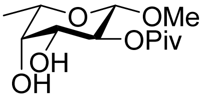

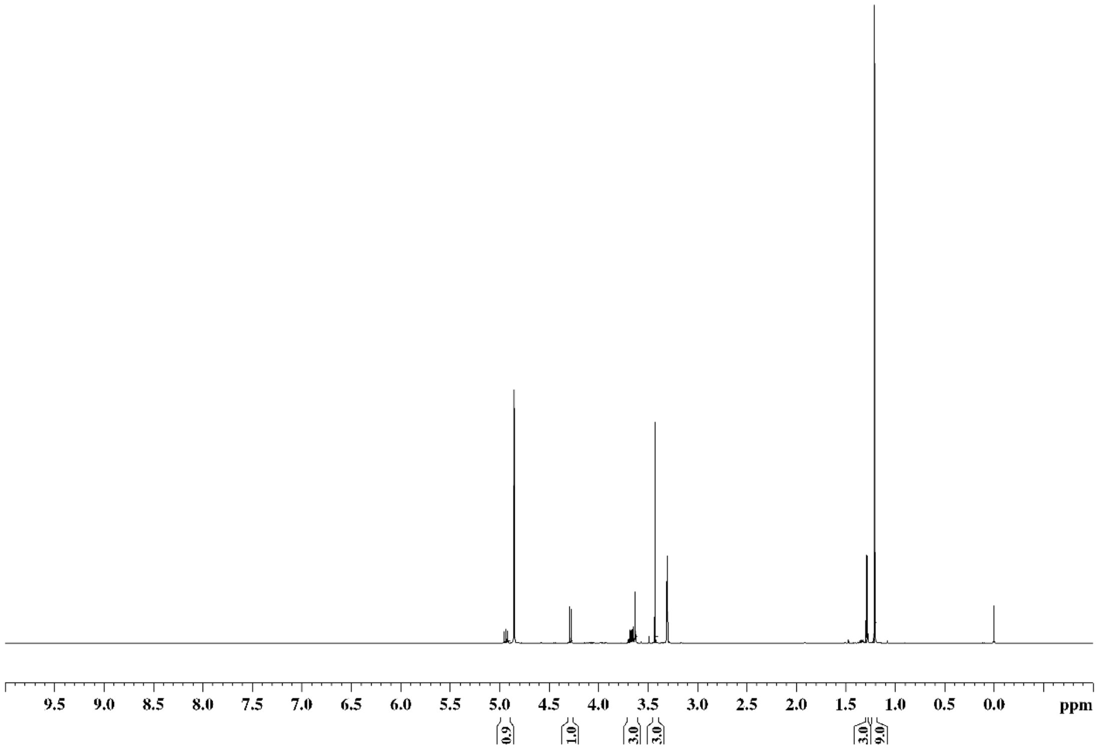

^{13}C NMR (125.8 MHz, MeOD, 25°C):

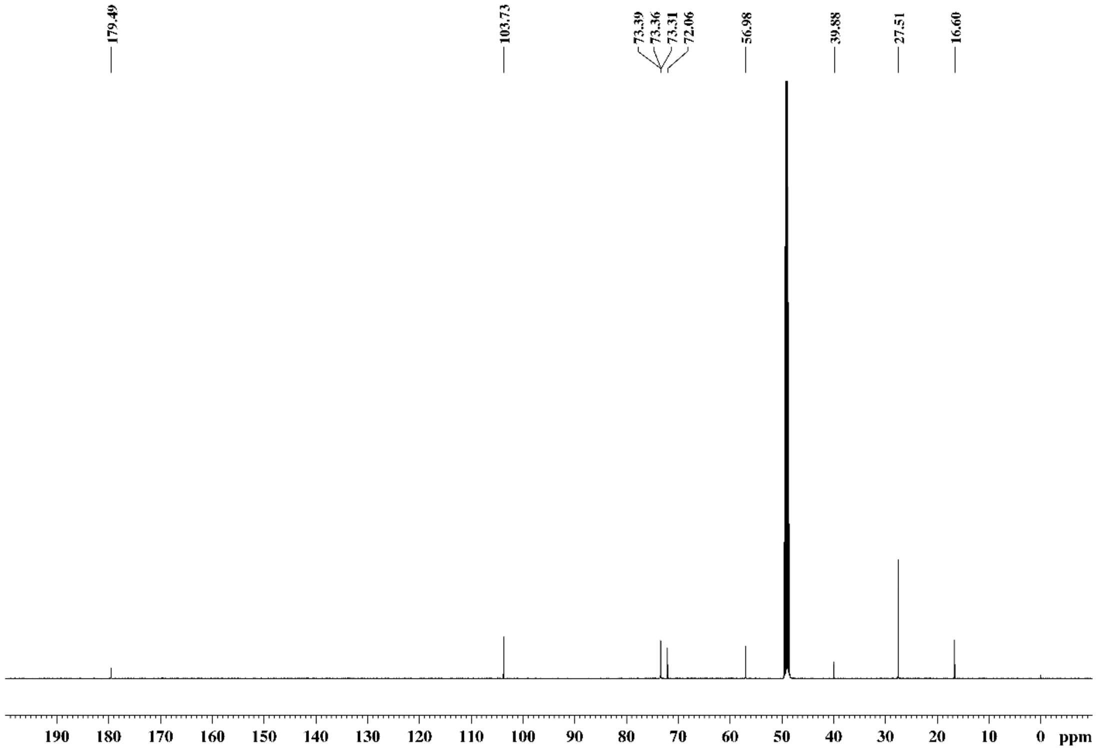

Methyl 3,4-*O*-isopropylidene-2-*O*-(*R*)-2-phenyl-propanoyl-β-L-fucopyranoside (120):

^{1}H NMR (500.20 MHz, $CDCl_3$, 25°C):

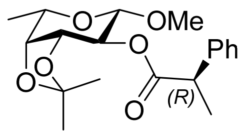

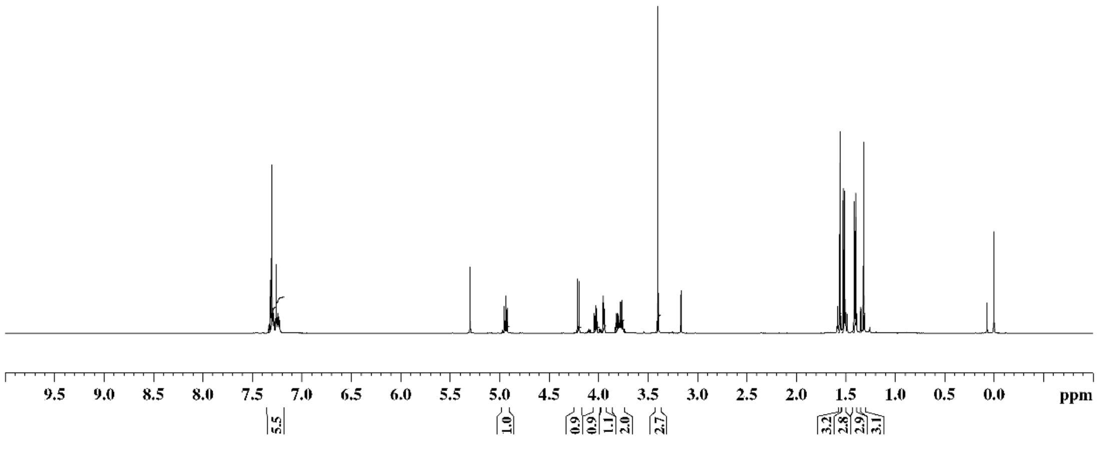

^{13}C NMR (125.8 MHz, $CDCl_3$, 25°C):

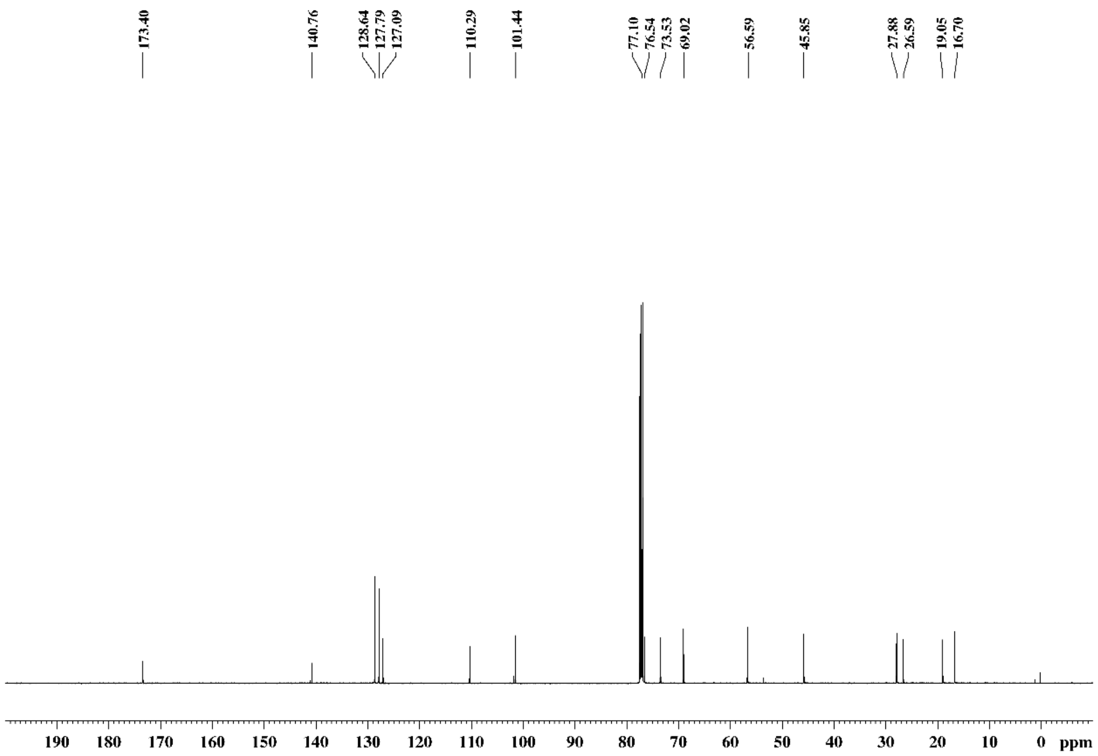

Methyl 2-*O*-(*R*)-2-phenyl-propanoyl-β-L-fucopyranoside (54):

^{1}H NMR (500.20 MHz, MeOD, 25°C):

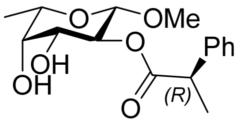

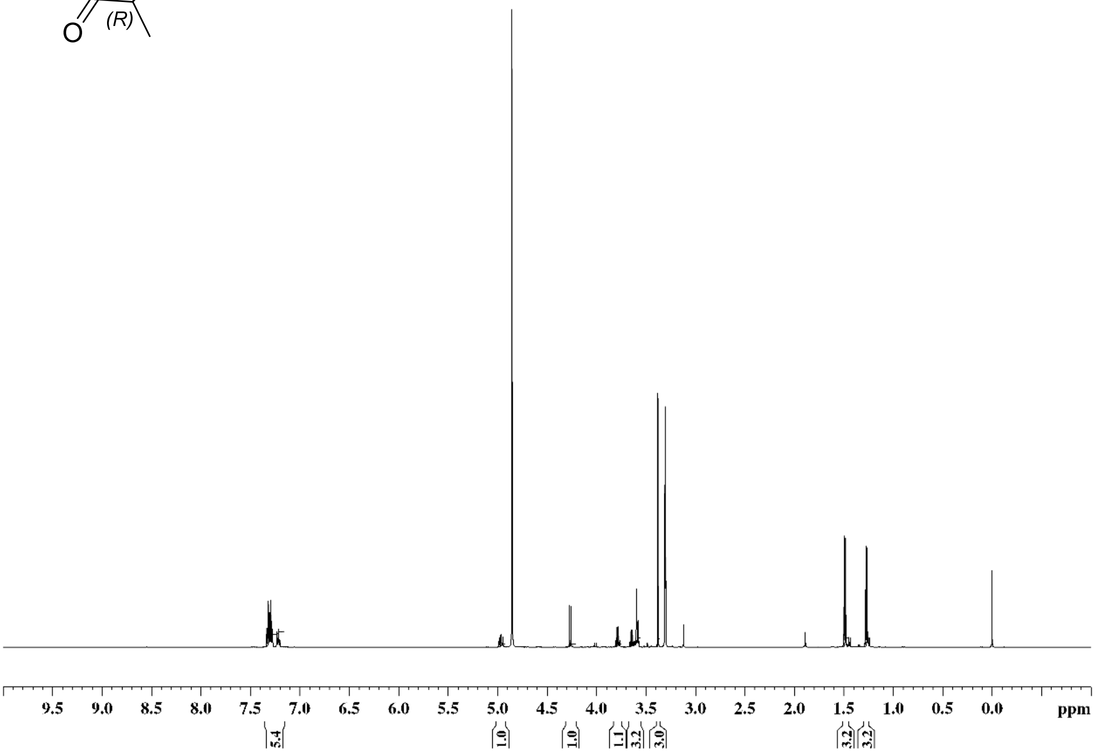

^{13}C NMR (125.8 MHz, MeOD, 25°C):

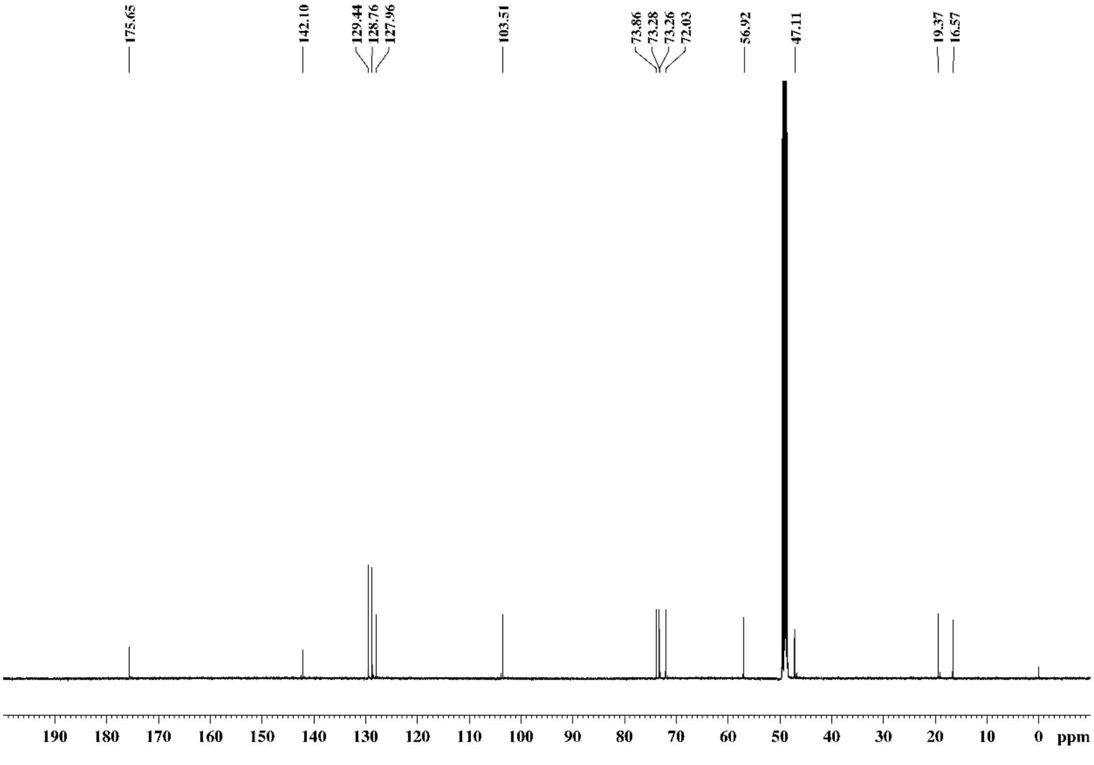

Methyl 3,4-*O*-isopropylidene-2-*O*-(*S*)-2-phenyl-propanoyl-β-L-fucopyranoside (121):

^{1}H NMR (500.20 MHz, $CDCl_3$, 25°C):

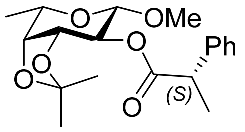

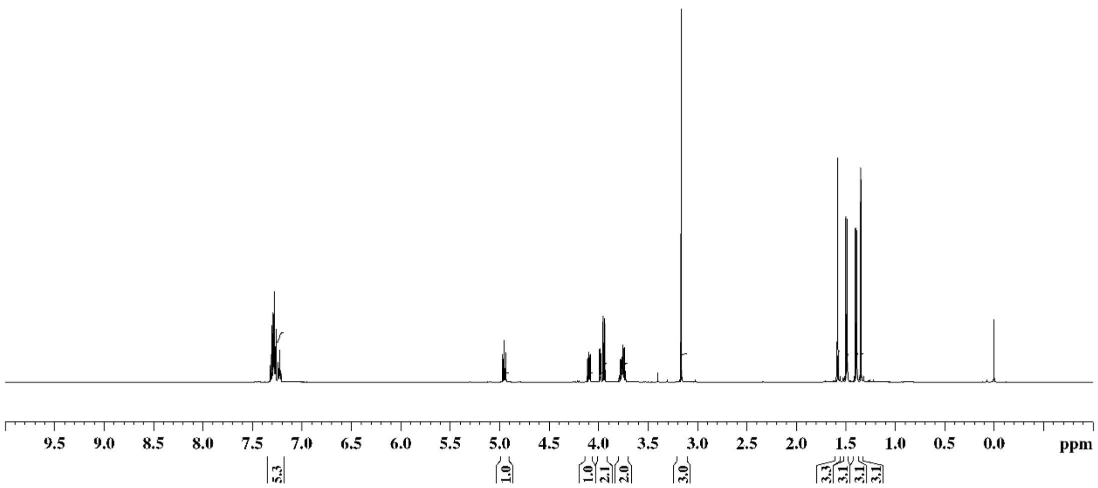

^{13}C NMR (125.8 MHz, $CDCl_3$, 25°C):

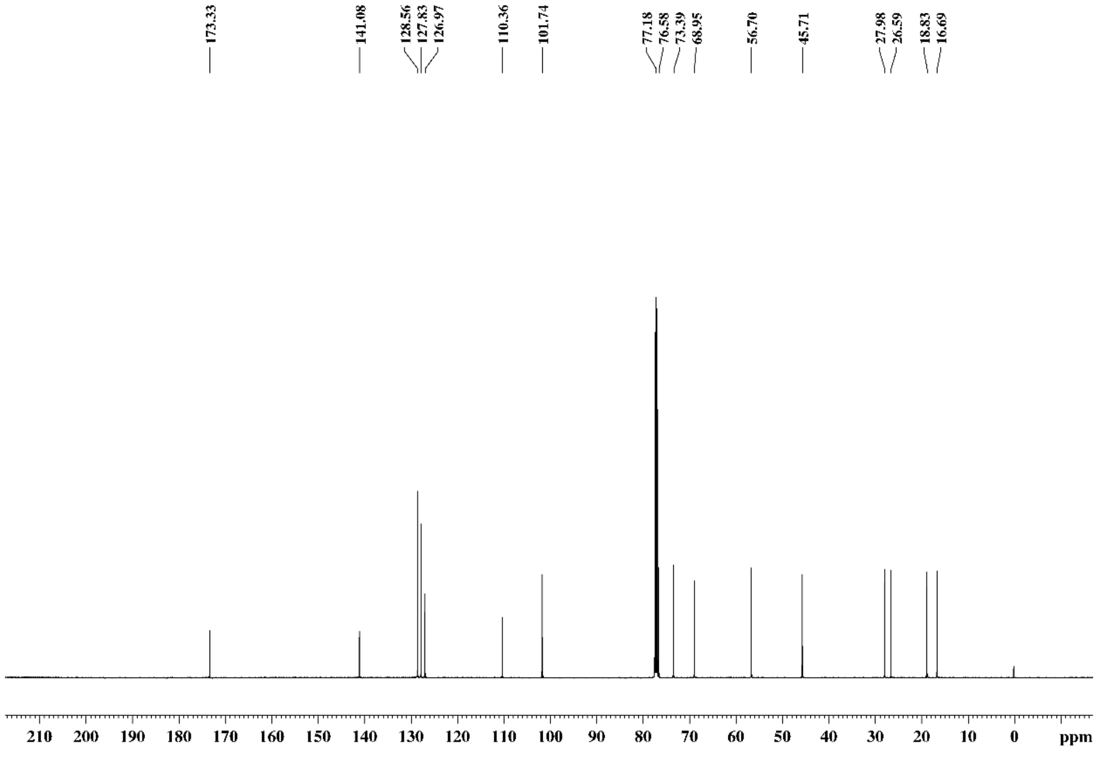

Methyl 2-*O*-(*S*)-2-phenyl-propanoyl-β-L-fucopyranoside (55):

^{1}H NMR (500.20 MHz, MeOD, 25°C):

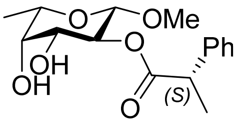

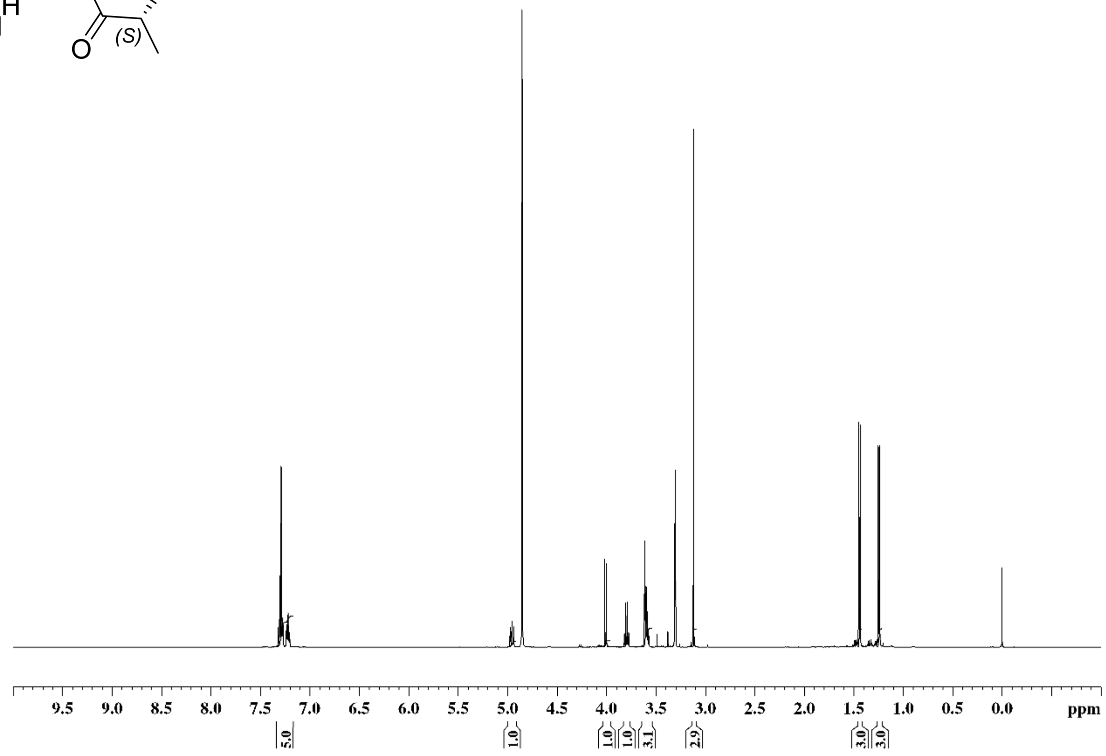

^{13}C NMR (125.8 MHz, MeOD, 25°C):

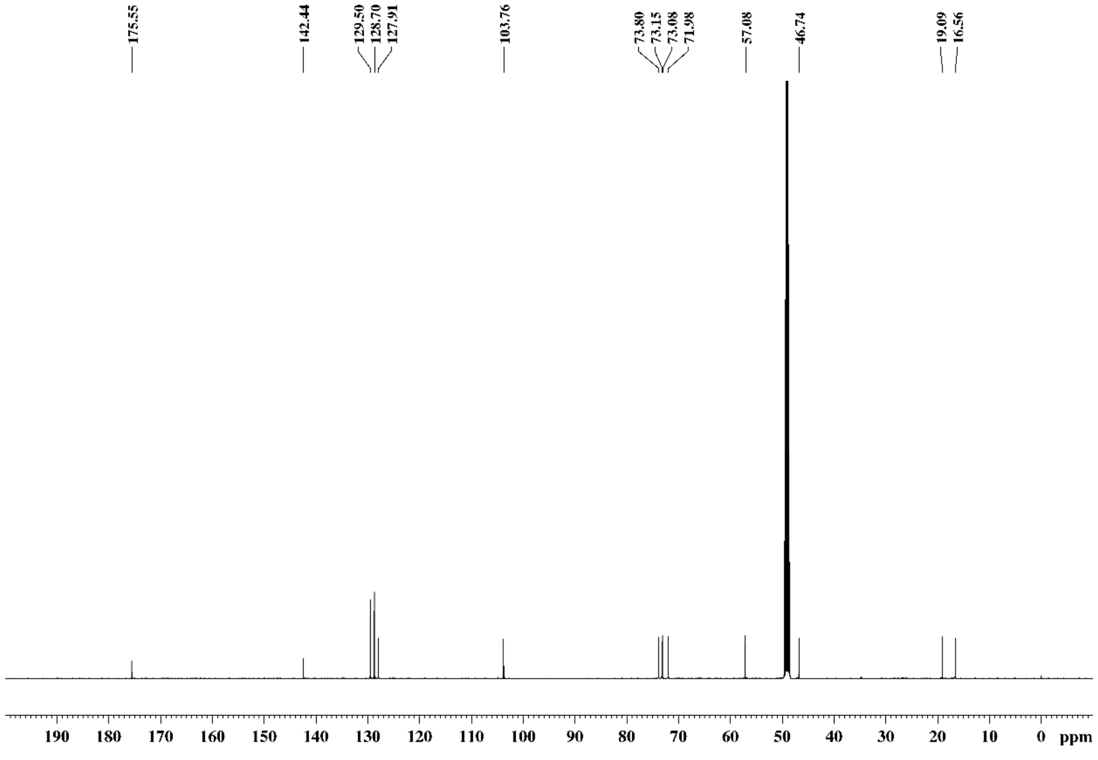

Migration Kinetics

Calculation of the rate constants

Reaction kinetics for the acetyl, benzoyl and pivaloyl group migration for compounds **1**, **2**, **3**, **6**, **7**, **8**, **11**, **12**, **13**, **16**, **17**, **18**, **21**, **22** and **23** were described with a reversible (O2⇌O3, O3⇌O4, and O4⇌O6 migration) and irreversible (hydrolysis) first order reaction scheme as follows

$$r_1 = k_1 \times c(\mathbf{Xa}) - k_{-1} \times c(\mathbf{Xb})$$

$$r_2 = k_2 \times c(\mathbf{Xb}) - k_{-2} \times c(\mathbf{Xc})$$

$$r_3 = k_3 \times c(\mathbf{Xc}) - k_{-3} \times c(\mathbf{Xd})$$

$$r_4 = k_4 \times c(\mathbf{Xa})$$

$$r_5 = k_4 \times c(\mathbf{Xb})$$

$$r_6 = k_4 \times c(\mathbf{Xc})$$

$$r_7 = k_4 \times c(\mathbf{Xd})$$

where

$$k_4 = k_{\boldsymbol{Xa \to Xe}} = k_{\boldsymbol{Xb \to Xe}} = k_{\boldsymbol{Xc \to Xe}} = k_{\boldsymbol{Xd \to Xe}}$$

The mass balances become

$$\frac{dc_{\boldsymbol{Xa}}}{dt} = -r_1 - r_4$$

$$\frac{dc_{\boldsymbol{Xb}}}{dt} = r_1 - r_2 - r_5$$

$$\frac{dc_{\boldsymbol{Xc}}}{dt} = r_2 - r_3 - r_6$$

$$\frac{dc_{\boldsymbol{Xd}}}{dt} = r_3 - r_7$$

$$\frac{dc_{\boldsymbol{Xe}}}{dt} = r_4 + r_5 + r_6 + r_7$$

The reaction kinetics for the *R*- and *S*-2-pH-propanoyl migration for compounds **4**, **5**, **9**, **10**, **14**, **15**, **19**, **20**, **24** and **25** were described with a reversible (O2⇌O3, O3⇌O4, and O4⇌O6 migration) and irreversible (hydrolysis and hydrolysis from primary position) first order reaction scheme as follows

$$r_1 = k_1 \times c(\mathbf{Xa}) - k_{-1} \times c(\mathbf{Xb})$$

$$r_2 = k_2 \times c(\mathbf{Xb}) - k_{-2} \times c(\mathbf{Xc})$$

$$r_3 = k_3 \times c(\mathbf{Xc}) - k_{-3} \times c(\mathbf{Xd})$$

$$r_4 = k_4 \times c(\mathbf{Xa})$$

$$r_5 = k_4 \times c(\mathbf{Xb})$$

$$r_6 = k_4 \times c(\mathbf{Xc})$$

$$r_7 = k_5 \times c(\mathbf{Xd})$$

where

$$k_4 = k_{\boldsymbol{Xa \to Xe}} = k_{\boldsymbol{Xb \to Xe}} = k_{\boldsymbol{Xc \to Xe}}$$

The mass balances become

$$\frac{dc_{\boldsymbol{Xa}}}{dt} = -r_1 - r_4$$

$$\frac{dc_{\boldsymbol{Xb}}}{dt} = r_1 - r_2 - r_5$$

$$\frac{dc_{\boldsymbol{Xc}}}{dt} = r_2 - r_3 - r_6$$

$$\frac{dc_{\boldsymbol{Xd}}}{dt} = r_3 - r_7$$

$$\frac{dc_{\boldsymbol{Xe}}}{dt} = r_4 + r_5 + r_6 + r_7$$

The reaction kinetics for the acyl group migration for compounds **26** – **55** were described with a reversible (O2⇌O3, and O3⇌O4 migration) and irreversible (hydrolysis) first order reaction scheme as

$$r_1 = k_1 \times c(\mathbf{Xa}) - k_{-1} \times c(\mathbf{Xb})$$

$$r_2 = k_2 \times c(\mathbf{Xb}) - k_{-2} \times c(\mathbf{Xc})$$

$$r_3 = k_3 \times c(\mathbf{Xa})$$

$$r_4 = k_3 \times c(\mathbf{Xb})$$

$$r_5 = k_3 \times c(\mathbf{Xc})$$

where

$$k_3 = k_{\boldsymbol{Xa \to Xd}} = k_{\boldsymbol{Xb \to Xd}} = k_{\boldsymbol{Xc \to Xd}}$$

The mass balances become

$$\frac{dc_{\boldsymbol{Xa}}}{dt} = -r_1 - r_3$$

$$\frac{dc_{\boldsymbol{Xb}}}{dt} = r_1 - r_2 - r_4$$

$$\frac{dc_{\boldsymbol{Xc}}}{dt} = r_2 - r_5$$

$$\frac{dc_{\boldsymbol{Xd}}}{dt} = r_3 + r_4 + r_5$$

The differential equations were solved with the backward difference method as a subtask to the optimizing methods (Simplex and/or Levenberg–Marquardt) with the software Modest.[11] As objective function, the sum of square function was used:

$$SSQ = \sum_t \sum_i (c_{i,t,model} - c_{i,t,experiment})^2$$

Errors in the rate constants shown are standard error = variance/sqrt(N) (N: number of samples), and within 95% confidence interval. Single experiments are used for modelling for particular compounds, but with the use of mathematical kinetic modelling combined with experimental methods one can identify if an experimental point or one complete experiment series for a compound does not fit well to the model. Such experiments are then repeated or the analysis of a specific data point is rechecked.

Fitting of the experimental data to the model

Glucopyranoside derivatives

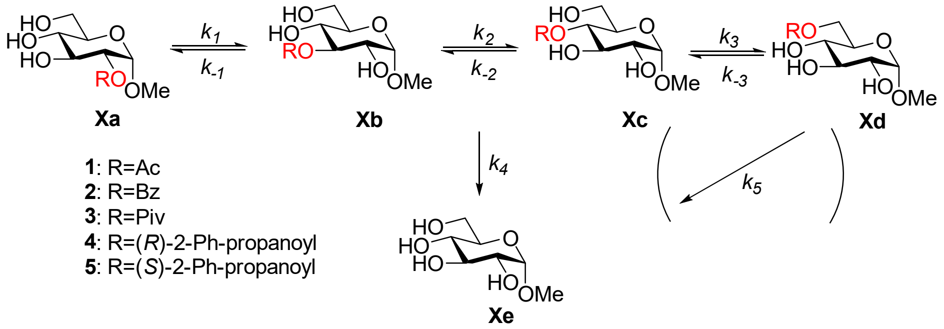

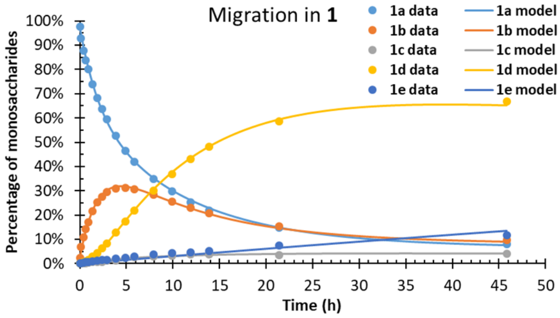

Figure S1. The experimental data and the kinetic model of the migration in **1**. Degree of explanation: 99.90%.

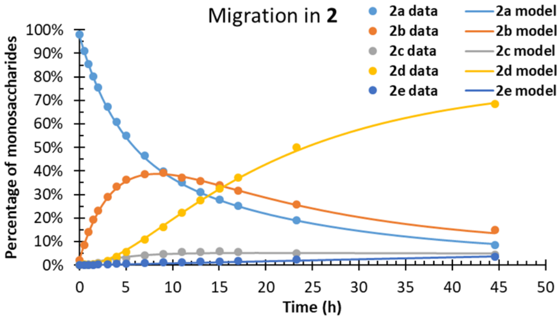

Figure S2. The experimental data and the kinetic model of the migration in **2**. Degree of explanation: 99.95%.

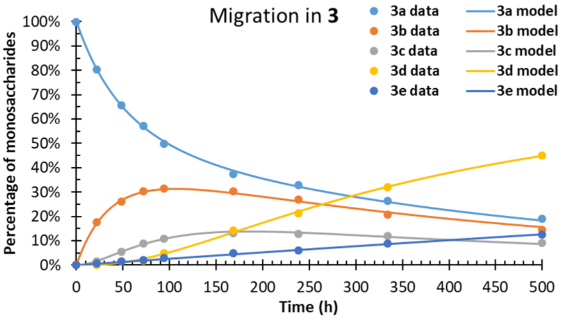

Figure S3. The experimental data and the kinetic model of the migration in **3**. Degree of explanation: 99.92%

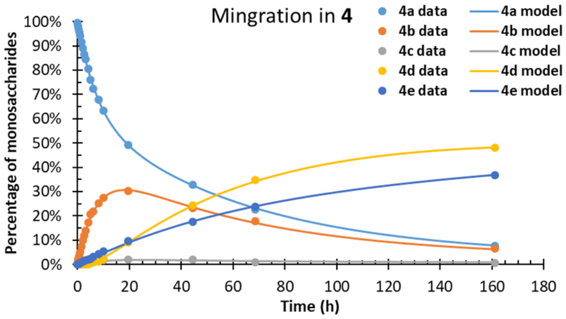

Figure S4. The experimental data and the kinetic model of the migration in **4**. Degree of explanation: 99.99%

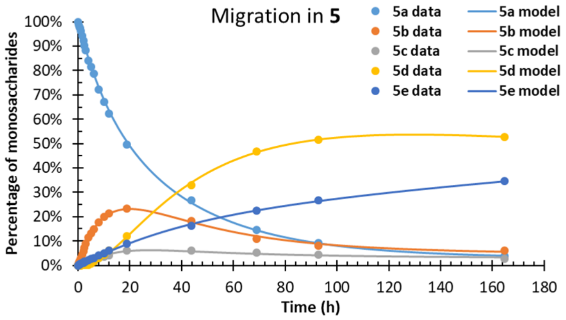

Figure S5. The experimental data and the kinetic model of the migration in **5**. Degree of explanation: 99.98%

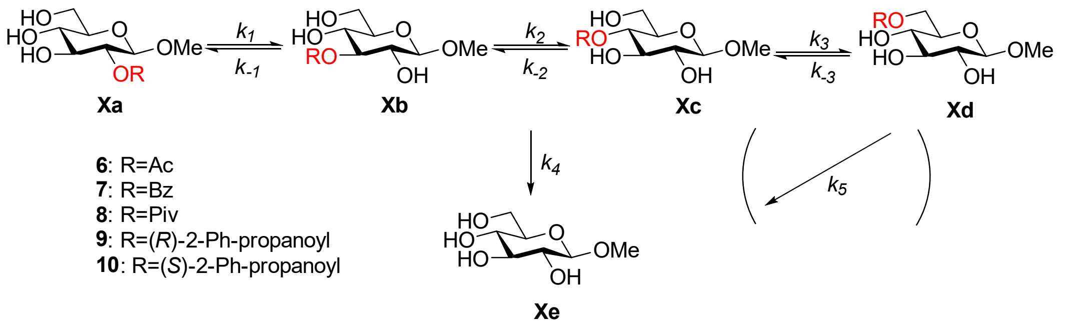

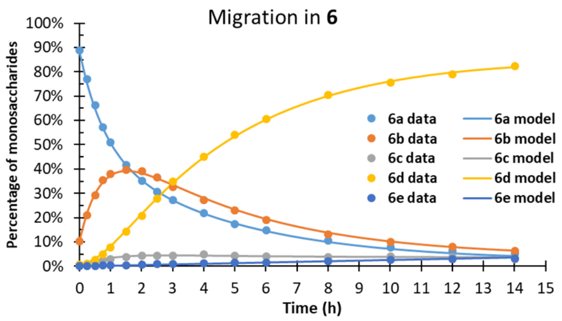

Figure S6. The experimental data and the kinetic model of the migration in **6**. Degree of explanation: 99.96%

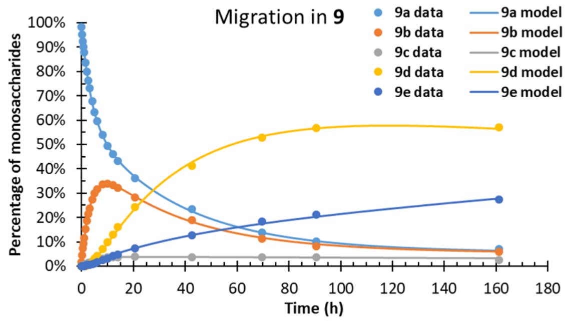

Figure S9. The experimental data and the kinetic model of the migration in **9**. Degree of explanation: 99.87%

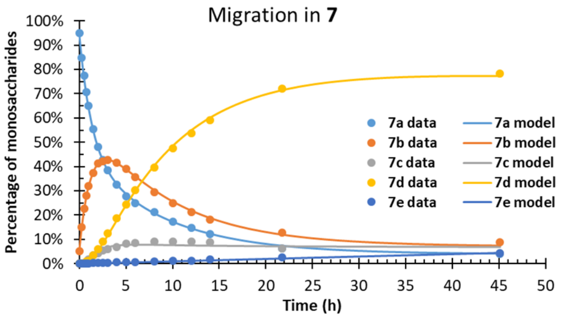

Figure S7. The experimental data and the kinetic model of the migration in **7**. Degree of explanation: 99.89%

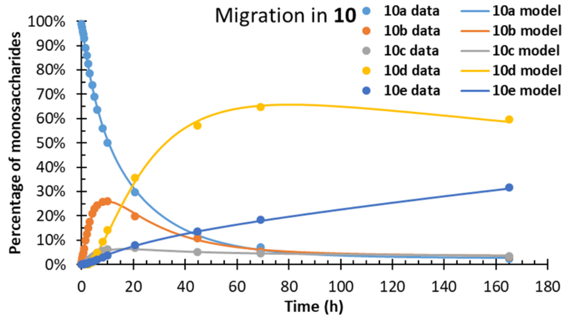

Figure S10. The experimental data and the kinetic model of the migration in **10**. Degree of explanation: 99.94%

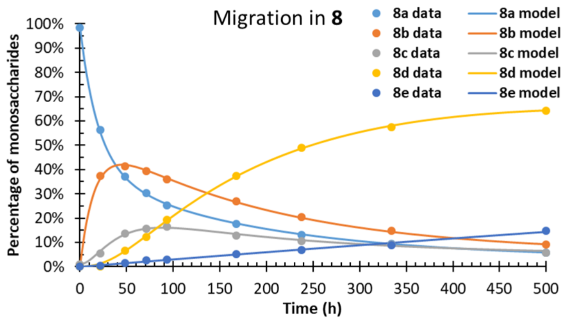

Figure S8. The experimental data and the kinetic model of the migration in **8**. Degree of explanation: 99.91%

Galactopyranoside derivatives

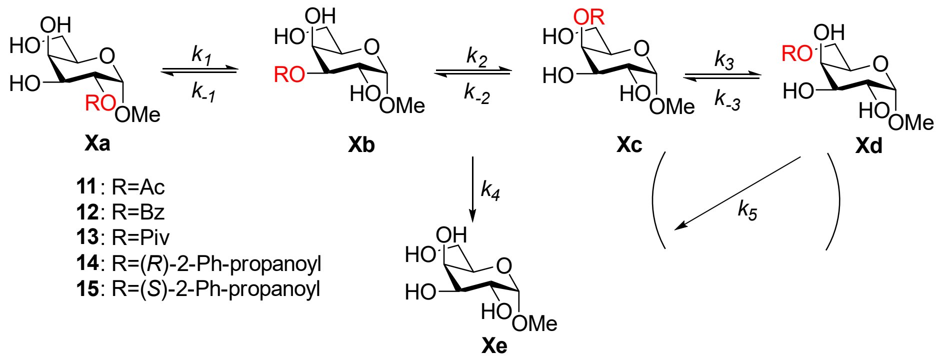

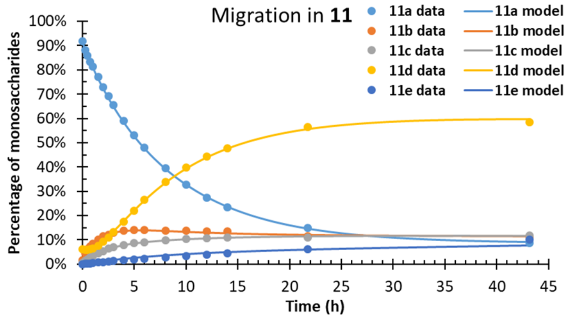

Figure S11. The experimental data and the kinetic model of the migration in **11**. Degree of explanation: 99.96%

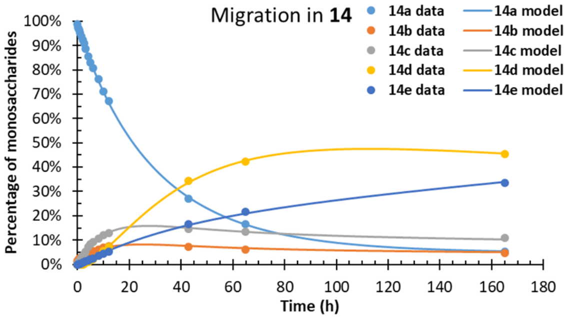

Figure S14. The experimental data and the kinetic model of the migration in **14**. Degree of explanation: 99.99%

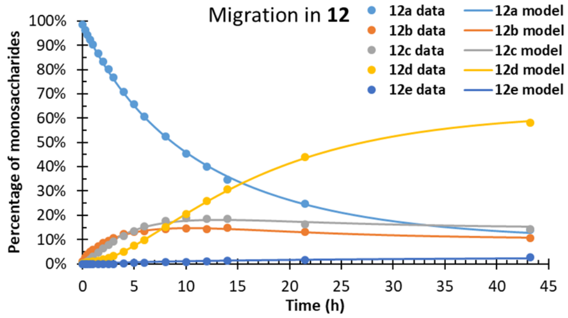

Figure S12. The experimental data and the kinetic model of the migration in **12**. Degree of explanation: 99.98%

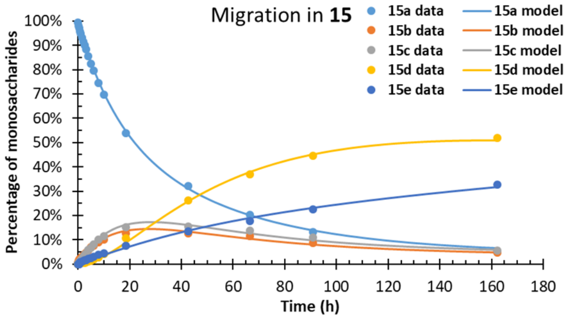

Figure S54. The experimental data and the kinetic model of the migration in **15**. Degree of explanation: 99.96%

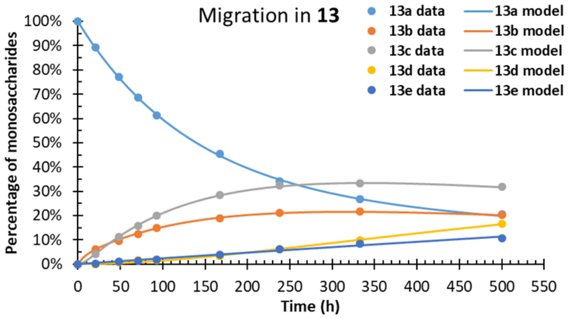

Figure S13. The experimental data and the kinetic model of the migration in **13**. Degree of explanation: 99.98%

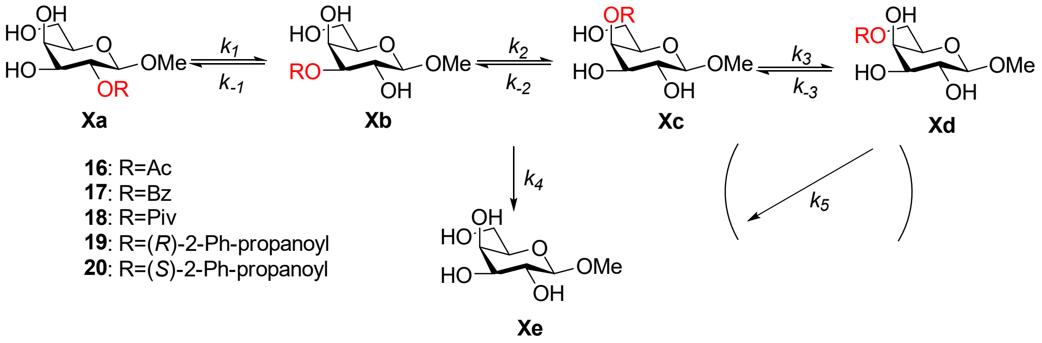

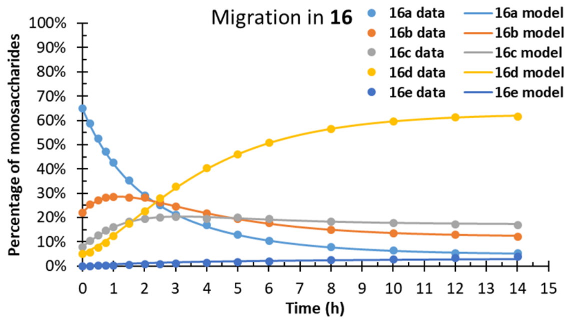

Figure S16. The experimental data and the kinetic model of the migration in **16**. Degree of explanation: 99.95%

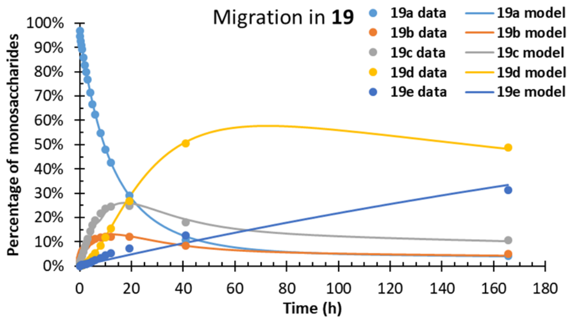

Figure S19. The experimental data and the kinetic model of the migration in **19**. Degree of explanation: 99.92%

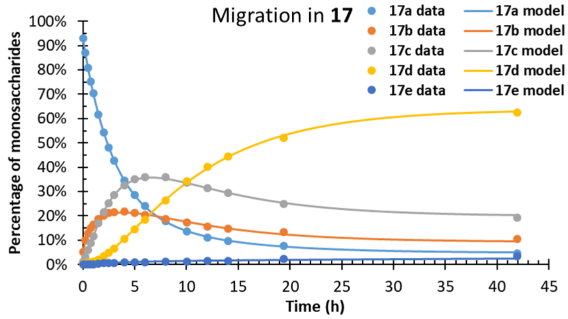

Figure S17. The experimental data and the kinetic model of the migration in **17**. Degree of explanation: 99.97%

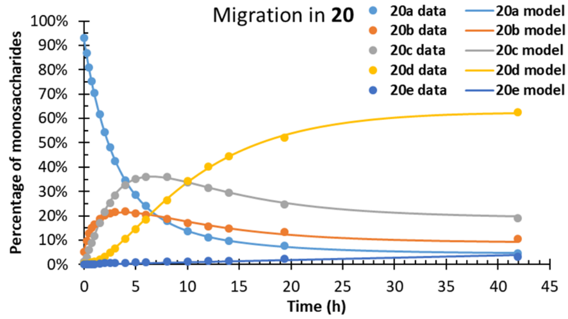

Figure S20. The experimental data and the kinetic model of the migration in **20**. Degree of explanation: 99.97%

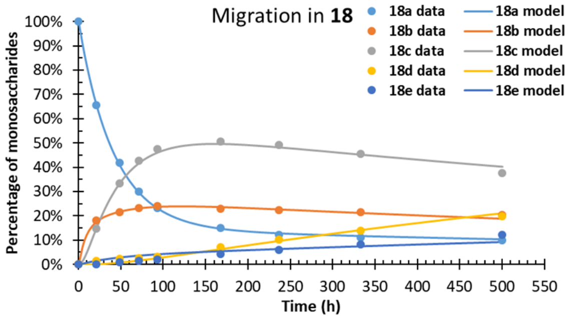

Figure S18. The experimental data and the kinetic model of the migration in **18**. Degree of explanation: 99.85%

Mannopyranoside derivatives

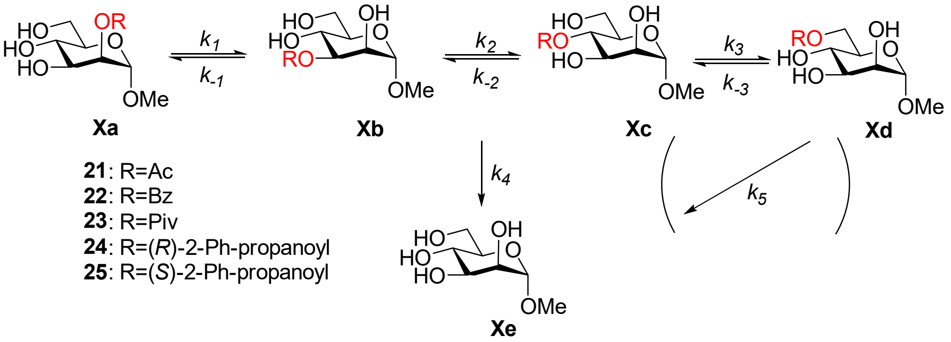

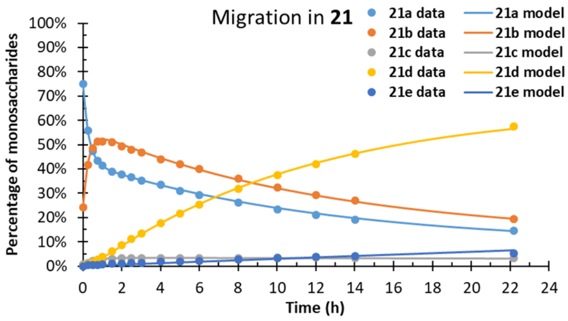

Figure S21. The experimental data and the kinetic model of the migration in **21**. Degree of explanation: 99.96%

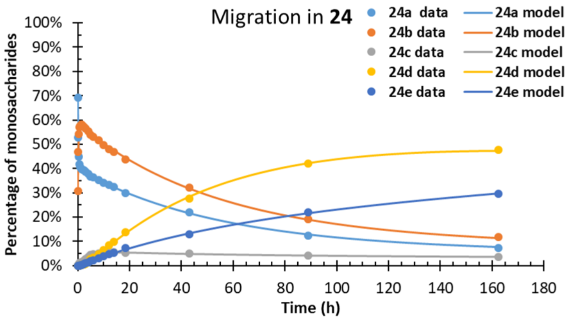

Figure S24. The experimental data and the kinetic model of the migration in **24**. Degree of explanation: 99.98%

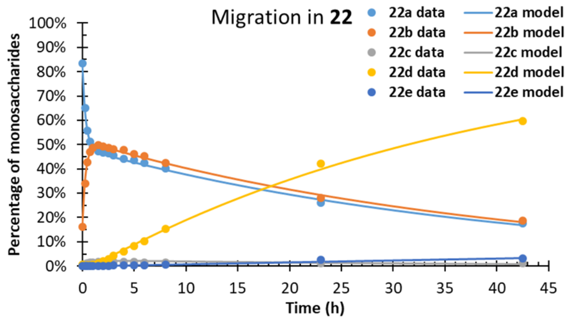

Figure S22. The experimental data and the kinetic model of the migration in **22**. Degree of explanation: 99.90%

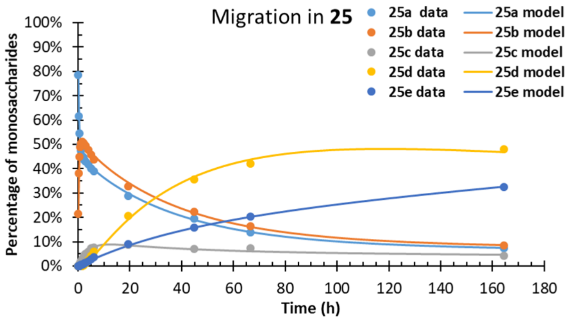

Figure S25. The experimental data and the kinetic model of the migration in **25**. Degree of explanation: 99.92%

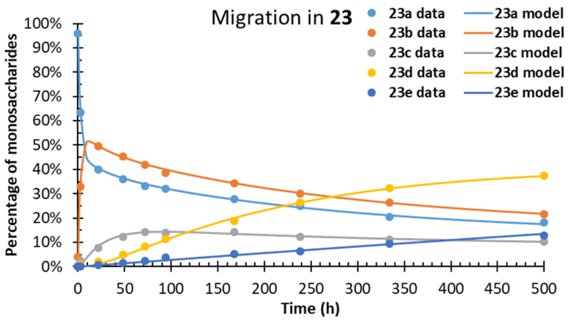

Figure S23. The experimental data and the kinetic model of the migration in **23**. Degree of explanation: 99.81%

Xylopyranoside derivatives

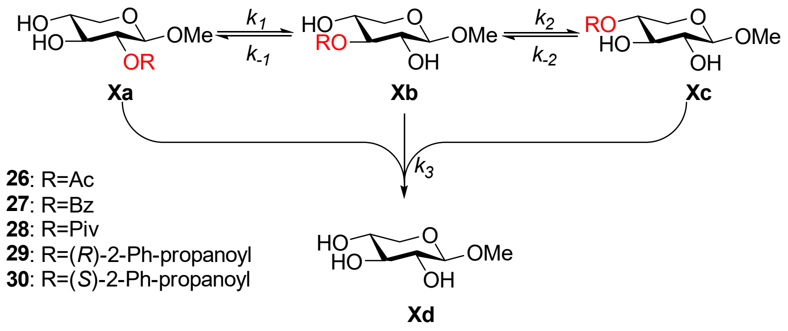

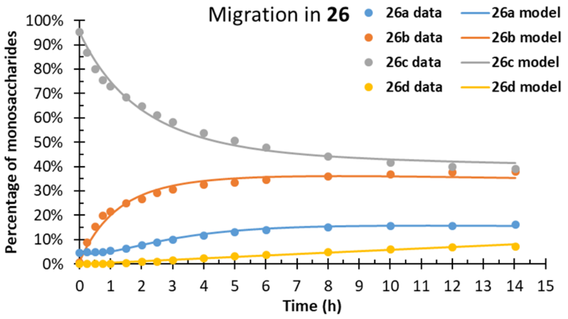

Figure S26. The experimental data and the kinetic model of the migration in **26**. Degree of explanation: 99.64%

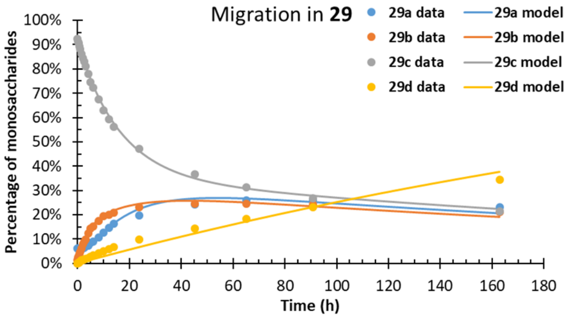

Figure S29. The experimental data and the kinetic model of the migration in **29**. Degree of explanation: 99.82%

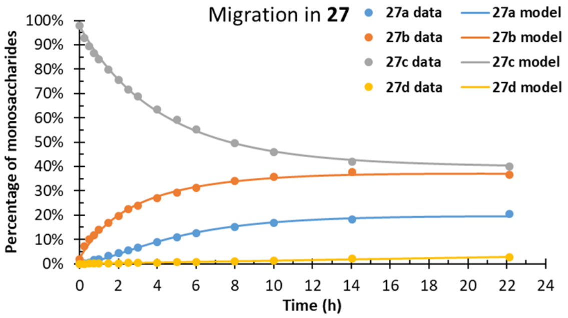

Figure S27. The experimental data and the kinetic model of the migration in **27**. Degree of explanation: 99.94%

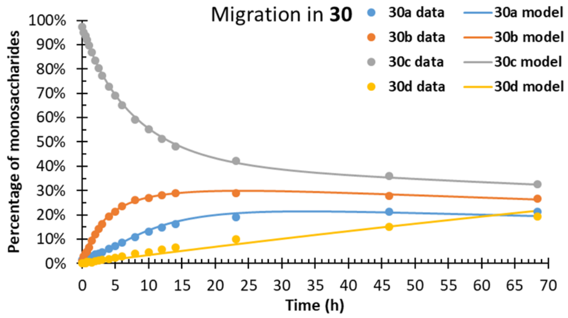

Figure S30. The experimental data and the kinetic model of the migration in **30**. Degree of explanation: 99.91%.

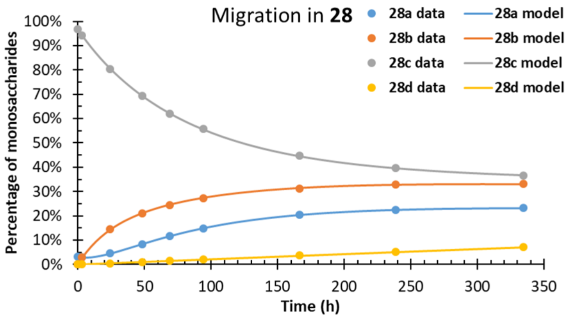

Figure S28. The experimental data and the kinetic model of the migration in **28**. Degree of explanation: 100.00%

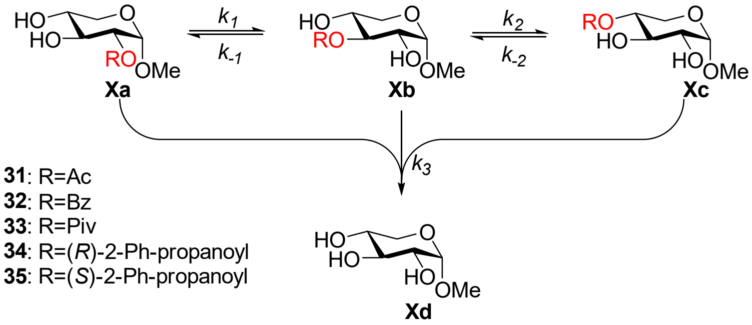

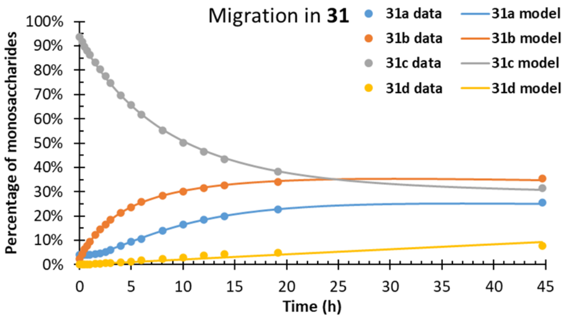

Figure S31. The experimental data and the kinetic model of the migration in **31**. Degree of explanation: 99.98%.

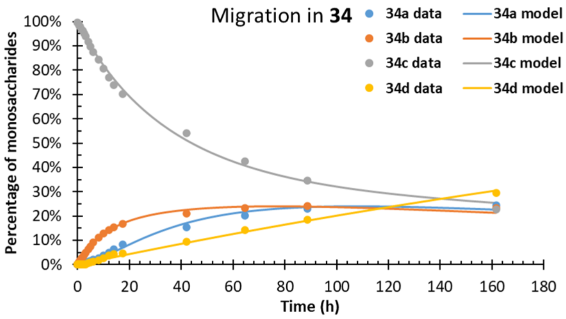

Figure S34. The experimental data and the kinetic model of the migration in **34**. Degree of explanation: 99.93%

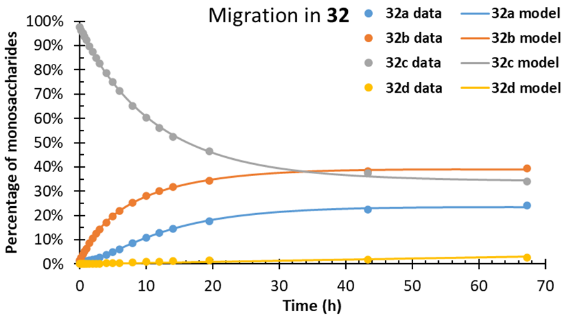

Figure S32. The experimental data and the kinetic model of the migration in **32**. Degree of explanation: 99.99%

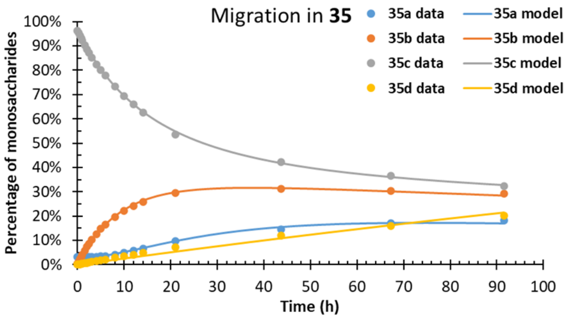

Figure S35. The experimental data and the kinetic model of the migration in **35**. Degree of explanation: 99.97%

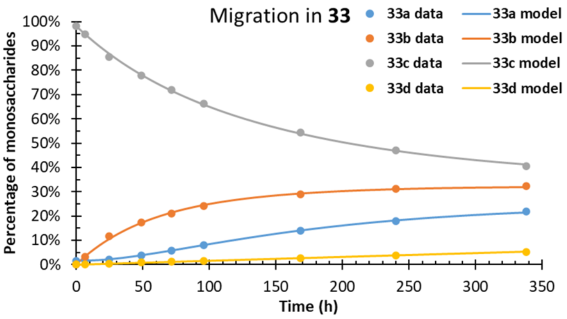

Figure S33. The experimental data and the kinetic model of the migration in **33**. Degree of explanation: 99.98%

Ribopyranoside derivatives

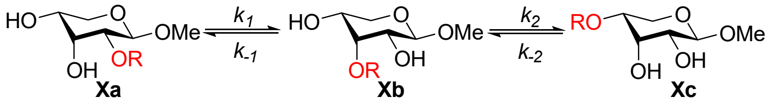

36: R=Ac
37: R=Bz
38: R=Piv
39: R=(*R*)-2-Ph-propanoyl
40: R=(*S*)-2-Ph-propanoyl

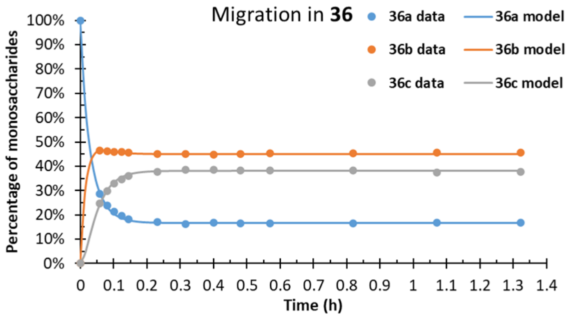

Figure S33. The experimental data and the kinetic model of the migration in **33**. Degree of explanation: 99.96%.

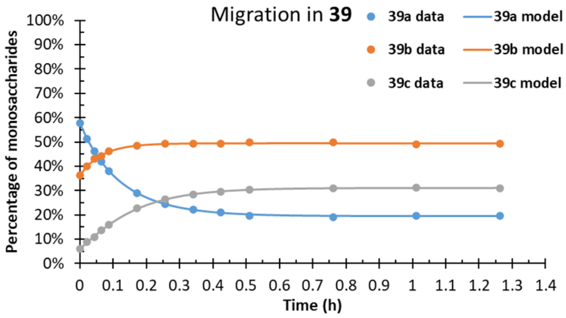

Figure S33. The experimental data and the kinetic model of the migration in **33**. Degree of explanation: 99.99%

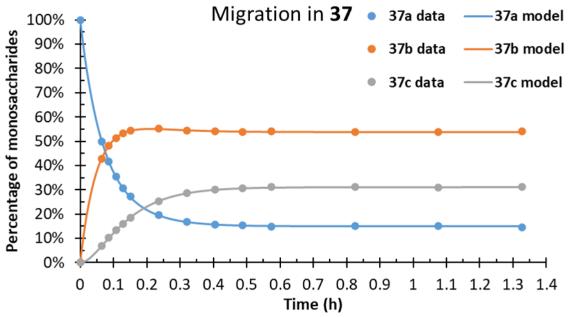

Figure S33. The experimental data and the kinetic model of the migration in **33**. Degree of explanation: 99.99%.

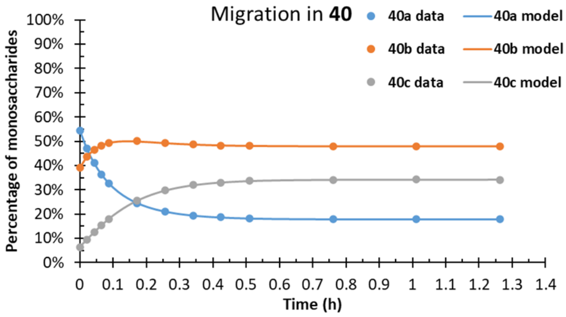

Figure S33. The experimental data and the kinetic model of the migration in **33**. Degree of explanation: 100.00%

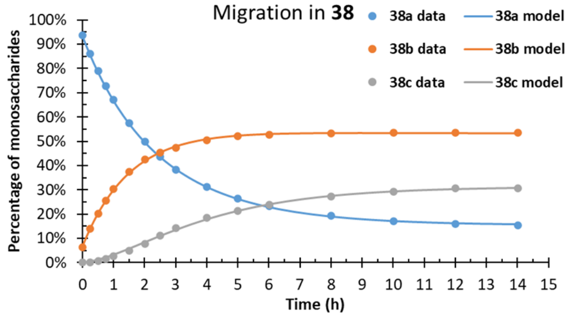

Figure S33. The experimental data and the kinetic model of the migration in **33**. Degree of explanation: 99.98%.

Arabinopyranoside derivatives

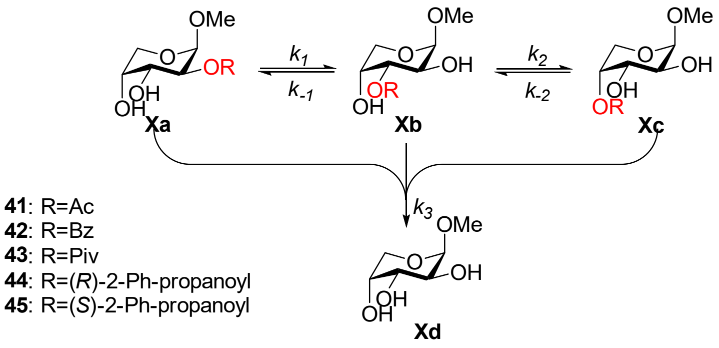

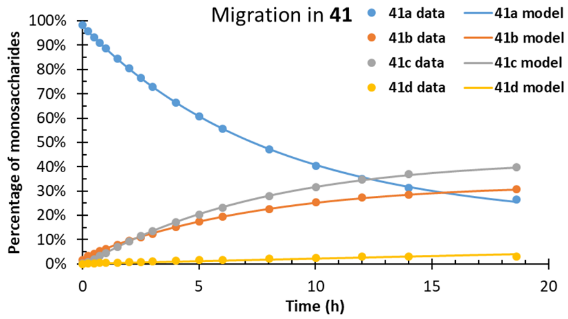

Figure S41. The experimental data and the kinetic model of the migration in **41**. Degree of explanation: 99.99%.

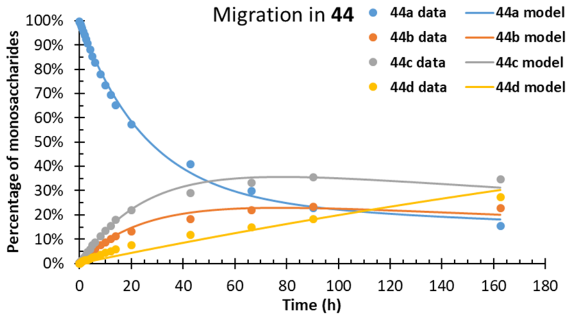

Figure S44. The experimental data and the kinetic model of the migration in **44**. Degree of explanation: 99.82%.

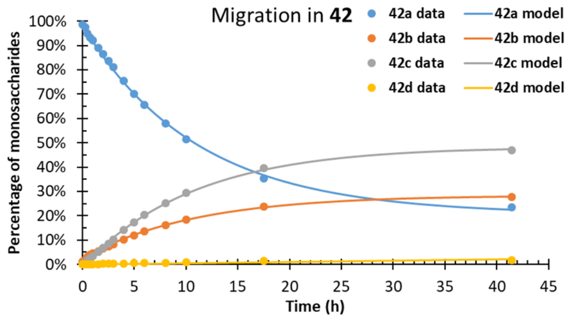

Figure S42. The experimental data and the kinetic model of the migration in **42**. Degree of explanation: 99.98%.

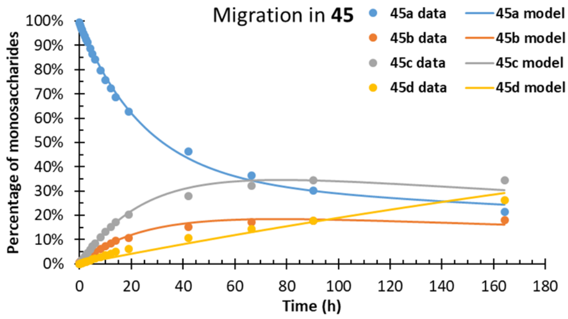

Figure S45. The experimental data and the kinetic model of the migration in **45**. Degree of explanation: 99.87%.

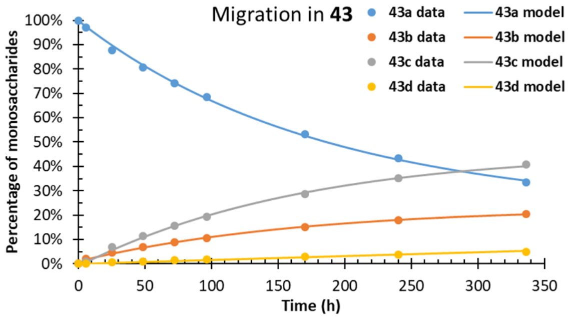

Figure S43. The experimental data and the kinetic model of the migration in **43**. Degree of explanation: 99.95%.

Rhamnopyranoside derivatives

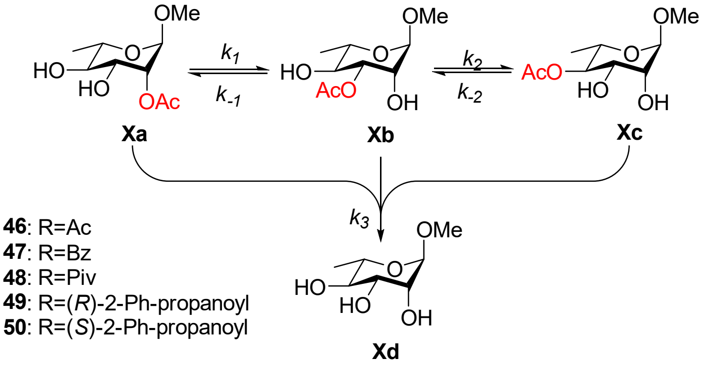

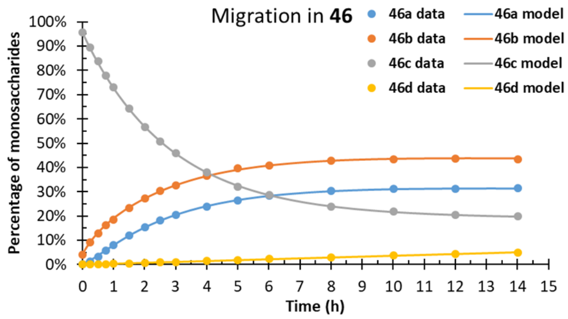

Figure S46. The experimental data and the kinetic model of the migration in **46**. Degree of explanation: 99.99%.

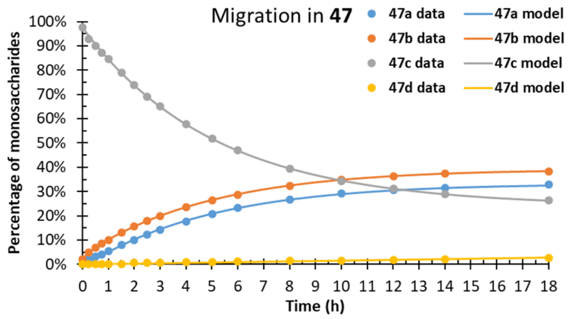

Figure S47. The experimental data and the kinetic model of the migration in **47**. Degree of explanation: 99.98%.

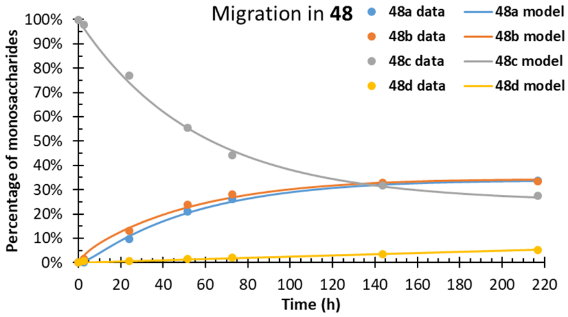

Figure S48. The experimental data and the kinetic model of the migration in **48**. Degree of explanation: 99.83%.

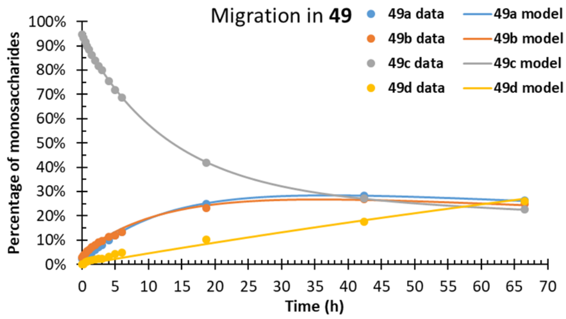

Figure S49. The experimental data and the kinetic model of the migration in **49**. Degree of explanation: 99.97%.

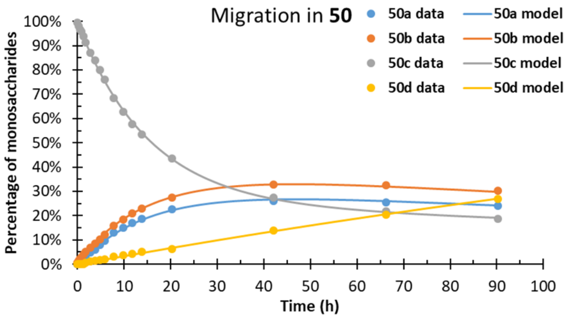

Figure S50. The experimental data and the kinetic model of the migration in **50**. Degree of explanation: 99.98%.

Fucopyranoside derivatives

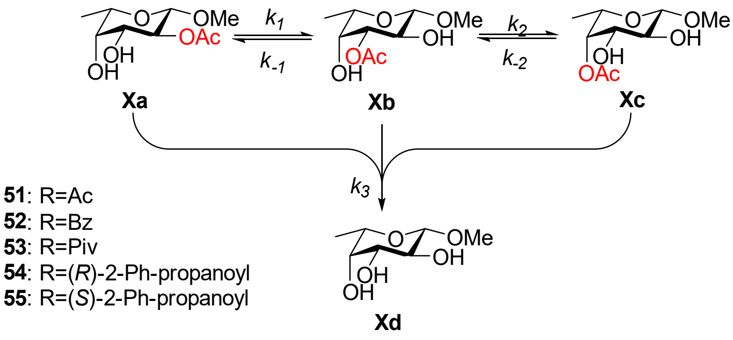

51: R=Ac
52: R=Bz
53: R=Piv
54: R=(*R*)-2-Ph-propanoyl
55: R=(*S*)-2-Ph-propanoyl

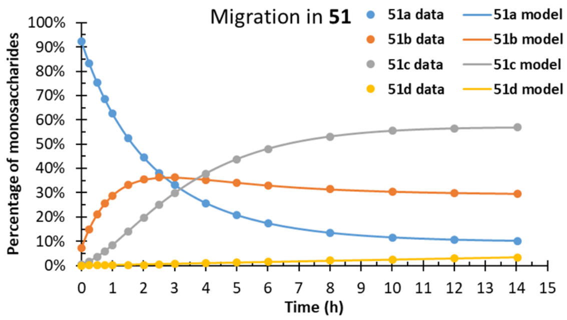

Figure S51. The experimental data and the kinetic model of the migration in **51**. Degree of explanation: 100.00%

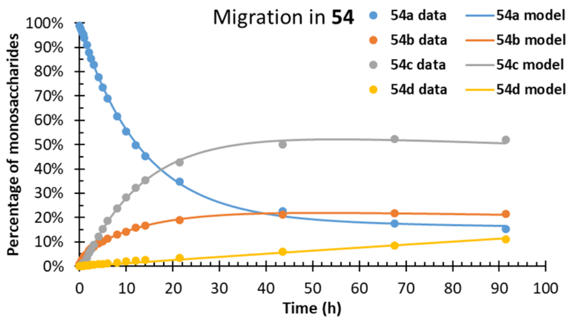

Figure S54. The experimental data and the kinetic model of the migration in **54**. Degree of explanation: 99.96%

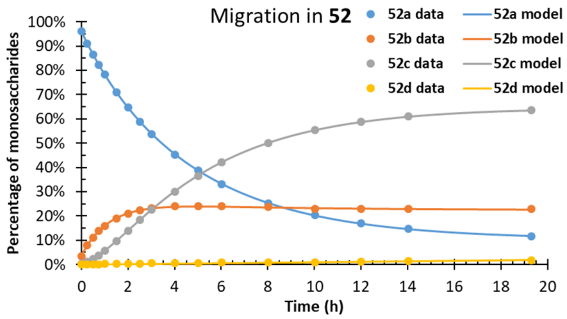

Figure S52. The experimental data and the kinetic model of the migration in **52**. Degree of explanation: 100.00%

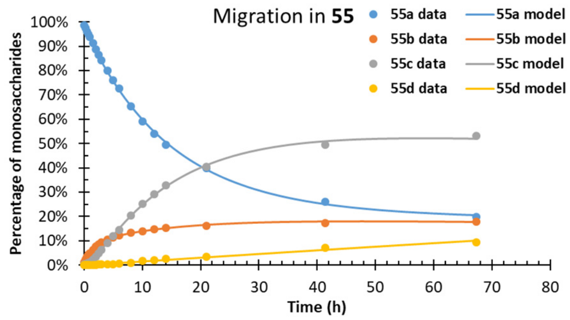

Figure S55. The experimental data and the kinetic model of the migration in **55**. Degree of explanation: 99.98%

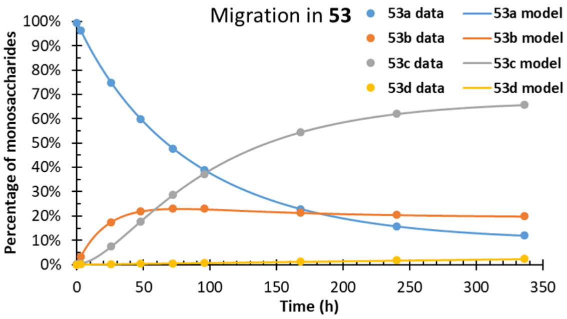

Figure S53. The experimental data and the kinetic model of the migration in **53**. Degree of explanation: 100.00%

Computational Studies

General Methods

All of the calculations were performed using the Gaussian09 program.[12] Preliminary computations on geometry optimizations were done with TPPSh[13,14] functional considering gd3bj Grimme's correction[15] and basis sets 6-31+G(d,p)[16,17] and def2spvd.[18,19] Solvent effects in these preliminary models were considered using the CPCM model.[20,21] In order to compare several levels of theory with experimental results (benchmarking) all geometry optimizations were further carried out with HF and DFT methods wb97xd[22] and m062x[23] functional in conjunction with standard basis set 6-31+G(d,p)[16,17] and SMD solvent model.[24] Single point calculations combining functionals wb97xd[22] and m062x[23] with basis sets 6-311++G(d,p),[16,17] def2tzvp, def2tzvppd[18,19] and cc-pvtz[25,26] were carried out over optimized geometries to obtain more accurate energy values; in particular the used levels for benchmarking were

1. TPPSh/6-311++G(d,p)/smd=water//TPPSh/6-31+G(d,p)/smd=water
2. HF/6-311++G(d,p)/smd=water//HF/6-31+G(d,p)/smd=water
3. wb97xd/6-311++G(d,p)/smd=water//wb97xd/6-31+G(d,p)/smd=water
4. wb97xd/def2tzpv/smd=water//wb97xd/6-31+G(d,p)/smd=water
5. wb97xd/def2tzvpp/smd=water//wb97xd/6-31+G(d,p)/smd=water
6. m062x/6-311++G(d,p)/smd=water//m062x/6-31+G(d,p)/smd=water
7. m062x/cc-pvtz/smd=water//m062x/6-31+G(d,p)/smd=water

The nature of stationary points was defined on the basis of calculations of normal vibrational frequencies (force constant Hessian matrix). The optimizations were carried out using the Berny analytical gradient optimization method.[27] Minimum energy pathways for the reactions studied were found by gradient descent of transition states in the forward and backward direction of the transition vector (IRC analysis).[28] Analytical second derivatives of the energy were calculated to classify the nature of every stationary point, to determine the harmonic vibrational frequencies, and to provide zero-point vibrational energy corrections. The thermal and entropic contributions to the free energies were also obtained from the vibrational frequency calculations, using the unscaled frequencies. Free energy calculations were calculated using a (99,590) grid to minimize errors.[29]

Since the observed process consists of the acyl migration from the corresponding monosaccharide in its neutral form, formal direct $\Delta G_{1(obs)}$ and inverse $\Delta G_{-1(obs)}$ energy barriers in kcal/mol, have been obtained from the observed rate constants $k_{1(obs)}$ and $k_{--1(obs)}$ using the Eyring's equation (eq 1).

$$k_T = \frac{k_B T}{hc^o} e^{\frac{-\Delta G^0}{RT}} \tag{1}$$

where k_T: rate constant in s^{-1}. T: temperature in K. k_B: Boltzman's constant ($1.380662 \cdot 10^{-23}$ $J \cdot K^{-1}$). h: Planck's constant ($6.626176 \cdot 10^{-34}$ $J \cdot s^{-1}$). c^0: concentration. R: gas constant (0.001987 $kcal \cdot mol^{-1} \cdot K^{-1}$). ΔG^0: energy barrier in $kcal \cdot mol^{-1}$

Those values have been compared with the computed formal barriers $\Delta G_{1(calc)}$ and $\Delta G_{-1(calc)}$ which have been calculated assuming that protonation/deprotonation processes are fast; under such conditions, the energy barrier of the process depends on the two steps of the acyl migration. Essentially the same values were obtained by using eq.1 and canonical variational theory,[30,31] including partition coefficients (Eq. 2):

$$k_T = \frac{k_B T}{hc^o} \frac{Q_{TS}}{\Pi Q_R} e^{\frac{-\Delta G^0}{RT}} \tag{2}$$

where k_T: rate constant in s^{-1}. T: temperature in K. k_B: Boltzman's constant ($1.380662 \cdot 10^{-23}$ $J \cdot K^{-1}$). h: Planck's constant ($6.626176 \cdot 10^{-34}$ $J \cdot s^{-1}$). c^0: concentration. R: gas constant (0.001987 $kcal \cdot mol^{-1} \cdot K^{-1}$). ΔG^0: energy barrier in $kcal \cdot mol^{-1}$ Q_{TS}: partition coefficients of transition structure. Q_R: partition coefficients of reactant.

The software eyringpy2[32] was employed for the automatic calculation of rate constants from gaussian output files.

Computational studies with carbohydrates are challenged because of several conformations in a short range of energy can be adopted. We considered several conformations for both minima and transition structures in order to choose those of minimum energy.

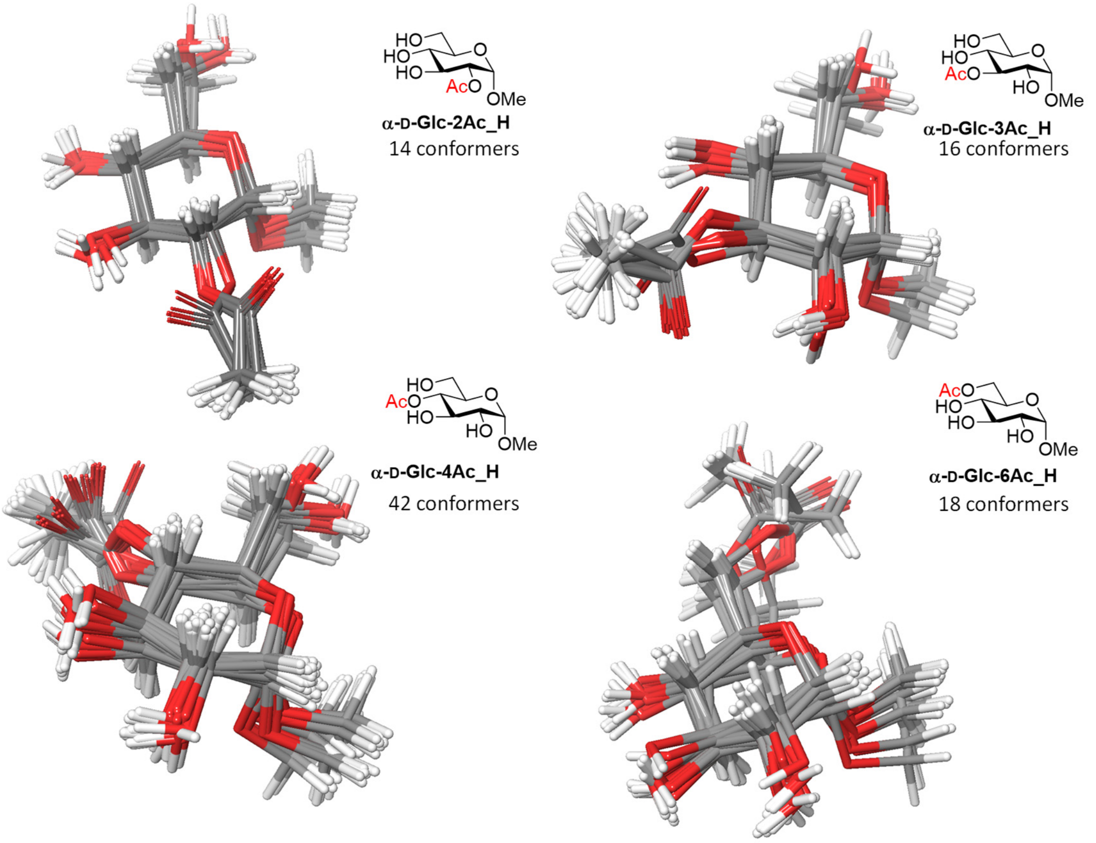

Figure S56. Conformational search for the different acetylated derivatives of Me α-D-glucopyranosides.

The selection of the starting conformations was initiated by a conformational search performed with the software Macromodel as implemented in the Schrödinger package.[33] A mixed torsional/low-mode sampling method with OPLS4 force field and water as a solvent was used. The cutoff for maximum atom deviation was 0.5 and an energy window of 2.0 kcal/mol were selected. The resulting conformers (Figure S56 illustrates the conformational search for acetylated Me D-glucooyranosides) were minimized at level b3lyp-gd3bj/6-31+G(d,p)/smd=water resulting in a lower number of structures. All the converged structures showed the same orientation for the anomeric methoxy group. From these structures those of minimum energy corresponding to representative conformations (i.e.: different rotamers of the acetyl group and C-6-hydroxymethyl group) were selected for further studies.

Kinetics

The analysis of the acyl migration through the different hydroxyl groups requires to consider all the species involved in the process. In principle, two mechanisms are theoretically possible, i.e. (i) a mechanism under neutral or slightly acidic conditions and (ii) a mechanism under basic conditions in which the anion is formed in some extent. Under acidic conditions the hydrolysis prevails over acyl migration, and, in any case, hydrolysis is always observed at any pH. We will illustrate the methodology with acetylated ME α-D-glucopyranoside. The complete process is illustrated in Scheme S3.

The process illustrated in Scheme S3 for Me 2-O-Ac-α-D-glucopyranoside experimentally showed the direct and reverse rate constants indicated in Table S1. Although, rate constants are directly related with energy barriers through Eqs. 1 and 2, discussions are usually preferred to be place on the basis of energy barriers for which DFT calculations of free energies can vary by as much as 5 kcal/mol considering several orientations of the molecule; however by using fine grids is possible to reduce differences around 1 – 2 kcal/mol.[29] When translated to rate constants, increasing 2 kcal/mol the barrier for a constant of 0.878 s^{-1} results in a constant of 0.00315 s^{-1}. This exponential difference makes very difficult to obtain accurate values of rate constants, particularly with carbohydrates that have a high conformational flexibility.

Table S1. Experimental rate constants (s^{-1}) and the corresponding formal energy barriers (kcal·mol^{-1}), calculated through the Eyring's equation for the acetyl migration in Me α-D-glucopyranoside at pH=8 (Scheme S3).

rate constants (s^{-1})		energy barriers (kcal·mol^{-1})	
K_1^{obs}	6.28E-05	ΔG_1	23.2
K_{-1}^{obs}	5.11E-05	ΔG_{-1}	23.3
K_2^{obs}	5.06E-05	ΔG_2	23.3
K_{-2}^{obs}	1.01E-04	ΔG_{-2}	22.9
K_3^{obs}	1.21E-03	ΔG_3	21.4
K_{-3}^{obs}	7.83E-05	ΔG_{-3}	23.1

The observed K_{obs}'s (in blue) are the experimental values obtained from the kinetics. For the calculation of those constants, we consider a fast equilibrium for (de)protonation (acid/base) stages; accordingly, these processes are thus governed by the corresponding K_{eq} (in green)

which can be obtained from the computationally calculated pK_a of the corresponding monosaccharide. The hydrolysis side-reaction is not considered assuming that affects all acylated compounds in a similar extent and that it can be neglected for calculations at pH>7.

The rate equations for compounds **A**-**L** are:

$$\frac{d[A]}{dt} = -(k_1 + k_3)[A] + k_{-1}[B][H^+] + k_{-3}[D] \quad (3)$$

$$\frac{d[B]}{dt} = k_1[A] - k_{-1}[B][H^+] - k_2[B] + k_{-2}[C] \quad (4)$$

$$\frac{d[C]}{dt} = k_2[B] - k_{-2}[C] - k_{-4}[C][H^+] + k_4[D] \quad (5)$$

$$\frac{d[D]}{dt} = k_3[A] + k_{-4}[C][H^+] + k_{-6}[G] + k_{-5}[E][H^+] -$$

$$-(k_4 + k_{-3} + k_5 + k_6)[D] \quad (6)$$

$$\frac{d[E]}{dt} = k_5[D] - k_{-5}[E][H^+] - k_7[E] + k_{-7}[F] \quad (7)$$

$$\frac{d[F]}{dt} = k_7[E] - k_{-7}[F] - k_{-8}[F][H^+] + k_8[G] \quad (8)$$

$$\frac{d[G]}{dt} = k_6[D] + k_{-8}[F][H^+] + k_{-11}[L] + k_{-9}[I][H^+] -$$

$$-(k_8 + k_{-6} + k_9 + k_{11})[G] \quad (9)$$

$$\frac{d[I]}{dt} = k_9[G] - k_{-9}[I][H^+] - k_{10}[I] + k_{-10}[J] \quad (10)$$

$$\frac{d[J]}{dt} = k_{10}[I] - k_{-10}[J] - k_{-12}[J][H^+] + k_{12}[L] \quad (11)$$

$$\frac{d[L]}{dt} = k_{11}[G] + k_{-12}[J][H^+] - (k_{12} + k_{-11})[L] \quad (12)$$

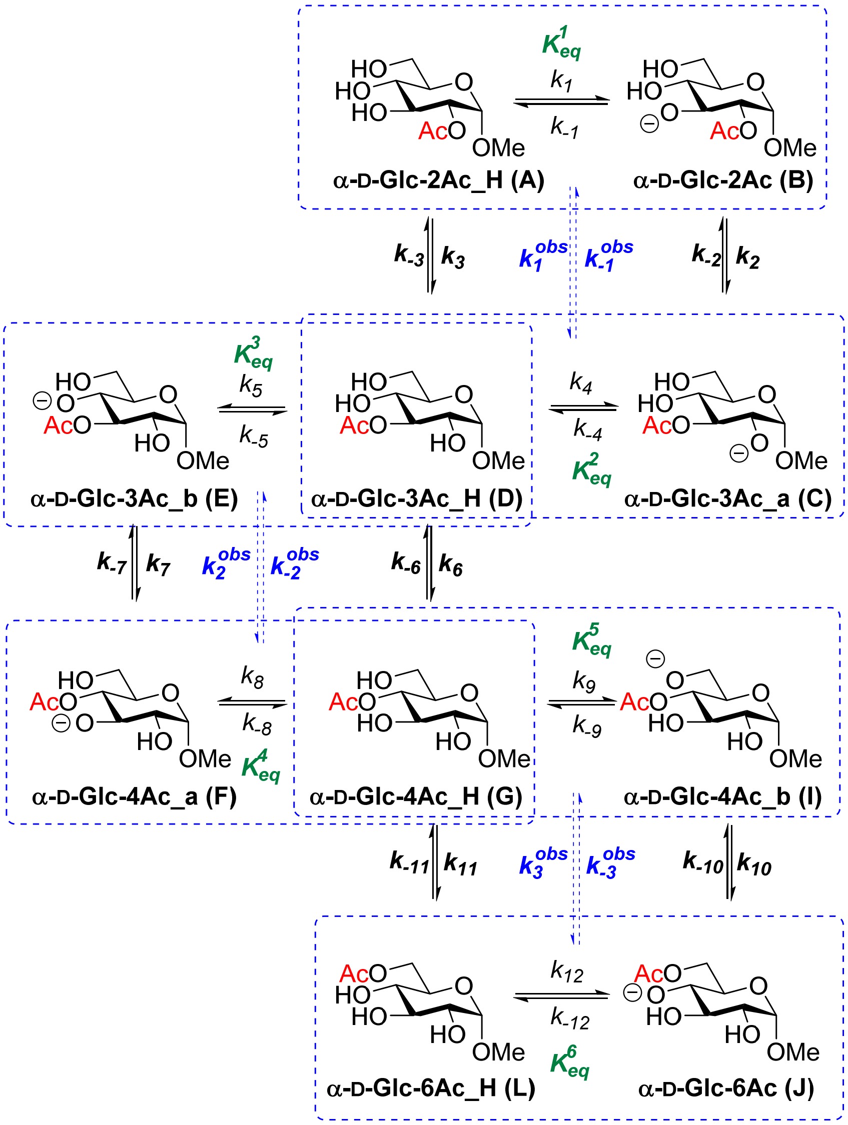

Scheme S3. Acetyl migration in acetylated Me α-D-glucopyranoside. (Labels for compounds H and K have been omitted intentionally to avoid confusion con hydrogen and rate constants in equations)

By considering a fast acid-base equilibrium Eqs. 4, 5, 7, 8, 10 and 11 are equal to zero, according to stationery transition state theory. The equilibrium constants are:

$$K_{eq}^1 = \frac{k_1}{k_{-1}} = \frac{[\mathrm{B}]\cdot[\mathrm{H}^+]}{[\mathrm{A}]} \tag{13}$$

$$K_{eq}^2 = \frac{k_4}{k_{-4}} = \frac{[C][H^+]}{[D]} \tag{14}$$

$$K_{eq}^3 = \frac{k_5}{k_{-5}} = \frac{[E]\cdot[H^+]}{[D]} \tag{15}$$

$$K_{eq}^4 = \frac{k_8}{k_{-8}} = \frac{[F][H^+]}{[G]} \tag{16}$$

$$K_{eq}^5 = \frac{k_9}{k_{-9}} = \frac{[I]\cdot[H^+]}{[G]} \tag{17}$$

$$K_{eq}^6 = \frac{k_{12}}{k_{-12}} = \frac{[J][H^+]}{[L]} \tag{18}$$

Mathematical treatment of the above equations provides the following values for the observed rate constants:

$$K_1^{obs} = -k_3 + \frac{k_2 \cdot K_{eq}^1}{[H]^+} \quad (19)$$

$$K_{-1}^{obs} = -k_{-3} + \frac{k_{-2} \cdot K_{eq}^2}{[H]^+} \quad (20)$$

$$K_2^{obs} = -k_6 + \frac{k_7 \cdot K_{eq}^3}{[H]^+} \quad (21)$$

$$K_{-2}^{obs} = -k_{-6} + \frac{k_{-7} \cdot K_{eq}^4}{[H]^+} \quad (22)$$

$$K_3^{obs} = -k_{11} + \frac{k_{10} \cdot K_{eq}^5}{[H]^+} \quad (23)$$

$$K_{-3}^{obs} = -k_{-11} + \frac{k_{-10} \cdot K_{eq}^6}{[H]^+} \quad (24)$$

Consequently, it is necessary to calculate both neutral and anionic mechanisms, as well as the pK_a of the corresponding hydroxyl group to obtain the equilibrium constant at a given pH (Eq. 25):

$$pK_a^n = -\log K_{eq}^n \qquad (n = 1 - 6) \quad (25)$$

Neutral mechanism

Computational modelling of intramolecular acyl migration has been reported by Nicholson and co-workers[34] at a semiempirical level and, more recently, in nucleobases, by Petrov and co-workers using DFT methods.[35] These studies confirmed the stepwise mechanism through a tetrahedral orthoester intermediate. A typical mechanism involving the participation of one water molecule afforded high barriers of ca. 40 kcal/mol. In the case of α-D-Glucopyranosides (Scheme S4, Path A1 shows the first migration from **α-D-Glc-2AcH**) similar values were obtained (Table S2), clearly too high to assume that this is the only mechanism operating in the reaction.

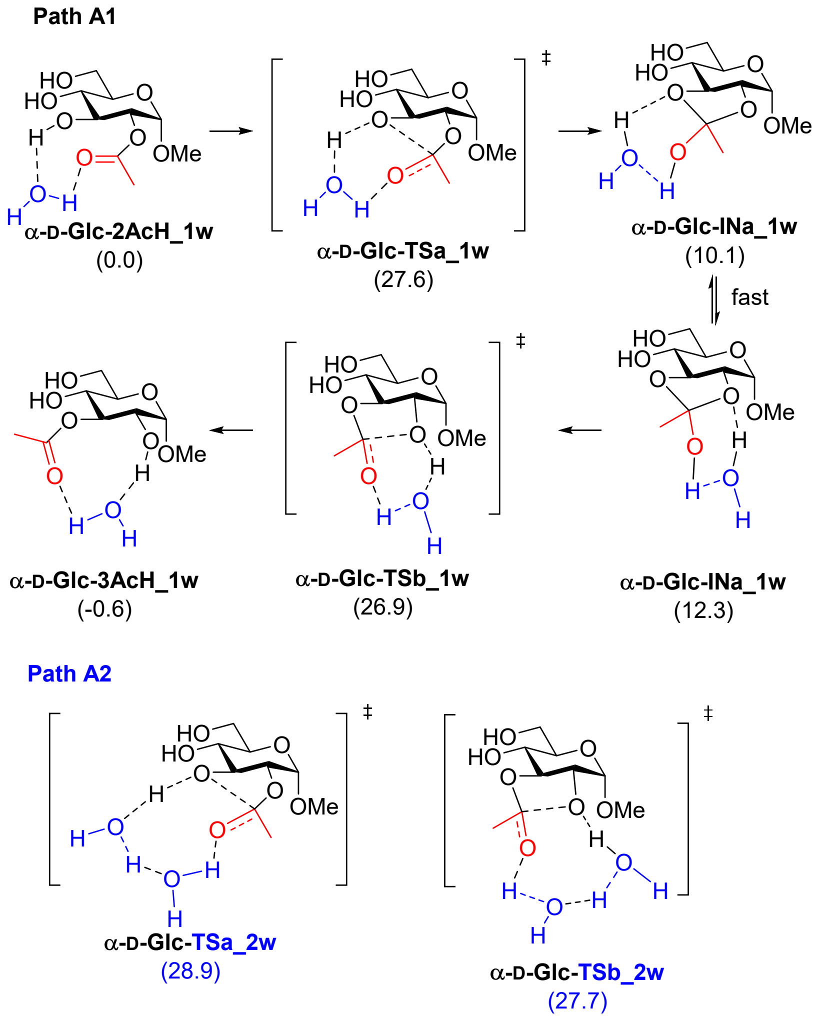

Scheme S4. Neutral mechanisms through water-bridged transition structures

For the purpose of comparison, we calculated the neutral mechanism using four levels of theory (see Table S2). In all cases, too high energy barriers (ca. 30 kcal/mmol) were observed. These values correspond to rate constants in the range of 10^{-10}-10^{-14} s^{-1}, extremely low when compared with the observed experimental values.

Table S2. Calculated free energy barriers (kcal·mol^{-1}) for the acetyl migration in Me α-D-glucopyranoside at pH=8 under a neutral mechanism involving one water molecule.[a] (Scheme S3).

	exp	calc[c]	calc[d]	calc[e]	calc[f]
ΔG_3	23.2	29.0	34.9	31.1	30.2
ΔG_{-3}	23.3	28.8	34.8	32.3	31.8
ΔG_6	23.3	34.0	40.6	36.8	36.3
ΔG_{-6}	22.9	27.8	37.9	32.9	32.3
ΔG_{11}	21.4	29.2	35.7	31.4	30.9
ΔG_{-11}	23.1	29.0	37.7	30.7	29.7

[a] Referred to Scheme S3. [b] The subindex refers to the corresponding rate constant in Scheme S4. [c] TPPSh/6-311++G(d,p)/smd=water //TPPSh/6-31+G(d,p)/smd=water . [d] wb97xd/6-311++G(d,p)/ smd=water //wb97xd/6-31+G(d,p)/smd=water. [e] m062x/6-311++G(d,p)/smd=water //m062x/6-31+G(d,p)/smd=water. [f] m062x/cc-pvtz/smd=water //m062x/6-31+G(d,p)/smd=water.

Table S3. Calculated rate constants (s^{-1}) for the acetyl migration in Me α-D-glucopyranoside at pH=8 under a neutral mechanism involving one water molecule.[a] (Scheme S3).

	exp	calc[c,g]	calc[d,g]	calc[e,g]	calc[f,g]
K_1^{obs}	6.28E-05	3.57E-09	1.64E-13	1.06E-10	4.76E-10
K_{-1}^{obs}	5.11E-05	5.01E-09	2.14E-13	1.28E-11	3.06E-11
K_2^{obs}	5.06E-05	7.38E-13	1.07E-17	7.59E-15	1.56E-14
K_{-2}^{obs}	1.01E-04	2.59E-08	1.05E-15	5.19E-12	1.28E-11
K_3^{obs}	1.21E-03	2.60E-09	4.53E-14	5.86E-11	1.54E-10
K_{-3}^{obs}	7.83E-05	3.64E-09	1.62E-15	2.20E-10	1.07E-09

[a] Referred to Scheme S3. [b] The subindex refers to the corresponding rate constant in Scheme S4. [c] TPPSh/6-311++G(d,p)/smd=water //TPPSh/6-31+G(d,p)/smd=water . [d] wb97xd/6-311++G(d,p)/ smd=water //wb97xd/6-31+G(d,p)/smd=water. [e] m062x/6-311++G(d,p)/smd=water //m062x/6-31+G(d,p)/smd=water. [f] m062x/cc-pvtz/smd=water //m062x/6-31+G(d,p)/smd=water. [g] Obtained from the calculated barriers through the Eyring's equation

We extended the studies to an additional water molecule, calculating the model with a bridge of two water molecules (Scheme S4, Path A2, only transition structures are shown) providing the system with more flexibility. Nevertheless, for the first migration step, similar values were observed so, we consider path A1 as a typical acyl migration mechanism under neutral conditions.

Anionic mechanism

The possibility of an initial deprotonation and further attack of the alkoxide anion, had been suggested by Petrov and co-workers by considering the reaction of an ionized vicinal hydroxyl group.[35] However, these authors indicated that no transition structures corresponding to the nucleophilic attack of an alkoxide on a carbonyl group could be located concluding that acyl group migration proceeds spontaneously when the vicinal hydroxyl group is deprotonated. On the contrary, in the case of α-D-glucopyranoside (Scheme S5, Path B, showing only the first migration from **α-D-Glc-2AcH**), we have no trouble in locating the corresponding transition structures for the two steps of the acyl migration.

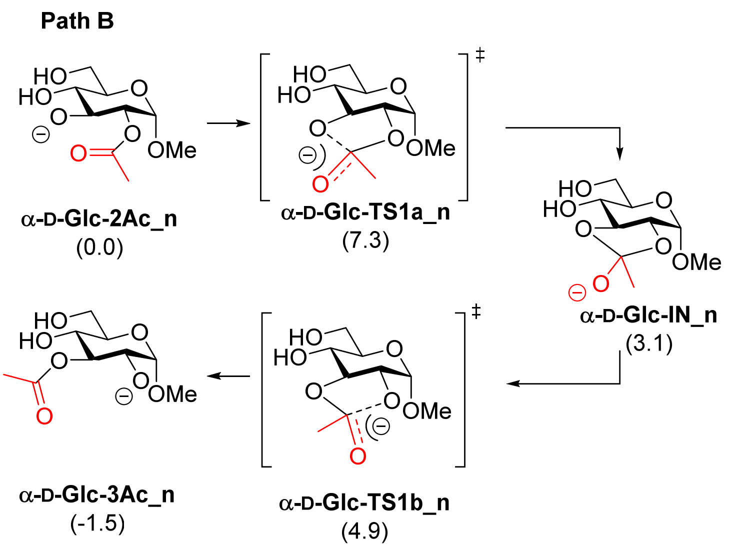

Scheme S5. Acyl migration in a naked anion

Table S4. Calculated (TPPSh/6-311++G(d,p)/smd=water //TPPSh/6-31+G(d,p)/smd=water) free energy barriers (kcal·mol^{-1}) and rate constants (s^{-1}) for the acetyl migration in Me α-D-glucopyranoside at pH=8 under an anionic mechanism.[a] (Scheme S3).

calculated energy barriers (kcal·mol^{-1})[b]		calculated rate constants (s^{-1})	
ΔG_2	7.3	k_2	2.72E+07
ΔG_{-2}	8.7	k_{-2}	2.56E+06
ΔG_7	7.4	k_7	2.20E+07
ΔG_{-7}	8.7	k_{-7}	2.45E+06
ΔG_{10}	8.4	k_{10}	4.31E+06
ΔG_{-10}	10.8	k_{-10}	7.49E+04

[a] Referred to Scheme S3. [b] The subindex refers to the corresponding rate constant in Scheme S4
[c] Obtained from the calculated barriers through the Eyring's equation

The calculated barriers for the three acyl migrations are given in Table S4 indicating that the process is not spontaneous, but far away from the experimentally observed barriers.

At this point, it is necessary to make an **analysis of the kinetics of the reaction**. As we have indicated above (Eqs. 19-24), the rate constant for a given migration can be represented in general terms as in Eq. 26:

$$K_{-i}^{obs} = -k_{neutral} + \frac{k_{anionic} \cdot K_{eq}}{[H]^{+}} \qquad (26)$$

where $K_{-i}^{obs} \approx 10^{-5}$. According to the calculated values, $k_{neutral} \approx 10^{-9}$ (see Table S2) and it can be considered negligible at pH>6 for the contribution to K_{-i}^{obs}. On the other hand, $k_{anionic} \approx 10^{6}$ (see Table 3) so, at pH = 8 ($[H^+] = 10^{-8}$) we need values of $K_{eq} \approx 10^{-19}$ corresponding to pK_a values ca. 19 – 20, clearly higher that those expected for a carbohydrate close to 12 for the anomeric hydroxyl and 13 – 14 for the other less acidic hydroxyl groups.[36] Moreover, the presence of an acetyl group is expected to reduce the pK_a value of adjacent hydroxyl groups in one or two units considering that we are in an aqueous medium. By considering a standard value of pK_a = 12, the expected value for the rate constants of the anionic mechanism should be $k_{anionic} \approx 10^{-1}$ corresponding to barriers of *ca.* 20 – 24 kcal·mol^{-1}.

By considering that the reaction is carried out in water and, under such conditions, a naked anion in a polar continuum medium might not represent precisely the real situation we introduced discrete molecules of water surrounding the alkoxide anion. In this context, it had been reported for the calculation of pK_a's of thiols that the presence of three explicit molecules of water is required for a correct description of the model.[37] We applied the same model by placing three explicit water molecules in a tetrahedral orientation surrounding the anion simulating interaction with the three lone pairs of the anion. In order to start from a suitable conformation and select the location of the three explicit ester molecules we carried out simple molecular dynamics simulations (MD)[1] for the corresponding anions in a 12 Å^3 box of water molecules (as an example, Figure S57 shows the 3-anion from Me 2-*O*-Ac-D-α-glucopyranoside immersed in the box of water) what allow identifying starting positions of explicit water molecules to be used in QM calculations. In addition, three different rotations of the C-O bond (Figure S57, B) were calculated to select a point of minimum energy always ensuring the correct interaction of the three water molecules.

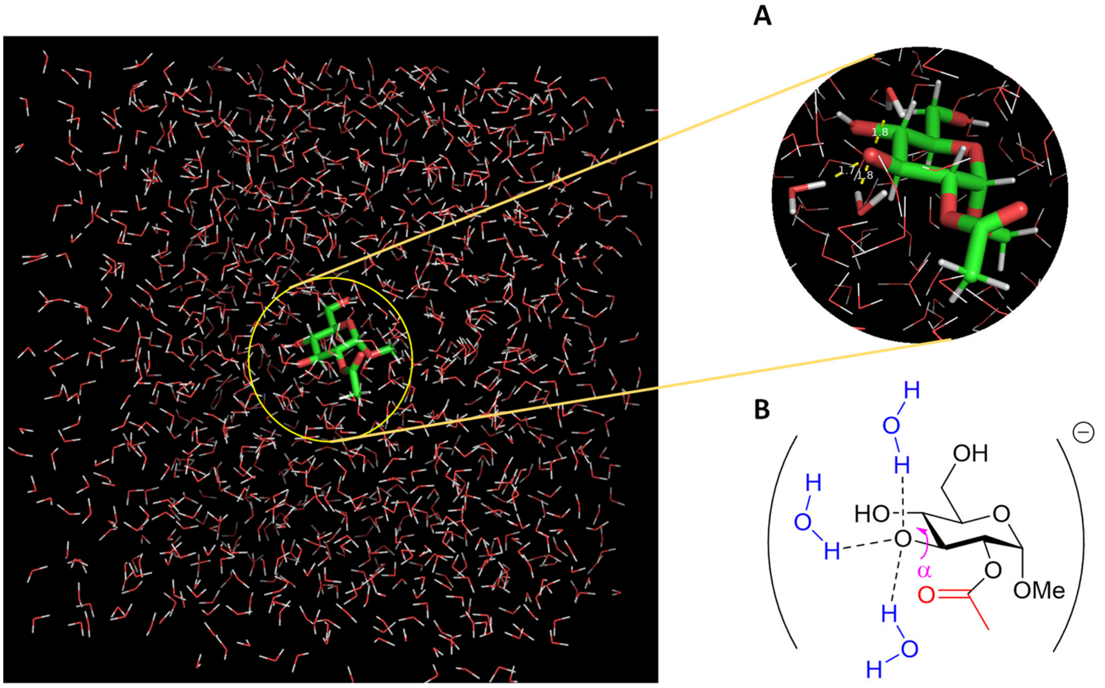

Figure S57. Molecular dynamics of the 3-anion from Me 2-*O*-Ac-D-α-glucopyranoside immersed in the a 12 Å^3 box of water (Na cation has been omitted for clarity). **A:** Detail of the surrounding water molecules after 100 ns. **B**: Model used for calculations (α indicates rotation of C-O bond; see text)

The importance of microsolvation in locating transition structures accurately has also been demonstrated recently.[38] At this point we decide to use the recommended levels of theory

---

[1] MD simulations were carried out with AMBER20 suite of programs.[45] Parameters were generated with the antechamber module using the general Amber force field (GAFF2) with partial charges calculated using AM1-BCC method. The carbohydrate was immersed in a water box with a 12 Å buffer of TIP3P[1] water molecules. After minimization, heating to 300 K and equilibration a production MD of 100 ns was carried out.

for this sort of calculations with thiols,[37,39] i.e.: m062x and wb97xd functionals with 6-31+G(d,p) and 6-311++G(d,p) basis sets for optimizations and single point calculations, respectively. In all cases, several orientations of, mainly, the acetyl group and the hydroxymethyl group were calculated. All the discussions will be based on the structures showing the minimum energy. The SMD solvent model for water was always used. We calculated the acyl migration for **α-D-Glc-2Ac** by introducing one, two and three explicit molecules of water. Noteworthy, the more water molecules were introduced the higher were the barriers with respect to the naked anion (Scheme S4), and with three water molecules (Scheme 6), the barriers were found to be 18.4 and 18.3 kcal/mol for the direct and inverse barriers, respectively, which are more adequate values to those expected according to kinetics. Including additional molecules of water interacting with the carbonyl group or other vicinal groups, resulted in lower barriers (with four molecules of water, we obtained 10.6 and 10.4 kcal/mol for direct and inverse barriers, respectively, and with 6 molecules of water the values were 15.0 and 12.7 kcal/mol).

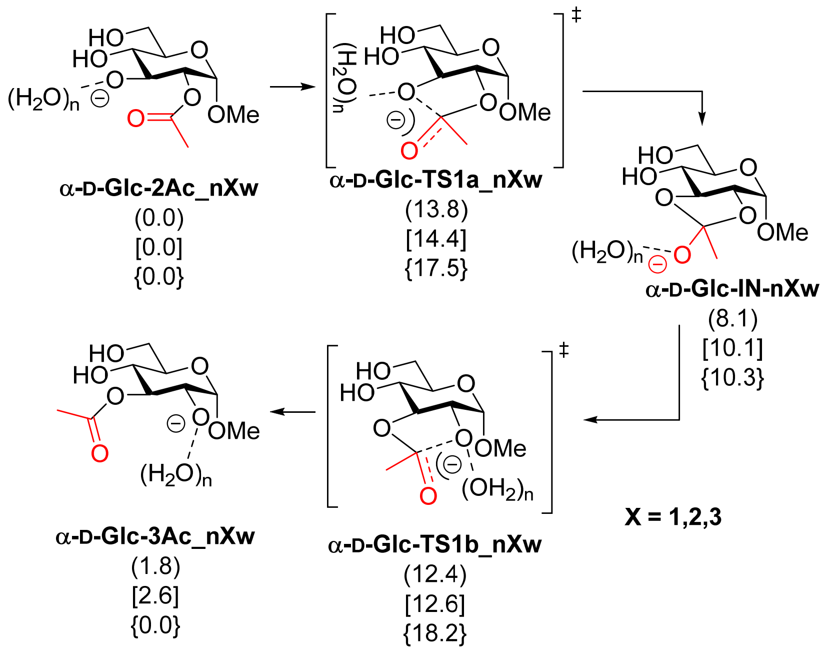

Scheme S6. Models incorporating explicit water molecules. Relative energies (wb97xd/6-311++G(d,p)/ smd=water //wb97xd/6-31+G(d,p)/smd=water) are given in kcal/mol for one (between parentheses), two (between brackets) and three (between curly brackets) molecules of water

Consequently, we decided to study all the migrations by including three molecules of water explicitly at the anionic site. We studied the migration from position 2 to position 3, then from position 3 to position 4 and finally from position 4 to position 6. The results are collected in Table S4 for the different levels of theory studied with the aim of comparing the different results with the experimental values observed after the complete analysis. Table S5 collects the corresponding energy barriers from which the rate constants listed in Table S6 have been calculated.

Table S5. Calculated free energy barriers (kcal·mol^{-1}) for the acetyl migration in Me α-D-glucopyranoside at pH=8 under an anionic mechanism considering three explicit molecules of water.[a] (Scheme S3).

	level 1	level 2	level 3	level 4	level 5
ΔG_2	19.7	20.1	20.0	14.8	14.8
ΔG_{-2}	19.5	16.0	16.0	16.4	16.3
ΔG_7	15.5	15.7	18.6	16.9	17.3
ΔG_{-7}	19.8	17.5	17.5	18.6	19.1
ΔG_{10}	17.6	17.6	17.9	14.8	14.3
ΔG_{-10}	17.8	17.0	17.2	17.5	18.0

[a] Referred to Scheme S3. [b] The subindex refers to the corresponding rate constant in Scheme S4.
level 1: wb97xd/6-311++G(d,p)/smd=water //wb97xd/6-31+G(d,p)/smd=water.
level 2: wb97xd/def2tzpv/smd=water //wb97xd/6-31+G(d,p)/smd=water.
level 3: wb97xd/def2tzvpp/smd=water //wb97xd/6-31+G(d,p)/smd=water
level 4: m062x/6-311++G(d,p)/smd=water //m062x/6-31+G(d,p)/smd=water
level 5: m062x/cc-pvtz/smd=water //m062x/6-31+G(d,p)/smd=water

Table S6. Calculated rate constants (s^{-1}), obtained from the calculated barriers through the Eyring's equation, for the acetyl migration in Me α-D-glucopyranoside at pH=8 under an anionic mechanism considering three explicit molecules of water.[a] (Scheme S3).

	level 1	level 2	level 3	level 4	level 5
k_2	2.29E-02	1.22E-02	1.46E-02	8.67E+01	8.80E+01
k_{-2}	3.01E-02	1.14E+01	1.22E+01	6.24E+00	6.82E+00
k_7	2.91E+01	2.04E+01	1.52E-01	2.76E+00	1.42E+00
k_{-7}	1.83E-02	9.88E-01	9.67E-01	1.60E-01	6.49E-02
k_{10}	8.57E-01	8.19E-01	5.23E-01	8.50E+01	2.19E+02
k_{-10}	5.81E-01	2.16E+00	1.70E+00	8.92E-01	4.01E-01

[a] Referred to Scheme S3. [b] The subindex refers to the corresponding rate constant in Scheme S4.
level 1: wb97xd/6-311++G(d,p)/smd=water //wb97xd/6-31+G(d,p)/smd=water.
level 2: wb97xd/def2tzpv/smd=water //wb97xd/6-31+G(d,p)/smd=water.
level 3: wb97xd/def2tzvppd/smd=water //wb97xd/6-31+G(d,p)/smd=water
level 4: m062x/6-311++G(d,p)/smd=water //m062x/6-31+G(d,p)/smd=water
level 5: m062x/cc-pvtz/smd=water //m062x/6-31+G(d,p)/smd=water

pK_a calculations

As in the case of rate constants, calculations of accurate pK_a' values is challenging because small variations in energy result in several units of difference (a difference of 1 unit of pK_a only needs of an error of 1.36 kcal/mol).[40] Even with accurate levels of theory differences of 1 – 2 units could be expected.[41]

For calculating pK_a values we used the same model employed for the anionic model, consisting of three explicit molecules of water in a continuum of water as a solvent. Models without considering explicit molecules of water gave in all cases (up to 6 levels checked including CBS--QB3) pKa's ca. 20. We carried out preliminary studies with Me α-D-glucopyranoside and directly considered the difference between solvated states (Eq. 27). Consideration of the classical cycle involving gas and solvated species did not afford better results.

$$\Delta G^*_{aq} = G^*_{aq}(A^-) + G^*_{aq}(H^+) - G^*_{aq}(AH) + \Delta G^{1atm\rightarrow 1M} \quad (27)$$

where the aqueous phase proton free energy is -265.9 kcal/mol according to the literature[42–44] and the free energy change due to changing the standard state from 1 atm to 1 M is 1.89 kcal/mol (Eq. 28).

$$\Delta G^{1atm\rightarrow 1M} = RTln(24.46) = 1.89\ kcal/mol \quad (28)$$

Further application of Eq. 29 provided the pK_a value.

$$pK_a = \frac{\Delta G^*_{aq}}{2.303RT} \quad (29)$$

Again, we optimized geometries with m062x and wb97xd functionals, 6-31+G(d,p) and 6-311++G(d,p) basis sets, as well as additional levels reported above, and SMD solvent model for water according to previous studies with thiols[37,39] and to keep coherence in the calculations. Levels considering three explicit water molecules gave coherent values with those that could be expected for a carbohydrate.

Table S7. Calculated pK_a values (Eq. 27-29) for the different species involved in acetyl migration of Me α-D-glucopyranoside.

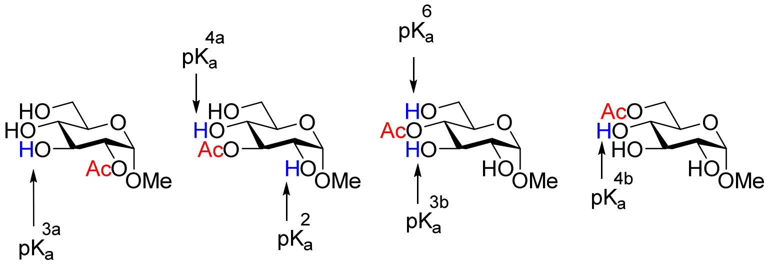

α-D-Glc-2Ac_H α-D-Glc-3Ac_H α-D-Glc-4Ac_H α-D-Glc-6Ac_H

	L1[a,b]	L2[a,c]	L3[a,d]	L4[e,f]	L5[e,d]	L6[e,g]	L7[e,h]	L8[e,i]	L9[e,j]
pK_a^{3a}	24.8	26.4	21.5	23.3	12.2	13.1	13.3	12.9	13.4
pK_a^{2}	n.d.[k]	27.4	20.3	21.4	10.4	14.3	14.5	12.0	13.2
pK_a^{4a}	n.d.[k]	27.9	20.8	19.1	13.1	14.7	12.9	12.6	13.7
pK_a^{3b}	n.d.[k]	24.4	20.5	23.1	11.7	15.1	15.2	11.0	12.2
pK_a^{6}	n.d.[k]	27.7	22.4	23.1	11.4	12.6	12.7	11.6	13.5
pK_a^{4b}	n.d.[k]	20.6	21.0	19.8	13.5	15.2	15.4	11.1	11.7

[a] Calculated through the thermodynamic cycle involving gas phase energies and solvated states without considering explicit molecules of water.
[b] L1: The solvated phase was calculated at HF/6-31+G(d)/cpcm=water and the gas phase was calculated with CBS-QB3 method according to ref. [41]
[c] L2: TPPSh-gd3bi/def2svpd/cpcm=water.
[d] L3 and L5: wb97xd/6-311++G(d,p)/smd=water/ /wb97xd/6-31+G(d,p)/smd=water.
[e] Calculated directly in solution and considering three explicit molecules of water.
[f] L4: HF/6-31+G(d)/cpcm=water.
[g] L6: wb97xd/def2tzvp/smd=water//wb97xd/6-31+G(d,p)/smd=water.
[h] L7: wb97xd/def2tzvppd/smd=water//wb97xd/6-31+G(d,p)/smd=water.
[i] L8: m062x/cc-pvtz/smd=water//m062x/6-31+G(d,p)/smd=water.
[j] L9: m062x/6-311++G(d,p)/smd=water//m062x/6-31+G(d,p)/smd=water
[k] n.d.: not determined. Given the observed difference with the first value and the computational cost, this method was abandoned.

Energy barriers and rate constants

Once both neutral and anionic mechanisms as well as pKa's have been calculated, it is possible to use Eqs. 19-24 to calculate predicted constants and barriers for the whole migration of the acetyl group in Me α-D-glucopyranoside. Scheme S7 illustrates the general scheme considered for the whole migration.

Tables S8 and S9 collects the predicted values of barriers and constants, respectively for the four different levels used. Experimental values are also included for comparison.

Table S8. Calculated free energy barriers (kcal·mol^{-1}) for the acetyl migration in Me α-D-glucopyranoside at pH=8.[a]

	experimental	level 1	level 2	level 3	level 4	level 5
ΔG_{1}^{obs}	23.2	25.5	27.1	27.3	21.5	22.2
ΔG_{-1}^{obs}	23.3	22.9	24.7	24.9	21.8	23.5
ΔG_{2}^{obs}	23.3	22.4	24.8	25.2	23.1	25.0
ΔG_{-2}^{obs}	22.9	25.0	27.1	27.3	22.7	24.8
ΔG_{3}^{obs}	21.4	22.2	23.9	24.2	19.8	21.8
ΔG_{-3}^{obs}	23.1	25.3	26.9	27.3	21.8	23.1

[a] Referred to Scheme S3. [b] The subindex refers to the corresponding rate constant in Scheme S4.
level 1: wb97xd/6-311++G(d,p)/smd=water //wb97xd/6-31+G(d,p)/smd=water.
level 2: wb97xd/def2tzvp/smd=water //wb97xd/6-31+G(d,p)/smd=water.
level 3: wb97xd/def2tzvpp/smd=water //wb97xd/6-31+G(d,p)/smd=water
level 4: m062x/6-311++G(d,p)/smd=water //m062x/6-31+G(d,p)/smd=water
level 5: m062x/cc-pvtz/smd=water //m062x/6-31+G(d,p)/smd=water

Table S9. Calculated rate constants (s^{-1}), obtained from Eqs. 19-24, for the acetyl migration in Me α-D-glucopyranoside at pH=8.[a]

	experimental	level 1	level 2	level 3	level 4	level 5
k_{1}^{obs}	6.28E-05	1.34E-06	9.23E-08	6.61E-08	6.28E-05	3.35E-04
k_{-1}^{obs}	5.11E-05	1.11E-04	5.28E-06	3.53E-06	5.11E-05	4.03E-05
k_{2}^{obs}	5.06E-05	2.35E-04	3.99E-06	2.06E-06	5.06E-05	3.15E-06
k_{-2}^{obs}	1.01E-04	3.26E-06	8.47E-08	6.28E-08	1.01E-04	4.50E-06
k_{3}^{obs}	1.21E-03	3.17E-04	1.86E-05	1.11E-05	1.21E-03	6.32E-04
k_{-3}^{obs}	7.83E-05	2.00E-06	1.25E-07	6.61E-08	7.83E-05	8.15E-05

[a] Referred to Scheme S3. [b] The subindex refers to the corresponding rate constant in Scheme S4.
level 1: wb97xd/6-311++G(d,p)/smd=water //wb97xd/6-31+G(d,p)/smd=water.
level 2: wb97xd/def2tzvp/smd=water //wb97xd/6-31+G(d,p)/smd=water.
level 3: wb97xd/def2tzvppd/smd=water //wb97xd/6-31+G(d,p)/smd=water
level 4: m062x/6-311++G(d,p)/smd=water //m062x/6-31+G(d,p)/smd=water
level 5: m062x/cc-pvtz/smd=water //m062x/6-31+G(d,p)/smd=water

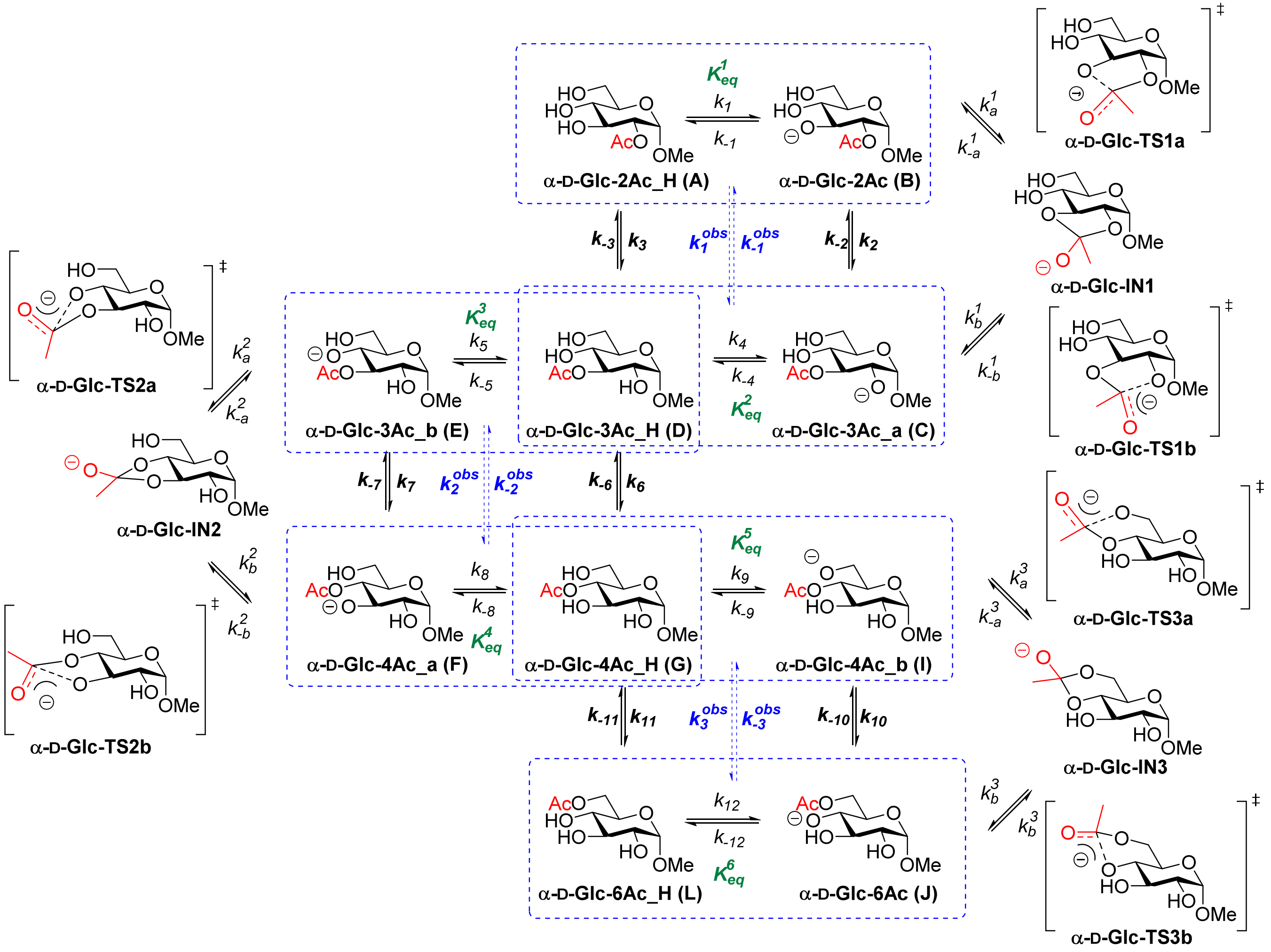

Scheme S7. General scheme for acetyl migration in Me α-D-glucopyranoside. The anionic mechanism is showed in detail. The neutral mechanism is not showed in detail.

The best level was level 5 showing differences less than 2.0 kcal/mol with respect to the experimental values. Nevertheless, for the purpose of comparison we will also use for the rest of the study levels 1 and 4 since these levels were the best one in past studies.[37,39]

The study was extended to the β anomer of D-glucopyranoside, and both α and β anomers of D-galactose and D-xylose following the same scheme illustrated for α-D- glucopyranoside in scheme S7. For these studies, the levels of theory were labeled as:

- level A: m062x/cc-pvtz/smd=water //m062x/6-31+G(d,p)/smd=water
- level B: m062x/6-311++G(d,p)/smd=water //m062x/6-31+G(d,p)/smd=water
- level C: wb97xd/6-311++G(d,p)/smd=water //wb97xd/6-31+G(d,p)/smd=water.

According to calculations with D-glucopyranoside, the neutral mechanism has rate constants in the range 10^{-9}-10^{-14} whereas the anionic mechanism has, at pH=8, rate constants in the range 10^{-4}-10^{-6} so, the former can be considered negligible and it is possible to assume that the reaction does not proceed through that mechanism. In Eq. 26 it is possible to approach $k_{neutral} \approx 0$ and the equation is reduced to:

$$K_i^{obs} = \frac{k_j^{anionic} \cdot K_{eq}^{m}}{[H]^+} \tag{30}$$

We can conclude that the migration proceeds exclusively through the anionic mechanism (barrier for neutral mechanism > 30 kcal/mol) and the rate depends on the availability of the anion, which is determined by concentration, a linear function of the corresponding pK_a and pH as reflects Eq. 30.

In fact, experiments carried out at different pH's correlate linearly with the observed rate constants. Consequently, for the rest of calculations with D-glucopyranoside, D-galactopyranoside and D-xylopyranoside, we used Eq. 30 and considered the general Scheme S7 (illustrated only for α-D-lucopyranoside but applicable just changing the configuration to D-gal and D-xyl).

Comparison of rate constants is not the best way to assess the validity of predictions because of the logarithmic dependence of the energy. So, we will discuss on the basis of energy barriers obtained from the calculated constants using the Eyring's equation (Eq. 1). Table S10 lists the predicted values of energy barriers obtained using the above-mentioned levels of theory. The differences in kcal/mol with respect to the experimental values are also indicated. For level 1, differences less than 2.0 kcal/mol,[29] within the limit of accuracy of DFT, are obtained in all cases demonstrating the validity of the methods. Actually, using ultrafine grids (as we did) it is possible to obtain free energy variances lower than 1 kcal/mol. Table S11 collects the corresponding values of rate constants.

Table S10. Calculated free energy barriers (kcal·mol^{-1}) for the acetyl migration at pH=8.[a]

		experimental[b]	level A		level B		level C	
			barrier	error[c]	barrier	error[c]	barrier	error[c]
α-D-Glc	ΔG_1	23.2	22.2	1.0	21.5	1.5	25.5	2.3
	ΔG_{-1}	23.3	23.5	0.1	21.8	0.3	22.9	0.5
	ΔG_2	23.3	25.0	1.6	23.1	0.3	22.4	0.9
	ΔG_{-2}	22.9	24.8	1.8	22.7	1.7	25.0	2.0
	ΔG_3	21.5	21.8	0.4	19.8	1.3	22.2	0.8
	ΔG_{-3}	23.1	23.1	0.0	21.8	0.0	25.3	2.2
β-D-Glc	ΔG_1	22.5	23.7	1.2	22.3	0.2	26.9	4.4
	ΔG_{-1}	22.9	22.1	0.8	20.7	2.1	25.0	2.1
	ΔG_2	22.8	22.2	0.7	20.8	2.0	24.3	1.4
	ΔG_{-2}	22.8	24.7	2.0	24.7	2.0	27.2	4.4
	ΔG_3	21.5	20.6	1.0	19.2	2.3	22.6	1.1
	ΔG_{-3}	23.4	22.1	1.3	19.5	3.9	23.4	0.0
α-D-Gal	ΔG_1	23.6	22.3	1.3	24.5	0.9	29.6	6.0
	ΔG_{-1}	23.7	22.7	1.1	22.6	1.1	29.5	5.7
	ΔG_2	22.4	20.4	2.0	21.2	1.2	24.0	1.6
	ΔG_{-2}	22.4	23.7	1.3	21.9	0.5	24.8	2.4
	ΔG_3	22.3	22.0	0.3	17.7	4.6	24.0	1.7
	ΔG_{-3}	23.3	22.4	0.9	17.9	5.4	23.0	0.3
β-D-Gal	ΔG_1	22.7	22.8	0.1	23.3	0.5	28.1	5.4
	ΔG_{-1}	23.2	23.5	0.3	22.9	0.3	28.6	5.4
	ΔG_2	22.4	22.6	0.2	18.7	3.7	22.9	0.5
	ΔG_{-2}	22.6	24.4	1.8	20.9	1.7	24.0	1.4
	ΔG_3	22.5	21.3	1.2	18.5	4.0	29.5	7.0
	ΔG_{-3}	23.3	23.3	0.0	20.4	2.9	28.5	5.3
α-D-Xyll	ΔG_1	23.2	22.4	0.8	22.1	1.1	27.2	3.9
	ΔG_{-1}	23.4	21.6	1.8	24.3	0.9	25.9	2.5
	ΔG_2	23.9	24.1	0.2	26.0	2.1	28.0	4.1
	ΔG_{-2}	23.8	22.8	1.0	24.4	0.6	28.4	4.6
β-D-Xyl	ΔG_1	22.6	23.7	1.0	22.5	0.2	27.2	4.5
	ΔG_{-1}	23.1	22.2	0.9	22.1	1.0	23.5	0.3
	ΔG_2	23.0	22.2	0.8	23.7	0.7	24.3	1.3
	ΔG_{-2}	23.1	21.2	1.9	19.8	3.3	27.5	4.4

[a] Referred to Scheme S7. General scheme for acetyl migration in pyranoside. The subindex of the free energy refers to the corresponding rate constant in Scheme 2. b obtained from the calculated constants using the Eyring's equation (Eq. 1). c in kcal/mol with respect to the corresponding experimental value.
level A: m062x/cc-pvtz/smd=water //m062x/6-31+G(d,p)/smd=water
level B: m062x/6-311++G(d,p)/smd=water //m062x/6-31+G(d,p)/smd=water
level C: wb97xd/6-311++G(d,p)/smd=water //wb97xd/6-31+G(d,p)/smd=water.

Table S11. Calculated rate constants (s^{-1}), obtained from Eqs 19-24, for the acetyl migration at pH=8.[a]

		experimental[b]	level A	level B	level C
α-D-Glc	K_{obs}^{1}	6.28E-05	3.35E-04	1.09E-03	1.34E-06
	K_{obs}^{-1}	5.11E-05	4.03E-05	6.78E-04	1.11E-04
	K_{obs}^{2}	5.06E-05	3.15E-06	7.72E-05	2.35E-04
	K_{obs}^{-2}	1.01E-04	4.50E-06	1.59E-04	3.26E-06
	K_{obs}^{3}	1.21E-03	6.32E-04	2.14E-02	3.17E-04
	K_{obs}^{-3}	7.83E-05	8.15E-05	7.32E-04	2.00E-06
β-D-Glc	K_{obs}^{1}	2.02E-04	2.72E-05	2.80E-04	1.24E-07
	K_{obs}^{-1}	1.11E-04	4.28E-04	4.15E-03	3.12E-06
	K_{obs}^{2}	1.15E-04	3.58E-04	3.48E-03	1.06E-05
	K_{obs}^{-2}	1.28E-04	4.71E-06	4.71E-06	7.07E-08
	K_{obs}^{3}	1.10E-03	5.48E-03	5.33E-02	1.77E-04
	K_{obs}^{-3}	4.56E-05	4.01E-04	3.15E-02	4.51E-05
α-D-Gal	K_{obs}^{1}	3.31E-05	2.99E-04	7.14E-06	1.33E-09
	K_{obs}^{-1}	2.65E-05	1.56E-04	1.62E-04	1.68E-09
	K_{obs}^{2}	2.43E-04	6.75E-03	1.97E-03	1.57E-05
	K_{obs}^{-2}	2.43E-04	2.94E-05	5.37E-04	4.15E-06
	K_{obs}^{3}	2.83E-04	4.83E-04	6.48E-01	1.62E-05
	K_{obs}^{-3}	5.47E-05	2.34E-04	4.64E-01	8.56E-05
β-D-Gal	K_{obs}^{1}	1.47E-04	1.19E-04	5.83E-05	1.68E-08
	K_{obs}^{-1}	6.08E-05	3.94E-05	1.08E-04	6.63E-09
	K_{obs}^{2}	2.43E-04	1.88E-04	1.17E-01	1.12E-04
	K_{obs}^{-2}	1.72E-04	8.78E-06	2.89E-03	1.64E-05
	K_{obs}^{3}	2.10E-04	1.49E-03	1.87E-01	1.46E-09
	K_{obs}^{-3}	5.72E-05	5.54E-05	7.35E-03	8.13E-09
α-D-Xyl	K_{obs}^{1}	5.86E-05	2.27E-04	3.98E-04	8.10E-08
	K_{obs}^{-1}	4.25E-05	9.44E-04	9.89E-06	6.53E-07
	K_{obs}^{2}	2.03E-05	1.47E-05	5.89E-07	2.10E-08
	K_{obs}^{-2}	2.34E-05	1.25E-04	8.10E-06	1.02E-08
β-D-Xyl	K_{obs}^{1}	1.65E-04	2.85E-05	2.20E-04	8.06E-08
	K_{obs}^{-1}	7.33E-05	3.52E-04	4.28E-04	4.16E-05
	K_{obs}^{2}	9.11E-05	3.27E-04	2.84E-05	1.02E-05
	K_{obs}^{-2}	7.81E-05	1.90E-03	1.99E-02	4.86E-08

[a] Referred to Scheme S7.
level A: m062x/cc-pvtz/smd=water //m062x/6-31+G(d,p)/smd=water
level B: m062x/6-311++G(d,p)/smd=water //m062x/6-31+G(d,p)/smd=water
level C: wb97xd/6-311++G(d,p)/smd=water //wb97xd/6-31+G(d,p)/smd=water.

However, calculations with carbohydrates are tricky because it is necessary to study many conformations for minima and transition structures that can vary their free energy values (see above). The protocol we have followed consisted of choosing the structure of minimum energy in which no weak intramolecular interactions, unlikely in an aqueous medium, were present. For this purpose, molecular dynamics simulations (see above) were carried out (as an example, Figure S58 shows Me 2-

O-Ac-α-D-glucopyranoside and its 3-anion) what allow identifying remaining intramolecular interactions and starting positions of explicit water molecules (see above, Figure S57) to be used in QM calculations. We obtained, in this way, structures with isodesmic non-covalent interactions so that they could be compared in a consistent manner.

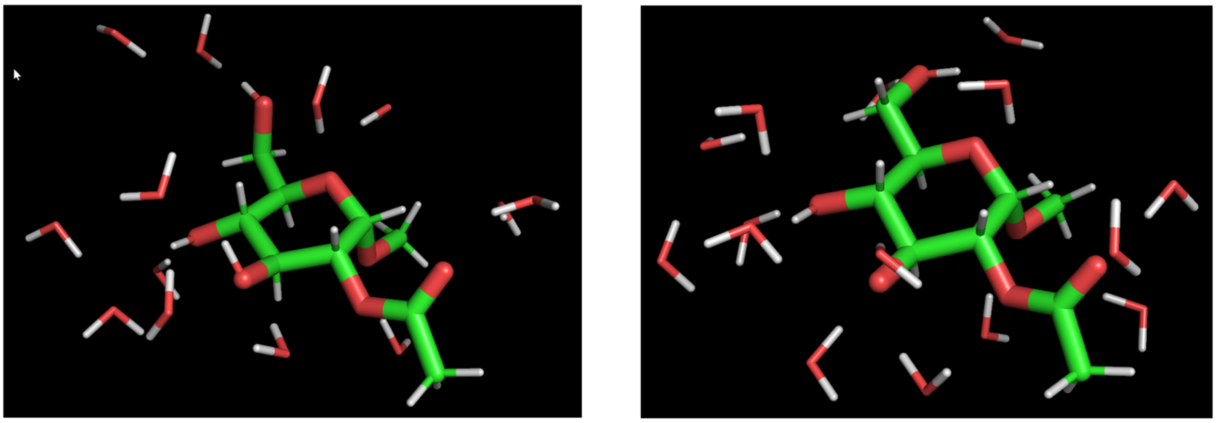

Figure S58. MD (100 ns, see above, Figure S57) of Me 2-*O*-Ac-α-D-glucopyranoside (left) and its 3-anion (right) immersed in a box of water (12 Å). Only those water molecules interacting in a direct way with the molecule are shown. It can be appreciated the absence of intramolecular H-bonds.

Energies

Preliminary calculations

Table S12. Calculated (wb97xd/6-311+G(d,p)/smd=water//tppsh-gd3bj/def2tzvpd/cpcm=water) absolute (hartree) and relative (kcal/mol) energies for acyl migration in Me α-D-Glucopyranoside from position 2 to position 3 under a neutral mechanism mediated by one molecule of water .

	E_0	ΔE_0	G	ΔG	im. freq
α-D-Glc-2Ac-1w	-955.716759	0.0	-955.766438	0.0	
α-D-Glc-TSa-1w	-955.676564	25.2	-955.722510	27.6	-1179.9
α-D-Glc-INa-1w	-955.701354	9.7	-955.750301	10.1	
α-D-Glc-TSb-1w	-955.677485	24.6	-955.723645	26.9	-1196.4
α-D-Glc-3Ac-1w	-955.718481	-1.1	-955.767388	-0.6	

Table S13. Calculated (wb97xd/6-311+G(d,p)/smd=water//tppsh-gd3bj/def2tzvpd/cpcm=water) absolute (hartree) and relative (kcal/mol) energies for acyl migration in Me α-D-Glucopyranoside from position 2 to position 3 under a neutral mechanism mediated by two molecules of water .

	E_0	ΔE_0	G	ΔG	im. freq
α-D-Glc-2Ac-2w	-1032.171957	0.0	-1032.225147	0.0	
α-D-Glc-TSa-1w	-1032.130043	26.3	-1032.179096	28.9	-819.0
α-D-Glc-INa-1w	-1032.151409	12.9	-1032.203485	13.6	
α-D-Glc-TSb-1w	-1032.131259	25.5	-1032.180944	27.7	-880.4
α-D-Glc-3Ac-2w	-1032.171681	0.2	-1032.22484	0.2	

Table S14. Calculated (wb97xd/6-311+G(d,p)/smd=water//tppsh-gd3bj/def2tzvpd/cpcm=water) absolute (hartree) and relative (kcal/mol) energies for acyl migration in Me α-D-Glucopyranoside from position 2 to position 3 through a naked anion .

	E_0	ΔE_0	G	ΔG	im. freq
α-D-Glc-2Ac-n	-878.7828466	0.0	-877.9009491	0.0	
α-D-Glc-TSa-n	-878.7757503	4.5	-877.8892455	7.3	-110.2
α-D-Glc-INa-n	-878.7820559	0.5	-877.8960551	3.4	
α-D-Glc-TSb-n	-878.7796771	2.0	-877.8930628	5.0	-65.7
α-D-Glc-3Ac-n	-878.7873378	-2.8	-877.9033487	-1.2	

Table S15. Calculated (wb97xd/6-311+G(d,p)/smd=water//tppsh-gd3bj/def2tzvpd/cpcm=water) absolute (hartree) and relative (kcal/mol) energies for acyl migration in Me α-D-Glucopyranoside from position 2 to position 3 through a naked anion including one explicit molecule of water surrounding the anion.

	E_0	ΔE_0	G	ΔG	im. freq
α-D-Glc-2Ac-n1w	-954.867833	0.0	-954.916268	0.0	
α-D-Glc-TSa-n1w	-954.847455	12.8	-954.894310	13.8	-243.6
α-D-Glc-IN-n1w	-954.855571	7.7	-954.902789	8.5	
α-D-Glc-TSb-n1w	-954.850208	11.1	-954.896556	12.4	-236.8
α-D-Glc-3Ac-n1w	-954.865451	1.5	-954.912060	2.6	

Table S16. Calculated (wb97xd/6-311+G(d,p)/smd=water//tppsh-gd3bj/def2tzvpd/cpcm=water) absolute (hartree) and relative (kcal/mol) energies for acyl migration in Me α-D-Glucopyranoside from position 2 to position 3 through a naked anion including two explicit molecules of water surrounding the anion.

	E_0	ΔE_0	G	ΔG	im. freq
α-D-Glc-2Ac-n2w	-1031.302686	0.0	-1031.354482	0.0	
α-D-Glc-TSa-n2w	-1031.281254	13.4	-1031.331575	14.4	-284.7
α-D-Glc-IN-n2w	-1031.287893	9.3	-1031.337345	10.8	
α-D-Glc-TSb-n2w	-1031.283270	12.2	-1031.334421	12.6	-210.3
α-D-Glc-3Ac-n2w	-1031.299964	1.7	-1031.351640	1.8	

Model with three explicit molecules of water surrounding the anion

Table S17. Calculated (m062x/cc-pvtz/smd=water//m062x/6-31+G(d,p)/smd=water) absolute (hartree) and relative (kcal/mol) energies for acyl migration in Me α-D-Glucopyranoside.[a]

	E_0	ΔE_0	G	ΔG	im. freq
α-D-Glc-2Ac_H	-1108.18289539	-24.6	-1108.23563372	-24.6	
α-D-Glc-2Ac	-1107.72293963	0.0	-1107.77568533	0.0	
α-D-Glc-TS1a	-1107.70175866	13.3	-1107.75211373	14.8	-252.3
α-D-Glc-IN1	-1107.70821336	9.2	-1107.76128070	9.0	
α-D-Glc-TS1b	-1107.70560602	10.9	-1107.75626732	12.2	-226.9
α-D-Glc-3Ac_a	-1107.72482594	-1.2	-1107.77809944	-1.5	
α-D-Glc-3Ac_H	-1108.18524946	-26.1	-1108.23763369	-25.9	
α-D-Glc-3Ac_b	-1107.72707306	-2.6	-1107.77716972	-0.9	
α-D-Glc-TS2a	-1107.70388695	12.0	-1107.75382009	13.7	-229.3
α-D-Glc-IN2	-1107.70745421	9.7	-1107.76085314	9.3	
α-D-Glc-TS2b	-1107.70010913	14.3	-1107.74970588	0.0	-260.1
α-D-Glc-4Ac_a	-1107.72973185	-4.3	-1107.78008584	-19.1	
α-D-Glc-4Ac_H	-1108.18371364	-25.1	-1108.23729121	-42.0	
α-D-Glc-4Ac_b	-1107.72183090	0.7	-1107.77406007	-15.3	
α-D-Glc-TS3a	-1107.70110502	13.7	-1107.75135187	-1.0	-270.2
α-D-Glc-IN3	-1107.71507348	4.9	-1107.76651129	-10.5	
α-D-Glc-TS3b	-1107.70674248	10.2	-1107.75524295	-3.5	-232.9
α-D-Glc-6Ac	-1107.72567236	-1.7	-1107.78000931	-19.0	
α-D-Glc-6Ac_H	-1108.18463507	-25.7	-1108.23921963	-43.2	

[a] Referred to α-D-Glc-2Ac. For the protonated derivatives the values have been calculated considering aqueous phase proton free energy (-265.9 kcal/mol)[42–44] and the free energy change due to changing the standard state from 1 atm to 1 M (1.89 kcal/mol; see Eq. 18) according to Eq. 27. The nomenclature of compounds corresponds to that given in Scheme S7.

Table S18. Calculated (m062x/6-311++G(d,p)/smd=water//m062x/6-31+G(d,p)/smd=water) absolute (hartree) and relative (kcal/mol) energies for acyl migration in Me α-D-Glucopyranoside.[a]

	E_0	ΔE_0	G	ΔG	im. freq
α-D-Glc-2Ac_H	-1108.09940531	-23.9	-1108.15214364	-23.9	
α-D-Glc-2Ac	-1107.64058254	0.0	-1107.69332824	0.0	
α-D-Glc-TS1a	-1107.61939194	13.3	-1107.66974701	14.8	-252.3
α-D-Glc-IN1	-1107.62672491	8.7	-1107.67979225	8.5	
α-D-Glc-TS1b	-1107.62290174	11.1	-1107.67356304	12.4	-226.9
α-D-Glc-3Ac_a	-1107.64253798	-1.2	-1107.69581148	-1.6	
α-D-Glc-3Ac_H	-1108.10020900	-24.4	-1108.15259323	-24.2	
α-D-Glc-3Ac_b	-1107.64443398	-2.4	-1107.69453064	-0.8	
α-D-Glc-TS2a	-1107.62138638	12.0	-1107.67131952	13.8	-229.3
α-D-Glc-IN2	-1107.62558854	9.4	-1107.67898747	9.0	
α-D-Glc-TS2b	-1107.61810167	14.1	-1107.66769842	0.0	-260.1
α-D-Glc-4Ac_a	-1107.64686600	-3.9	-1107.69721999	-18.5	
α-D-Glc-4Ac_H	-1108.09833200	-23.2	-1108.15190957	-23.8	
α-D-Glc-4Ac_b	-1107.64067100	-0.1	-1107.69290017	-15.8	
α-D-Glc-TS3a	-1107.61905932	13.5	-1107.66930617	-1.0	-270.2
α-D-Glc-IN3	-1107.63247081	5.1	-1107.68390862	-10.2	
α-D-Glc-TS3b	-1107.62435375	10.2	-1107.67285422	-3.2	-232.9
α-D-Glc-6Ac	-1107.64286500	-1.4	-1107.69720195	-18.5	
α-D-Glc-6Ac_H	-1108.10050879	-24.6	-1108.15509335	-25.8	

[a] Referred to α-D-Glc-2Ac. For the protonated derivatives the values have been calculated considering aqueous phase proton free energy (-265.9 kcal/mol)[42–44] and the free energy change due to changing the standard state from 1 atm to 1 M (1.89 kcal/mol; see Eq. 18) according to Eq. 27. The nomenclature of compounds corresponds to that given in Scheme S7.

Table S19. Calculated (wb97xd/6-311++G(d,p)/smd=water//wb97xd/6-31+G(d,p)/smd=water) absolute (hartree) and relative (kcal/mol) energies for acyl migration in Me α-D-Glucopyranoside.[a]

	E_0	ΔE_0	G	ΔG	im. freq
α-D-Glc-2Ac_H	-1108.09940531	-23.9	-1108.15214364	-23.9	
α-D-Glc-2Ac	-1107.64058254	0.0	-1107.69332824	0.0	
α-D-Glc-TS1a	-1107.61939194	13.3	-1107.66974701	14.8	-252.3
α-D-Glc-IN1	-1107.62672491	8.7	-1107.67979225	8.5	
α-D-Glc-TS1b	-1107.62290174	11.1	-1107.67356304	12.4	-226.9
α-D-Glc-3Ac_a	-1107.64253798	-1.2	-1107.69581148	-1.6	
α-D-Glc-3Ac_H	-1108.10020900	-24.4	-1108.15259323	-24.2	
α-D-Glc-3Ac_b	-1107.64443398	-2.4	-1107.69453064	-0.8	
α-D-Glc-TS2a	-1107.62138638	12.0	-1107.67131952	13.8	-229.3
α-D-Glc-IN2	-1107.62558854	9.4	-1107.67898747	9.0	
α-D-Glc-TS2b	-1107.61810167	14.1	-1107.66769842	0.0	-260.1
α-D-Glc-4Ac_a	-1107.64686600	-3.9	-1107.69721999	-18.5	
α-D-Glc-4Ac_H	-1108.09833200	-23.2	-1108.15190957	-23.8	
α-D-Glc-4Ac_b	-1107.64067100	-0.1	-1107.69290017	-15.8	
α-D-Glc-TS3a	-1107.61905932	13.5	-1107.66930617	-1.0	-270.2
α-D-Glc-IN3	-1107.63247081	5.1	-1107.68390862	-10.2	
α-D-Glc-TS3b	-1107.62435375	10.2	-1107.67285422	-3.2	-232.9
α-D-Glc-6Ac	-1107.64286500	-1.4	-1107.69720195	-18.5	
α-D-Glc-6Ac_H	-1108.10050879	-24.6	-1108.15509335	-25.8	

[a] Referred to α-D-Glc-2Ac. For the protonated derivatives (*_H compounds) the values have been calculated considering aqueous phase proton free energy (-265.9 kcal/mol)[42–44] and the free energy change due to changing the standard state from 1 atm to 1 M (1.89 kcal/mol; see Eq. 18) according to Eq. 27. The nomenclature of compounds corresponds to that given in Scheme S7.

Table S20. Calculated (wb97xd/def2tzvppd/smd=water//wb97xd/6-31+G(d,p)/smd=water) absolute (hartree) and relative (kcal/mol) energies for acyl migration in Me α-D-Glucopyranoside.[a]

	E_0	ΔE_0	G	ΔG	im. freq
α-D-Glc-2Ac_H	-1108.32512493	-26.9	-1108.37731604	-24.5	
α-D-Glc-2Ac	-1107.86147815	0.0	-1107.91752936	0.0	
α-D-Glc-TS1a	-1107.83604325	16.0	-1107.88690405	19.2	-248.4
α-D-Glc-IN1	-1107.84557551	10.0	-1107.89995086	11.0	
α-D-Glc-TS1b	-1107.83491945	16.7	-1107.88605245	19.8	-183.3
α-D-Glc-3Ac_a	-1107.85880135	1.7	-1107.91117826	4.0	
α-D-Glc-3Ac_H	-1108.32172209	-24.8	-1108.37356302	-22.2	
α-D-Glc-3Ac_b	-1107.86365403	-1.4	-1107.91481566	1.7	
α-D-Glc-TS2a	-1107.83999357	13.5	-1107.89006395	17.2	-236.1
α-D-Glc-IN2	-1107.84457358	10.6	-1107.89948893	11.3	
α-D-Glc-TS2b	-1107.83466093	16.8	-1107.88523157	0.0	-238.8
α-D-Glc-4Ac_a	-1107.86153591	0.0	-1107.91306799	-17.5	
α-D-Glc-4Ac_H	-1108.32539947	-27.1	-1108.37686465	-24.2	
α-D-Glc-4Ac_b	-1107.85965233	1.1	-1107.91552201	-19.0	
α-D-Glc-TS3a	-1107.83801278	14.7	-1107.88936538	-2.6	-314.3
α-D-Glc-IN3	-1107.84986688	7.3	-1107.90372017	-11.6	
α-D-Glc-TS3b	-1107.83951759	13.8	-1107.88718893	-1.2	-225.3
α-D-Glc-6Ac	-1107.86260316	-0.7	-1107.91441110	-18.3	
α-D-Glc-6Ac_H	-1108.32921455	-29.5	-1108.38171136	-27.3	

[a] Referred to α-D-Glc-2Ac. For the protonated derivatives (*_H compounds) the values have been calculated considering aqueous phase proton free energy (-265.9 kcal/mol)[42–44] and the free energy change due to changing the standard state from 1 atm to 1 M (1.89 kcal/mol; see Eq. 18) according to Eq. 27. The nomenclature of compounds corresponds to that given in Scheme S7.

Table S21. Calculated (wb97xd/def2tzvp/smd=water//wb97xd/6-31+G(d,p)/smd=water) absolute (hartree) and relative (kcal/mol) energies for acyl migration in Me α-D-Glucopyranoside.[a]

	E_0	ΔE_0	G	ΔG	im. freq
α-D-Glc-2Ac_H	-1108.30068403	-26.6	-1108.35287514	-24.2	
α-D-Glc-2Ac	-1107.83752508	0.0	-1107.89357629	0.0	
α-D-Glc-TS1a	-1107.81227332	15.8	-1107.86313412	19.1	-248.4
α-D-Glc-IN1	-1107.82058458	10.6	-1107.87495993	11.7	
α-D-Glc-TS1b	-1107.81068114	16.8	-1107.86181414	19.9	-183.3
α-D-Glc-3Ac_a	-1107.83474039	1.7	-1107.88711730	4.1	
α-D-Glc-3Ac_H	-1108.29721721	-24.5	-1108.34905814	-21.8	
α-D-Glc-3Ac_b	-1107.83514427	1.5	-1107.88630590	4.6	
α-D-Glc-TS2a	-1107.81620827	13.4	-1107.86627865	17.1	-236.1
α-D-Glc-IN2	-1107.81971056	11.2	-1107.87462591	11.9	
α-D-Glc-TS2b	-1107.81077563	16.8	-1107.86134627	0.0	-238.8
α-D-Glc-4Ac_a	-1107.83763082	-0.1	-1107.88916290	-17.5	
α-D-Glc-4Ac_H	-1108.30123260	-27.0	-1108.35269778	-24.1	
α-D-Glc-4Ac_b	-1107.83554309	1.2	-1107.89141277	-18.9	
α-D-Glc-TS3a	-1107.81388566	14.8	-1107.86523826	-2.4	-314.3
α-D-Glc-IN3	-1107.82564614	7.5	-1107.87949943	-11.4	
α-D-Glc-TS3b	-1107.81589103	13.6	-1107.86356237	-1.4	-225.3
α-D-Glc-6Ac	-1107.83868926	-0.7	-1107.89049720	-18.3	
α-D-Glc-6Ac_H	-1108.30492674	-29.3	-1108.35742355	-27.1	

[a] Referred to α-D-Glc-2Ac. For the protonated derivatives (*_H compounds) the values have been calculated considering aqueous phase proton free energy (-265.9 kcal/mol)[42–44] and the free energy change due to changing the standard state from 1 atm to 1 M (1.89 kcal/mol; see Eq. 18) according to Eq. 27. The nomenclature of compounds corresponds to that given in Scheme S7.

Table S22. Calculated (m062x/cc-pvtz/smd=water//m062x/6-31+G(d,p)/smd=water) absolute (hartree) and relative (kcal/mol) energies for acyl migration in Me β-D-Glucopyranoside.[a]

	E_0	ΔE_0	G	ΔG	im. freq
β-D-Glc-2Ac_H	-1108.18347748	-22.8	-1108.23696550	-23.9	
β-D-Glc-2Ac	-1107.72647407	0.0	-1107.77808327	0.0	
β-D-Glc-TS1a	-1107.70129077	15.8	-1107.75204089	16.3	-253.6
β-D-Glc-IN1	-1107.70270867	14.9	-1107.75226737	16.2	
β-D-Glc-TS1b	-1107.70200222	15.4	-1107.75148103	16.7	-215.7
β-D-Glc-3Ac_a	-1107.72193223	2.9	-1107.77424658	2.4	
β-D-Glc-3Ac_H	-1108.18134713	-21.4	-1108.23436732	-22.3	
β-D-Glc-3Ac_b	-1107.72725909	-0.5	-1107.78025825	-1.4	
β-D-Glc-TS2a	-1107.70284010	14.8	-1107.75314998	15.6	-223.5
β-D-Glc-IN2	-1107.70648947	12.5	-1107.75919086	11.9	
β-D-Glc-TS2b	-1107.70017220	16.5	-1107.75099813	-0.8	-262.6
β-D-Glc-4Ac_a	-1107.72955275	-1.9	-1107.78041131	-19.3	
β-D-Glc-4Ac_H	-1108.18319254	-22.6	-1108.23846119	-24.9	
β-D-Glc-4Ac_b	-1107.72025628	3.9	-1107.77635012	-16.7	
β-D-Glc-TS3a	-1107.70528571	13.3	-1107.75671037	-4.4	-297.0
β-D-Glc-IN3	-1107.71133277	9.5	-1107.76376828	-8.8	
β-D-Glc-TS3b	-1107.70448702	13.8	-1107.75464709	-3.1	-221.9
β-D-Glc-6Ac	-1107.72546257	0.6	-1107.77867266	-18.2	
β-D-Glc-6Ac_H	-1108.18598253	-24.3	-1108.24092935	-26.4	

[a] Referred to β-D-Glc-2Ac. For the protonated derivatives (*_H compounds) the values have been calculated considering aqueous phase proton free energy (-265.9 kcal/mol)[42–44] and the free energy change due to changing the standard state from 1 atm to 1 M (1.89 kcal/mol; see Eq. 18) according to Eq. 27. The nomenclature of compounds corresponds to that given in Scheme S7 in which α-D-Glc- configuration must be changed by β-D-Glc-.

Table S23. Calculated (m062x/6-311++G(d,p)/smd=water//m062x/6-31+G(d,p)/smd=water) absolute (hartree) and relative (kcal/mol) energies for acyl migration in Me β-D-Glucopyranoside.[a]

	E_0	ΔE_0	G	ΔG	im. freq
β-D-Glc-2Ac_H	-1108.18120648	-21.3	-1108.23476175	-22.6	
β-D-Glc-2Ac	-1107.72647407	-53.9	-1107.77808327	-53.2	
β-D-Glc-TS1a	-1107.70129077	-38.1	-1107.75204089	-36.8	-253.6
β-D-Glc-IN1	-1107.70270867	-39.0	-1107.75226737	-37.0	
β-D-Glc-TS1b	-1107.70200222	-38.5	-1107.75148103	-36.5	-215.7
β-D-Glc-3Ac_a	-1107.72193223	-51.0	-1107.77424658	-50.8	
β-D-Glc-3Ac_H	-1108.17836282	-19.6	-1108.23221833	-21.0	
β-D-Glc-3Ac_b	-1107.73010290	-56.2	-1107.78097795	-55.0	
β-D-Glc-TS2a	-1107.70284010	-39.1	-1107.75314998	-37.5	-223.5
β-D-Glc-IN2	-1107.70648947	-41.4	-1107.75919086	-41.3	
β-D-Glc-TS2b	-1107.70017220	-37.4	-1107.75099813	-52.3	-262.6
β-D-Glc-4Ac_a	-1107.72955275	-55.8	-1107.78041131	-70.7	
β-D-Glc-4Ac_H	-1108.18319254	-22.6	-1108.23846119	-24.9	
β-D-Glc-4Ac_b	-1107.72025628	-50.0	-1107.77635012	-68.2	
β-D-Glc-TS3a	-1107.70528571	-40.6	-1107.75671037	-55.9	-297.0
β-D-Glc-IN3	-1107.71133277	-44.4	-1107.76376828	-60.3	
β-D-Glc-TS3b	-1107.71140522	-44.4	-1107.76057932	-58.3	-265.2
β-D-Glc-6Ac	-1107.72546257	-53.3	-1107.77867266	-69.6	
β-D-Glc-6Ac_H	-1108.18467810	-23.5	-1108.23895673	-25.2	

[a] Referred to β-D-Glc-2Ac. For the protonated derivatives (*_H compounds) the values have been calculated considering aqueous phase proton free energy (-265.9 kcal/mol)[42–44] and the free energy change due to changing the standard state from 1 atm to 1 M (1.89 kcal/mol; see Eq. 18) according to Eq. 27. The nomenclature of compounds corresponds to that given in Scheme S7 in which α-D-Glc- configuration must be changed by β-D-Glc-.

Table S24. Calculated (wb97xd/6-311++G(d,p)/smd=water//wb97xd/6-31+G(d,p)/smd=water) absolute (hartree) and relative (kcal/mol) energies for acyl migration in Me β-D-Glucopyranoside.[a]

	E_0	ΔE_0	G	ΔG	im. freq
β-D-Glc-2Ac_H	-1108.19840445	-26.8	-1108.25154522	-25.8	
β-D-Glc-2Ac	-1107.73494129	1.0	-1107.78972705	1.8	
β-D-Glc-TS1a	-1107.71121757	15.9	-1107.76186542	19.3	-254.0
β-D-Glc-IN1	-1107.71325675	14.6	-1107.76327056	18.4	
β-D-Glc-TS1b	-1107.71112676	15.9	-1107.76081829	19.9	-196.4
β-D-Glc-3Ac_a	-1107.73402945	1.6	-1107.78960691	1.9	
β-D-Glc-3Ac_H	-1108.19673406	-25.8	-1108.24849381	-23.9	
β-D-Glc-3Ac_b	-1107.73730756	-0.5	-1107.78747244	3.2	
β-D-Glc-TS2a	-1107.71409664	14.1	-1107.76440265	17.7	-230.5
β-D-Glc-IN2	-1107.71771983	11.8	-1107.77046386	13.9	
β-D-Glc-TS2b	-1107.71052435	16.3	-1107.76175934	0.1	-259.1
β-D-Glc-4Ac_a	-1107.74213233	-3.5	-1107.79476926	-20.6	
β-D-Glc-4Ac_H	-1108.19857695	-26.9	-1108.25322358	-26.8	
β-D-Glc-4Ac_b	-1107.73440768	1.3	-1107.78995107	-17.6	
β-D-Glc-TS3a	-1107.71500267	13.5	-1107.76614273	-2.6	-311.2
β-D-Glc-IN3	-1107.72082602	9.9	-1107.77289441	-6.8	
β-D-Glc-TS3b	-1107.71899234	11.0	-1107.76847708	-4.1	-262.0
β-D-Glc-6Ac	-1107.73906079	-1.6	-1107.79374351	-19.9	
β-D-Glc-6Ac_H	-1108.20104246	-28.5	-1108.25450959	-27.6	

[a] Referred to β-D-Glc-2Ac. For the protonated derivatives (*_H compounds) the values have been calculated considering aqueous phase proton free energy (-265.9 kcal/mol)[42–44] and the free energy change due to changing the standard state from 1 atm to 1 M (1.89 kcal/mol; see Eq. 18) according to Eq. 27. The nomenclature of compounds corresponds to that given in Scheme S7 in which α-D-Glc- configuration must be changed by β-D-Glc-.

Table S25. Calculated (m062x/cc-pvtz/smd=water//m062x/6-31+G(d,p)/smd=water) absolute (hartree) and relative (kcal/mol) energies for acyl migration in Me α-D-Galactopyranoside.[a]

	E_0	ΔE_0	G	ΔG	im. freq
α-D-Gal-2Ac_H	-1108.17915995	-24.0	-1108.23116008	-22.9	
α-D-Gal-2Ac	-1107.72024187	0.0	-1107.77386393	0.0	
α-D-Gal-TS1a	-1107.69928022	13.2	-1107.74891105	15.7	-272.7
α-D-Gal-IN1	-1107.70304944	10.8	-1107.75444970	12.2	
α-D-Gal-TS1b	-1107.69823580	13.8	-1107.74777241	16.4	-224.7
α-D-Gal-3Ac_a	-1107.72174208	-0.9	-1107.77286000	0.6	
α-D-Gal-3Ac_H	-1108.17934798	-24.1	-1108.23177613	-23.3	
α-D-Gal-3Ac_b	-1107.72149219	-0.8	-1107.77178501	1.3	
α-D-Gal-TS2a	-1107.70108006	12.0	-1107.75108062	14.3	-194.5
α-D-Gal-IN2	-1107.72320216	-1.9	-1107.77278792	0.7	
α-D-Gal-TS2b	-1107.70784872	7.8	-1107.75761172	10.2	-241.4
α-D-Gal-4Ac_a	-1107.72475976	-2.8	-1107.77749749	-2.3	
α-D-Gal-4Ac_H	-1108.18362708	-26.8	-1108.23690686	-26.6	
α-D-Gal-4Ac_b	-1107.72190388	-1.0	-1107.77212804	1.1	
α-D-Gal-TS3a	-1107.70181370	11.6	-1107.75069582	14.5	-288.4
α-D-Gal-IN3	-1107.72164682	-0.9	-1107.77173526	1.3	
α-D-Gal-TS3b	-1107.71406996	3.9	-1107.76286225	6.9	-265.0
α-D-Gal-6Ac	-1107.72676513	-4.1	-1107.77812286	-2.7	
α-D-Gal-6Ac_H	-1108.18607488	-28.3	-1108.23758190	-27.0	

[a] Referred to α-D-Gal-2Ac. For the protonated derivatives (*_H compounds) the values have been calculated considering aqueous phase proton free energy (-265.9 kcal/mol)[42–44]and the free energy change due to changing the standard state from 1 atm to 1 M (1.89 kcal/mol; see Eq. 18) according to Eq. 27. The nomenclature of compounds corresponds to that given in Scheme S7 in which α-D-Glc- configuration must be changed by α-D-Gal-.

Table S26 Calculated (m062x/6-311++G(d,p)/smd=water//m062x/6-31+G(d,p)/smd=water) absolute (hartree) and relative (kcal/mol) energies for acyl migration in Me α-D-Galactopyranoside.[a]

	E_0	ΔE_0	G	ΔG	im. freq
α-D-Gal-2Ac_H	-1108.09838388	-25.0	-1108.15308206	-25.7	
α-D-Gal-2Ac	-1107.63775779	1.8	-1107.69137985	1.2	
α-D-Gal-TS1a	-1107.61735676	14.6	-1107.66698759	16.5	-272.7
α-D-Gal-IN1	-1107.62150937	12.0	-1107.67290963	12.8	
α-D-Gal-TS1b	-1107.61674360	15.0	-1107.66628021	17.0	-224.7
α-D-Gal-3Ac_a	-1107.63932782	0.8	-1107.69044574	1.8	
α-D-Gal-3Ac_H	-1108.09677918	-24.0	-1108.15013223	-23.9	
α-D-Gal-3Ac_b	-1107.63838076	1.4	-1107.68867358	2.9	
α-D-Gal-TS2a	-1107.61899245	13.5	-1107.66827340	15.7	-222.9
α-D-Gal-IN2	-1107.64008545	0.3	-1107.68967121	2.3	
α-D-Gal-TS2b	-1107.62598143	9.2	-1107.67574443	-5.0	-241.4
α-D-Gal-4Ac_a	-1107.64358741	-1.9	-1107.69632514	-18.0	
α-D-Gal-4Ac_H	-1108.09895514	-25.4	-1108.15135262	-24.6	
α-D-Gal-4Ac_b	-1107.64007294	0.3	-1107.69029710	-14.2	
α-D-Gal-TS3a	-1107.62601726	9.1	-1107.67615070	-5.3	-316.2
α-D-Gal-IN3	-1107.64197175	-0.9	-1107.69115651	-14.7	
α-D-Gal-TS3b	-1107.62424185	10.3	-1107.67195767	-2.7	-229.4
α-D-Gal-6Ac	-1107.64445703	-2.4	-1107.69581476	-17.6	
α-D-Gal-6Ac_H	-1108.10015434	-26.1	-1108.15166136	-24.8	

[a] Referred to α-D-Gal-2Ac. For the protonated derivatives (*_H compounds) the values have been calculated considering aqueous phase proton free energy (-265.9 kcal/mol)[42–44]and the free energy change due to changing the standard state from 1 atm to 1 M (1.89 kcal/mol; see Eq. 18) according to Eq. 27. The nomenclature of compounds corresponds to that given in Scheme S7 in which α-D-Glc- configuration must be changed by α-D-Gal-.

Table S27. Calculated (wb97xd/6-311++G(d,p)/smd=water//wb97xd/6-31+G(d,p)/smd=water) absolute (hartree) and relative (kcal/mol) energies for acyl migration in Me α-D-Galactopyranoside.[a]

	E_0	ΔE_0	G	ΔG	im. freq
α-D-Gal-2Ac_H	-1108.19846209	-27.9	-1108.25120198	-27.7	
α-D-Gal-2Ac	-1107.73329114	2.0	-1107.78637221	3.9	
α-D-Gal-TS1a	-1107.70644100	18.9	-1107.75599285	23.0	-209.4
α-D-Gal-IN1	-1107.71852284	11.3	-1107.77212820	12.8	
α-D-Gal-TS1b	-1107.70828290	17.7	-1107.75836533	21.5	-253.3
α-D-Gal-3Ac_a	-1107.73348712	1.9	-1107.78564506	4.4	
α-D-Gal-3Ac_H	-1108.19739134	-27.2	-1108.25098109	-27.5	
α-D-Gal-3Ac_b	-1107.73282441	2.3	-1107.78442963	5.1	
α-D-Gal-TS2a	-1107.71534770	13.3	-1107.76462784	17.5	-215.9
α-D-Gal-IN2	-1107.72802662	5.3	-1107.78073379	7.4	
α-D-Gal-TS2b	-1107.71743456	12.0	-1107.76698693	-3.1	-297.6
α-D-Gal-4Ac_a	-1107.73857198	-1.3	-1107.79147576	-18.5	
α-D-Gal-4Ac_H	-1108.19916501	-28.3	-1108.25223156	-28.3	
α-D-Gal-4Ac_b	-1107.73367345	1.8	-1107.78641161	-15.3	
α-D-Gal-TS3a	-1107.71661175	12.5	-1107.76711026	-3.2	-324.3
α-D-Gal-IN3	-1107.73069558	3.7	-1107.77892743	-10.6	
α-D-Gal-TS3b	-1107.71482413	13.6	-1107.76282637	-0.5	-252.5
α-D-Gal-6Ac	-1107.73915299	-1.6	-1107.79245469	-19.1	
α-D-Gal-6Ac_H	-1108.19753624	-27.3	-1108.25065248	-27.3	

[a] Referred to α-D-Gal-2Ac. For the protonated derivatives (*_H compounds) the values have been calculated considering aqueous phase proton free energy (-265.9 kcal/mol)[42–44]and the free energy change due to changing the standard state from 1 atm to 1 M (1.89 kcal/mol; see Eq. 18) according to Eq. 27. The nomenclature of compounds corresponds to that given in Scheme S7 in which α-D-Glc- configuration must be changed by α-D-Gal-.

Table S28. Calculated (m062x/cc-pvtz/smd=water//m062x/6-31+G(d,p)/smd=water) absolute (hartree) and relative (kcal/mol) energies for acyl migration in Me β-D-Galactopyranoside.[a]

	E_0	ΔE_0	G	ΔG	im. freq
β-D-Gal-2Ac_H	-1108.180181	-24.4	-1108.23296917	-25.8	
β-D-Gal-2Ac	-1107.720527	0.0	-1107.77114740	0.0	
β-D-Gal-TS1a	-1107.698626	13.7	-1107.74871114	14.1	-271.4
β-D-Gal-IN1	-1107.710000	6.6	-1107.76123968	6.2	
β-D-Gal-TS1b	-1107.700389	12.6	-1107.74982926	13.4	-215.8
β-D-Gal-3Ac_a	-1107.718343	1.4	-1107.77042905	0.5	
β-D-Gal-3Ac_H	-1108.181265	-25.1	-1108.23401583	-26.4	
β-D-Gal-3Ac_b	-1107.720126	0.3	-1107.77083219	0.2	
β-D-Gal-TS2a	-1107.70072	12.4	-1107.74993294	13.3	-188.6
β-D-Gal-IN2	-1107.721055	-0.3	-1107.77197718	-0.5	
β-D-Gal-TS2b	-1107.706745	8.6	-1107.75835027	8.0	-271.6
β-D-Gal-4Ac_a	-1107.724368	-2.4	-1107.77612383	-3.1	
β-D-Gal-4Ac_H	-1108.183552	-26.5	-1108.23690342	-28.3	
β-D-Gal-4Ac_b	-1107.724243	-2.3	-1107.77533013	-2.6	
β-D-Gal-TS3a	-1107.701142	12.2	-1107.75179319	12.1	-287.6
β-D-Gal-IN3	-1107.721197	-0.4	-1107.77207912	-0.6	
β-D-Gal-TS3b	-1107.706086	9.1	-1107.75505277	10.1	-241.1
β-D-Gal-6Ac	-1107.724285	-2.4	-1107.77534719	-2.6	
β-D-Gal-6Ac_H	-1108.185621	-27.8	-1108.24001761	-30.2	

[a] Referred to β-D-Gal-2Ac. For the protonated derivatives (*_H compounds) the values have been calculated considering aqueous phase proton free energy (-265.9 kcal/mol)[42–44] and the free energy change due to changing the standard state from 1 atm to 1 M (1.89 kcal/mol; see Eq. 18) according to Eq. 27. The nomenclature of compounds corresponds to that given in Scheme S7 in which α-D-Glc- configuration must be changed by β-D-Gal-.

Table S29. Calculated (m062x/6-311++G(d,p)/smd=water//m062x/6-31+G(d,p)/smd=water) absolute (hartree) and relative (kcal/mol) energies for acyl migration in Me β-D-Galactopyranoside.[a]

	E_0	ΔE_0	G	ΔG	im. freq
β-D-Gal-2Ac_H	-1108.09798	-25.3	-1108.14939761	-25.8	
β-D-Gal-2Ac	-1107.636987	2.3	-1107.68760771	3.6	
β-D-Gal-TS1a	-1107.613835	16.8	-1107.66425653	18.2	-220.1
β-D-Gal-IN1	-1107.62481	9.9	-1107.67702304	10.2	
β-D-Gal-TS1b	-1107.616643	15.0	-1107.66712973	16.4	-239.6
β-D-Gal-3Ac_a	-1107.642389	-1.1	-1107.69332514	0.0	
β-D-Gal-3Ac_H	-1108.096053	-24.1	-1108.14880383	-25.4	
β-D-Gal-3Ac_b	-1107.631981	5.4	-1107.68174416	7.3	
β-D-Gal-TS2a	-1107.621144	12.2	-1107.67079221	14.1	-197.5
β-D-Gal-IN2	-1107.638944	1.0	-1107.68986860	2.2	
β-D-Gal-TS2b	-1107.624677	10.0	-1107.67628229	-5.4	-271.6
β-D-Gal-4Ac_a	-1107.642334	-1.1	-1107.69408975	-16.6	
β-D-Gal-4Ac_H	-1108.098933	-25.9	-1108.15228418	-27.6	
β-D-Gal-4Ac_b	-1107.641003	-0.3	-1107.69209068	-15.3	
β-D-Gal-TS3a	-1107.623848	10.5	-1107.67257039	-3.1	-296.3
β-D-Gal-IN3	-1107.639018	1.0	-1107.68989926	-13.9	
β-D-Gal-TS3b	-1107.623218	10.9	-1107.67218544	-2.8	-241.1
β-D-Gal-6Ac	-1107.641986	-0.9	-1107.69304723	-15.9	
β-D-Gal-6Ac_H	-1108.100944	-27.1	-1108.15534052	-29.5	

[a] Referred to β-D-Gal-2Ac. For the protonated derivatives (*_H compounds) the values have been calculated considering aqueous phase proton free energy (-265.9 kcal/mol)[42–44] and the free energy change due to changing the standard state from 1 atm to 1 M (1.89 kcal/mol; see Eq. 18) according to Eq. 27. The nomenclature of compounds corresponds to that given in Scheme S7 in which α-D-Glc- configuration must be changed by β-D-Gal-.

Table S30. Calculated (wb97xd/6-311++G(d,p)/smd=water//wb97xd/6-31+G(d,p)/smd=water) absolute (hartree) and relative (kcal/mol) energies for acyl migration in Me β-D-Galactopyranoside.[a]

	E_0	ΔE_0	G	ΔG	im. freq
β-D-Gal-2Ac_H	-1108.19806	-27.8	-1108.25038473	-27.7	
β-D-Gal-2Ac	-1107.733111	2.1	-1107.78558994	4.4	
β-D-Gal-TS1a	-1107.706554	18.8	-1107.75760755	21.9	-266.8
β-D-Gal-IN1	-1107.72059	10.0	-1107.77110951	13.5	
β-D-Gal-TS1b	-1107.709302	17.1	-1107.75962782	20.7	-218.3
β-D-Gal-3Ac_a	-1107.7382	-1.0	-1107.79059035	1.2	
β-D-Gal-3Ac_H	-1108.197906	-27.7	-1108.25126076	-28.2	
β-D-Gal-3Ac_b	-1107.733018	2.2	-1107.78480438	4.9	
β-D-Gal-TS2a	-1107.716716	12.4	-1107.76712185	16.0	-213.8
β-D-Gal-IN2	-1107.72981	4.2	-1107.77917958	8.4	
β-D-Gal-TS2b	-1107.717212	12.1	-1107.76763598	-3.5	-263.2
β-D-Gal-4Ac_a	-1107.736631	-0.1	-1107.78812222	-16.4	
β-D-Gal-4Ac_H	-1108.19928	-28.5	-1108.25307907	-29.3	
β-D-Gal-4Ac_b	-1107.733814	1.7	-1107.78783775	-16.2	
β-D-Gal-TS3a	-1107.71634	12.7	-1107.76628544	-2.7	-317.0
β-D-Gal-IN3	-1107.730242	3.9	-1107.78017041	-11.4	
β-D-Gal-TS3b	-1107.707354	18.3	-1107.75487004	4.5	-242.4
β-D-Gal-6Ac	-1107.737244	-0.4	-1107.79073794	-18.0	
β-D-Gal-6Ac_H	-1108.199433	-28.6	-1108.25145347	-28.3	

[a] Referred to β-D-Gal-2Ac. For the protonated derivatives (*_H compounds) the values have been calculated considering aqueous phase proton free energy (-265.9 kcal/mol)[42–44] and the free energy change due to changing the standard state from 1 atm to 1 M (1.89 kcal/mol; see Eq. 18) according to Eq. 27. The nomenclature of compounds corresponds to that given in Scheme S7 in which α-D-Glc- configuration must be changed by β-D-Gal-.

Table S31. Calculated (m062x/cc-pvtz/smd=water//m062x/6-31+G(d,p)/smd=water) absolute (hartree) and relative (kcal/mol) energies for acyl migration in Me α-D-Xylopyranoside.[a]

	E_0	ΔE_0	G	ΔG	im. freq
α-D-Xyl-2Ac_H	-993.6740175	-23.8	-993.72391484	-23.6	
α-D-Xyl-2Ac	-993.2154175	0.0	-993.26564607	0.0	
α-D-Xyl-TS1a	-993.1941685	13.3	-993.24003154	16.1	-271.9
α-D-Xyl-IN1	-993.2009256	9.1	-993.25065501	9.4	
α-D-Xyl-TS1b	-993.196725	11.7	-993.24399794	13.6	-232.4
α-D-Xyl-3Ac_a	-993.2135928	1.1	-993.26371308	1.2	
α-D-Xyl-3Ac_H	-993.6731669	-287.2	-993.72256980	-286.7	
α-D-Xyl-3Ac_b	-993.2161133	-0.4	-993.26562588	0.0	
α-D-Xyl-TS2a	-993.189012	16.6	-993.23617462	18.5	-233.6
α-D-Xyl-IN2	-993.2023474	8.2	-993.25095746	9.2	
α-D-Xyl-TS2b	-993.191992	14.7	-993.23844722	17.1	-270.8
α-D-Xyl-4Ac	-993.2172728	-1.2	-993.26696185	-0.8	
α-D-Xyl-4Ac_H	-993.6684541	-284.3	-993.72054756	-285.5	

[a] Referred to α-D-Xyl-2Ac. For the protonated derivatives (*_H compounds) the values have been calculated considering aqueous phase proton free energy (-265.9 kcal/mol)[42–44] and the free energy change due to changing the standard state from 1 atm to 1 M (1.89 kcal/mol; see Eq. 18) according to Eq. 27. The nomenclature of compounds corresponds to that given in Scheme S7 in which α-D-Glc- configuration must be changed by α-D-Xyl-.

Table S322. Calculated (m062x/6-311++G(d,p)/smd=water//m062x/6-31+G(d,p)/smd=water) absolute (hartree) and relative (kcal/mol) energies for acyl migration in Me α-D-Xylopyranoside.[a]

	E_0	ΔE_0	G	ΔG	im. freq
α-D-Xyl-2Ac_H	-993.5995333	-23.7	-993.64892292	-23.2	
α-D-Xyl-2Ac	-993.1410431	0.0	-993.19127165	0.0	
α-D-Xyl-TS1a	-993.1197033	13.4	-993.16556631	16.1	-271.9
α-D-Xyl-IN1	-993.1272159	8.7	-993.17694531	9.0	
α-D-Xyl-TS1b	-993.122615	11.6	-993.16988791	13.4	-232.4
α-D-Xyl-3Ac_a	-993.1394829	1.0	-993.18960308	1.0	
α-D-Xyl-3Ac_H	-993.6027346	-289.7	-993.65241827	-289.4	
α-D-Xyl-3Ac_b	-993.1422283	-0.7	-993.19174086	-0.3	
α-D-Xyl-TS2a	-993.1159659	15.7	-993.16312854	17.7	-233.6
α-D-Xyl-IN2	-993.1278097	8.3	-993.17641969	9.3	
α-D-Xyl-TS2b	-993.1178809	14.5	-993.16433612	16.9	-270.8
α-D-Xyl-4Ac	-993.1427723	-1.1	-993.19246141	-0.7	
α-D-Xyl-4Ac_H	-993.6004968	-288.3	-993.64994028	-287.8	

[a] Referred to α-D-Xyl-2Ac. For the protonated derivatives (*_H compounds) the values have been calculated considering aqueous phase proton free energy (-265.9 kcal/mol)[42–44] and the free energy change due to changing the standard state from 1 atm to 1 M (1.89 kcal/mol; see Eq. 18) according to Eq. 27. The nomenclature of compounds corresponds to that given in Scheme S7 in which α-D-Glc- configuration must be changed by α-D-Xyl-.

Table S33. Calculated (wb97xd/6-311++G(d,p)/smd=water//wb97xd/6-31+G(d,p)/smd=water) absolute (hartree) and relative (kcal/mol) energies for acyl migration in Me α-D-Xylopyranoside.[a]

	E_0	ΔE_0	G	ΔG	im. freq
α-D-Xyl-2Ac_H	-993.6913525	-26.9	-993.74262301	-26.7	
α-D-Xyl-2Ac	-993.2277122	0.0	-993.27927705	0.0	
α-D-Xyl-TS1a	-993.2040932	14.8	-993.25175908	17.3	-254.3
α-D-Xyl-IN1	-993.2120582	9.8	-993.26251269	10.5	
α-D-Xyl-TS1b	-993.203199	15.4	-993.25201691	17.1	-206.6
α-D-Xyl-3Ac_a	-993.2288607	-0.7	-993.27709373	1.4	
α-D-Xyl-3Ac_H	-993.6915926	-291.1	-993.74065246	-289.5	
α-D-Xyl-3Ac_b	-993.2260801	1.0	-993.27544666	2.4	
α-D-Xyl-TS2a	-993.2007733	16.9	-993.24920280	18.9	-202.5
α-D-Xyl-IN2	-993.2115128	10.2	-993.26266469	10.4	
α-D-Xyl-TS2b	-993.2012564	16.6	-993.24828188	19.4	-263.9
α-D-Xyl-4Ac	-993.2290615	-0.8	-993.27811260	0.7	
α-D-Xyl-4Ac_H	-993.6920857	-291.4	-993.74133349	-289.9	

[a] Referred to α-D-Xyl-2Ac. For the protonated derivatives (*_H compounds) the values have been calculated considering aqueous phase proton free energy (-265.9 kcal/mol)[42–44] and the free energy change due to changing the standard state from 1 atm to 1 M (1.89 kcal/mol; see Eq. 18) according to Eq. 27. The nomenclature of compounds corresponds to that given in Scheme S7 in which α-D-Glc- configuration must be changed by α-D-Xyl-.

Table S34. Calculated (m062x/cc-pvtz/smd=water//m062x/6-31+G(d,p)/smd=water) absolute (hartree) and relative (kcal/mol) energies for acyl migration in Me β-D-Xylopyranoside.[a]

	E_0	ΔE_0	G	ΔG	im. freq
β-D-Xyl-2Ac_H	-993.6736471	-23.5	-993.72363886	-22.8	
β-D-Xyl-2Ac	-993.2154697	0.0	-993.26664911	0.0	
β-D-Xyl-TS1a	-993.1931412	14.0	-993.24036736	16.5	-228.8
β-D-Xyl-IN1	-993.1947661	13.0	-993.24458024	13.8	
β-D-Xyl-TS1b	-993.189671	16.2	-993.23784788	18.1	-252.4
β-D-Xyl-3Ac_a	-993.2115342	2.5	-993.26070660	3.7	
β-D-Xyl-3Ac_H	-993.6719777	-22.5	-993.72126982	-21.3	
β-D-Xyl-3Ac_b	-993.2168587	-0.9	-993.26800607	-0.9	
β-D-Xyl-TS2a	-993.1895556	16.3	-993.23815800	17.9	-226.3
β-D-Xyl-IN2	-993.2001778	9.6	-993.24884711	11.2	
β-D-Xyl-TS2b	-993.1914562	15.1	-993.23866546	17.6	-258.9
β-D-Xyl-4Ac	-993.2179859	-1.6	-993.26610520	0.3	
β-D-Xyl-4Ac_H	-993.6677625	-19.8	-993.71960996	-20.2	

[a] Referred to β-D-Xyl-2Ac. For the protonated derivatives (*_H compounds) the values have been calculated considering aqueous phase proton free energy (-265.9 kcal/mol)[42–44] and the free energy change due to changing the standard state from 1 atm to 1 M (1.89 kcal/mol; see Eq. 18) according to Eq. 27. The nomenclature of compounds corresponds to that given in Scheme S7 in which α-D-Glc- configuration must be changed by β-D-Xyl-.

Table S35. Calculated (m062x/6-311++G(d,p)/smd=water//m062x/6-31+G(d,p)/smd=water) absolute (hartree) and relative (kcal/mol) energies for acyl migration in Me β-D-Xylopyranoside.[a]

	E_0	ΔE_0	G	ΔG	im. freq
β-D-Xyl-2Ac_H	-993.5993543	-23.5	-993.64998656	-23.1	
β-D-Xyl-2Ac	-993.141227	0.0	-993.19240639	0.0	
β-D-Xyl-TS1a	-993.1193255	13.7	-993.16655171	16.2	-228.8
β-D-Xyl-IN1	-993.1261375	9.5	-993.17595168	10.3	
β-D-Xyl-TS1b	-993.1187889	14.1	-993.16691586	16.0	-237.4
β-D-Xyl-3Ac_a	-993.1372869	2.5	-993.18645936	3.7	
β-D-Xyl-3Ac_H	-993.6002105	-24.0	-993.64936610	-22.7	
β-D-Xyl-3Ac_b	-993.142516	-0.8	-993.19366331	-0.8	
β-D-Xyl-TS2a	-993.1152577	16.3	-993.16373751	18.0	-245.1
β-D-Xyl-IN2	-993.1258553	9.6	-993.17457384	11.2	
β-D-Xyl-TS2b	-993.1180528	14.5	-993.16496241	17.2	-234.7
β-D-Xyl-4Ac	-993.1432173	-1.2	-993.19133665	0.7	
β-D-Xyl-4Ac_H	-993.5913257	-18.4	-993.64317316	-18.9	

[a] Referred to β-D-Xyl-2Ac. For the protonated derivatives (*_H compounds) the values have been calculated considering aqueous phase proton free energy (-265.9 kcal/mol)[42–44] and the free energy change due to changing the standard state from 1 atm to 1 M (1.89 kcal/mol; see Eq. 18) according to Eq. 27. The nomenclature of compounds corresponds to that given in Scheme S7 in which α-D-Glc- configuration must be changed by β-D-Xyl-.

Table S36. Calculated (wb97xd/6-311++G(d,p)/smd=water//wb97xd/6-31+G(d,p)/smd=water) absolute (hartree) and relative (kcal/mol) energies for acyl migration in Me β-D-Xylopyranoside.[a]

	E_0	ΔE_0	G	ΔG	im. freq
β-D-Xyl-2Ac_H	-993.6907547	-26.5	-993.74130899	-27.4	
β-D-Xyl-2Ac	-993.2278601	0.0	-993.27686143	0.0	
β-D-Xyl-TS1a	-993.2038229	15.1	-993.25036900	16.6	-236.4
β-D-Xyl-IN1	-993.2111774	10.5	-993.26097828	10.0	
β-D-Xyl-TS1b	-993.2026475	15.8	-993.25079811	16.4	-248.6
β-D-Xyl-3Ac_a	-993.2257549	1.3	-993.27558971	0.8	
β-D-Xyl-3Ac_H	-993.6854582	-23.1	-993.73540730	-23.7	
β-D-Xyl-3Ac_b	-993.2289334	-0.7	-993.27916754	-1.4	
β-D-Xyl-TS2a	-993.2000545	17.4	-993.24950450	17.2	-238.4
β-D-Xyl-IN2	-993.2105823	10.8	-993.25907970	11.2	
β-D-Xyl-TS2b	-993.2023998	16.0	-993.24902876	17.5	-227.6
β-D-Xyl-4Ac	-993.2300593	-1.4	-993.27883560	-1.2	
β-D-Xyl-4Ac_H	-993.6901819	-26.1	-993.74046008	-26.9	

[a] Referred to β-D-Xyl-2Ac. For the protonated derivatives (*_H compounds) the values have been calculated considering aqueous phase proton free energy (-265.9 kcal/mol)[42–44] and the free energy change due to changing the standard state from 1 atm to 1 M (1.89 kcal/mol; see Eq. 18) according to Eq. 27. The nomenclature of compounds corresponds to that given in Scheme S7 in which α-D-Glc- configuration must be changed by β-D-Xyl-.

pKa values

Table S37. Calculated pK_a values (Eq. 27-29) for the different species involved in acetyl migration of Me α-D-glucopyranoside and Me β-D-glucopyranoside.[a]

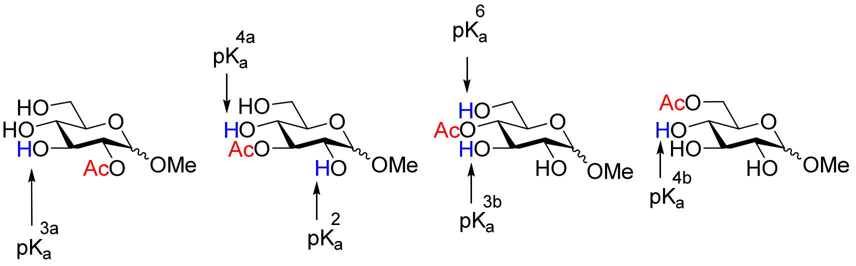

α/β-D-**Glc-2Ac_H** α/β-D-**Glc-3Ac_H** α/β-D-**Glc-4Ac_H** α/β-D-**Glc-6Ac_H**

	Me α-D-glucopyranoside			Me β-D-glucopyranoside		
	Level A	Level B	Level C	Level A	Level B	Level C
pK_a^{3a}	13.4	12.9	12.2	12.9	11.9	14.3
pK_a^{2}	13.2	12.0	10.4	13.5	12.5	12.9
pK_a^{4a}	13.7	12.6	13.1	10.7	9.4	13.9
pK_a^{3b}	12.2	11.0	11.7	12.5	12.5	12.7
pK_a^{6}	13.5	11.6	11.4	13.0	13.0	13.6
pK_a^{4b}	11.7	11.1	13.5	13.1	12.2	12.4

[a] Calculated directly in solution and considering three explicit molecules of water.
Level A: m062x/cc-pvtz/smd=water//m062x/6-31+G(d,p)/smd=water.
Level B: m062x/6-311++G(d,p)/smd=water//m062x/6-31+G(d,p)/smd=water.
Level C: wb97xd/6-311++G(d,p)/smd=water//wb97xd/6-31+G(d,p)/smd=water.

Table S38. Calculated pK_a values (Eq. 27-29) for the different species involved in acetyl migration of Me α-D-glucopyranoside and Me β-D-galactopyranoside.[a]

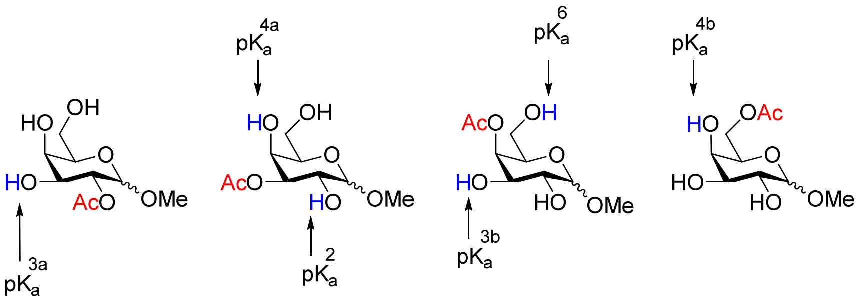

α/β-D-**Gal-2Ac_H** α/β-D-**Gal-3Ac_H** α/β-D-**Gal-4Ac_H** α/β-D-**Gal-6Ac_H**

	Me α-D-galactopyranoside			Me β-D-galactopyranoside		
	Level A	Level B	Level C	Level A	Level B	Level C
pK_a^{3a}	12.2	14.2	15.7	14.3	14.3	15.6
pK_a^{2}	12.9	13.3	15.9	15.1	11.4	13.8
pK_a^{4a}	13.4	14.1	16.5	14.9	16.7	16.4
pK_a^{3b}	13.2	11.2	13.8	13.8	12.6	15.7
pK_a^{6}	14.3	12.5	14.7	12.8	12.1	14.5
pK_a^{4b}	11.8	10.1	11.2	14.2	13.1	12.4

[a] Calculated directly in solution and considering three explicit molecules of water.
Level A: m062x/cc-pvtz/smd=water//m062x/6-31+G(d,p)/smd=water.
Level B: m062x/6-311++G(d,p)/smd=water//m062x/6-31+G(d,p)/smd=water.
Level C: wb97xd/6-311++G(d,p)/smd=water//wb97xd/6-31+G(d,p)/smd=water.

TableS39. Calculated pK_a values (Eq. 27-29) for the different species involved in acetyl migration of Me α-D-xylopyranoside and Me β-D-xylopyranoside.[a]

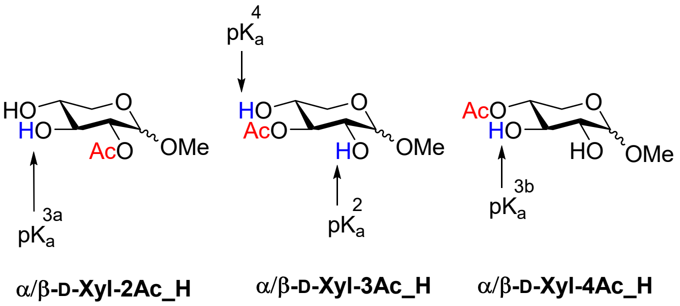

	Me α-D-xylopyranoside			Me β-D-xylopyranoside		
	Level A	Level B	Level C	Level A	Level B	Level C
pK_a^{3a}	12.6	12.4	15.0	12.1	12.3	15.5
pK_a^{2}	12.9	14.7	15.1	13.7	14.8	13.4
pK_a^{4}	12.0	13.8	15.8	10.3	11.5	11.7
pK_a^{3b}	10.5	12.3	14.9	10.5	9.7	14.2

[a] Calculated directly in solution and considering three explicit molecules of water.
Level A: m062x/cc-pvtz/smd=water//m062x/6-31+G(d,p)/smd=water.
Level B: m062x/6-311++G(d,p)/smd=water//m062x/6-31+G(d,p)/smd=water.
Level C: wb97xd/6-311++G(d,p)/smd=water//wb97xd/6-31+G(d,p)/smd=water.

Constant values

(see Scheme S7)

k_1 and k_2 correspond to pK_a^{3a} (see Eq.13 and Tables 37-39)

k_5 and k_6 correspond to pK_a^{2} (see Eq.14 and Tables 37-39)

k_9 and k_{10} correspond to pK_a^{4a} (see Eq.15 and Tables 37-39)

k_{13} and k_{14} correspond to pK_a^{3b} (see Eq.16 and Tables 37-39)

k_{17} and k_{18} correspond to pK_a^{6} (see Eq.17 and Tables 37-39)

k_{21} and k_{22} correspond to pK_a^{4b} (see Eq.18 and Tables 37-39)

$k_7 \approx 0$ ($3.6 \cdot 10^{-11}$ for Me α-D-Glucopyranoside)

$k_8 \approx 0$ ($4.8 \cdot 10^{-10}$ for Me α-D-Glucopyranoside)

$k_{15} \approx 0$ ($1.3 \cdot 10^{-11}$ for Me α-D-Glucopyranoside)

$k_{16} \approx 0$ ($1.6 \cdot 10^{-14}$ for Me α-D-Glucopyranoside)

$k_{23} \approx 0$ ($1.1 \cdot 10^{-9}$ for Me α-D-Glucopyranoside)

$k_{24} \approx 0$ ($1.5 \cdot 10^{-10}$ for Me α-D-Glucopyranoside)

	Me α-D-glucopyranoside			Me β-D-glucopyranoside		
	Level A	Level B	Level C	Level A	Level B	Level C
k_3	8.80E+01	8.67E+01	2.29E-02	2.32E+00	2.32E+00	2.35E-01
k_4	6.82E+00	6.24E+00	3.01E-02	1.35E+02	1.35E+02	2.67E-01
k_{11}	1.42E+00	2.76E+00	2.91E+01	1.95E-01	9.11E-02	8.69E+00
k_{12}	6.49E-02	1.60E-01	1.83E-02	1.66E-01	1.66E-01	3.82E-03
k_{19}	2.19E+02	8.50E+01	8.57E-01	5.80E+02	5.64E+03	6.39E+01
k_{20}	4.01E-01	8.92E-01	5.81E-01	4.95E+01	4.82E+02	1.15E+00
	Me α-D-galactopyranoside			Me β-D-galactopyranoside		
	Level A	Level B	Level C	Level A	Level B	Level C
k_3	4.75E+00	1.20E+01	6.09E-02	2.27E+02	1.07E+02	7.46E-01
k_4	1.38E+01	3.22E+01	1.31E-01	4.86E+02	2.52E-01	3.73E-03
k_{11}	1.85E+03	2.56E+03	4.46E+03	1.51E+03	5.67E+07	2.89E+04
k_{12}	4.37E+00	7.74E-01	2.56E+00	5.56E+00	1.19E+02	8.59E+02
k_{19}	8.59E+02	2.25E+04	8.69E+01	8.96E+01	2.60E+03	4.24E-03
k_{20}	1.50E+00	6.51E+01	1.44E-01	8.80E+01	9.44E+02	1.96E-04
	Me α-D-xylopyranoside			Me β-D-xylopyranoside		
	Level A	Level B	Level C	Level A	Level B	Level C
k_3	1.01E+01	9.20E+00	7.74E-01	3.27E-01	4.72E+00	2.47E+00
k_4	7.81E+01	5.39E+01	7.82E+00	1.77E+02	2.57E+03	9.50E+00
k_{11}	1.61E-01	3.34E-01	1.44E+00	7.29E-02	8.35E-02	5.29E-02
k_{12}	3.92E-02	1.56E-01	8.54E-02	5.46E-01	9.82E-01	7.52E-02

Level A: m062x/cc-pvtz/smd=water//m062x/6-31+G(d,p)/smd=water.
Level B: m062x/6-311++G(d,p)/smd=water//m062x/6-31+G(d,p)/smd=water.
Level C: wb97xd/6-311++G(d,p)/smd=water//wb97xd/6-31+G(d,p)/smd=water.

$k_a^1, k_{-a}^1, k_b^1, k_{-b}^1, k_a^2, k_{-a}^2, k_b^2, k_{-b}^2, k_a^3, k_{-a}^3, k_b^3$ and k_{-b}^3correspond to the energy barriers collected in Tables S17-36.

Transition Structures

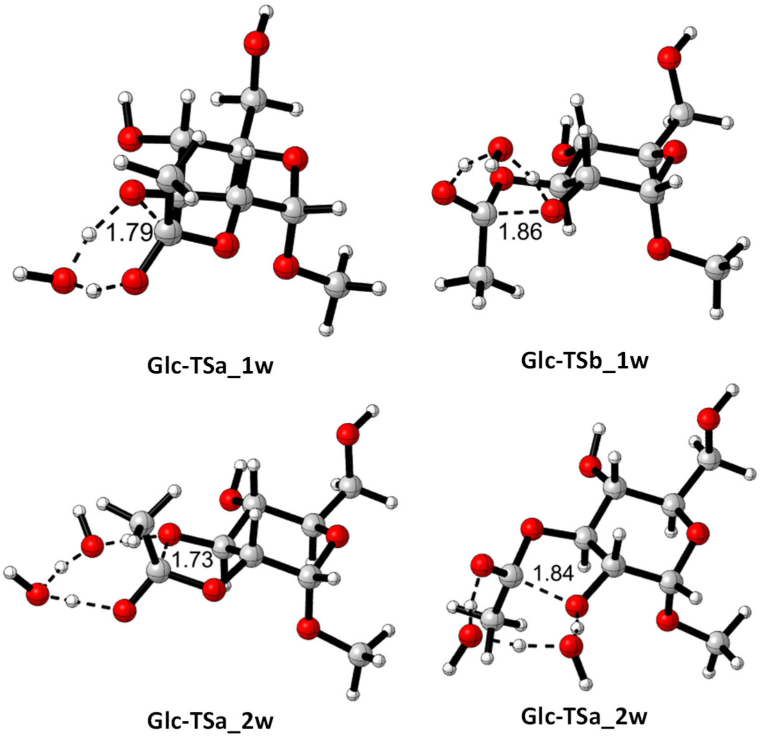

Figure S59. Optimized geometries (tppsh-gd3bj/def2tzvpd/cpcm=water) of the transition structures corresponding to acyl migration in Me α-D-Glucopyranoside from position 2 to position 3 under a neutral mechanism mediated by one (**1w**) and two (**2w**) molecules of water (Scheme S4).

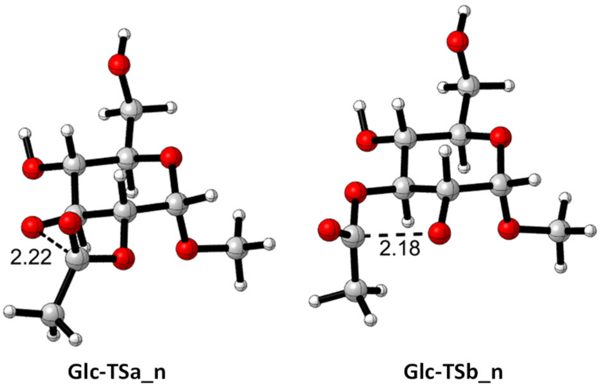

Figure S60. Optimized geometries (tppsh-gd3bj/def2tzvpd/cpcm=water) of the transition structures corresponding to acyl migration in Me α-D-Glucopyranoside from position 2 to position 3 under an anionic mechanism (Scheme S5).

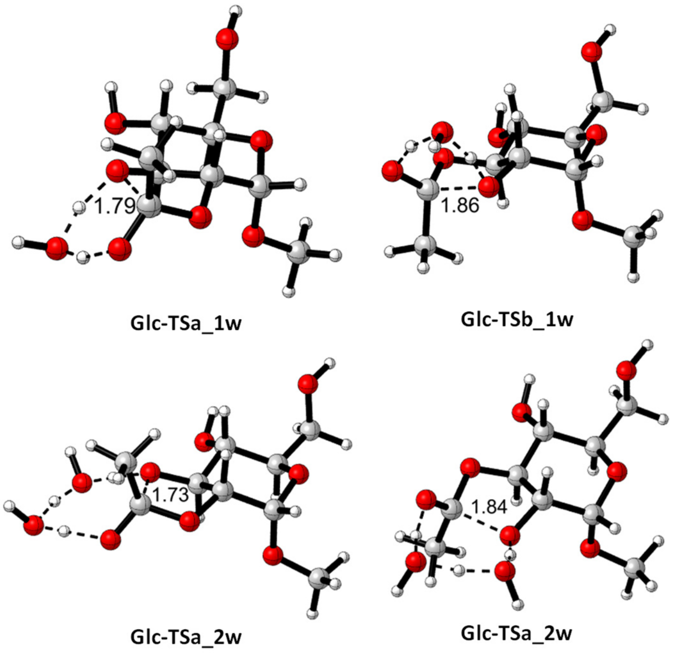

Figure S61. Optimized geometries (tppsh-gd3bj/def2tzvpd/cpcm=water) of the transition structures corresponding to acyl migration in Me α-D-Glucopyranoside from position 2 to position 3 through a naked anion including one (**n1w**) and two (**n2w**) explicit molecules of water surrounding the anion (Scheme S6). Transition structures with three water molecules are illustrated in the next Figure.

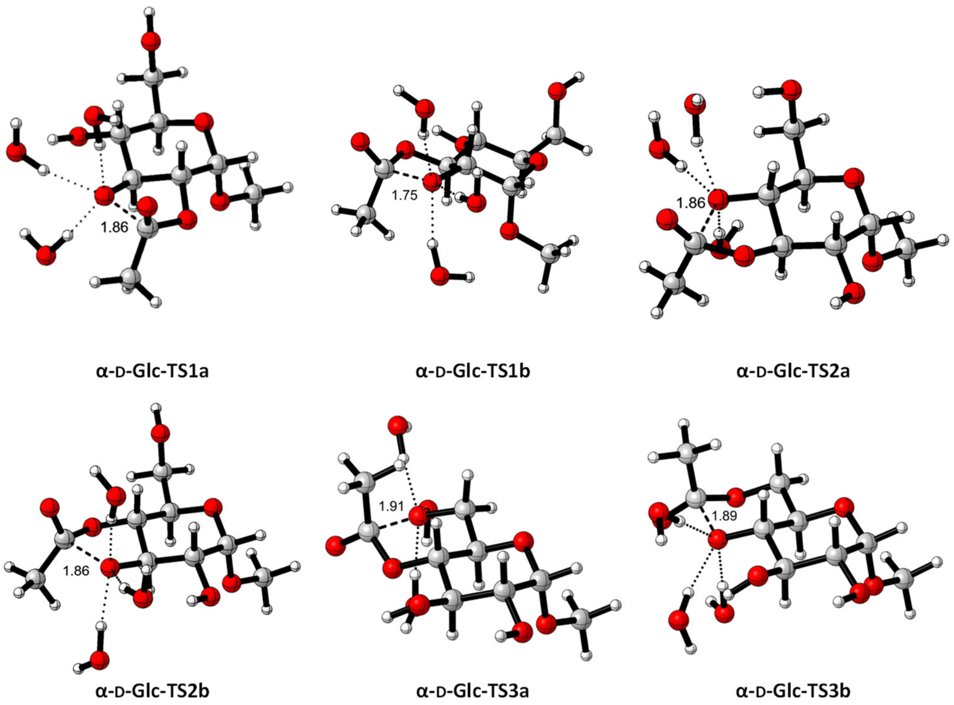

Figure S62. Optimized geometries (wb97xd/6-31+G(d,p)/SMD=water) of the transition structures corresponding to acyl migrations in Me α-D-glucopyranoside (Scheme S7).

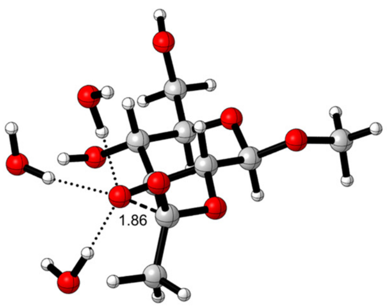

β-D-Glc-TS1a

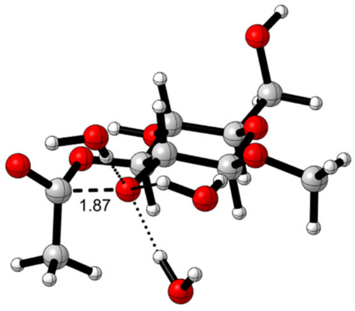

β-D-Glc-TS1b

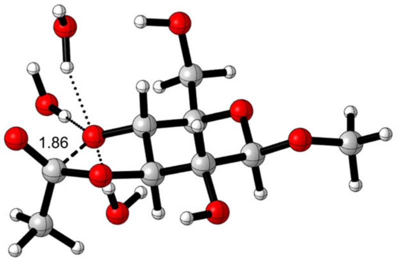

β-D-Glc-TS2a

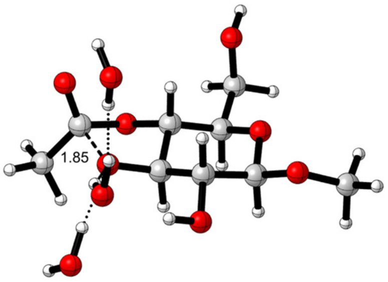

β-D-Glc-TS2b

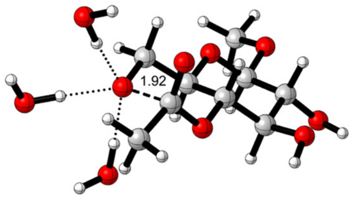

β-D-Glc-TS3a

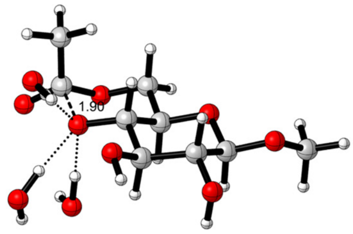

β-D-Glc-TS3b

Figure S63. Optimized geometries (m062x/6-31+G(d,p)/SMD=water) of the transition structures corresponding to acyl migrations in Me β-D-glucopyranoside (Scheme S7).

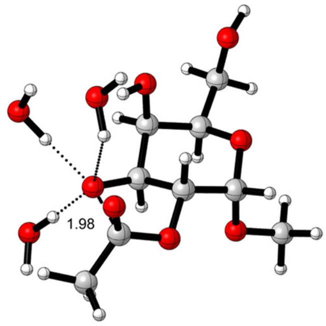

α-D-Gal-TS1a

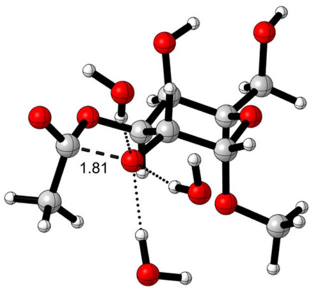

α-D-Gal-TS1b

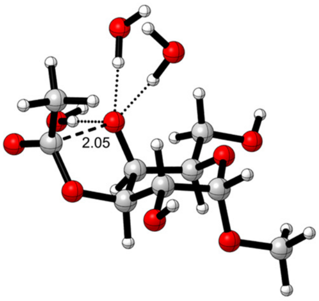

α-D-Gal-TS2a

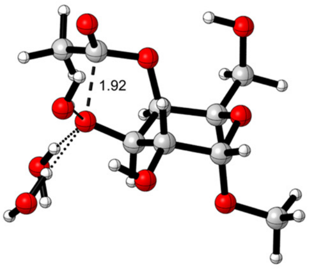

α-D-Gal-TS2b

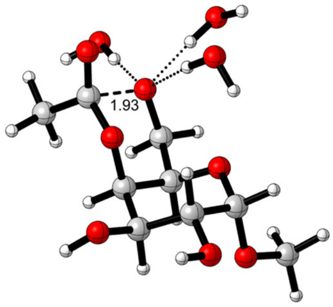

α-D-Gal-TS3a

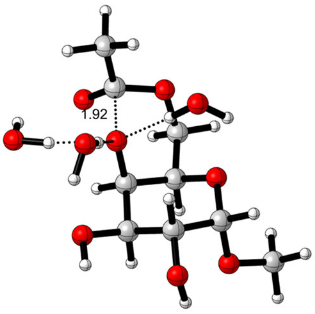

α-D-Gal-TS3b

Figure S64. Optimized geometries (m062x/6-31+G(d,p)/SMD=water) of the transition structures corresponding to acyl migrations in Me α-D-galactopyranoside (Scheme S7).

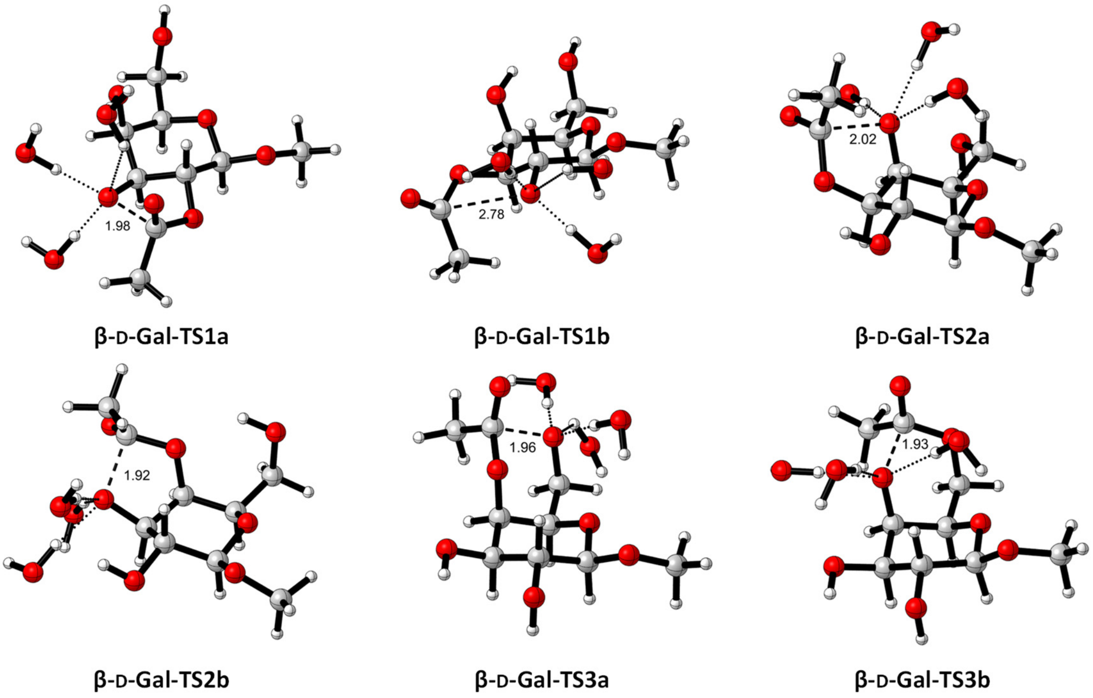

Figure S65. Optimized geometries (m062x/6-31+G(d,p)/SMD=water) of the transition structures corresponding to acyl migrations in Me β-D-galactopyranoside (Scheme S7).

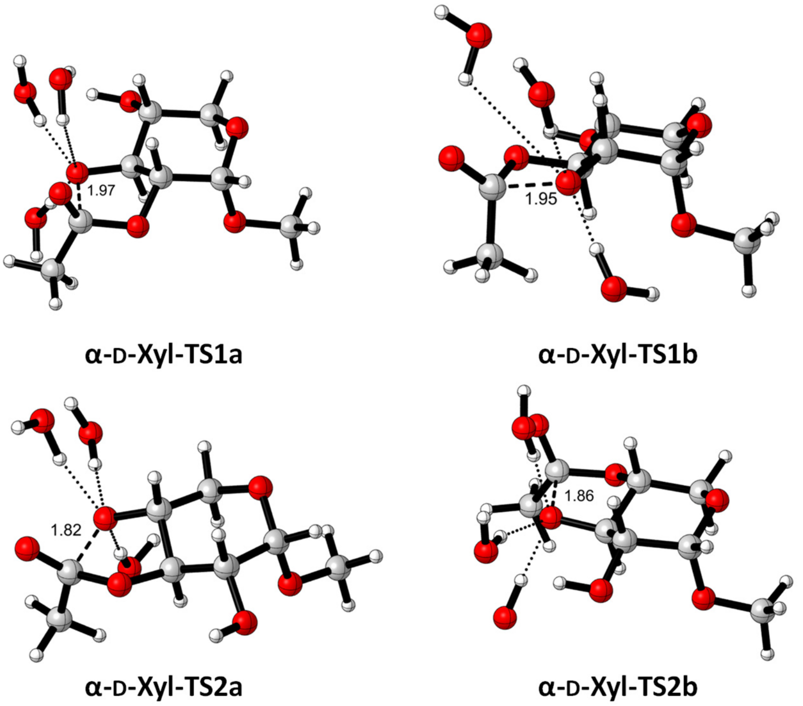

Figure S66. Optimized geometries (m062x/6-31+G(d,p)/SMD=water) of the transition structures corresponding to acyl migrations in Me α-D-xylopyranoside (Scheme S7).

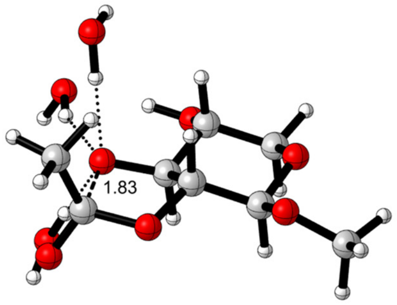

β-D-Xyl-TS1a

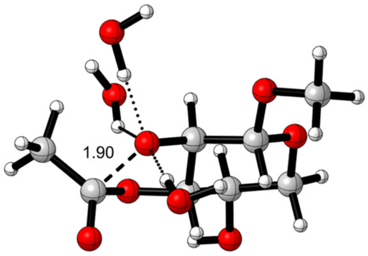

β-D-Xyl-TS1b

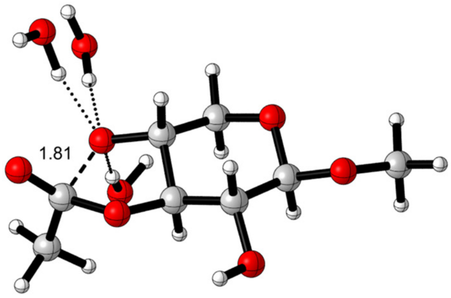

β-D-Xyl-TS2a

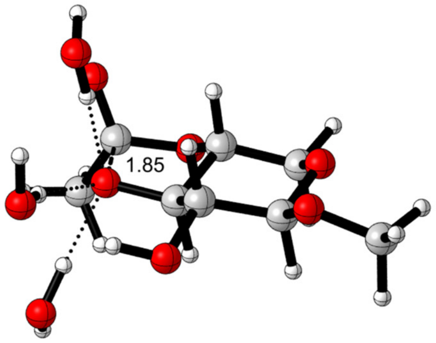

β-D-Xyl-TS2b

Figure S67. Optimized geometries (m062x/6-31+G(d,p)/SMD=water) of the transition structures corresponding to acyl migrations in Me β-D-xylopyranoside (Scheme S7).

Cartesian Coordinates

Neutral mechanism with a one water molecules bridge

alpha-D-Glc-2Ac-1w

0 1

C	-2.2361748296	-0.3363753038	-0.2389668198
C	-1.2412675877	-1.5007637652	-0.4230855250
C	0.1533417608	-0.9548871624	-0.7252698093
C	0.5284279506	0.0860573943	0.3391207620
C	-0.5259570415	1.1908563408	0.4520111074
H	-1.1961530785	-2.0952810966	0.5013575491
H	-2.3259382212	0.1921073839	-1.2018818489
H	0.6086397047	-0.4107639895	1.3125002437
H	-0.3222608739	1.8625578553	1.3002433972
H	0.1270518190	-0.4562172988	-1.7084014766
O	-1.7657771806	0.5810918044	0.7630574907
O	-0.5538563615	1.9040849331	-0.7481263251
O	1.8091939358	0.6130154715	-0.0572258059
O	1.0899917010	-2.0125687468	-0.7278766057
O	-1.6470016580	-2.3155524748	-1.5190809191
H	-2.2410894101	-3.0071779835	-1.1987228541
C	-3.6280006789	-0.7881206829	0.1656270012
H	-4.2436701264	0.1060507094	0.3509552318
H	-4.0723520674	-1.3439276240	-0.6758658144
C	-1.3522399981	3.0874455864	-0.7044937083
H	-1.2505015250	3.5692653433	-1.6822568388
H	-2.4083405106	2.8440866464	-0.5190290057
H	-0.9888367547	3.7655990597	0.0838641578
O	-3.5464628479	-1.6051973037	1.3300087253
H	-4.4403769142	-1.8542272330	1.5998663528
C	2.8790890977	0.6734672437	0.7545298279
O	3.4423166267	-1.0552753510	-1.9156724270
H	3.7482227601	-0.3362587483	-1.3234202610
H	1.9221404172	-1.7031334361	-1.1561744323
H	4.1863855492	-1.6705007773	-1.9791878008
O	3.9698726204	0.8066117927	0.2193812363
C	2.6863092521	0.5820772098	2.2426540944
H	3.6115023008	0.8971481578	2.7326142875
H	1.8486280731	1.2039396576	2.5822990228
H	2.4760445568	-0.4596454827	2.5257927998

alpha-D-Glc-3Ac-1w

0 1

C	-2.0725447864	-0.0895939663	-0.3322010753
C	-0.8476048999	-1.0191742582	-0.1991800675
C	0.4147476603	-0.1678490188	-0.1660606910
C	0.3267843618	0.8962583525	0.9361462615
C	-0.9344582233	1.7384436795	0.7218807780
H	-0.9233784996	-1.5839889944	0.7409813343
H	-2.0157936836	0.4201541139	-1.3079294013
H	0.1916578533	0.3774379925	1.8996678335
H	-1.0954453781	2.4225243776	1.5688506844
H	0.5757179884	0.3157126161	-1.1361289638
O	-2.0705242758	0.8861037325	0.7194436475
O	-0.8011607324	2.4506488285	-0.4743035423
O	1.4694623694	1.7179247614	0.9694335217
O	1.5588334389	-0.9816576462	0.1781891389
O	-0.7583493750	-1.9057150295	-1.3096330643
H	-1.2582652855	-2.7112598564	-1.1197997189

C	-3.3951831979	-0.8335091286	-0.2786138177
H	-4.2100891918	-0.0927148588	-0.2800298620
H	-3.4872064622	-1.4494660166	-1.1877519550
C	-1.8132593664	3.4348268699	-0.6829482153
H	-1.5653422143	3.9467789549	-1.6185934468
H	-2.8063293499	2.9696462808	-0.7675321342
H	-1.8196490049	4.1598602400	0.1466279890
O	-3.4375274285	-1.6433238933	0.8925242772
H	-4.2938211961	-2.0891579657	0.9346116488
C	2.5764016393	-1.2433088375	-0.6577050020
O	3.6159686014	-1.6432067974	-0.1504624771
C	2.3911628742	-1.0432310234	-2.1350305788
H	2.4272734749	0.0308877045	-2.3697729813
H	3.2108791196	-1.5422816320	-2.6591343016
H	1.4211468815	-1.4355510342	-2.4632508423
O	3.5487838886	0.2027134910	2.0529502115
H	2.2157054368	1.2088953288	1.3665535841
H	3.6710530849	-0.5427539566	1.4268299270
H	4.4162648784	0.6257315897	2.1223623013

alpha-D-Glc-IN1-1w
0 1

C	-2.1140203565	-0.4527798340	-0.2286358884
C	-1.0258217993	-1.4721365877	0.2250157033
C	0.2831551366	-0.7332616879	0.0834972478
C	0.2484191930	0.5272978475	0.9229683551
C	-0.7908375369	1.4989700426	0.3871366389
H	-1.1931912910	-1.7654595107	1.2709567611
H	-1.9564918012	-0.2649436155	-1.3036422427
H	-0.0636434929	0.2808033166	1.9519257150
H	-0.9260712669	2.3833869087	1.0274892611
H	0.4383501825	-0.4802807138	-0.9779678841
O	-2.0311843608	0.7952830975	0.4864379556
O	-0.4747928590	1.8694915999	-0.9181290946
O	1.6127708659	0.9125397555	0.9113853416
O	1.4546587329	-1.3602366828	0.6056087532
O	-1.0310695986	-2.6130126916	-0.6260405495
H	-1.6897820196	-3.2457867041	-0.3090647226
C	-3.5286245751	-0.9772595177	-0.0564470124
H	-4.2333440417	-0.1768241780	-0.3309750013
H	-3.6775144502	-1.8189998120	-0.7522613920
C	-1.3083692995	2.8988703019	-1.4515826827
H	-0.9251754678	3.1254854063	-2.4519009999
H	-2.3528347467	2.5623652604	-1.5221343637
H	-1.2555802280	3.8004135659	-0.8206599459
O	-3.7207286028	-1.3908702273	1.2931897388
H	-4.6319971097	-1.6939607191	1.4007944189
C	2.3857862051	-0.3090109440	0.9433676365
O	2.6234030243	0.2323343783	-2.5686394825
H	3.0637898630	-0.0489368213	-0.8738715424
H	2.4563450732	-0.5751873422	-3.0770833285
H	3.3143127278	0.6977307546	-3.0620729751
O	3.4129020944	-0.2148887719	0.0362538967
C	2.9605605561	-0.5749074778	2.3213763360
H	3.4637452972	-1.5498225654	2.3272385159
H	3.6877957398	0.2064333093	2.5720126162
H	2.1568439425	-0.5804002201	3.0665449268

alpha-D-Glc-TSa-1w
0 1

C	-2.1177398144	-0.2378838777	-0.2651529058
C	-0.9611044230	-1.2630508530	-0.1066210016
C	0.3226615818	-0.4755955472	-0.2932471302
C	0.3604914677	0.6548517520	0.7284649081
C	-0.7757209354	1.6379794909	0.4938392680
H	-0.9899756545	-1.7133113607	0.8960884914
H	-2.0914750312	0.1321862352	-1.3033789994
H	0.2112580693	0.2457671494	1.7397490707
H	-0.8409695669	2.4060552074	1.2789296361
H	0.3248002885	-0.0460268204	-1.3089335525
O	-1.9731410664	0.8760687414	0.6370551060
O	-0.6442937043	2.2198996837	-0.7659986659
O	1.6777570089	1.1628744040	0.5927110679
O	1.5412514184	-1.1577727607	-0.0550067095
O	-1.0438206612	-2.2671033939	-1.1118940905
H	-1.6530868763	-2.9600638364	-0.8229370867
C	-3.4909312724	-0.8410555856	-0.0288285258
H	-4.2394349834	-0.0347121354	-0.0763030522
H	-3.7033306921	-1.5549121396	-0.8413478511
C	-1.5722814209	3.2752346843	-1.0208100580
H	-1.3303922679	3.6720032429	-2.0121144649
H	-2.6059196741	2.9003882689	-1.0135168460
H	-1.4652057518	4.0701967689	-0.2658111185
O	-3.5139832759	-1.4904442104	1.2389750699
H	-4.4026180447	-1.8353390884	1.3977709869
C	2.6010755193	0.1071536032	0.6474204750
O	2.9863416278	-1.1213604907	-1.9655795491
H	3.4605809089	-0.3866006770	-1.2185936723
H	2.2117681472	-1.3385857867	-1.1694182009
H	3.5650081126	-1.8902341001	-2.0862787572
O	3.6214554308	0.2585629882	-0.1226460740
C	2.8550488133	-0.4475884381	2.0318029810
H	3.3549260019	-1.4188440792	1.9461984115
H	3.5254679945	0.2513858323	2.5512925769
H	1.9369207258	-0.5642578717	2.6171692627

alpha-D-Glc-TSb-1w
0 1

C	-2.1832918768	-0.1964034366	-0.2306566285
C	-1.0908058792	-1.2916108597	-0.3927632991
C	0.2108933765	-0.5461427601	-0.5705266872
C	0.4608497357	0.3725968743	0.6238245882
C	-0.6346569367	1.4374336573	0.6725293723
H	-1.0508274477	-1.9209946835	0.5074346941
H	-2.2469892061	0.3460987243	-1.1885352052
H	0.3445279228	-0.2167389804	1.5497581485
H	-0.5685510286	2.0672941463	1.5722134857
H	0.1612049531	0.0429145786	-1.4984544354
O	-1.8669045380	0.7323995539	0.8202858684
O	-0.5889037855	2.2130972279	-0.4856696730
O	1.7931073185	0.7930036743	0.4732146538
O	1.3825246165	-1.3613141709	-0.5738462086
O	-1.3467721386	-2.0800048638	-1.5492964534
H	-1.9513433175	-2.7986843258	-1.3192860350
C	-3.5590663201	-0.7677906319	0.0631631963
H	-4.2550097588	0.0694895072	0.2287443188
H	-3.9015473268	-1.3290085208	-0.8214376286
C	-1.4557456901	3.3472832854	-0.4661359539
H	-1.2879344163	3.8867682761	-1.4040683851
H	-2.5087482275	3.0373810295	-0.4004112900

H -1.2128034149 4.0008755947 0.3867501801
O -3.4893433634 -1.6124591083 1.2078530805
H -4.3735136682 -1.9515187868 1.4006431271
C 2.5235071533 -0.5713375704 -0.5498302021
O 3.5088793617 -1.0688129061 0.1099898080
C 2.8496718488 0.1729347304 -1.8227252043
H 1.9665720834 0.6097042314 -2.2990751384
H 3.5728588499 0.9653072517 -1.6018754412
H 3.3121249563 -0.5452891707 -2.5150016946
O 3.1655448187 -0.0713974666 2.2480107874
H 2.4931670959 0.5163087077 1.5714104175
H 3.4644415232 -0.7078973233 1.3070464696
H 3.9120577266 0.4800725145 2.5286533675

Neutral mechanism with one water molecules bridge

alpha-D-Glc-2Ac-2w
0 1
C -2.4929465802 -0.0548433670 -0.3649925287
C -1.7051600577 -1.2024522448 0.2733732245
C -0.2075965100 -1.0212210810 0.0172275282
C 0.2144284244 0.3527821979 0.5410355167
C -0.6401342576 1.4535420692 -0.1095386885
H -1.8798729100 -1.1893856536 1.3628549330
H -2.3374470483 -0.0790199007 -1.4559159276
H 0.0635472907 0.3886466605 1.6253889189
H -0.4496461734 2.4373916768 0.3465359690
H -0.0264321152 -1.0577338520 -1.0686866384
O -2.0057671116 1.1929738450 0.1600309151
O -0.3519111231 1.4785685749 -1.4758566252
O 1.6090743945 0.5310013248 0.2249975896
O 0.4702667894 -2.0783405659 0.6654780711
O -2.1461722696 -2.4346916794 -0.2779308347
H -1.4765069195 -3.0951057137 -0.0349973531
C -3.9888277140 -0.1155834397 -0.1152818848
H -4.4523736003 0.7799762601 -0.5590156434
H -4.3845674724 -1.0056538429 -0.6294801457
C -0.9178243512 2.5905835582 -2.1707901244
H -0.5816910016 2.5132458801 -3.2097949207
H -2.0162317286 2.5615603823 -2.1331232691
H -0.5610478058 3.5366908751 -1.7338152990
O -4.2414607551 -0.1799386015 1.2846706686
H -5.1969121439 -0.1963271807 1.4277228275
C 2.4989154042 1.0787936036 1.0632292645
O 2.6138075662 -2.7174582959 -0.8489550366
H 3.2776611858 -1.9954099194 -1.0089518242
H 1.2866266882 -2.3073548662 0.1512939070
H 3.1233736611 -3.4880302751 -0.5632863576
O 3.6685838806 1.1022610787 0.7022188402
C 2.0442313328 1.6238839844 2.3885598470
H 2.8889953862 2.1314702715 2.8609341801
H 1.2079051660 2.3251419027 2.2718710758
H 1.7096394692 0.8018454353 3.0374842785
H 4.3208825132 -0.2924223251 -2.1238737057
O 4.3756571880 -0.6931223166 -1.2449143055
H 4.1229503085 0.0223985401 -0.6107924426

alpha-D-Glc-3Ac-2w
0 1
C -1.2158427118 1.2878138437 0.5237474506

C	0.1157489811	0.5582141838	0.3163402977
C	-0.0843554647	-0.9273711754	0.0331122303
C	-1.0977900937	-1.1520534620	-1.0892369282
C	-2.3691675805	-0.3309507512	-0.8164577426
H	0.5963838065	0.9871166234	-0.5758733964
H	-1.6937283423	0.9302110675	1.4504369970
H	-0.6600021547	-0.8044135475	-2.0336641018
H	-3.0320713509	-0.3452866943	-1.6944467626
H	-0.3991224758	-1.4585615166	0.9395240532
O	-2.0815887665	1.0311657555	-0.5941072938
O	-2.9945657734	-0.9466394206	0.2829363541
O	-1.4147849038	-2.5215755874	-1.2620925117
O	1.1939643017	-1.4242312399	-0.4292863996
O	0.9636115481	0.7015706804	1.4431095698
C	-1.0182784089	2.8004669605	0.6382855911
H	-2.0041461156	3.2817007557	0.6069120943
H	-0.5662798740	3.0130996349	1.6198199449
C	-4.3397879843	-0.5258629990	0.5152334913
H	-4.7078131735	-1.1065614228	1.3672882467
H	-4.3772131325	0.5467285548	0.7528628993
H	-4.9615051702	-0.7287076789	-0.3708152648
O	-0.2464343208	3.3639822320	-0.4123839808
H	0.6993093823	3.1583360385	-0.2686873327
C	2.1347511714	-1.9011656797	0.3997316563
O	3.2973315910	-1.8484467324	0.0170365343
C	1.7215764192	-2.5192741799	1.7058751382
H	0.8946827920	-3.2268197683	1.5592773512
H	2.5843191376	-3.0332668556	2.1381294857
H	1.3851659202	-1.7319293125	2.3933613922
O	2.4877158545	2.5348685645	0.1144902447
H	1.6244528903	1.3923634452	1.2150029809
H	3.0167795881	1.8890195159	-0.4285190808
O	3.7235331095	0.5931887633	-1.3092147014
H	3.6077592097	-0.3039956594	-0.9169635142
H	4.6679824903	0.6746247680	-1.5010315362
H	3.1193270996	3.1647837260	0.4864787972
H	-2.0472504954	-2.7530944302	-0.5607882534

alpha-D-Glc-IN1-2w

0 1

C	-2.3456979530	-0.6274455328	-0.4135719354
C	-1.0105402932	-1.3225677981	-0.0070514787
C	0.0255142515	-0.2282557157	-0.0732745513
C	-0.3650974061	0.8999283007	0.8584284559
C	-1.6387107581	1.5703315604	0.3652355154
H	-1.0881138329	-1.7226373520	1.0136759717
H	-2.2468975889	-0.3237950286	-1.4688190258
H	-0.5954912681	0.4955133377	1.8574844620
H	-2.0231472282	2.3315889534	1.0600823237
H	0.1086868350	0.1363344086	-1.1088077807
O	-2.6247343652	0.5351579808	0.3895910367
O	-1.4368192105	2.1048358660	-0.9045998606
O	0.8289085328	1.6687606078	0.9213208442
O	1.3352631794	-0.5144523272	0.4309044379
O	-0.6829955401	-2.3524558137	-0.9310635741
H	-1.1236474482	-3.1716714138	-0.6671113286
C	-3.5482264913	-1.5488918948	-0.3119703700
H	-4.4537283031	-0.9668952047	-0.5443695516
H	-3.4457284382	-2.3419412411	-1.0704835842
C	-2.5259551065	2.8922318214	-1.3882180388

H -2.2196873063 3.2846038544 -2.3633460171
H -3.4326740867 2.2808529347 -1.5024951899
H -2.7306747997 3.7270453412 -0.6993405122
O -3.6138125719 -2.1019348192 0.9989290926
H -4.3967920622 -2.6647942925 1.0621818294
C 1.9275136721 0.7897758580 0.6788526652
O 2.5553849695 1.2769625098 -0.4506974662
C 2.8338884205 0.6801562050 1.8915695069
H 2.2595276647 0.3379345729 2.7600731275
H 3.6459981167 -0.0305788031 1.6930697677
H 3.2671417279 1.6646136872 2.1026353299
O 3.1046285335 -2.4562653685 -0.5197664826
H 2.3823329470 -1.8878542315 -0.1692889546
H 4.2903206313 -1.1991119208 -0.8276753920
O 4.6892990431 -0.3099261289 -1.0078153699
H 5.4622417897 -0.2387450520 -0.4305999977
H 3.3495650056 0.7200888447 -0.6715539237
H 3.3115567379 -3.0785257055 0.1918460191

alpha-D-Glc-TSa-2w
0 1
C -2.3525447659 -0.5629065121 -0.2743156791
C -1.0262349707 -1.3036332863 0.0479663080
C 0.0834409657 -0.2932346461 -0.1741204735
C -0.1687800011 0.9187815878 0.7111696319
C -1.4672979146 1.6146201668 0.3327049451
H -1.0282519333 -1.6410681761 1.0945430579
H -2.3330407747 -0.2974030970 -1.3444213398
H -0.2894685439 0.5850200263 1.7555985930
H -1.7350870276 2.4232805744 1.0295090392
H 0.0727531423 0.0129370033 -1.2346312165
O -2.4934186349 0.6382759297 0.5080581988
O -1.3880543189 2.0916004198 -0.9758731332
O 1.0180840510 1.6748019613 0.5701860290
O 1.3845338477 -0.6663513709 0.2213147254
O -0.8392188204 -2.4069195725 -0.8339777922
H -1.3186450079 -3.1740630572 -0.4925603918
C -3.5905169446 -1.4091936647 -0.0368709529
H -4.4799109135 -0.7829747841 -0.2091408033
H -3.6002878891 -2.2289970900 -0.7736236838
C -2.4975785737 2.8989012247 -1.3694222969
H -2.2881221509 3.2455637834 -2.3867246012
H -3.4308126669 2.3172794382 -1.3611264580
H -2.6003213713 3.7649033684 -0.6960075284
O -3.5721354026 -1.9202842847 1.2931757495
H -4.3825131979 -2.4243479494 1.4450594427
C 2.1536405465 0.8188441674 0.6489271885
O 3.0555427913 1.1320875992 -0.2006908443
C 2.5722492297 0.5172550779 2.0789935846
H 1.7164882755 0.2612189972 2.7143058900
H 3.2828658892 -0.3186753311 2.0880749849
H 3.0699336508 1.4086365558 2.4848303315
O 2.8735674626 -2.0308608714 -1.2017759344
H 2.1582247493 -1.4801728465 -0.6082126184
H 3.8661501171 -1.4067242072 -1.0692497056
O 4.7343820872 -0.5214622677 -0.9046787648
H 5.3831658853 -0.7710530570 -0.2299863370
H 4.1006525392 0.2498058217 -0.5068522090
H 2.9666975941 -2.9223036314 -0.8320869362

alpha-D-Glc-TSb-2w
0 1

C	2.5752795597	-0.1018823742	0.0664517585
C	1.6024689774	-1.3128121487	0.0324299119
C	0.2604350451	-0.7843877519	0.4827693922
C	-0.1888482673	0.3729741080	-0.4050623057
C	0.8121501426	1.5230373828	-0.2794620247
H	1.5295574927	-1.7075034293	-0.9909537503
H	2.6861633287	0.2012075956	1.1210064460
H	-0.1363129754	0.0497976338	-1.4612834252
H	0.6028575501	2.3403850786	-0.9863859326
H	0.3387684540	-0.4435338295	1.5275852519
O	2.0771556688	1.0093625625	-0.6965434343
O	0.8296091920	1.9967266613	1.0337362979
O	-1.5070041211	0.5958399184	-0.0069774861
O	-0.8131553756	-1.7120922651	0.3504485005
O	2.0399664655	-2.3269419304	0.9323836371
H	2.6908027138	-2.8878280896	0.4889555140
C	3.9598608684	-0.4335245636	-0.4614965562
H	4.5619286443	0.4886753520	-0.4633942085
H	4.4351171756	-1.1521110693	0.2260464635
C	1.6062502092	3.1793576673	1.2173660102
H	1.4938833823	3.4701124556	2.2671918672
H	2.6675526889	2.9935448040	0.9964456927
H	1.2347993314	3.9876931363	0.5670777996
O	3.8513977776	-0.9783857859	-1.7733559813
H	4.7383698671	-1.1653980313	-2.1082459999
C	-2.0527200226	-1.0830587686	0.5058846444
O	-2.9379788886	-1.5226070025	-0.2931735562
C	-2.4349699045	-0.7628993989	1.9375385465
H	-1.5956963239	-0.3554070308	2.5109442426
H	-3.2583195833	-0.0389221621	1.9480935078
H	-2.7775921492	-1.6940947924	2.4109653613
O	-3.2268849155	1.6099418330	-1.4611176116
H	-2.4152485275	1.2199926586	-0.8645196679
H	-3.4283370319	2.5068691207	-1.1547568680
O	-4.8317118482	-0.1186627229	-0.9467999896
H	-4.0822825579	-0.7959441355	-0.5540172449
H	-5.4676209790	0.0972554470	-0.2480266359
H	-4.1477480639	0.8180238673	-1.2102841667

Naked anion

alpha-D-Glc-2Ac-n
-1 1

C	-1.9832960000	0.2417220000	-0.1621340000
C	-1.4139660000	-1.1831060000	-0.2656650000
C	0.0687340000	-1.1845960000	-0.7309810000
C	0.8042220000	-0.1895560000	0.2123460000
C	0.1456030000	1.1845310000	0.3566980000
H	-1.4630570000	-1.6659500000	0.7235240000
H	-1.9281430000	0.7137330000	-1.1577170000
H	0.8611810000	-0.6378250000	1.2118620000
H	0.6103880000	1.7699460000	1.1675100000
H	0.0526040000	-0.6975600000	-1.7435860000
O	-1.1999400000	1.0151280000	0.7648230000
O	0.2482350000	1.8874560000	-0.8547370000
O	2.1545720000	-0.0028520000	-0.2725260000
O	0.6499080000	-2.4086570000	-0.7292380000
O	-2.1804780000	-1.9379380000	-1.2123790000

H	-2.9912860000	-2.2402600000	-0.7815990000
C	-3.4327760000	0.3135670000	0.2860420000
H	-3.6980440000	1.3699060000	0.4523610000
H	-4.0698340000	-0.0762520000	-0.5247720000
C	-0.1623750000	3.2483250000	-0.7651860000
H	0.0294790000	3.7022030000	-1.7436690000
H	-1.2336680000	3.3260730000	-0.5264710000
H	0.4195890000	3.7776910000	0.0075960000
O	-3.6133330000	-0.4496330000	1.4791580000
H	-4.5246330000	-0.3405850000	1.7816500000
C	3.1910150000	-0.3048900000	0.5212870000
O	3.0863020000	-0.6785080000	1.6781020000
C	4.5013310000	-0.1370600000	-0.2046330000
H	4.6615750000	-1.0140170000	-0.8492190000
H	4.4800620000	0.7514470000	-0.8474160000
H	5.3206950000	-0.0721430000	0.5176840000

alpha-D-Glc-3Ac-n
-1 1

C	-1.4460240000	0.9074220000	0.2658830000
C	0.0553020000	1.0511030000	-0.0406410000
C	0.7209260000	-0.3214230000	-0.0339280000
C	-0.0001710000	-1.3622200000	-0.9379370000
C	-1.4968580000	-1.3433040000	-0.5236080000
H	0.1683340000	1.4900270000	-1.0449160000
H	-1.5457670000	0.5578980000	1.3079490000
H	-0.0217810000	-0.8814030000	-1.9551360000
H	-2.0844560000	-1.9459490000	-1.2351900000
H	0.7968130000	-0.7104020000	0.9911020000
O	-2.0583030000	-0.0334790000	-0.6165520000
O	-1.6358510000	-1.8615240000	0.7759060000
O	0.5614430000	-2.5902420000	-0.9274500000
O	2.0849570000	-0.2116910000	-0.5639010000
O	0.6164820000	1.9459370000	0.9245300000
C	-2.2040860000	2.2195770000	0.1612400000
H	-3.2674290000	2.0251990000	0.3765280000
H	-1.8101260000	2.9072090000	0.9254390000
C	-2.9816600000	-2.1070620000	1.1629050000
H	-3.5645810000	-1.1737940000	1.2046730000
H	-3.4673430000	-2.8012930000	0.4562630000
H	-2.9506690000	-2.5624350000	2.1595590000
O	-2.0470000000	2.7697010000	-1.1458940000
H	-2.5124500000	3.6155210000	-1.1836670000
C	3.0860300000	0.1024290000	0.2524060000
O	2.9236960000	0.4943000000	1.4050620000
C	4.4323830000	-0.0913550000	-0.3879630000
H	4.7073870000	-1.1531740000	-0.3039690000
H	4.3959570000	0.1634990000	-1.4535430000
H	5.1830460000	0.5120240000	0.1314080000
H	1.4146160000	1.5220550000	1.2997660000

alpha-D-Glc-INa-n
-1 1

C	1.8792650000	-0.1796340000	0.3081640000
C	0.8040450000	-1.3004890000	0.2697370000
C	-0.5342580000	-0.5994730000	0.3634830000
C	-0.6642910000	0.4017540000	-0.7765230000
C	0.3997430000	1.4864300000	-0.6597000000
H	0.8742370000	-1.8584650000	-0.6754240000
H	1.8179510000	0.2979540000	1.3008760000

H	-0.4364580000	-0.1196090000	-1.7283520000
H	0.4198050000	2.1610240000	-1.5302090000
H	-0.5767340000	-0.0631530000	1.3310130000
O	1.6629300000	0.8162980000	-0.7076390000
O	0.2272100000	2.2173910000	0.5199230000
O	-2.0074930000	0.7800940000	-0.7208250000
O	-1.6831690000	-1.3918390000	0.1825960000
O	0.9723270000	-2.1823850000	1.3806530000
H	1.6498360000	-2.8377000000	1.1651690000
C	3.2975960000	-0.6969000000	0.1420430000
H	3.9816550000	0.1653760000	0.1013660000
H	3.5582000000	-1.2992700000	1.0277350000
C	1.0847820000	3.3501790000	0.6323010000
H	0.8132050000	3.8618810000	1.5620140000
H	2.1409630000	3.0450620000	0.6740600000
H	0.9373910000	4.0320180000	-0.2213400000
O	3.3884520000	-1.4784310000	-1.0471850000
H	4.3045270000	-1.7632330000	-1.1633910000
C	-2.8235190000	-0.4800350000	-0.2384010000
O	-3.5406780000	-0.9964200000	-1.1502500000
C	-3.5265640000	0.0020740000	1.0362440000
H	-2.8168200000	0.3637050000	1.7931400000
H	-4.2143930000	0.8208250000	0.7820940000
H	-4.1107800000	-0.8275180000	1.4589700000

alpha-D-Glc-TSa-n
-1 1

C	-1.9318550000	-0.1153860000	-0.2540130000
C	-0.9262110000	-1.2883740000	-0.3261110000
C	0.4742290000	-0.7202830000	-0.6109460000
C	0.7438250000	0.3180810000	0.5019640000
C	-0.2724680000	1.4453280000	0.5489400000
H	-0.9129340000	-1.8296330000	0.6330200000
H	-1.9391150000	0.3799950000	-1.2400010000
H	0.7036220000	-0.1868420000	1.4800280000
H	-0.1380240000	2.0997820000	1.4257250000
H	0.3754260000	-0.1519290000	-1.5703250000
O	-1.5486550000	0.8454600000	0.7485580000
O	-0.2139820000	2.2043160000	-0.6283380000
O	2.0771740000	0.7569380000	0.2636020000
O	1.5248160000	-1.5898460000	-0.6190970000
O	-1.2973660000	-2.1796000000	-1.3836190000
H	-2.0106760000	-2.7535790000	-1.0730810000
C	-3.3600100000	-0.5354890000	0.0475560000
H	-3.9745230000	0.3704190000	0.1711470000
H	-3.7505830000	-1.0987050000	-0.8160030000
C	-1.0260310000	3.3745200000	-0.6018020000
H	-0.8472880000	3.9051730000	-1.5434510000
H	-2.0924000000	3.1159780000	-0.5213340000
H	-0.7449430000	4.0216820000	0.2456000000
O	-3.3910830000	-1.3362460000	1.2285890000
H	-4.3113960000	-1.5401000000	1.4414140000
C	2.9794890000	-0.2902890000	0.4346840000
O	3.1020080000	-0.8480710000	1.5244180000
C	4.0752120000	-0.2582330000	-0.6026950000
H	3.6903440000	0.0127730000	-1.5907410000
H	4.8257930000	0.4888660000	-0.2964190000
H	4.5663210000	-1.2367460000	-0.6439560000

alpha-D-Glc-TSb-n

-1 1
C 1.8384910000 -0.2921640000 0.3159100000
C 0.6596820000 -1.2908670000 0.2141220000
C -0.6142510000 -0.4762580000 0.2451150000
C -0.6437380000 0.5869790000 -0.8728380000
C 0.5775980000 1.5071110000 -0.6803270000
H 0.7241220000 -1.8470600000 -0.7322590000
H 1.7782970000 0.1908160000 1.3065070000
H -0.3977520000 0.0444850000 -1.8219330000
H 0.6994050000 2.1992640000 -1.5293290000
H -0.6993410000 0.0191770000 1.2254950000
O 1.7669200000 0.7107240000 -0.7052630000
O 0.4624660000 2.2269800000 0.5173540000
O -1.8813820000 1.1490260000 -0.8827650000
O -1.7657650000 -1.2801010000 -0.0021000000
O 0.6787490000 -2.1965510000 1.3215520000
H 1.2964170000 -2.9158170000 1.1311560000
C 3.2025920000 -0.9536490000 0.2216020000
H 3.9725360000 -0.1659540000 0.2278530000
H 3.3510250000 -1.5850900000 1.1130300000
C 1.4466420000 3.2414880000 0.6840680000
H 1.2102040000 3.7655230000 1.6170520000
H 2.4571460000 2.8102660000 0.7504980000
H 1.4138270000 3.9540440000 -0.1571030000
O 3.2797040000 -1.7336430000 -0.9698730000
H 4.1624790000 -2.1218800000 -1.0311960000
C -2.9613950000 -0.5776180000 -0.1089610000
O -3.7762960000 -0.9926220000 -0.9352420000
C -3.4119590000 0.1760070000 1.1248030000
H -2.5844520000 0.5791260000 1.7160070000
H -4.0692730000 0.9987220000 0.8231790000
H -3.9918010000 -0.5222930000 1.7507780000

Naked anion with one explicit water molecule

alpha-D-Glc-2Ac-n1w
-1 1
C 2.1631170000 0.1906310000 0.1640680000
C 1.3491630000 -1.0573530000 0.4975790000
C -0.0636510000 -0.6889320000 0.9766600000
C -0.6864750000 0.1653010000 -0.1355570000
C 0.1831270000 1.3712920000 -0.4938900000
H 1.2566450000 -1.6793390000 -0.4047570000
H 2.2582350000 0.8103970000 1.0661180000
H -0.8116040000 -0.4337840000 -1.0392760000
H -0.1997410000 1.8833110000 -1.3849260000
H 0.0590640000 -0.0384870000 1.8637410000
O 1.4755000000 0.9310700000 -0.8557160000
O 0.2063310000 2.2533460000 0.5949860000
O -1.9717020000 0.6411090000 0.2964860000
O -0.7979650000 -1.8223580000 1.2931290000
O 2.0140310000 -1.7924640000 1.5168580000
H 1.3276890000 -2.3631200000 1.8953460000
C 3.5641550000 -0.0794430000 -0.3377550000
H 4.0436170000 0.8777610000 -0.5752780000
H 4.1336990000 -0.5605980000 0.4652610000
C 0.8995770000 3.4673840000 0.3270330000
H 0.7252380000 4.1229350000 1.1809130000
H 1.9749450000 3.2934670000 0.2160460000
H 0.5124370000 3.9417660000 -0.5830590000

O	3.5197050000	-0.9129700000	-1.4921690000
H	4.4255560000	-1.0806410000	-1.7729570000
C	-3.0736020000	0.2773570000	-0.3684480000
O	-3.0612110000	-0.4137440000	-1.3762170000
C	-4.3098230000	0.8063890000	0.2864740000
H	-4.4471830000	0.2971760000	1.2450030000
H	-4.2039250000	1.8749590000	0.4869060000
H	-5.1743850000	0.6245590000	-0.3503920000
O	-1.7004270000	-3.0532290000	-0.7985410000
H	-2.1912010000	-2.3543040000	-1.2505030000
H	-1.3167160000	-2.5778820000	0.0143070000

alpha-D-Glc-3Ac-n1w
-1 1

C	-2.0032110000	-0.5559710000	-0.2072910000
C	-0.6605380000	-1.2672860000	0.0281920000
C	0.4733900000	-0.2805630000	-0.1874460000
C	0.3099080000	0.9901230000	0.6639380000
C	-1.0880090000	1.5699400000	0.3906100000
H	-0.6175020000	-1.6380500000	1.0585060000
H	-2.0578890000	-0.2613740000	-1.2641840000
H	0.2661560000	0.6479990000	1.7173270000
H	-1.3087290000	2.3785200000	1.0982250000
H	0.5294130000	-0.0203100000	-1.2460840000
O	-2.0983270000	0.6022060000	0.6231290000
O	-1.1327410000	2.0601910000	-0.9242470000
O	1.3224500000	1.9070010000	0.4623250000
O	1.6904650000	-0.9338990000	0.2297140000
O	-0.4931230000	-2.3426680000	-0.8868850000
H	-0.9311050000	-3.1237280000	-0.5307210000
C	-3.2113060000	-1.4223380000	0.0747910000
H	-4.1150740000	-0.8288670000	-0.1053290000
H	-3.2098440000	-2.2681040000	-0.6228640000
C	-2.3303320000	2.7618610000	-1.2334110000
H	-2.1935020000	3.2000340000	-2.2231180000
H	-3.1943870000	2.0888140000	-1.2536320000
H	-2.5108210000	3.5604690000	-0.5029950000
O	-3.1748960000	-1.8823770000	1.4221690000
H	-3.9641050000	-2.4119860000	1.5781870000
C	2.8077460000	-0.9514240000	-0.4986420000
O	3.8027900000	-1.4366690000	0.0270420000
C	2.8061150000	-0.4145550000	-1.8976390000
H	2.5623330000	0.6508850000	-1.8986350000
H	3.7964010000	-0.5618030000	-2.3256670000
H	2.0623780000	-0.9354240000	-2.5069830000
O	3.3431900000	1.3870690000	1.9428680000
H	3.6505890000	0.5011190000	1.7230940000
H	2.5346560000	1.5562420000	1.3373310000

alpha-D-Glc-IN-n1w
-1 1

C	-2.0606610000	-0.2332810000	-0.3535150000
C	-0.8493410000	-1.1192490000	-0.7539860000
C	0.3411110000	-0.1945620000	-0.7481940000
C	0.4882530000	0.4209830000	0.6252720000
C	-0.7018770000	1.3045040000	0.9476940000
H	-0.7064410000	-1.9266230000	-0.0271030000
H	-2.2088320000	0.5031120000	-1.1552150000
H	0.4850110000	-0.3756130000	1.3828650000
H	-0.6881330000	1.6772320000	1.9778470000

H	0.1810580000	0.5876710000	-1.5047680000
O	-1.8422460000	0.4567640000	0.8881310000
O	-0.7661460000	2.3684160000	0.0442980000
O	1.7587970000	1.0218760000	0.5520120000
O	1.6171910000	-0.7738950000	-0.9240300000
O	-1.0352140000	-1.6532080000	-2.0559880000
H	-1.5964320000	-2.4347270000	-1.9945460000
C	-3.3540660000	-1.0042260000	-0.2088480000
H	-4.1506090000	-0.3048240000	0.0696530000
H	-3.6106200000	-1.4461860000	-1.1788910000
C	-1.8155940000	3.2907000000	0.3205700000
H	-1.6899840000	4.1248680000	-0.3703530000
H	-2.7970290000	2.8321490000	0.1619330000
H	-1.7467660000	3.6563790000	1.3522450000
O	-3.2053450000	-2.0152580000	0.7821760000
H	-4.0453830000	-2.4790780000	0.8648390000
C	2.6043370000	0.1600740000	-0.3414040000
O	3.5203010000	-0.4978460000	0.3242490000
C	3.1288940000	1.0763240000	-1.4401850000
H	2.3128490000	1.5482090000	-1.9948590000
H	3.7485860000	1.8599170000	-0.9950140000
H	3.7432400000	0.4943700000	-2.1326520000
O	2.6214820000	-2.2828130000	2.0623680000
H	2.9085540000	-1.5870430000	1.4037050000
H	1.8240350000	-1.9297110000	2.4701700000

alpha-D-Glc-TSa-n1w
-1 1

C	2.0163330000	-0.2448960000	0.3439130000
C	0.7896660000	-1.1571210000	0.5175570000
C	-0.4294210000	-0.2553150000	0.6665330000
C	-0.5054420000	0.6303990000	-0.5682000000
C	0.7228330000	1.5057180000	-0.7220530000
H	0.6744540000	-1.7970910000	-0.3668740000
H	2.1319630000	0.3544200000	1.2574920000
H	-0.5615030000	0.0054410000	-1.4689140000
H	0.7383330000	2.0403870000	-1.6784580000
H	-0.2559760000	0.3826760000	1.5511240000
O	1.8415570000	0.6334690000	-0.7797940000
O	0.8004120000	2.4140770000	0.3390420000
O	-1.7216370000	1.3381650000	-0.3988060000
O	-1.6743360000	-0.8713170000	0.7471690000
O	1.0085900000	-1.9598760000	1.6714660000
H	0.2090580000	-2.4803860000	1.8169110000
C	3.3182170000	-0.9855780000	0.1318100000
H	4.1219320000	-0.2505240000	0.0052030000
H	3.5327790000	-1.5794720000	1.0268710000
C	1.8609000000	3.3548500000	0.1980880000
H	1.7528670000	4.0756860000	1.0091810000
H	2.8366300000	2.8646020000	0.2771370000
H	1.7889620000	3.8744350000	-0.7650830000
O	3.2233470000	-1.8222080000	-1.0168020000
H	4.0613340000	-2.2867110000	-1.1166750000
C	-2.7828070000	0.4265360000	-0.2035630000
O	-3.2301050000	-0.1800500000	-1.1966130000
C	-3.6793570000	0.8724980000	0.9205890000
H	-3.1066810000	1.1847320000	1.7945750000
H	-4.2770170000	1.7202000000	0.5669230000
H	-4.3576910000	0.0614290000	1.1908290000
O	-1.8898140000	-3.0604910000	-0.7415510000

H	-2.2274910000	-2.7635680000	-1.5934620000
H	-1.7916030000	-2.2229460000	-0.2077060000

alpha-D-Glc-TSb-n1w
-1 1

C	-2.1390880000	-0.2105190000	-0.1837870000
C	-1.0874550000	-1.3408400000	-0.0582410000
C	0.2348510000	-0.7265600000	-0.4420860000
C	0.5530070000	0.4647860000	0.4545970000
C	-0.5209790000	1.5318430000	0.2653170000
H	-1.0463950000	-1.7111840000	0.9720430000
H	-2.2042560000	0.0630050000	-1.2459840000
H	0.4455590000	0.1378720000	1.5043120000
H	-0.4135930000	2.3571550000	0.9788610000
H	0.1824340000	-0.4129380000	-1.4923830000
O	-1.7757670000	0.9442930000	0.5843710000
O	-0.4756340000	2.0194480000	-1.0467510000
O	1.8653820000	0.8028360000	0.1654790000
O	1.3492250000	-1.5835160000	-0.2521630000
O	-1.3919650000	-2.4024000000	-0.9521430000
H	-2.0423060000	-2.9813570000	-0.5387570000
C	-3.5276110000	-0.6201420000	0.2552500000
H	-4.2016930000	0.2340830000	0.1241590000
H	-3.8759390000	-1.4348650000	-0.3904140000
C	-1.3896530000	3.0816410000	-1.2951420000
H	-1.1676820000	3.4612620000	-2.2932400000
H	-2.4255390000	2.7277690000	-1.2640320000
H	-1.2581690000	3.8860160000	-0.5608630000
O	-3.5034930000	-1.0346290000	1.6178590000
H	-4.4018110000	-1.2691660000	1.8749870000
C	2.5717220000	-0.8951490000	-0.3861340000
O	3.4666630000	-1.2542680000	0.4082130000
C	2.9324640000	-0.5111810000	-1.8039610000
H	2.0885070000	-0.1212960000	-2.3733650000
H	3.7243660000	0.2394500000	-1.7829650000
H	3.3132430000	-1.4081830000	-2.3046010000
O	3.1206080000	1.2635000000	2.4453370000
H	3.6220660000	0.4592180000	2.6172700000
H	2.6575120000	1.0877670000	1.5784700000

Naked anion with two explicit water molecules

alpha-D-Glc-2Ac-n2w
-1 1

C	2.0850870000	-0.6104660000	-0.0829750000
C	1.4024730000	0.7506130000	-0.2519100000
C	-0.0445390000	0.5914190000	-0.7623670000
C	-0.7488260000	-0.4093010000	0.1644620000
C	0.0091740000	-1.7243980000	0.3137240000
H	1.3683210000	1.2656410000	0.7189500000
H	2.1677030000	-1.0840050000	-1.0708020000
H	-0.8515010000	0.0245560000	1.1605990000
H	-0.4451430000	-2.3569990000	1.0857820000
H	0.0236130000	0.1325650000	-1.7656070000
O	1.3111580000	-1.4502870000	0.7828440000
O	0.0114460000	-2.3939550000	-0.9172880000
O	-2.0552120000	-0.6920300000	-0.3636250000
O	-0.7424610000	1.7960340000	-0.8157560000
O	2.1969620000	1.4944790000	-1.1664310000
H	1.8269260000	2.3966900000	-1.2211350000

C	3.4836570000	-0.5453170000	0.4907880000
H	3.8653640000	-1.5685330000	0.5891280000
H	4.1267190000	-0.0028740000	-0.2102490000
C	0.5911400000	-3.6927900000	-0.8566000000
H	0.4222630000	-4.1578160000	-1.8284320000
H	1.6677530000	-3.6379810000	-0.6640210000
H	0.1114400000	-4.2935210000	-0.0742660000
O	3.4654950000	0.1014830000	1.7598780000
H	4.3629150000	0.0948700000	2.1102030000
C	-3.1438080000	-0.3715750000	0.3446420000
O	-3.1066760000	0.1120250000	1.4661760000
C	-4.3976740000	-0.6694270000	-0.4144680000
H	-4.4615480000	0.0083390000	-1.2709730000
H	-4.3718990000	-1.6921800000	-0.7974860000
H	-5.2650310000	-0.5283540000	0.2287740000
O	0.6953920000	3.8580160000	-1.2610900000
H	0.7839830000	4.3116070000	-0.4157370000
H	0.0740750000	3.0659050000	-1.0834850000
O	-1.4770580000	2.6542020000	1.5565440000
H	-2.0549550000	1.9375460000	1.8502750000
H	-1.1634740000	2.3322550000	0.6566700000

alpha-D-Glc-3Ac-n2w
-1 1

C	-2.1708000000	-0.4440910000	-0.2679930000
C	-0.9145340000	-1.2811960000	0.0208290000
C	0.3071770000	-0.3781560000	-0.0312850000
C	0.1647880000	0.8236290000	0.9178330000
C	-1.1602840000	1.5369550000	0.6026760000
H	-0.9875850000	-1.7150760000	1.0247150000
H	-2.0997760000	-0.0519540000	-1.2918330000
H	0.0251950000	0.4029040000	1.9311140000
H	-1.3774510000	2.2899500000	1.3701360000
H	0.4723680000	-0.0260560000	-1.0515410000
O	-2.2548780000	0.6386170000	0.6600230000
O	-1.0578570000	2.1508640000	-0.6548130000
O	1.2497830000	1.6790170000	0.8757540000
O	1.4508780000	-1.1273210000	0.4270770000
O	-0.7556010000	-2.3102150000	-0.9455570000
H	-1.3088670000	-3.0583320000	-0.6944610000
C	-3.4629560000	-1.2266400000	-0.1811840000
H	-4.2973300000	-0.5431820000	-0.3767230000
H	-3.4600330000	-1.9982370000	-0.9601010000
C	-2.1839480000	2.9494300000	-0.9966940000
H	-1.9209980000	3.4932360000	-1.9050740000
H	-3.0673950000	2.3307890000	-1.1880490000
H	-2.4098600000	3.6641410000	-0.1955330000
O	-3.5888750000	-1.8149900000	1.1094380000
H	-4.4290430000	-2.2847410000	1.1445780000
C	2.4548680000	-1.5154820000	-0.3617290000
O	3.4096900000	-2.0517990000	0.1881140000
C	2.3817040000	-1.2958700000	-1.8414010000
H	2.3897840000	-0.2255860000	-2.0660050000
H	3.2472640000	-1.7672030000	-2.3043710000
H	1.4625380000	-1.7207010000	-2.2509700000
O	3.3174320000	0.7675260000	2.1603840000
H	3.4562350000	-0.1390470000	1.8641100000
H	2.5021410000	1.0868870000	1.6552300000
O	2.4854920000	2.2582820000	-1.3300480000
H	3.2940900000	1.7370030000	-1.2878900000

H	1.9641240000	1.9938970000	-0.5066180000

alpha-D-Glc-IN-n2w
-1 1

C	-2.0449040000	-0.5066150000	-0.3428360000
C	-0.6340720000	-1.1209540000	-0.4838100000
C	0.3046140000	0.0616320000	-0.6042400000
C	0.1792870000	0.9133630000	0.6365070000
C	-1.2070870000	1.5224640000	0.7172840000
H	-0.3908130000	-1.7021940000	0.4152250000
H	-2.2828630000	0.0154370000	-1.2798920000
H	0.2870810000	0.2822990000	1.5300050000
H	-1.3842160000	2.0544010000	1.6584280000
H	0.0339200000	0.6377080000	-1.5002980000
O	-2.1125320000	0.4260700000	0.7493310000
O	-1.4212500000	2.3731910000	-0.3707520000
O	1.2831440000	1.7799240000	0.5172990000
O	1.6927220000	-0.2062940000	-0.6220190000
O	-0.6318140000	-1.9625880000	-1.6241480000
H	0.2108350000	-2.4529500000	-1.6050810000
C	-3.1331710000	-1.5318360000	-0.1139720000
H	-4.0900970000	-1.0068300000	-0.0141930000
H	-3.1876210000	-2.1870740000	-0.9900080000
C	-2.6812670000	3.0360360000	-0.3347820000
H	-2.6734890000	3.7690620000	-1.1420820000
H	-3.5031700000	2.3302350000	-0.4927650000
H	-2.8203740000	3.5500070000	0.6239620000
O	-2.8515090000	-2.2838280000	1.0617360000
H	-3.5869970000	-2.8866940000	1.2141550000
C	2.4051860000	0.9580310000	-0.0164180000
O	3.2259700000	0.5474660000	0.9175450000
C	3.0303130000	1.7693000000	-1.1414460000
H	2.2897420000	2.0266120000	-1.9035810000
H	3.4566240000	2.6905610000	-0.7341400000
H	3.8327960000	1.1876390000	-1.6028460000
O	2.0162400000	-2.9067490000	-1.1006000000
H	1.9614760000	-3.3884430000	-0.2671190000
H	2.1273370000	-1.9712770000	-0.8405680000
O	2.3224810000	-1.6196560000	2.1816290000
H	2.6355640000	-0.8018010000	1.7028870000
H	1.5832490000	-1.9355200000	1.6500340000

alpha-D-Glc-TSa-n2w
-1 1

C	-2.0540870000	-0.4727160000	-0.2682880000
C	-0.6764660000	-1.1629720000	-0.2673150000
C	0.3743100000	-0.0761700000	-0.4698680000
C	0.1954450000	0.9845560000	0.6032780000
C	-1.1770930000	1.6253500000	0.5539810000
H	-0.5088470000	-1.6593060000	0.6975940000
H	-2.2180640000	-0.0424440000	-1.2658290000
H	0.2935100000	0.5231190000	1.5949790000
H	-1.3616540000	2.2944210000	1.4022460000
H	0.1942970000	0.3762360000	-1.4595550000
O	-2.1158950000	0.5740420000	0.7128840000
O	-1.3345880000	2.3165790000	-0.6519360000
O	1.2752970000	1.8736330000	0.3770040000
O	1.7161740000	-0.4437810000	-0.3420270000
O	-0.6935300000	-2.1202680000	-1.3162130000
H	0.2137950000	-2.4547920000	-1.4492060000

C	-3.2117200000	-1.4071650000	0.0078250000
H	-4.1389280000	-0.8221420000	0.0108120000
H	-3.2699080000	-2.1420600000	-0.8022160000
C	-2.5895540000	2.9798270000	-0.7680380000
H	-2.5386600000	3.5911070000	-1.6696680000
H	-3.4093000000	2.2603390000	-0.8636480000
H	-2.7690640000	3.6233710000	0.1017920000
O	-3.0272070000	-2.0547410000	1.2627800000
H	-3.7922860000	-2.6174250000	1.4230060000
C	2.4974980000	1.1541760000	0.3263560000
O	3.0383570000	0.8492700000	1.4098550000
C	3.3146920000	1.5704570000	-0.8695740000
H	2.7119390000	1.6022710000	-1.7782360000
H	3.7198270000	2.5702560000	-0.6777000000
H	4.1482720000	0.8783380000	-1.0017710000
O	2.0627550000	-2.6841800000	-1.7400540000
H	2.3532000000	-3.3415090000	-1.0978180000
H	2.0961700000	-1.8204920000	-1.2556330000
O	2.0787510000	-2.1672210000	1.7277370000
H	2.3048360000	-1.6184590000	2.4866100000
H	1.9318190000	-1.5195530000	0.9938560000

alpha-D-Glc-TSb-n2w
-1 1

C	-2.3613690000	0.0816310000	-0.2343040000
C	-1.5626620000	-1.2473170000	-0.2490540000
C	-0.1236130000	-0.8723190000	-0.4985310000
C	0.3598250000	0.0970650000	0.5700580000
C	-0.4536340000	1.3825880000	0.4941530000
H	-1.6548490000	-1.7582030000	0.7156900000
H	-2.3210120000	0.5025490000	-1.2482670000
H	0.1273670000	-0.3327910000	1.5582270000
H	-0.2205460000	2.0688920000	1.3161310000
H	-0.0451300000	-0.4182220000	-1.4948400000
O	-1.8161490000	1.0277010000	0.6960090000
O	-0.2397480000	2.0073730000	-0.7372560000
O	1.7339740000	0.1727100000	0.3823800000
O	0.7935140000	-1.9472820000	-0.3857740000
O	-2.0201360000	-2.0884090000	-1.2983240000
H	-2.8015140000	-2.5659680000	-0.9971540000
C	-3.8224260000	-0.0949510000	0.1168780000
H	-4.3061780000	0.8883250000	0.1065230000
H	-4.2961990000	-0.7185100000	-0.6506920000
C	-0.8554530000	3.2857840000	-0.8473000000
H	-0.5156810000	3.7195270000	-1.7884630000
H	-1.9470460000	3.1993710000	-0.8612370000
H	-0.5526370000	3.9331780000	-0.0153920000
O	-3.9429700000	-0.6997510000	1.4003160000
H	-4.8798730000	-0.7623990000	1.6151650000
C	2.1315110000	-1.4826370000	-0.3318150000
O	2.8691670000	-2.1178000000	0.4591190000
C	2.6819790000	-1.0377640000	-1.6705670000
H	1.9899870000	-0.4087730000	-2.2319820000
H	3.6135800000	-0.4904670000	-1.5137910000
H	2.8999560000	-1.9364970000	-2.2572900000
O	2.9707270000	0.2777940000	2.7506420000
H	3.2467690000	-0.6272550000	2.9311430000
H	2.5214030000	0.2294210000	1.8680420000
O	3.0131080000	2.2909850000	-0.7241640000
H	3.7859130000	1.8872150000	-1.1331520000

H	2.4988570000	1.5315770000	-0.3593460000

Naked anion with three explicit water molecules

D-Glucopyranoside

alpha-D-Glc-2Ac
-1 1

C	-2.0131687736	-0.9955449181	0.1229041835
C	-1.5646277980	0.4644844614	0.0016288445
C	-0.1313526285	0.6346874332	0.5145999200
C	0.7473665322	-0.3744204177	-0.2262076707
C	0.2107267799	-1.8031953633	-0.1550046568
H	-1.5977137832	0.7676949976	-1.0535806130
H	-1.9906016666	-1.2889395271	1.1820348644
H	0.8114497974	-0.0890531587	-1.2814351229
H	0.7668317205	-2.4751018056	-0.8203372420
H	-0.1369116447	0.3635789082	1.5876930137
O	-1.1192655525	-1.8285031241	-0.6273959174
O	0.3097193746	-2.2490807462	1.1702367956
O	2.0542551984	-0.3082670767	0.3611864042
O	0.3451631119	1.9321613056	0.3389485590
O	-2.4027231474	1.3090927713	0.7804144055
H	-3.2036375697	1.5078413779	0.2761159951
C	-3.4106403365	-1.2528242807	-0.3938178597
H	-3.6170655623	-2.3275472632	-0.3297076585
H	-4.1274306608	-0.7226636538	0.2439513024
C	-0.1162685811	-3.5994958070	1.3313802841
H	0.1565911950	-3.8992424391	2.3432524188
H	-1.2003149421	-3.6862549762	1.2046494190
H	0.3877405621	-4.2480625381	0.6052558442
O	-3.5036482034	-0.8016463136	-1.7409474149
H	-4.3871387467	-1.0113446722	-2.0699262940
C	3.1121920028	-0.5867807676	-0.4164039083
O	3.0045510223	-0.9340524174	-1.5788937099
C	4.4024711096	-0.4026322893	0.3169480878
H	4.5219198707	0.6576456255	0.5573933574
H	4.3803148108	-0.9621481621	1.2546041846
H	5.2305617413	-0.7368255144	-0.3056177342
O	-0.3081934288	3.2525921588	2.4447358484
H	-1.2436617338	3.0564538342	2.5772829402
O	-1.1307674495	3.5258144909	-1.1089912582
H	-2.0382610845	3.3981815710	-0.8043994273
O	2.3543924524	2.3202867533	-1.3055365656
H	2.3304175931	1.6152880975	-1.9655750664
H	-0.0479599510	2.7368530593	1.6094375895
H	-0.5853031457	2.8823212609	-0.5591584751
H	1.5909475155	2.1288121257	-0.6821266667

alpha-D-Glc-2Ac-H
0 1

C	2.3905967788	-0.1237801235	-0.2041728028
C	1.3900822502	1.0142617348	0.0351316570
C	-0.0155002659	0.5631288829	-0.3507654229
C	-0.3434352679	-0.7351417603	0.3823705893
C	0.7276570421	-1.7943761257	0.1332183916

H	1.3904971709	1.2867433162	1.0990118440
H	2.4138693234	-0.3504307034	-1.2791460169
H	-0.3982805759	-0.5566714330	1.4597713140
H	0.5590671677	-2.6794238054	0.7577311448
H	-0.0557871836	0.3924936033	-1.4335410532
O	1.9833451101	-1.2853777104	0.5280206917
O	0.6984391720	-2.1383376456	-1.2225990562
O	-1.5913131223	-1.2568753568	-0.0870141313
O	-0.9779289079	1.5668946533	-0.0760354389
O	1.7129899227	2.1387253641	-0.7654137445
H	2.4027115808	2.6522677695	-0.3233612268
C	3.8001496594	0.2126637650	0.2260254171
H	4.4346564590	-0.6658181932	0.0620147448
H	4.1746164901	1.0333471404	-0.3971369011
C	1.6113013001	-3.1839262608	-1.5504688834
H	1.3975134361	-3.4764436801	-2.5783650046
H	2.6468391233	-2.8364152230	-1.4768317081
H	1.4639293916	-4.0411538321	-0.8836925052
O	3.7956836268	0.5820114732	1.6002861847
H	4.7077049802	0.7461778970	1.8734778243
C	-2.6861450561	-1.1281767677	0.6794355271
O	-2.6917891272	-0.5432026034	1.7482712250
C	-3.8694460945	-1.8299142459	0.0934172782
H	-4.7824572241	-1.3280849756	0.4123029194
H	-3.8062356391	-1.8713495721	-0.9939549718
H	-3.8743038335	-2.8532509022	0.4825932933
O	-1.2115845979	1.7356758255	2.6357580430
H	-1.7718078919	0.9412440354	2.6025972432
H	-1.0128847875	1.7454794769	0.8941014498
H	-1.7879779684	2.4474391028	2.9449383215
O	-0.7995987524	3.5483203922	-2.0835723693
H	0.1401428842	3.6437428212	-2.2841789572
O	-3.3767855466	1.0793465194	-1.4511877840
H	-3.2075880194	0.2579265264	-1.9308087174
H	-0.8281884141	2.9145016401	-1.3428215371
H	-2.5544885932	1.2524639807	-0.9538638713

alpha-D-Glc-3Ac-a
-1 1

C	2.3063124043	-0.3967502065	0.1630880539
C	1.1784764444	-1.3668323153	-0.1894840952
C	-0.1584083461	-0.7097851214	0.1203028897
C	-0.3039591585	0.6319375407	-0.6012878566
C	0.9157981369	1.4970568632	-0.2554237082
H	1.2240322725	-1.5971985931	-1.2622268042
H	2.2783863512	-0.1965098696	1.2436274066
H	-0.2247625660	0.4264216913	-1.6854186709
H	0.9328114893	2.4072845201	-0.8685580588
H	-0.2738712281	-0.5833737483	1.2012525996
O	2.1235603970	0.8209985291	-0.5623799778
O	0.8486935396	1.8322995387	1.1034936256
O	-1.5012596658	1.2703627644	-0.3016468237
O	-1.1758575608	-1.6130124180	-0.3578388719
O	1.3657634732	-2.5464657601	0.5763942263
H	0.6825664341	-3.1832760738	0.3218563117
C	3.6833938737	-0.9254806537	-0.1688378410
H	4.4188950904	-0.1483585695	0.0697909626

H	3.8864435362	-1.8026987390	0.4547988542
C	1.8833789341	2.7143634377	1.5229222708
H	1.6328611115	3.0382727866	2.5339752157
H	2.8538894930	2.2067600074	1.5335204004
H	1.9362952629	3.5863709968	0.8602605537
O	3.7383464343	-1.2614671076	-1.5506710403
H	4.6390237650	-1.5417768695	-1.7589413893
C	-2.2740932982	-1.8200109619	0.3843223789
O	-2.4742542017	-1.2740776641	1.4539579785
C	-3.1842765550	-2.8366168404	-0.2328441612
H	-3.1045019801	-2.8318186445	-1.3202199245
H	-2.8834439422	-3.8229884644	0.1349927284
H	-4.2097036609	-2.6424859294	0.0806795951
O	-1.7824745057	3.1376368926	-2.0816658169
H	-1.6802190890	2.4273571287	-1.3710300337
H	-2.4554099823	2.7965525095	-2.6836564551
O	-1.8576087288	1.8250553601	2.2286840304
H	-1.7198047902	1.5816702411	1.2615520617
H	-0.9717322561	2.0833754159	2.5145385601
O	-3.4382492082	0.1616816937	-1.6281681173
H	-3.0438567570	-0.5655893048	-2.1258025461
H	-2.6783409929	0.5633156770	-1.0968315415

alpha-D-Glc-3Ac-b
-1 1

C	1.1910632688	1.0132751420	-0.1809599023
C	0.4193950850	-0.2444999721	-0.6136792711
C	-1.0629437785	-0.0366265916	-0.3012200428
C	-1.5853344305	1.2589413346	-0.9098627189
C	-0.7184039881	2.4265724854	-0.4523824895
H	0.5030553726	-0.3132809995	-1.7153413201
H	1.1359780845	1.1097615733	0.9142508447
H	-1.4945631457	1.1993993577	-2.0013670501
H	-0.9950746184	3.3488327081	-0.9780094245
H	-1.2305908201	-0.0468747243	0.7793597297
O	0.6285764346	2.1855739698	-0.7933154976
O	-0.8930189495	2.5877295806	0.9297069012
O	-2.9281105486	1.5218118180	-0.5409739033
O	-1.7884372469	-1.1276041804	-0.9067191109
C	2.6600083970	0.9819556766	-0.5736350864
H	3.0982003738	1.9563522494	-0.3366030505
H	3.1739430734	0.2241792857	0.0264347520
C	-0.1861811125	3.7101581045	1.4498357722
H	-0.4967958363	3.8264430388	2.4883157198
H	0.8956091087	3.5461000459	1.4090671452
H	-0.4399201644	4.6150501591	0.8853373718
O	2.8687324971	0.7401886621	-1.9580516971
H	2.8632753661	-0.2320841854	-2.0759733243
H	-3.5114525530	0.9759776426	-1.0866108137
C	-2.7379946750	-1.7714667469	-0.2166334831
O	-3.0280760460	-1.5065005384	0.9384142745
C	-3.3728957734	-2.8534010116	-1.0321428188
H	-3.7423140422	-2.4380285668	-1.9725462177
H	-2.6173588439	-3.6074485162	-1.2692129412
H	-4.1879137521	-3.3069430757	-0.4709860196
O	0.9230936996	-1.3948054319	-0.0082028468
O	2.7724830665	-2.0197156455	-1.6221930977

H	2.4068794500	-2.5558953263	-2.3368628493
H	2.0048851824	-1.8573989292	-0.9562802920
H	2.0440340622	-1.3486455746	1.2283007512
O	2.6748944765	-1.4501259078	1.9929782114
H	3.5566583737	-1.4360763102	1.6004122989
H	0.0473708537	-1.9596832674	1.3075917742
O	-0.4018846100	-2.3106318798	2.1243169350
H	-1.3276936509	-2.0456310424	2.0316610775

alpha-D-Glc-3Ac-H
0 1

C	2.2284325628	-0.7289231895	0.2775053808
C	0.9836985423	-1.5777960061	-0.0066516139
C	-0.2497842127	-0.6941272840	0.0808120402
C	-0.1202000025	0.5263724634	-0.8261561709
C	1.2069104635	1.2462549183	-0.5725013060
H	1.0519182519	-1.9991672856	-1.0170872560
H	2.1599378155	-0.3261833278	1.2976391316
H	-0.1176249751	0.1894037716	-1.8679021940
H	1.3911852512	2.0015713789	-1.3466589170
H	-0.4085797001	-0.3921228880	1.1209445388
O	2.2885375950	0.3439600330	-0.6670788196
O	1.1301734335	1.8507715253	0.6869376089
O	-1.3709017113	-1.4748604354	-0.3673274561
O	0.8340582757	-2.6067867509	0.9561792373
H	1.3951049016	-3.3540939898	0.7072497324
C	3.5213880026	-1.5051324111	0.1737855917
H	4.3568840508	-0.8167467635	0.3452013423
H	3.5351261185	-2.2728102756	0.9563946241
C	2.2757549168	2.6411539578	0.9957094079
H	2.0386549511	3.2026077279	1.8995945217
H	3.1508774910	2.0084280268	1.1754061647
H	2.4925586331	3.3356554476	0.1744444709
O	3.6098437931	-2.0955544650	-1.1174364729
H	4.4538912084	-2.5611344077	-1.1818635874
C	-2.4713795557	-1.5605770815	0.3912574477
O	-2.6200049239	-0.9483128450	1.4359637209
C	-3.4648825669	-2.5314885195	-0.1627370751
H	-4.4707645740	-2.2243365127	0.1222327129
H	-3.3747954070	-2.6176149032	-1.2456488482
H	-3.2557917589	-3.5091431399	0.2840367712
O	-1.2138101935	1.4138109818	-0.6816237255
H	-1.3726072428	1.5969255153	0.2782081715
H	-0.7069645007	3.2672924548	-0.9420841211
H	-2.7688707076	0.7125979218	-1.3439672196
O	-1.8060985435	1.7272627780	1.9541299598
O	-0.4016018944	4.1909021270	-0.9219730271
O	-3.6196686392	0.3606798651	-1.6709060014
H	-2.1912717215	0.8341748199	1.9962941728
H	-2.5454576898	2.3371383685	2.0804324957
H	0.2615619795	4.2079427059	-0.2191452313
H	-3.3847497168	-0.4217273070	-2.1862782027

alpha-D-Glc-4Ac-a
-1 1

C	0.6278666384	-1.6057881836	0.1409627352
C	-0.3063260139	-0.4155827286	-0.0831236550

C	0.3673815597	0.9102209442	0.2761639151
C	1.6795125048	0.9980748665	-0.5157076462
C	2.5369220946	-0.2484743277	-0.3205474426
H	-0.6404900715	-0.3828228893	-1.1262171903
H	0.8983829455	-1.6529073273	1.2055108853
H	1.4323944866	1.0513393567	-1.5863061536
H	3.3944750559	-0.2490588984	-1.0047609580
H	0.6239320307	0.8691325307	1.3509585340
O	1.8003591016	-1.4096472763	-0.6529849064
O	2.9836879388	-0.2795767277	1.0080716167
O	2.4523041226	2.1245433548	-0.1392019668
O	-0.4762742023	1.9912453372	0.0137706284
O	-1.4551448955	-0.5322066886	0.7799627020
C	0.0400879830	-2.9501811399	-0.2523831764
H	0.8078731880	-3.7149793915	-0.1070576943
H	-0.8003857512	-3.1850191685	0.4122268980
C	3.8608584110	-1.3693712423	1.2759752998
H	4.2733085250	-1.2069772564	2.2720166823
H	3.3218450423	-2.3223354056	1.2556549004
H	4.6739492240	-1.3958070255	0.5407181457
O	-0.3557428951	-3.0096480308	-1.6139547469
H	2.0144377913	2.9124452779	-0.5162995050
C	-2.5842411062	-1.0594552324	0.2974970598
O	-2.6687443939	-1.5454718950	-0.8205781944
C	-3.7211703005	-0.9457874285	1.2605423296
H	-3.3739468799	-1.0741347961	2.2867236647
H	-4.1419820972	0.0607901214	1.1609667017
H	-4.4880862537	-1.6784878897	1.0139324763
H	-1.1884305820	-2.5153455960	-1.6936098118
O	-1.9773992959	2.2457197327	2.1128215618
H	-1.4010142423	2.1541244293	1.2882096117
H	-2.2429093567	1.3429557952	2.3287361865
O	0.6121966121	3.8982122944	-1.2732418010
H	0.4926868259	4.7184249847	-0.7782257650
H	0.1146279042	3.1731246771	-0.7512304142
O	-2.6799262804	1.5886909280	-1.3644024812
H	-2.6528697689	0.7033477121	-1.7504346623
H	-1.8216145987	1.6880942028	-0.8555243637

alpha-D-Glc-4Ac-b
-1 1

C	-0.2720857223	0.5943890003	-0.3796426170
C	-0.4392234828	-0.9214838249	-0.2467379071
C	-1.7966128901	-1.2603537609	0.3415764753
C	-2.8919698318	-0.6115601235	-0.5054984163
C	-2.6227692708	0.8793388288	-0.6978312660
H	-0.3303066492	-1.3983310286	-1.2260820609
H	-0.3449011727	1.0544610684	0.6160243666
H	-2.8952473723	-1.0840821166	-1.4927364217
H	-3.3050558877	1.3139385828	-1.4383453883
H	-1.8513225204	-0.8720011758	1.3689675066
O	-1.3254964791	1.0839916507	-1.2168800151
O	-2.8012435926	1.4979673976	0.5474098823
O	-4.1720851482	-0.8358054491	0.0554332728
O	-1.9370442830	-2.6718350490	0.3483558065
O	0.5536937192	-1.4349121263	0.6527070675
C	1.0413336741	1.0304925116	-1.0310850744

H	1.1673124631	0.4640045616	-1.9710591822
H	0.9057484519	2.0898168608	-1.3121893344
C	-2.6781180514	2.9169024542	0.4934798273
H	-2.9666866415	3.2968081609	1.4734965177
H	-1.6462778544	3.2113882988	0.2765322255
H	-3.3453046976	3.3280899287	-0.2729810430
O	2.1413688128	0.8664213702	-0.1840094095
H	-4.2356520898	-0.3143720714	0.8707459958
H	-2.7794592619	-2.8882023688	0.7733868156
C	1.6729938031	-1.9636337638	0.1349622041
O	1.8306907979	-2.1579517154	-1.0574672472
C	2.6878257052	-2.2732432331	1.1882106710
H	3.2806206294	-1.3666810261	1.3508049879
H	2.2084374069	-2.5456753264	2.1289643451
H	3.3461581775	-3.0685555649	0.8398028684
O	1.8086328346	0.8030420471	2.4076770480
H	1.8939410208	0.8083688899	1.4054467552
H	2.1446600461	1.6618748251	2.6930142189
O	3.1646692195	3.1798056954	0.1952854686
H	2.7792216424	2.2528773284	0.0231350113
H	3.7166396562	3.3808619815	-0.5695709614
O	4.0762858446	-0.2479587752	-1.4346729316
H	3.7961205771	-1.1663866920	-1.5443789628
H	3.3047304176	0.1876317490	-0.9324950990

alpha-D-Glc-4Ac-H
0 1

C	-0.4585612807	0.7465439835	-0.6480058743
C	-0.1766985697	-0.7342649364	-0.4025746275
C	-1.2921185456	-1.3680793557	0.4203146303
C	-2.6213645774	-1.1298691641	-0.2868585625
C	-2.8228696841	0.3578369509	-0.5554385215
H	-0.0767361951	-1.2585527875	-1.3572674378
H	-0.5025998879	1.2836771868	0.3088326084
H	-2.5927902956	-1.6336772777	-1.2634137218
H	-3.7038576883	0.5285800831	-1.1857397545
H	-1.3182645493	-0.9076414464	1.4162468812
O	-1.7285823844	0.8590479573	-1.3075903914
O	-2.9568803474	1.0197036382	0.6668700765
O	-3.7152708784	-1.5964443529	0.4778060555
O	-1.1210276384	-2.7716209229	0.5350924909
O	1.0532318202	-0.8205970428	0.3296418543
C	0.5915167969	1.4197289408	-1.5154668815
H	0.2041215190	2.3945914854	-1.8346582899
H	1.4890666095	1.5852723298	-0.9137542573
C	-3.2540721678	2.4070056752	0.5201799034
H	-3.4702288980	2.7892823840	1.5176827207
H	-2.4009951663	2.9477153312	0.0980166367
H	-4.1294580858	2.5423831343	-0.1254071977
H	-3.6398242006	-2.5586890949	0.5511536239
C	1.9731542137	-1.7340352483	-0.0392451166
O	1.8247155050	-2.4833400890	-0.9852429230
C	3.1562592293	-1.7189531018	0.8751802768
H	3.4483502440	-0.6933751085	1.1055252770
H	2.8717013089	-2.2138368942	1.8092250241
H	3.9807956126	-2.2615450854	0.4148972782
H	-0.4629224284	-2.9534750347	1.2203587780

O	1.0015213425	0.6471246347	-2.6378821547
H	0.2291083861	0.3828400889	-3.1805142646
H	2.2886877688	1.8085303706	-3.4988881063
H	2.6930897492	-0.1156479257	-2.2433118083
O	-1.3581493618	-0.1968846118	-3.8949799043
O	3.1547651791	2.2182915532	-3.6698762081
O	3.6329153705	-0.1075052553	-1.9837373819
H	-1.7760080036	0.0529317894	-3.0514078341
H	-1.3443172197	-1.1636792104	-3.8944058403
H	3.7670051027	1.6081579966	-3.2311687088
H	3.9154209363	-1.0311725575	-2.0096396571

alpha-D-Glc-6Ac
-1 1

C	0.2981203018	-0.2596473636	-0.0544466429
C	-1.1501320232	-0.4286017614	-0.5434413821
C	-1.8805832150	0.8959449911	-0.3144749317
C	-1.1183528842	2.0665487068	-0.9308727128
C	0.3340506690	2.0852476368	-0.4611988584
H	-1.1118874987	-0.5998036778	-1.6377897887
H	0.2921008454	-0.0787846529	1.0309014205
H	-1.1171988415	1.9523037497	-2.0195670381
H	0.9267788230	2.8184946478	-1.0212962725
H	-1.9734329398	1.0558836715	0.7716756299
O	0.9426157716	0.8379026846	-0.7115575182
O	0.3319738226	2.4092504544	0.9043950731
O	-1.7595162734	3.3028043061	-0.6584636869
O	-3.1765937556	0.7987235584	-0.8940489184
O	-1.7793508036	-1.4911832187	0.1042059665
C	1.1315646951	-1.4878016159	-0.3409998375
H	0.6615316559	-2.3701386716	0.0946135383
H	1.2728100753	-1.6296923509	-1.4150369280
C	1.6411755590	2.5136606016	1.4566605937
H	1.5272643632	2.8978851431	2.4704610893
H	2.1318468588	1.5347265127	1.4891658616
H	2.2509665323	3.2074011925	0.8657048348
O	2.4077133875	-1.3598087164	0.3130997879
H	-1.6727438985	3.4795887639	0.2911631794
H	-3.6466652280	1.6260735530	-0.7154642476
C	3.4630861629	-0.9352211902	-0.3996676898
O	3.4227049009	-0.7276059821	-1.5987992523
C	4.6709934503	-0.7507172060	0.4647798109
H	5.5481485899	-0.5820867738	-0.1577615526
H	4.5072692570	0.1167570998	1.1118435735
H	4.8154239983	-1.6249963246	1.1025422859
O	-3.9704909835	-0.9125441693	1.4207201855
H	-4.3502874617	-0.1702065197	0.9323448820
H	-3.1377488768	-1.1267997398	0.9021874034
O	-0.8431100317	-2.1953171719	2.4490844207
H	-1.1674008301	-1.9019666843	1.5437773670
H	0.1120505621	-2.0542712988	2.4330733358
O	-1.4852849870	-3.5862189137	-1.3320712625
H	-0.5471892369	-3.6398119479	-1.5519837254
H	-1.5862265125	-2.7569033229	-0.7484529931

alpha-D-Glc-6Ac-H
0 1

C	-0.2090731418	-0.7485642157	0.6139021152
C	0.1774955895	0.4435381433	-0.2665134104
C	1.6715882725	0.3984236970	-0.5776489561
C	2.0288260503	-0.9659172842	-1.1567867016
C	1.5650317272	-2.0879045825	-0.2368213459
H	-0.3777968640	0.3970583819	-1.2148442964
H	0.3019748517	-0.6497293032	1.5823999826
H	1.4930556518	-1.0886800131	-2.1080093016
H	1.7006701134	-3.0687175514	-0.7079339048
H	2.2382268035	0.5656253714	0.3495679452
O	0.1719972800	-1.9705286296	-0.0168011163
O	2.2922880071	-2.0146992167	0.9564577168
O	3.4232667407	-1.0945168192	-1.3586879440
O	2.0416532524	1.3579866075	-1.5558327169
C	-1.6900005598	-0.8261383117	0.8990508403
H	-1.9092057432	-1.6929179845	1.5284792171
H	-2.0278962683	0.0830166257	1.4037724616
C	1.9879628434	-3.0717820803	1.8630380046
H	2.7104127440	-3.0056311805	2.6765756520
H	0.9746479465	-2.9654292832	2.2635462850
H	2.0826384069	-4.0429007955	1.3634550305
O	-2.3785611796	-0.9648095303	-0.3544234285
H	3.6956084529	-0.3936918991	-1.9690351560
H	2.0893878650	2.2363269930	-1.1372581354
C	-3.7161327085	-0.9248238715	-0.3069243731
O	-4.3286566721	-0.8014317671	0.7390797230
C	-4.3337874501	-1.0382104560	-1.6649873144
H	-5.4111884248	-1.1615269603	-1.5687286339
H	-4.1178865776	-0.1269501149	-2.2309865069
H	-3.8988966006	-1.8819469279	-2.2044678909
O	-0.1935676262	1.6195357034	0.4481844176
H	0.3647180222	2.3851397933	0.1942251873
H	-1.9201687492	1.9912570638	-0.1426081783
H	-0.4000291735	1.5614819477	2.3122002142
O	1.5724584024	3.6907477023	-0.0863251894
O	-2.7894534851	2.1395112296	-0.5606890918
O	-0.5872623059	1.4866584941	3.2659219496
H	2.0788574055	3.9714661686	0.6882779400
H	1.2582799730	4.5029475549	-0.5064714994
H	-2.7649254847	1.6071383201	-1.3665682731
H	-0.1112731370	2.2189691708	3.6770466625

alpha-D-Glc-IN1
-1 1

C	-2.6075981116	0.0165657874	-0.1339262493
C	-1.7871872265	-1.1731449988	-0.6776518917
C	-0.4145926161	-0.6100420095	-0.9515297004
C	0.1642876779	-0.0589147717	0.3311752089
C	-0.6561910170	1.1257408696	0.8013017473
H	-1.7300123793	-1.9710802508	0.0734037668
H	-2.7000151523	0.7589102473	-0.9399219365
H	0.0972764605	-0.8198206674	1.1219819115
H	-0.3490414905	1.5069397473	1.7817967316
H	-0.5053433477	0.1832194300	-1.7082729007
O	-1.9732600636	0.6236011950	1.0027913369
O	-0.6091851972	2.1415306465	-0.1562097814
O	1.5117041567	0.1619434681	-0.0241441680

O	0.6022080314	-1.5179221094	-1.3312774283
O	-2.4464153881	-1.6328956737	-1.8461155994
H	-1.9758708662	-2.4131931924	-2.1721193664
C	-4.0033861766	-0.3750167710	0.2973919280
H	-4.5084192359	0.5129838019	0.6945231453
H	-4.5568341879	-0.7307410206	-0.5782845957
C	-1.3162681869	3.3149882453	0.2368672170
H	-1.1148708487	4.0737132820	-0.5190917903
H	-2.3929437429	3.1255156270	0.2865959841
H	-0.9619026500	3.6652674851	1.2129981411
O	-3.9163098794	-1.3909525049	1.2895612310
H	-4.8107030278	-1.6044616597	1.5852181836
C	1.8732464600	-0.8637073372	-1.0298715576
O	2.7293036353	-1.7489125792	-0.5323956766
C	2.3772929244	-0.1261178430	-2.2594735838
H	2.6155820381	-0.8507620651	-3.0420982029
H	1.6276704201	0.5754640002	-2.6340441029
H	3.2848591832	0.4261640588	-1.9995929894
O	4.7869372532	-0.3997717028	0.6054830322
H	4.0377644064	-0.8794252403	0.1735444206
H	4.5658557434	-0.3928430982	1.5457291226
O	4.5959256384	-2.6296737894	-2.2283938921
H	5.0161649137	-3.3741643562	-1.7805648025
H	3.8895397314	-2.3132040011	-1.6061111174
O	2.2434523173	-2.2240605341	2.0495879847
H	1.9948404455	-1.3663429663	2.4173098450
H	2.3827641553	-2.0537404682	1.0791809349

alpha-D-Glc-IN2
-1 1

C	1.5546788676	1.3202710029	-0.1742093245
C	0.2875684639	0.5393870300	0.1217548307
C	0.5439976715	-0.9382584582	-0.0711793711
C	1.5482021748	-1.4163107262	0.9513374498
C	2.8028916619	-0.5336933540	0.7662521085
H	0.0095710106	0.7372973156	1.1696534185
H	1.9127188612	1.1167992992	-1.1928798898
H	1.1586840392	-1.2650133042	1.9624940123
H	3.5103509064	-0.6864055965	1.5895171825
H	0.9418356054	-1.1129253990	-1.0833211068
O	2.5077746629	0.8507918552	0.7953095331
O	3.3858375990	-0.9117334270	-0.4513581550
O	1.8812252995	-2.7867190576	0.8405731041
O	-0.7646863429	-1.4656931220	0.0237975935
O	-0.8330419897	0.7238059067	-0.7220154176
C	1.4574334137	2.8164279725	0.0479607721
H	1.0430402985	3.0141203216	1.0443438715
H	2.4632806138	3.2477920691	-0.0106600967
C	4.6374665239	-0.2768849208	-0.6990635450
H	5.0651290570	-0.7556249797	-1.5802518928
H	4.5013287464	0.7916020193	-0.8930047443
H	5.3092162286	-0.4114479711	0.1564201375
O	0.6170868627	3.3470183663	-0.9679487733
H	2.3128023867	-2.9218270998	-0.0171149286
C	-1.6788818561	-0.4601013366	-0.5260151897
C	-2.1503130331	-0.9058482145	-1.8981595180
H	-1.2991582367	-1.0916766558	-2.5579252534

H	-2.7360179320	-1.8226500979	-1.7958091274
H	-2.7835195093	-0.1284078617	-2.3360264587
H	0.5601524738	4.3035657714	-0.8461859933
H	-2.1231632064	-0.2071101263	1.9263975422
O	-1.8187564284	-0.1957533519	2.8755447848
H	-2.5696454528	-0.5192600811	3.3886237984
H	-3.5905715355	1.0696433878	-0.2084574770
O	-4.1729596391	1.8004341229	-0.5429050056
H	-4.7770647336	1.3696741455	-1.1616456127
H	-4.0871306168	-1.0484800926	0.0707754293
O	-4.9753546910	-1.4666675605	-0.0859833853
H	-5.3104865366	-1.0397064109	-0.8842452312
O	-2.6562436885	-0.2253923803	0.3487979288

alpha-D-Glc-IN3
-1 1

C	0.7649018877	-0.8425061244	-0.4797561894
C	0.3914087211	0.6329155757	-0.3990915437
C	1.4455574989	1.3604263336	0.4041740092
C	2.7936689053	1.1261163919	-0.2896153392
C	3.0726906928	-0.3627197159	-0.5335049937
H	0.3639367933	1.0639931504	-1.4108816663
H	0.9038187025	-1.2337670733	0.5357841616
H	2.7641241736	1.6243575923	-1.2638510166
H	3.9376677035	-0.4938176624	-1.1946779172
H	1.4766252702	0.9559913662	1.4270905480
O	1.9917450924	-0.9878950880	-1.1995171410
O	3.3289203234	-0.9418137027	0.7182451118
O	3.8512931474	1.7274529479	0.4349304127
O	1.1386829801	2.7446245321	0.4366441852
O	-0.8819616411	0.7144842929	0.2094081024
C	-0.3811348543	-1.5824815703	-1.1759475616
H	-0.0116292664	-2.1125038211	-2.0563793088
H	-0.8476745487	-2.3030186566	-0.4969741940
C	3.7162586974	-2.3108633722	0.6396378339
H	4.0342019930	-2.6072064663	1.6392289401
H	2.8769500906	-2.9375420529	0.3224316124
H	4.5486120983	-2.4309848040	-0.0634928757
O	-1.3522927488	-0.6495783176	-1.6420526151
H	3.9776103624	1.2202733620	1.2518726118
H	1.8440034583	3.1960469537	0.9225213422
C	-1.9553626057	0.1307498843	-0.5991921435
C	-2.6854651512	1.2619097656	-1.3059881921
H	-3.4474901855	0.8508154246	-1.9734369436
H	-3.1701150119	1.8922907855	-0.5558782544
H	-1.9865269747	1.8679460573	-1.8890191365
H	-1.8132269959	-1.2250313220	1.4933408728
O	-1.3202784374	-1.5751432860	2.2830457771
H	-0.5348585599	-1.0161981382	2.3440013341
H	-4.2577615905	-0.8068855643	-0.3502915480
O	-5.2046996018	-0.9090549747	-0.6464826741
H	-5.4971111668	-0.0115086646	-0.8489732093
H	-3.4411341565	0.3997976486	1.3551281717
O	-3.8895035998	0.9785904984	2.0279292684
H	-4.5246625302	1.5001421593	1.5213371629
O	-2.7217569648	-0.6037783450	0.2185320052

alpha-D-Glc-TS1a
-1 1

C	-2.1510243004	0.5452342133	0.3430549211
C	-0.7327884299	1.1322033393	0.2274352719
C	0.2395372296	-0.0308734412	0.3584272719
C	-0.0897367794	-1.0688660561	-0.7009402066
C	-1.4943660566	-1.6087811421	-0.5334116584
H	-0.6073428035	1.6148743422	-0.7519974684
H	-2.2691962130	0.1287337768	1.3540807426
H	-0.0359405091	-0.6120333096	-1.6992747842
H	-1.7961728903	-2.2759889926	-1.3493052000
H	0.0894682352	-0.4765272536	1.3575057749
O	-2.3666712614	-0.4923664122	-0.6263794482
O	-1.5989829361	-2.2665651633	0.6964484765
O	0.9449997886	-2.0237207277	-0.5484914271
O	-0.5896827842	2.0836177512	1.2707494192
H	0.2646832014	2.5384163070	1.1409407448
C	-3.2439621001	1.5702798890	0.1392297485
H	-4.2138258112	1.0625858564	0.1951237631
H	-3.1899588841	2.3110053465	0.9440410746
C	-2.8817489723	-2.8506001389	0.9076090219
H	-2.8166014501	-3.4299146036	1.8287121174
H	-3.6510107933	-2.0792170080	1.0156949901
H	-3.1420716581	-3.5131211436	0.0739136053
O	-3.0751221593	2.1905614567	-1.1306986378
H	-3.8144939255	2.7955435007	-1.2732116128
C	2.2023542087	-1.3619581152	-0.6172305593
O	2.6348317994	-1.0573599789	-1.7490543015
C	3.1221724860	-1.8637885276	0.4654558397
H	2.6127250981	-1.9207387636	1.4284791067
H	3.4648758896	-2.8646161875	0.1821082549
H	3.9902276628	-1.2059598971	0.5379616145
O	1.6007823106	0.2279415844	0.1472321056
O	2.5101847945	0.5486387865	2.6809155168
H	2.2268316325	0.3909426440	1.7476359368
H	2.1979044103	1.4404949661	2.8815283590
O	2.0216359156	2.9657283141	0.5935759361
H	2.1032561409	2.0141427001	0.3809472107
H	1.9706750899	3.4059089163	-0.2661846034
H	1.6987741230	1.1208228382	-1.4685319663
O	1.7213974892	1.6973215304	-2.2620687779
H	2.0137162123	1.1052258027	-2.9673571725

alpha-D-Glc-TS1b

-1 1

C	2.4836787011	0.1153476254	0.1797289593
C	1.7664961665	-1.2506321175	0.2235069266
C	0.3242371539	-0.9697905187	0.5818927879
C	-0.2857851533	-0.0099384080	-0.4208979788
C	0.4510991085	1.3235474177	-0.3585736992
H	1.8181573223	-1.7351137546	-0.7588510842
H	2.5050795442	0.5182548623	1.2014756231
H	-0.0860380823	-0.3883972093	-1.4352877412
H	0.1200782288	2.0215368714	-1.1351433438
H	0.2875893580	-0.5469432305	1.5948373450
O	1.8070897125	1.0415173597	-0.6819811523

O	0.3220242846	1.9038716511	0.9062703888
O	-1.6604004835	-0.0610570534	-0.1561517167
O	-0.5348962055	-2.0909872352	0.4920993606
O	2.4328266907	-2.0407142906	1.1977045501
H	2.0658847682	-2.9319031846	1.1646831208
C	3.9170259632	0.0314713034	-0.2983946376
H	4.3378874649	1.0433737643	-0.3117785320
H	4.4911143146	-0.5728848725	0.4121919407
C	0.9123975966	3.1975478561	1.0052653857
H	0.6650832573	3.5784486024	1.9969394566
H	2.0001311326	3.1447569453	0.8966492307
H	0.4991566133	3.8673567338	0.2417110995
O	3.9599604086	-0.5448639119	-1.5995637735
H	4.8773811094	-0.5542612536	-1.8925983551
C	-1.9049154061	-1.6694884905	0.4869711521
O	-2.6382141414	-2.3430980591	-0.2911483429
C	-2.4181526057	-1.3715670201	1.8834029435
H	-2.5702017792	-2.3285999917	2.3925363055
H	-1.7282534185	-0.7650261997	2.4728469700
H	-3.3798443794	-0.8581349150	1.8171018869
O	-2.7294576671	2.2935361438	-1.5264040995
H	-2.4249746021	1.5151961856	-1.0284180298
H	-2.7809307638	1.9871993049	-2.4386225138
O	-2.4777021618	1.9180686107	1.7102657601
H	-2.2936342755	1.1531866370	1.1295889599
H	-1.6232902675	2.3682746184	1.7044417078
O	-2.5906243516	-0.6424569252	-2.8096635560
H	-2.8272866667	-1.5738427167	-2.8768117791
H	-2.3505704889	-0.5271021351	-1.8693265257

alpha-D-Glc-TS2a
-1 1

C	-0.6117482990	1.2924531090	0.0917835541
C	0.1974837948	0.1042835857	-0.4050811636
C	-0.5843091107	-1.1776253324	-0.2035492238
C	-1.8720441088	-1.1221832619	-0.9943534664
C	-2.6267118736	0.1444824256	-0.5546467300
H	0.3294017177	0.2440512396	-1.4937194598
H	-0.8574538473	1.1820863244	1.1572505754
H	-1.6407663824	-1.0141425332	-2.0613979016
H	-3.4914232705	0.3214133478	-1.2056272261
H	-0.8039332248	-1.3260057579	0.8636871834
O	-1.8244657556	1.3037951190	-0.6847650647
O	-3.0480577645	-0.0453694603	0.7690013068
O	-2.7222294781	-2.2355881747	-0.7825803325
O	0.3215295075	-2.1718868084	-0.6611445004
C	0.0910454189	2.6231815872	-0.0998231869
H	-0.5985571983	3.4318469060	0.1592540442
H	0.9403973514	2.6608808003	0.5946352000
C	-3.8593925548	1.0209432082	1.2534113312
H	-4.2460165558	0.7074763761	2.2232790316
H	-3.2738229413	1.9381789378	1.3719709126
H	-4.6938680529	1.2076557057	0.5672023213
O	0.5203583870	2.8477931028	-1.4347662056
H	1.3269342967	2.3201070045	-1.5983349111
H	-2.3302258550	-3.0020992415	-1.2240212689
C	1.6348207148	-1.9613225961	-0.1598423101

O	2.5451192932	-2.0928154025	-1.0054171836
C	1.8264133131	-2.4545642717	1.2553289369
H	0.9526523164	-2.2693383399	1.8818129247
H	2.0086859805	-3.5329645941	1.2091661427
H	2.7005714395	-1.9684784725	1.6940607429
O	1.4240842232	-0.1502554818	0.2089461750
O	2.8334445836	1.1845777774	-1.7209362148
H	2.7980298247	0.7343172840	-2.5754836102
H	2.4757498645	0.5405154027	-1.0679606936
H	2.6620875334	0.9984930254	0.9669729735
O	3.3840853533	1.5569773951	1.3154447043
H	3.7852453451	1.9494876927	0.5288213102
H	1.0900484894	0.0111596892	1.9990142270
O	0.9197771610	0.1818541137	2.9506331947
H	0.6725143638	1.1150855692	2.9907388615

alpha-D-Glc-TS2b
-1 1

C	-1.9176197912	-0.8271544849	-0.3843739789
C	-0.4407568145	-0.9621507796	-0.0713544494
C	0.2620173285	0.3746963888	-0.2300587843
C	-0.3343844329	1.3555147015	0.7708772575
C	-1.8524514979	1.4027852161	0.5318692258
H	-0.3250079819	-1.3255210178	0.9600766589
H	-2.0552565591	-0.4349167024	-1.4013343913
H	-0.1724607554	0.9812643954	1.7915149765
H	-2.3551351131	1.9634012663	1.3297037948
H	0.0614721757	0.7566651435	-1.2464877913
O	-2.4314038161	0.1101336683	0.5778474397
O	-2.0778196749	2.0207472680	-0.7065317979
O	0.1667346513	2.6738309714	0.6433562076
O	0.2761121490	-1.7998361434	-0.9639637505
C	-2.7167375776	-2.1026987818	-0.2622751104
H	-3.7757821452	-1.8752215884	-0.4324890448
H	-2.3785934507	-2.7978388406	-1.0398029212
C	-3.4601526037	2.2034360702	-0.9965213088
H	-3.5154702878	2.8100492805	-1.9006479926
H	-3.9572464436	1.2434420532	-1.1696110803
H	-3.9579283219	2.7262839760	-0.1710451681
O	-2.5230375900	-2.6582841418	1.0325611296
H	-3.0517107207	-3.4639851048	1.0989089081
H	1.0905318811	2.6656849613	0.9599773788
C	1.6677008073	-1.7083571877	-0.6805455802
O	2.1069879993	-2.3779358317	0.2798918715
C	2.4546107369	-1.5665737535	-1.9584893175
H	2.0056543871	-0.8285787755	-2.6253700006
H	3.4830249745	-1.2858822260	-1.7234747834
H	2.4644966769	-2.5396530571	-2.4607473996
O	1.6200477807	0.0552899997	-0.0977274447
H	2.6046430534	1.2934385223	0.8694295560
H	1.8767839672	-0.4883155192	1.6731284738
H	2.4192500619	1.0942941514	-1.2504172436
O	2.8247001769	2.0829305621	1.4058033414
O	2.0212831028	-0.6064006532	2.6353356209
O	2.8322950295	1.7696009154	-1.8388190310
H	2.8081062158	1.7672221358	2.3200574406
H	2.0492907100	-1.5654017835	2.7491337112

H 2.9307777126 1.3341217264 -2.6947226221

alpha-D-Glc-TS3a
-1 1
C -0.3131145904 0.5565387368 -0.4076673042
C -0.5031436027 -0.9288906674 -0.1150360104
C -1.8893268192 -1.1636833680 0.4625067122
C -2.9226556887 -0.6080064015 -0.5123161265
C -2.6273534696 0.8515199788 -0.8548755476
H -0.4083194506 -1.5087983140 -1.0426132712
H -0.4197589832 1.1187608966 0.5301183278
H -2.8553299651 -1.1784947903 -1.4485182099
H -3.2725717491 1.1993632511 -1.6705254176
H -1.9748567715 -0.6445970677 1.4269315320
O -1.3053950099 0.9799507431 -1.3430842344
O -2.8400884002 1.6245780157 0.2935748106
O -4.2344297232 -0.6824702817 0.0140329176
O -2.1725326651 -2.5447827059 0.6254611451
O 0.4528114382 -1.3599463173 0.8491122184
C 1.0588096539 0.8236750705 -1.0079942082
H 1.1015023163 0.3582455757 -2.0075830432
H 1.1927959406 1.9075978924 -1.1445832884
C -2.6711981729 3.0207964208 0.0633647023
H -3.0036085543 3.5326910177 0.9664817219
H -1.6207828129 3.2625048182 -0.1280611699
H -3.2806517360 3.3431506000 -0.7890561906
H -4.4575559340 -1.6177221456 0.1241356225
H -1.6452477955 -2.8823539312 1.3630223872
C 1.7879929286 -1.4795840042 0.4521485033
O 2.5859969017 -1.5956933983 1.3992693579
C 2.0335485093 -2.2137059991 -0.8478449725
H 1.6368218732 -3.2289810418 -0.7374641641
H 1.5712027410 -1.7527515984 -1.7201580308
H 3.1111764666 -2.2715303933 -1.0028365310
O 2.0550248154 0.3083598951 -0.1560220211
H 1.7320667609 0.9033582920 1.4891348361
H 3.2068761275 1.6197724799 0.2018775216
H 3.3883805275 0.1148360753 -1.2317339238
O 1.5575430833 1.2983571802 2.3746204381
O 3.8239518990 2.3735186165 0.3280593862
O 4.1325460741 0.0935386681 -1.8832823490
H 0.9333464478 0.6900135772 2.7913840869
H 3.6313704246 2.7067141497 1.2137984021
H 4.6244689645 0.9081944745 -1.7173146150

alpha-D-Glc-TS3b
-1 1
C 0.5160958764 -1.1716757752 -0.3116098258
C -0.1279383930 0.1976061338 -0.5336748279
C 0.8627277813 1.2675402877 -0.0731901136
C 2.1803205800 1.0730035376 -0.8167782684
C 2.7087368823 -0.3520084237 -0.6652006159
H -0.2770511295 0.3313301712 -1.6221909770
H 0.7382174407 -1.3008290013 0.7568915586
H 1.9918910928 1.2311183241 -1.8879841142
H 3.5619489176 -0.5274854470 -1.3315478988
H 1.0285978938 1.1599919845 1.0097266968

O	1.7209238736	-1.2815610150	-1.0703382128
O	3.0910362362	-0.5436852940	0.6689875943
O	3.1760291138	1.9715165218	-0.3609316297
O	0.4267495093	2.5883787569	-0.3628409664
C	-0.4133946999	-2.2886986968	-0.7454532365
H	-0.6503124919	-2.2153295984	-1.8127128244
H	0.0443320602	-3.2621367898	-0.5566818142
C	3.6820274537	-1.8189280307	0.9019983726
H	4.0733683739	-1.8042437873	1.9193229252
H	2.9405267644	-2.6189939036	0.8082217047
H	4.5009822680	-1.9955635382	0.1947718595
O	-1.5918920998	-2.2440399132	0.0585378584
H	2.8054390883	2.8642887676	-0.4223854885
H	-0.2397762247	2.8332394344	0.3065717145
C	-2.5042868777	-1.2031037167	-0.1373449572
O	-3.3515395246	-1.1041619410	0.7731262227
C	-2.9028202464	-0.9411478628	-1.5737000216
H	-3.6538061352	-0.1509366569	-1.5788378742
H	-2.0777379707	-0.6566733621	-2.2261154756
H	-3.3556453312	-1.8596528119	-1.9625989460
O	-1.3541017287	0.2650573461	0.1472617499
H	-1.6155307455	1.6946018405	1.2530261841
H	-2.2929043226	3.0557817484	-0.8851476896
H	-1.1000450305	-0.2503941500	1.8471395244
O	-1.5548397052	2.5866786232	1.6579007236
O	-2.6836283208	2.2483391415	-1.2440075500
O	-0.9632933932	-0.4653597994	2.7974113088
H	-1.1113277921	2.4495769944	2.5059905113
H	-2.2347571807	1.5204668456	-0.7549982314
H	-1.5658108623	-1.2014209441	2.9659640500

beta-D-Glc-2Ac
-1 1

C	2.1350770686	-0.3780594128	-0.3357876292
C	1.3032352039	0.9055798288	-0.2854557576
C	-0.1455012812	0.6384002299	-0.7226425260
C	-0.6880723656	-0.5466964957	0.0809503730
C	0.2379167881	-1.7502680583	0.0023287824
H	1.2883152864	1.2845146817	0.7476211227
H	2.1780992799	-0.7333400326	-1.3776810040
H	-0.8228496083	-0.2729534288	1.1311551680
H	0.3280964287	-2.1125096990	-1.0362824301
H	-0.1059459926	0.3218678068	-1.7827304945
O	1.5178514438	-1.3751705787	0.4794531316
O	-0.2627964673	-2.7529535712	0.8270856705
O	-1.9457444850	-0.9416532597	-0.4876904944
O	-0.9567160498	1.7634753210	-0.5700158028
O	1.9322816018	1.8463168969	-1.1428771401
H	1.5395298354	2.7219346108	-0.9579244823
C	3.5567867409	-0.2007242023	0.1499782129
H	4.0566633825	-1.1760965952	0.1355201222
H	4.0825764093	0.4734066127	-0.5339307739
C	0.3456827629	-4.0257260197	0.6051605560
H	-0.1535402870	-4.7320489405	1.2682760990
H	0.2041289590	-4.3354025129	-0.4362057620
H	1.4134207310	-3.9966412258	0.8403002945
O	3.5380487260	0.3313865272	1.4703430021
H	4.4508146242	0.4169473649	1.7740074166

C	-3.0584260793	-0.8820073567	0.2558218058
O	-3.0832242227	-0.4579455798	1.3990250386
C	-4.2439574125	-1.4392957457	-0.4666614212
H	-4.1871023289	-1.2236719804	-1.5337125319
H	-4.2374656530	-2.5258553544	-0.3311962701
H	-5.1585017211	-1.0328779233	-0.0363296677
O	0.2900869580	3.9742528652	-0.3742347281
H	0.4412739789	4.1249245296	0.5678137892
O	-1.7405625391	2.1281344173	1.9410025731
H	-2.2363605985	1.3127620546	2.1072010989
O	-3.5166411325	1.7700350988	-1.0194964620
H	-3.9086041767	1.7432943209	-0.1371457761
H	-0.2571203111	3.1123578724	-0.4348118353
H	-1.4044523804	2.0000957040	1.0087537604
H	-2.5220160568	1.7141233905	-0.8682534585

beta-D-Glc-2Ac-H
0 1

C	2.1412486332	-0.1378001556	-0.3645218428
C	1.2419164156	1.0326503924	0.0550980704
C	-0.2139146669	0.7138457205	-0.2744416023
C	-0.5993267871	-0.6484031517	0.2964710647
C	0.3976710790	-1.7170989635	-0.1333424809
H	1.3358168470	1.1995141061	1.1354341061
H	2.0694209190	-0.2631240930	-1.4559934018
H	-0.6310631480	-0.6214774927	1.3900124837
H	0.3922257038	-1.8458396915	-1.2291858232
H	-0.3241746624	0.6783297334	-1.3674294461
O	1.6926988827	-1.3257755432	0.2864837946
O	0.0653217402	-2.9060176247	0.5026113507
O	-1.8775179177	-1.0284694329	-0.2252462269
O	-1.0892785533	1.7299068058	0.1781235799
O	1.5970495520	2.2077267853	-0.6601305151
H	2.2889587686	2.6825231579	-0.1793451134
C	3.5974128648	0.0690155831	-0.0143840241
H	4.1587750588	-0.8269889196	-0.3012897961
H	3.9804701265	0.9207358750	-0.5881771718
C	0.7625562709	-4.0399251252	-0.0161945975
H	0.3657173944	-4.9133199546	0.5007919848
H	0.5814635822	-4.1350380003	-1.0923909523
H	1.8364682752	-3.9597842053	0.1732453326
O	3.7093952905	0.3082798169	1.3839186903
H	4.6468781260	0.3800157417	1.6058391033
C	-2.9608639799	-0.9522884013	0.5638500635
O	-2.9325241999	-0.5135612921	1.7001986975
C	-4.1767087329	-1.5166370851	-0.0983275354
H	-5.0685150435	-1.0516015897	0.3201406889
H	-4.1352612609	-1.3791343249	-1.1789054975
H	-4.2018892501	-2.5903108658	0.1152987439
O	-1.2459772973	1.4561635070	2.9076637416
H	-1.9035726033	0.7514866976	2.7775591735
H	-1.1011853978	1.7391834485	1.1628294017
H	-1.7078410711	2.1627005989	3.3783102901
O	-0.5229687024	3.4684693046	-2.1314272185
H	0.2664355745	3.1267024760	-1.6755662779
O	-3.5374109823	1.5482173303	-1.1485574905
H	-3.3991829489	0.8405404582	-1.7914914289

H	-1.2510029443	3.1440669066	-1.5827743397
H	-2.7017066449	1.5939754668	-0.6435031292

beta-D-Glc-3Ac-a
-1 1

C	2.4733329311	-0.3404005227	0.3699806836
C	1.3658482566	-1.3309692604	0.0049631258
C	0.0201863263	-0.6408863010	0.1574874581
C	-0.0584289835	0.6507067941	-0.6610915235
C	1.1337253816	1.5199509069	-0.2394054368
H	1.4945006444	-1.6442497206	-1.0394327701
H	2.3597212262	-0.0547952863	1.4280183493
H	0.0877376446	0.3631178279	-1.7186165181
H	1.0474724227	1.7774296924	0.8266776156
H	-0.1736211256	-0.4344763805	1.2156810479
O	2.3553749332	0.8171226384	-0.4520615556
O	1.2141353721	2.7389343529	-0.9144333645
O	-1.2629326816	1.3146706416	-0.4737490674
O	-0.9749866661	-1.5607304625	-0.3325176454
O	1.4746508860	-2.4473710718	0.8733179290
H	0.7788956259	-3.0795341745	0.6411259769
C	3.8641952029	-0.9073001788	0.1913032252
H	4.5929221722	-0.1209789531	0.4183936048
H	4.0045749910	-1.7312562922	0.8991205675
O	4.0166370918	-1.3607151156	-1.1491016075
H	4.9146791140	-1.7014305079	-1.2527216883
C	-2.1069479716	-1.7446285976	0.3602475993
O	-2.3549836784	-1.1781083653	1.4102848772
C	-2.9959072012	-2.7669115963	-0.2779009954
H	-2.8398128959	-2.8121368018	-1.3559041810
H	-2.7447946744	-3.7417333999	0.1527322735
H	-4.0360173636	-2.5394500362	-0.0452191949
O	-1.6288443229	3.8712354461	-0.9886843269
H	-1.4218785246	2.9073238083	-0.8126190246
H	-2.5424959518	3.9893926034	-0.7010351802
O	-1.7821952103	1.6509403843	2.0557396851
H	-1.5616107248	1.5481852772	1.0773143142
H	-1.9979255063	0.7454512591	2.3192102678
O	-3.0749589298	0.2049210043	-1.9236762181
H	-2.6790933187	-0.6029701745	-2.2737949439
H	-2.3554747031	0.6288241127	-1.3454399607
C	1.0951542467	2.6739875309	-2.3362624503
H	1.3262047890	3.6739002732	-2.7045721560
H	1.8067924203	1.9598322544	-2.7617190655
H	0.0761497956	2.4086332611	-2.6349847660

beta-D-Glc-3Ac-b
-1 1

C	-1.3024640640	1.0168221792	0.3318912989
C	0.0510727423	0.7219108200	-0.3379284197
C	0.4298250275	-0.7213373402	-0.0090161162
C	-0.6796675786	-1.7031340846	-0.3781257851
C	-1.9816365242	-1.2523040019	0.2731385688
H	-0.1099827735	0.7767254663	-1.4309997807
H	-1.1929268640	0.9266483444	1.4253404558
H	-0.8256832927	-1.6975499590	-1.4664981370
H	-1.8990702692	-1.2962382517	1.3734294264

H	0.6689529872	-0.8069984568	1.0569308143
O	-2.2790081583	0.0741826448	-0.1210928805
O	-3.0091060106	-2.0800802285	-0.1693998566
O	-0.3965263477	-3.0136633351	0.0805180897
O	1.6018550936	-1.0499813615	-0.7807548107
C	-1.8308311294	2.4091018278	0.0288886242
H	-2.8335562471	2.4998939717	0.4572581472
H	-1.1832562007	3.1458420680	0.5178475612
C	-4.2121853085	-1.9412474559	0.5856766286
H	-4.9127097323	-2.6806934909	0.1975344033
H	-4.0187036556	-2.1388027059	1.6459223279
H	-4.6370927432	-0.9403184727	0.4682769403
O	-1.9310916544	2.6811123396	-1.3616360146
H	-1.0531702721	3.0039450128	-1.6531052178
H	0.2243637443	-3.4334169779	-0.5305655531
C	2.6092180875	-1.7255623038	-0.2054838683
O	2.5786862185	-2.1244691080	0.9452144801
C	3.7512488908	-1.9177058845	-1.1532214181
H	3.4044038212	-2.4634278937	-2.0344086864
H	4.1147427297	-0.9412129957	-1.4831241909
H	4.5502103761	-2.4689410043	-0.6605670033
O	1.0340902377	1.6249814287	0.0632410276
O	0.7015166267	3.5336593340	-1.5613022148
H	1.1933624204	3.3505592705	-2.3714736749
H	0.9235558179	2.7717697323	-0.9046076762
H	0.8491048434	2.3263384472	1.5154630319
O	0.8180229167	2.7621057389	2.4187057898
H	0.2705521630	3.5487206193	2.3046263959
H	2.4609697591	1.1042289244	0.6469683326
O	3.3609489813	0.8841734677	1.0279031183
H	3.2166483414	0.1194396754	1.5999518419

beta-D-Glc-3Ac-H
0 1

C	2.3464056331	-0.7124087592	0.4606787220
C	1.1529238491	-1.5506516963	-0.0199844879
C	-0.1141098794	-0.7215096766	0.1118587795
C	0.0290044466	0.6171848852	-0.6066955251
C	1.2870740274	1.3286068214	-0.0942837115
H	1.2976780123	-1.8232169194	-1.0722634164
H	2.2013895318	-0.4715212607	1.5254592072
H	0.1275224977	0.4221525497	-1.6802190161
H	1.1778113632	1.5480859290	0.9778384244
H	-0.3352330791	-0.5678835353	1.1737554336
O	2.4146965593	0.4933952429	-0.2975415168
O	1.5279463653	2.5558889152	-0.7074826706
O	-1.1844533855	-1.4620933162	-0.4951730245
O	0.9895561654	-2.7078470797	0.7809503889
H	1.5780303009	-3.4023691013	0.4544756093
C	3.6708850547	-1.4282070413	0.3217375783
H	4.4710006085	-0.7455808844	0.6281672127
H	3.6772567158	-2.2966274045	0.9907325326
O	3.8417085993	-1.8310847018	-1.0322404360
H	4.7165137396	-2.2311401797	-1.1216093223
C	-2.3535778946	-1.5845965617	0.1494542871
O	-2.5918927751	-1.0491778299	1.2187612753
C	-3.3044184663	-2.4890043760	-0.5680546412

H	-4.3257866859	-2.1540194594	-0.3877109737
H	-3.0894049571	-2.5263924529	-1.6359210730
H	-3.1859986915	-3.4934472398	-0.1487147414
O	-1.1125273655	1.4360971167	-0.4364043333
H	-1.3168343087	1.5263266877	0.5276864002
H	-0.9515451146	3.2868782321	-0.7916252672
H	-2.5047551037	0.8205021914	-1.4611474761
O	-1.6721841458	1.4582929619	2.2205316157
O	-0.9683452412	4.2553810222	-0.9022038255
O	-3.2209504113	0.4821393812	-2.0317795919
H	-2.0942346024	0.5840299579	2.1577986979
H	-2.3726621971	2.0696436166	2.4855527801
H	-0.0389094086	4.5131688072	-0.8462360687
H	-2.8403429381	-0.2866160568	-2.4759689205
C	1.5665922059	2.5469366709	-2.1378827579
H	1.9733382474	3.5139654637	-2.4342492778
H	2.2166350560	1.7508607498	-2.5109489827
H	0.5619608321	2.4374361702	-2.5587060767

beta-D-Glc-4Ac-a
-1 1

C	0.7762683493	-1.3082551030	0.2913239742
C	-0.2781666592	-0.2546916004	-0.0556855027
C	0.2235913472	1.1590714057	0.2434354906
C	1.5771890859	1.3708297906	-0.4520278401
C	2.5318270831	0.2345579357	-0.1129879911
H	-0.5608415569	-0.3305411733	-1.1114671545
H	0.9989879584	-1.2502326488	1.3685007115
H	1.4262859092	1.3766473657	-1.5426496908
H	2.7646396132	0.2330452277	0.9663435270
H	0.4149253678	1.2089547049	1.3336910136
O	1.9478898650	-1.0119361806	-0.4617099532
O	3.6977696213	0.3821493550	-0.8587799669
O	2.1911247755	2.5755513896	-0.0276266128
O	-0.7187897836	2.1155764303	-0.1349828035
O	-1.4463976621	-0.4443734232	0.7662038757
C	0.3719775741	-2.7368930340	-0.0311979867
H	1.2189331349	-3.3906672978	0.1925270791
H	-0.4629279985	-3.0327352822	0.6154695745
C	4.7584505805	-0.4694390023	-0.4267230805
H	5.6296056720	-0.2171905836	-1.0314460143
H	4.9811133815	-0.2898079787	0.6311059942
H	4.5033395231	-1.5223538741	-0.5755300695
O	0.0484502870	-2.9262448597	-1.3997180587
H	1.6683459872	3.3200157040	-0.3865889419
C	-2.4839706534	-1.1355858999	0.2830470580
O	-2.4615776796	-1.7028661844	-0.7984809192
C	-3.6584303017	-1.1059180567	1.2070070054
H	-3.3330207627	-1.2324579786	2.2409753954
H	-4.1375831188	-0.1251771178	1.1146966790
H	-4.3679034247	-1.8824782085	0.9257772106
H	-0.8345070374	-2.5475714506	-1.5434499222
O	-2.3048998001	2.4086177416	1.8919835099
H	-1.6947039661	2.2948634004	1.0943414636
H	-2.6506254972	1.5249971790	2.0699864493
O	0.2400869053	4.2610337059	-1.1059159041
H	0.0236987939	4.9919871747	-0.5133135443

H	-0.2095314033	3.4312636212	-0.7114354238
O	-2.8327576899	1.3526519366	-1.5066600382
H	-2.6508441043	0.4963421219	-1.9155455580
H	-1.9984555557	1.5955481375	-1.0081927447

beta-D-Glc-4Ac-b
-1 1

C	0.4632651107	0.3299397401	-0.8146193532
C	-0.0097109713	-0.8937568778	-0.0304221511
C	1.1688679972	-1.7860845259	0.3083852328
C	2.2149541099	-0.9700888723	1.0685715331
C	2.5658934863	0.2943156159	0.2873887782
H	-0.5032217164	-0.5711934103	0.8876096169
H	0.9739082837	0.0050278081	-1.7354133214
H	1.8025677098	-0.6695041008	2.0388263214
H	3.0603686681	0.0373093115	-0.6671758845
H	1.6189904440	-2.1584870122	-0.6257718474
O	1.3878871823	1.0362396531	0.0141550493
O	3.4031073177	1.0814958820	1.0709066164
O	3.3618049734	-1.7536591320	1.3440958201
O	0.7045396603	-2.8741990824	1.0885085455
O	-0.9480632767	-1.6080447565	-0.8444270333
C	-0.6723675732	1.2734062056	-1.1840243956
H	-0.2073701204	2.1567616114	-1.6539898670
H	-1.2644478059	0.7741589740	-1.9692737432
C	4.0317717557	2.1361598628	0.3431058081
H	4.6864411168	2.6547483527	1.0435333110
H	4.6263635534	1.7249711094	-0.4803580324
H	3.2913295857	2.8382435051	-0.0507442573
O	-1.4794608866	1.6348402516	-0.1010703496
H	3.7541303072	-2.0405836460	0.5050676882
H	1.4589291929	-3.4509449990	1.2773562556
C	-2.0844867861	-2.0478317970	-0.2692751041
O	-2.3225114365	-1.9207263860	0.9164119582
C	-2.9741562356	-2.7258881587	-1.2621848045
H	-3.0456380779	-2.1288698082	-2.1731191118
H	-2.5347687114	-3.6937032962	-1.5221657209
H	-3.9594539430	-2.8786322612	-0.8247841207
O	-0.9577197121	1.3635527511	2.4626339928
H	-1.1017097099	1.4050854716	1.4729366651
H	-1.3512595096	0.5274920914	2.7433218895
O	-3.7031207980	0.4537757352	-0.5171733536
H	-2.8296498574	0.9477470863	-0.3273996978
H	-4.2470200791	1.0747012209	-1.0168752555
O	-0.8715753120	4.0708233540	0.4558876879
H	-0.4009042457	4.0226318222	1.2971367934
H	-1.1316723904	3.1133920165	0.2433102214

beta-D-Glc-4Ac-H
0 1

C	-0.5017241714	-0.4264618581	-0.6822739529
C	-0.1575917247	0.8957473313	0.0020497503
C	-1.4114635137	1.7403399224	0.1782635220
C	-2.4624029535	0.9411344202	0.9406793889
C	-2.7049484724	-0.3828025735	0.2289359949
H	0.3044489911	0.7043927393	0.9743502548
H	-0.9304376249	-0.2323359549	-1.6766506207

H	-2.0898888347	0.7227521894	1.9530733853
H	-3.1085627384	-0.2173987767	-0.7841175666
H	-1.8122719185	1.9995630294	-0.8119301436
O	-1.4751766794	-1.0943163735	0.1240458640
O	-3.5772342180	-1.1481262361	0.9893315857
O	-3.6905544671	1.6396697542	0.9975355582
O	-1.1549599688	2.9186057970	0.9230580634
O	0.7627186599	1.5936009590	-0.8455984763
C	0.6993037851	-1.3406995053	-0.8563141339
H	0.3388026826	-2.3363605008	-1.1399923239
H	1.3227775717	-0.9536080591	-1.6662172249
C	-4.0658157683	-2.3031912720	0.3041596166
H	-4.8345471015	-2.7405481743	0.9407220024
H	-4.5030950268	-2.0152544500	-0.6580044851
H	-3.2651434029	-3.0304651009	0.1444919476
H	-3.5257791569	2.4972042096	1.4167986471
C	1.8685323629	2.1389412570	-0.3024003667
O	2.1180121880	2.0917336845	0.8867272599
C	2.7167820623	2.8009504739	-1.3412641737
H	2.8920721095	2.1135332370	-2.1713921025
H	2.1821601380	3.6721642800	-1.7307232513
H	3.6604871103	3.1149515893	-0.8981428110
H	-0.7201570803	3.5649104486	0.3486623636
O	1.5371182023	-1.4173991178	0.2920614405
H	1.0193979093	-1.6554819520	1.0930801166
H	2.7314434087	-2.7765202815	-0.1235529972
H	3.1396424985	-0.5074978347	0.1015357786
O	-0.1058675640	-1.8519124824	2.4815071400
O	3.3720394052	-3.4282296860	-0.4648819449
O	4.0009604305	-0.1329474708	-0.1607762022
H	-0.8333383258	-1.5262837518	1.9221975734
H	0.0703223831	-1.1422142467	3.1142026620
H	2.9777683367	-4.2907301925	-0.2849637721
H	4.0839218666	0.6822425092	0.3510951640

beta-D-Glc-6Ac
-1 1

C	0.1611246759	-0.3822757487	0.1139112567
C	-1.2294912221	-0.2636854426	-0.5262876335
C	-1.8313940646	1.0858302982	-0.1067032633
C	-0.8807739435	2.2466846008	-0.3892468871
C	0.4952988102	1.9511508184	0.2028200025
H	-1.0840570705	-0.2341987302	-1.6235232828
H	0.0596611494	-0.3704801636	1.2119291947
H	-0.7649062272	2.3685278403	-1.4725919664
H	0.4429641451	1.9049883277	1.3061935746
H	-2.0164519104	1.0467243822	0.9814277609
O	0.9769640191	0.7146139465	-0.2903340365
O	1.3812675859	2.9480323257	-0.1968715144
O	-1.3879305095	3.4771855172	0.0987273390
O	-3.0492632985	1.3507146378	-0.7855159167
O	-2.0508866366	-1.3341448977	-0.1639882170
C	0.8760904252	-1.6486055015	-0.3003769719
H	0.2870226587	-2.5230459318	-0.0208541816
H	1.0754584225	-1.6569577078	-1.3746579000
C	2.6535370280	2.8591276807	0.4440334374
H	3.2157350857	3.7451039056	0.1488772654

H	2.5302615876	2.8476940315	1.5331616482
H	3.1920969748	1.9610116612	0.1255610650
O	2.1100875045	-1.7524743865	0.4331915864
H	-1.5799263462	3.3823158197	1.0439071177
H	-3.7302729324	0.7722078171	-0.3935704333
C	3.2441125787	-1.3212994056	-0.1440249309
O	3.3080879799	-0.9489238805	-1.3010002042
C	4.3931638553	-1.3505024502	0.8143687480
H	5.3277679601	-1.2069278243	0.2747226830
H	4.2598316999	-0.5400057218	1.5374592779
H	4.4067119844	-2.2939376683	1.3630453405
O	-4.3930521311	-0.6917905959	0.6261269922
H	-4.3337876046	-0.4709304133	1.5645250378
H	-3.4586855186	-0.9921965478	0.3506377197
O	-1.5462970453	-2.4270337972	2.1687333120
H	-1.7079912482	-2.0004544956	1.2747772466
H	-0.5855439380	-2.4515239411	2.2642652839
O	-1.7934947199	-3.1704008644	-1.9395733028
H	-0.8621948244	-3.4228380608	-1.9418266365
H	-1.8818658690	-2.4544358435	-1.2252917014

beta-D-Glc-6Ac-H
0 1

C	-0.0996655787	-0.5393868901	0.8141490389
C	-0.0794498526	0.7084672123	-0.0766908372
C	1.3632479416	1.1811145051	-0.2384753747
C	2.2533190740	0.0516729392	-0.7387888365
C	2.1085113930	-1.1499852301	0.1818279720
H	-0.4879720771	0.4604473334	-1.0670156419
H	0.2688448429	-0.2676865603	1.8169076024
H	1.9335792562	-0.2480360699	-1.7476609204
H	2.4614271961	-0.9074047609	1.1991480363
H	1.7303297643	1.5021305139	0.7494169545
O	0.7426907826	-1.5357534422	0.2422703997
O	2.8330614991	-2.2118965265	-0.3461485702
O	3.6135795476	0.4444422161	-0.7415770737
O	1.4708150806	2.2529520594	-1.1591306202
C	-1.4743290355	-1.1479972107	0.9888856173
H	-1.4043761166	-2.0548701563	1.5910941918
H	-2.1508412042	-0.4369892129	1.4641136029
C	3.0080093101	-3.2904976595	0.5729115709
H	3.6303482872	-4.0313177374	0.0713596671
H	3.5133931066	-2.9372232954	1.4784068609
H	2.0472865982	-3.7409233837	0.8379232019
O	-1.9951651252	-1.5581065554	-0.2887098613
H	3.6740854398	1.2775991942	-1.2324168334
H	1.2602868041	3.0753826785	-0.6777446715
C	-2.9706184435	-0.8332921874	-0.8529618974
O	-3.4989540590	0.1165552012	-0.2993152835
C	-3.2965367294	-1.3067439638	-2.2329307875
H	-4.2634569064	-0.9101103110	-2.5384608927
H	-2.5215344637	-0.9314364795	-2.9093375246
H	-3.2901861947	-2.3966095485	-2.2789425242
O	-0.8568608025	1.7353236586	0.5223500083
H	-1.4969123474	2.0806231426	-0.1442165822
H	-1.1766083247	1.6282736618	2.3392037284
H	0.0218302280	3.3922282718	0.9476901882

O	-2.5333201656	2.4950296140	-1.4586893306
O	-1.2683055165	1.5600624080	3.3078715054
O	0.5850363657	4.1585003596	0.7327748610
H	-2.9512361208	3.3601134574	-1.3497361504
H	-3.2298705767	1.8413112725	-1.2835790540
H	-1.3193174212	0.6111916139	3.4817631292
H	1.2227283344	4.2236596081	1.4558954909

beta-D-Glc-IN1
-1 1

C	-2.3927507173	0.0133700793	-0.1440762676
C	-1.5096881619	-1.1545215358	-0.6314767993
C	-0.1645073830	-0.5392137411	-0.9303730207
C	0.3801678811	0.1325575791	0.3077849374
C	-0.5261247219	1.2838020017	0.7038136633
H	-1.4134314313	-1.9116779108	0.1568041939
H	-2.5152208207	0.7168680156	-0.9817730739
H	0.3931640149	-0.5734931526	1.1525947802
H	-0.6082714298	2.0433062315	-0.0923351131
H	-0.2874746011	0.2069007897	-1.7344756730
O	-1.7904870078	0.6955684247	0.9613484670
O	-0.1676567845	1.8819681386	1.9178907988
O	1.7017870220	0.4295250373	-0.1006259948
O	0.8884233141	-1.4250491290	-1.2507088809
O	-2.1411013735	-1.6992812850	-1.7781726321
H	-1.6335310656	-2.4702781070	-2.0678720148
C	-3.7698171323	-0.4300772875	0.2972225203
H	-4.3221867355	0.4456103742	0.6561471200
H	-4.2969417685	-0.8478928637	-0.5669156331
C	0.7700954960	2.9463095707	1.7671861251
H	0.9904400264	3.3082977689	2.7714395464
H	1.6952686814	2.6002156106	1.2949792783
H	0.3321855010	3.7575722792	1.1731422598
O	-3.6384008314	-1.4000051542	1.3294203627
H	-4.5239468303	-1.6681310477	1.6064331740
C	2.1568079091	-0.7421419737	-0.9513417067
O	2.9695991097	-1.5331112997	-0.3002656762
C	2.7127304049	-0.1384238005	-2.2293256423
H	3.0619897785	-0.9476314742	-2.8753797380
H	1.9522640281	0.4409009596	-2.7595692930
H	3.5579949926	0.5120972438	-1.9877672428
O	2.7592762272	0.2041546307	2.5607710875
H	2.5775444110	0.4630853661	1.6401996731
H	2.5168749346	-0.7417931062	2.5660350460
O	1.6412061724	2.8837099810	-1.5785738177
H	1.6804930277	2.0570572601	-1.0611012972
H	1.0806601175	3.4738507755	-1.0582300184
O	2.0374140343	-2.4543748988	1.9201897779
H	2.6466067668	-3.1487511901	2.2011846574
H	2.3824115750	-2.1272302005	1.0206171165

beta-D-Glc-IN2
-1 1

C	1.6714144663	1.4012463442	-0.1339232574
C	0.3276376049	0.7330655156	0.1073133101
C	0.4147164643	-0.7248515595	-0.2831725228
C	1.3860970995	-1.4546414027	0.6119532238

C	2.7215039578	-0.7021906846	0.4506526992
H	0.0980565024	0.8221294226	1.1810705475
H	1.9719721890	1.3056407894	-1.1879223351
H	1.0707306631	-1.4093328578	1.6604053863
H	3.0967240802	-0.8351133904	-0.5795333066
H	0.7755626620	-0.8001139090	-1.3245838759
O	2.5848830341	0.6931492001	0.7132404307
O	3.6276843254	-1.2119801712	1.3715774714
O	1.5374408657	-2.8233426683	0.2872895431
O	-0.9393086159	-1.1192073175	-0.2217525666
O	-0.7812079809	1.1470485977	-0.6665314580
C	1.7473672258	2.8523278029	0.3001611080
H	1.4003780616	2.9401879810	1.3372707431
H	2.7893352452	3.1873185322	0.2449623069
C	4.9860815040	-0.8845265642	1.0775069670
H	5.5954483802	-1.3556371899	1.8485907403
H	5.2648852411	-1.2806288058	0.0949202882
H	5.1433565410	0.1975631535	1.0988254188
O	0.9236761951	3.6079102386	-0.5777843515
H	1.6925480075	-2.9056927306	-0.6660453559
C	-1.7421495317	0.0366195684	-0.6434392423
C	-2.2135738121	-0.1744019980	-2.0711253282
H	-1.3710112755	-0.4175734984	-2.7236370320
H	-2.9383534681	-0.9931983003	-2.0928060515
H	-2.6961113201	0.7368727920	-2.4343252355
H	0.9574977457	4.5337855245	-0.3042444841
H	-2.1589222470	-0.0805665389	1.8024110858
O	-1.8670755551	-0.2937573163	2.7284747967
H	-0.9992431651	-0.7037710651	2.6196954826
H	-4.1531038325	0.8223163496	-0.3857519724
O	-5.0414229377	1.1155985483	-0.7216772012
H	-5.3844454027	0.3558620764	-1.2090259950
H	-3.6464131514	-1.1885951524	0.3641543645
O	-4.2138978830	-1.9982067927	0.4257701578
H	-4.8762416295	-1.8887176973	-0.2682547214
O	-2.7155412532	0.2377964439	0.2418743815

beta-D-Glc-IN3
-1 1

C	-0.9728818958	-1.0731034358	-0.1484903449
C	-0.3724302365	0.3242066676	-0.2374299682
C	-1.4393120731	1.2888526953	-0.6967069658
C	-2.6149292897	1.1856612226	0.2842839733
C	-3.0685358204	-0.2644738505	0.4917523095
H	-0.0257419797	0.6401408611	0.7562204220
H	-1.4432745268	-1.3240172217	-1.1103622137
H	-2.2938166964	1.5783835212	1.2559637547
H	-3.5139012946	-0.6657243574	-0.4361894810
H	-1.7842572847	1.0099747684	-1.7047364430
O	-1.9716362149	-1.0911256122	0.8673240716
O	-4.0004863094	-0.2802376153	1.5231819320
O	-3.6921611431	2.0032540643	-0.1374036134
O	-0.9140913120	2.6047476699	-0.7089478791
O	0.7172048644	0.2394732590	-1.1349569140
C	0.1691584197	-2.0500894753	0.1421434438
H	-0.0714792921	-2.6665110407	1.0113050183
H	0.3359331989	-2.7085053265	-0.7166574069

C	-4.7187734858	-1.5095629663	1.6258027449
H	-5.4775904803	-1.3640404877	2.3946127954
H	-5.2015885160	-1.7474337673	0.6715928729
H	-4.0561530098	-2.3288891031	1.9188406834
O	1.3631536993	-1.3461822864	0.4705895242
H	-3.9927486360	1.7040073475	-1.0092967671
H	-1.6247112207	3.2098443930	-0.9667369730
C	1.8618433976	-0.4241354170	-0.5248742450
C	2.5561994354	-1.1726000789	-1.6529886126
H	2.8378544536	-0.4636196754	-2.4358973363
H	3.4591297427	-1.6485028720	-1.2610672086
H	1.9060946125	-1.9344269642	-2.0906265420
H	2.1174967028	0.7822054405	1.6726822187
O	1.8652974815	0.9910681340	2.6125942585
H	1.1505534631	0.3752049806	2.8177866130
H	3.8727540922	1.0780084072	-0.8080814193
O	4.6508930532	1.4456146611	-1.3099169106
H	5.1720164745	0.6718439920	-1.5587697895
H	3.9314312710	-0.3762857510	0.8462125419
O	4.7088488625	-0.8310166843	1.2638197955
H	5.3746126771	-0.8725970857	0.5657453459
O	2.6469821153	0.4503900793	0.1153378647

beta-D-Glc-TS1a
-1 1

C	-1.8750944069	-1.0888879585	-0.6681461420
C	-0.4409743620	-1.3569966135	-0.1745579300
C	0.3645000802	-0.1031648572	-0.4827447692
C	-0.2948172605	1.1009520126	0.1690341126
C	-1.6845160999	1.2927992665	-0.3871618115
H	-0.4472452097	-1.5441350279	0.9084237267
H	-1.8403265068	-0.9642041314	-1.7618286124
H	-0.3784996454	0.9583441496	1.2567728689
H	-1.6770498435	1.4400457270	-1.4799333925
H	0.3330134175	0.0400131173	-1.5798727313
O	-2.4029953077	0.1009658986	-0.0737055724
O	-2.3113419990	2.3586554858	0.2479793455
O	0.6094549733	2.1501761787	-0.1250936253
O	0.0367501501	-2.4952096412	-0.8737131956
H	0.9038764909	-2.7335486736	-0.4914762166
C	-2.8362508404	-2.2157445330	-0.3606205009
H	-3.8351557154	-1.9316643952	-0.7105773452
H	-2.5149483854	-3.1111652799	-0.9031017569
C	-3.5410932021	2.7467106787	-0.3657903880
H	-3.8848727205	3.6370064432	0.1605695057
H	-3.3777356078	2.9837493426	-1.4227701643
H	-4.2913711015	1.9560893126	-0.2762659620
O	-2.8456324653	-2.4505430609	1.0432518475
H	-3.5013857055	-3.1340236836	1.2323501536
C	1.9154468400	1.7747142260	0.2987452157
O	2.1768812110	1.8777014512	1.5163959731
C	2.9345145118	2.1243028946	-0.7553830318
H	2.6026269686	1.8208416188	-1.7494311205
H	3.0788969360	3.2097614054	-0.7432779813
H	3.8849352657	1.6445430990	-0.5142839414
O	1.6880348457	-0.0415403173	-0.0316386135
O	3.0384308383	-0.8763839359	-2.2495418523

H	2.5968474652	-0.5133096882	-1.4459336557
H	2.8070561786	-1.8144229903	-2.2485212644
O	2.5528263910	-2.6709766717	0.4024872755
H	2.4643716494	-1.6991488816	0.3288185912
H	2.3979353498	-2.8611505589	1.3381982625
H	1.6353941334	-0.4364583801	1.7707387962
O	1.6100078252	-0.7608844385	2.6961225053
H	1.7369518637	0.0399174104	3.2215583966

beta-D-Glc-TS1b
-1 1

C	-2.5351492522	0.3003407482	-0.4645631472
C	-2.0901513672	-1.0698321806	0.0842354812
C	-0.6326779347	-1.2052592767	-0.2848864792
C	0.1916565975	-0.0667840004	0.3011844965
C	-0.3131972446	1.2189425840	-0.3293054248
H	-2.2106563556	-1.0971711144	1.1744106245
H	-2.4706978796	0.2612668694	-1.5631695294
H	-0.0063556924	0.0111785876	1.3849198493
H	-0.2154879072	1.2057783594	-1.4280304141
H	-0.5517440166	-1.1888171516	-1.3828478052
O	-1.6906084986	1.3434704248	0.0254926176
O	0.3546327637	2.3177228326	0.2050638941
O	1.5054005944	-0.4384740677	0.0485953057
O	0.0011727777	-2.3638184098	0.2260002213
O	-2.9068042950	-2.0510822274	-0.5346527083
H	-2.6562480060	-2.9178474529	-0.1853180629
C	-3.9585937575	0.6546719580	-0.0960712843
H	-4.1852547461	1.6494162706	-0.4958608076
H	-4.6348111857	-0.0724611892	-0.5583647099
C	0.1533187784	3.5236249901	-0.5379061438
H	0.7162513837	4.3048207792	-0.0263937025
H	0.5311922652	3.4020681262	-1.5589805549
H	-0.9059646467	3.7924810519	-0.5599557447
O	-4.0896849591	0.6398680844	1.3207859319
H	-4.9864060582	0.9197569260	1.5459526750
C	1.4073027315	-2.3032287853	0.0608308756
O	2.0672849541	-2.7445467370	1.0240197367
C	1.8798428692	-2.5472775085	-1.3544270398
H	1.8442132286	-3.6266865279	-1.5347071664
H	1.2703377894	-2.0391763092	-2.1021610056
H	2.9143540877	-2.2103052612	-1.4443596985
O	3.1983927318	2.4464814776	0.4649304256
H	3.4719703187	1.6876122148	1.0112382809
H	2.2275258580	2.3652208156	0.4428027264
O	2.6965727925	0.9193308365	-1.9824219793
H	2.2241771802	0.3456444483	-1.3343180157
H	3.0673347703	1.6119599761	-1.4114240129
O	3.1146370177	0.0919022127	2.0375664032
H	3.6598124303	-0.6898821504	2.1928261982
H	2.4893908825	-0.1631032239	1.2966916934

beta-D-Glc-TS2a
-1 1

C	-0.5548027174	1.1873923081	0.5043604136
C	0.2200760708	0.1133772807	-0.2483273871
C	-0.4169943408	-1.2369223894	0.0067670275

C	-1.8441608416	-1.2570772526	-0.4926087516
C	-2.5599649994	-0.0890578039	0.2001153933
H	0.1110642452	0.3395282912	-1.3246667364
H	-0.5783432009	0.9676890833	1.5829137907
H	-1.8724172942	-1.0951029620	-1.5785125769
H	-2.6114800709	-0.2773253268	1.2869326735
H	-0.4117569534	-1.4550806831	1.0866427740
O	-1.8889671526	1.1451304067	-0.0217239553
O	-3.8391905252	0.0173239366	-0.3352601260
O	-2.5389884494	-2.4405000942	-0.1394343250
O	0.4434866841	-2.1327422709	-0.6781275459
C	0.0083668571	2.5834070673	0.3143939646
H	-0.6652530428	3.3081471503	0.7802055114
H	0.9749255747	2.6297114552	0.8333271425
C	-4.7112147106	0.8444686942	0.4348450520
H	-5.6965051910	0.7746377728	-0.0258478022
H	-4.7625178794	0.4813900475	1.4672671044
H	-4.3754206076	1.8850754761	0.4255770907
O	0.1489828927	2.9564544009	-1.0481468597
H	0.9305618013	2.5046429364	-1.4224276010
H	-2.1310584772	-3.1849260686	-0.6038425772
C	1.8118602500	-1.8539129572	-0.4123309897
O	2.5587867847	-1.8910002368	-1.4124348506
C	2.2911685150	-2.3892379366	0.9170491219
H	1.5447310578	-2.2836850772	1.7056628818
H	2.5182819276	-3.4517473142	0.7835972472
H	3.2057513066	-1.8675999936	1.2076190409
O	1.5608455573	-0.0793641677	0.0830599428
O	2.4287310703	1.4938833318	-1.9974459549
H	2.2303511986	1.1022184790	-2.8585460839
H	2.2629349359	0.7813222460	-1.3403749579
H	2.9040525177	1.0821823037	0.6194056937
O	3.6626983079	1.6594541935	0.8312268439
H	3.9535028731	1.9988214870	-0.0255777078
H	1.6181561946	0.0064914191	1.9012290093
O	1.6530329859	0.1390090487	2.8735100414
H	1.3876338449	1.0592367195	3.0021450271

beta-D-Glc-TS2b
-1 1

C	1.5116056513	1.3500401046	-0.7085056959
C	0.1359001550	1.0951097013	-0.1213379659
C	-0.3283668683	-0.3094456666	-0.4626456788
C	0.6282578922	-1.3219037441	0.1543438976
C	2.0269267142	-0.9730177543	-0.3670194374
H	0.1859335759	1.2214600418	0.9694664369
H	1.4955235940	1.1932444777	-1.7974761183
H	0.6275666937	-1.2344035061	1.2506554592
H	2.0702708677	-1.1328103042	-1.4585700060
H	-0.2725683698	-0.4337465294	-1.5610562743
O	2.3672624634	0.3819558654	-0.0887854360
O	2.9577869770	-1.7817245274	0.2771724060
O	0.3504012939	-2.6529544243	-0.2439906349
O	-0.8987865047	1.9026684569	-0.6573424733
C	2.0719492784	2.7264043144	-0.4380591413
H	3.1000782377	2.7738819172	-0.8150261775
H	1.4645360648	3.4581071478	-0.9838407365

C	4.2513195481	-1.7526169040	-0.3246127083
H	4.8506324096	-2.5080265631	0.1836780706
H	4.1796736558	-1.9969431010	-1.3904629724
H	4.7203868751	-0.7722684473	-0.2028971798
O	2.0373342792	2.9828549977	0.9605618868
H	2.4201311294	3.8557570579	1.1177544044
H	-0.4695582624	-2.9283289863	0.2093581073
C	-2.1514425007	1.4502944503	-0.1559096160
O	-2.4678314099	1.7933760619	1.0045532188
C	-3.1740101173	1.4079040577	-1.2627720617
H	-2.7726005447	0.9361375070	-2.1611625752
H	-4.0610605446	0.8720057831	-0.9198301568
H	-3.4583275236	2.4386043823	-1.4996631609
O	-1.6639533813	-0.3355913083	-0.0449064735
H	-2.1900978223	-1.9443726895	0.7455818630
H	-1.5994474725	-0.2273880660	1.8337647241
H	-2.4808233414	-1.2657269822	-1.2639285352
O	-2.1177841198	-2.8367097328	1.1408072186
O	-1.5264973886	-0.3561271347	2.8022459046
O	-2.8708722328	-1.8860822934	-1.9249014295
H	-1.9061737184	-2.6663902646	2.0697006931
H	-1.7279387768	0.5147548563	3.1685126003
H	-3.3726004559	-1.3292262517	-2.5329822461

beta-D-Glc-TS3a

-1 1

C	0.3300156926	-0.5945505616	0.0429694334
C	0.3729193364	0.9018026889	-0.2634785955
C	1.6180099561	1.5010658710	0.3619937902
C	2.8427385838	0.7492295957	-0.1523635310
C	2.6795601111	-0.7530197272	0.0600843040
H	0.3968288056	1.0559539721	-1.3493600364
H	0.3542367626	-0.7376640301	1.1348841707
H	2.9454779259	0.9318541757	-1.2321030790
H	2.6549904984	-0.9890852955	1.1377297161
H	1.5642249270	1.3900334469	1.4548825095
O	1.4743684445	-1.2071806783	-0.5407308909
O	3.7429741250	-1.4018922681	-0.5576881337
O	4.0128145285	1.1534703387	0.5347778534
O	1.7992013003	2.8665996354	0.0217681930
O	-0.7766816560	1.5328036457	0.2923457270
C	-0.9338695552	-1.2348252848	-0.5188125171
H	-0.9188676007	-1.1367702035	-1.6179494097
H	-0.9405125016	-2.3079968871	-0.2801384658
C	3.8818110040	-2.7665262929	-0.1604939304
H	4.7598006222	-3.1535846661	-0.6773186075
H	4.0337039645	-2.8317540642	0.9223858168
H	3.0026666375	-3.3503516471	-0.4473474059
H	4.0864111160	2.1154920959	0.4528580430
H	1.1623865657	3.3966794892	0.5209172900
C	-1.9604510196	1.2731777416	-0.4068347254
O	-1.9528520498	1.3269775951	-1.6498041584
C	-3.1529815267	1.6958999720	0.4062426839
H	-4.0533675834	1.2650945758	-0.0355914656
H	-3.0626029065	1.3846232892	1.4474990733
H	-3.2257230986	2.7872715526	0.3603991193
O	-2.0590009776	-0.5964551052	0.0320816362

H	-1.8346960949	-0.5392232649	1.7612336461
H	-3.3498299935	-1.5399291300	0.7106600808
H	-3.2324817660	-0.8249757446	-1.2395932355
O	-1.7142735269	-0.5594710615	2.7420237264
O	-4.1071285403	-2.0288379758	1.1082937317
O	-3.8480492720	-0.9188947283	-2.0049171540
H	-2.6110554272	-0.5699165245	3.1004385612
H	-4.7688091687	-2.0735015602	0.4061555886
H	-3.6502396427	-0.1344869798	-2.5343763531

beta-D-Glc-TS3b
-1 1

C	0.4851197765	-1.1253478054	0.1632287959
C	-0.1587837414	0.1346846412	-0.4197939427
C	0.6690540255	1.3388144690	0.0139728745
C	2.1380947239	1.1322012050	-0.3596852506
C	2.6487804313	-0.2145024642	0.1515002348
H	-0.0913217262	0.0664072810	-1.5226996387
H	0.4991934060	-1.0515422618	1.2620269195
H	2.2347424981	1.1394889754	-1.4514647119
H	2.6548360172	-0.2334702508	1.2561198502
H	0.5892123560	1.4428930256	1.1093503124
O	1.8157967046	-1.2652767446	-0.3207297004
O	3.9319783955	-0.4193868330	-0.3432957359
O	2.9387653599	2.2007564639	0.1177907703
O	0.1596314021	2.5056313225	-0.6162361530
C	-0.2975365510	-2.3647129883	-0.2308741279
H	-0.3188620726	-2.4849550787	-1.3197002066
H	0.1532102039	-3.2562656628	0.2092580050
C	4.6260351565	-1.4867108298	0.3026524413
H	5.6401899696	-1.4887684363	-0.0967358869
H	4.6576514558	-1.3170475404	1.3846080300
H	4.1497933724	-2.4489207436	0.0941247544
O	-1.6140367551	-2.2783981377	0.3131526346
C	-2.5139043413	-1.3626025746	-0.2360469387
O	-3.5252088553	-1.1680441201	0.4643751559
C	-2.6247864303	-1.3654050480	-1.7441781683
H	-3.3666986233	-0.6196159434	-2.0310865081
H	-1.6905950184	-1.1636872939	-2.2673335313
H	-2.9857606799	-2.3550299011	-2.0452350519
O	-1.4922440425	0.2256563337	-0.0028100379
H	-2.0796003277	1.7988021910	0.7877934183
H	-1.6149353344	2.5309654393	-1.8759019702
H	-1.5706114914	-0.0132208930	1.7679492788
O	-2.4205714564	2.4815660672	1.4012249772
O	-2.3572240711	1.9194433338	-1.9858738383
O	-1.6223693992	-0.0384455324	2.7519463846
H	-2.4190128117	2.0245496297	2.2547909549
H	-2.1746101689	1.2460570779	-1.2910298297
H	-2.1986366271	-0.7891994881	2.9464501286
H	2.8609015880	2.2416595990	1.0833773508
H	0.6695676825	3.2663875169	-0.3024190427

D-Galactopyranoside

alpha-D-Gal-2Ac

-1 1

C	-2.0474767849	-0.7149110829	0.4981395553
C	-1.5461352504	0.6719527349	0.1043801763
C	-0.0270980750	0.7396615905	0.3589157988
C	0.6302684133	-0.4028836257	-0.4176220280
C	0.0145993714	-1.7625440401	-0.0738725318
H	-2.0378055016	1.4138950966	0.7510317395
H	-1.7985172391	-0.8520561938	1.5601812774
H	0.5219296850	-0.2328391568	-1.4923300237
H	0.3786731931	-2.5532512390	-0.7405738137
H	0.1186609483	0.5402148077	1.4364135651
O	-1.3815524453	-1.7214490462	-0.2765376075
O	0.3441498127	-2.0662002678	1.2555733925
O	2.0261707905	-0.3936946542	-0.0792472701
O	0.5339145835	1.9712005375	0.0169773985
O	-1.8616448776	0.9224155339	-1.2582226078
H	-1.5925613022	1.8355841693	-1.4744117924
C	-3.5386241152	-0.9641364838	0.3885944862
H	-3.7593092187	-1.9238211264	0.8705055639
H	-4.0587064778	-0.1711046662	0.9403082578
C	-0.1436346442	-3.3380068224	1.6720691448
H	0.3070632405	-3.5466752834	2.6425413269
H	-1.2340803503	-3.3283220425	1.7706961393
H	0.1491892051	-4.1141678523	0.9551524946
O	-3.9600863080	-0.9992675072	-0.9700733794
H	-4.8734921782	-1.3125338874	-0.9893818803
C	2.8931613295	-0.9356910875	-0.9452691132
O	2.5438204940	-1.4808621979	-1.9770478089
C	4.3102944203	-0.7567988585	-0.4999169724
H	4.5931023541	0.2874693467	-0.6667980820
H	4.4045666699	-0.9684275869	0.5668139798
H	4.9637009339	-1.4059240398	-1.0807035797
O	-0.4321874021	3.5972265555	1.8481612945
H	-1.3789262299	3.6358150027	1.6621938241
O	-0.6998875394	3.4719334680	-1.6361324470
H	-1.1385540896	4.1451831668	-1.1001568926
O	2.7647418143	2.1635323325	1.3477914348
H	3.1612875190	1.2843590130	1.3012719784
H	-0.0571208615	2.9874171241	1.1453509758
H	-0.1560368000	2.9103650612	-0.9828942258
H	1.9198389022	2.0859962078	0.8043862620

alpha-D-Gal-2Ac-H
0 1

C	2.3310054192	-0.0145439252	-0.3546444919
C	1.2975498812	1.1022892038	-0.2429722412
C	-0.0946677628	0.5286863484	-0.5528177507
C	-0.3611739823	-0.6912308923	0.3251410830
C	0.7707819199	-1.7134341266	0.2096581212
H	1.5362425366	1.8649844991	-0.9955511197
H	2.2945164325	-0.3696576622	-1.3939254301
H	-0.4479645648	-0.3963772629	1.3740356907
H	0.6466938378	-2.5185889768	0.9434194179
H	-0.1233671120	0.2272761924	-1.6051747023
O	1.9988591611	-1.0949917094	0.5257441239
O	0.7617752435	-2.2321935959	-1.0911250128
O	-1.5677885604	-1.3377773353	-0.1003230117

O	-1.1053584614	1.5121744968	-0.3888742217
O	1.3245432087	1.6679345428	1.0588737570
H	0.8179051665	2.4933442990	1.0477065127
C	3.7758536523	0.3666458996	-0.0987353084
H	4.4041356107	-0.4815003032	-0.3935544669
H	4.0219687707	1.2212190378	-0.7410703755
C	1.7410784905	-3.2495013894	-1.2865991317
H	1.5713797030	-3.6647683301	-2.2799212994
H	2.7530476723	-2.8355532929	-1.2320271239
H	1.6267887267	-4.0372907660	-0.5330534922
O	3.9961535351	0.6779902495	1.2713756281
H	4.9498330699	0.7397165247	1.4107728603
C	-2.6641536717	-1.2773108763	0.6701310346
O	-2.7205582933	-0.6466698765	1.7118500660
C	-3.7762945615	-2.1210310726	0.1350016731
H	-4.7325387528	-1.6940708544	0.4359125581
H	-3.7156626112	-2.2185753326	-0.9488314210
H	-3.6784863726	-3.1158667305	0.5822078741
O	-1.6464545232	1.9102998499	2.2620029800
H	-2.0526682781	1.0275336599	2.3225633511
H	-1.2640815171	1.6902847135	0.5704733437
H	-2.3738391465	2.5359296688	2.3826858467
O	-0.1601300947	4.1875537324	-0.7823054734
H	0.8009674618	4.0889415330	-0.7809623845
O	-3.5947345488	0.7489735089	-1.4245603687
H	-3.4533288355	-0.0613974907	-1.9307349239
H	-0.5044541793	3.2763542486	-0.7560958582
H	-2.7106355998	0.9948753127	-1.0888088127

alpha-D-Gal-3Ac-a
-1 1

C	-2.1456201368	-0.3848137259	-0.4461207912
C	-1.0072415065	-1.3339689377	-0.0548954747
C	0.1462734702	-0.4759537543	0.4504200899
C	-0.2916116521	0.4070334464	1.6157705628
C	-1.5073303345	1.2342137503	1.1749586817
H	-0.6894443698	-1.8825899559	-0.9518448151
H	-1.7713177709	0.2726494374	-1.2445747193
H	-0.6297630346	-0.2331184974	2.4387992799
H	-1.9451585562	1.7655016652	2.0285941700
H	0.5128855204	0.1418925888	-0.3720609305
O	-2.5408105677	0.4030132284	0.6808170087
O	-1.0798828652	2.1550183675	0.2065929397
O	0.7474420058	1.2282448364	2.0872201355
O	1.2306196658	-1.2913979352	0.9255734754
O	-1.3685852983	-2.2398548887	0.9782114015
H	-2.2688406849	-2.5555808046	0.7907214901
C	-3.3782883557	-1.0738597413	-0.9899627938
H	-4.1245213308	-0.3148260993	-1.2500312644
H	-3.0969356688	-1.6214269008	-1.8959749898
C	-2.1122911828	3.0325948792	-0.2334653223
H	-1.6347434631	3.8042922792	-0.8386219472
H	-2.8512673774	2.4977145189	-0.8391471586
H	-2.6115055349	3.4957422561	0.6255115436
O	-3.9028541804	-1.9666465957	-0.0067216551
H	-4.6039392228	-2.4950813704	-0.4106100621
C	2.1644517237	-1.6565780323	0.0355829692

O	2.0766665075	-1.4038738621	-1.1540137797
C	3.3062936861	-2.3740808044	0.6817635279
H	3.8284123374	-1.6765399658	1.3430748375
H	2.9306483878	-3.1995802867	1.2904172129
H	3.9884943877	-2.7430629228	-0.0822465442
O	2.3572830034	1.8179410192	0.2365919105
H	1.3862833448	1.4897088986	1.3103837992
H	2.7003941065	2.7003839095	0.4308992034
O	4.2646678170	0.6045437762	-1.0835860081
H	3.5318709520	1.0584412712	-0.5620004086
H	3.8467798292	-0.1873689158	-1.4480858434
O	0.9498526756	1.9992225943	-1.9415397334
H	1.5144248108	1.9457739051	-1.1021206637
H	0.0440090624	1.9789223492	-1.5998104440

alpha-D-Gal-3Ac-b
-1 1

C	-1.3605104492	-0.3404425111	-0.9216351692
C	0.1206180817	-0.5226383951	-0.5484296322
C	0.6263202300	0.8705504279	-0.1336027540
C	-0.1883909766	1.4004200872	1.0389344867
C	-1.6780231423	1.3972409681	0.6785215800
H	0.6485872477	-0.8097330429	-1.4773693706
H	-1.4097106453	0.4082132914	-1.7277812566
H	-0.0440757902	0.7440154334	1.9009910423
H	-2.2922726949	1.6051465672	1.5636727147
H	0.5601433072	1.5744862620	-0.9682965557
O	-2.1020737677	0.1401118499	0.2092998690
O	-1.8626530867	2.4057249492	-0.2831723755
O	0.2239075433	2.6944352253	1.4390044402
O	1.9856469937	0.7827332921	0.3293587494
C	-2.0910895498	-1.5668869452	-1.4501291143
H	-3.0646159129	-1.2381705818	-1.8270656932
H	-1.5188732358	-1.9692310972	-2.2962844384
C	-3.2272323641	2.5935643411	-0.6454056022
H	-3.2714710727	3.4939519800	-1.2583973299
H	-3.6000670931	1.7400168538	-1.2206065734
H	-3.8442854984	2.7266508656	0.2508293534
O	-2.3475724661	-2.5757467582	-0.4867116128
H	-1.5424378738	-3.1246530121	-0.4060593851
H	-0.0268670556	3.3180043655	0.7400465233
C	3.0355165204	0.9264294902	-0.4882856323
O	4.1351897157	0.6693791026	-0.0218820443
C	2.8269739563	1.4046563540	-1.8928243037
H	2.1535166539	0.7362084762	-2.4351690298
H	2.3829150871	2.4041120067	-1.8847935490
H	3.7943730612	1.4371069533	-2.3902598759
O	0.3536780311	-1.4525672281	0.4633256224
O	2.9213326120	-2.0665929806	0.6802864794
H	3.4476901473	-1.2591393504	0.7607725630
H	1.9710079011	-1.7616986369	0.6233705104
H	-0.6632830255	-1.5023567400	1.7143514025
O	-1.2891745419	-1.5719237392	2.4981788375
H	-2.0899366922	-1.1424157482	2.1690629994
H	0.3037641649	-2.8250755381	-0.0945497924
O	0.1612752324	-3.7831506153	-0.4590679245
H	0.4607146279	-3.7683274022	-1.3772742089

alpha-D-Gal-3Ac-H
0 1

C	2.2853153186	-0.7864602641	0.4738379961
C	1.1340534256	-1.6772367392	-0.0135782781
C	-0.1207244468	-0.8090244765	-0.0395846804
C	0.0777308771	0.3983090029	-0.9575091750
C	1.2702111643	1.1896315917	-0.4130518111
H	0.9905523303	-2.4857767126	0.7141921511
H	2.0599305344	-0.4805012556	1.5042683135
H	0.3346153480	0.0887600898	-1.9736806733
H	1.5260149596	2.0125490364	-1.0913654721
H	-0.2767242837	-0.4428194573	0.9788578843
O	2.4203787397	0.3707222991	-0.3581759713
O	0.9176431968	1.6883410690	0.8457769999
O	-1.3512654782	-1.5308852211	-0.2780681250
O	1.4862071710	-2.2245918049	-1.2786867162
H	0.7088534020	-2.2507579592	-1.8637449217
C	3.6341407342	-1.4904454447	0.4955502409
H	4.3628968361	-0.8400758121	0.9855939272
H	3.5306925702	-2.4052384421	1.0932087168
C	1.8691724974	2.6118503640	1.3680908268
H	1.4433762336	3.0192402091	2.2859229090
H	2.8154664194	2.1119472559	1.5985393573
H	2.0483174879	3.4211287310	0.6503336995
O	4.1539787739	-1.7793421062	-0.7948426953
H	3.4450544948	-2.1976207233	-1.3083639142
C	-1.7531938204	-1.8802490997	-1.5023862191
O	-1.0085004413	-1.8830853097	-2.4729978944
C	-3.2029240591	-2.2335807563	-1.5407526983
H	-3.4291415832	-2.7691801372	-2.4610912354
H	-3.4726300346	-2.8271888148	-0.6656866783
H	-3.7751795619	-1.3000160900	-1.5084490087
O	-1.0735471805	1.2191560232	-0.9848540119
H	-1.4821317665	1.1456599405	-1.8811088306
H	-2.3970750133	0.8597756349	0.2878521181
H	-1.0321618167	2.9150398338	-0.1580951052
O	-2.0184120338	0.7462515221	-3.4657938049
O	-3.0810981001	0.5625164965	0.9162526211
O	-1.1343056111	3.8183981055	0.1929031086
H	-1.7141415092	-0.1666791028	-3.5881255779
H	-1.5601032183	1.2610710083	-4.1442453570
H	-3.0285588081	-0.4017899497	0.8642278569
H	-0.5629226682	3.8419402753	0.9711620286

alpha-D-Gal-4Ac-a
-1 1

C	-1.6546393321	0.6889594416	0.4599159814
C	-0.1291145070	0.6807676790	0.4202035285
C	0.4083037061	-0.7564223926	0.3751936038
C	-0.2879375510	-1.5034145976	-0.7660849294
C	-1.8037981446	-1.4329570189	-0.6314315660
H	0.2598775866	1.2059992615	1.2965636714
H	-1.9436152571	0.2445157938	1.4226648863
H	-0.0308916042	-1.0166255744	-1.7180278977
H	-2.3069026013	-1.8696213947	-1.5033739394
H	0.0939544446	-1.2245727738	1.3257420458

O	-2.2104916121	-0.0779789181	-0.6114860081
O	-2.1897947642	-2.1122445253	0.5325946877
O	0.1071213887	-2.8641865050	-0.7985379730
O	0.2910773005	1.3880861846	-0.7701403188
C	-2.2847604079	2.0753265865	0.3980195352
H	-3.3369544112	1.9970573624	0.6813237678
H	-1.7768794137	2.7172755316	1.1290374226
C	-3.6032256234	-2.1632588426	0.7022729540
H	-4.0073549758	-1.1718472914	0.9314294393
H	-4.0835465494	-2.5519757562	-0.2035402088
H	-3.7979877876	-2.8367194293	1.5371187168
O	-2.2729795456	2.6640055121	-0.8955159403
H	-1.3635496080	2.6736770117	-1.2251658197
H	1.0768226798	-2.8719340476	-0.7830305278
C	1.3521917852	2.2074493893	-0.7090729841
O	2.0027113318	2.3910636180	0.3034791711
C	1.5835438386	2.9154699008	-2.0083491165
H	1.2539662996	2.3107147693	-2.8535957306
H	0.9964326772	3.8401932496	-1.9913510389
H	2.6385138131	3.1716237653	-2.1020158554
O	1.7913667886	-0.8226518013	0.2294605737
H	3.1741244858	-2.6209537833	2.2042097794
O	2.6615296227	-2.9469693878	1.4542462239
H	2.3356305682	-2.1238086112	0.9743336473
O	2.8274331230	0.3910199183	2.3003314667
H	2.4434406042	-0.0812643458	1.4998693375
H	2.6914282657	1.3233173614	2.0835842631
H	2.3191147100	-0.4339219936	-1.2023719434
O	2.7123581991	-0.2021295441	-2.1057743942
H	1.9568132174	0.0485514076	-2.6524269012

alpha-D-Gal-4Ac-b
-1 1

C	0.2748294815	0.4989347409	-1.0823290109
C	0.4038376344	-1.0039304016	-0.8369266966
C	1.8424656825	-1.3533563353	-0.4558277770
C	2.3020304559	-0.4911737960	0.7118578735
C	2.1132364181	0.9855850465	0.3751643534
H	0.1167551551	-1.5500112187	-1.7390917759
H	0.8923011932	0.7233358470	-1.9645735812
H	1.6772339625	-0.7081359956	1.5887548619
H	2.3225680105	1.6168080067	1.2481010237
H	2.4789768561	-1.1436760435	-1.3241910795
O	0.7668522008	1.2384176692	0.0432694735
O	2.9850644123	1.3125902224	-0.6738445348
O	3.6679770907	-0.7059139810	1.0152048898
O	1.9799789338	-2.7082422128	-0.0623871264
O	-0.4317343115	-1.4214563189	0.2567592512
C	-1.1378896636	1.0112379456	-1.3631426972
H	-1.0199630055	2.0486368727	-1.7235137499
H	-1.5269842221	0.4253291899	-2.2141621239
C	2.9237246978	2.6885981540	-1.0379654024
H	3.7514145289	2.8720062268	-1.7233672807
H	1.9775294224	2.9211218314	-1.5375042030
H	3.0325605119	3.3242528164	-0.1513240628
H	3.7688044866	-1.6289849001	1.2880160496
H	1.9474493107	-3.2662103546	-0.8517481414

C	-1.6561445570	-1.8854861567	-0.0208474600
O	-2.0534778813	-2.0862956893	-1.1561405346
C	-2.4685237968	-2.1130600769	1.2138782389
H	-1.8392851312	-2.4372775446	2.0436200449
H	-2.9327832002	-1.1590127008	1.4850146829
H	-3.2507210648	-2.8433664937	1.0085723037
O	-2.0031591340	0.9634448818	-0.2666012322
H	-1.4153679815	0.8348646243	1.2944779576
O	-1.1528597725	0.8475119587	2.2577274353
H	-0.1876012909	0.8758643256	2.2249062161
H	-2.2196521455	2.4205743831	0.1774164945
O	-2.3342868903	3.3936326738	0.4605803534
H	-1.8619773734	3.4645879209	1.2992590325
H	-3.3363503762	0.3621805326	-0.7006027480
O	-4.2238332645	-0.0374089623	-1.0152545958
H	-4.0074885328	-0.9565021080	-1.2211888621

alpha-D-Gal-4Ac-H
0 1

C	-0.1827326556	0.6517325015	-0.5039094054
C	0.1507541642	-0.7758414509	-0.0876511038
C	-1.0677671473	-1.4076235082	0.5883880830
C	-2.2922866038	-1.2761134309	-0.3119665593
C	-2.4952073256	0.1726647464	-0.7482626766
H	1.0153063113	-0.7839121578	0.5823918137
H	-0.4084183640	1.2214987149	0.4096387798
H	-2.1231369515	-1.8667244712	-1.2214454821
H	-3.2781402274	0.2477843606	-1.5119810264
H	-1.2470156848	-0.8701689708	1.5292070986
O	-1.3189410183	0.6589546471	-1.3679448809
O	-2.8404089598	0.9287691873	0.3788281029
O	-3.4702012851	-1.7085145399	0.3431347306
O	-0.8920879949	-2.7923853471	0.8299782952
O	0.4603223945	-1.5201821289	-1.2801889324
C	0.9468110304	1.3617784733	-1.2348137169
H	1.3064955647	0.7615893147	-2.0779443054
H	0.5633866314	2.3110637300	-1.6214212145
C	-3.1039735845	2.2956661697	0.0747140282
H	-3.5114310268	2.7474718595	0.9791056975
H	-2.1853949237	2.8191381608	-0.2118990536
H	-3.8355228301	2.3732615916	-0.7379774480
H	-3.3750236064	-2.6524540000	0.5349773919
C	1.3797714236	-2.4910190578	-1.2150627813
O	2.0097702735	-2.7476383208	-0.2018617027
C	1.5720498037	-3.1680431386	-2.5337808853
H	0.6139425259	-3.3253629556	-3.0309258565
H	2.1823095245	-2.5128743505	-3.1641952810
H	2.0936342192	-4.1124986169	-2.3872820311
H	-0.2328004369	-2.9092380069	1.5286536575
O	2.0084715951	1.6451736529	-0.3326569624
H	2.6790387267	0.9233050674	-0.3880638786
H	1.3773581589	3.2944674613	0.4560694560
H	1.6569415810	1.2955848335	1.5508787602
O	3.7478774515	-0.3975624196	-0.5151828637
O	0.7621636297	4.0011697978	0.7135035627
O	1.4974021332	1.0790936520	2.4870695057
H	3.8452796368	-0.6288557359	-1.4501231387

H	3.3077278644	-1.1655485866	-0.1163197053
H	-0.1140278398	3.6142721136	0.5820128921
H	0.6294717121	0.6537586693	2.4947354363

alpha-D-Gal-6Ac
-1 1

C	0.2411357703	-0.1793182947	-0.0088373613
C	-1.1571455959	-0.4774746775	-0.5753388647
C	-2.0267619000	0.7408965991	-0.2308823349
C	-1.4093819527	2.0049633029	-0.8207387808
C	0.0315430349	2.1721553523	-0.3389142080
H	-1.5585218658	-1.3414317971	-0.0122796971
H	0.1753807892	-0.0384846181	1.0794928152
H	-1.3941822400	1.9198878297	-1.9112718259
H	0.5414644924	2.9783729743	-0.8808187944
H	-2.0732966625	0.8437316710	0.8634223565
O	0.7825432253	1.0102089841	-0.5986920832
O	-0.0147361143	2.4734927764	1.0334315161
O	-2.1850781130	3.1548091623	-0.5187957356
O	-3.3373562586	0.5427078334	-0.7438140344
O	-1.1313737188	-0.7382912216	-1.9439918422
C	1.2257880484	-1.2919649754	-0.2958295747
H	0.9088921806	-2.2070680426	0.2073421117
H	1.3362269225	-1.4750529566	-1.3648730840
C	1.2716009201	2.7266051966	1.5906171667
H	1.1131162350	3.0735930565	2.6118507762
H	1.8779351374	1.8144779972	1.6038334573
H	1.7914192606	3.5010754915	1.0140305278
O	2.5053958664	-0.9512052076	0.2690260593
H	-2.1165830915	3.3193067914	0.4342540880
H	-3.8494704254	1.3428472155	-0.5563540117
C	3.4204852703	-0.3757189899	-0.5236587376
O	3.2648620492	-0.2077325789	-1.7215307069
C	4.6360855825	0.0383261467	0.2440240121
H	5.4460260305	0.2726972711	-0.4447530182
H	4.3833214939	0.9306074416	0.8256065501
H	4.9348074914	-0.7466262479	0.9405926249
O	-0.6154466882	-3.2410946793	-2.3621153974
H	0.3309693668	-3.3907057385	-2.2491467630
H	-0.7626657669	-2.2550741373	-2.1858590224
O	0.8568740241	0.0522399061	-3.5001278156
H	0.1134957671	-0.1897540926	-2.8761204514
H	1.6478994278	0.0655105256	-2.9395501369
O	-3.4575914346	-1.6639283343	-2.6765771497
H	-4.0938076646	-1.0931228536	-2.2273698572
H	-2.5679862242	-1.2784129116	-2.4007487833

alpha-D-Gal-6Ac-H
0 1

C	0.0055089822	-0.6313455584	0.4086145411
C	0.2662851342	0.6851874819	-0.3313055653
C	1.7673837347	0.7969233832	-0.6021382377
C	2.2469995511	-0.4326968861	-1.3630844380
C	1.8657878030	-1.7095543223	-0.6150529832
H	-0.0490958824	1.5173673552	0.3144828009
H	0.5512077675	-0.5880480546	1.3620427405
H	1.7468883531	-0.4646608932	-2.3393030796

H	2.0686388625	-2.5967097050	-1.2276689000
H	2.2917308012	0.8519511068	0.3599888606
O	0.4740361369	-1.7397798359	-0.3647742067
O	2.6051212468	-1.7598398210	0.5734993735
O	3.6532121831	-0.4307540812	-1.5331373532
O	2.0836754515	1.9272651977	-1.3992469632
C	-1.4415335119	-0.8899102292	0.7603502856
H	-1.5342925185	-1.8034033155	1.3543856402
H	-1.8387635895	-0.0446167559	1.3284228159
C	2.4015827461	-2.9645718743	1.3070373296
H	3.1121768874	-2.9548605324	2.1336104938
H	1.3820402918	-3.0169794498	1.7024812617
H	2.5906261769	-3.8353152131	0.6682045566
O	-2.1842068922	-1.0516188499	-0.4571064627
H	3.8855397737	0.3393389575	-2.0710558394
H	1.9729497544	2.7276758162	-0.8665864596
C	-3.5084539670	-0.8832906068	-0.4026726293
O	-4.1052953347	-0.6795521764	0.6400535590
C	-4.1326693851	-0.9866762342	-1.7589615152
H	-5.1914820265	-0.7402312225	-1.6981939698
H	-3.6212411020	-0.3112285959	-2.4497676556
H	-4.0092819763	-2.0071286088	-2.1325017314
O	-0.4068706172	0.7692478610	-1.5827815970
H	-1.3532399021	0.9433693887	-1.4000971518
H	-0.2569154890	2.8099409224	-1.7827893058
H	-0.5364600094	-0.7668225408	-2.6600781133
O	-2.7649760476	1.9485582427	-0.6955178499
O	-0.6653864384	3.6656234693	-1.5648934217
O	-0.6078573466	-1.6288970297	-3.1125478225
H	-3.5596847411	1.9545911867	-1.2462063100
H	-2.2631794547	2.7480622202	-0.9431638029
H	-0.1049581954	4.0380298832	-0.8707797371
H	-0.3926302804	-2.2486934400	-2.4011823774

alpha-D-Gal-IN1
-1 1

C	-2.7007275374	-0.3110077322	-0.0836821807
C	-1.5369029330	-1.3030827916	-0.3110981829
C	-0.3761287447	-0.4245731988	-0.7267050846
C	-0.0793476465	0.5945607089	0.3496104510
C	-1.2539724102	1.5380707353	0.5142413053
H	-1.8302709007	-1.9851742301	-1.1196300450
H	-2.9220939703	0.1503532427	-1.0560146672
H	0.0618689992	0.0951963006	1.3163730584
H	-1.1394779486	2.2304193917	1.3559114904
H	-0.6588494112	0.0890853537	-1.6562347011
O	-2.3657206184	0.7168953064	0.8625770847
O	-1.4639747680	2.2414543636	-0.6743752096
O	1.1432948546	1.1310067323	-0.1102670947
O	0.8882804869	-1.0380293543	-0.8848482056
O	-1.1799363430	-2.0425905426	0.8484548034
H	-1.9937526312	-2.2401398827	1.3449170215
C	-3.9792895132	-0.9647288546	0.3954007230
H	-4.7579575025	-0.2013563314	0.4993373358
H	-4.2956916504	-1.6959298030	-0.3561476859
C	-2.5311880164	3.1825161931	-0.5883810149
H	-2.5146051322	3.7622959912	-1.5112602108

H	-3.4957116160	2.6735362583	-0.4937669630
H	-2.3840980472	3.8495147636	0.2689715419
O	-3.7436717083	-1.6056739671	1.6499447261
H	-4.5063981313	-2.1589189801	1.8641023592
C	1.9080337910	-0.0128014218	-0.6355267722
O	2.7856126592	-0.4676854667	0.2557998879
C	2.5102670806	0.4291701262	-1.9564930446
H	3.0534843629	-0.4075151516	-2.4031002782
H	1.7297426712	0.7624115304	-2.6451834066
H	3.2096847942	1.2513772023	-1.7789888805
O	4.0182772852	1.6649659402	1.3227214346
H	3.5468169067	0.8841282398	0.9322764007
H	3.3362802038	2.3396083657	1.4292824246
O	5.2229754780	-0.9962357624	-0.6809903698
H	5.7580679628	-1.1676977541	0.1036697242
H	4.3065011004	-0.8138179661	-0.3396972436
O	1.4861590795	-2.1007658241	1.9871133462
H	0.5789815834	-2.1163570483	1.6376887782
H	1.9727173007	-1.5051647730	1.3635980344

alpha-D-Gal-IN2
-1 1

C	1.5772734473	1.2704742666	-0.0363379136
C	0.4419935832	0.8853900950	-0.9753417525
C	0.3577747458	-0.6071685128	-1.2844490190
C	0.6457681510	-1.4699825108	-0.0551744242
C	1.9034171198	-0.9966604296	0.6534993848
H	0.5476933838	1.4548026167	-1.9089926273
H	2.5027633883	1.2794090258	-0.6266482268
H	-0.1650718570	-1.3343319491	0.6651766373
H	2.0696753904	-1.5524514602	1.5845863463
H	1.0006597304	-0.9065071363	-2.1158940182
O	1.7169751661	0.3533355017	1.0532742371
O	2.9963835821	-1.1448084676	-0.2077917509
O	0.7457727898	-2.8401974990	-0.3958150758
O	-0.9987488080	-0.7370072265	-1.6914703845
C	1.3773659428	2.6360257740	0.6119439129
H	0.5187504599	2.5963608440	1.2918394203
H	2.2692877115	2.8860110130	1.1914080116
C	4.2415429137	-0.8226440408	0.4073222417
H	4.3060047186	0.2486654893	0.6245613540
H	4.3652038571	-1.3906409036	1.3365896075
H	5.0247218522	-1.0981610119	-0.2988275372
O	1.2122686459	3.6569237941	-0.3607282244
H	0.3111046346	3.6120174230	-0.7099502648
H	-0.1637305837	-3.1907435387	-0.3116911291
C	-1.8028575680	0.1801258258	-0.9016420833
O	-2.4157243852	-0.4319356623	0.1202042161
C	-2.7479889457	0.8908453519	-1.8545127309
H	-2.1909369306	1.4191214616	-2.6327892494
H	-3.4122377685	0.1580462555	-2.3206098709
H	-3.3528789369	1.6075296706	-1.2929421520
O	-0.8294875577	1.1371497263	-0.3917564307
O	-1.0098534035	0.2404749457	2.4607223307
H	-0.1042114333	0.4209626077	2.1612450275
H	-1.4912406147	0.0212463642	1.6310743126
H	-2.1685709114	-2.0701080394	0.1303899973

O	-1.9780699286	-3.0503927091	0.1859282580
H	-2.5480983901	-3.4640358827	-0.4744496440
H	-3.4782917060	0.5645674819	0.9166514373
O	-4.0823919700	1.1528691920	1.4474676135
H	-3.5380983153	1.4658633338	2.1810002828

alpha-D-Gal-IN3
-1 1

C	0.5018785961	-1.2784856044	0.7852313118
C	0.2738119757	0.1374456457	1.3074619878
C	1.4932283382	1.0155415500	1.0332460019
C	1.8683531054	0.9450742017	-0.4403149878
C	2.0452921086	-0.5074289193	-0.8744750094
H	0.0943144614	0.0967615353	2.3915570138
H	1.2821691369	-1.7820952816	1.3683270749
H	1.0483892992	1.3657247412	-1.0375143846
H	2.1850918131	-0.5767233800	-1.9605548973
H	2.3299898957	0.6387072122	1.6351461417
O	0.8688076521	-1.2440765263	-0.6025988512
O	3.1559436758	-1.0390358431	-0.2069739963
O	3.0767368819	1.6340980292	-0.7086003909
O	1.2500827565	2.3815031496	1.3313556896
O	-0.8304013411	0.7171575479	0.6315809953
C	-0.7961570027	-2.0519798753	0.8839923010
H	-0.7057793475	-3.0180716774	0.3819659638
H	-1.0284004014	-2.2282917574	1.9418612230
C	3.4691691464	-2.3677899316	-0.6162856192
H	4.4277935216	-2.6198749055	-0.1626026827
H	2.7063192230	-3.0747988476	-0.2746744701
H	3.5534569931	-2.4215413170	-1.7079708550
H	2.9311114653	2.5722144869	-0.5207777938
H	1.1767736529	2.4839204798	2.2905768465
C	-2.0864760041	0.0027931927	0.6667794975
O	-2.8813822144	0.5795842134	-0.2407640955
C	-2.6659046456	0.0138455445	2.0783398660
H	-3.6260664668	-0.5085035164	2.0610293707
H	-2.0222014645	-0.4648951422	2.8202415994
H	-2.8309743512	1.0521304883	2.3770681724
O	-1.8472801236	-1.3539291510	0.2299899781
H	-1.0047091776	3.0044078839	-0.4442187219
O	-1.9313003193	3.0766091802	-0.7097179370
H	-2.2955127405	2.1742569728	-0.5278642889
H	-4.0924161370	-0.4455597135	-0.6463654003
O	-4.8399864448	-1.0533194150	-0.9121974833
H	-4.4154572294	-1.8962749455	-1.1136949351
H	-0.5117273589	-0.4477173069	-1.8557373008
O	-1.1854963072	0.0135165336	-2.3832547963
H	-1.8582415916	0.2594737977	-1.7072030579

alpha-D-Gal-TS1a
-1 1

C	-2.1470507911	0.4789410409	0.6282783775
C	-0.7323219197	1.0779729311	0.6913241700
C	0.2243365485	-0.1081167481	0.5572963312
C	-0.0723517017	-0.8161640347	-0.7612469687
C	-1.4863255327	-1.3633930788	-0.7861040238
H	-0.6080092380	1.5472128313	1.6784316210

H	-2.2324279050	-0.2046991968	1.4854170546
H	0.0241673296	-0.1195511494	-1.6027560779
H	-1.7689757337	-1.7773253816	-1.7614582671
H	-0.0135965445	-0.7996241520	1.3839167163
O	-2.3589677278	-0.2625237963	-0.5855719608
O	-1.6201060951	-2.3385472070	0.2096252967
O	0.9439424034	-1.8048936777	-0.8401959642
O	1.5872251629	0.1620098194	0.5258245750
O	-0.5464014616	2.0409171954	-0.3324309820
H	0.3746383507	2.0209070690	-0.6594295854
C	-3.2989304014	1.4541876694	0.7634504758
H	-4.2250296125	0.8761164609	0.8624532734
H	-3.1500715840	2.0321113153	1.6840252883
C	-2.9272513500	-2.9014012250	0.2677419794
H	-2.8737701852	-3.7534785198	0.9456514199
H	-3.6496028912	-2.1742254463	0.6526168509
H	-3.2439677135	-3.2406318920	-0.7255783309
O	-3.3820512386	2.3135954961	-0.3680882756
H	-4.2151085052	2.7988908242	-0.3104939479
C	2.2015348590	-1.1884549868	-0.7858867584
O	2.5818553251	-0.5053045905	-1.7555486683
C	3.1636639933	-1.9688366382	0.0621454863
H	4.0258209196	-1.3444514178	0.3014584190
H	2.6877054858	-2.3277366989	0.9749427107
H	3.5059589574	-2.8287616084	-0.5239791360
O	2.4763551246	2.6788830112	0.9248448875
H	2.4257704742	3.0278012143	0.0234489204
O	1.8400564897	2.1640943222	-1.8562071162
H	1.5168604284	2.3386699991	-2.7499324709
O	2.4765763163	-0.2731853425	2.9582014642
H	2.9679437298	-1.1036807801	2.9550013887
H	2.1441576050	1.7540022774	0.8167762312
H	2.0852156790	1.2142652750	-1.8448229149
H	2.1712109502	-0.1385711838	2.0143855108

alpha-D-Gal-TS1b
-1 1

C	-2.4601218045	0.1335337687	-0.4637107400
C	-1.7670539933	-1.2347458787	-0.6313238981
C	-0.2971099092	-0.9108474083	-0.7985807989
C	0.2352203457	-0.1230836225	0.3850895060
C	-0.5250134187	1.1914157248	0.5116509538
H	-2.1683650557	-1.7074696345	-1.5381482920
H	-2.3074838174	0.6917435984	-1.3979389849
H	0.0210045652	-0.6821504268	1.3088257633
H	-0.2800754221	1.7323811177	1.4333146438
H	-0.1818263598	-0.3203130331	-1.7179472319
O	-1.9090111009	0.8780301099	0.6312547728
O	-0.2637939001	1.9897573871	-0.6044860255
O	1.6125719644	-0.0667169027	0.1495463449
O	0.5631040178	-2.0357706390	-0.8319349671
O	-1.9291332022	-2.0945500183	0.4862869914
H	-2.8424917644	-1.9970163882	0.8049920659
C	-3.9561407977	0.0471300896	-0.2480235454
H	-4.3674522528	1.0599427318	-0.1758045329
H	-4.4021251580	-0.4567372376	-1.1124192431
C	-0.8317049271	3.2930057565	-0.5114550119

H	-0.4773910761	3.8550011825	-1.3766480662
H	-1.9254757931	3.2476455954	-0.5299882336
H	-0.5003902250	3.7869530688	0.4096837171
O	-4.2255199729	-0.6815431174	0.9504283675
H	-5.1732817655	-0.8641594716	0.9965006577
C	1.9250272506	-1.6424732819	-0.6867368880
O	2.6015901364	-2.3849229721	0.0657555455
C	2.5313407014	-1.1387393796	-1.9779492059
H	2.7580563727	-2.0127273157	-2.5967611959
H	1.8606221586	-0.4778413600	-2.5291861275
H	3.4630264703	-0.6120064889	-1.7608437542
O	2.4892133701	2.1421946222	1.7761801782
H	2.2602990604	1.4481336099	1.1309245187
H	2.6160131804	1.6569517914	2.6028495500
O	2.5229571563	2.0495425467	-1.4888590556
H	2.1662945057	1.2814660058	-0.9947576686
H	1.8211689967	2.7091313349	-1.4099134242
O	2.6086658610	-0.7784339560	2.6345457991
H	2.8434170393	-1.7149092600	2.6534892900
H	2.3228465632	-0.6149832492	1.7087672255

alpha-D-Gal-TS2a
-1 1

C	0.9469231039	1.1301402225	-0.5389791707
C	-0.1960189624	0.1685179087	-0.8711462013
C	0.2839668067	-1.2875567694	-0.8399295270
C	1.1003260351	-1.5894123798	0.4087019260
C	2.2191466820	-0.5567581041	0.5590367549
H	-0.5037625308	0.3993639733	-1.9070322397
H	1.6456678721	1.1468198432	-1.3875020469
H	0.4769370267	-1.4940363232	1.3023498640
H	2.7621026321	-0.7016557448	1.5024451439
H	0.8846952583	-1.5368730220	-1.7202938352
O	1.6609300265	0.7368970571	0.6412267084
O	3.0977321147	-0.6871400432	-0.5267219245
O	1.6073779270	-2.9084692731	0.2977180305
O	-0.8914833132	-2.0955860571	-0.9293431148
C	0.4326462108	2.5294310619	-0.2972268412
H	-0.2612810726	2.7982839096	-1.1046614879
H	-0.1073488081	2.5604338229	0.6559078198
C	4.2166832447	0.1899190561	-0.4443447657
H	3.9076261389	1.2340673134	-0.5606075771
H	4.7282099469	0.0677846486	0.5178926675
H	4.8924120183	-0.0802594661	-1.2561031275
O	1.5455761427	3.4188828841	-0.2688788167
H	1.2352093146	4.2879590791	0.0170498707
C	-2.0823154011	-1.6219364464	-0.3633111452
O	-3.0264574827	-1.4636406283	-1.1402743059
C	-2.3230149474	-1.9741582585	1.0802823784
H	-1.4141300594	-1.9679400837	1.6780718336
H	-3.0504598602	-1.2773952836	1.5018797255
H	-2.7518473449	-2.9821244098	1.1003216903
O	-1.2806511291	0.2335022397	-0.0020529128
O	-0.6655612513	0.4896526534	2.6577763766
H	-0.8224078416	0.3546834448	1.6892985167
H	-2.5064825249	0.9068009815	-1.1567710672
O	-3.0552371514	1.4216232199	-1.7897296488

H	-2.2160042718	1.6010222095	0.7326828375
O	-2.7464248506	2.2457664397	1.2468469747
H	-1.4440340549	0.1123092360	3.0876591248
H	-2.8099871711	2.3423627863	-1.6293977742
H	-2.3165098049	2.2507138589	2.1127790504
H	1.9380983326	-3.1878005572	1.1627932364

alpha-D-Gal-TS2b
-1 1

C	-1.9602177011	0.7368022367	0.5796193470
C	-0.4476616033	0.8914876600	0.6374908285
C	0.3230317591	-0.4181492330	0.4312556240
C	-0.2767872966	-1.1951154995	-0.7449346679
C	-1.7860463917	-1.3297175020	-0.6022992214
H	-0.1820291260	1.3351738521	1.6057781118
H	-2.2555527146	0.2303646450	1.5092903596
H	-0.1019485984	-0.6230462612	-1.6656473259
H	-2.2252196476	-1.8089901017	-1.4861233511
H	0.2642019693	-1.0361180243	1.3405431639
O	-2.3764860451	-0.0450914348	-0.5425398417
O	-2.0625118264	-2.0876173231	0.5447573213
O	0.2889047999	-2.4899318973	-0.8681714850
O	0.0161751838	1.7215967907	-0.4285914769
C	-2.7175415643	2.0549570548	0.5243179333
H	-3.7868940415	1.8545292389	0.6260429060
H	-2.3988725858	2.6701874795	1.3753236962
C	-3.4478333696	-2.3840952316	0.6953096000
H	-4.0264066049	-1.4746772788	0.8879915513
H	-3.8313314446	-2.8767860594	-0.2058138038
H	-3.5372091107	-3.0584839017	1.5471513950
O	-2.5554478682	2.7550277691	-0.7015923984
H	-1.6051551142	2.8601566988	-0.8615783500
H	1.1480094866	-2.3776908250	-1.3140479802
C	1.4129945848	1.7329719079	-0.5825588381
O	1.8204555253	1.7195005189	-1.7572794603
C	2.1568228224	2.5088842345	0.4776116495
H	3.1965282412	2.1776912715	0.5039159973
H	2.1317723664	3.5641867404	0.1867898001
H	1.7152060899	2.4045313447	1.4687379282
O	1.6391494008	-0.0178214837	0.1706957762
H	3.4957156827	-2.1292467218	1.6650093548
O	3.1678907100	-2.3021382948	0.7731464989
H	2.6606864252	-1.4995503196	0.5336638639
O	2.5319235933	0.2227683984	2.7526221878
H	2.3368555787	-0.6558745105	3.1025834404
H	2.2488898265	0.1901484861	1.8078645244
H	2.3120191689	-0.6467266423	-1.3687783759
O	2.5662393296	-1.2536224977	-2.1015961984
H	2.2408771099	-0.8232712837	-2.9034600846

alpha-D-Gal-TS3a
-1 1

C	0.2812525683	0.4494585816	-0.9896917673
C	0.4432478012	-1.0464497782	-0.7228342976
C	1.9069104789	-1.3694737185	-0.4215971979
C	2.4258026603	-0.4800870440	0.6994452394
C	2.1896513957	0.9876197776	0.3553124729

H	0.1344995387	-1.6136427882	-1.6086151726
H	0.8220470294	0.7018212552	-1.9117950567
H	1.8641383305	-0.6939184449	1.6182014525
H	2.4364971203	1.6347333948	1.2069012820
H	2.4882993671	-1.1701318650	-1.3305143297
O	0.8201850885	1.2114214581	0.1012654906
O	2.9886011912	1.3182722756	-0.7486565044
O	3.8128917180	-0.6676854832	0.9154258768
O	2.0914094498	-2.7131893658	-0.0075788674
O	-0.3214914438	-1.4419537279	0.4173037075
C	-1.1785996512	0.8496410457	-1.1481868515
H	-1.2275470870	1.9485240765	-1.2025838821
H	-1.5203061218	0.4660719301	-2.1241496192
C	2.8834375997	2.6901398146	-1.1184331129
H	3.6655318544	2.8806294485	-1.8535860088
H	1.9055006685	2.9037493089	-1.5619343375
H	3.0351579952	3.3342724908	-0.2444294419
H	3.9498549699	-1.5906579270	1.1729070778
H	1.9793088121	-3.2934404903	-0.7731815192
C	-1.7053810267	-1.4938646097	0.3205129944
O	-2.2885512218	-1.6383032368	1.4085014623
C	-2.2721887719	-2.1107523006	-0.9385567494
H	-1.9951589043	-1.5958843097	-1.8581432078
H	-1.9108926528	-3.1437517676	-0.9943565938
H	-3.3585860628	-2.1229623258	-0.8475316221
O	-1.9857633961	0.3733747977	-0.1015135293
H	-1.2986589204	0.7012932404	1.5770991866
O	-0.8998764731	0.9989703407	2.4244770856
H	-0.0051037938	1.2465505409	2.1537238096
H	-2.7770819036	1.7934329835	0.5692584229
O	-3.1998124576	2.6233756612	0.8885006438
H	-2.7049939721	2.8545828274	1.6849462372
H	-3.5244240851	0.3658024281	-0.7808317913
O	-4.4177753594	0.4585685673	-1.2075592945
H	-4.4775943325	-0.2713520620	-1.8362816871

alpha-D-Gal-TS3b
-1 1

C	-0.5217645923	-1.2700846426	-0.7032267943
C	0.1896497003	0.0764301121	-0.9179997849
C	-0.8685416293	1.1792168409	-0.9486259944
C	-1.7048590365	1.1453009603	0.3246190988
C	-2.2932992205	-0.2510163904	0.5371966510
H	0.6653682532	0.0396532657	-1.9138073333
H	-1.1903051515	-1.4755483109	-1.5504587367
H	-1.0697540938	1.3857057646	1.1817412255
H	-2.7627540445	-0.3375194803	1.5256194733
H	-1.5287030265	0.9974848542	-1.8099024465
O	-1.2909859196	-1.2417243792	0.5099294783
O	-3.2542003151	-0.4469448625	-0.4691335940
O	-2.7289316830	2.1274891394	0.2965038822
O	-0.2254687985	2.4375661466	-1.0984200111
O	1.1510212935	0.3015062798	0.0742544724
C	0.4581972478	-2.4218650645	-0.5870859749
H	-0.0511950195	-3.3134144287	-0.2162965151
H	0.8895621798	-2.6367874661	-1.5684145985
C	-3.9704649502	-1.6704714001	-0.3360734347

H	-4.7690101942	-1.6518333551	-1.0779474774
H	-3.3166256300	-2.5280823556	-0.5243175900
H	-4.4010390466	-1.7542566274	0.6687619969
O	1.4941890337	-2.1369614014	0.3565285272
H	-3.3705770301	1.8677323928	-0.3827614799
H	-0.9063755128	3.1243988049	-1.0452228633
C	2.3914136680	-1.1588199625	-0.0640038448
O	2.7848129510	-1.1654699957	-1.2484403799
C	3.2925794241	-0.7376324275	1.0640241425
H	4.0517792530	-1.5149204966	1.1992913927
H	3.7902546806	0.1941691495	0.7893484574
H	2.7432499523	-0.6082790902	1.9963735896
O	2.9938454551	1.6942466722	-1.4550772724
H	3.3617146065	0.8487852506	-1.7550212031
H	2.2934592397	1.3825594123	-0.8404689300
O	0.3474092642	-0.2288871805	2.7041384222
H	0.6025564990	-0.0770885696	1.7649559695
H	-0.4113843089	-0.8227187351	2.6297604021
O	1.3741180460	2.6747230567	1.4289748114
H	0.8608480915	3.2755546268	0.8719105097
H	1.2772443639	1.8128618931	0.9628447568

beta-D-Gal-2Ac
-1 1

C	-1.4520254908	1.5754910929	-0.3913735031
C	-1.4065770402	0.0486256726	-0.3868939635
C	-0.0622918410	-0.3984415603	-0.9730958049
C	1.0604536668	0.2384755433	-0.1557228483
C	0.9073060922	1.7550682810	-0.1597772890
H	-2.2133036441	-0.3345336566	-1.0258023288
H	-1.3810291438	1.9257207488	-1.4314341931
H	1.0646218106	-0.1260574262	0.8748883208
H	0.9936425989	2.1515723525	-1.1861899415
H	-0.0019855353	0.0195163330	-1.9896275763
O	-0.3621199310	2.0994502329	0.3686370449
O	1.8846356997	2.3063202473	0.6610542615
O	2.3158683016	-0.0544005945	-0.7836696531
O	0.1051924869	-1.7942622553	-1.0628029881
O	-1.5840940476	-0.3785990044	0.9552923229
H	-1.6763403293	-1.3489016512	0.9618136025
C	-2.7080534895	2.1335813834	0.2548600733
H	-2.7095242699	1.8844130070	1.3212932560
H	-2.7120238170	3.2235281333	0.1453527916
C	2.0165740756	3.7204302082	0.5116847617
H	2.8478052283	4.0249984850	1.1473390084
H	2.2389755856	3.9715135593	-0.5312499257
H	1.1055446514	4.2347773876	0.8300255564
O	-3.8273108185	1.5535844861	-0.4029489492
H	-4.6318266796	1.9249683955	-0.0178887726
C	3.0904547656	-1.0217519311	-0.2724371449
O	2.7988707658	-1.6604993616	0.7236622785
C	4.3349487618	-1.2167993170	-1.0786872915
H	4.0595789413	-1.6571911993	-2.0414002163
H	4.8137696337	-0.2547756419	-1.2706336026
H	5.0129108382	-1.8849932692	-0.5507146367
O	-1.6134996986	-3.0255660665	0.3310059610
H	-1.6487076207	-3.9654460585	0.1134234125
O	0.4781035206	-2.8466227970	2.0515745993

H	-0.2970780447	-2.9436107409	1.4422093845
O	-4.0827655743	-2.1428273388	-0.1234718677
H	-4.0368019352	-1.2012215127	0.0896891888
H	-0.6038153110	-2.3094886429	-0.5195125533
H	1.1515456408	-2.3735509849	1.5377918893
H	-3.1590995833	-2.4918583191	0.0372493560

beta-D-Gal-2Ac-H
0 1

C	-2.0947550332	-0.4929183313	0.3514375806
C	-1.3891085620	0.8633114104	0.2987931529
C	0.0693744741	0.6557909960	0.7170945222
C	0.6983297112	-0.4089028255	-0.1751623244
C	-0.1155171602	-1.6967950264	-0.1241367641
H	-1.8709089715	1.5344250906	1.0221091805
H	-2.0112754218	-0.8545085471	1.3882397625
H	0.7516561930	-0.0617537096	-1.2095816239
H	-0.1222810712	-2.1152433880	0.8973310499
H	0.0888333281	0.3069612168	1.7589156848
O	-1.4466487629	-1.4197012883	-0.5225587377
O	0.4404809635	-2.6026785038	-1.0182668933
O	2.0135259432	-0.6865122313	0.3233070021
O	0.7766702697	1.8802228877	0.6133457751
O	-1.4668554916	1.4030851236	-1.0088955327
H	-1.2389501951	2.3510340042	-0.9575591296
C	-3.5760463969	-0.4930934896	0.0257100496
H	-3.9783397057	-1.4789056560	0.2822002291
H	-4.0655128461	0.2571053347	0.6592203181
C	-0.0580098898	-3.9320540256	-0.8592014338
H	0.5110649900	-4.5630733041	-1.5415340566
H	0.0941752606	-4.2716140638	0.1712224820
H	-1.1200720580	-3.9841092531	-1.1141649792
O	-3.8019408244	-0.2222303922	-1.3520135974
H	-4.7087329329	-0.4812188902	-1.5602131132
C	3.0484444261	-0.7050438404	-0.5335129118
O	2.9352805820	-0.4685705061	-1.7230107438
C	4.3420078795	-1.0131525809	0.1514772064
H	4.8040187323	-0.0642485808	0.4428110323
H	4.1813737036	-1.6153862614	1.0456619621
H	5.0060764486	-1.5220220067	-0.5468630366
O	2.9636463811	1.4636140725	2.1553019648
H	2.9446699703	0.4969516274	2.2084752424
H	1.5898825340	1.8148946113	1.1675698508
H	2.8643819751	1.7732205906	3.0659806724
O	1.8651839625	2.3190688848	-1.9905053878
H	2.2447126440	1.4413992311	-2.1482520653
O	-0.6416342987	4.0542963222	-0.4517495564
H	-1.2791192273	4.5157375974	0.1085405066
H	1.4309939187	2.2310161136	-1.1215276430
H	-0.0529665624	3.5753985670	0.1577217636

beta-D-Gal-3Ac-a
-1 1

C	2.2567821887	-0.7998901179	0.7078689623
C	1.2102371225	-1.7358096146	0.0866110039
C	-0.1448946326	-1.0643046620	0.2501034279
C	-0.1827374732	0.3445885677	-0.3530593932

C	0.9744759264	1.1394859012	0.2604743684
H	1.2186873198	-2.6844634812	0.6411745853
H	2.0058105042	-0.6821193298	1.7738511261
H	0.0237400798	0.2634901250	-1.4351244358
H	0.8141941790	1.2733363139	1.3449180868
H	-0.3684017838	-1.0007176866	1.3193285784
O	2.2186257548	0.4709919428	0.0654533829
O	1.0590202079	2.3739403567	-0.3735560469
O	-1.4126703935	0.9455624360	-0.1121186111
O	-1.1350995415	-1.8590614269	-0.4216827612
O	1.4278908765	-1.9687094706	-1.2972197000
H	2.3853096966	-2.0750128548	-1.4303462836
C	3.6754017882	-1.3261055652	0.6483070138
H	4.3457409247	-0.6050911502	1.1285929137
H	3.7170061569	-2.2720926116	1.1987508823
C	1.7148337506	3.3731129485	0.4049427182
H	1.7668928001	4.2719729163	-0.2105681383
H	1.1377410942	3.5838378147	1.3131153055
H	2.7282307566	3.0602881496	0.6753639254
O	4.0561145384	-1.5227194070	-0.7139513246
H	4.8886073798	-2.0128825697	-0.7356078420
C	-2.3588881602	-2.0651142327	0.0857872399
O	-3.1976832858	-2.5294786631	-0.6715703192
C	-2.6324232754	-1.7698192706	1.5297498321
H	-1.9107273551	-2.2882104130	2.1667323931
H	-2.5519901408	-0.6972982336	1.7238387841
H	-3.6406630833	-2.1088913540	1.7602428318
O	-1.6607704161	3.4280400779	-0.9062454193
H	-1.5253080049	2.4749278274	-0.6160324385
H	-0.7626261326	3.7751907825	-0.9775668829
O	-1.6804152680	1.7313817295	2.3482863232
H	-1.5716093952	1.4113649568	1.3962365412
H	-1.0119074742	2.4200990534	2.4562691535
O	-2.9820943449	0.2789014502	-2.0815230075
H	-3.1688470802	-0.6645130953	-1.9838562260
H	-2.3909129043	0.5162792402	-1.3010725501

beta-D-Gal-3Ac-b
-1 1

C	1.1654012795	0.2005540215	-1.2807460291
C	-0.1507561518	0.6597166693	-0.6272765071
C	-0.9603928848	-0.6256080666	-0.3971800372
C	-0.1927920254	-1.6056314741	0.4802524176
C	1.1636079952	-1.8911967430	-0.1560074191
H	-0.6852969360	1.2663086831	-1.3821368105
H	0.9015022014	-0.3429029982	-2.2038889664
H	-0.0243009845	-1.1644594059	1.4705679086
H	1.0297086372	-2.4364929932	-1.1073754331
H	-1.1911335888	-1.1165007848	-1.3476094424
O	1.8645013080	-0.6865999711	-0.4034978788
O	1.9142289395	-2.6500145761	0.7376597221
O	-0.8772846473	-2.8419911893	0.5903937148
O	-2.1772232525	-0.3359006699	0.3145374076
C	2.1376595125	1.2943256720	-1.7022999841
H	2.9178309332	0.8320122340	-2.3142184835
H	1.5951165079	2.0130029709	-2.3303340278
C	3.0774235424	-3.2326645824	0.1498338513

H	3.5451253313	-3.8443923093	0.9212625558
H	2.7966327789	-3.8649815146	-0.6996980324
H	3.7800206150	-2.4619849542	-0.1794330830
O	2.7973179518	1.9513237754	-0.6329500615
H	2.1988042843	2.6441326316	-0.2887902712
H	-1.6768750120	-2.6997841329	1.1165049099
C	-3.3246296414	-0.0391865676	-0.3112770578
O	-4.2486336150	0.3451471614	0.3881347352
C	-3.4264943205	-0.2076127404	-1.7962270585
H	-2.6733453808	0.3957621568	-2.3091650421
H	-3.2657032913	-1.2559852780	-2.0635650341
H	-4.4217986576	0.1026867426	-2.1081652013
O	0.0036298652	1.3666372548	0.5638086545
O	-2.2679400187	2.5749195028	1.2030288607
H	-2.9796592527	1.9269628427	1.2954017105
H	-1.4485242915	2.0478102759	0.9814225497
H	1.1361802897	0.8722982904	1.6108845116
O	1.8298814878	0.5994744965	2.2838484895
H	2.3382863786	-0.0717818953	1.8088999843
H	0.4056035451	2.7672417418	0.3061553520
O	0.7930222794	3.7135471887	0.1428005881
H	0.3745971588	4.0392780345	-0.6644344530

beta-D-Gal-3Ac-H
0 1

C	2.2258709761	-0.6161889653	0.3352162492
C	1.0329415081	-1.5057594781	-0.0095718352
C	-0.2283544673	-0.6444629237	0.0614696323
C	-0.1131305970	0.5972720722	-0.8183303159
C	1.1699447473	1.3480882679	-0.4577151176
H	0.9605539647	-2.3030350141	0.7412871725
H	2.0518109854	-0.2421014757	1.3564346475
H	-0.0483081803	0.2994540526	-1.8703118734
H	1.1136442811	1.7375367075	0.5751832921
H	-0.4065225600	-0.3526249053	1.1008509076
O	2.2918623558	0.4853087899	-0.5672592808
O	1.3341737130	2.3894789827	-1.3632625870
O	-1.3248540473	-1.4429905799	-0.4090019296
O	1.1934224919	-2.0562609597	-1.3061826562
H	0.4004737497	-2.5728383237	-1.5113530803
C	3.5805901185	-1.2965984362	0.3639899893
H	4.3026712545	-0.5964556452	0.7981562306
H	3.5071148837	-2.1727615089	1.0198745141
C	2.3448096918	3.3229649931	-0.9818946671
H	2.3189877561	4.1276646995	-1.7165453517
H	2.1323883521	3.7273214717	0.0140827643
H	3.3333998634	2.8554280497	-0.9874764570
O	3.9922180115	-1.6699972732	-0.9447971651
H	4.9121869321	-1.9608568014	-0.8953050515
C	-2.4361980419	-1.5553236864	0.3397056442
O	-2.5975431663	-0.9568542770	1.3872302648
C	-3.4052379133	-2.5343193732	-0.2419817546
H	-4.4128429900	-2.2903339061	0.0930010160
H	-3.3486255543	-2.5439910703	-1.3301651523
H	-3.1375879701	-3.5283185196	0.1311763689
O	-1.2428729581	1.4377001771	-0.6949589951
H	-1.4268009863	1.6305664631	0.2549873156

H	-4.4427523625	1.1857986240	0.0979298745
H	-2.8092413334	0.7569945983	-1.2590459020
O	-2.0840877025	1.8478859710	1.8999666214
O	-4.6820326805	1.5875030786	0.9612208974
O	-3.7265446581	0.4271525354	-1.3672835809
H	-1.9703350498	0.9220655524	2.1656103510
H	-3.0281991802	1.8865609762	1.6322466967
H	-5.1689446425	2.3942209051	0.7487628940
H	-4.0649907752	0.8556644151	-2.1646159307

beta-D-Gal-4Ac-a
-1 1

C	-1.7078135262	0.7031936801	0.5047916416
C	-0.1864531672	0.7736816315	0.4535995286
C	0.4363780489	-0.6245322085	0.3168740550
C	-0.2384698499	-1.3558485531	-0.8510100043
C	-1.7561347177	-1.3508953357	-0.7080761218
H	0.1829305808	1.2653780141	1.3571588250
H	-1.9637097977	0.1839908123	1.4393907205
H	-0.0074339155	-0.8216619789	-1.7835349411
H	-2.2397148857	-1.7628509291	-1.6027092547
H	0.1710365441	-1.1639423948	1.2448797704
O	-2.2315484694	-0.0228717786	-0.6094232811
O	-2.1037736217	-2.1143428803	0.4162952412
O	0.1941962326	-2.7031612309	-0.9490729054
O	0.1990148299	1.5610847310	-0.6963742842
C	-2.4109625104	2.0537944878	0.5381628178
H	-3.4620328367	1.8962594357	0.7912301083
H	-1.9518237600	2.6621349448	1.3277104556
C	-3.5115731257	-2.2391672085	0.5910561161
H	-3.9570701969	-1.2851430874	0.8916458844
H	-3.9836024200	-2.5849050306	-0.3362748746
H	-3.6686601037	-2.9766734463	1.3783852957
O	-2.4080573740	2.7424720322	-0.7054007613
H	-1.4927602156	2.8386436188	-1.0034677119
H	1.0894303118	-2.6811205054	-1.3344651097
C	1.2216756977	2.4243267323	-0.5833016511
O	1.7762657830	2.6764945005	0.4703818110
C	1.5633266931	3.0369330302	-1.9050252226
H	0.6606336045	3.4450874654	-2.3665225344
H	2.3086518878	3.8184954520	-1.7693654030
H	1.9558090046	2.2573286145	-2.5641851166
O	1.8204835911	-0.5652258780	0.1559341200
H	3.2041545329	-2.3405208153	2.1749814224
O	2.7343069720	-2.6862094746	1.4058776113
H	2.3970092505	-1.8768117408	0.9180351012
O	2.7704540802	0.5937989650	2.3016589696
H	2.4262345594	0.1569853439	1.4646341496
H	2.5452038331	1.5249867989	2.1722652100
H	2.3842803317	-1.1996191526	-1.1476812582
O	2.6946276922	-1.7539174445	-1.9408364387
H	2.4388231722	-1.2560340069	-2.7279203701

beta-D-Gal-4Ac-b
-1 1

C	0.1596802519	0.1779459111	-1.3561177758
C	0.2248342904	-1.2719275469	-0.8713322265

C	1.6736267427	-1.6840342873	-0.6517768145
C	2.3883226129	-0.6877654914	0.2514366491
C	2.2473220256	0.7095888597	-0.3424346743
H	-0.2352827749	-1.9255775288	-1.6158647747
H	0.6393568622	0.1881526335	-2.3481095722
H	1.9305451217	-0.6888542815	1.2507791626
H	2.7366077437	0.7631461083	-1.3312499177
H	2.1679876648	-1.6704717332	-1.6340598346
O	0.8787743676	1.0392192732	-0.4741883520
O	2.8320802487	1.6173466809	0.5375044371
O	3.7469464194	-1.0835539022	0.3188912751
O	1.6995766157	-2.9920742186	-0.1108205152
O	-0.4546936179	-1.4381975751	0.3862882151
C	-1.2448207113	0.7667514618	-1.5022937873
H	-1.1282812462	1.6929436239	-2.0915488465
H	-1.8260346975	0.0677324460	-2.1273236691
C	2.9488954779	2.9350211911	0.0007153758
H	3.5343131247	3.5138841522	0.7150910779
H	3.4681956876	2.9069165299	-0.9638232941
H	1.9643844759	3.3960755490	-0.1232706636
H	4.1798508622	-0.5878708192	1.0281030104
H	2.6254256402	-3.2455962097	0.0148365215
C	-1.7315440571	-1.8415223952	0.3668608624
O	-2.3201671144	-2.1449444790	-0.6567248482
C	-2.3439209933	-1.8667519699	1.7313673532
H	-1.5971952790	-2.0825399441	2.4961905620
H	-2.7644801227	-0.8746714747	1.9256443971
H	-3.1484611498	-2.6016691117	1.7532469910
O	-1.8844987780	1.0316607263	-0.2873108138
H	-1.0901187116	0.9303324370	1.2084485543
O	-0.7122777308	0.9585918300	2.1294087283
H	-0.1595689346	0.1667813295	2.1705030864
H	-1.5533215670	2.5350847016	-0.0263541825
O	-1.2900530768	3.5062750783	0.1131437680
H	-0.3602779527	3.4723750065	0.3719307563
H	-3.3158508698	0.5114176538	-0.3839974583
O	-4.2688340762	0.1497139768	-0.4732236500
H	-4.1563119336	-0.8089670819	-0.5208090630

beta-D-Gal-4Ac-H
0 1

C	-0.4067387903	-0.3263423449	-0.8039047056
C	-0.1919535998	1.1549421933	-0.4974588071
C	-1.5476010691	1.8513832949	-0.3980565575
C	-2.4556284834	1.1421992648	0.6000368794
C	-2.5572836020	-0.3322738284	0.2365046150
H	0.4109449363	1.6193374718	-1.2807464420
H	-0.9017394767	-0.3853117156	-1.7850132646
H	-2.0283903260	1.2182885178	1.6102742249
H	-3.0429095509	-0.4630146234	-0.7454456117
H	-2.0186131727	1.8025429363	-1.3889809688
O	-1.2560573062	-0.9059377144	0.1884603239
O	-3.2700322559	-0.9852862724	1.2330225843
O	-3.7627696890	1.6835518196	0.5665713210
O	-1.4320921839	3.1962707010	0.0280572173
O	0.4595736244	1.3076227662	0.7738335618
C	0.8450976478	-1.1832535137	-0.8953981916

H	0.5382293338	-2.1968737547	-1.1813451509
H	1.4907645632	-0.7810231837	-1.6813595546
C	-3.6489631844	-2.3173287536	0.8828840540
H	-4.2552151964	-2.6950758497	1.7059096484
H	-4.2407336917	-2.3121146965	-0.0388873734
H	-2.7691275210	-2.9540660281	0.7554257393
H	-3.6968424020	2.6293322405	0.7639156010
C	1.7769170171	1.5562565223	0.7922870165
O	2.4304600793	1.7609546234	-0.2152636347
C	2.3318491447	1.5190466827	2.1797562152
H	1.6251310102	1.9453019061	2.8934501473
H	2.4960524150	0.4674712744	2.4419226429
H	3.2844923038	2.0463959716	2.2108585442
H	-1.1193579546	3.7354142102	-0.7115707934
O	1.6267911287	-1.2321468258	0.2943791654
H	1.0698901524	-1.3442818181	1.0937828878
H	2.4663587913	-2.8932997630	0.1367733264
H	3.4321743132	-0.7805978587	0.1505917497
O	0.0797736336	-1.2660209581	2.6530647494
O	2.8511199001	-3.7682148027	-0.0558382952
O	4.3403834279	-0.4669634621	-0.0109976510
H	-0.6491503141	-1.0171867217	2.0585270264
H	0.3410960928	-0.4440257332	3.0908227388
H	2.5432522755	-3.9800728994	-0.9462120790
H	4.2287491491	0.4873631349	-0.1264826492

beta-D-Gal-6Ac
-1 1

C	0.3045713960	-0.2670852659	-0.5071252076
C	-1.1929200559	-0.5373731031	-0.7387100043
C	-1.9429906700	0.6234870086	-0.0688623115
C	-1.4766580664	1.9676258202	-0.6239353728
C	0.0337067668	2.0881396941	-0.4500923043
H	-1.4473825709	-1.4592426657	-0.1822887295
H	0.5116859515	-0.2487857855	0.5746282969
H	-1.7096420713	2.0319095726	-1.6923712748
H	0.3015051900	2.1069728121	0.6232510821
H	-1.7196205293	0.6053191506	1.0104042199
O	0.6750915699	0.9911859429	-1.0694923851
O	0.4741921082	3.2522610613	-1.0759415146
O	-2.1628050786	3.0419883223	0.0014192156
O	-3.3393240067	0.4604634532	-0.2655247653
O	-1.5094436559	-0.6633066091	-2.0892388457
C	1.1880339595	-1.3082990137	-1.1608696910
H	1.0241800893	-2.2834841759	-0.6996750950
H	1.0124106243	-1.3681073048	-2.2354951547
C	1.8517206883	3.5337224092	-0.8319356580
H	2.0505910758	4.5194478762	-1.2526297527
H	2.0526562537	3.5463922694	0.2456724505
H	2.4959010852	2.7932046185	-1.3168850555
O	2.5669683992	-0.9764647523	-0.9156603377
H	-1.9856909813	3.0137613148	0.9543992268
H	-3.7810712091	1.2358079654	0.1106109845
C	3.2293908318	-0.2717410706	-1.8451960168
O	2.7624304660	0.0149617373	-2.9341882236
C	4.5991986503	0.1059043944	-1.3769463171
H	5.1411000387	0.5989193972	-2.1820089677

H	4.5056186916	0.7811940958	-0.5217468462
H	5.1370766721	-0.7849944395	-1.0450811846
O	-1.1108250621	-3.1069145125	-2.8534361454
H	-0.1675933268	-3.2401271468	-3.0066554116
H	-1.2107944581	-2.1442361623	-2.5570383316
O	-0.0067384697	0.4093241591	-3.9794922488
H	-0.5675916088	0.0582347283	-3.2286334164
H	0.8972479872	0.3893434804	-3.6294127900
O	-3.9468643814	-1.5805936671	-2.2713415970
H	-4.4220343278	-1.0853391147	-1.5918485601
H	-3.0147800748	-1.2023710440	-2.2147364493

beta-D-Gal-6Ac-H

0 1

C	0.0514954232	0.0122569751	-1.6820123694
C	-0.3566264786	1.3374964864	-1.0309495612
C	-1.8786715092	1.3351617449	-0.9133307925
C	-2.3467484983	0.1333422074	-0.1029665104
C	-1.7763498212	-1.1573521983	-0.6824954479
H	-0.0412887055	2.1734524199	-1.6677180720
H	-0.4212826440	-0.0614342978	-2.6728564494
H	-1.9917008879	0.2328989157	0.9314385753
H	-2.2533091783	-1.3911722920	-1.6497472420
H	-2.2940873894	1.2572683601	-1.9273301558
O	-0.3733014566	-1.0682389933	-0.8583255213
O	-2.0089156067	-2.1760321215	0.2406418604
O	-3.7588804293	0.0176976561	-0.1297572059
O	-2.3907269207	2.4886993533	-0.2668392520
C	1.5495378909	-0.1010852162	-1.8239915909
H	1.9417750036	0.7523133536	-2.3848450307
H	2.0289734134	-0.1499792068	-0.8412280872
C	-1.6929686791	-3.4787223333	-0.2535276778
H	-2.0529304761	-4.1901081584	0.4895930898
H	-2.2011423893	-3.6554811398	-1.2077850658
H	-0.6137516688	-3.5973179555	-0.3822148614
O	1.8237967739	-1.3141920757	-2.5408815415
H	-4.1273675659	0.8355147692	0.2348921764
H	-2.3254505360	3.2426003065	-0.8693008397
C	3.1175442241	-1.6003280603	-2.7470246877
O	4.0136676320	-0.8661230351	-2.3698101792
C	3.2987980124	-2.8964701134	-3.4711220929
H	4.3579645716	-3.0732176988	-3.6495596599
H	2.8799760969	-3.7063963335	-2.8682432737
H	2.7553296947	-2.8658007939	-4.4183895658
O	0.2195670228	1.4634159116	0.2634069698
H	1.1176761219	1.8540762969	0.1726964209
H	-0.7314110531	2.0169219415	1.8097482728
H	0.2552199553	-0.1346229151	1.4023249950
O	2.6692274584	2.5582531426	-0.2432523403
O	-1.3738454858	2.3540871932	2.4601681008
O	0.1805936811	-0.8761606706	2.0307446872
H	3.3003397533	1.8823833783	-0.5284880393
H	3.0809120819	2.9812103080	0.5226427742
H	-2.1224719977	2.5908910435	1.8936196120
H	-0.4284827641	-1.4870288946	1.5868528990

beta-D-Gal-IN1

-1 1

C	-2.6250827379	-0.5066799498	-0.2526441447
C	-1.3097722226	-1.3172331989	-0.3376936553
C	-0.2613292613	-0.2890707899	-0.7018321788
C	-0.2069264608	0.8162006087	0.3301840443
C	-1.5165482657	1.5665203076	0.3022445746
H	-1.4266975970	-2.0627526383	-1.1347279375
H	-2.8343137617	-0.1328234349	-1.2659487843
H	-0.0776216413	0.4072362087	1.3414640665
H	-1.7397435952	1.9828478148	-0.6940478896
H	-0.5443568732	0.1430654070	-1.6747076757
O	-2.5154658357	0.6005304234	0.6484489603
O	-1.5293278878	2.5643917336	1.2682980592
O	0.9525302829	1.5095301781	-0.0820182003
O	1.0867876392	-0.7072833940	-0.7408816962
O	-0.9469504175	-1.9524790567	0.8797531030
H	-1.7609247438	-2.2532240144	1.3210650301
C	-3.8249815360	-1.3275481740	0.1713213533
H	-4.7168056453	-0.6920462034	0.1661677164
H	-3.9609679969	-2.1386574880	-0.5521976323
C	-2.6498986305	3.4433601032	1.1598945703
H	-2.5211459923	4.2115705293	1.9221265577
H	-2.6705991226	3.9090189139	0.1684874889
H	-3.5861693533	2.9070894037	1.3375587311
O	-3.6007160894	-1.8545775188	1.4793114195
H	-4.2974198763	-2.4936967265	1.6796010473
C	1.9200562631	0.4758470703	-0.4791282329
O	2.7801164618	0.1992980857	0.4977318423
C	2.5535910785	0.9359608465	-1.7790550772
H	3.2905707752	0.1946824458	-2.0995062575
H	1.7951578551	1.0564887222	-2.5567709143
H	3.0597028150	1.8918723956	-1.6205230981
O	5.1750504968	1.3129839170	0.3493242649
H	4.2526113675	0.9385384521	0.3998155711
H	5.1370703842	2.1559640242	0.8173322291
O	4.0769271004	-2.0799672307	-0.1225717953
H	3.4883273427	-2.7801771418	0.1865455832
H	3.5999188921	-1.2381688065	0.0918896794
O	1.5748801558	-1.5354962044	2.2393058554
H	0.7174945234	-1.7084183123	1.8139744422
H	2.0082102602	-0.8791500481	1.6424620294

beta-D-Gal-IN2

-1 1

C	1.0919496878	1.7103792332	-0.2638952519
C	-0.1335303991	1.0601128114	-0.8831589728
C	0.1028618001	-0.3270461955	-1.4812823602
C	1.0786662815	-1.1573620541	-0.6513577308
C	2.2838807744	-0.3168315998	-0.2509223881
H	-0.5407821520	1.7328669196	-1.6497738050
H	1.7230580353	2.0761752035	-1.0889630364
H	0.5823257706	-1.4786233745	0.2746285060
H	2.8293948036	0.0560718440	-1.1353479022
H	0.4558633412	-0.2776101518	-2.5170225871
O	1.8367045249	0.7869272337	0.5218833156
O	3.1160942183	-1.1041145524	0.5376539084
O	1.4665965587	-2.2775932025	-1.4273459130

O	-1.1950050152	-0.9058642366	-1.4489537836
C	0.7359452890	2.8726939492	0.6553089155
H	0.1359506281	2.5103064187	1.4929494350
H	1.6569527122	3.3163814223	1.0466881310
C	4.3901182687	-0.5120475462	0.7938573175
H	4.8822623827	-0.2497555311	-0.1491625090
H	4.2882672839	0.3803530661	1.4175082077
H	4.9822708317	-1.2593538701	1.3218085394
O	-0.0482704738	3.8475392909	-0.0194326271
H	0.4938573663	4.2759807309	-0.6973882584
H	2.0028132958	-2.8600089934	-0.8704992532
C	-2.0500076990	-0.1394810873	-0.5404618194
O	-2.9892642580	0.5120646068	-1.2376734246
C	-2.5953743860	-1.0699994653	0.5263581703
H	-1.7823264075	-1.5860594489	1.0422154455
H	-3.1758937530	-0.4925450582	1.2510192305
H	-3.2512147580	-1.8082576650	0.0574690543
O	-1.1244520224	0.7688547925	0.0931104050
O	-2.7265750658	3.2302985858	-0.9116486542
H	-1.8177833536	3.3937823295	-0.6040652102
H	-2.8024376389	2.2512459024	-0.9947989250
H	-4.5300094819	0.3090954831	-0.7527889931
O	-5.4906300790	0.2339061488	-0.4833931208
H	-5.5504239983	-0.5900225830	0.0157120503
H	-2.5048252453	0.9138190208	-2.7677736213
O	-2.2534856601	1.1999214255	-3.6902417681
H	-1.9913697578	2.1243253369	-3.5949986865

beta-D-Gal-IN3
-1 1

C	0.6870545886	-1.5834555262	0.8131218401
C	-0.2088096792	-0.4775642209	1.3664842914
C	0.6164716025	0.7676304395	1.6745464537
C	1.4495941774	1.1809806350	0.4677715489
C	2.2780299738	-0.0068563471	-0.0024957093
H	-0.6906324600	-0.8301035474	2.2897522056
H	1.3790204678	-1.9392204738	1.5884058741
H	0.7865165496	1.4881553836	-0.3538786225
H	2.9914983591	-0.3188143601	0.7799136505
H	1.3020445424	0.5243463615	2.4982920167
O	1.4226736677	-1.0974001121	-0.3132422375
O	2.9518353856	0.3522765222	-1.1640161218
O	2.3461243934	2.2249212473	0.8048665037
O	-0.1881651195	1.8838911295	2.0192335731
O	-1.1732020334	-0.1209225041	0.3913244376
C	-0.1880636726	-2.7212880369	0.3290444157
H	0.4081667784	-3.4642037268	-0.2053279255
H	-0.6571013962	-3.2051722151	1.1953108778
C	3.9674055326	-0.5766815000	-1.5436153492
H	4.4809812809	-0.1442953600	-2.4021476260
H	4.6787109325	-0.7177530587	-0.7224342326
H	3.5325118998	-1.5396259212	-1.8254224938
H	1.8224318074	2.9694764011	1.1349407748
H	-0.5411678976	1.7537942568	2.9103114300
C	-2.0139138472	-1.1738024776	-0.1339675229
O	-2.6333067112	-0.6684550829	-1.2051716241
C	-2.9708930539	-1.6734080345	0.9452419150

H	-3.6062821109	-2.4519107254	0.5145404829
H	-2.4647680339	-2.0833433420	1.8224011426
H	-3.5990437933	-0.8377400252	1.2643119894
O	-1.1617887164	-2.2470671544	-0.5925958671
H	-5.0277021298	-2.5218427582	-1.5205788655
O	-4.4167486486	-2.2896590233	-2.2304265626
H	-3.7426522121	-1.6772070626	-1.8116192823
H	-2.6554587007	1.0542873120	-1.0367161482
O	-2.6145199430	2.0359698345	-0.9193941087
H	-1.8865983548	2.1534304947	-0.2940277707
H	0.3334382504	-0.4323049492	-1.8997576804
O	-0.2742999297	-0.0944693664	-2.5792035804
H	-1.1610469263	-0.2654929956	-2.1864846521

beta-D-Gal-TS1a
-1 1

C	-1.9303432772	-0.9789018588	-0.8887652880
C	-0.5025900285	-1.3979086076	-0.4971248920
C	0.3649719721	-0.1571257361	-0.7097622447
C	-0.1848212604	0.9857730829	0.1413559767
C	-1.5846470640	1.3296959772	-0.3107682369
H	-0.1846509989	-2.1943608828	-1.1858984648
H	-1.8902345074	-0.7029026513	-1.9538321090
H	-0.2202233534	0.7150509255	1.2045977564
H	-1.6104424554	1.6253031773	-1.3731722934
H	0.2400282225	0.1307870977	-1.7708237325
O	-2.3699415047	0.1532345148	-0.1285863045
O	-2.1159964170	2.3353275301	0.4898947902
O	0.7604322399	2.0246121504	-0.0681131678
O	1.7114665341	-0.2434105621	-0.3856806596
O	-0.4627562619	-1.8662971851	0.8398424882
H	0.3705398396	-1.5966567049	1.2754258477
C	-2.9982435346	-2.0501659592	-0.7785543074
H	-3.9085946979	-1.6787209312	-1.2621310264
H	-2.6505481586	-2.9347906161	-1.3265436057
C	-3.3507215296	2.8592933408	-0.0011635961
H	-3.6204130568	3.6876861019	0.6537299861
H	-3.2254612199	3.2258594569	-1.0260940937
H	-4.1375869579	2.1005512096	0.0261251889
O	-3.2694077137	-2.3689807576	0.5814451213
H	-4.0676811964	-2.9122931852	0.6051205901
C	2.0368567247	1.5864098965	0.3099524311
O	2.2789850997	1.4021684364	1.5169777324
C	3.0900678100	2.0485015735	-0.6546293330
H	2.7592587054	1.9345779930	-1.6870573072
H	3.2867664354	3.1076938697	-0.4556603244
H	4.0088827255	1.4851187536	-0.4860312964
O	3.0229868833	-0.6954697752	-2.6111072288
H	3.0609846350	-1.6575716847	-2.6802005450
O	2.7581841036	-2.5889467215	0.4206657964
H	2.5478615080	-2.5791736952	1.3651907739
O	1.5932309097	-1.0635764149	2.6005797399
H	1.0883538339	-0.8782191297	3.4035559066
H	2.5361065950	-0.5142826384	-1.7545080098
H	2.3702317120	-1.7362785143	0.1026453120
H	1.8102347046	-0.1861108768	2.2193316290

beta-D-Gal-TS1b
-1 1
beta-D-Gal-TS2a
-1 1

C	-0.5532790488	-1.3443952377	-0.9240060165
C	0.4515939042	-0.1867520821	-0.9587917015
C	-0.2859931336	1.1483224418	-1.1050407940
C	-1.4918204345	1.2806194450	-0.1760202970
C	-2.3781730315	0.0563937212	-0.3463742408
H	1.0657331652	-0.3306594449	-1.8647408905
H	-0.9684644082	-1.4617980329	-1.9383049133
H	-1.1817911018	1.3054400581	0.8743471123
H	-2.7427605474	-0.0332876302	-1.3850229660
H	-0.6404606614	1.2995289521	-2.1310247470
O	-1.6258285775	-1.0974467619	-0.0136967989
O	-3.4480993098	0.1465015106	0.5386755465
O	-2.2557217667	2.4315814391	-0.4967523081
O	0.6827951221	2.1700394910	-0.8687522759
C	0.0705326758	-2.6556180211	-0.4667638233
H	0.3984088293	-2.5529953442	0.5712223427
H	-0.6811936877	-3.4513589654	-0.5176905298
C	-4.4553191903	-0.8384594130	0.3067900632
H	-4.8205148680	-0.7710530194	-0.7240657512
H	-4.0710774952	-1.8445111239	0.4959927370
H	-5.2701319557	-0.6231663708	0.9978699609
O	1.2204846449	-2.9894528367	-1.2282635732
H	0.9455413291	-3.1900717952	-2.1337039500
H	-1.6954195129	3.2106325181	-0.3692683981
C	1.7228357952	1.9069610840	0.0379946674
O	2.8664709727	2.0014359501	-0.4193617142
C	1.4503744871	2.2287432420	1.4837176702
H	0.4247242257	2.0231896564	1.7816677537
H	2.1382410238	1.6576197411	2.1105660283
H	1.6535756852	3.2960184391	1.6236113044
O	1.2557872725	-0.0581584241	0.1695711586
O	-0.1742571610	-0.5744913538	2.4698870331
H	-0.9938117797	-0.9244741482	2.0908616091
H	0.3301846177	-0.3088992503	1.6625443484
H	2.8049725577	-0.4935573361	-0.5098212781
O	3.6739782175	-0.6893691665	-0.9352487133
H	4.0291127420	0.1949676184	-1.0986742841
H	2.1534239386	-1.2559552306	1.2430870070
O	2.5477645091	-1.7800351964	1.9692458631
H	1.8314759567	-1.8301401226	2.6177367591

beta-D-Gal-TS2b
-1 1

C	-1.6469151031	1.1466114072	0.9151196938
C	-0.1496186981	1.0962476369	0.6568257275
C	0.4305245412	-0.3247405460	0.6280793151
C	-0.4919630847	-1.2921715772	-0.1256126638
C	-1.9282800377	-1.1337981582	0.3462962815
H	0.3594421128	1.6993482475	1.4189694244
H	-1.7886867787	0.8925754095	1.9773413116
H	-0.4726365807	-1.0469290347	-1.1978437163
H	-2.0245700096	-1.3557061467	1.4239629463
H	0.5425675787	-0.6942413750	1.6609626594

O	-2.3452417258	0.2032139409	0.1083367569
O	-2.7479753284	-1.9794020055	-0.3954725817
O	-0.0911273516	-2.6374692971	0.0785078617
O	0.1717380078	1.5966158511	-0.6440520334
C	-2.2682320833	2.5161781994	0.6883803552
H	-3.3097823156	2.4938662063	1.0168909074
H	-1.7259405457	3.2450825890	1.3035911321
C	-4.0666792782	-2.1028488226	0.1354902040
H	-4.0264459901	-2.4359106825	1.1786250915
H	-4.6076521693	-1.1544290283	0.0718041864
H	-4.5781008550	-2.8532604324	-0.4674887545
O	-2.2860911966	2.9130811274	-0.6761635881
H	-1.3773815028	2.8675323489	-1.0104678111
H	0.6872713847	-2.7906547927	-0.4879448243
C	1.5525534698	1.4990771428	-0.8839663747
O	2.3236091779	2.2410968417	-0.2484649827
C	1.8294850314	1.1043293985	-2.3101693540
H	1.7204422923	1.9978897536	-2.9338903497
H	2.8571530515	0.7464389634	-2.3948697950
H	1.1346440025	0.3391875633	-2.6589043037
O	1.6728598634	-0.2035417497	-0.0081686351
H	3.9570650387	-1.9116936171	1.5205765051
O	3.0943079292	-2.2474327715	1.2458794106
H	2.6614079162	-1.4962102414	0.7874930932
O	3.1254673392	0.8224009260	2.2028628732
H	3.1206318636	-0.0179605813	2.6795750549
H	2.6765866395	0.6248878492	1.3544949158
H	1.9567224816	-1.3812610195	-1.2264496348
O	2.0391644588	-2.2039268676	-1.7732897214
H	1.5736204541	-2.0119696552	-2.5980415828

beta-D-Gal-TS3a
-1 1

C	0.0560512277	0.3468858850	-1.0574750025
C	0.3380664523	-1.1359653690	-0.8142339973
C	1.8280970138	-1.3999477154	-0.9667232889
C	2.6428928545	-0.4745617854	-0.0663001418
C	2.2430781496	0.9755102735	-0.3287630261
H	-0.2059367083	-1.7471593132	-1.5425290957
H	0.3209823663	0.5896303121	-2.0973032370
H	2.4438680939	-0.7035438684	0.9858284553
H	2.5270174130	1.2798565572	-1.3533357501
H	2.0921029639	-1.1792071307	-2.0129303739
O	0.8459973273	1.1358752237	-0.1655895693
O	2.8781095882	1.7822076547	0.6116416644
O	4.0313700057	-0.6787436775	-0.2649067215
O	2.0910909731	-2.7610312425	-0.6782738817
O	-0.0191248079	-1.4982933497	0.5206719136
C	-1.4053480175	0.6988811199	-0.8138983511
H	-1.5097848220	1.7923769754	-0.8952622869
H	-1.9926721138	0.2552932348	-1.6361748348
C	2.7339754816	3.1787729623	0.3561435182
H	3.3688014577	3.6949797967	1.0762610784
H	3.0644217826	3.4150509628	-0.6617103536
H	1.6965184695	3.4984863792	0.4896711684
H	4.2469995723	-0.4929020801	-1.1918593843
H	3.0496395408	-2.8931577457	-0.7211869290

C	-1.3583269694	-1.6036352852	0.8608631520
O	-1.5731071800	-1.6859638051	2.0795007906
C	-2.2607787729	-2.3009437415	-0.1308414514
H	-3.2632257690	-2.3404362031	0.2961800170
H	-2.3067335886	-1.8248018185	-1.1097441595
H	-1.8907406196	-3.3248033565	-0.2563218231
O	-1.8553202329	0.2483373120	0.4367285342
H	0.3658384516	1.5829838395	1.9347656991
O	-0.4872638454	1.4573690885	2.3732711825
H	-1.0238893208	0.9929387497	1.6716868836
H	-4.7062294037	-0.5203329762	-0.0378027824
O	-4.3950404325	0.3363181272	0.2813026669
H	-3.3795330718	0.2551218833	0.3756065447
H	-3.5662204144	2.4461887589	-1.8933350472
O	-4.4834974902	2.1682436193	-1.7774627935
H	-4.4592326045	1.5082977481	-1.0490679865

beta-D-Gal-TS3b
-1 1

C	0.2275249318	0.9934603996	-1.4115533459
C	-0.4882119941	-0.3307135807	-1.1178419629
C	0.5628978629	-1.4417121226	-1.1530339548
C	1.6892408830	-1.1557977026	-0.1691162980
C	2.2685619001	0.2366645795	-0.4092481846
H	-1.1887850309	-0.5189850084	-1.9521822060
H	0.6591786536	0.9707317689	-2.4234363514
H	1.3045721110	-1.2003789146	0.8541562756
H	2.8410226800	0.2605579985	-1.3544531709
H	0.9908559430	-1.4644653415	-2.1688079020
O	1.2559057557	1.2235353174	-0.4479943096
O	3.0977670463	0.5407692750	0.6713322794
O	2.7031804055	-2.1464024364	-0.2509234098
O	-0.0506983124	-2.6902199313	-0.8680183540
O	-1.1667530535	-0.2643471037	0.1056367148
C	-0.7178076380	2.1733524029	-1.3148807158
H	-0.1638753008	3.1055519312	-1.4410824824
H	-1.4789154540	2.1119024425	-2.0991602736
C	3.8888594485	1.7134607452	0.4787680815
H	4.5780357105	1.7661078843	1.3216564934
H	4.4555617179	1.6370947238	-0.4560473566
H	3.2647208346	2.6111407909	0.4601193657
O	-1.3109078430	2.2549885365	-0.0179963024
H	3.0658739686	-2.1513239749	-1.1502377351
H	0.6455419035	-3.3633834111	-0.8414194908
C	-2.2623137132	1.3055919439	0.3383918794
O	-2.5357619707	1.2845541466	1.5518223353
C	-3.3348989010	1.0284514647	-0.6931491570
H	-4.0688761414	0.3566758011	-0.2480254787
H	-3.8257768985	1.9799961070	-0.9254621503
H	-2.9662501315	0.5941378352	-1.6224060849
O	-3.0932764367	-2.1824073488	-0.1650844258
H	-3.6848528265	-1.8749155248	-0.8630946875
H	-2.4139746345	-1.4695331535	-0.0635548951
O	0.4609448596	0.8228524582	2.3369561829
H	-0.0836113825	0.5681213626	1.5650414681
H	1.3557150968	0.9038120001	1.9719582252
O	-0.7869493483	-2.2504050103	2.0078908462

H	-0.4992550710	-2.9368701557	1.3894188915
H	-0.9067686307	-1.4768051934	1.4137246472

D-Xylopyranoside

alpha-D-Xyl-2Ac
-1 1

C	-2.0849047320	-0.7772438285	0.1213787665
C	-1.5034779830	0.6294910902	0.0153093354
C	-0.0573053885	0.6609887819	0.5146285683
C	0.7050216359	-0.4259786753	-0.2459965176
C	0.0365231915	-1.7986206287	-0.1604437604
H	-1.5179756652	0.9524804368	-1.0356773197
H	-2.1389122899	-1.0809328683	1.1745730168
H	0.7733668100	-0.1453638874	-1.3021578303
H	0.5257311264	-2.5237351971	-0.8222925129
H	-0.0781516610	0.3812958516	1.5851129805
O	-1.2940058503	-1.7061876637	-0.6252313767
O	0.1030813656	-2.2357853602	1.1691497809
O	2.0253041996	-0.4883030371	0.3117592727
O	0.5425788688	1.9069192890	0.3440068844
O	-2.2573166074	1.5334689320	0.8136368567
H	-3.1174264377	1.6740100241	0.3938594088
C	-0.3880828929	-3.5617657182	1.3436449304
H	-0.1699994088	-3.8464469458	2.3731108390
H	-1.4687890680	-3.6057647237	1.1757246483
H	0.1173063282	-4.2494709097	0.6555808370
C	3.0350876895	-0.8566741234	-0.4919055901
O	2.8695552674	-1.1807831328	-1.6544566388
C	4.3505176817	-0.8121641475	0.2176478199
H	4.5372650768	0.2076053234	0.5643062635
H	4.3119670400	-1.4647836644	1.0933558221
H	5.1438155420	-1.1316846093	-0.4557006735
O	0.0758477969	3.2132210114	2.5093815276
H	-0.8803242102	3.1528137550	2.6248340095
O	-0.8370433218	3.6514784831	-1.0185076431
H	-1.7434391614	3.5575921261	-0.6981823141
O	2.5508630593	2.1130058431	-1.3340022350
H	2.4589724862	1.4068712422	-1.9866387091
H	0.2694515153	2.7094329747	1.6495978955
H	-0.3202627938	2.9558682436	-0.5046112775
H	1.7814627890	1.9939724609	-0.6995120899
H	-3.0873193082	-0.8163367182	-0.3100256948

alpha-D-Xyl-2Ac-H
0 1

C	-2.2454462637	-0.6160407038	-0.0574697514
C	-1.4553939218	0.6884600032	0.0177975270
C	-0.0471710207	0.4155902211	0.5219959041
C	0.5764874167	-0.6706236620	-0.3547797866
C	-0.2988058283	-1.9202734790	-0.4182396095
H	-1.3897654057	1.1430110312	-0.9815369522
H	-2.3837445361	-1.0235074673	0.9514765809
H	0.7097774623	-0.2934628777	-1.3718169286
H	0.0953737401	-2.6401850120	-1.1452975917
H	-0.0995754550	0.0625664578	1.5609205198

O	-1.5803304738	-1.5633435641	-0.8939009272
O	-0.3467615886	-2.4900759457	0.8588570879
O	1.8455755704	-1.0434809384	0.1949872959
O	0.7041212137	1.6144672077	0.4575099446
O	-2.0786526245	1.5898336272	0.9246717414
H	-2.9794385885	1.7689516189	0.6150193857
C	-1.0605913569	-3.7239470478	0.8907179102
H	-0.9131742958	-4.1464977604	1.8845998366
H	-2.1290560408	-3.5644053573	0.7159669969
H	-0.6644909810	-4.4116412503	0.1347237216
C	2.9738486604	-0.6876078994	-0.4436588242
O	2.9853872647	-0.0395759067	-1.4743223704
C	4.1924501546	-1.2169321959	0.2436341134
H	5.0523260416	-0.5996368557	-0.0143888985
H	4.0475872043	-1.2545261291	1.3234515269
H	4.3651234674	-2.2355793498	-0.1182490398
O	2.9742237592	1.3955046008	1.9687378653
H	2.9832225587	2.1375145829	2.5891419965
H	1.5280793744	1.5192414589	0.9873050921
H	3.7865230610	1.4935077227	1.4521446607
O	1.2913801710	2.2225804654	-2.2379982990
H	1.8933499566	1.4726748781	-2.3561418780
O	-0.8696422173	4.0878865059	0.1887849558
H	-1.3262510204	3.2924196837	0.5178025854
H	1.0126355439	2.1224541488	-1.3079134385
H	0.0358316913	3.7750244899	0.0589956541
H	-3.2238183431	-0.4427235117	-0.5092052372

alpha-D-Xyl-3Ac-a
-1 1

C	2.3606918586	-0.3066595791	0.1301458583
C	1.3022774386	-1.3310980240	-0.2604658966
C	-0.0709883245	-0.7847476541	0.0908785949
C	-0.3048014413	0.5849223287	-0.5505083829
C	0.8489780564	1.5108428739	-0.1393897342
H	1.3486504891	-1.5084976328	-1.3432618220
H	2.3847398707	-0.1953955305	1.2218567310
H	-0.2010429653	0.4516803659	-1.6435330456
H	0.7962210722	2.4614915601	-0.6849598347
H	-0.1689586898	-0.7186655908	1.1780231796
O	2.1017212320	0.9477816494	-0.4967029968
O	0.7637515215	1.7382525718	1.2396832552
O	-1.5527163700	1.1019510178	-0.2298201865
O	-1.0454599390	-1.6978297681	-0.4480492559
O	1.5888959895	-2.5260748168	0.4487671543
H	0.9848102699	-3.2163080178	0.1396951472
C	1.6883418166	2.7098097825	1.7155708053
H	1.4320382803	2.9072479335	2.7574332778
H	2.7166622888	2.3382472700	1.6577475547
H	1.6026460293	3.6357715665	1.1346681203
C	-2.1291422454	-2.1006578618	0.2290390432
O	-2.9788511025	-2.7076615040	-0.4052462242
C	-2.2432000603	-1.8180904601	1.6955735711
H	-1.3728154569	-2.2099615453	2.2290857281
H	-2.2939284660	-0.7395095247	1.8708725274
H	-3.1500070162	-2.2940886400	2.0640192892
O	-1.9158623150	3.1709271070	-1.7652758460

H	-1.7778531475	2.3818148396	-1.1523982069
H	-2.4789140016	2.8420914082	-2.4770166115
O	-1.9688353044	1.7873029487	2.2516029443
H	-1.7945965528	1.4967562768	1.3023986846
H	-1.0903410952	2.0422391009	2.5625838660
O	-3.2747444185	0.0601819706	-1.9101326105
H	-3.3004793893	-0.8964685343	-1.7721959721
H	-2.6255966919	0.4153307248	-1.2302203855
H	3.3444329698	-0.6287539125	-0.2144009005

alpha-D-Xyl-3Ac-b
-1 1

C	1.6564208138	0.4486747528	0.2304087495
C	0.5707403348	-0.4542643445	-0.3629650916
C	-0.7537310815	0.3102796702	-0.3200399570
C	-0.6292705449	1.6715496877	-0.9871274836
C	0.5099795301	2.4559487775	-0.3411025317
H	0.8108761763	-0.6218044709	-1.4293597238
H	1.4775967016	0.5830789718	1.3061884947
H	-0.3673036571	1.5324217982	-2.0439534744
H	0.6982996074	3.3910711354	-0.8824988628
H	-1.0961123132	0.4249246115	0.7120966336
O	1.7115053579	1.7206744591	-0.4273772049
O	0.1531040889	2.7298982061	0.9868862534
O	-1.8190855855	2.4312622871	-0.8704936888
O	-1.7460228798	-0.4228512312	-1.0669322635
C	1.1200349057	3.5218663888	1.6707478062
H	0.6738063203	3.8122748383	2.6220799447
H	2.0355640423	2.9513618139	1.8563151056
H	1.3609992429	4.4190071058	1.0882476051
H	-2.5146687948	1.9819016960	-1.3720052203
C	-2.6878316340	-1.1147742464	-0.4116940816
O	-2.7754593781	-1.1523247814	0.8033355090
C	-3.6447223402	-1.7867314569	-1.3475894997
H	-3.1661658996	-2.0280076423	-2.2969940566
H	-4.0421290597	-2.6848393127	-0.8753008259
H	-4.4707111753	-1.0939098493	-1.5382695060
O	0.4935697134	-1.6623065029	0.3289821544
O	-0.7100703586	-3.4245048423	-1.1087874031
H	-0.9911918570	-2.9735764008	-1.9146101505
H	-0.2424388192	-2.7163872383	-0.5501148908
H	1.8743274647	-2.1560419656	0.9565899658
O	2.7183558850	-2.5028934506	1.3843144853
H	3.1228839776	-1.7348559112	1.8063759263
H	-0.0965832726	-1.4464019831	1.7997042037
O	-0.5008535772	-1.3191346011	2.7144314518
H	-1.4226336278	-1.1050812300	2.5138588303
H	2.6402612834	-0.0023667886	0.0888580072

alpha-D-Xyl-3Ac-H
0 1

C	-2.3747916501	0.3116552176	0.0446373835
C	-1.1641708216	1.2212562627	0.2385204462
C	0.0942069642	0.3763320970	0.3254098812
C	0.1966790511	-0.5659719443	-0.8739451895
C	-1.0957267962	-1.3652242940	-1.0358252044
H	-1.0714410829	1.8979059998	-0.6216698789

H	-2.5177126559	-0.3140589797	0.9340983365
H	0.3258574200	0.0419668736	-1.7796496289
H	-1.0871662371	-1.9240290654	-1.9792222000
H	0.0950431949	-0.1890739919	1.2620879776
O	-2.2094809343	-0.4987744341	-1.1189623343
O	-1.1879154139	-2.2391577693	0.0508846226
O	1.2084856182	1.2872641318	0.2821907122
O	-1.2767503952	1.9579587612	1.4438925046
H	-1.9301665614	2.6601873658	1.3186858674
C	-2.2684603392	-3.1611161897	-0.0542234155
H	-2.1713009266	-3.8560431614	0.7802406923
H	-3.2327501561	-2.6469917218	0.0089780202
H	-2.2080682401	-3.7112314922	-1.0004455239
C	2.3166136231	1.1686467607	1.0358842157
O	3.2110346318	1.9666286590	0.8188039215
C	2.3888957034	0.1013930368	2.0820985055
H	1.5863222728	0.2314190217	2.8136801901
H	2.2791565374	-0.8859366259	1.6267875251
H	3.3544057627	0.1761642217	2.5788808986
O	1.2645169301	-1.4785180754	-0.7334399168
H	2.1088586673	-1.0348479761	-0.9888539258
H	1.1098267393	-2.6635106787	0.7384478273
H	1.6379015608	2.0963481672	-1.4929644111
O	3.5001069741	-0.1992994279	-1.5735620405
O	1.1608558616	-3.2978656204	1.4784343070
O	2.1483367800	2.1347157500	-2.3189495466
H	4.1034802169	0.0312308217	-0.8538137893
H	3.1263049543	0.6527269716	-1.8827804981
H	2.0812020764	-3.2702503769	1.7693686298
H	1.5230665766	1.8872629093	-3.0139353475
H	-3.2729350265	0.9098285057	-0.1184048340

alpha-D-Xyl-4Ac
-1 1

C	-0.6327175833	-1.6800020137	-0.0124790465
C	0.3251397000	-0.5260450946	0.2515445147
C	-0.3395159768	0.8274557327	-0.0162028601
C	-1.6322022228	0.8764304284	0.8143569800
C	-2.5105210480	-0.3532274994	0.5749197341
H	0.6938468882	-0.5659425627	1.2805353077
H	-0.9181214450	-1.7010771602	-1.0715966883
H	-1.3634813367	0.8934824442	1.8766508459
H	-3.3530664447	-0.3816012538	1.2768167903
H	-0.6244237198	0.8436880466	-1.0869319079
O	-1.7874563807	-1.5447382793	0.8137780480
O	-2.9849142532	-0.2812849978	-0.7434586447
O	-2.3723233824	2.0616150481	0.5740907770
O	0.5012783992	1.8979636220	0.2798885388
O	1.4261678212	-0.7004186055	-0.6590629440
C	-3.9327032434	-1.2967248000	-1.0586822153
H	-4.3302001870	-1.0637895805	-2.0466967649
H	-3.4577190002	-2.2826879911	-1.0797844863
H	-4.7477485943	-1.2991336705	-0.3254004039
H	-2.6319759126	2.0665684834	-0.3601554490
C	2.6827789634	-0.5644526189	-0.2139010249
O	2.9635959629	-0.2931768664	0.9408291993
C	3.6925102958	-0.8440859873	-1.2834043080

H	3.2928954145	-0.6247392217	-2.2736206605
H	4.5937075381	-0.2621937971	-1.0910892617
H	3.9442407137	-1.9085162451	-1.2342066874
O	2.3640638330	2.1547064856	-1.5029963707
H	1.6403838914	2.0163977079	-0.8089647770
H	2.1806534887	1.5079928473	-2.1958583381
O	-0.4463606956	4.0067207749	-0.9087004632
H	-1.3586849588	4.1068351638	-0.6101573278
H	-0.1032639854	3.1952353964	-0.4160790939
O	1.5686017126	1.6663043191	2.6345277936
H	2.1700199599	0.9254901635	2.4744254449
H	1.1235515142	1.7835514217	1.7357623325
H	-0.1725997662	-2.6334394198	0.2516581175

alpha-D-Xyl-4Ac-H
0 1

C	1.4961270697	-2.4740781210	0.1302116432
C	-0.0304274639	-2.3795188518	0.1411598432
C	-0.4367576194	-0.9214531868	0.0374584753
C	0.2098095256	-0.2784327979	-1.1815460204
C	1.7275424858	-0.4735951023	-1.1210846837
H	-0.4442328420	-2.9276823594	-0.7147327153
H	1.8984905722	-2.0115783112	1.0399828444
H	-0.1507910677	-0.7922472557	-2.0826999954
H	2.2076667485	-0.1189013701	-2.0409951445
H	-0.1319108422	-0.4011575820	0.9501801759
O	2.0416872562	-1.8476736053	-1.0320568646
O	2.2046792826	0.2435330752	-0.0158818663
O	-1.8599049123	-0.8624807105	-0.1279991368
O	-0.5494165316	-2.8877053359	1.3581583608
H	-0.5111770204	-3.8536324056	1.3324615394
C	3.6270591164	0.2862389247	0.0742756704
H	3.8691986222	0.9745786097	0.8848947117
H	4.0325064449	-0.7042968423	0.3008912414
H	4.0542142925	0.6541941120	-0.8650989426
C	-2.6162275116	0.0789094070	0.4668135026
O	-3.7819092235	0.1484474921	0.1187661365
C	-2.0232309533	0.9372222582	1.5425221994
H	-2.7903101545	1.6273023851	1.8891369353
H	-1.6914472156	0.3053936243	2.3725584805
H	-1.1602754037	1.4969341424	1.1756157394
O	-0.1457483356	1.0844971982	-1.2038703967
H	0.0865926106	1.4505844859	-2.0863606175
H	1.3816685286	1.7547852430	0.7102021014
H	-1.2115589170	2.9496018379	-0.7123148379
O	0.6090318886	1.9647086006	-3.6826354378
O	1.1368548546	2.5337006277	1.2461336250
O	-0.8991545679	3.7550760413	-0.2762016941
H	1.1875996140	2.7359076687	-3.6084665814
H	-0.1283110219	2.2566459514	-4.2358190250
H	1.9632878025	3.0145185938	1.3856552005
H	-0.1617594103	3.4319381470	0.2781389709
H	1.8095473593	-3.5186635982	0.0934316231

alpha-D-Xyl-IN1
-1 1

C	-2.7830201223	-0.2507769722	-0.1503597228

C	-1.6699174674	-1.2924766309	-0.3493559099
C	-0.4399142956	-0.4900342898	-0.6966187948
C	-0.1512973134	0.4821980273	0.4260362126
C	-1.2682487069	1.5014445367	0.5402071761
H	-1.5075549804	-1.8626652266	0.5744059232
H	-3.0023951527	0.2176843445	-1.1186431454
H	-0.1261056513	-0.0584002875	1.3828227339
H	-1.1541141037	2.1725068387	1.3989903086
H	-0.6292084663	0.0500382875	-1.6362874480
O	-2.4463692402	0.7505429948	0.8165409240
O	-1.3651759922	2.2359128326	-0.6445007242
O	1.1394742810	0.9437556515	0.0921667027
O	0.7966265907	-1.1737796692	-0.7775257504
O	-2.1070423483	-2.1462133150	-1.3942494963
H	-1.4786580686	-2.8768312273	-1.4774478360
C	-2.3673768745	3.2490665724	-0.5891185359
H	-2.2708769261	3.8381735046	-1.5010757274
H	-3.3675653273	2.8075413771	-0.5424359775
H	-2.2100937598	3.8932339398	0.2836593336
C	1.8402056991	-0.1697328559	-0.5900816055
O	2.8123711649	-0.6668982706	0.1659667062
C	2.3016632521	0.3530370974	-1.9398196909
H	2.7973392675	-0.4533005624	-2.4867085343
H	1.4583461643	0.7242861965	-2.5278570173
H	3.0138343557	1.1685689933	-1.7850033603
O	4.1024591235	1.4082924458	1.3395328589
H	3.6331740165	0.6626130223	0.8910596028
H	3.4298081474	1.8185364476	1.8977248780
O	5.1927713490	-1.0088762975	-1.0179263764
H	5.7917563765	-1.2642483491	-0.3054228536
H	4.3057280094	-0.9001213701	-0.5873077907
O	2.0187564187	-1.0543724456	2.6747683427
H	1.6591579895	-0.1993799416	2.9437297178
H	2.2878150822	-0.9290573371	1.7238679022
H	-3.6875470408	-0.7328587318	0.2226621944

alpha-D-Xyl-IN2

-1 1

C	1.7177773402	1.3414628900	-0.0505782838
C	0.3562466475	0.7166173370	0.1587119569
C	0.4121072685	-0.7472308145	-0.2082905440
C	1.3272093583	-1.4686615187	0.7530203600
C	2.6941646104	-0.7491240658	0.6699225710
H	0.0859093532	0.8223271008	1.2204606041
H	2.0614642645	1.2287611984	-1.0859120313
H	0.9475526664	-1.3803343236	1.7758196905
H	3.3610262279	-1.0933374483	1.4693203404
H	0.8008556996	-0.8544694765	-1.2335478373
O	2.5955430890	0.6508298294	0.8570377151
O	3.2362124899	-1.0676398878	-0.5839815105
O	1.4728529504	-2.8506210435	0.4893060205
O	-0.9579636771	-1.0957011819	-0.1803957126
O	-0.7099571923	1.1572303384	-0.6598136438
C	4.5773827233	-0.6164097021	-0.7496879862
H	4.9356871425	-1.0299753679	-1.6924244110
H	4.6207850053	0.4762523903	-0.7900472978
H	5.2047892721	-0.9781154992	0.0732061095

H	1.8886111717	-2.9457367274	-0.3814886638
C	-1.7099684236	0.0809030236	-0.6379359947
C	-2.1595123418	-0.1386095358	-2.0713091516
H	-1.3133885842	-0.4221602113	-2.7025664421
H	-2.9118848059	-0.9321901522	-2.0941748582
H	-2.6029155537	0.7818111097	-2.4606983958
H	-2.2057122875	-0.0568573938	1.7962797276
O	-1.9674284042	-0.3022931522	2.7290527161
H	-1.2128466600	-0.8983623294	2.6391091155
H	-4.1012350697	0.9219278729	-0.4582142906
O	-4.9731257517	1.2242572982	-0.8288086079
H	-5.2565950982	0.5065049911	-1.4091259082
H	-3.6524641648	-1.0968924212	0.3225561561
O	-4.2156621526	-1.9092213886	0.3666635976
H	-4.7561566242	-1.8792484830	-0.4328724734
O	-2.6943225850	0.3316105181	0.2235339048
H	1.7566085756	2.3925076468	0.2366510090

alpha-D-Xyl-TS1a
-1 1

C	1.8845537289	-1.8480901225	-0.1929539656
C	0.3571392914	-1.7493647623	-0.1667929259
C	0.0088914865	-0.3461660057	0.3145669395
C	0.6833258358	0.6453838459	-0.6259160124
C	2.1922045968	0.4985462993	-0.6182445955
H	-0.0404376109	-1.9067126066	-1.1812339347
H	2.2689648859	-1.7745271994	0.8329756901
H	0.3469997568	0.4725830743	-1.6568555482
H	2.6841084758	1.1295732000	-1.3679076336
H	0.4485658664	-0.2321295831	1.3227583488
O	2.4762134902	-0.8342676067	-1.0142605963
O	2.6814264862	0.7933540556	0.6593582626
O	0.2169329122	1.9056188744	-0.1697977748
O	-0.1089711711	-2.7669558405	0.7052743902
H	-1.0808525123	-2.7939890714	0.6158218388
C	4.1009656211	0.7007410004	0.7462942240
H	4.3841035997	1.0974462264	1.7213419515
H	4.4315617910	-0.3394612531	0.6651887036
H	4.5728198417	1.2961092752	-0.0441178936
C	-1.1867112869	1.9318211838	-0.1787094657
O	-1.7788886654	2.0017988110	-1.2720066099
C	-1.7307439826	2.5786506273	1.0629507773
H	-1.1993509872	2.2382764668	1.9522291653
H	-1.6002899264	3.6616327787	0.9622646006
H	-2.7964877594	2.3623795383	1.1507985429
O	-1.3328340472	0.0318374880	0.3244695966
O	-2.1914034329	-0.3343704908	2.7852760357
H	-1.8730667107	-0.1485962484	1.8553930272
O	-2.8043715230	-2.1846200626	0.0838149064
H	-2.3730715343	-1.2925057472	0.0454377118
H	-3.0295897886	-2.4183031013	-0.8257822437
H	-1.9013369726	0.5180737911	-2.2859842235
O	-2.0519532143	-0.2755026415	-2.8392164788
H	2.2002958780	-2.8006558662	-0.6213257215
H	-2.0736050812	0.4930171811	3.2678276432
H	-1.8327893372	-1.0158475081	-2.2585077323

alpha-D-Xyl-TS1b
-1 1

C	2.3123398506	-1.6569338877	-0.4551069191
C	0.9417374282	-2.1427186561	0.0315530919
C	0.2192526146	-0.9201355344	0.5461348412
C	0.1120290969	0.1255541989	-0.5582751235
C	1.5197628650	0.5550021496	-0.9603415851
H	0.3795640574	-2.5873716547	-0.8001505624
H	2.8988577598	-1.3244058548	0.4112791725
H	-0.3084840272	-0.3669720215	-1.4537291755
H	1.5179965008	1.2419964829	-1.8154825106
H	0.7689742607	-0.5205013919	1.4082881052
O	2.2093616790	-0.6036050891	-1.4162812313
O	2.1580786497	1.1438537035	0.1363882557
O	-0.7566370319	1.0923660599	-0.0740207876
O	-1.1342708425	-1.1622226649	0.9025506857
O	1.1907501213	-3.1119931128	1.0381277181
H	0.3462972869	-3.5022171328	1.3040080529
C	3.4398157831	1.6800026566	-0.1791244933
H	3.7975623026	2.1918433797	0.7150223898
H	4.1424765898	0.8853735468	-0.4492097159
H	3.3616208604	2.3962905062	-1.0055364467
C	-1.8486217077	0.0199680895	1.1338439904
O	-3.0233409774	0.0154110085	0.7130327231
C	-1.4982997839	0.7612850127	2.3981545552
H	-1.9888938786	0.2409342537	3.2277400614
H	-0.4254083903	0.8010446086	2.5843136237
H	-1.8936978722	1.7763061649	2.3366176313
O	-2.4063991105	1.4311227322	-2.1424212501
H	-1.8136489487	1.3802212031	-1.3468221232
H	-2.7576201307	0.5281603290	-2.2186776828
O	0.3180362888	3.3379153469	0.9062858348
H	-0.0756006164	2.4911389498	0.5679094902
H	1.2677675568	3.1618549040	0.8886351046
O	-3.1605668160	-1.3283012205	-1.6681087925
H	-4.0755523353	-1.5912626559	-1.8299088147
H	-3.1513932290	-0.9424761238	-0.7613392380
H	2.8458071460	-2.4688272860	-0.9514368755

alpha-D-Xyl-TS2a
-1 1

C	0.6028291410	-1.2834491384	0.1886087316
C	-0.2116647929	-0.1354395307	-0.3865165443
C	0.5862753158	1.1472444365	-0.2476884426
C	1.8731431083	1.0507986667	-1.0334167259
C	2.6271505128	-0.1848430849	-0.5119691200
H	-0.3580688591	-0.3415978118	-1.4612935857
H	0.8193451805	-1.1297269376	1.2532679780
H	1.6485047762	0.8793019649	-2.0935333499
H	3.4926858366	-0.4051242660	-1.1486922214
H	0.8157121413	1.3273396006	0.8129936547
O	1.8237724302	-1.3486729716	-0.5696913015
O	3.0479685107	0.0894272704	0.7968618047
O	2.7194392642	2.1770527205	-0.8817279192
O	-0.3144638777	2.1322782407	-0.7252114897
C	3.8578139914	-0.9447168493	1.3483685735
H	4.2437008830	-0.5706415548	2.2968504854

H	3.2721751432	-1.8521263863	1.5255699953
H	4.6926180752	-1.1750900670	0.6758632440
H	2.3178356416	2.9208534412	-1.3518941223
C	-1.6132654580	1.9460380353	-0.1683246709
O	-2.5533708818	2.0928790461	-0.9850562977
C	-1.7432247167	2.5030131799	1.2323945268
H	-0.8612445791	2.3098083076	1.8447860709
H	-1.8853218315	3.5848213069	1.1464213927
H	-2.6231090593	2.0715257599	1.7143921089
O	-1.4312831462	0.1805916461	0.2224682309
O	-3.2542662737	-0.7795265864	-1.6747850688
H	-3.5394932588	-0.1097751568	-2.3092500750
H	-2.6769766264	-0.2984950129	-1.0452144232
H	-2.3036309757	-1.3566825134	0.7823675515
O	-2.7448118136	-2.2051355496	0.9826691842
H	-3.2643835837	-2.3916119110	0.1892371413
H	-1.1879955334	0.0503506411	2.0228666889
O	-1.0650994755	-0.1097713379	2.9836354600
H	-0.8020433705	-1.0373338224	3.0446183488
H	0.1146486717	-2.2497171343	0.0526048353

alpha-D-Xyl-TS2b
-1 1

C	1.8971368776	0.8466276302	-0.3908739620
C	0.4202366856	0.9643796042	-0.0907004443
C	-0.2678039871	-0.3795815290	-0.2506622635
C	0.3415018700	-1.3562445872	0.7462390510
C	1.8617474850	-1.3822786901	0.5150696400
H	0.2954974011	1.3265373171	0.9405893627
H	2.0710739701	0.4911050058	-1.4138127564
H	0.1718416579	-0.9903439475	1.7686123124
H	2.3652622867	-1.9378802442	1.3159835697
H	-0.0629711149	-0.7559465866	-1.2686051593
O	2.4262179525	-0.0833208661	0.5673147636
O	2.1047132978	-1.9982025227	-0.7208660506
O	-0.1418272714	-2.6808599871	0.6103499523
O	-0.3094419039	1.7921770828	-0.9825370266
C	3.4922762379	-2.1767887025	-0.9888415640
H	3.5655537624	-2.8046949980	-1.8769991098
H	3.9832855665	-1.2173193683	-1.1792389965
H	3.9830834268	-2.6749913470	-0.1443350246
H	-1.0642679322	-2.6873111095	0.9313991941
C	-1.6948611327	1.6956561213	-0.6768771019
O	-2.1173331501	2.3498143425	0.3021210872
C	-2.5050526590	1.5712721660	-1.9419053606
H	-2.0628534988	0.8497667207	-2.6308345462
H	-3.5264281518	1.2772809711	-1.6931191162
H	-2.5318085218	2.5544364136	-2.4236380259
O	-1.6297078907	-0.0770301743	-0.1154349511
H	-2.6042751944	-1.3271484231	0.8744083853
H	-1.8605942998	0.4605214447	1.6750431529
H	-2.4144438893	-1.1083500772	-1.2692030180
O	-2.7956084141	-2.1210256235	1.4141537577
O	-1.9782602446	0.5413125722	2.6445285918
O	-2.8221791398	-1.7851060847	-1.8612881166
H	-2.7565527785	-1.8047748552	2.3276330178
H	-2.0360154182	1.4931928844	2.7978162359

H	-2.9338293889	-1.3463850191	-2.7137162215
H	2.4358227936	1.7819776663	-0.2341023775

beta-D-Xyl-2Ac
-1 1

C	-0.3124847421	-2.7869353959	0.0545733093
C	-1.3061425896	-1.6356683015	-0.0910728457
C	-0.7569696868	-0.3668999352	0.5637730678
C	0.6501710026	-0.1231421650	0.0058613862
C	1.5423132351	-1.3481146820	0.1569319125
H	-1.4837868829	-1.4352084990	-1.1576546222
H	-0.1847524758	-3.0314167276	1.1192264662
H	0.5985767668	0.1544693046	-1.0514593490
H	1.6686852012	-1.6095020108	1.2220030478
H	-0.6432419841	-0.5901255078	1.6439083665
O	0.9448930672	-2.4397865502	-0.5211707929
O	2.7755800351	-1.0818496824	-0.4269765399
O	1.2269984542	0.9499138673	0.7662534386
O	-1.5736905636	0.7430709359	0.3752514135
O	-2.5275417513	-1.9670001864	0.5567319090
H	-2.9837869851	-2.6389377275	0.0311968638
C	3.7879209736	-2.0206218098	-0.0635932752
H	4.7161881831	-1.6716586357	-0.5157978098
H	3.8998414286	-2.0551400794	1.0256664070
H	3.5514341012	-3.0190908621	-0.4424907703
C	1.8908438341	1.9264174392	0.1319827878
O	2.0294998713	1.9661627629	-1.0784974338
C	2.3902655234	2.9681838112	1.0820414899
H	1.5562445297	3.6379513554	1.3179488009
H	2.7381794721	2.5125630648	2.0099332540
H	3.1848483270	3.5439595667	0.6097283343
O	-1.1354774306	2.3369333076	2.3443254168
H	-0.1741514956	2.3678365950	2.4262897475
O	-3.8017491351	0.3578620934	-0.9375397762
H	-4.0685315517	-0.5476635391	-0.7338797225
O	-0.9470035832	2.4302775519	-1.5357117724
H	0.0046069851	2.3608607419	-1.6939515149
H	-1.3118174184	1.7170686251	1.5589527579
H	-2.9501552774	0.4807990569	-0.4179415529
H	-1.1416914900	1.7606805246	-0.8134647028
H	-0.6660038483	-3.6747573470	-0.4734650970

beta-D-Xyl-2Ac-H
0 1

C	-2.0217699999	-0.5953586787	0.4166167867
C	-1.3309356671	0.7673878627	0.3604178560
C	0.1372861434	0.6083148118	0.7207122033
C	0.7516454707	-0.4856516303	-0.1466902809
C	-0.0447271847	-1.7811299310	-0.0502397595
H	-1.4020179534	1.1772809502	-0.6570948247
H	-2.0069207287	-0.9725436951	1.4492022580
H	0.7778905326	-0.1717320351	-1.1936350993
H	-0.0466092795	-2.1720724814	0.9819551789
H	0.2116284904	0.3138211094	1.7779947517
O	-1.3777944598	-1.5205314790	-0.4542549912
O	0.5170582359	-2.7021676608	-0.9242961904
O	2.0807495525	-0.7235428697	0.3309375686
O	0.8126880690	1.8380591868	0.5196881276

O	-1.9165264454	1.6559994206	1.2973108844
H	-2.7669983281	1.9587285369	0.9485559841
C	0.0193468080	-4.0295317708	-0.7470668858
H	0.5850762219	-4.6681091582	-1.4250988307
H	0.1771474706	-4.3575617087	0.2862183067
H	-1.0440257943	-4.0847811908	-0.9953340254
C	3.1026955280	-0.6996893063	-0.5412504916
O	2.9604559505	-0.4775055442	-1.7303589388
C	4.4219569877	-0.9496459171	0.1172298726
H	4.9487379497	0.0066387504	0.1900358489
H	4.3009480845	-1.3724934822	1.1138017817
H	5.0115657785	-1.6151883514	-0.5149371905
O	3.0186463120	1.5341640260	2.0699429978
H	2.9855844708	0.5741544005	2.1924247330
H	1.6290623610	1.8398451452	1.0723592024
H	2.9445181715	1.9078212293	2.9586545380
O	1.6907134120	2.1718663324	-2.1941810756
H	2.1597484360	1.3295096244	-2.2945218483
O	-0.7492689137	4.0560123711	-0.3356114639
H	-1.6452421661	3.8189104222	-0.0626125219
H	1.3464304541	2.1345806538	-1.2833418772
H	-0.1960822585	3.3350006497	0.0156342872
H	-3.0571080123	-0.5128914826	0.0820436282

beta-D-Xyl-3Ac-a
-1 1

C	2.3626944441	-0.3696738336	0.2939915994
C	1.3028402932	-1.3572769552	-0.1809057334
C	-0.0691024435	-0.7627950286	0.0881545690
C	-0.2327672762	0.6323515153	-0.5213625871
C	0.9311190294	1.4859288876	-0.0044187308
H	1.4186578531	-1.5259122857	-1.2596437833
H	2.3017940688	-0.2571945054	1.3866229710
H	-0.1002822659	0.5458372994	-1.6167526162
H	0.8777800533	1.5915183260	1.0940452229
H	-0.2226093338	-0.7038396464	1.1709706283
O	2.1788943212	0.8868322137	-0.3437008374
O	0.8750722338	2.7336975464	-0.6136933845
O	-1.4689343619	1.1779934756	-0.2001023827
O	-1.0453118598	-1.6323273273	-0.5116840826
O	1.4945989870	-2.5673414411	0.5341894159
H	0.8655327334	-3.2232871437	0.2004547797
C	1.6954416913	3.7148780323	0.0180667822
H	1.5078171819	4.6587772864	-0.4940392783
H	1.4240742074	3.8108380976	1.0755091081
H	2.7554990593	3.4584184690	-0.0683712695
C	-2.1324062179	-2.0786712393	0.1322654897
O	-2.9569278229	-2.6826099452	-0.5368620955
C	-2.2883076989	-1.8320770715	1.6014872657
H	-1.4314494589	-2.2311585543	2.1512640264
H	-2.3532824482	-0.7579806430	1.7990683993
H	-3.2017786909	-2.3223029820	1.9327140036
O	-1.9654728442	3.1531921374	-1.8343708943
H	-1.7490946750	2.4150743144	-1.1847323082
H	-2.7932566542	2.8829683697	-2.2508289845
O	-1.6399653721	1.7005122757	2.3339013397
H	-1.5675103184	1.4841419676	1.3483126245

H	-1.1156549710	1.0220614461	2.7784122452
O	-3.3297531947	0.2043385905	-1.7910155336
H	-3.3648563720	-0.7576198881	-1.7033867995
H	-2.6162570365	0.5130988687	-1.1582770506
H	3.3603607598	-0.7222805490	0.0281440616

beta-D-Xyl-3Ac-b
-1 1

C	1.5806448013	0.5785411396	0.6863320605
C	0.6225723155	-0.4020468873	0.0050797528
C	-0.6945235279	0.3371713948	-0.2304056129
C	-0.4931000309	1.6530301783	-0.9699070031
C	0.5288555456	2.4909611458	-0.2105394646
H	1.0408420111	-0.6567267610	-0.9869367471
H	1.2006686370	0.8293368258	1.6884168132
H	-0.0959497609	1.4592558648	-1.9761142614
H	0.1512328390	2.7417640789	0.7964195245
H	-1.1895456154	0.5294809370	0.7274589541
O	1.7403606570	1.7678617020	-0.0877073716
O	0.7806203399	3.6513015906	-0.9361742807
O	-1.7001247584	2.3912524739	-1.0413191420
O	-1.5617217279	-0.4647724481	-1.0550716824
C	1.5273850416	4.6230235053	-0.2038756560
H	1.5737571287	5.5167685749	-0.8260501581
H	1.0217179396	4.8578665961	0.7394383540
H	2.5410155639	4.2671481497	0.0001595873
H	-2.3553995583	1.8533384066	-1.5097576053
C	-2.5981834509	-1.1068972312	-0.4977218716
O	-2.8677147819	-1.0497683304	0.6893313145
C	-3.4088732623	-1.8514674240	-1.5124250358
H	-4.2076301933	-1.1860573490	-1.8557303292
H	-2.8015121317	-2.1400724672	-2.3702012139
H	-3.8595508858	-2.7266047987	-1.0446896302
O	0.4497370276	-1.5460775942	0.7828087197
O	-0.5981397702	-3.4368927636	-0.6513418527
H	-0.7038653079	-3.0749153242	-1.5401467730
H	-0.2160062027	-2.6808531449	-0.0991101771
H	1.7867168232	-2.3914999320	0.6428344814
O	2.6362186997	-2.9190855543	0.5060004125
H	2.3788017438	-3.6776749996	-0.0322294665
H	-0.3607502534	-1.2485335405	2.1164838787
O	-0.9182267450	-1.0641658187	2.9375754284
H	-1.8000675481	-0.9115317893	2.5709709848
H	2.5717680385	0.1316134042	0.7850593689

beta-D-Xyl-3Ac-H
0 1

C	-2.1480820033	-0.1548434466	0.1827645654
C	-1.0945724576	0.9393225354	0.3349907708
C	0.2827684889	0.3019704696	0.3454636680
C	0.4875974645	-0.6405445711	-0.8420922100
C	-0.6900152980	-1.6066895550	-0.9551319418
H	-1.1601247916	1.6307039212	-0.5152579762
H	-2.1218538345	-0.8234835886	1.0552734300
H	0.5351160061	-0.0481884523	-1.7672334077
H	-0.7117624634	-2.2875153450	-0.0862506414
H	0.4144227618	-0.2520802473	1.2812989570

O	-1.9109929395	-0.8878751171	-1.0125280671
O	-0.5539021545	-2.3240426680	-2.1373821005
O	1.2403721131	1.3710592725	0.2483942131
O	-1.2547562889	1.6337876046	1.5596605330
H	-2.0205792098	2.2203206489	1.4859781652
C	-1.3728529007	-3.4932226143	-2.1862380003
H	-1.1817658601	-3.9678477736	-3.1484807901
H	-1.1004364673	-4.1786074603	-1.3764524505
H	-2.4326250610	-3.2331688354	-2.1116343946
C	2.3664038471	1.4610489176	0.9808965792
O	3.1002929765	2.4006754203	0.7335349235
C	2.6554796872	0.4325713525	2.0286170337
H	1.8423148499	0.3806985128	2.7578257241
H	2.7650054552	-0.5500658852	1.5616318630
H	3.5818363220	0.7087616743	2.5288094803
O	1.6600232954	-1.4119157894	-0.6775294130
H	2.4486405987	-0.8696346093	-0.9199179792
H	1.5317979584	-2.3588683113	0.9243001294
H	1.5942703694	2.1478951410	-1.5665603436
O	3.7485412924	0.1048688115	-1.4961092207
O	1.3751132760	-2.8666776043	1.7431720597
O	2.1780194710	2.2558888922	-2.3358065783
H	4.3456119125	0.4087867136	-0.7988853993
H	3.2981134685	0.9100988675	-1.8292229387
H	1.2775111507	-2.1980587675	2.4337189693
H	1.6591257505	1.9558239360	-3.0944493232
H	-3.1438520154	0.2836025102	0.0985614312

beta-D-Xyl-4Ac
-1 1

C	-0.5308481169	-1.9391553796	-0.2444632390
C	0.3952398437	-0.7813173420	0.1040915073
C	-0.1478838425	0.5444797187	-0.4237817172
C	-1.5956412424	0.7133843314	0.0649943066
C	-2.4202493096	-0.5270075006	-0.2592417554
H	0.5382451466	-0.7220293748	1.1881359689
H	-0.5962217694	-2.0508249117	-1.3363103826
H	-1.5947574273	0.8465437116	1.1579021088
H	-2.4981925114	-0.6625837982	-1.3523599419
H	-0.1936715749	0.4589285850	-1.5277450979
O	-1.8167014035	-1.6828093930	0.3055375853
O	-3.6865282412	-0.3899706107	0.3018997200
O	-2.2270064572	1.8131960690	-0.5684183371
O	0.6680004884	1.6124369590	-0.0457302660
O	1.6706886316	-0.9822373359	-0.5306532752
C	-4.6329259808	-1.3352799329	-0.1954402324
H	-5.5960009951	-1.0777446448	0.2455055322
H	-4.6987686065	-1.2671754439	-1.2869948404
H	-4.3619312815	-2.3546080404	0.0942958892
H	-1.8239376914	2.6301751686	-0.2132835361
C	2.6217262968	-1.6450464044	0.1416635384
O	2.4327528354	-2.1498433322	1.2344176916
C	3.9218119673	-1.6523079693	-0.5987466218
H	3.7558922041	-1.8503247646	-1.6592475912
H	4.3730322999	-0.6588812400	-0.5034473474
H	4.5898584340	-2.3960270520	-0.1670991694
O	2.5385910360	1.7076868555	-1.8416608844

H	1.8134284838	1.6934749782	-1.1390001038
H	2.8768739946	0.8032891412	-1.8552462630
O	-0.5786309885	3.7171987914	0.6401383280
H	-0.3244960950	4.4337353530	0.0450536471
H	-0.0152615449	2.9065289555	0.3681557677
O	2.6563295143	1.1270655494	1.6236750984
H	2.4691626928	0.3115994326	2.1066249359
H	1.8718905644	1.2667988227	1.0174758327
H	-0.1769213539	-2.8751279422	0.1909321544

beta-D-Xyl-4Ac-H
0 1

C	-1.2728049552	0.5555802191	-0.2504691286
C	0.2412888632	0.3451241993	-0.3020887477
C	0.5538553028	-0.9810803149	0.3777046393
C	-0.2598090233	-2.1369809279	-0.2086730424
C	-1.7410285117	-1.7519217737	-0.1847440131
H	0.5685956525	0.3326581315	-1.3463617983
H	-1.6011669740	0.6490655831	0.7955736425
H	0.0225638348	-2.3251490637	-1.2473023990
H	-2.0922174742	-1.6336915822	0.8566813816
H	0.2885650327	-0.8793410168	1.4387817768
O	-1.9373488252	-0.5314841307	-0.8822062300
O	-2.4618807965	-2.7454392211	-0.8349622680
O	-0.0244936927	-3.3389166030	0.4978400892
O	1.9603847149	-1.2770644760	0.4371877467
C	-3.8702448719	-2.6632385371	-0.6137073662
H	-4.3165745073	-3.5191563159	-1.1198108482
H	-4.0892000405	-2.7148895553	0.4583597586
H	-4.2799698145	-1.7392904811	-1.0312918118
C	2.6840805750	-1.4385015065	-0.6831615260
O	2.2086517474	-1.3871030520	-1.8016455940
C	4.1320019489	-1.6559634473	-0.3729200453
H	4.5816714028	-0.6851088911	-0.1409276710
H	4.2468395058	-2.3033417429	0.4977034317
H	4.6307557171	-2.0834413981	-1.2416020438
H	-0.2435736141	-3.2095806533	1.4337484435
O	0.9123132383	1.3737302214	0.4091358393
H	0.8766480642	2.1932316206	-0.1389067214
H	0.2408090815	1.4940370012	2.1507201067
H	2.4365281806	1.0382760676	1.4699786453
O	0.6258683867	3.4689585045	-1.2790268103
O	-0.0964410995	1.5154097370	3.0659301361
O	3.1636328676	0.8640153352	2.0948606300
H	-0.0910111006	3.1805513412	-1.8615114169
H	1.3954724514	3.5675302485	-1.8562506007
H	-1.0509108006	1.3964434821	2.9800910251
H	3.2844277723	-0.0934939133	2.0448952802
H	-1.5476488385	1.4618665319	-0.7939456197

beta-D-Xyl-IN1
-1 1

C	2.5878998098	-0.1723430191	0.5003589667
C	1.5383935579	-1.2555643542	0.1993439848
C	0.2249937020	-0.5177635435	0.1450601835
C	0.2684652390	0.5757379891	-0.8975297626
C	1.2924930920	1.6043177975	-0.4763439818

H	1.7521971476	-1.7399988484	-0.7618297801
H	2.4184263638	0.2077195502	1.5177202992
H	0.5816039820	0.1698627345	-1.8718132015
H	1.0750419704	2.0224265466	0.5203257514
H	0.0441960832	-0.0702193292	1.1370751701
O	2.5383303855	0.9014523652	-0.4386642906
O	1.3940120336	2.6133010433	-1.4250644810
O	-1.0845321874	0.9764900609	-0.9345341789
O	-0.9215311185	-1.2355865780	-0.2695118561
O	1.6071657406	-2.1928895924	1.2619808472
H	0.9802290427	-2.9085843961	1.0844713270
C	2.1867849496	3.7198193221	-0.9906277437
H	2.1396369488	4.4686300038	-1.7810129877
H	1.7768889954	4.1365197092	-0.0641872446
H	3.2260605324	3.4184980441	-0.8333453607
C	-1.9019788468	-0.2217106537	-0.6647740146
O	-2.7626308876	0.0400198083	0.3172129153
C	-2.5369324746	-0.6840062755	-1.9629637360
H	-3.2739489973	0.0572729261	-2.2829263143
H	-1.7818434112	-0.8077103825	-2.7434129420
H	-3.0446978128	-1.6382556659	-1.7983421192
O	-4.4930690471	-1.9778981103	0.6560746069
H	-3.8679199902	-1.2229257627	0.4957794441
H	-4.1410891958	-2.4229921597	1.4372769166
O	-4.0855451515	2.2806351495	-0.2460587622
H	-3.4150934637	2.9075438676	-0.5443033039
H	-3.5851690319	1.4460854743	-0.0394548409
O	-1.9878133153	-1.0935042790	2.6715456077
H	-1.1845546828	-1.5981776276	2.4880697677
H	-2.2137504991	-0.6556049778	1.8144146230
H	3.5943184373	-0.5882202063	0.4388566713

beta-D-Xyl-IN2
-1 1

C	1.6916659245	1.3920582847	-0.1742863066
C	0.3499689449	0.7385282055	0.0810415619
C	0.4251588382	-0.7212389777	-0.2977621340
C	1.4002461407	-1.4441841689	0.6014285436
C	2.7375861434	-0.6894904316	0.4411189559
H	0.1194955639	0.8347405347	1.1523817527
H	1.9970004396	1.2912799032	-1.2242768926
H	1.0822841045	-1.3949140275	1.6489282894
H	3.1171869362	-0.8333516158	-0.5867601172
H	0.7825320028	-0.8065768237	-1.3395479898
O	2.6075924442	0.7073539659	0.6903537048
O	3.6387656016	-1.1982040963	1.3687984086
O	1.5572197619	-2.8155660618	0.2884650676
O	-0.9326762596	-1.1036612208	-0.2286588405
O	-0.7541885666	1.1587879467	-0.6958483341
C	4.9970563919	-0.8543911628	1.0940226966
H	5.6038067902	-1.3502602689	1.8515839933
H	5.2836356723	-1.2130531368	0.0992535988
H	5.1489363592	0.2268772379	1.1554317771
H	1.7833887499	-2.9041623252	-0.6502352489
C	-1.7271347739	0.0574350519	-0.6567529524
C	-2.2080017792	-0.1635141578	-2.0798752667
H	-1.3725569656	-0.4278865227	-2.7332233848

H	-2.9450368538	-0.9715157793	-2.0886378598
H	-2.6776865291	0.7507303150	-2.4524513938
H	-2.1266793689	-0.0222732748	1.7929533102
O	-1.8302018913	-0.2211189982	2.7206921484
H	-0.9669224657	-0.6413459045	2.6146235042
H	-4.1475514725	0.8012863652	-0.4255420950
O	-5.0382456503	1.0538996892	-0.7858614309
H	-5.2398634728	0.3731764965	-1.4405465351
H	-3.6188046628	-1.1534507093	0.4109412729
O	-4.1769061196	-1.9654784854	0.5059205547
H	-4.9672396629	-1.7845963800	-0.0183686083
O	-2.6927991379	0.2788609235	0.2319652403
H	1.7192546829	2.4420741297	0.1181172495

beta-D-Xyl-TS1a
-1 1

C	1.8748605023	1.0904407951	-0.6290005090
C	0.4505252336	1.3678731647	-0.1326131301
C	-0.3615684843	0.1164705115	-0.4298313976
C	0.3116205630	-1.0877503029	0.2088030134
C	1.6985855814	-1.2775095486	-0.3607920383
H	0.4625948093	1.5622902327	0.9492016836
H	1.8573121920	0.9960729858	-1.7244757079
H	0.4073997568	-0.9399359475	1.2952859013
H	1.6775617372	-1.4160149194	-1.4545923074
H	-0.3525290873	-0.0205024644	-1.5275631322
O	2.4247609645	-0.0907086183	-0.0467366189
O	2.3286244858	-2.3507061837	0.2587655506
O	-0.5813477853	-2.1444180526	-0.0812864889
O	-0.0055401348	2.5095172626	-0.8395254381
H	-0.9154855221	2.7106445718	-0.5441985155
C	3.5424805599	-2.7426879781	-0.3836684306
H	3.9008116732	-3.6298204422	0.1383167295
H	3.3520735966	-2.9869257791	-1.4344940552
H	4.2946735777	-1.9516908983	-0.3177062705
C	-1.9317887116	-1.7714684630	0.1799131806
O	-2.7498233592	-2.1352149654	-0.6947151023
C	-2.3005193062	-1.8698263761	1.6430320513
H	-1.5039694154	-1.5216567402	2.3021731822
H	-3.2078933271	-1.2911318825	1.8281506283
H	-2.5051445504	-2.9231184131	1.8594740946
O	-1.6735819882	0.0363168346	0.0549669362
O	-3.1902280590	0.3566223888	-2.2246240668
H	-2.6799020743	0.1957672463	-1.3998679164
H	-3.4563542218	-0.5300763115	-2.5005668286
O	-2.6908818543	2.6528254477	0.0277597219
H	-2.5752050234	1.6843311072	0.0936053788
H	-3.2444860078	2.7884139977	-0.7530649372
H	-1.6383595583	0.6877056186	1.7562970240
O	-1.6285370133	1.1150265834	2.6401814017
H	-1.4164259664	2.0404638901	2.4608132406
H	2.5414631073	1.9092655936	-0.3540297677

beta-D-Xyl-TS1b
-1 1

C	-1.2351519522	2.5635968932	0.1304702849
C	0.2828373982	2.3447707820	0.0411156046

C	0.5236247358	0.9437000804	0.5490496051
C	-0.2697142328	-0.0705815021	-0.2625683976
C	-1.7443519985	0.2389984593	-0.0680612644
H	0.6163212686	2.4375334546	-1.0005331880
H	-1.5280925118	2.5722980797	1.1903016939
H	-0.0553345424	0.0828339305	-1.3368043716
H	-2.0336321107	0.2076181052	0.9964425383
H	0.2261349876	0.8945749857	1.6072049273
O	-1.9638463424	1.5591133174	-0.5712880991
O	-2.5154472986	-0.6472896874	-0.8100952750
O	0.1867255760	-1.3116538204	0.1663404209
O	1.8586128462	0.4652735535	0.4201366851
O	0.8835527467	3.3438709735	0.8496656306
H	1.8438506355	3.2872520114	0.7464905021
C	-3.8964378379	-0.6368312377	-0.4483814698
H	-4.3899491243	-1.3861041911	-1.0672963329
H	-4.0123148937	-0.8994780741	0.6095904628
H	-4.3441431043	0.3438082614	-0.6347884478
C	1.9049976812	-0.8849378572	0.8642635135
O	1.8307152502	-1.0951588103	2.0879765688
C	2.7685871834	-1.7239601861	-0.0386083928
H	2.6175904152	-2.7781036828	0.2004772182
H	2.5439154875	-1.5463944549	-1.0911927701
H	3.8155406720	-1.4669174578	0.1549563640
O	0.2322965521	-2.7926671869	-2.0185884937
H	0.2250908803	-2.2624516993	-1.1737521659
O	-1.3518901086	-2.2568124854	2.1535488424
H	-0.7642301570	-1.9033843491	1.4365674463
H	-2.2461743914	-2.0003999081	1.8921296289
O	2.6723799494	0.9840360516	-2.3031615478
H	2.7506544698	0.1262085065	-2.7401195317
H	2.4056270528	0.7758210806	-1.3899266754
H	-1.5066200523	3.5187180571	-0.3210412531
H	-0.6825631293	-2.7835199930	-2.3268422608

beta-D-Xyl-TS2a
-1 1

C	0.6244487516	-1.5131748535	-0.4354087267
C	-0.2567350838	-0.2783029264	-0.5626290250
C	0.4683862792	0.8911981940	0.0747023653
C	1.7703239013	1.1790654291	-0.6369811759
C	2.5752926253	-0.1263312196	-0.5845895968
H	-0.3811837532	-0.0739406445	-1.6399873699
H	0.8351293209	-1.7505401835	0.6167061142
H	1.5883815445	1.4432681354	-1.6872007516
H	2.8360874986	-0.3601248394	0.4625129291
H	0.6888705438	0.6515901202	1.1280134814
O	1.8414310470	-1.2143799478	-1.1317731542
O	3.7265694026	0.0366255018	-1.3471265988
O	2.5434830223	2.1753499795	0.0093476011
O	-0.4815786305	1.9382545690	0.0032969751
C	4.7046918501	-0.9795616431	-1.1263599304
H	5.5870682396	-0.6944655761	-1.6993482003
H	4.9591474932	-1.0343640363	-0.0621301942
H	4.3457876650	-1.9532141309	-1.4711719657
H	2.0903409094	3.0244724337	-0.0891418083
C	-1.7697498677	1.4850457716	0.4180220761

O	-2.7167541110	1.9004212568	-0.2910593844
C	-1.9237486852	1.4397898167	1.9225237592
H	-1.0357708888	1.0520647274	2.4241871418
H	-2.1079701832	2.4620789844	2.2673330763
H	-2.7860840791	0.8212872209	2.1804823798
O	-1.4970122818	-0.2749766403	0.0852470766
O	-3.2136286384	-0.4535564701	-2.1155779356
H	-3.4638606917	0.4220211452	-2.4371669765
H	-2.6793850241	-0.2762392195	-1.3129277976
H	-2.3338546253	-1.9274975554	-0.0423859484
O	-2.7367210702	-2.8062215159	-0.1825616405
H	-3.2093086990	-2.7202817404	-1.0211663185
H	-1.2844365745	-1.1119499691	1.6803569159
O	-1.1545996233	-1.6515049460	2.4904281536
H	-0.8703220452	-2.5150282986	2.1630831048
H	0.1945906314	-2.3872566391	-0.9270018009

beta-D-Xyl-TS2b
-1 1

C	1.4945517106	-2.0585693764	-0.1449128192
C	0.1136037946	-1.4717085242	-0.3427992588
C	0.0661503014	-0.0584768984	0.2092035788
C	1.0595729145	0.8154523451	-0.5461852208
C	2.4263305755	0.1330878010	-0.4155892765
H	-0.1237326049	-1.4680815018	-1.4167934733
H	1.7671611488	-2.0801119229	0.9191069692
H	0.7927203706	0.8715248322	-1.6118099155
H	2.7458770160	0.1387050252	0.6413167254
H	0.3999313085	-0.0935828969	1.2640815888
O	2.3881032585	-1.2137703595	-0.8762903852
O	3.3457428576	0.8192445437	-1.2030371531
O	1.1834701560	2.1122936033	0.0100934869
O	-0.9201999958	-2.1181600543	0.3807925207
C	4.7026651224	0.4856453053	-0.9130401088
H	5.3236494818	1.1269213377	-1.5385072486
H	4.9224422388	0.6788610937	0.1428623666
H	4.9102775600	-0.5622225213	-1.1473852916
H	0.3525751948	2.5918733872	-0.1777070901
C	-2.1213776912	-1.3632946605	0.2590253953
O	-2.7806350792	-1.4914596699	-0.7968487000
C	-2.8096492553	-1.2573710488	1.5967653148
H	-2.1061419605	-1.0029205838	2.3911291245
H	-3.6011847581	-0.5077177652	1.5401661902
H	-3.2636354912	-2.2285159973	1.8204188421
O	-1.2894806188	0.2863818225	0.1492003899
H	-1.6255448898	2.0958908472	-0.3188945467
H	-1.6831626430	0.4696665645	-1.6383572660
H	-1.5584821322	1.1931994420	1.6112667448
O	-1.4157264656	3.0058882721	-0.6072307503
O	-1.8427068725	0.6808744938	-2.5833956222
O	-1.6259881390	1.7947235840	2.3902613380
H	-1.4970911953	2.9758919535	-1.5709178966
H	-2.3217356199	-0.0897205684	-2.9149739977
H	-1.9191278396	1.2371025500	3.1214762891
H	1.5962653110	-3.0589696145	-0.5672939145

References

[1] X.-A. Lu, C.-H. Chou, C.-C. Wang, S.-C. Hung, *Synlett* **2003**, 1364–1366.

[2] P. L. Barili, G. Berti, D. Bertozzi, G. Catelani, F. Colonna, T. Corsetti, F. D'Andrea, *Tetrahedron* **1990**, *46*, 5365–5376.

[3] R. Lassfolk, J. Rahkila, M. P. Johansson, F. S. Ekholm, J. Wärnå, R. Leino, *J. Am. Chem. Soc.* **2019**, *141*, 1646–1654.

[4] R. F. Helm, J. Ralph, L. Anderson, *J. Org. Chem.* **1991**, *56*, 7015–7021.

[5] N. Morishima, S. Koto, C. Kusuhara, S. Zen, *Bull. Chem. Soc. Jpn.* **1982**, *55*, 631–632.

[6] N. A. Hughes, C. D. Maycock, *Carbohydr. Res.* **1974**, *35*, 247–250.

[7] V. Popsavin, G. Benedeković, M. Popsavin, V. Divjaković, T. Armbruster, *Tetrahedron* **2004**, *60*, 5225–5235.

[8] A. Lipták, J. Imre, P. Nánási, *Carbohydr. Res.* **1981**, *92*, 154–156.

[9] O. K. Kwang-dae, Y. Takagi, T. Tsuchiya, S. Umezawa, H. Umezawa, *Carbohydr. Res.* **1987**, *169*, 69–81.

[10] ChemAdder/SpinAdder, *Spin Discov. Ltd*, (http://www.chemadder.com).

[11] H. Haario, *Modest 6.0- A Users's Guide*, ProMath, Helsinki, **2001**.

[12] Gaussian 09, Revision, M. J. Frisch, G. W. Trucks, H. B. Schlegel, G. E. Scuseria, M. A. Robb, J. R. Cheeseman, V. Scalmani, G.; Barone, B. Mennucci, G. A. Petersson, H. Nakatsuji, M. Caricato, X. Li, H. P. Hratchian, A. F. Izmaylov, J. Bloino, G. Zheng, J. L. Sonnenberg, M. Hada, M. Ehara, K. Toyota, R. Fukuda, J. Hasegawa, M. Ishida, T. Nakajima, Y. Honda, O. Kitao, H. Nakai, T. Vreven, J. J. A. Montgomery, J. E. Peralta, F. Ogliaro, M. Bearpark, J. J. Heyd, E. Brothers, K. N. Kudin, V. N. Staroverov, R. Kobayashi, J. Normand, K. Raghavachari, A. Rendell, J. C. Burant, S. S. Iyengar, J. Tomasi, M. Cossi, N. Rega, J. M. Millam, M. Klene, J. E. Knox, J. B. Cross, V. Bakken, C. Adamo, J. Jaramillo, R. Gomperts, R. E. Stratmann, O. Yazyev, A. J. Austin, R. Cammi, C. Pomelli, J. W. Ochterski, R. L. Martin, K. Morokuma, V. G. Zakrzewski, G. A. Voth, P. Salvador, J. J. Dannenberg, S. Dapprich, A. D. Daniels, Ö. Farkas, J. B. Foresman, J. V. Ortiz, J. Cioslowski, D. J. Fox, Gaussian, Inc., Wallingford CT, **2009**.

[13] J. Tao, J. P. Perdew, V. N. Staroverov, G. E. Scuseria, *Phys. Rev. Lett.* **2003**, *91*, 146401.

[14] V. N. Staroverov, G. E. Scuseria, J. Tao, J. P. Perdew, *J. Chem. Phys.* **2003**, *119*, 12129–12137.

[15] S. Grimme, S. Ehrlich, L. Goerigk, *J. Comput. Chem.* **2011**, *32*, 1456–1465.

[16] R. Ditchfield, W. J. Hehre, J. A. Pople, *J. Chem. Phys.* **1971**, *54*, 724–728.

[17] W. J. Hehre, R. Ditchfield, J. A. Pople, *J. Chem. Phys.* **1972**, *56*, 2257–2261.

[18] F. Weigend, R. Ahlrichs, *Phys. Chem. Chem. Phys.* **2005**, *7*, 3297–3305.

[19] F. Weigend, *Phys. Chem. Chem. Phys.* **2006**, *8*, 1057–1065.

[20] V. Barone, M. Cossi, *J. Phys. Chem. A* **1998**, *102*, 1995–2001.

[21] M. Cossi, N. Rega, G. Scalmani, V. Barone, *J. Comput. Chem.* **2003**, *24*, 669–681.

[22] J.-D. Chai, M. Head-Gordon, *Phys. Chem. Chem. Phys.* **2008**, *10*, 6615–6620.

[23] M. Walker, A. J. A. Harvey, A. Sen, C. E. H. Dessent, *J. Phys. Chem. A* **2013**, *117*, 12590–12600.

[24] A. V Marenich, C. J. Cramer, D. G. Truhlar, *J. Phys. Chem. B* **2009**, *113*, 6378–6396.

[25] T. H. Dunning, *J. Chem. Phys.* **1989**, *90*, 1007–1023.

[26] D. E. Woon, T. H. Dunning, *J. Chem. Phys.* **1993**, *98*, 1358–1371.

[27] H. B. Schlegel, *J. Comput. Chem.* **1982**, *3*, 214–218.

[28] K. Fukui, *J. Phys. Chem.* **1970**, *74*, 4161–4163.

[29] A. N. Bootsma, S. E. Wheeler, *ChemRxiv.* **2019**. This content is a preprint and has not been peer-reviewed; DOI: 10.26434/chemrxiv.8864204.v5

[30] B. C. Garrett, D. G. Truhlar, R. S. Grev, A. W. Magnuson, *J. Phys. Chem.* **1980**, *84*, 1730–1748.

[31] C. Alhambra, J. Corchado, M. L. Sánchez, M. Garcia-Viloca, J. Gao, D. G. Truhlar, *J. Phys. Chem. B* **2001**, *105*, 11326–11340.

[32] E. Dzib, J. L. Cabellos, F. Ortíz-Chi, S. Pan, A. Galano, G. Merino, *Int. J. Quantum Chem.* **2019**, *119*, e25686.

[33] Schrödinger Release 2020-1: MacroModel, Schrödinger, LLC, New York, NY, **2021**.

[34] O. Corcoran, R. W. Mortensen, S. H. Hansen, J. Troke, J. K. Nicholson, *Chem. Res. Toxicol.* **2001**, *14*, 1363–1370.

[35] M. A. Rangelov, G. N. Vayssilov, D. D. Petkov, *Int. J. Quantum Chem.* **2006**, *106*, 1346–1356.

[36] D. Lim, A. J. Fairbanks, *Chem. Sci.* **2017**, *8*, 1896–1900.

[37] B. Thapa, H. B. Schlegel, *J. Phys. Chem. A* **2016**, *120*, 5726–5735.

[38] S. Kannath, P. Adamczyk, D. Ferro-Costas, A. Fernández-Ramos, D. T. Major, A. Dybala-Defratyka, *J. Chem. Theory Comput.* **2020**, *16*, 847–859.

[39] Y. Zheng, W. Zheng, D. Zhu, H. Chang, *New J. Chem.* **2019**, *43*, 5239–5254.

[40] M. D. Liptak, G. C. Shields, *J. Am. Chem. Soc.* **2001**, *123*, 7314–7319.

[41] S. Feng, C. Bagia, G. Mpourmpakis, *J. Phys. Chem. A* **2013**, *117*, 5211–5219.

[42] D. M. Camaioni, C. A. Schwerdtfeger, *J. Phys. Chem. A* **2005**, *109*, 10795–10797.

[43] A. A. Isse, A. Gennaro, *J. Phys. Chem. B* **2010**, *114*, 7894–7899.

[44] A. V Marenich, J. Ho, M. L. Coote, C. J. Cramer, D. G. Truhlar, *Phys. Chem. Chem. Phys.* **2014**, *16*, 15068–15106.

[45] D. A. Case, H. M. Aktulga, K. Belfon, I. Y. Ben-Shalom, S. R. Brozell, D. S. Cerutti, T. E. I. Cheatham, G. A. Cisneros, V. W. D. Cruzeiro, T. A. Darden, R. E. Duke, G. Giambasu, M. K. Gilson, H. Gohlke, A. W. Goetz, R. Harris, S. Izadi, S. A. Izmailov, C. Jin, K. Kasavajhala, M. C. Kaymak, E. King, A. Kovalenko, T. Kurtzman, T. S. Lee, S. LeGrand, P. Li, C. Lin, J. Liu, T. Luchko, R. Luo, M. Machado, V. Man, M. Manathunga, K. M. Merz, Y. Miao, O. Mikhailovskii, G. Monard, H. Nguyen, K. A. O'Hearn, A. Onufriev, F. Pan, S. Pantano, R. Qi, A. Rahnamoun, D. R. Roe, A. Roitberg, C. Sagui, S. Schott-Verdugo, J. Shen, C. L. Simmerling, N. R. Skrynnikov, J. Smith, J. Swails, R. C. Walker, J. Wang, H. Wei, R. M. Wolf, X. Wu, Y. Xue, D. M. York, S. Zhao, P. A. Kollman, *Univ. California, San Fr.* **2020**.
